# Supplementary material for: Neurons dispose of hyperactive kinesin into glial cells for clearance
Source: EMBO J. 2024 May 28;43(13):5. doi: 10.1038/s44318-024-00118-0 (PMC11217292; doi:10.1038/s44318-024-00118-0)
Supplement: Supplementary file 1 — Appendix [file 44318_2024_118_MOESM1_ESM.pdf]

Appendix

Appendix figure S1: page 2-3

Appendix figure S2: page 4-5

Appendix table S1: page 6

Appendix table S2: page 7-11

Appendix table S3: page 12-15

Appendix Figure S1

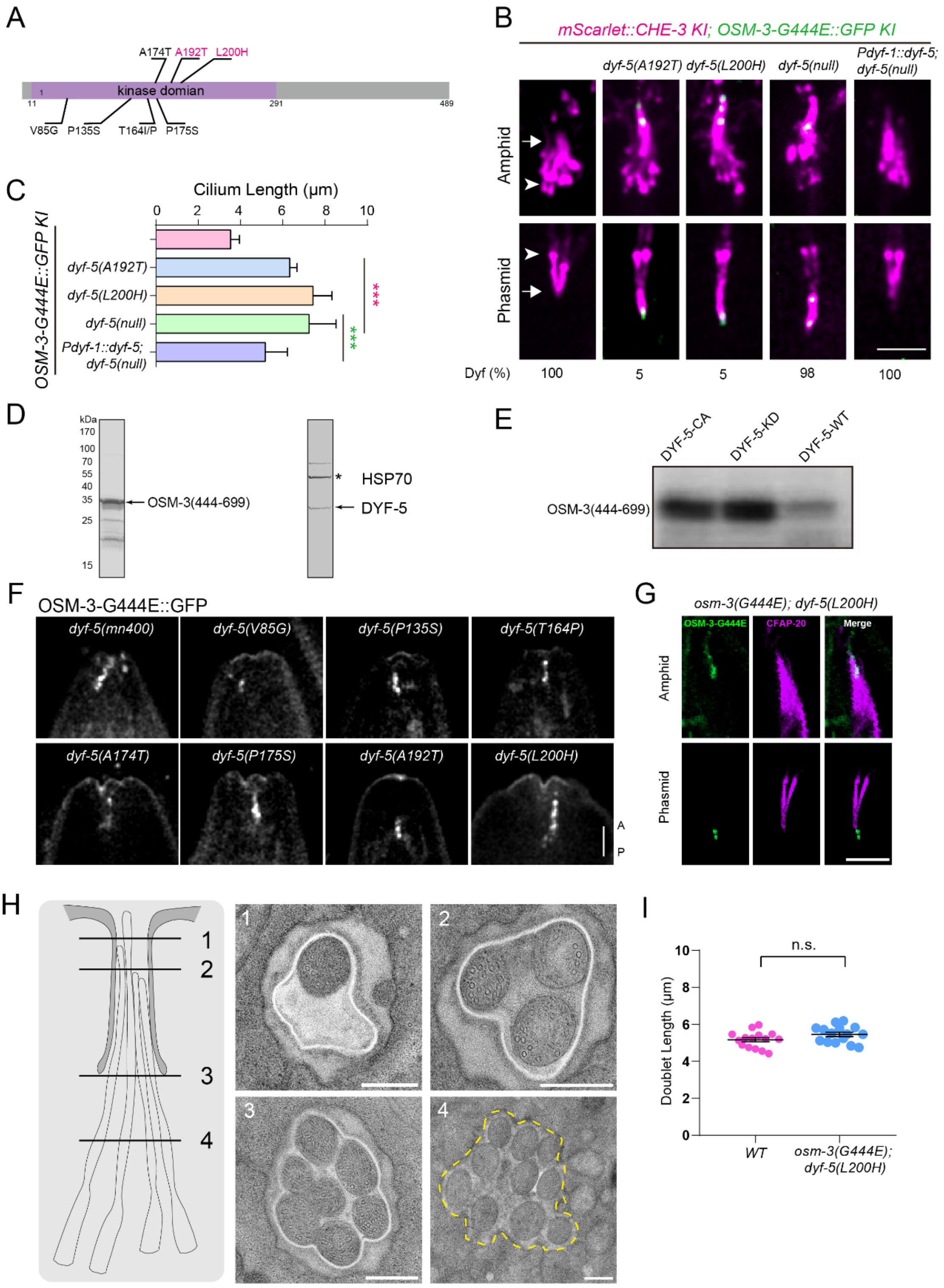

Appendix Figure S1. DYF-5 mutations rescue ciliary phenotypes in *osm-3(G444E)* mutant and partially restore the ciliary localization of OSM-3-G444E. **(A)** Schematic of the mutations on DYF-5 kinase domain that suppress the phenotypes in *osm-3(G444E)*. **(B)** Ciliary phenotypes and localizations of endogenous OSM-3-G444E in OSM-3-G444E::GFP KI, OSM-3-G444E::GFP KI; *dyf-5* double-mutant and DYF-5 transgenic animals. For the transgenic strain, the WT DYF-5 is expressed in *dyf-5* null allele mutant *dyf-5(mn400)* and OSM-3-G444E::GFP KI double-mutant animals under the control of P*dyf-1*. mScarlet-tagged endogenous CHE-3 fluorescence within cilia is shown in magenta. Arrowheads indicate the ciliary base. Arrows indicate the junctions between the middle and distal segments. Dyf, dye-filling defective; N ≥ 100. Scale bar, 5 μm. The control panel on the most left use a same figure from Fig. 1F. **(C)** Quantification of cilium length in the animals shown in (B). Pink asterisk represent comparisons between OSM-3-G444E::GFP KI and OSM-3-G444E::GFP KI; *dyf-5* double-mutant animals. Green asterisk represent comparison between OSM-3-G444E::GFP KI; *dyf-5(mn400)* double-mutant and DYF-5 transgenic animals. N = 34-53. ****P* < 0.001 by one way ANOVA using BH method to adjust P values. Data are mean ± S.D. **(D)** SDS-page showed the purified protein for autoradiography in (E). **(E)** Autoradiography testing the phosphorylation of OSM-3 (444-699) by DYF-5. Purified truncated OSM-3 (444-699) protein was treated with constitutively activated DYF-5 (DYF-5-CA), DYF-5 kinase domain (DYF-5-KD) and WT DYF-5 (DYF-5-WT) respectively and evaluated by radiograph. **(F)** The presence of endogenous OSM-3-G444E in amphid cilia in *dyf-5* mutants. A, anterior; P, posterior. Scale bar, 5 μm. **(G)** Representative images of OSM-3-G444E and doublet microtubule marker CFAP-20 in *dyf-5(L200H)* mutant. Scale bar, 5 μm. **(H)** Representative transmission electron microscopy (TEM) images of the sensory cilia of *osm-3(G444E); dyf-5(L200H)* double mutant worm. Left, schematic of four sensory cilia in amphid channel, the numbers indicate the approximate position of EM cross-sections. Right, EM cross-sections as indicated on the left, scale bar, 300 nm. **(I)** Quantification of doublet microtubule length indicated by CFAP-20 in *WT* and *osm-3(G444E); dyf-5(L200H)* double mutant worms, n.s., not significant by student t-test.

Appendix Figure S2

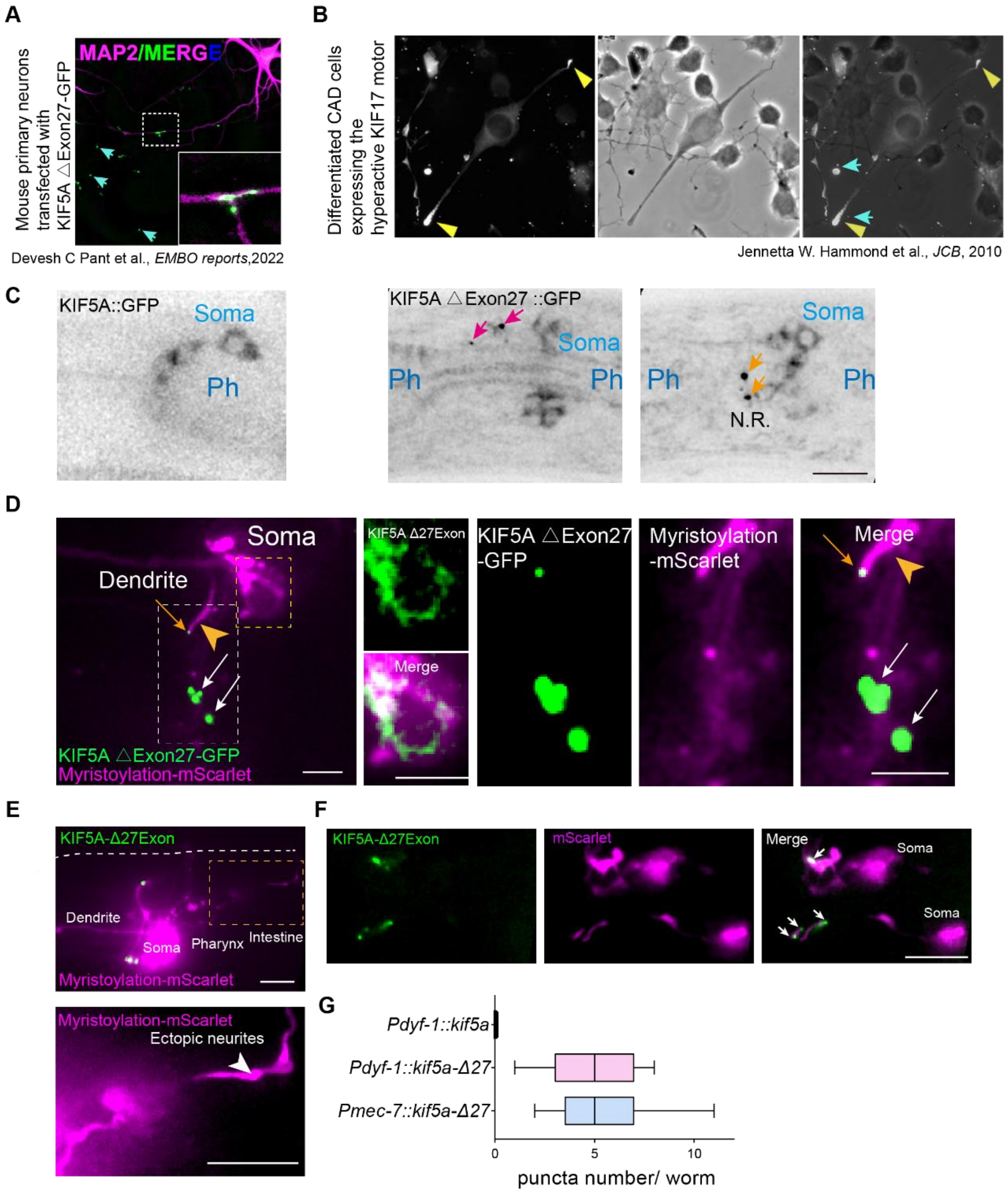

Appendix Figure S2. Hyperactive kinesins form puncta in different types of neurons. **(A-B)** Accumulation and potential disposal of other hyperactive kinesins in mammalian neurites. Figures modified from the papers cited below. The cyan arrows indicate the putative granules outside the cells and neurites. **(C)** Representative fluorescence images of the overexpressed hyperactive KIF5A that abnormally accumulates between two pharynxes like the hyperactive OSM-3 compared to the WT KIF5A. The arrows indicate the abnormal accumulation of hyperactive KIF5A at the tips of the ectopic neurites (magenta) and axons (orange). Scale bar, 10 μm. **(D)** Representative fluorescence image of the localization of KIF5A△Exon27 in sensory neurons. GFP-tagged 27th exon deleted mutant KIF5A (KIF5A△Exon27) and the membrane marker Myristoylation::mScarlet are expressed under the control of P*dyf-1*. The dashed boxes (yellow: soma; white: axon) are enlarged on the right. Orange arrowheads, an ectopic neurite; orange arrows, the KIF5A△Exon27 granules colocalized with axon; white arrows, the KIF5A△Exon27 puncta that does not colocalize with axon. Scale bars, 5 μm. **(E)** Representative fluorescence image of the ectopic neurite of the KIF5A△Exon27 overexpressed sensory neurons. GFP-tagged 27th exon deleted mutant KIF5A (KIF5A△Exon27) and the membrane marker Myristoylation::mScarlet are expressed under the control of P*dyf-1*. The dashed box is enlarged at the bottom. White dashed line, the periphery of the worm. Arrowhead, an ectopic neurite. Scale bars, 10 μm. **(F)** Representative fluorescence image of the localization of KIF5A△Exon27 in mechanosensory neurons. GFP-tagged 27th exon deleted mutant KIF5A (KIF5A△Exon27) and the red fluorescence protein mScarlet are expressed under the control of P*mec-7*. Arrows, the granule-like KIF5A△Exon27. Scale bars, 10 μm. **(G)** Statistics of puncta number of the strains in (D-F) as labeled.

Appendix Table S1
Targets of CRISPR and primers for molecular analysis.

**Appendix Table S1. Targets of CRISPR and primers for molecular analysis**		
**Gene**	**CRISPR-Cas9 targets (PAM)**	**Primers (For: forward; Rev: reverse)**
osm-3	sgRNA: GACGATCAACGAGGATCCAA **AGG**	For: GAAGGCATGACAGAAACGTTTTAGAGCTAGAAATAGC
		Rev: TCCTATTGCGAGATGTCTTGGTGGAAGGCATGACAGAA

Appendix Table S2.
Primers and plasmids used for plasmid cloning.

**Appendix Table S2. Primers and plasmids used for plasmid cloning**			
**Plasmid Name**	**Primer 5'**	**Primer 3'**	**Notes**
pDD162-*Peft-3::Cas9 + PU6::osm-3 knock-in sgRNA*	GAAGGCATGACAGAAACGTTTTAGAGCTAGAAATAGC	TCCTATTGCGAGATGTCTTGGTGGAAGGCATGACAGAA	PCR from pDD162-*Peft-3::Cas9+PU6::Empty sgRNA*
pPD95.77-*osm-3-5'arm-G444E-3'arm knock-in*	TCAGTTTATTGAAGGAGAAGAAGCAGG	CCTTCAATAAACTGATCTTGGAGCT	PCR from pPD95.77-*osm-3-5'arm-3'arm knock-in*
pPD95.77-*osm-3-5'arm-G235A-3'arm knock-in*	TTGGCTGCAAGTGAGCGGCAGAGC	CTCACTTGCAGCCAAATCGACCAG	PCR from pPD95.77-*osm-3-5'arm-3'arm knock-in*
pPD95.77-*osm-3-5'arm-G235A-G444E-3'arm knock-in*	TTGGCTGCAAGTGAGCGGCAGAGC	CTCACTTGCAGCCAAATCGACCAG	PCR from pPD95.77-*osm-3-5'arm-3'arm knock-in*
pPD95.77-*osm-3-5'arm-R238W-G444E -3'arm knock-in*	AGTGAGTGGCAGAGCAAGACAGG	GCTCTGCCACTCACTTCCAGCCAAATCG	PCR from pPD95.77-*osm-3-5'arm- G444E-3'arm knock-in*
pPD95.77-*osm-3-5'arm-H207Q-G444E -3'arm knock-in*	GAAGTCAGTCAATCTTCACGGTATACGTGG	AGATTGACTGACTTCGGGAAGAGTCCTGAA	PCR from pPD95.77-*osm-3-5'arm- G444E-3'arm knock-in*
pDONR-*Pdyf-1::osm-3::mScarlet*	GAGCCTCAGGAGCATCGATGGTCAGCAAGGGAGAGGCAGTTATCA	ATGCTCCTGAGGCTCCCGATGCTCCTTTGGGATTCAGAGAGGCTA	PCR from pDONR-*Pdyf-1::osm-3::gfp::mScarlet*
pDONR-*Phlh-17::mScarlet*	AGTGACCTGTTCGTTTTCGATTGACACATAGACACTTTGAAGCAG	TCCCTTGCTGACCATTGTAACGACAACTATTTCCCG	The *Phlh-17* sequences PCR from genomic DNA sequences

	ATGGTCAGCAAGGGAGAGG	AACGAACAGGTCACTATCAGTC	The *Phlh-17* sequences were cloned into pDONR-*mScarlet* via In-Fusion Advantage PCR Cloning Kit.
pDONR-*Phlh-17::ced-1*	GACAATTTGTACACCTAGGTCCAATTACTCTTCAACATCCC	GAGAATGAGACGCATTGTAACGACAACTATTTCCCG	The *ced-1* sequences were cloned into pDONR-*Phlh-17* via In-Fusion Advantage PCR Cloning Kit.
	ATGCGTCTCATTCTCCTTGTGC	CGTGCTCAGGACAATTTGTACACCTAG	*ced-1* cDNA sequences PCR from pDONR-*Pced-1-ced-1::mCherry*
pDONR-*PY37A1.B::ced-1*	AGTGACCTGTTCGTTGTTGCAGAAAAATATTTCACTGTTTCAC	GAGAATGAGACGCATTGGGATTTTTGATVTGCAAATATTGAC	The *PY37A1.B* sequences PCR from genomic DNA sequences
	ATGCGTCTCATTCTCCTTGTGC	AACGAACAGGTCACTATCAGTC	The *PY37A1.B* sequences were cloned into pDONR-*ced-1* via In-Fusion Advantage PCR Cloning Kit.
pDONR-*Pdyf-1::osm-3::gfp*	GCTTGTCAAAATGGCAGAGAGCGTCCGGGTCGCCGTAAGATGTCGTCC	CTTGTAGAGCTCGTCCATTCCGTGGGTGATTCCGGCGGCGGTG	*osm-3* gDNA sequence was amplified from N2
	GACGAGCTCTACAAGTAAGTCCAATTACTCTTCAACATCCCTACATGC	GCCATTTTGACAAGCTTACACAGAAATATAGCAAAATAGTGATAAG	*osm-3* gDNA was cloned into pDONR-*Pdyf-1- gfp* backbone via In-Fusion Advantage PCR Cloning Kit.
pDONR-*Pdyf-1::osm-3::gfp::mScarlet*	AAGCTTGTCAAAATGGCAGAGAGCGTCCGGGTCGCCGTAAGATGTCG	CTTCTCCTTTACTCATCGATGCTCCTGAGGCTCCCGATGCTCCTTTGGGATTCAG	mScarlet was amplified from pDONR- *mScarlet*
	TGAGTAAAGGAGAAGAATTGTTCACTGGAGTTGTCCCAATCCTCGTCG	CATTTTGACAAGCTTACACAGAAATATAGCAAAATAGTGATAAGTATC	mScarlet was cloned into pDONR-*Pdyf-1::osm-3::gfp*
pDONR-*Pdyf-1::mScarlet::osm-3::gfp*	TTGTCAAAATGGTCAGCAAGGGAGAGGCAGTTATCAAGGAGTTCATGC	CATACCGCTACCACTTCCAGCACCGCTACCACTTCCAGCCTTGTAGAGCTCGTCCATTCCTCCGG	mScarlet was amplified from pDONR- *mScarlet*

	AGTGGTAGCGGTATGGCAGAGAGCGTCCGGGTCGCCGTAAGATGTCG	TGACCATTTTGACAAGCTTACACAGAAATATAGCAAAATAGTGATAAG	mScarlet was cloned into pDONR-*Pdyf-1::osm-3::gfp*
pDONR-*Pdyf-1::mScarlet::osm-3-G444E::gfp*	GTTTATTGAAGGAGAAGAAGCAGGCAATACTC	TCTCCTTCAATAAACTGATCTTGGAGCTGTTG	PCR from pDONR-*Pdyf-1::mScarlet::osm-3::gfp*
pDONR-*Pdyf-1::mScarlet::osm-3-H207Q-G444E::gfp*	GAAGTCAGTCAATCTTCACGGTATACGTGG	AGATTGACTGACTTCGGGAAGAGTCCTGAA	PCR from pDONR-*Pdyf-1::mScarlet::osm-3-G444E::gfp*
pDONR-*Pdyf-1::mScarlet::osm-3-R238W-G444E::gfp*	AAGTGAGTGGCAGAGCAAGACAGGAGCCA	GCTCTGCCACTCACTTCCAGCCAAATCGAC	PCR from pDONR-*Pdyf-1::mScarlet::osm-3-G444E::gfp*
pDONR-*Pdyf-1::kif5a::gfp*	TGTAAGCTTGTCAAAATGGCGGAGACTAACAACG	GGCTCCCGATGCTCCGCTGGCTGCTGTCTCTTGG	*kif-5a* cDNA was amplified from addgene plasmid #127616
	GGAGCATCGGGAGCCTCA	TTTGACAAGCTTACACAGAAAT	*kif-5a* cDNA was cloned into pDONR-*Pdyf-1-gfp* backbone via In-Fusion Advantage PCR Cloning Kit.
pDONR-*Pdyf-1::kif5a △27 exon::gfp*	TGTAAGCTTGTCAAAATGGCGGAGACTAACAACGAATG	GGCTCCCGATGCTCCGAAACTGAAAGTGCAGGTATGCAGC	*kif5a △ 27 exon* sequence was modified from WT *kif5a*
	GGAGCATCGGGAGCCTCA	TTTGACAAGCTTACACAGAAAT	*kif5a △ 27 exon* sequence was cloned into pDONR-*Pdyf-1-gfp* backbone via In-Fusion Advantage PCR Cloning Kit.
pDONR-*Pmec-7::kif5a △ 27 exon::gfp*	AGTGACCTGTTCGTTGGTGGAGAACGTCATGAAATTAC	GTTAGTCTCCGCCATGTTGCTTGAAATTTGGACCCGAC	Promotor of *mec-7* was amplified from N2.
	ATGGCGGAGACTAACAACG	AACGAACAGGTCACTATCAGTC	*Pmec-7* was cloned into pDONR-*Pdyf-1::kif5a △ 27 exon::gfp* to replace *Pdyf-1*
pDONR-*Pdyf-1::kif5a(G235A)::gfp*	GCCGCTAGCGAGAAGGTCAGC	TTCTCGCTAGCGGCCAGATCC	PCR from pDONR-*Pdyf-1::kif5a::gfp*

pDONR-*Pdyf-1::kif5a(G235A)-△27 exon::gfp*	GCCGCTAGCGAGAAGGTCAGC	TTCTCGCTAGCGGCCAGATCC	PCR from pDONR-*Pdyf-1::kif5a△27 exon::gfp*
pDONR-*Pdyf-1::gfp::moesinABD*	-	-	pOG2074
pDONR-Pdyf-1::gfp::tsg-101	TAAGTCCAATTACTCTTCAACATCC	ACCGCTACCACTTCCAGC	PCR from pOG2074
	GGAAGTGGTAGCGGTATGTCGGCCCACCAGGTAC	GAGTAATTGGACTTAAATAGGAAGACCAGCAGTTCGTCG	*tsg-101* genomic DNA was cloned into pOG2074 via In-Fusion Advantage PCR Cloning Kit.
pDONR-*Pdyf-1::his-54::bfp*	TGTAAGCTTGTCAAAATGGCCCCACCAAAGCCG	GCTACCACTTCCAGCCTTGCTGGAGGTGTACTTGG	The *his-54* cDNA sequences were amplified from N2 and cloned into pDONR-*Pdyf-1::bfp* via In-Fusion Advantage PCR Cloning Kit.
pDONR-*Pdyf-1::Myristoylation::mScarlet*	GGTTCCTGTATTGGAAAAATGGTCAGCAAGGGAGAGGCAG	TCCAATACAGGAACCCATTTTGACAAGCTTACACAGAAATATAGC	PCR from pDONR-*Pdyf-1::mScarlet*
pET.M.3C-OSM-3-WT-eGFP-His6	TATGGCAGAGAGCGTCCGGGT	TGGACGAGCTGTACAAGTACC	*osm-3* cDNA was joined with eGFP and cloned into pET.M.3C backbone by restriction-ligation
pET.M.3C-OSM-3-G444E-eGFP-His6	ATCAGTTTATTGAAGGAGAAGAAGCAGGCAATACTCAG	TCTTCTCCTTCAATAAACTGATCTTGGAGCTGTTGGAT	PCR from pET.M.3C-OSM-3-WT-eGFP-His6
pET.M.3C-OSM-3-H207Q-eGFP-His6	CGAAGTCAGTCAATCTTCACGGTATACGTGG	AAGATTGACTGACTTCGGGAAGAGTCCTTGTT	PCR from pET.M.3C-OSM-3-WT-eGFP-His6
pET.M.3C-OSM-3-R238W-eGFP-His6	GAAGTGAGTGGCAGAGCAAGACAGGAGCCA	GCTCTGCCACTCACTTCCAGCCAAATCGAC	PCR from pET.M.3C-OSM-3-WT-eGFP-His6

pET.M.3C-OSM-3-H207Q-G444E-eGFP-His6	CGAAGTCAGTCAATCTTCACGGTATACGTGG	AAGATTGACTGACTTCGGGAAGAGTCCTTGTT	PCR from pET.M.3C-OSM-3-G444E-eGFP-His6
pET.M.3C-OSM-3-R238W-G444E-eGFP-His6	GAAGTGAGTGGCAGAGCAAGACAGGAGCCA	GCTCTGCCACTCACTTCCAGCCAAATCGAC	PCR from pET.M.3C-OSM-3-G444E-eGFP-His6

Appendix Table S3
***C. elegans* strains used in this study.**

**Appendix Table S3. *C. elegans* strains used in this study**			
**Strain name**	**Genotype**	**Method**	**Resource**
N2	Wild type	-	CGC
GJ3461	*p802(osm-3 null)*	-	CGC
-	*sa125[osm-3(G444E)]*	-	CGC
SYD0199	*osm-3::gfp knock-in*	-	From Yidong Shen lab
GOU5307	*cas1608[osm-3-G444E::gfp knock-in]*	Microinjection	This study
GOU5308	*cas1608[osm-3-G444E::gfp knock-in]; cas865[mScarlet::che-3 knock-in]*	Genetic cross	This study
GOU5309	*cas1608[osm-3-G444E::gfp knock-in]; casEx7010[*P*dyf-1:: osm-3::mScarlet; pRF4(+)]*	Microinjection	This study
GOU5310	*cas1608[osm-3-G444E::gfp knock-in]; casEx7011[*P*dyf-1:: Myri::mScarlet;* P*dyf-1::his-54::bfp; pRF4(+)]*	Microinjection	This study
GOU5311	*cas1608[osm-3-G444E::gfp knock-in]; casEx7012[*P*hlh-17:: mScarlet; pRF4(+)]*	Microinjection	This study
GOU5312	*cas1608[osm-3-G444E::gfp knock-in]; ced-1(e1735)*	Genetic cross	This study
GOU5313	*cas1608[osm-3-G444E::gfp knock-in]; ced-1(e1735); casEx7013[*P*ced-1::ced-1; pRF4(+)]*	Microinjection	This study
GOU5314	*cas1608[osm-3-G444E::gfp knock-in]; ced-1(e1735); casEx7014[*P*hlh-17::ced-1; pRF4(+)]*	Microinjection	This study
GOU5315	*cas1608[osm-3-G444E::gfp knock-in]; ced-1(e1735); casEx7015[*P*Y37A1.B::ced-1; pRF4(+)]*	Microinjection	This study

PHX5583	*syb5583[osm-3-H207Q::gfp knock-in]*	SunyBiotech company	This study
GOU5317	*cas2368[osm-3-R238W::gfp knock-in]*	Microinjection	This study
GOU5318	*cas1611[osm-3(H207Q-G444E)::gfp knock-in]*	Genetic Screen	This study
GOU5319	*cas1612[osm-3(R238W-G444E)::gfp knock-in]*	Genetic Screen	This study
GOU5320	*osm-3(p802); casEx7016[*P*dyf-1::osm-3::gfp; pRF4(+)]*	Microinjection	This study
GOU5321	*osm-3(p802); casEx7017[*P*dyf-1::osm-3::gfp::mScarlet; pRF4(+)]*	Microinjection	This study
GOU5322	*osm-3(p802); casEx7018[*P*dyf-1::mScarlet::osm-3::gfp; pRF4(+)]*	Microinjection	This study
GOU5323	*osm-3(p802); casEx7019[*P*dyf-1::mScarlet::osm-3-G444E::gfp; pRF4(+)]*	Microinjection	This study
GOU5324	*osm-3(p802); casEx7020[*P*dyf-1::mScarlet::osm-3-H207Q-G444E::gfp; pRF4(+)]*	Microinjection	This study
GOU5325	*osm-3(p802); casEx7021[*P*dyf-1::mScarlet::osm-3-R238W-G444E::gfp; pRF4(+)]*	Microinjection	This study
GOU5326	*cas2371[osm-3-G235A::gfp knock-in]*	Microinjection	This study
GOU5327	*cas2371[osm-3-G235A::gfp knock-in]; klp-11(tm324)*	Genetic cross	This study
GOU5328	*cas2372[osm-3-G235A-G444E::gfp knock-in]*	Microinjection	This study
GOU5329	*cas2372[osm-3-G235A-G444E::gfp knock-in]; casEx7011[*P*dyf-1::Myri::mScarlet;* P*dyf-1::his-54::bfp; pRF4(+)]*	Genetic cross	This study
GOU5330	*casEx7022[*P*dyf-1::kif5a::gfp;* P*dyf-1::Myri::mScarlet; pRF4(+)]*	Microinjection	This study
GOU5331	*casEx7023[*P*dyf-1::kif5a* △ *27exon::gfp;* P*dyf-1::Myri::mScarlet; pRF4(+)]*	Microinjection	This study
GOU5332	*cas11134[osm-3(H132Y-G444E)::gfp knock-in]*	Genetic Screen	This study

GOU5333	*cas1696[osm-3(E215K-G444E)::gfp knock-in]*	Genetic Screen	This study
GOU5334	*cas11157[osm-3(K250E-G444E)::gfp knock-in]*	Genetic Screen	This study
GOU5335	*cas1650[osm-3(T285I-G444E)::gfp knock-in]*	Genetic Screen	This study
GOU5336	*cas2375[osm-3-H207Q-G444E::gfp knock-in]*	Microinjection	This study
GOU5337	*cas2376[osm-3-R238W-G444E::gfp knock-in]*	Microinjection	This study
GOU5338	*cas11333[osm-3(H207Q-G445E)::gfp knock-in]*	Genetic Screen	This study
GOU5339	*cas1608[osm-3-G444E::gfp knock-in]; dyf-5(mn400)*	Genetic cross	This study
GOU5340	*cas1608[osm-3-G444E::gfp knock-in]; cas1622[dyf-5(V85G)]*	Genetic Screen	This study
GOU5341	*cas1608[osm-3-G444E::gfp knock-in]; cas1610[dyf-5(P135S)]*	Genetic Screen	This study
GOU5342	*cas1608[osm-3-G444E::gfp knock-in]; cas1651[dyf-5(T164P)]*	Genetic Screen	This study
GOU5343	*cas1608[osm-3-G444E::gfp knock-in]; cas1632[dyf-5(A174T)]*	Genetic Screen	This study
GOU5344	*cas1608[osm-3-G444E::gfp knock-in]; cas1628[dyf-5(P175S)]*	Genetic Screen	This study
GOU5345	*cas1608[osm-3-G444E::gfp knock-in]; cas1639[dyf-5(A192T)]*	Genetic Screen	This study
GOU5346	*cas1608[osm-3-G444E::gfp knock-in]; cas1644[dyf-5(L200H)]*	Genetic Screen	This study
GOU5347	*cas1608[osm-3-G444E::gfp knock-in]; cas865[mScarlet::che-3 knock-in]; cas1639[dyf-5(A192T)]*	Genetic cross	This study
GOU5348	*cas1608[osm-3-G444E::gfp knock-in]; cas865[mScarlet::che-3 knock-in]; cas1644[dyf-5(L200H)]*	Genetic cross	This study

GOU5349	*cas1608[osm-3-G444E::gfp knock-in]; cas865[mScarlet::che-3 knock-in]; dyf-5(mn400)*	Genetic cross	This study
GOU5350	*cas1608[osm-3-G444E::gfp knock-in]; cas865[mScarlet::che-3 knock-in]; dyf-5(mn400); casEx7024[*P*dyf-1::dyf-5; pRF4(+)]*	Genetic cross	This study
GOU5370	*casEx7025[*P*mec-7::kif5a △ 27exon::gfp;* P*mec-7:: mScarlet; pRF4(+)]*	Microinjection	This study
GOU5371	*casEx7026[Pdyf-1::kif5a-G235A-△27exon::gfp; Pdyf-1::Myri:: mScarlet; pRF4(+)]*	Microinjection	This study
GOU5372	*casEx7027[Pdyf-1::kif5a-G235A::gfp; Pdyf-1::Myri:: mScarlet; pRF4(+)]*	Microinjection	This study
PHX8212	*syb8212[osm-3-G444E-mScarlet knock-in]*	SunyBiotech company	This study
GOU5373	*casEx7028[Pdyf-1::gfp::moesinABD]; syb8212*	Microinjection	This study
GOU5374	*casEx7029[Pdyf-1::gfp::tsg-101]; syb8212*	Microinjection	This study
GOU5375	*cas1608[osm-3-G444E::gfp knock-in]; cas1644[dyf-5(L200H)]; casEx7030[Pdyf-1::cfap-20::Scarlet; pRF4(+)]*	Microinjection	This study
GOU5376	*syb8212; tbb-2::gfp11; che-3::t2a::gfp1-10*	Genetic cross	This study
