# Supplementary figures and images for: Neurons dispose of hyperactive kinesin into glial cells for clearance (part 2 of 9)
Source: EMBO J. 2024 May 28;43(13):5. doi: 10.1038/s44318-024-00118-0 (PMC11217292; doi:10.1038/s44318-024-00118-0)

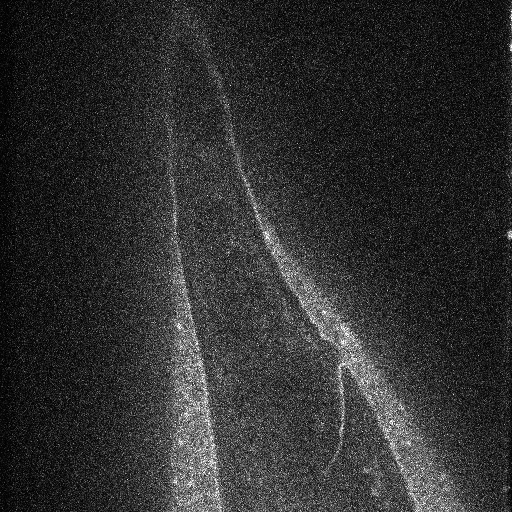

Supplement: Supplementary file 10 — Source data Fig. 1 [file 44318_2024_118_MOESM10_ESM.zip › Figure 1F Micr. image/20230324 Scarlet-che-3; osm-3-g444e-gfp_2 phasmid/img_000000000_L-488_015.tif]

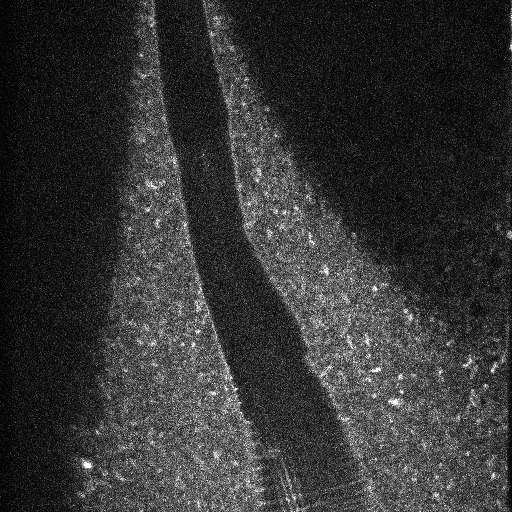

Supplement: Supplementary file 10 — Source data Fig. 1 [file 44318_2024_118_MOESM10_ESM.zip › Figure 1F Micr. image/20230324 Scarlet-che-3; osm-3-g444e-gfp_2 phasmid/img_000000000_L-488_003.tif]

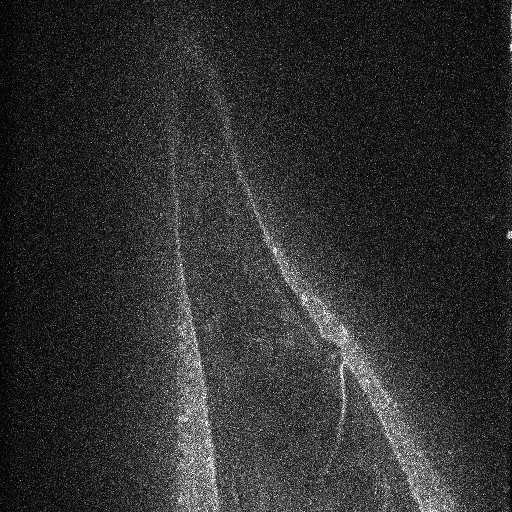

Supplement: Supplementary file 10 — Source data Fig. 1 [file 44318_2024_118_MOESM10_ESM.zip › Figure 1F Micr. image/20230324 Scarlet-che-3; osm-3-g444e-gfp_2 phasmid/img_000000000_L-488_017.tif]

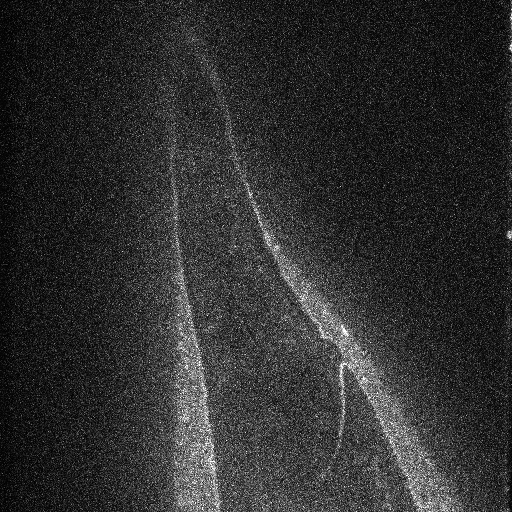

Supplement: Supplementary file 10 — Source data Fig. 1 [file 44318_2024_118_MOESM10_ESM.zip › Figure 1F Micr. image/20230324 Scarlet-che-3; osm-3-g444e-gfp_2 phasmid/img_000000000_L-488_016.tif]

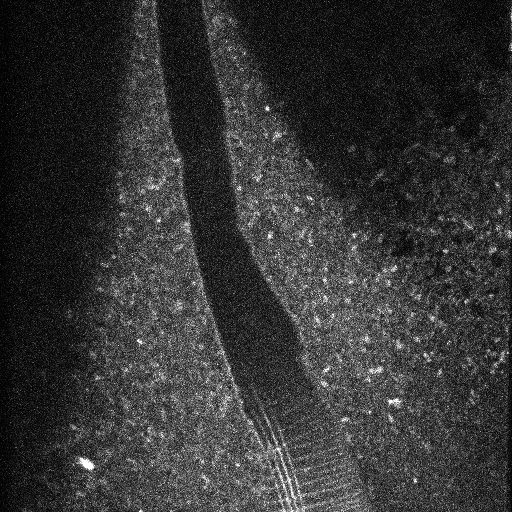

Supplement: Supplementary file 10 — Source data Fig. 1 [file 44318_2024_118_MOESM10_ESM.zip › Figure 1F Micr. image/20230324 Scarlet-che-3; osm-3-g444e-gfp_2 phasmid/img_000000000_L-488_002.tif]

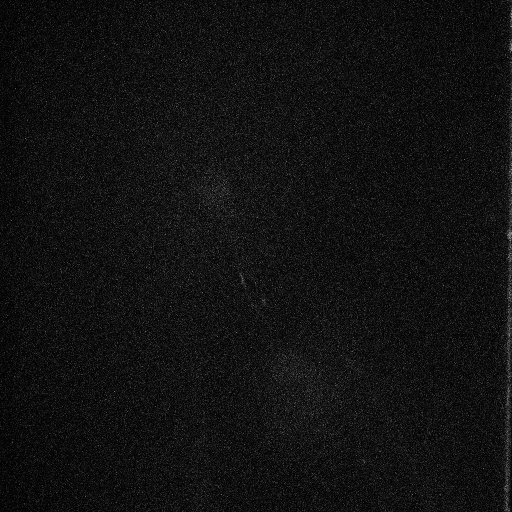

Supplement: Supplementary file 10 — Source data Fig. 1 [file 44318_2024_118_MOESM10_ESM.zip › Figure 1F Micr. image/20230324 Scarlet-che-3; osm-3-g444e-gfp_2 phasmid/img_000000000_L-561_024.tif]

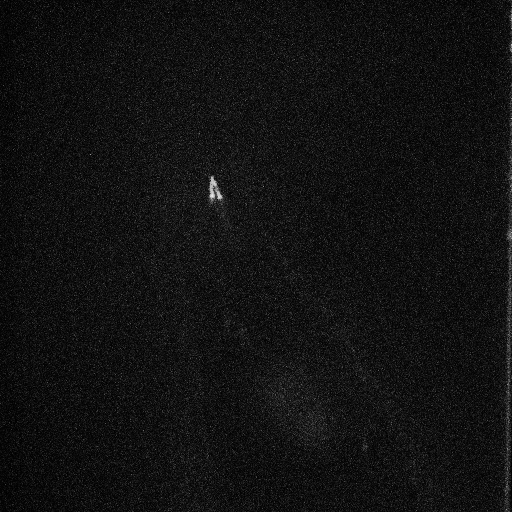

Supplement: Supplementary file 10 — Source data Fig. 1 [file 44318_2024_118_MOESM10_ESM.zip › Figure 1F Micr. image/20230324 Scarlet-che-3; osm-3-g444e-gfp_2 phasmid/img_000000000_L-561_018.tif]

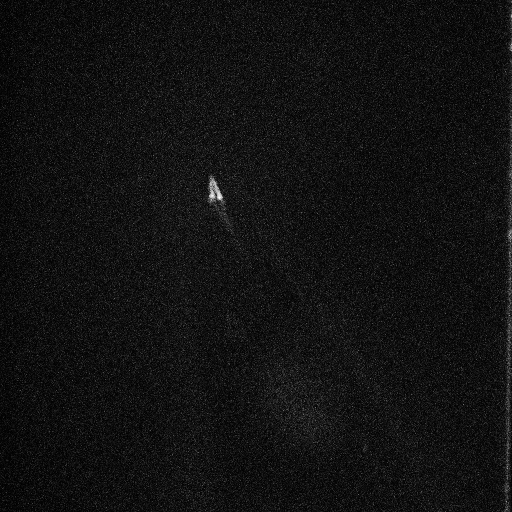

Supplement: Supplementary file 10 — Source data Fig. 1 [file 44318_2024_118_MOESM10_ESM.zip › Figure 1F Micr. image/20230324 Scarlet-che-3; osm-3-g444e-gfp_2 phasmid/img_000000000_L-561_019.tif]

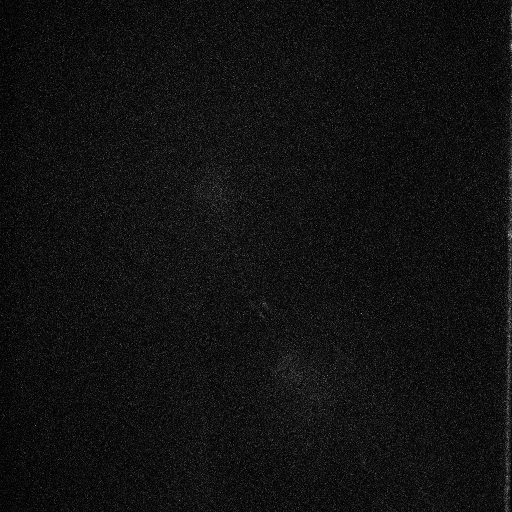

Supplement: Supplementary file 10 — Source data Fig. 1 [file 44318_2024_118_MOESM10_ESM.zip › Figure 1F Micr. image/20230324 Scarlet-che-3; osm-3-g444e-gfp_2 phasmid/img_000000000_L-561_025.tif]

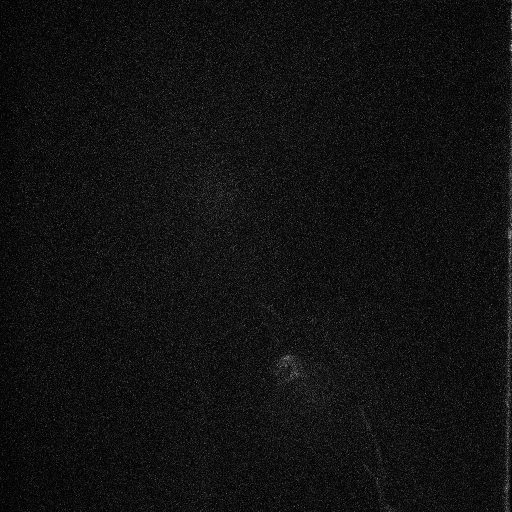

Supplement: Supplementary file 10 — Source data Fig. 1 [file 44318_2024_118_MOESM10_ESM.zip › Figure 1F Micr. image/20230324 Scarlet-che-3; osm-3-g444e-gfp_2 phasmid/img_000000000_L-561_027.tif]

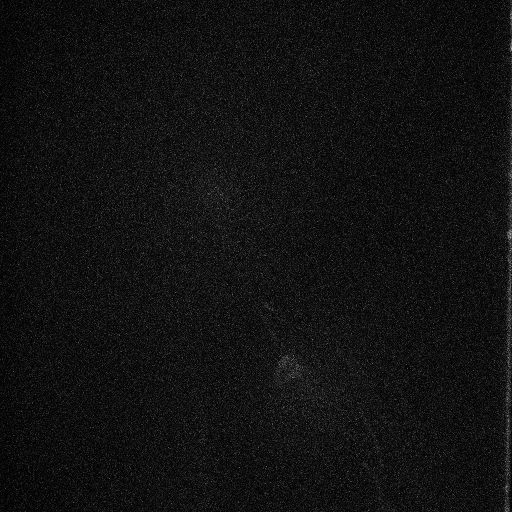

Supplement: Supplementary file 10 — Source data Fig. 1 [file 44318_2024_118_MOESM10_ESM.zip › Figure 1F Micr. image/20230324 Scarlet-che-3; osm-3-g444e-gfp_2 phasmid/img_000000000_L-561_026.tif]

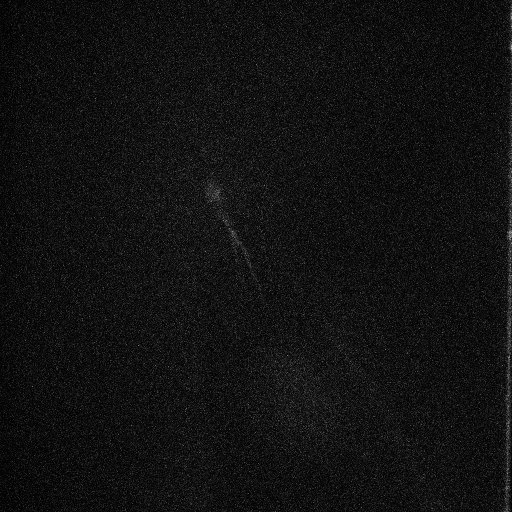

Supplement: Supplementary file 10 — Source data Fig. 1 [file 44318_2024_118_MOESM10_ESM.zip › Figure 1F Micr. image/20230324 Scarlet-che-3; osm-3-g444e-gfp_2 phasmid/img_000000000_L-561_022.tif]

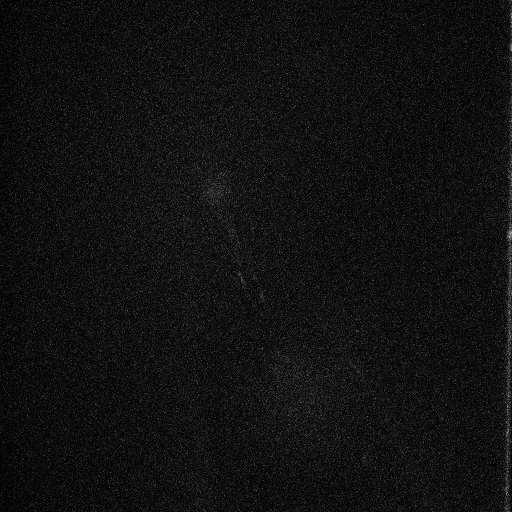

Supplement: Supplementary file 10 — Source data Fig. 1 [file 44318_2024_118_MOESM10_ESM.zip › Figure 1F Micr. image/20230324 Scarlet-che-3; osm-3-g444e-gfp_2 phasmid/img_000000000_L-561_023.tif]

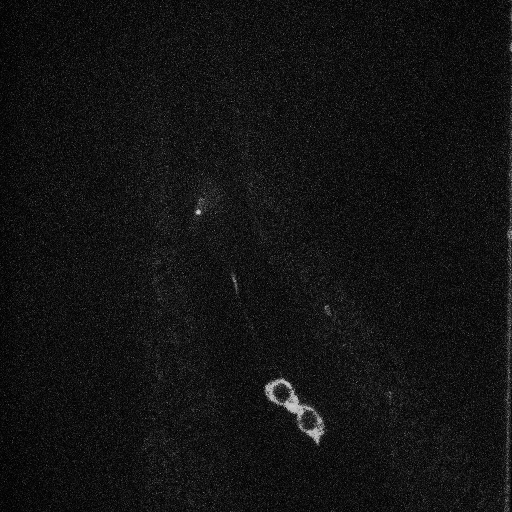

Supplement: Supplementary file 10 — Source data Fig. 1 [file 44318_2024_118_MOESM10_ESM.zip › Figure 1F Micr. image/20230324 Scarlet-che-3; osm-3-g444e-gfp_2 phasmid/img_000000000_L-561_009.tif]

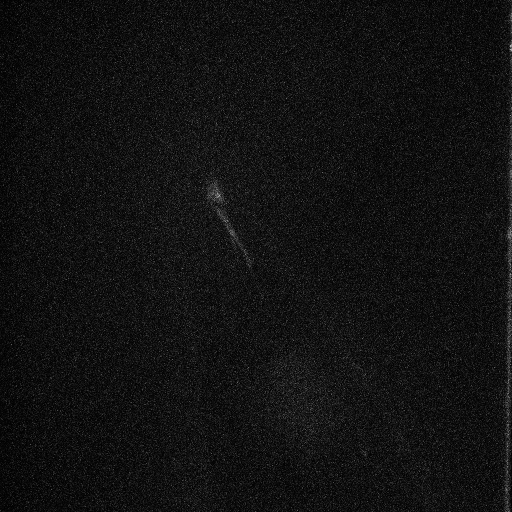

Supplement: Supplementary file 10 — Source data Fig. 1 [file 44318_2024_118_MOESM10_ESM.zip › Figure 1F Micr. image/20230324 Scarlet-che-3; osm-3-g444e-gfp_2 phasmid/img_000000000_L-561_021.tif]

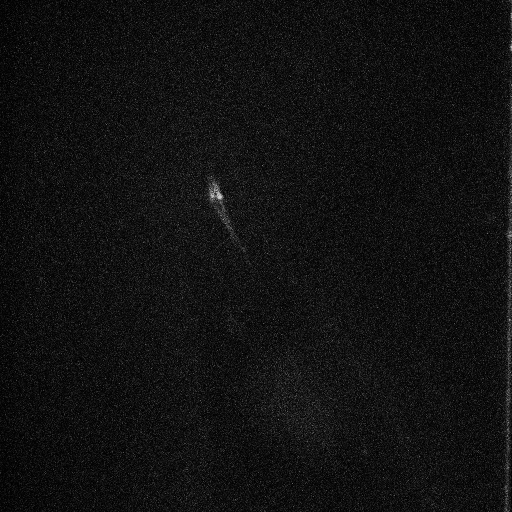

Supplement: Supplementary file 10 — Source data Fig. 1 [file 44318_2024_118_MOESM10_ESM.zip › Figure 1F Micr. image/20230324 Scarlet-che-3; osm-3-g444e-gfp_2 phasmid/img_000000000_L-561_020.tif]

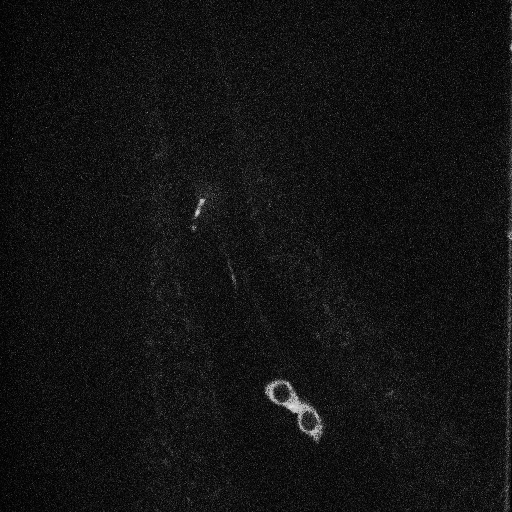

Supplement: Supplementary file 10 — Source data Fig. 1 [file 44318_2024_118_MOESM10_ESM.zip › Figure 1F Micr. image/20230324 Scarlet-che-3; osm-3-g444e-gfp_2 phasmid/img_000000000_L-561_008.tif]

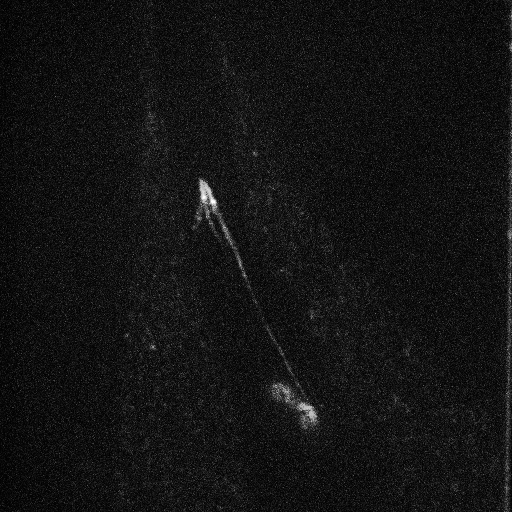

Supplement: Supplementary file 10 — Source data Fig. 1 [file 44318_2024_118_MOESM10_ESM.zip › Figure 1F Micr. image/20230324 Scarlet-che-3; osm-3-g444e-gfp_2 phasmid/img_000000000_L-561_005.tif]

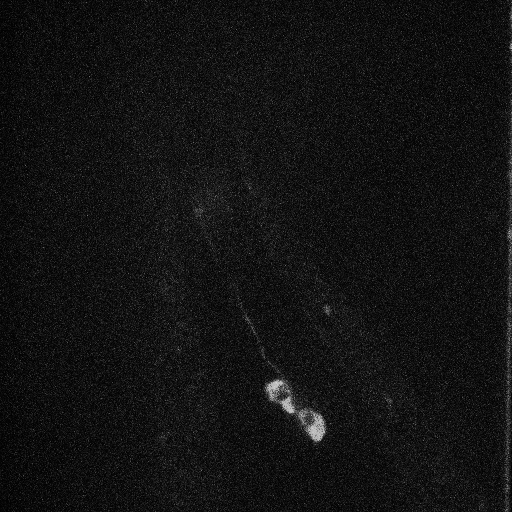

Supplement: Supplementary file 10 — Source data Fig. 1 [file 44318_2024_118_MOESM10_ESM.zip › Figure 1F Micr. image/20230324 Scarlet-che-3; osm-3-g444e-gfp_2 phasmid/img_000000000_L-561_011.tif]

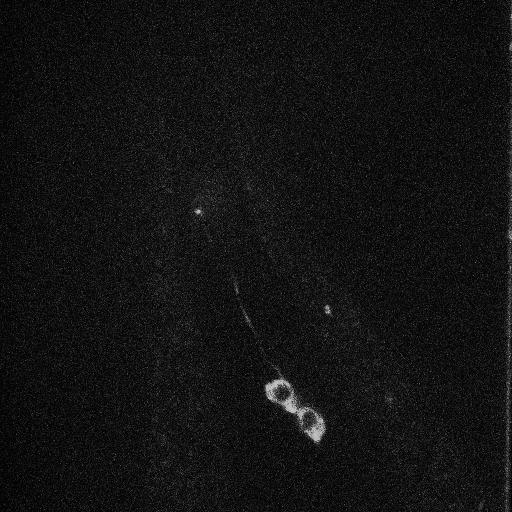

Supplement: Supplementary file 10 — Source data Fig. 1 [file 44318_2024_118_MOESM10_ESM.zip › Figure 1F Micr. image/20230324 Scarlet-che-3; osm-3-g444e-gfp_2 phasmid/img_000000000_L-561_010.tif]

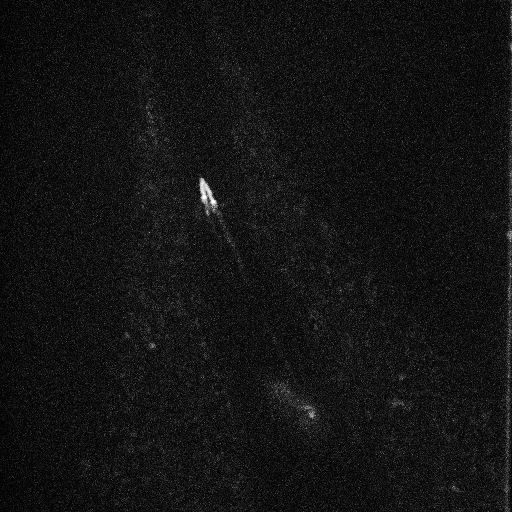

Supplement: Supplementary file 10 — Source data Fig. 1 [file 44318_2024_118_MOESM10_ESM.zip › Figure 1F Micr. image/20230324 Scarlet-che-3; osm-3-g444e-gfp_2 phasmid/img_000000000_L-561_004.tif]

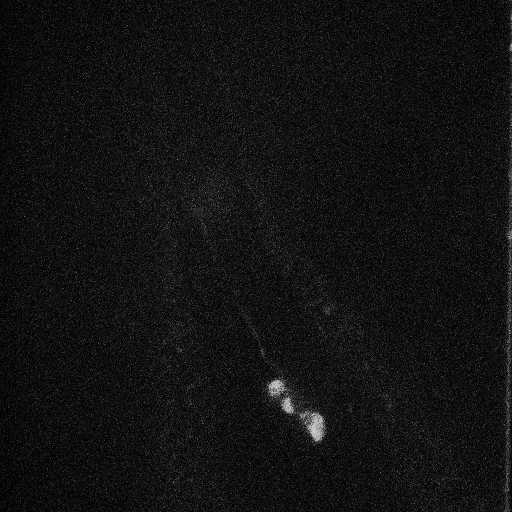

Supplement: Supplementary file 10 — Source data Fig. 1 [file 44318_2024_118_MOESM10_ESM.zip › Figure 1F Micr. image/20230324 Scarlet-che-3; osm-3-g444e-gfp_2 phasmid/img_000000000_L-561_012.tif]

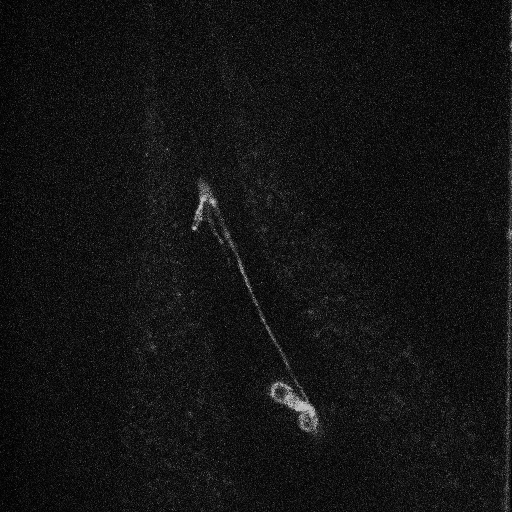

Supplement: Supplementary file 10 — Source data Fig. 1 [file 44318_2024_118_MOESM10_ESM.zip › Figure 1F Micr. image/20230324 Scarlet-che-3; osm-3-g444e-gfp_2 phasmid/img_000000000_L-561_006.tif]

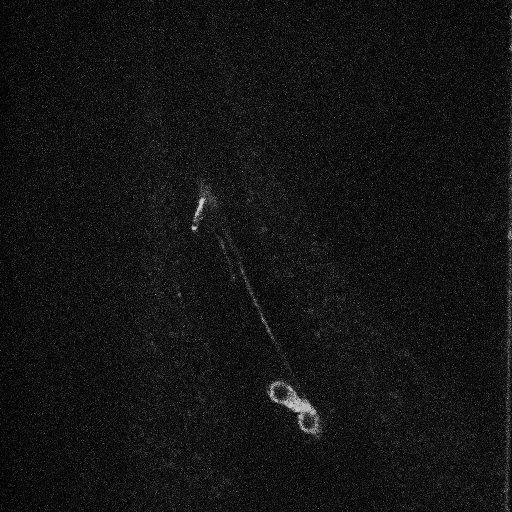

Supplement: Supplementary file 10 — Source data Fig. 1 [file 44318_2024_118_MOESM10_ESM.zip › Figure 1F Micr. image/20230324 Scarlet-che-3; osm-3-g444e-gfp_2 phasmid/img_000000000_L-561_007.tif]

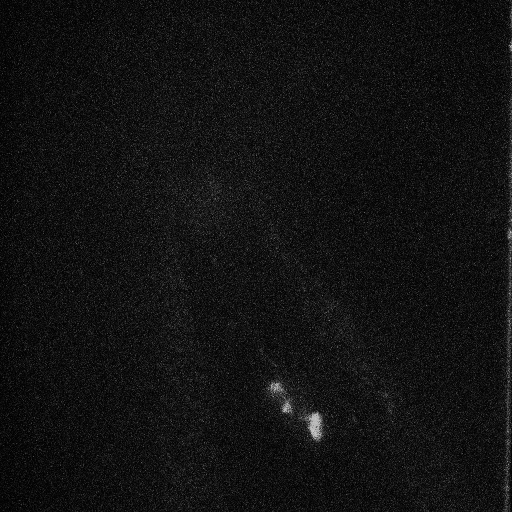

Supplement: Supplementary file 10 — Source data Fig. 1 [file 44318_2024_118_MOESM10_ESM.zip › Figure 1F Micr. image/20230324 Scarlet-che-3; osm-3-g444e-gfp_2 phasmid/img_000000000_L-561_013.tif]

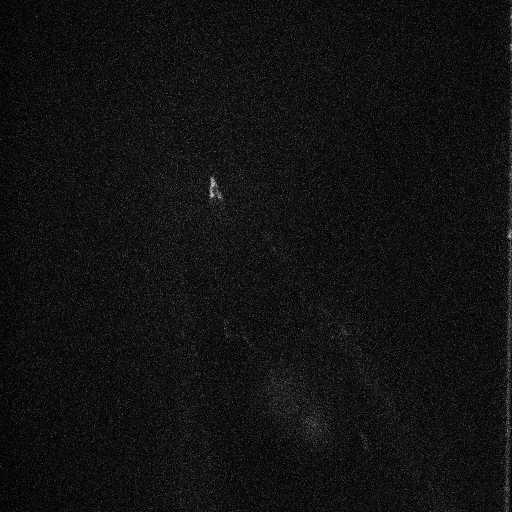

Supplement: Supplementary file 10 — Source data Fig. 1 [file 44318_2024_118_MOESM10_ESM.zip › Figure 1F Micr. image/20230324 Scarlet-che-3; osm-3-g444e-gfp_2 phasmid/img_000000000_L-561_017.tif]

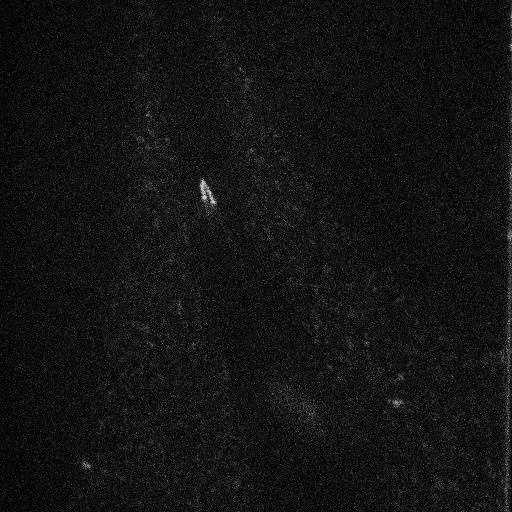

Supplement: Supplementary file 10 — Source data Fig. 1 [file 44318_2024_118_MOESM10_ESM.zip › Figure 1F Micr. image/20230324 Scarlet-che-3; osm-3-g444e-gfp_2 phasmid/img_000000000_L-561_003.tif]

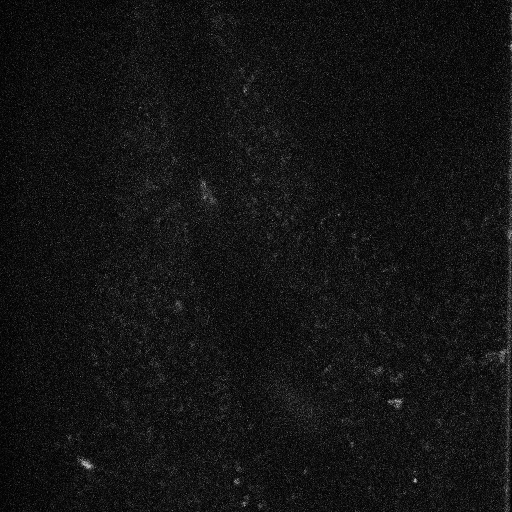

Supplement: Supplementary file 10 — Source data Fig. 1 [file 44318_2024_118_MOESM10_ESM.zip › Figure 1F Micr. image/20230324 Scarlet-che-3; osm-3-g444e-gfp_2 phasmid/img_000000000_L-561_002.tif]

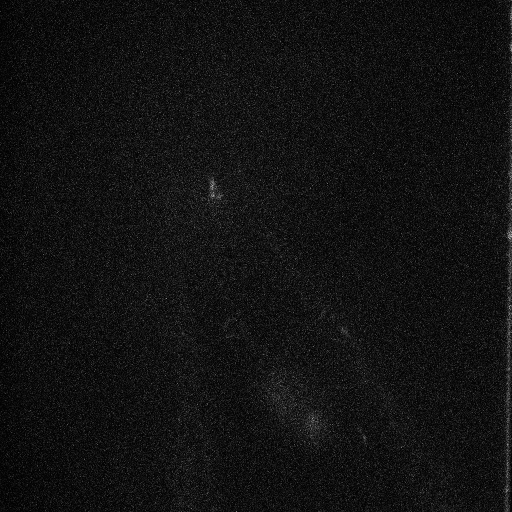

Supplement: Supplementary file 10 — Source data Fig. 1 [file 44318_2024_118_MOESM10_ESM.zip › Figure 1F Micr. image/20230324 Scarlet-che-3; osm-3-g444e-gfp_2 phasmid/img_000000000_L-561_016.tif]

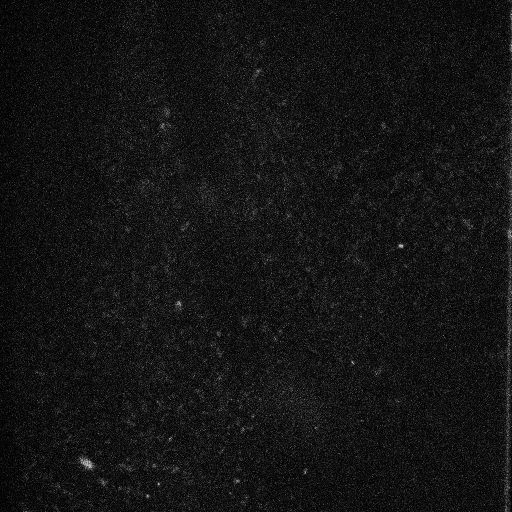

Supplement: Supplementary file 10 — Source data Fig. 1 [file 44318_2024_118_MOESM10_ESM.zip › Figure 1F Micr. image/20230324 Scarlet-che-3; osm-3-g444e-gfp_2 phasmid/img_000000000_L-561_000.tif]

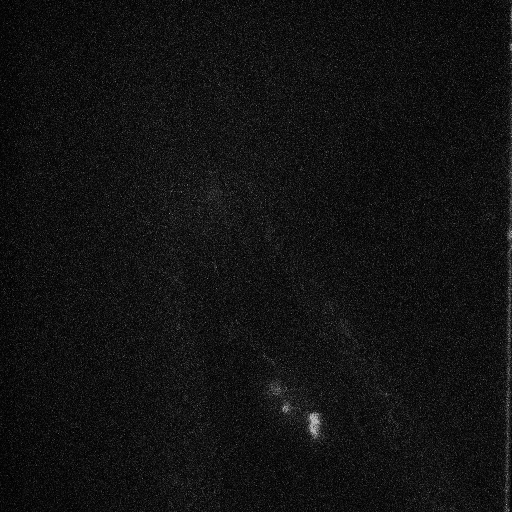

Supplement: Supplementary file 10 — Source data Fig. 1 [file 44318_2024_118_MOESM10_ESM.zip › Figure 1F Micr. image/20230324 Scarlet-che-3; osm-3-g444e-gfp_2 phasmid/img_000000000_L-561_014.tif]

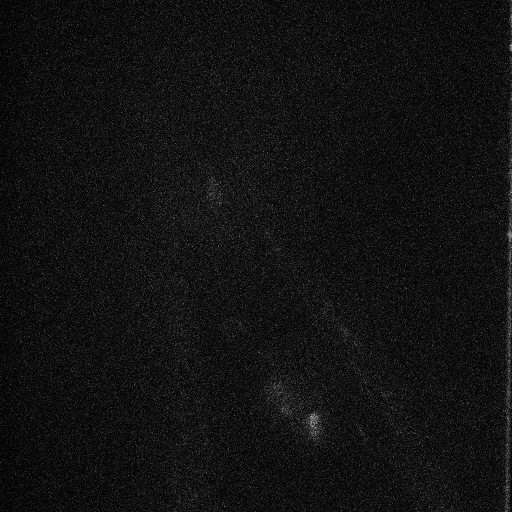

Supplement: Supplementary file 10 — Source data Fig. 1 [file 44318_2024_118_MOESM10_ESM.zip › Figure 1F Micr. image/20230324 Scarlet-che-3; osm-3-g444e-gfp_2 phasmid/img_000000000_L-561_015.tif]

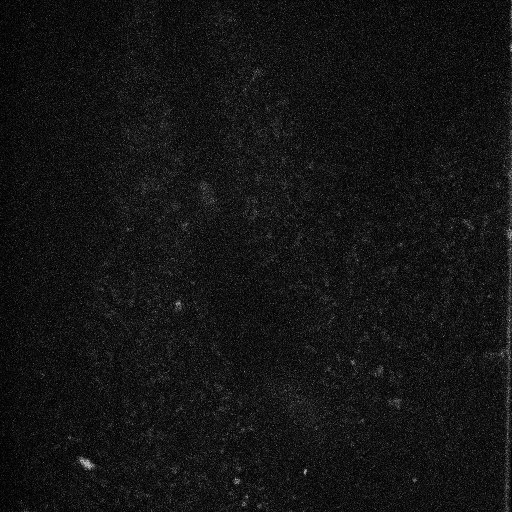

Supplement: Supplementary file 10 — Source data Fig. 1 [file 44318_2024_118_MOESM10_ESM.zip › Figure 1F Micr. image/20230324 Scarlet-che-3; osm-3-g444e-gfp_2 phasmid/img_000000000_L-561_001.tif]

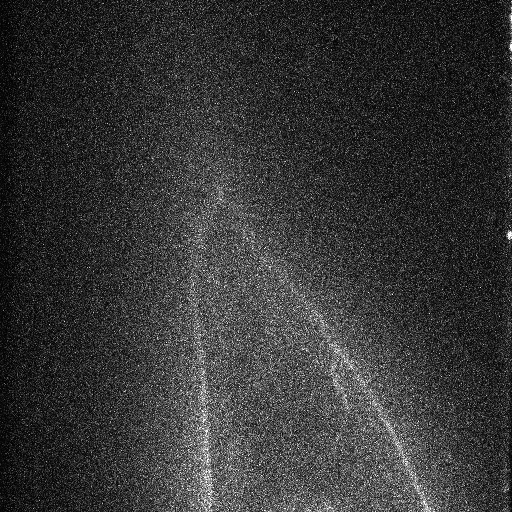

Supplement: Supplementary file 10 — Source data Fig. 1 [file 44318_2024_118_MOESM10_ESM.zip › Figure 1F Micr. image/20230324 Scarlet-che-3; osm-3-g444e-gfp_2 phasmid/img_000000000_L-488_027.tif]

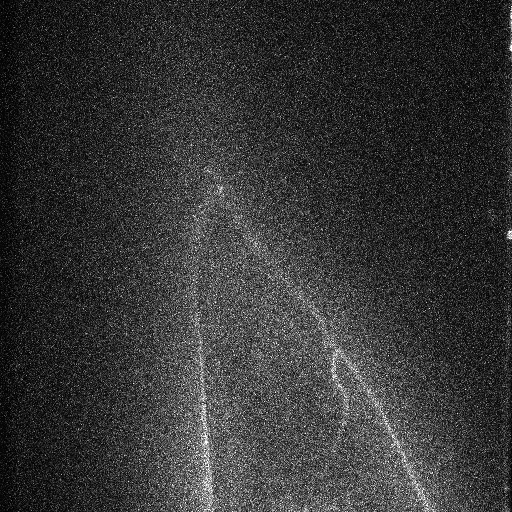

Supplement: Supplementary file 10 — Source data Fig. 1 [file 44318_2024_118_MOESM10_ESM.zip › Figure 1F Micr. image/20230324 Scarlet-che-3; osm-3-g444e-gfp_2 phasmid/img_000000000_L-488_026.tif]

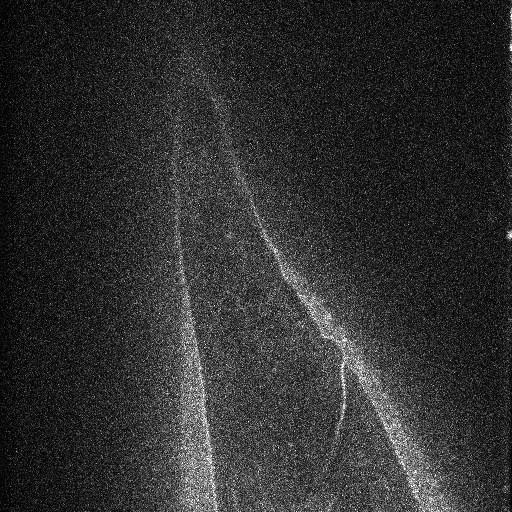

Supplement: Supplementary file 10 — Source data Fig. 1 [file 44318_2024_118_MOESM10_ESM.zip › Figure 1F Micr. image/20230324 Scarlet-che-3; osm-3-g444e-gfp_2 phasmid/img_000000000_L-488_018.tif]

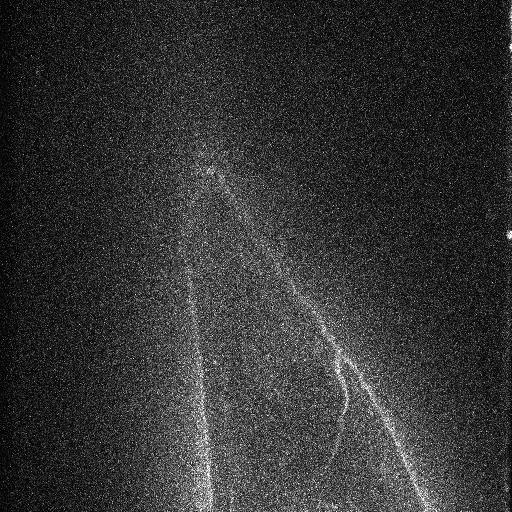

Supplement: Supplementary file 10 — Source data Fig. 1 [file 44318_2024_118_MOESM10_ESM.zip › Figure 1F Micr. image/20230324 Scarlet-che-3; osm-3-g444e-gfp_2 phasmid/img_000000000_L-488_024.tif]

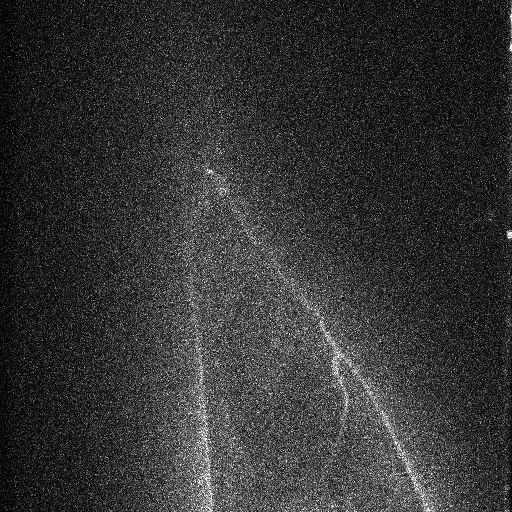

Supplement: Supplementary file 10 — Source data Fig. 1 [file 44318_2024_118_MOESM10_ESM.zip › Figure 1F Micr. image/20230324 Scarlet-che-3; osm-3-g444e-gfp_2 phasmid/img_000000000_L-488_025.tif]

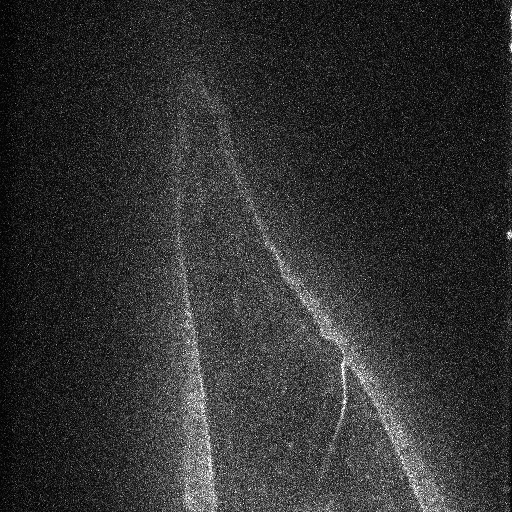

Supplement: Supplementary file 10 — Source data Fig. 1 [file 44318_2024_118_MOESM10_ESM.zip › Figure 1F Micr. image/20230324 Scarlet-che-3; osm-3-g444e-gfp_2 phasmid/img_000000000_L-488_019.tif]

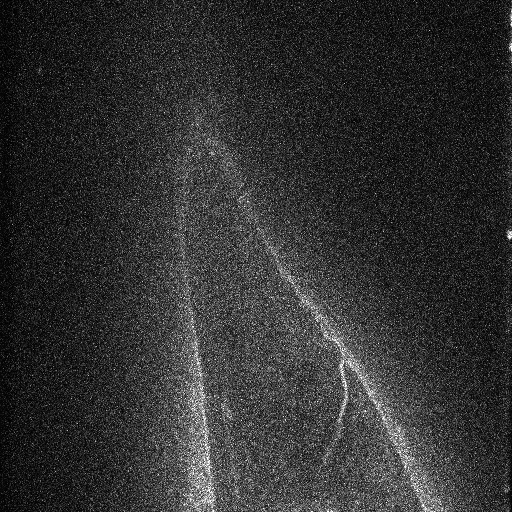

Supplement: Supplementary file 10 — Source data Fig. 1 [file 44318_2024_118_MOESM10_ESM.zip › Figure 1F Micr. image/20230324 Scarlet-che-3; osm-3-g444e-gfp_2 phasmid/img_000000000_L-488_021.tif]

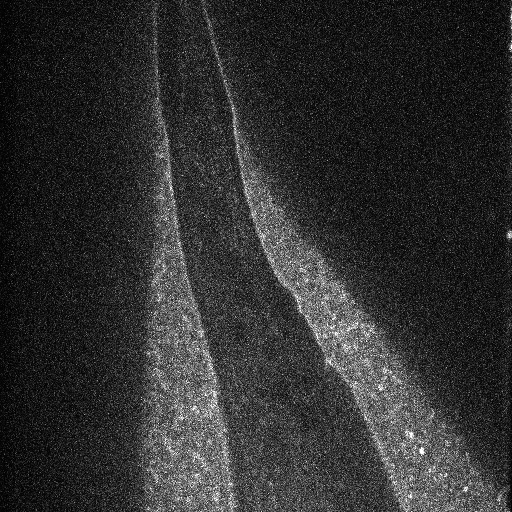

Supplement: Supplementary file 10 — Source data Fig. 1 [file 44318_2024_118_MOESM10_ESM.zip › Figure 1F Micr. image/20230324 Scarlet-che-3; osm-3-g444e-gfp_2 phasmid/img_000000000_L-488_009.tif]

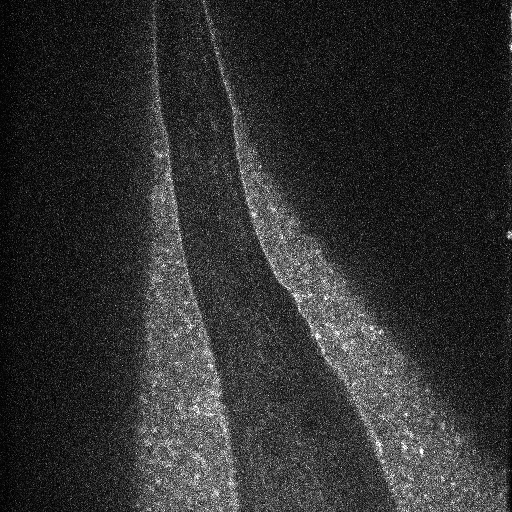

Supplement: Supplementary file 10 — Source data Fig. 1 [file 44318_2024_118_MOESM10_ESM.zip › Figure 1F Micr. image/20230324 Scarlet-che-3; osm-3-g444e-gfp_2 phasmid/img_000000000_L-488_008.tif]

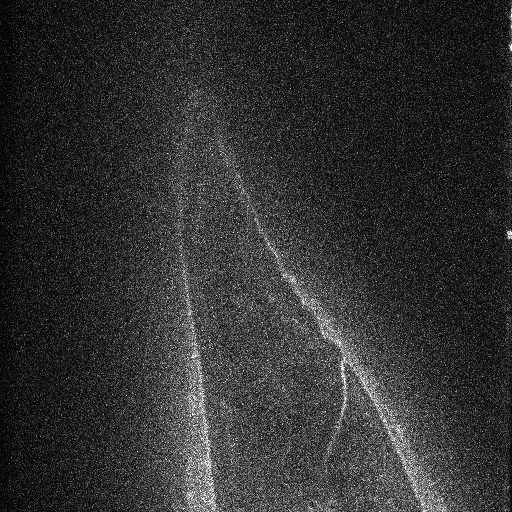

Supplement: Supplementary file 10 — Source data Fig. 1 [file 44318_2024_118_MOESM10_ESM.zip › Figure 1F Micr. image/20230324 Scarlet-che-3; osm-3-g444e-gfp_2 phasmid/img_000000000_L-488_020.tif]

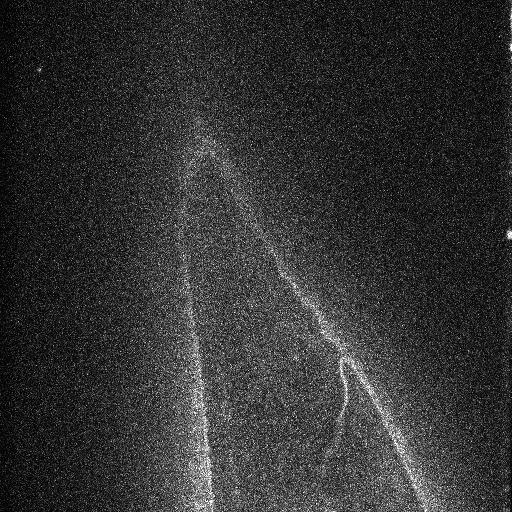

Supplement: Supplementary file 10 — Source data Fig. 1 [file 44318_2024_118_MOESM10_ESM.zip › Figure 1F Micr. image/20230324 Scarlet-che-3; osm-3-g444e-gfp_2 phasmid/img_000000000_L-488_022.tif]

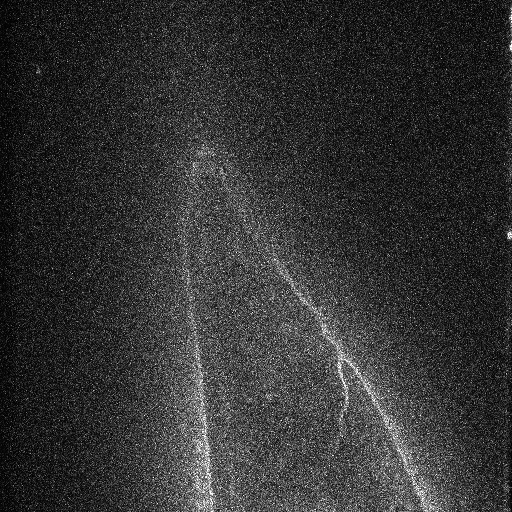

Supplement: Supplementary file 10 — Source data Fig. 1 [file 44318_2024_118_MOESM10_ESM.zip › Figure 1F Micr. image/20230324 Scarlet-che-3; osm-3-g444e-gfp_2 phasmid/img_000000000_L-488_023.tif]

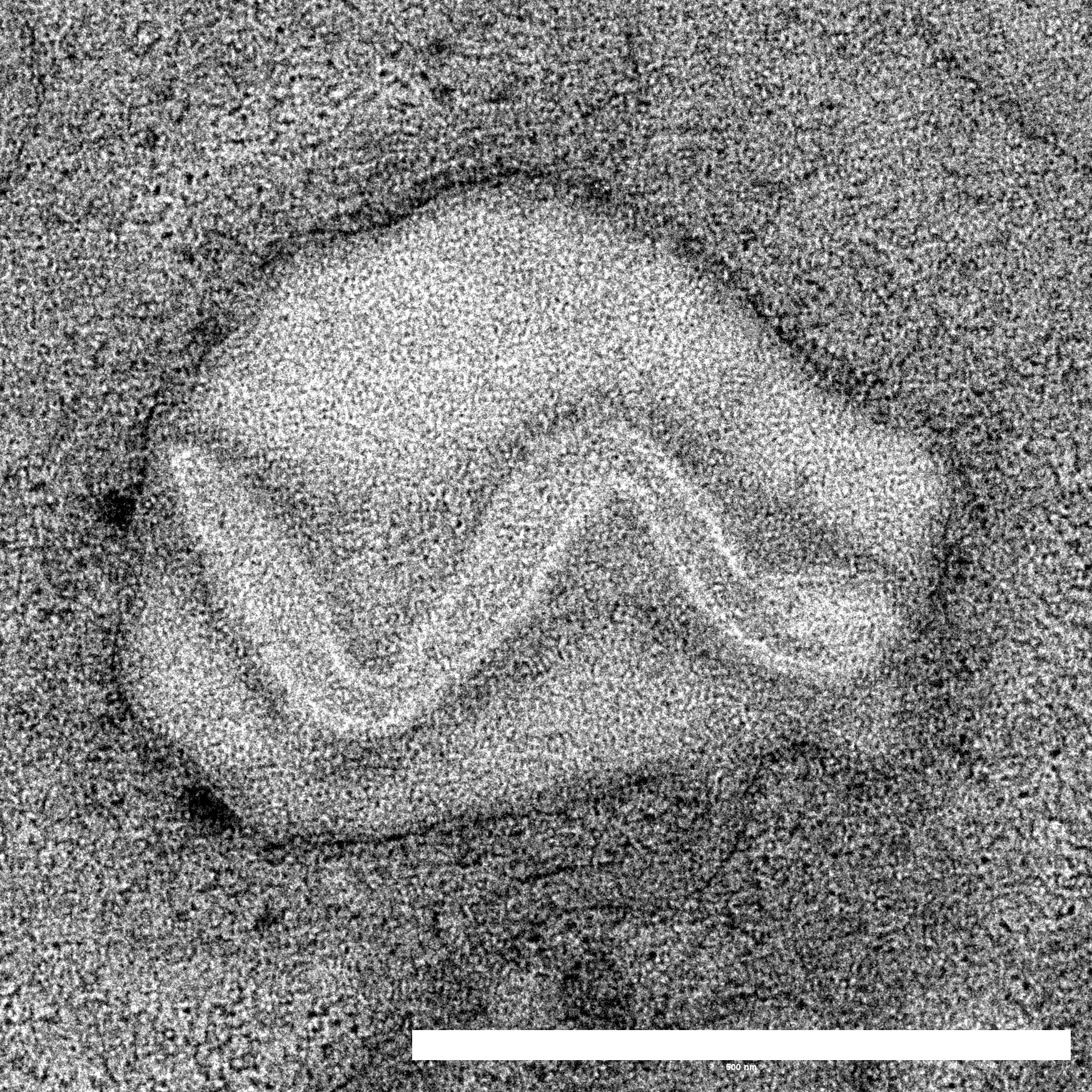

Supplement: Supplementary file 10 — Source data Fig. 1 [file 44318_2024_118_MOESM10_ESM.zip › Figure 1G EM image/G444E-ds.jpg]

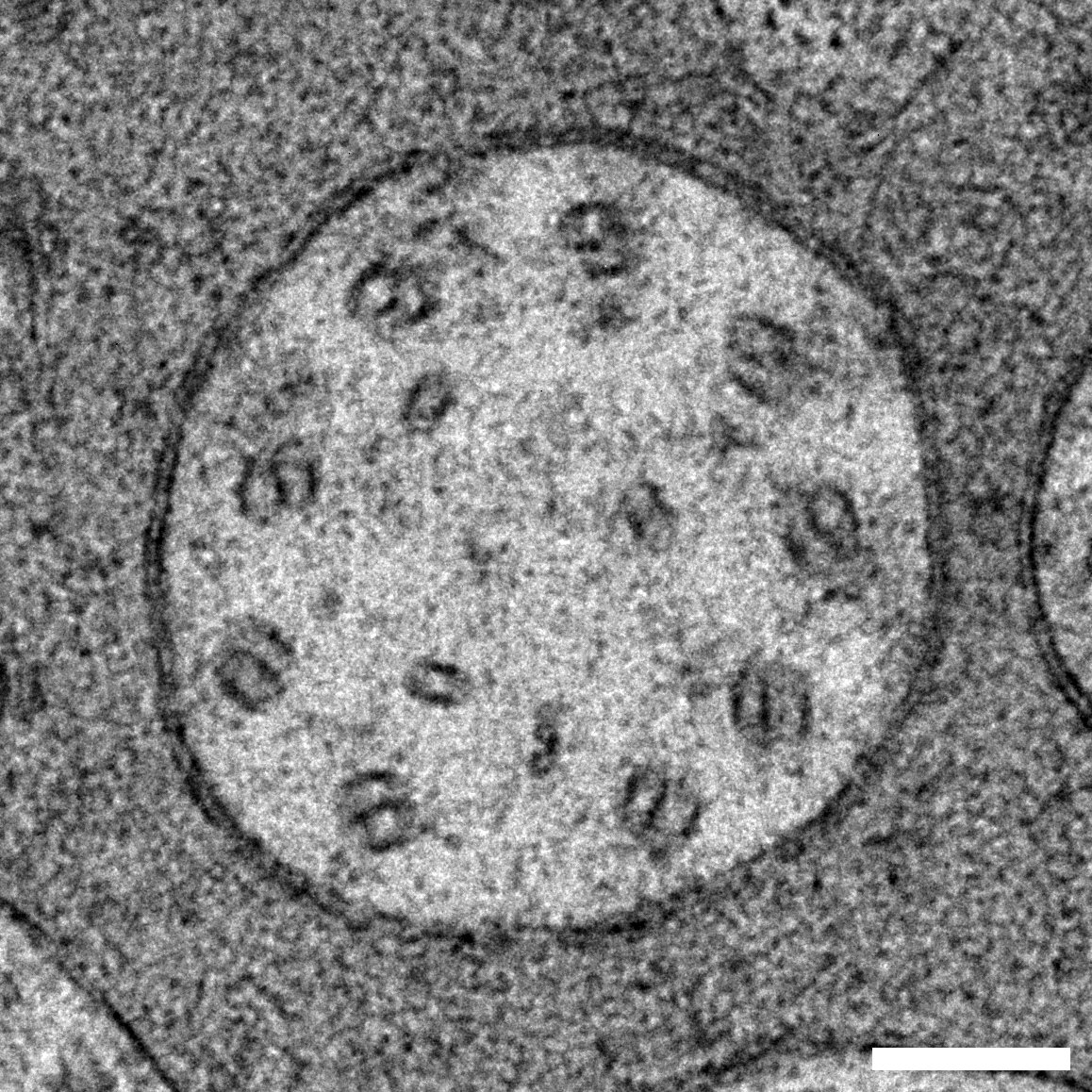

Supplement: Supplementary file 10 — Source data Fig. 1 [file 44318_2024_118_MOESM10_ESM.zip › Figure 1G EM image/N2-cilia.jpg]

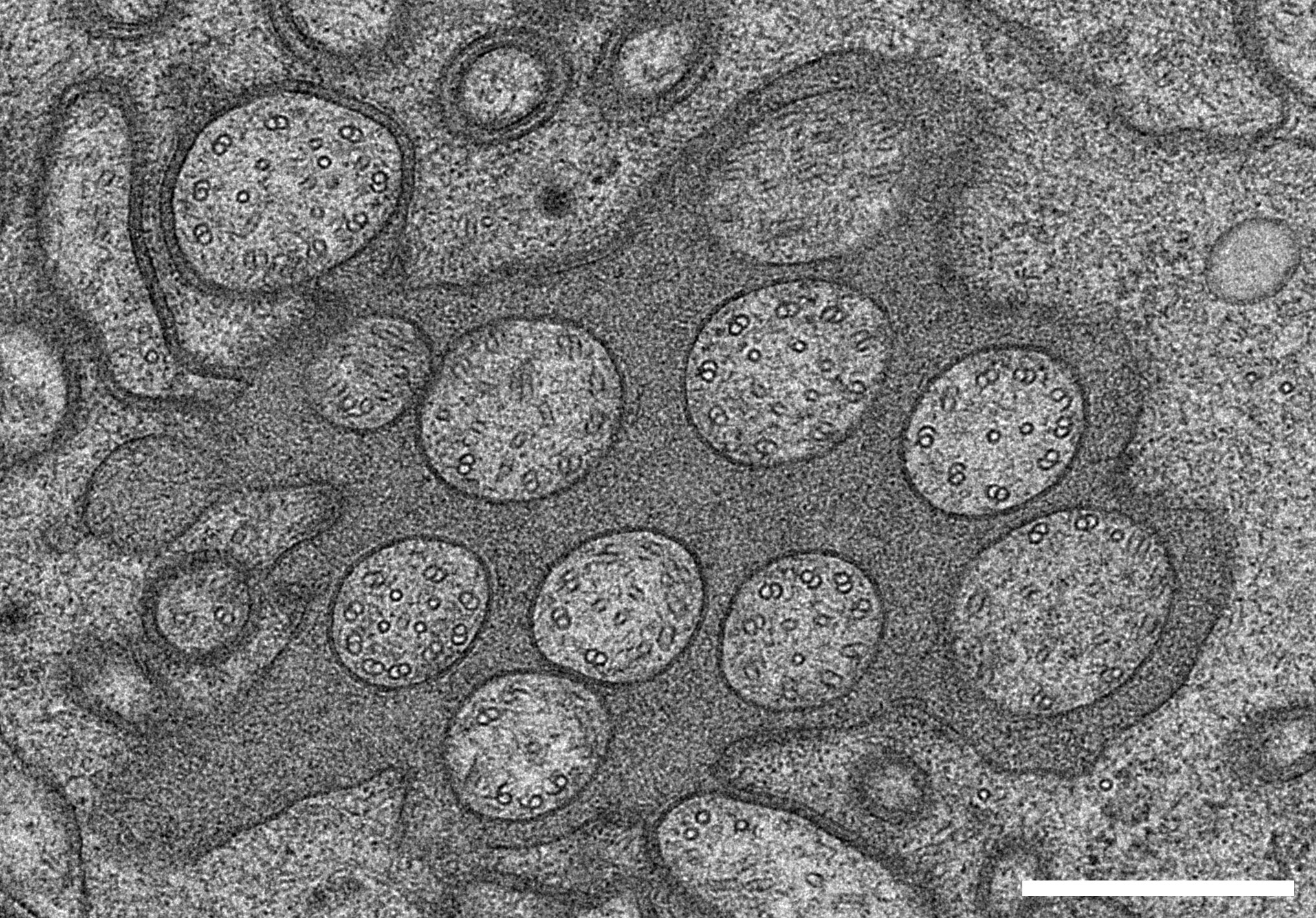

Supplement: Supplementary file 10 — Source data Fig. 1 [file 44318_2024_118_MOESM10_ESM.zip › Figure 1G EM image/N2-ms-s.jpg]

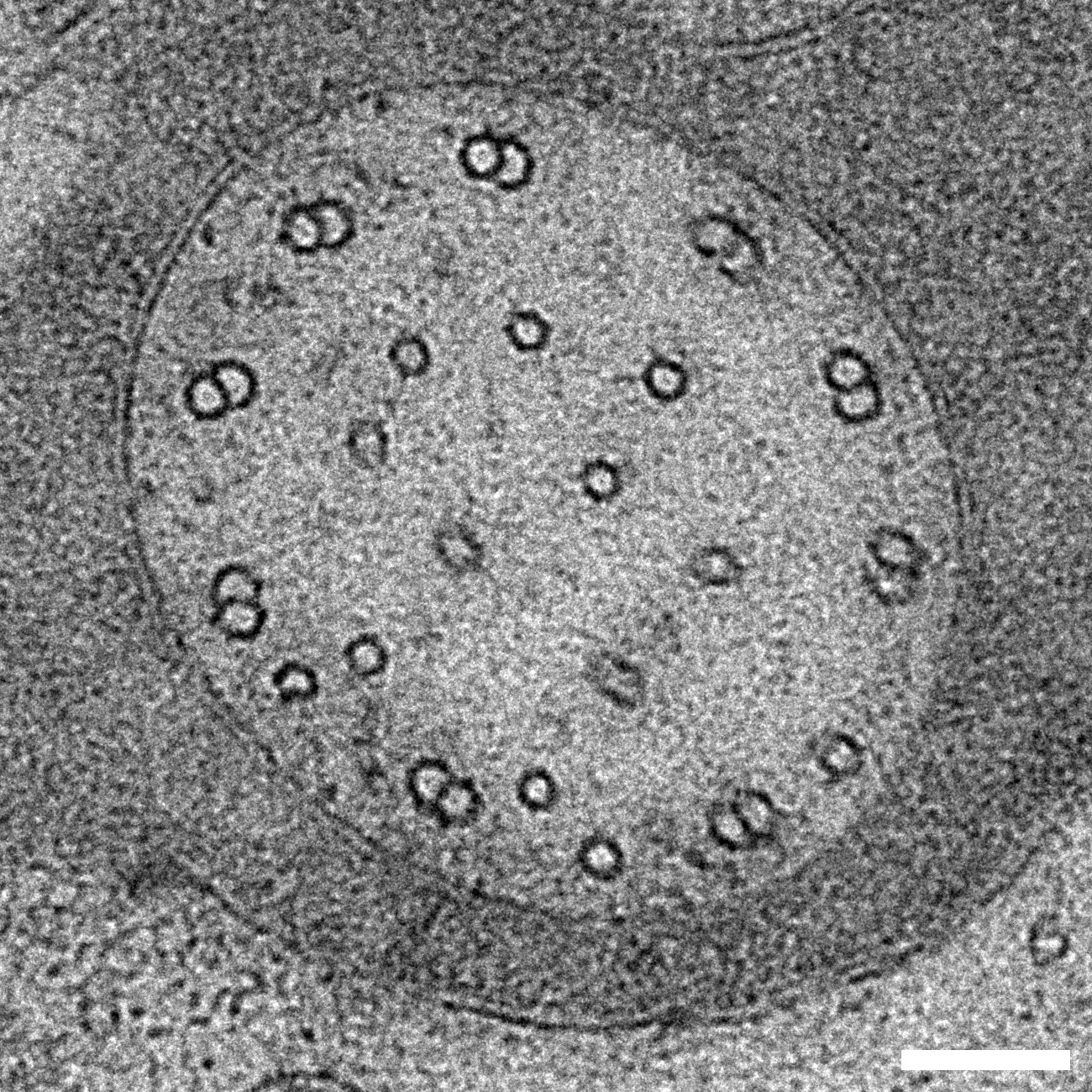

Supplement: Supplementary file 10 — Source data Fig. 1 [file 44318_2024_118_MOESM10_ESM.zip › Figure 1G EM image/osm-3-cilia.jpg]

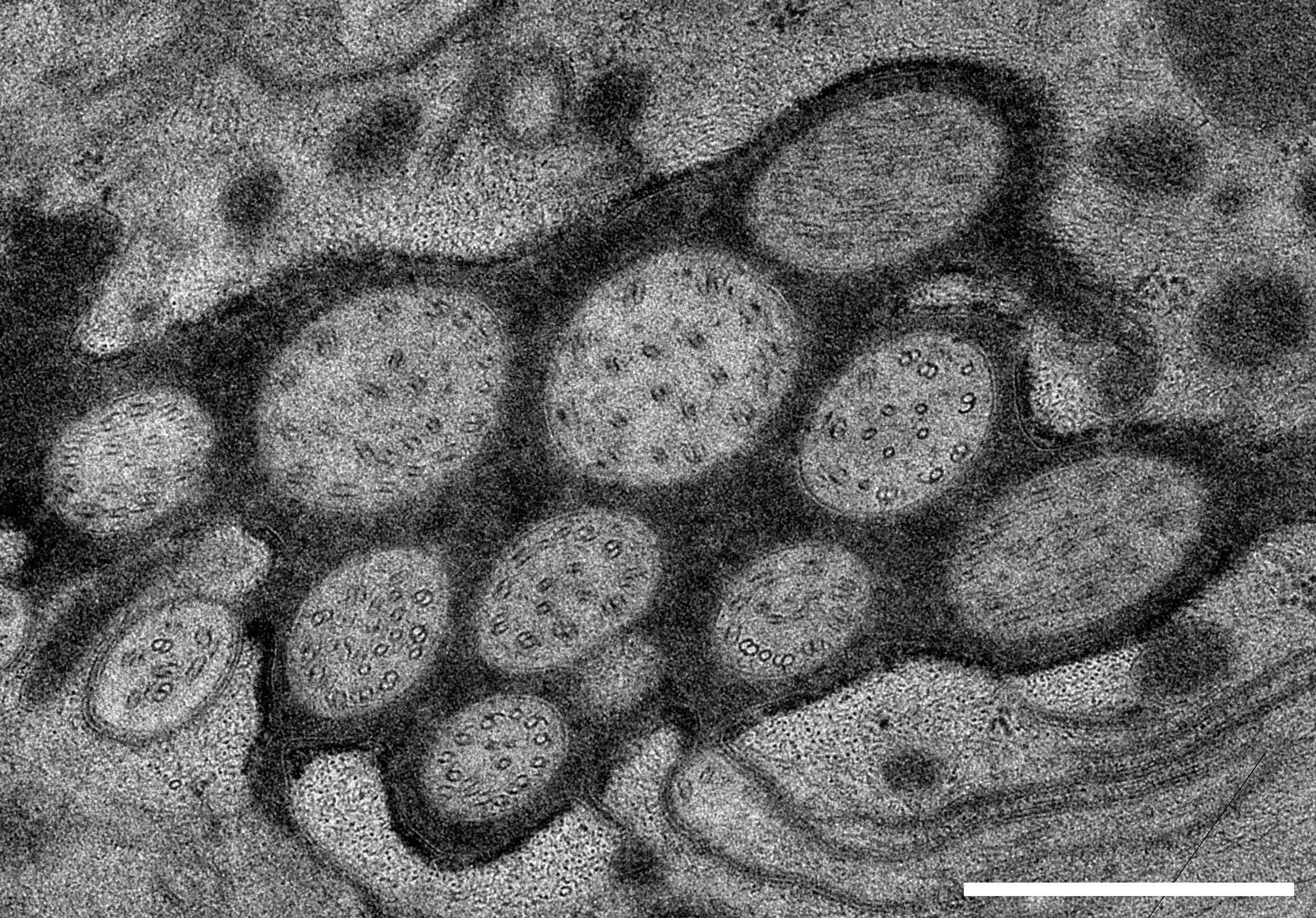

Supplement: Supplementary file 10 — Source data Fig. 1 [file 44318_2024_118_MOESM10_ESM.zip › Figure 1G EM image/sa125-ms-s.jpg]

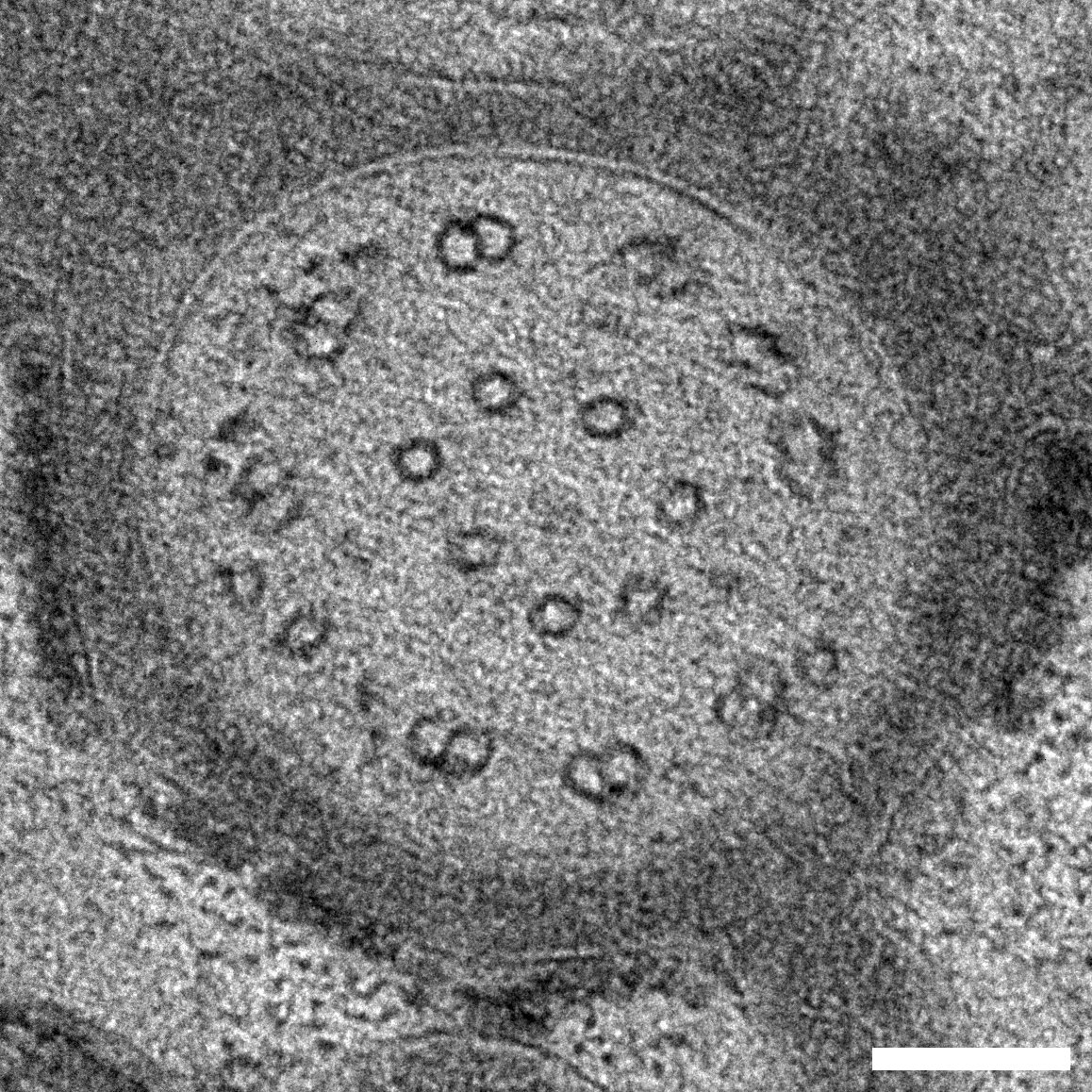

Supplement: Supplementary file 10 — Source data Fig. 1 [file 44318_2024_118_MOESM10_ESM.zip › Figure 1G EM image/sa125-cilia.jpg]

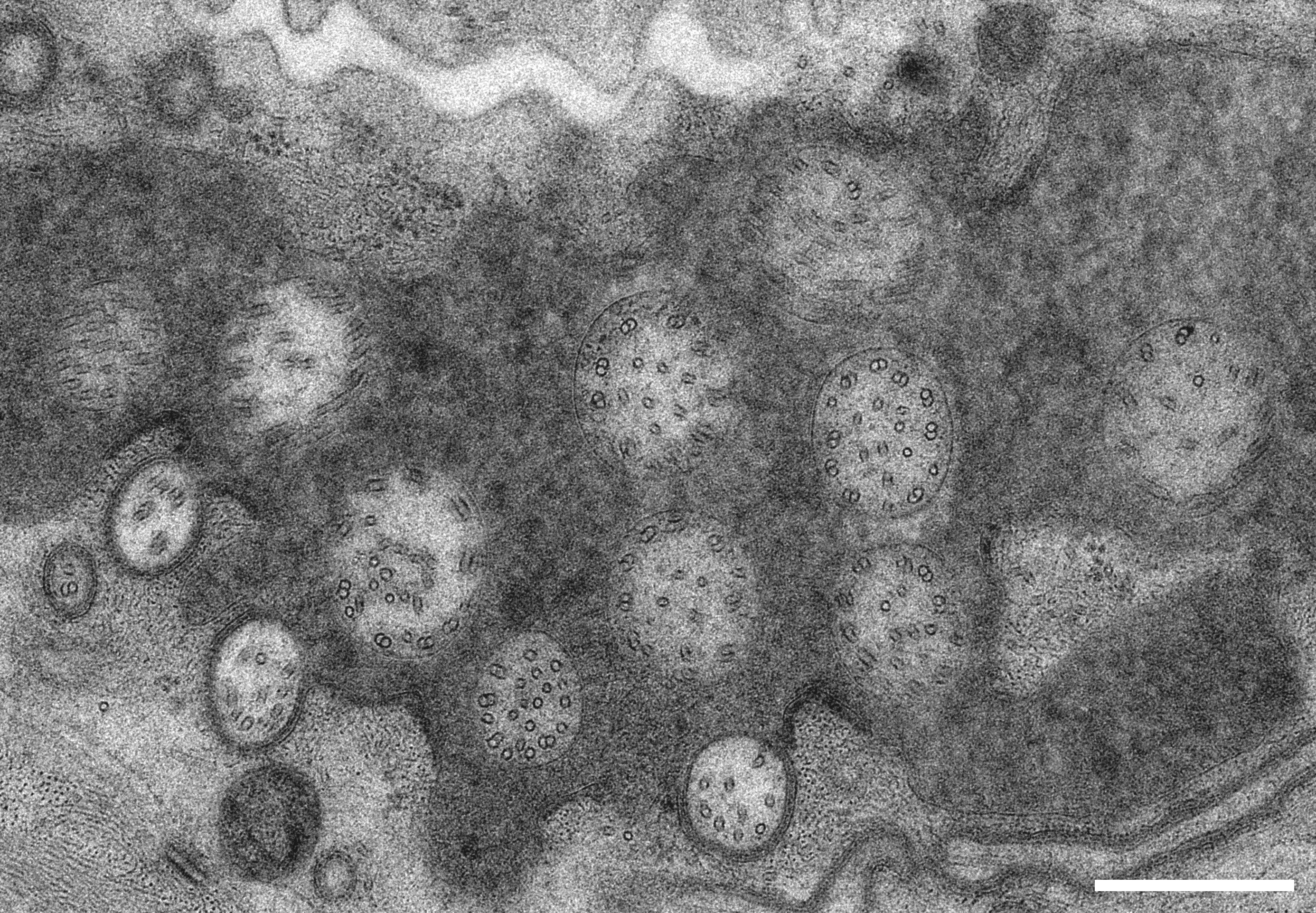

Supplement: Supplementary file 10 — Source data Fig. 1 [file 44318_2024_118_MOESM10_ESM.zip › Figure 1G EM image/osm-3-ms-s.jpg]

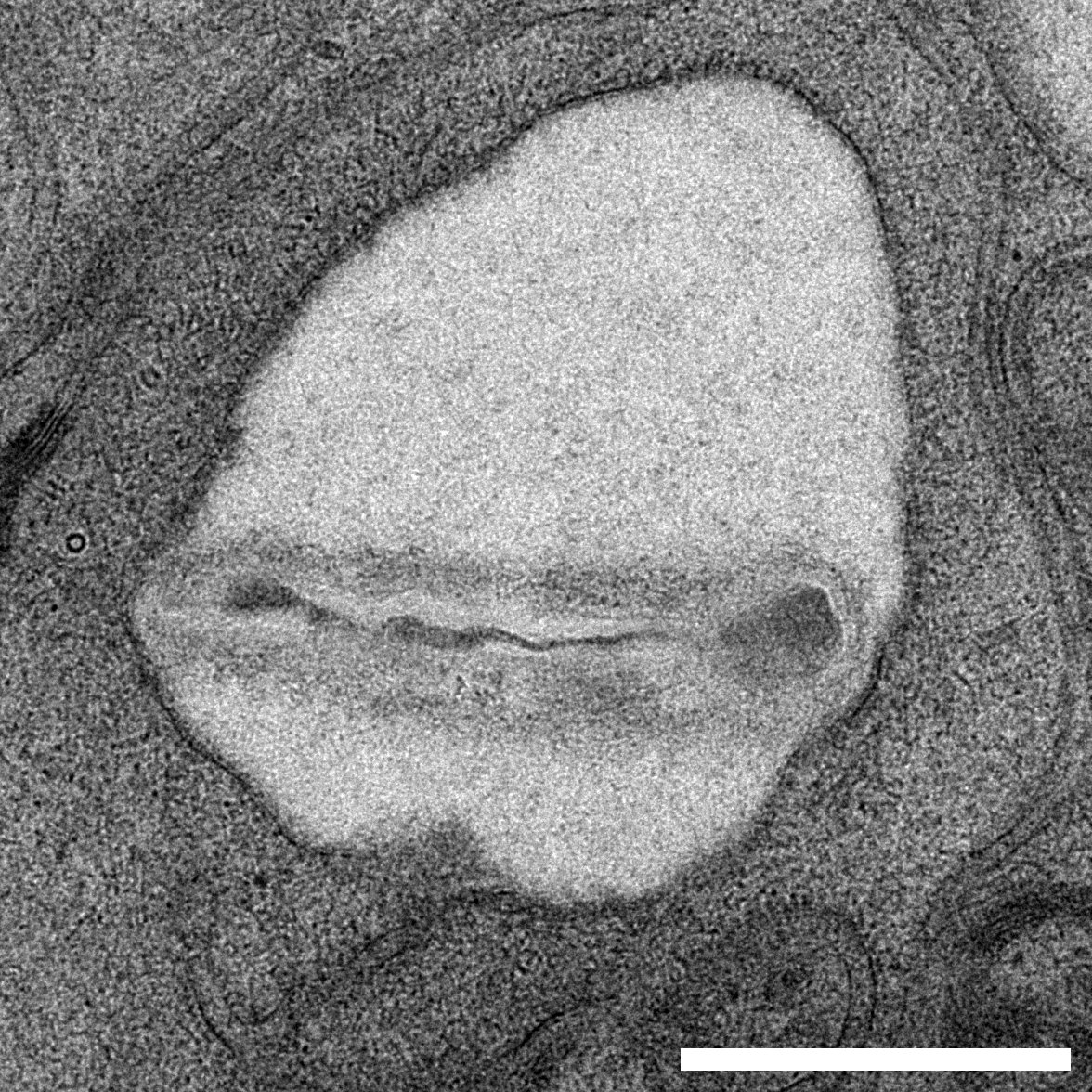

Supplement: Supplementary file 10 — Source data Fig. 1 [file 44318_2024_118_MOESM10_ESM.zip › Figure 1G EM image/osm-3-ds-s.jpg]

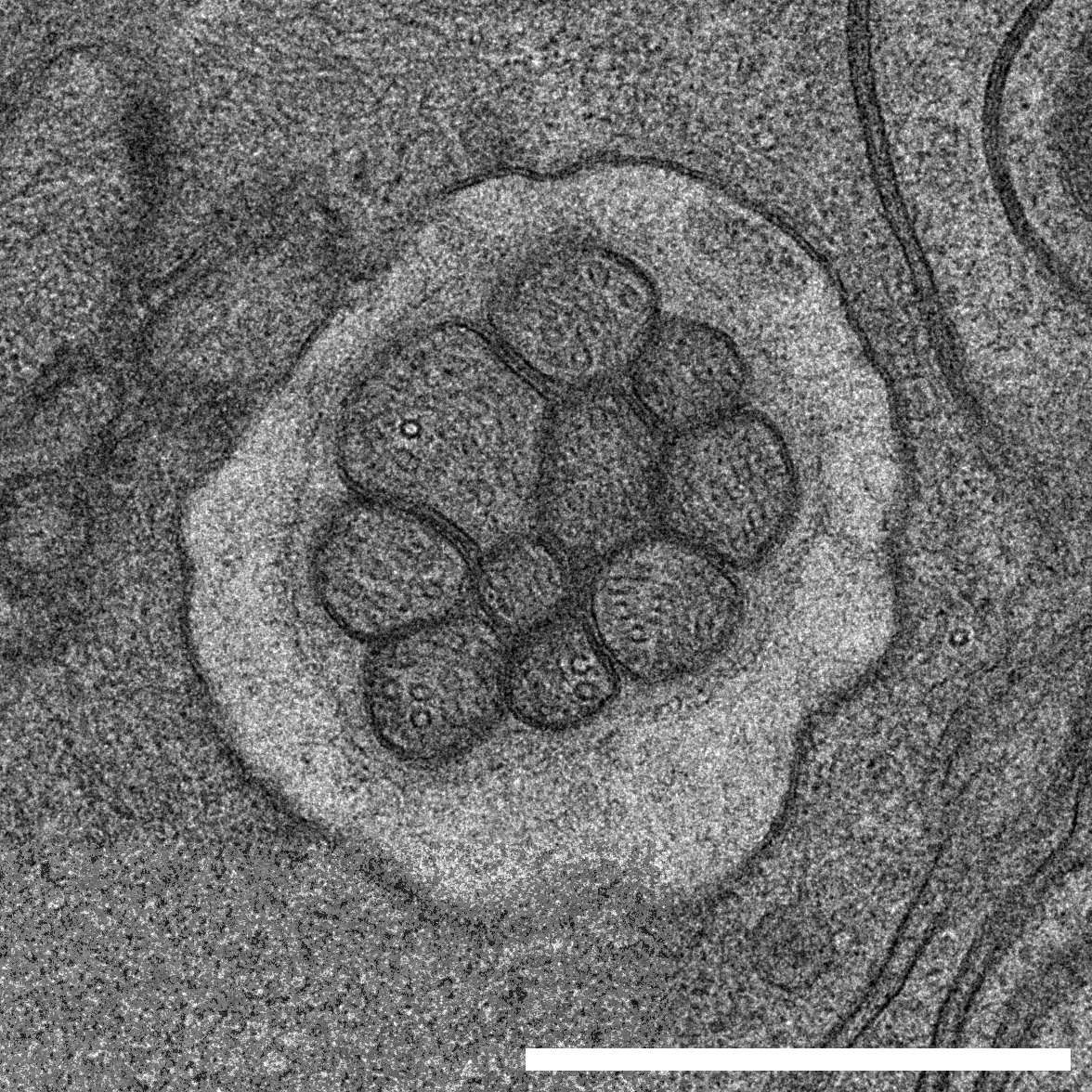

Supplement: Supplementary file 10 — Source data Fig. 1 [file 44318_2024_118_MOESM10_ESM.zip › Figure 1G EM image/N2-ds-s.jpg]

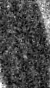

Supplement: Supplementary file 10 — Source data Fig. 1 [file 44318_2024_118_MOESM10_ESM.zip › Figure 1H Micr. image/AVG_20230602 OSM-3-G444E-GFP; Pdyf-1-osm-3-Scarlet_13-1 phasmid 50x88.tif]

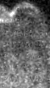

Supplement: Supplementary file 10 — Source data Fig. 1 [file 44318_2024_118_MOESM10_ESM.zip › Figure 1H Micr. image/AVG_20230602 OSM-3-G444E-GFP; Pdyf-1-osm-3-Scarlet_2-1 amphid 50x88.tif]

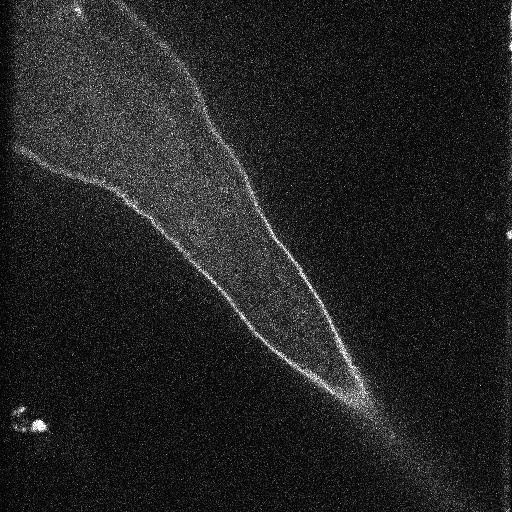

Supplement: Supplementary file 10 — Source data Fig. 1 [file 44318_2024_118_MOESM10_ESM.zip › Figure 1H Micr. image/20230602 OSM-3-G444E-GFP; Pdyf-1-osm-3-Scarlet_13/img_000000000_L-488_006.tif]

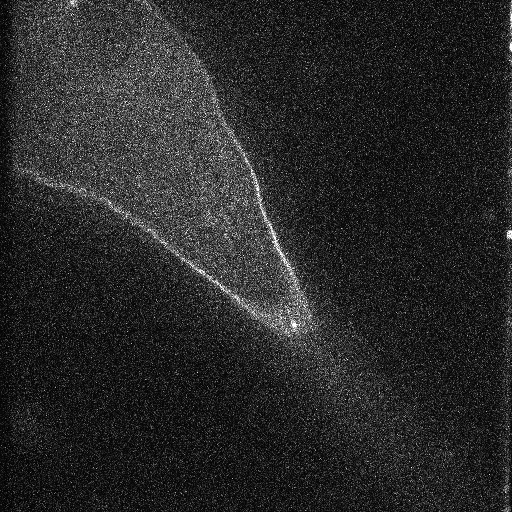

Supplement: Supplementary file 10 — Source data Fig. 1 [file 44318_2024_118_MOESM10_ESM.zip › Figure 1H Micr. image/20230602 OSM-3-G444E-GFP; Pdyf-1-osm-3-Scarlet_13/img_000000000_L-488_012.tif]

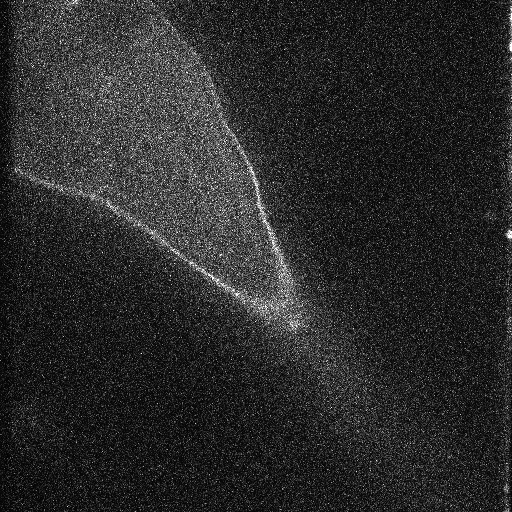

Supplement: Supplementary file 10 — Source data Fig. 1 [file 44318_2024_118_MOESM10_ESM.zip › Figure 1H Micr. image/20230602 OSM-3-G444E-GFP; Pdyf-1-osm-3-Scarlet_13/img_000000000_L-488_013.tif]

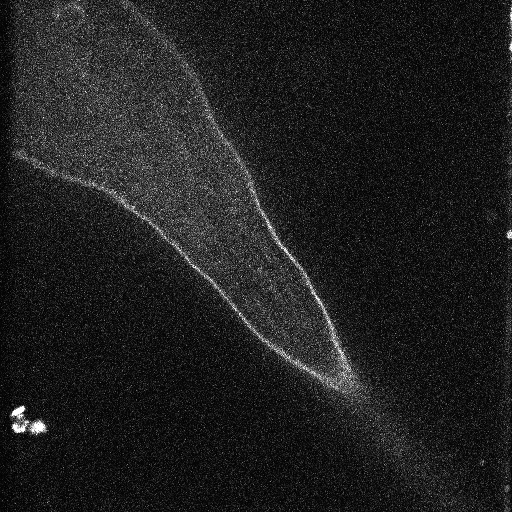

Supplement: Supplementary file 10 — Source data Fig. 1 [file 44318_2024_118_MOESM10_ESM.zip › Figure 1H Micr. image/20230602 OSM-3-G444E-GFP; Pdyf-1-osm-3-Scarlet_13/img_000000000_L-488_007.tif]

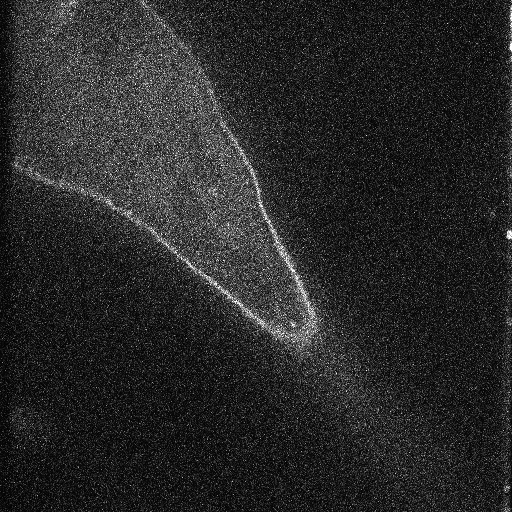

Supplement: Supplementary file 10 — Source data Fig. 1 [file 44318_2024_118_MOESM10_ESM.zip › Figure 1H Micr. image/20230602 OSM-3-G444E-GFP; Pdyf-1-osm-3-Scarlet_13/img_000000000_L-488_011.tif]

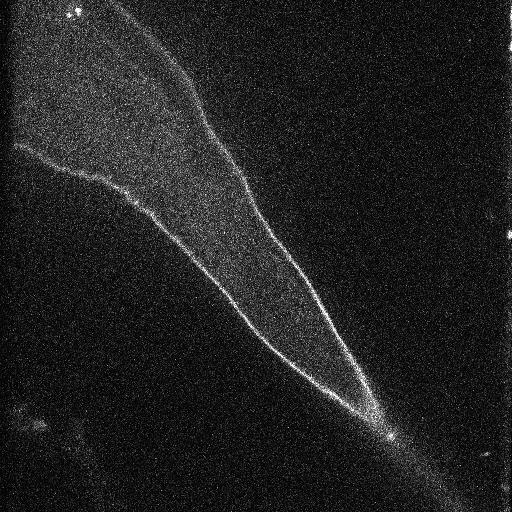

Supplement: Supplementary file 10 — Source data Fig. 1 [file 44318_2024_118_MOESM10_ESM.zip › Figure 1H Micr. image/20230602 OSM-3-G444E-GFP; Pdyf-1-osm-3-Scarlet_13/img_000000000_L-488_005.tif]

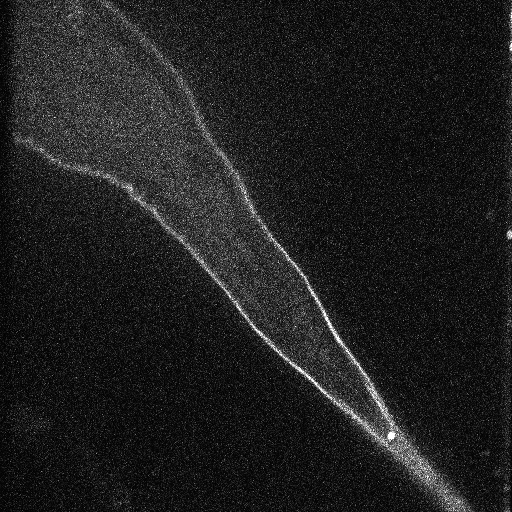

Supplement: Supplementary file 10 — Source data Fig. 1 [file 44318_2024_118_MOESM10_ESM.zip › Figure 1H Micr. image/20230602 OSM-3-G444E-GFP; Pdyf-1-osm-3-Scarlet_13/img_000000000_L-488_004.tif]

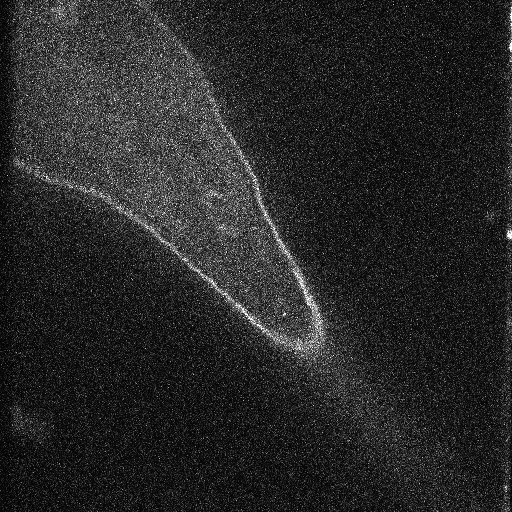

Supplement: Supplementary file 10 — Source data Fig. 1 [file 44318_2024_118_MOESM10_ESM.zip › Figure 1H Micr. image/20230602 OSM-3-G444E-GFP; Pdyf-1-osm-3-Scarlet_13/img_000000000_L-488_010.tif]

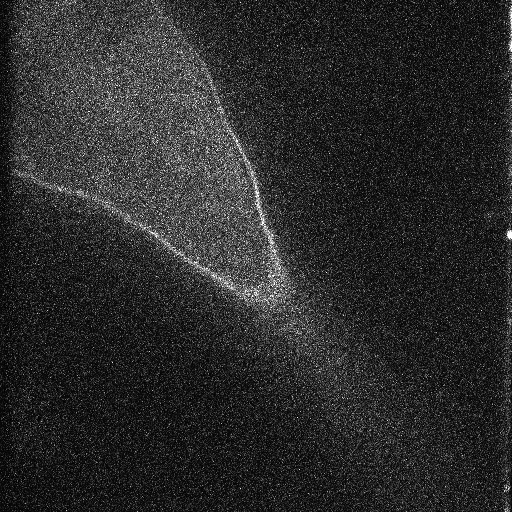

Supplement: Supplementary file 10 — Source data Fig. 1 [file 44318_2024_118_MOESM10_ESM.zip › Figure 1H Micr. image/20230602 OSM-3-G444E-GFP; Pdyf-1-osm-3-Scarlet_13/img_000000000_L-488_014.tif]

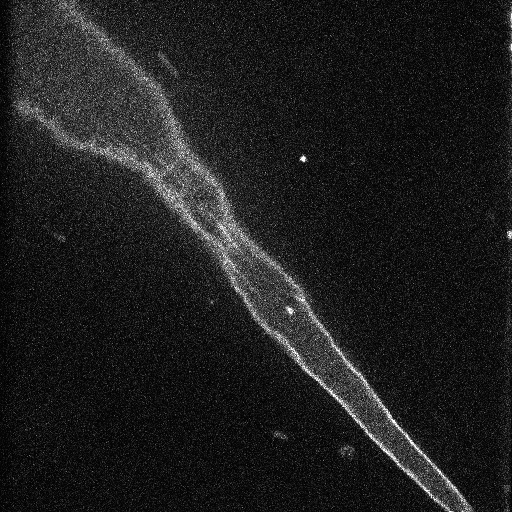

Supplement: Supplementary file 10 — Source data Fig. 1 [file 44318_2024_118_MOESM10_ESM.zip › Figure 1H Micr. image/20230602 OSM-3-G444E-GFP; Pdyf-1-osm-3-Scarlet_13/img_000000000_L-488_000.tif]

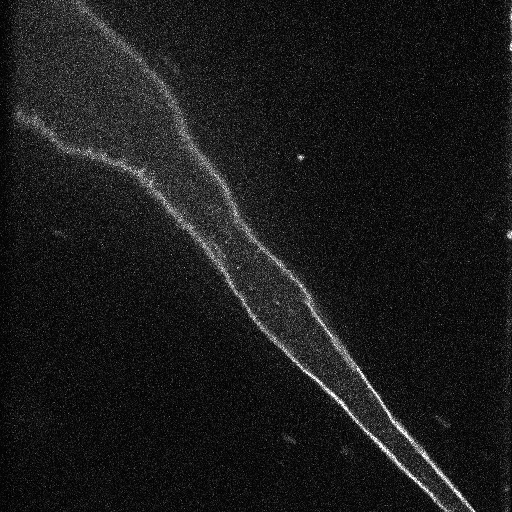

Supplement: Supplementary file 10 — Source data Fig. 1 [file 44318_2024_118_MOESM10_ESM.zip › Figure 1H Micr. image/20230602 OSM-3-G444E-GFP; Pdyf-1-osm-3-Scarlet_13/img_000000000_L-488_001.tif]

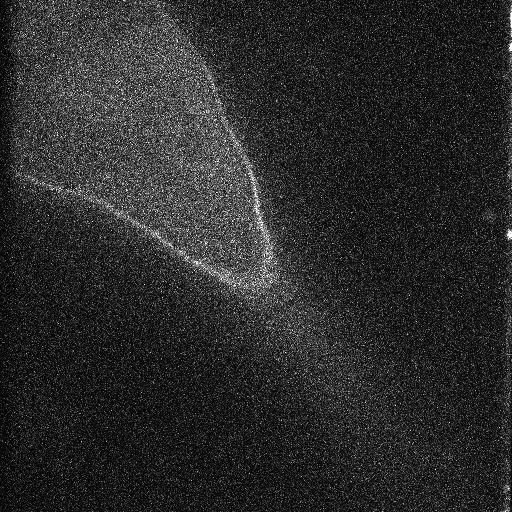

Supplement: Supplementary file 10 — Source data Fig. 1 [file 44318_2024_118_MOESM10_ESM.zip › Figure 1H Micr. image/20230602 OSM-3-G444E-GFP; Pdyf-1-osm-3-Scarlet_13/img_000000000_L-488_015.tif]

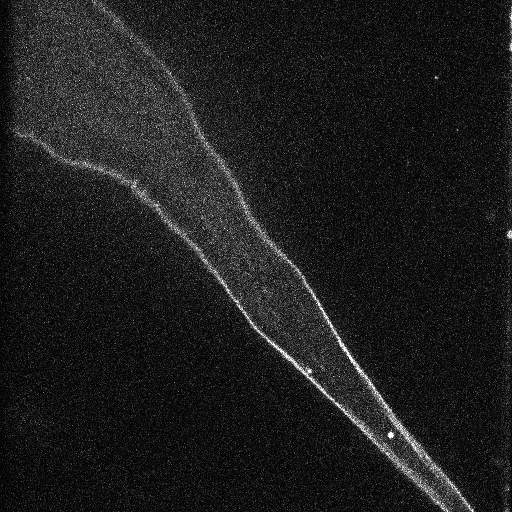

Supplement: Supplementary file 10 — Source data Fig. 1 [file 44318_2024_118_MOESM10_ESM.zip › Figure 1H Micr. image/20230602 OSM-3-G444E-GFP; Pdyf-1-osm-3-Scarlet_13/img_000000000_L-488_003.tif]

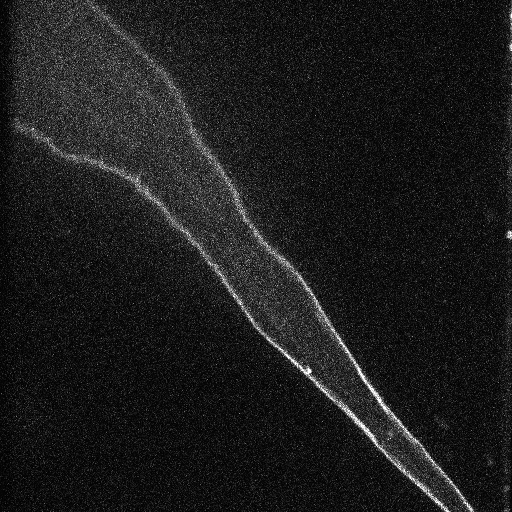

Supplement: Supplementary file 10 — Source data Fig. 1 [file 44318_2024_118_MOESM10_ESM.zip › Figure 1H Micr. image/20230602 OSM-3-G444E-GFP; Pdyf-1-osm-3-Scarlet_13/img_000000000_L-488_002.tif]

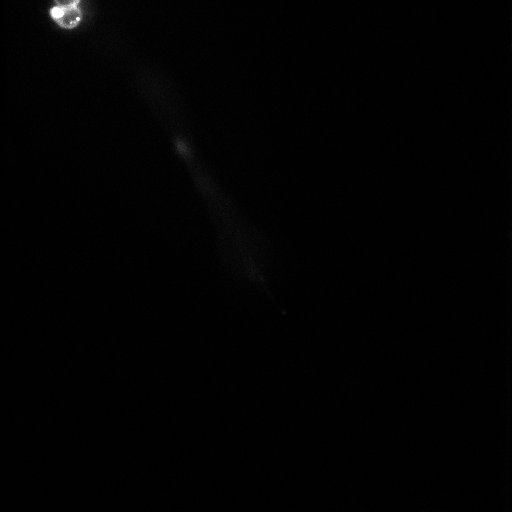

Supplement: Supplementary file 10 — Source data Fig. 1 [file 44318_2024_118_MOESM10_ESM.zip › Figure 1H Micr. image/20230602 OSM-3-G444E-GFP; Pdyf-1-osm-3-Scarlet_13/img_000000000_L-561_009.tif]

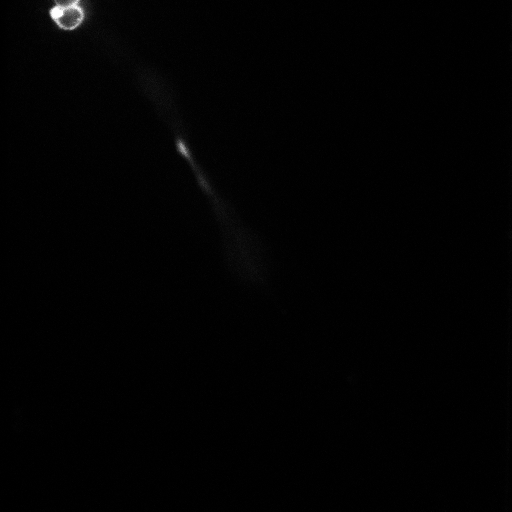

Supplement: Supplementary file 10 — Source data Fig. 1 [file 44318_2024_118_MOESM10_ESM.zip › Figure 1H Micr. image/20230602 OSM-3-G444E-GFP; Pdyf-1-osm-3-Scarlet_13/img_000000000_L-561_008.tif]

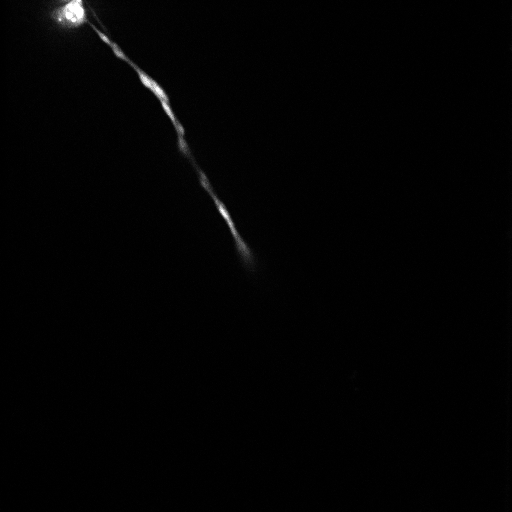

Supplement: Supplementary file 10 — Source data Fig. 1 [file 44318_2024_118_MOESM10_ESM.zip › Figure 1H Micr. image/20230602 OSM-3-G444E-GFP; Pdyf-1-osm-3-Scarlet_13/img_000000000_L-561_005.tif]

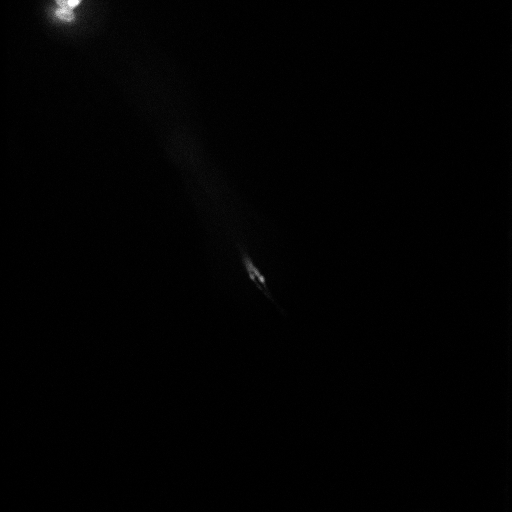

Supplement: Supplementary file 10 — Source data Fig. 1 [file 44318_2024_118_MOESM10_ESM.zip › Figure 1H Micr. image/20230602 OSM-3-G444E-GFP; Pdyf-1-osm-3-Scarlet_13/img_000000000_L-561_011.tif]

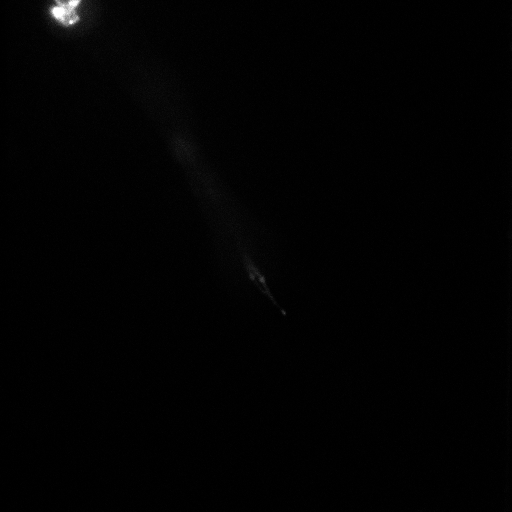

Supplement: Supplementary file 10 — Source data Fig. 1 [file 44318_2024_118_MOESM10_ESM.zip › Figure 1H Micr. image/20230602 OSM-3-G444E-GFP; Pdyf-1-osm-3-Scarlet_13/img_000000000_L-561_010.tif]

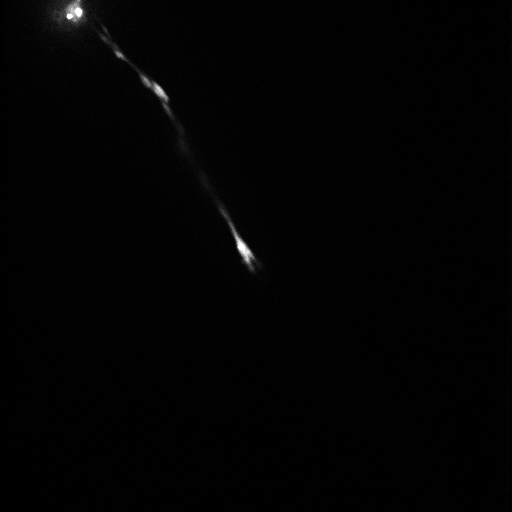

Supplement: Supplementary file 10 — Source data Fig. 1 [file 44318_2024_118_MOESM10_ESM.zip › Figure 1H Micr. image/20230602 OSM-3-G444E-GFP; Pdyf-1-osm-3-Scarlet_13/img_000000000_L-561_004.tif]

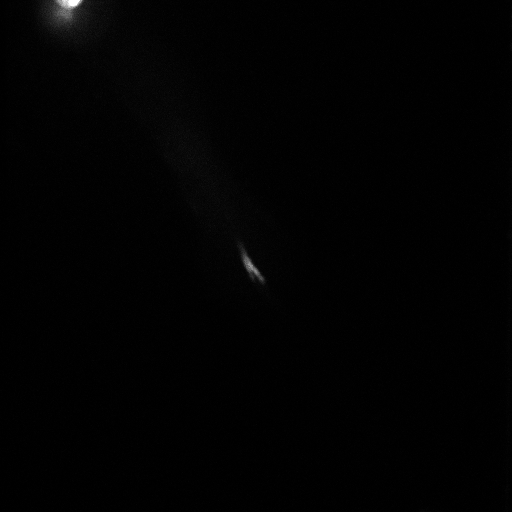

Supplement: Supplementary file 10 — Source data Fig. 1 [file 44318_2024_118_MOESM10_ESM.zip › Figure 1H Micr. image/20230602 OSM-3-G444E-GFP; Pdyf-1-osm-3-Scarlet_13/img_000000000_L-561_012.tif]

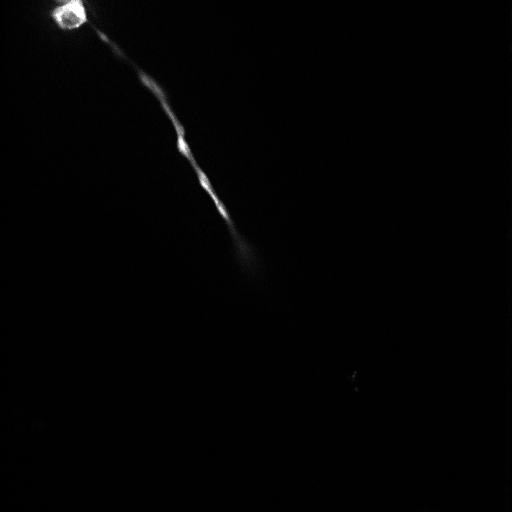

Supplement: Supplementary file 10 — Source data Fig. 1 [file 44318_2024_118_MOESM10_ESM.zip › Figure 1H Micr. image/20230602 OSM-3-G444E-GFP; Pdyf-1-osm-3-Scarlet_13/img_000000000_L-561_006.tif]

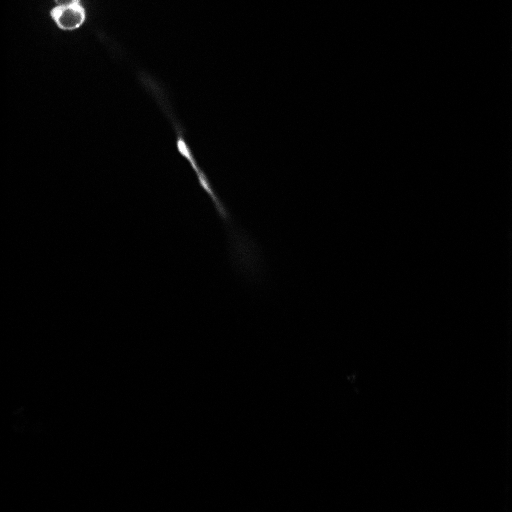

Supplement: Supplementary file 10 — Source data Fig. 1 [file 44318_2024_118_MOESM10_ESM.zip › Figure 1H Micr. image/20230602 OSM-3-G444E-GFP; Pdyf-1-osm-3-Scarlet_13/img_000000000_L-561_007.tif]

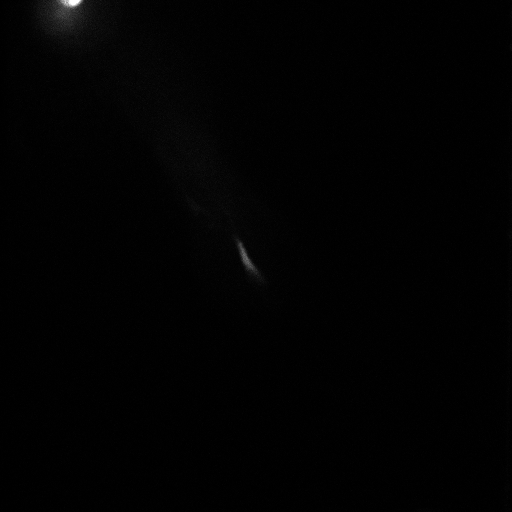

Supplement: Supplementary file 10 — Source data Fig. 1 [file 44318_2024_118_MOESM10_ESM.zip › Figure 1H Micr. image/20230602 OSM-3-G444E-GFP; Pdyf-1-osm-3-Scarlet_13/img_000000000_L-561_013.tif]

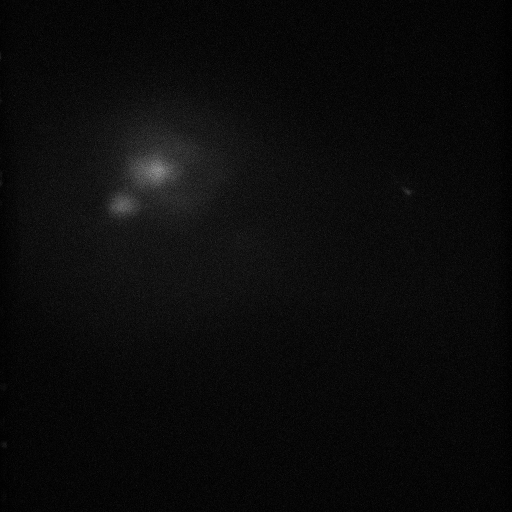

Supplement: Supplementary file 11 — Source data Fig. 2 [file 44318_2024_118_MOESM11_ESM.zip › Figure2/Figure 2A Micr. image/20201128 osm-3 G444E-gfp; HIS-54-BFP_3/Pos0/img_000000000_Confocal-405_000.tif]

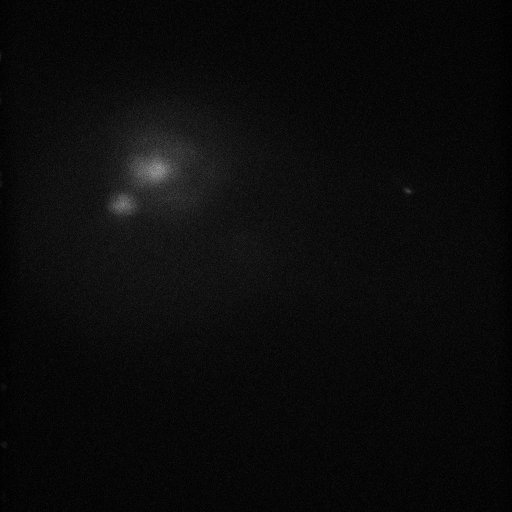

Supplement: Supplementary file 11 — Source data Fig. 2 [file 44318_2024_118_MOESM11_ESM.zip › Figure2/Figure 2A Micr. image/20201128 osm-3 G444E-gfp; HIS-54-BFP_3/Pos0/img_000000000_Confocal-405_001.tif]

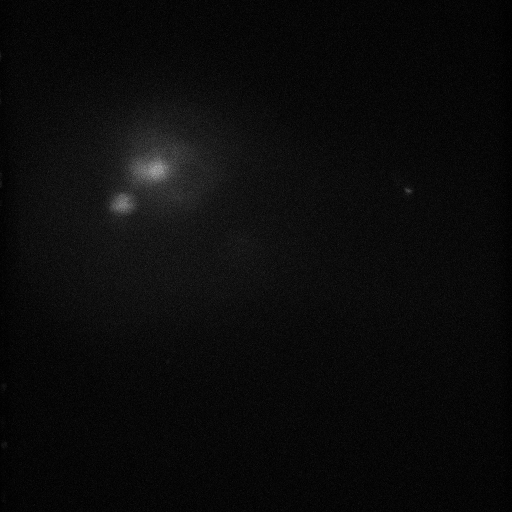

Supplement: Supplementary file 11 — Source data Fig. 2 [file 44318_2024_118_MOESM11_ESM.zip › Figure2/Figure 2A Micr. image/20201128 osm-3 G444E-gfp; HIS-54-BFP_3/Pos0/img_000000000_Confocal-405_002.tif]

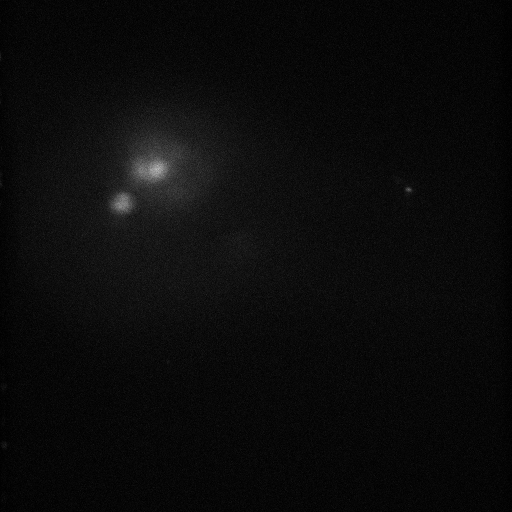

Supplement: Supplementary file 11 — Source data Fig. 2 [file 44318_2024_118_MOESM11_ESM.zip › Figure2/Figure 2A Micr. image/20201128 osm-3 G444E-gfp; HIS-54-BFP_3/Pos0/img_000000000_Confocal-405_003.tif]

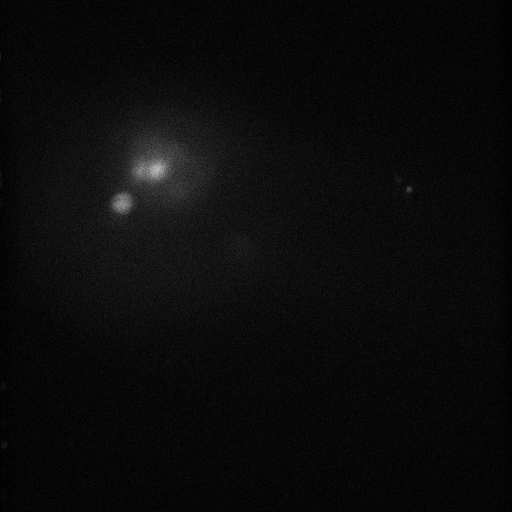

Supplement: Supplementary file 11 — Source data Fig. 2 [file 44318_2024_118_MOESM11_ESM.zip › Figure2/Figure 2A Micr. image/20201128 osm-3 G444E-gfp; HIS-54-BFP_3/Pos0/img_000000000_Confocal-405_004.tif]

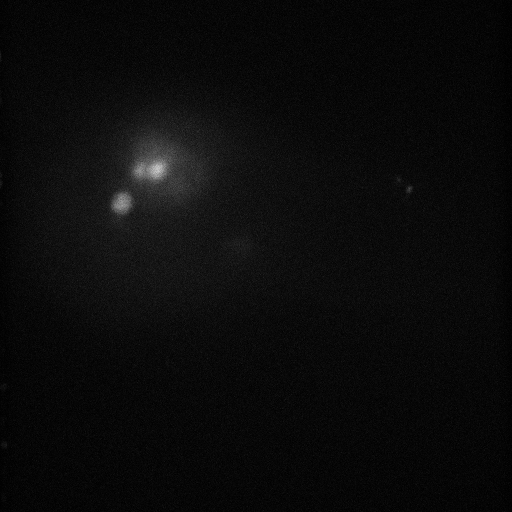

Supplement: Supplementary file 11 — Source data Fig. 2 [file 44318_2024_118_MOESM11_ESM.zip › Figure2/Figure 2A Micr. image/20201128 osm-3 G444E-gfp; HIS-54-BFP_3/Pos0/img_000000000_Confocal-405_005.tif]

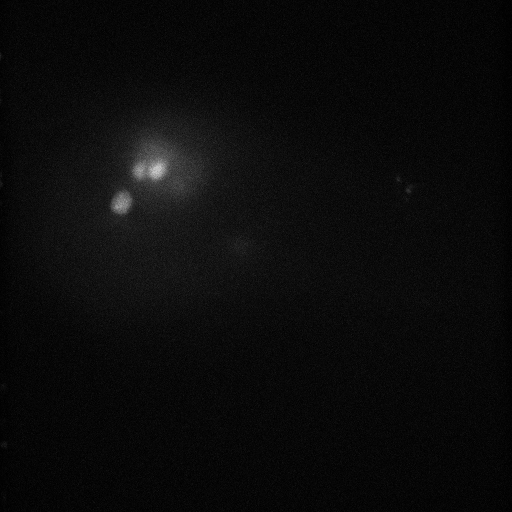

Supplement: Supplementary file 11 — Source data Fig. 2 [file 44318_2024_118_MOESM11_ESM.zip › Figure2/Figure 2A Micr. image/20201128 osm-3 G444E-gfp; HIS-54-BFP_3/Pos0/img_000000000_Confocal-405_006.tif]

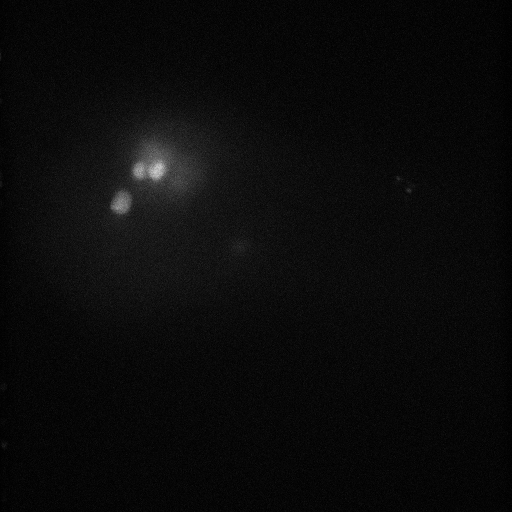

Supplement: Supplementary file 11 — Source data Fig. 2 [file 44318_2024_118_MOESM11_ESM.zip › Figure2/Figure 2A Micr. image/20201128 osm-3 G444E-gfp; HIS-54-BFP_3/Pos0/img_000000000_Confocal-405_007.tif]

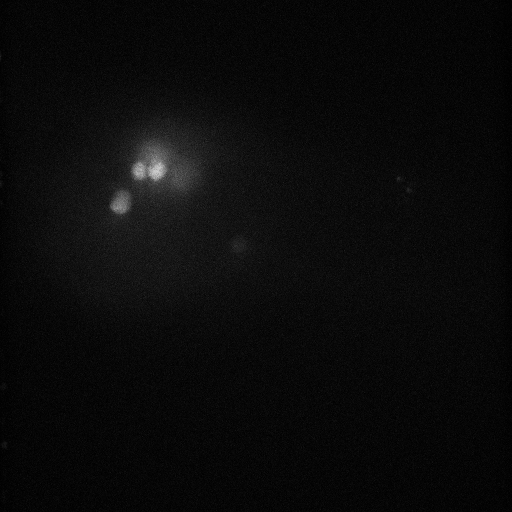

Supplement: Supplementary file 11 — Source data Fig. 2 [file 44318_2024_118_MOESM11_ESM.zip › Figure2/Figure 2A Micr. image/20201128 osm-3 G444E-gfp; HIS-54-BFP_3/Pos0/img_000000000_Confocal-405_008.tif]

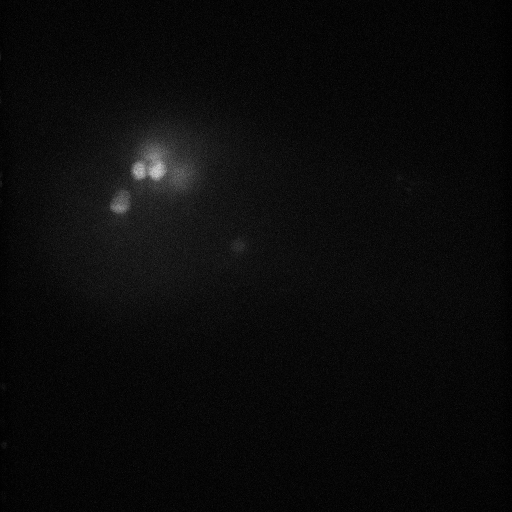

Supplement: Supplementary file 11 — Source data Fig. 2 [file 44318_2024_118_MOESM11_ESM.zip › Figure2/Figure 2A Micr. image/20201128 osm-3 G444E-gfp; HIS-54-BFP_3/Pos0/img_000000000_Confocal-405_009.tif]

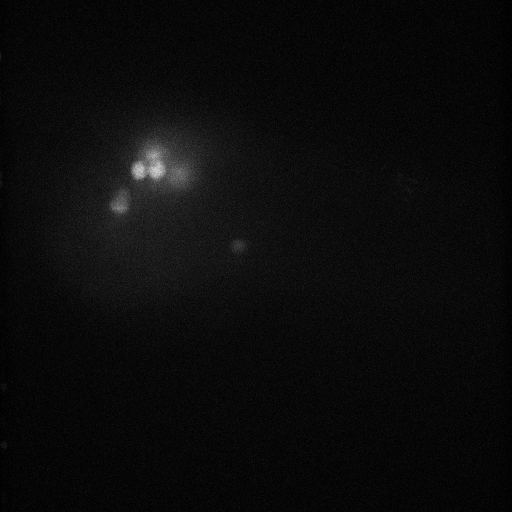

Supplement: Supplementary file 11 — Source data Fig. 2 [file 44318_2024_118_MOESM11_ESM.zip › Figure2/Figure 2A Micr. image/20201128 osm-3 G444E-gfp; HIS-54-BFP_3/Pos0/img_000000000_Confocal-405_010.tif]

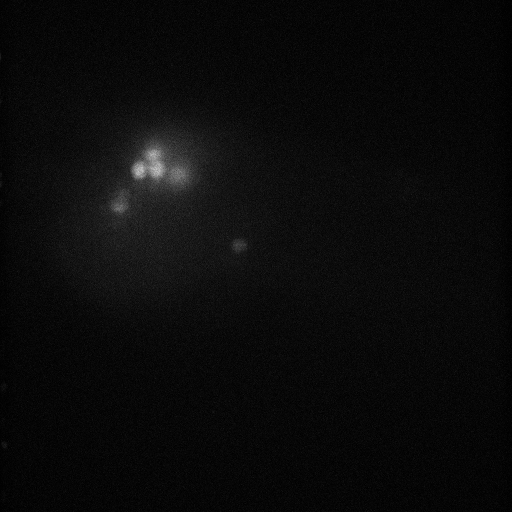

Supplement: Supplementary file 11 — Source data Fig. 2 [file 44318_2024_118_MOESM11_ESM.zip › Figure2/Figure 2A Micr. image/20201128 osm-3 G444E-gfp; HIS-54-BFP_3/Pos0/img_000000000_Confocal-405_011.tif]

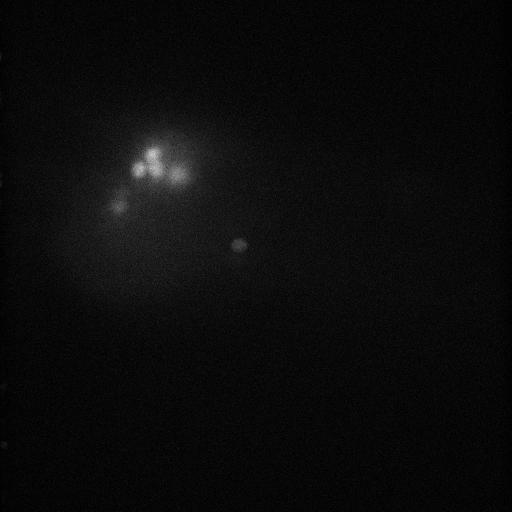

Supplement: Supplementary file 11 — Source data Fig. 2 [file 44318_2024_118_MOESM11_ESM.zip › Figure2/Figure 2A Micr. image/20201128 osm-3 G444E-gfp; HIS-54-BFP_3/Pos0/img_000000000_Confocal-405_012.tif]

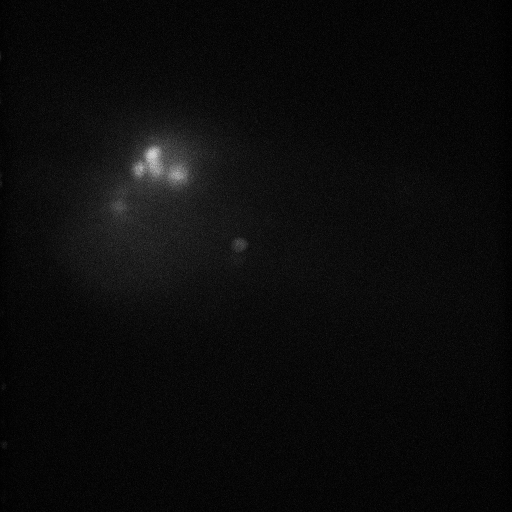

Supplement: Supplementary file 11 — Source data Fig. 2 [file 44318_2024_118_MOESM11_ESM.zip › Figure2/Figure 2A Micr. image/20201128 osm-3 G444E-gfp; HIS-54-BFP_3/Pos0/img_000000000_Confocal-405_013.tif]

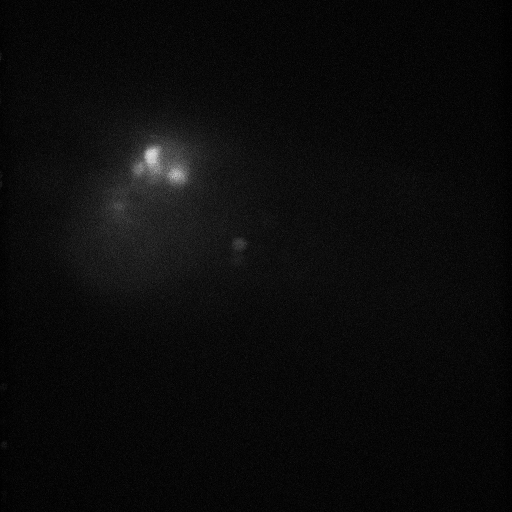

Supplement: Supplementary file 11 — Source data Fig. 2 [file 44318_2024_118_MOESM11_ESM.zip › Figure2/Figure 2A Micr. image/20201128 osm-3 G444E-gfp; HIS-54-BFP_3/Pos0/img_000000000_Confocal-405_014.tif]

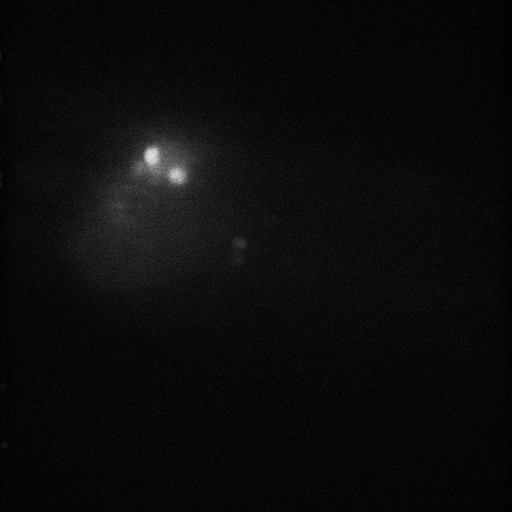

Supplement: Supplementary file 11 — Source data Fig. 2 [file 44318_2024_118_MOESM11_ESM.zip › Figure2/Figure 2A Micr. image/20201128 osm-3 G444E-gfp; HIS-54-BFP_3/Pos0/img_000000000_Confocal-405_015.tif]

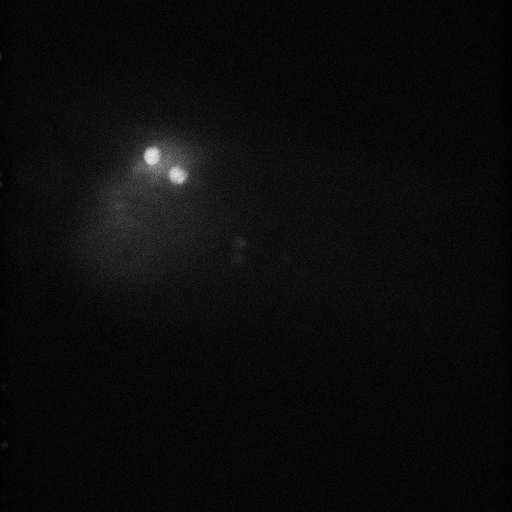

Supplement: Supplementary file 11 — Source data Fig. 2 [file 44318_2024_118_MOESM11_ESM.zip › Figure2/Figure 2A Micr. image/20201128 osm-3 G444E-gfp; HIS-54-BFP_3/Pos0/img_000000000_Confocal-405_016.tif]

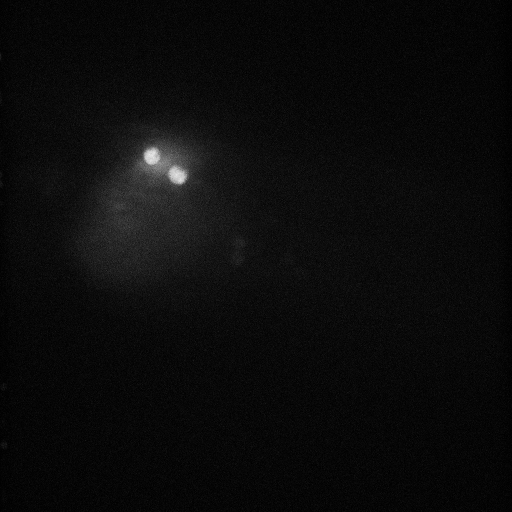

Supplement: Supplementary file 11 — Source data Fig. 2 [file 44318_2024_118_MOESM11_ESM.zip › Figure2/Figure 2A Micr. image/20201128 osm-3 G444E-gfp; HIS-54-BFP_3/Pos0/img_000000000_Confocal-405_017.tif]

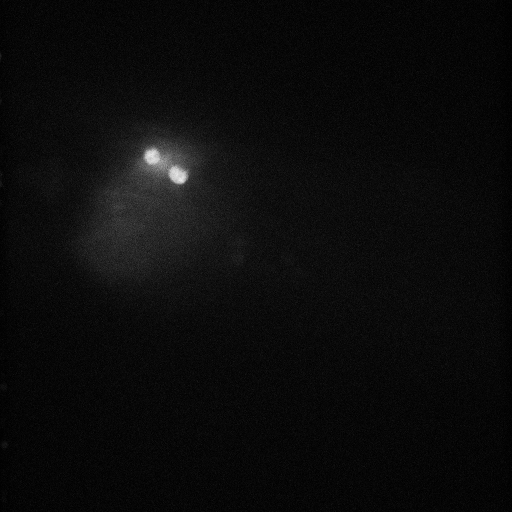

Supplement: Supplementary file 11 — Source data Fig. 2 [file 44318_2024_118_MOESM11_ESM.zip › Figure2/Figure 2A Micr. image/20201128 osm-3 G444E-gfp; HIS-54-BFP_3/Pos0/img_000000000_Confocal-405_018.tif]

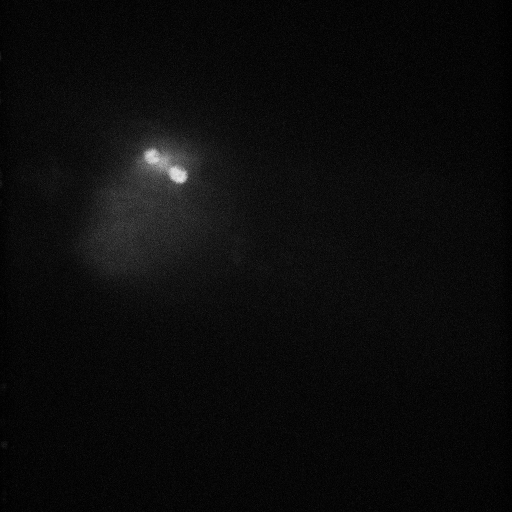

Supplement: Supplementary file 11 — Source data Fig. 2 [file 44318_2024_118_MOESM11_ESM.zip › Figure2/Figure 2A Micr. image/20201128 osm-3 G444E-gfp; HIS-54-BFP_3/Pos0/img_000000000_Confocal-405_019.tif]
