# Supplementary figures and images for: Neurons dispose of hyperactive kinesin into glial cells for clearance (part 1 of 9)
Source: EMBO J. 2024 May 28;43(13):5. doi: 10.1038/s44318-024-00118-0 (PMC11217292; doi:10.1038/s44318-024-00118-0)

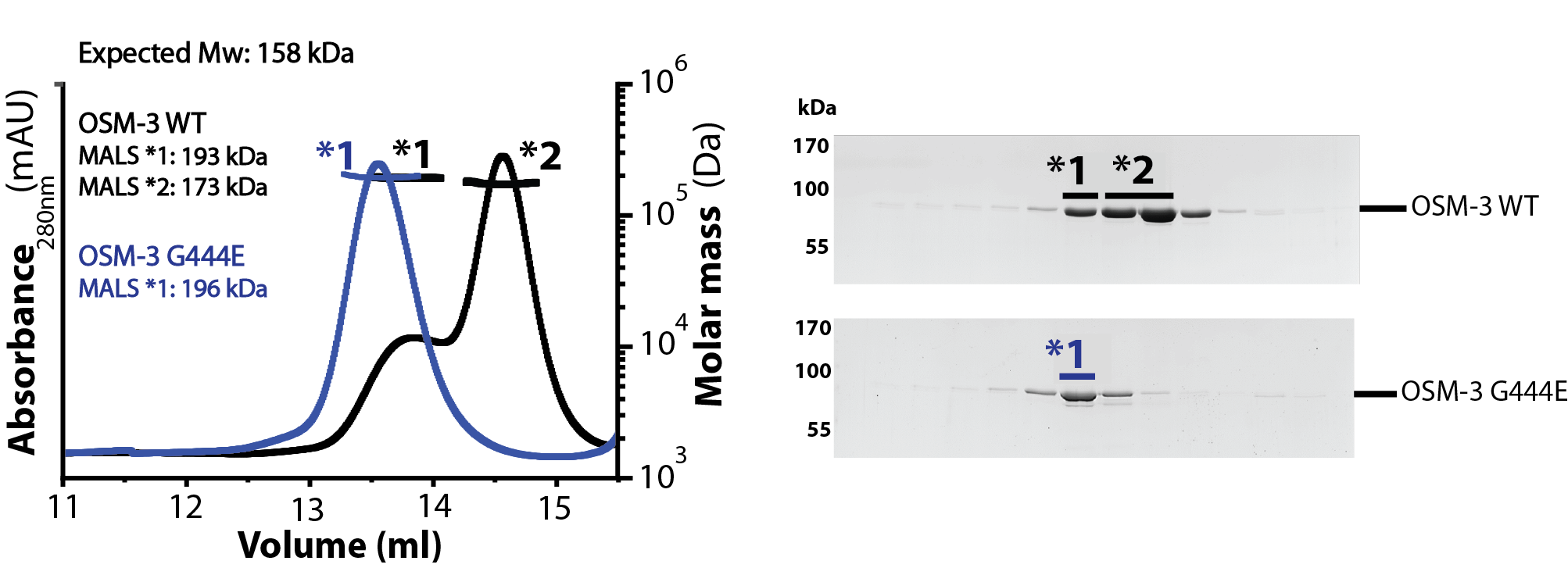

Supplement: Supplementary file 10 — Source data Fig. 1 [file 44318_2024_118_MOESM10_ESM.zip › Figure 1D Blot/Osm3+wt+and+ee+sec+mals_corrected.png]

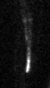

Supplement: Supplementary file 10 — Source data Fig. 1 [file 44318_2024_118_MOESM10_ESM.zip › Figure 1F Micr. image/AVG_20230224 Scarlet-che-3; osm-3-gfp_2-3 phasmid 50x88.tif]

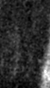

Supplement: Supplementary file 10 — Source data Fig. 1 [file 44318_2024_118_MOESM10_ESM.zip › Figure 1F Micr. image/AVG_20230324 Scarlet-che-3; osm-3-g444e-gfp_2-1 phasmid 50x88.tif]

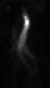

Supplement: Supplementary file 10 — Source data Fig. 1 [file 44318_2024_118_MOESM10_ESM.zip › Figure 1F Micr. image/AVG_20230224 Scarlet-che-3; osm-3-gfp_1-2 amphid 50x88.tif]

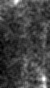

Supplement: Supplementary file 10 — Source data Fig. 1 [file 44318_2024_118_MOESM10_ESM.zip › Figure 1F Micr. image/AVG_20230306 OSM-3-G444E-GFP; Scarlet-CHE-3_13-1 amphid 50x88.tif]

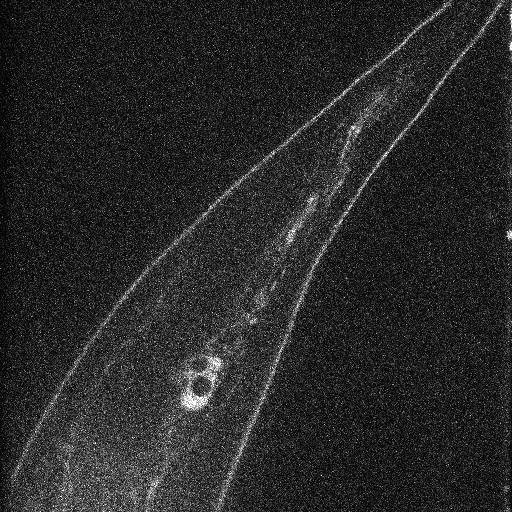

Supplement: Supplementary file 10 — Source data Fig. 1 [file 44318_2024_118_MOESM10_ESM.zip › Figure 1F Micr. image/20230224 Scarlet-che-3; osm-3-gfp_2 phasmid/img_000000000_L-488_006.tif]

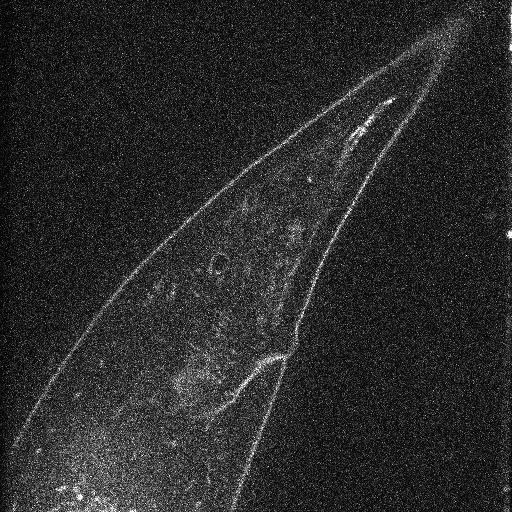

Supplement: Supplementary file 10 — Source data Fig. 1 [file 44318_2024_118_MOESM10_ESM.zip › Figure 1F Micr. image/20230224 Scarlet-che-3; osm-3-gfp_2 phasmid/img_000000000_L-488_012.tif]

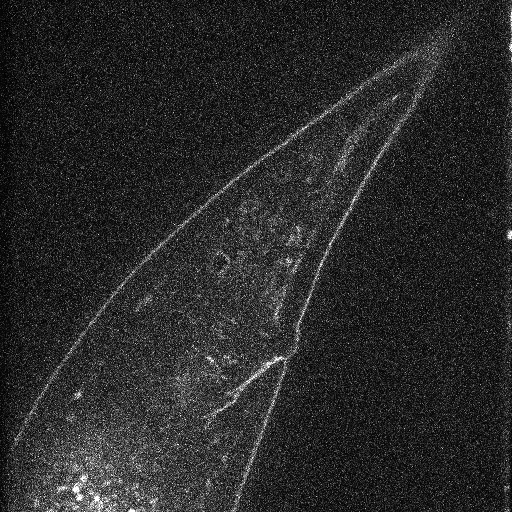

Supplement: Supplementary file 10 — Source data Fig. 1 [file 44318_2024_118_MOESM10_ESM.zip › Figure 1F Micr. image/20230224 Scarlet-che-3; osm-3-gfp_2 phasmid/img_000000000_L-488_013.tif]

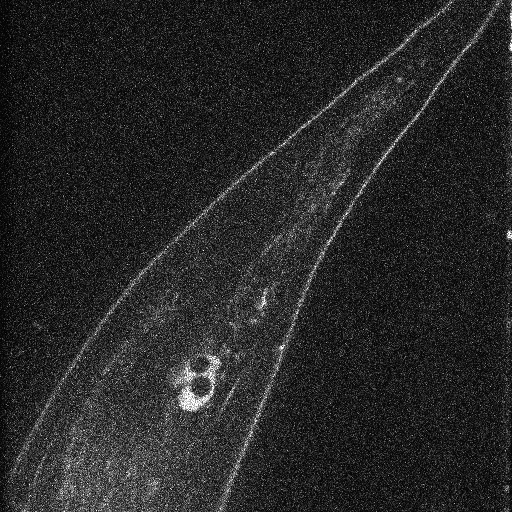

Supplement: Supplementary file 10 — Source data Fig. 1 [file 44318_2024_118_MOESM10_ESM.zip › Figure 1F Micr. image/20230224 Scarlet-che-3; osm-3-gfp_2 phasmid/img_000000000_L-488_007.tif]

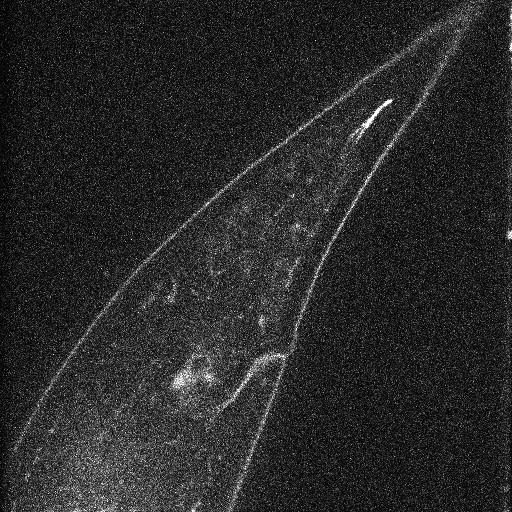

Supplement: Supplementary file 10 — Source data Fig. 1 [file 44318_2024_118_MOESM10_ESM.zip › Figure 1F Micr. image/20230224 Scarlet-che-3; osm-3-gfp_2 phasmid/img_000000000_L-488_011.tif]

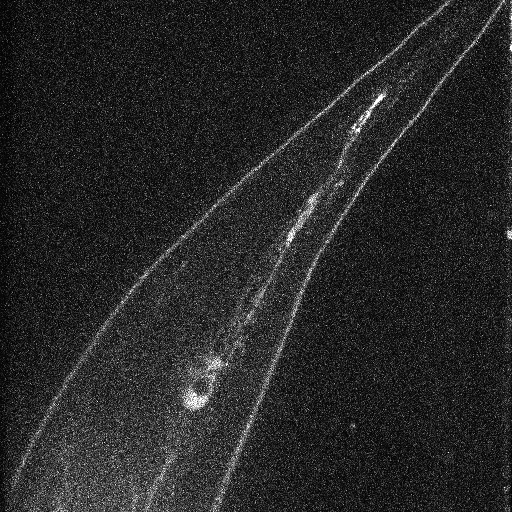

Supplement: Supplementary file 10 — Source data Fig. 1 [file 44318_2024_118_MOESM10_ESM.zip › Figure 1F Micr. image/20230224 Scarlet-che-3; osm-3-gfp_2 phasmid/img_000000000_L-488_005.tif]

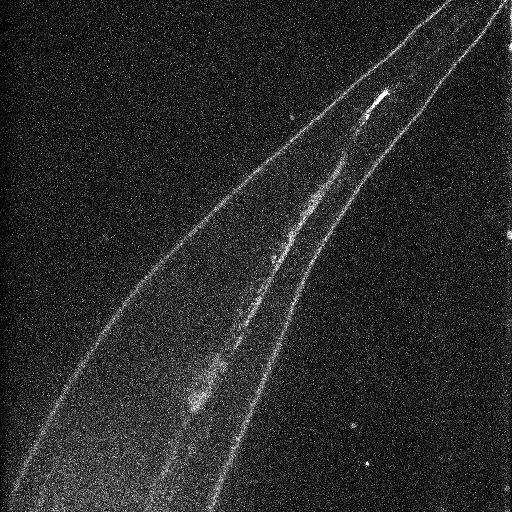

Supplement: Supplementary file 10 — Source data Fig. 1 [file 44318_2024_118_MOESM10_ESM.zip › Figure 1F Micr. image/20230224 Scarlet-che-3; osm-3-gfp_2 phasmid/img_000000000_L-488_004.tif]

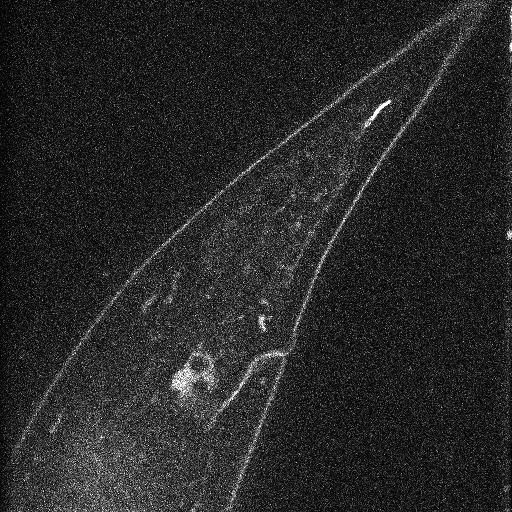

Supplement: Supplementary file 10 — Source data Fig. 1 [file 44318_2024_118_MOESM10_ESM.zip › Figure 1F Micr. image/20230224 Scarlet-che-3; osm-3-gfp_2 phasmid/img_000000000_L-488_010.tif]

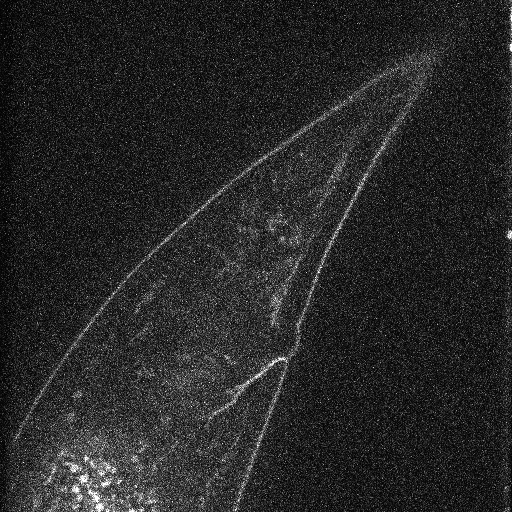

Supplement: Supplementary file 10 — Source data Fig. 1 [file 44318_2024_118_MOESM10_ESM.zip › Figure 1F Micr. image/20230224 Scarlet-che-3; osm-3-gfp_2 phasmid/img_000000000_L-488_014.tif]

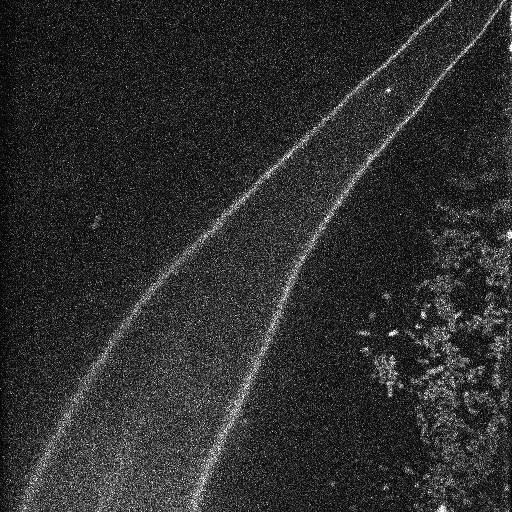

Supplement: Supplementary file 10 — Source data Fig. 1 [file 44318_2024_118_MOESM10_ESM.zip › Figure 1F Micr. image/20230224 Scarlet-che-3; osm-3-gfp_2 phasmid/img_000000000_L-488_000.tif]

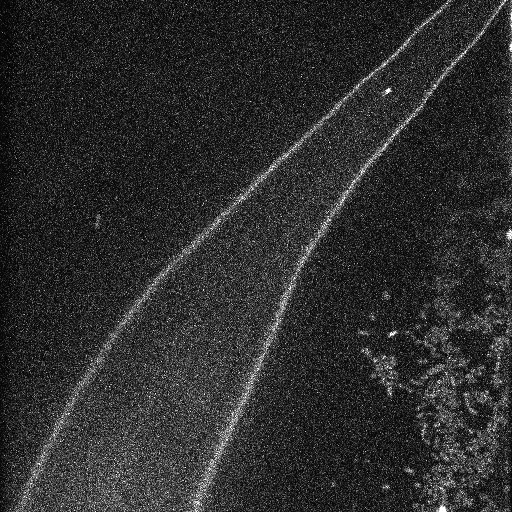

Supplement: Supplementary file 10 — Source data Fig. 1 [file 44318_2024_118_MOESM10_ESM.zip › Figure 1F Micr. image/20230224 Scarlet-che-3; osm-3-gfp_2 phasmid/img_000000000_L-488_001.tif]

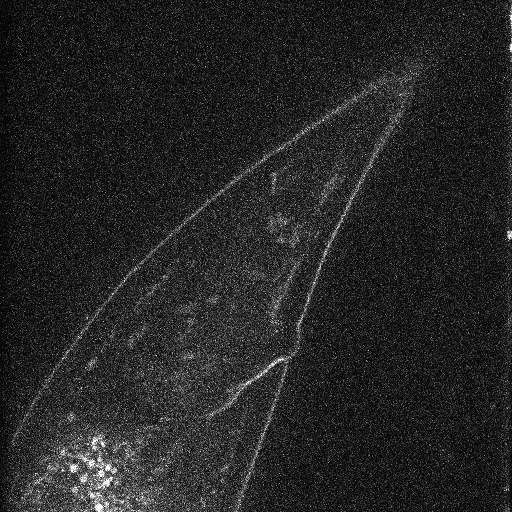

Supplement: Supplementary file 10 — Source data Fig. 1 [file 44318_2024_118_MOESM10_ESM.zip › Figure 1F Micr. image/20230224 Scarlet-che-3; osm-3-gfp_2 phasmid/img_000000000_L-488_015.tif]

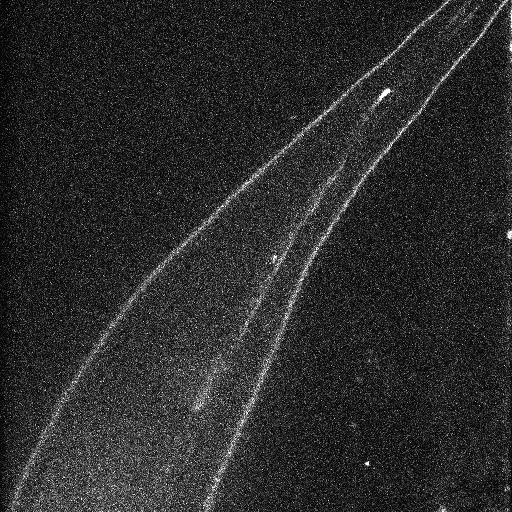

Supplement: Supplementary file 10 — Source data Fig. 1 [file 44318_2024_118_MOESM10_ESM.zip › Figure 1F Micr. image/20230224 Scarlet-che-3; osm-3-gfp_2 phasmid/img_000000000_L-488_003.tif]

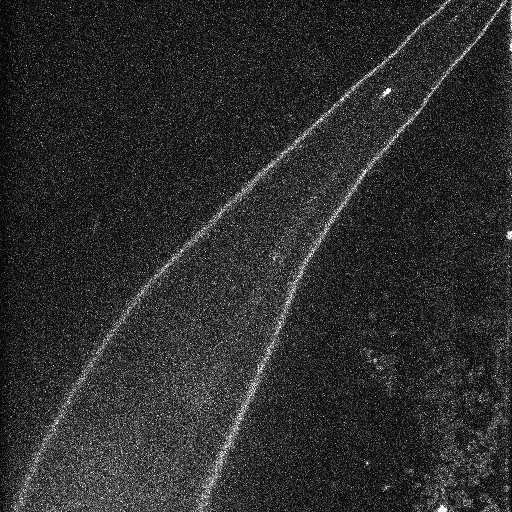

Supplement: Supplementary file 10 — Source data Fig. 1 [file 44318_2024_118_MOESM10_ESM.zip › Figure 1F Micr. image/20230224 Scarlet-che-3; osm-3-gfp_2 phasmid/img_000000000_L-488_002.tif]

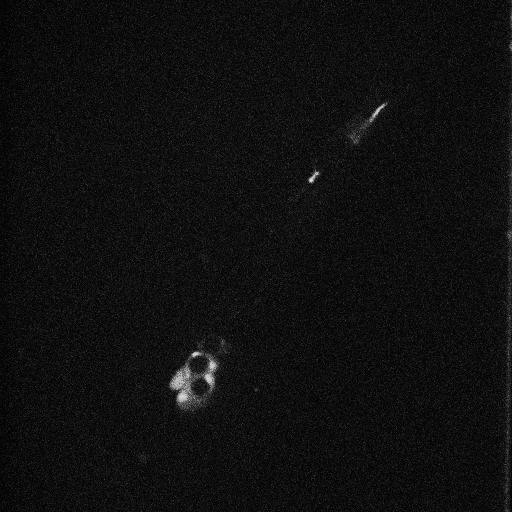

Supplement: Supplementary file 10 — Source data Fig. 1 [file 44318_2024_118_MOESM10_ESM.zip › Figure 1F Micr. image/20230224 Scarlet-che-3; osm-3-gfp_2 phasmid/img_000000000_L-561_009.tif]

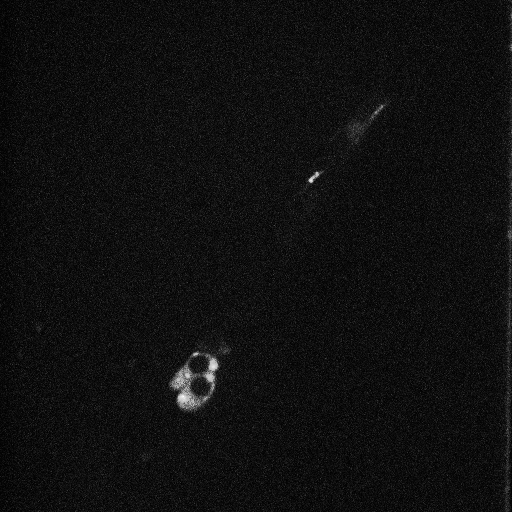

Supplement: Supplementary file 10 — Source data Fig. 1 [file 44318_2024_118_MOESM10_ESM.zip › Figure 1F Micr. image/20230224 Scarlet-che-3; osm-3-gfp_2 phasmid/img_000000000_L-561_008.tif]

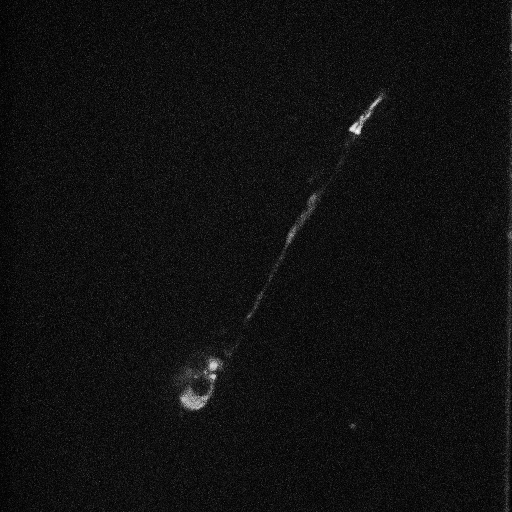

Supplement: Supplementary file 10 — Source data Fig. 1 [file 44318_2024_118_MOESM10_ESM.zip › Figure 1F Micr. image/20230224 Scarlet-che-3; osm-3-gfp_2 phasmid/img_000000000_L-561_005.tif]

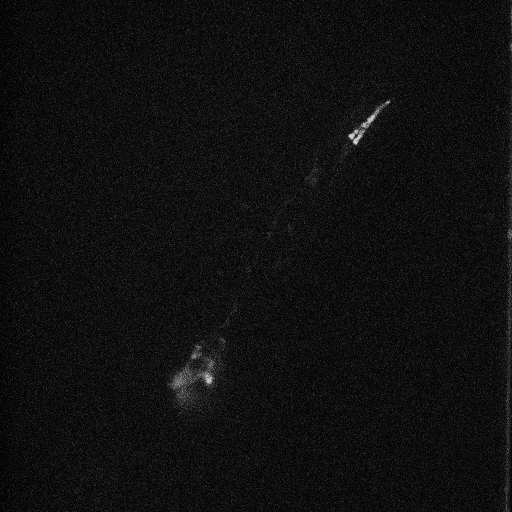

Supplement: Supplementary file 10 — Source data Fig. 1 [file 44318_2024_118_MOESM10_ESM.zip › Figure 1F Micr. image/20230224 Scarlet-che-3; osm-3-gfp_2 phasmid/img_000000000_L-561_011.tif]

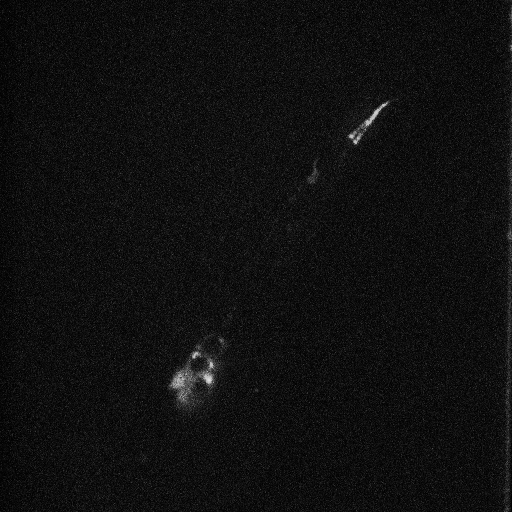

Supplement: Supplementary file 10 — Source data Fig. 1 [file 44318_2024_118_MOESM10_ESM.zip › Figure 1F Micr. image/20230224 Scarlet-che-3; osm-3-gfp_2 phasmid/img_000000000_L-561_010.tif]

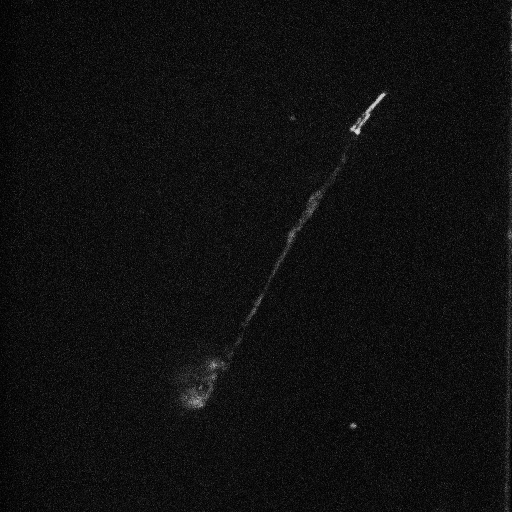

Supplement: Supplementary file 10 — Source data Fig. 1 [file 44318_2024_118_MOESM10_ESM.zip › Figure 1F Micr. image/20230224 Scarlet-che-3; osm-3-gfp_2 phasmid/img_000000000_L-561_004.tif]

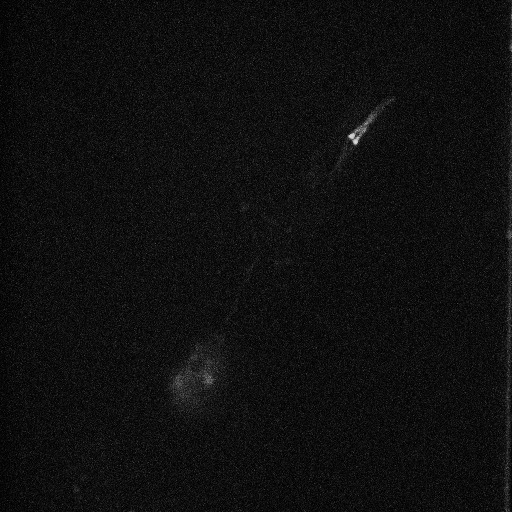

Supplement: Supplementary file 10 — Source data Fig. 1 [file 44318_2024_118_MOESM10_ESM.zip › Figure 1F Micr. image/20230224 Scarlet-che-3; osm-3-gfp_2 phasmid/img_000000000_L-561_012.tif]

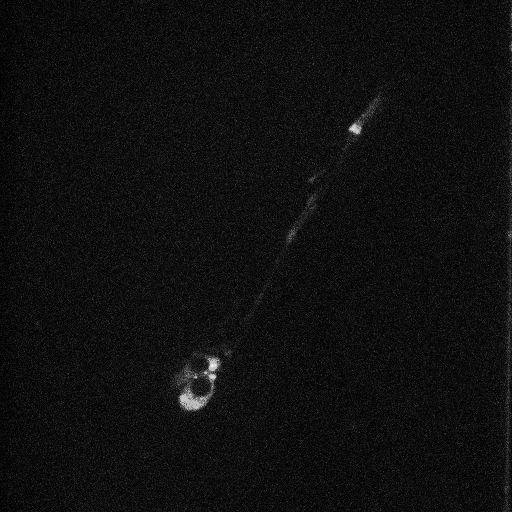

Supplement: Supplementary file 10 — Source data Fig. 1 [file 44318_2024_118_MOESM10_ESM.zip › Figure 1F Micr. image/20230224 Scarlet-che-3; osm-3-gfp_2 phasmid/img_000000000_L-561_006.tif]

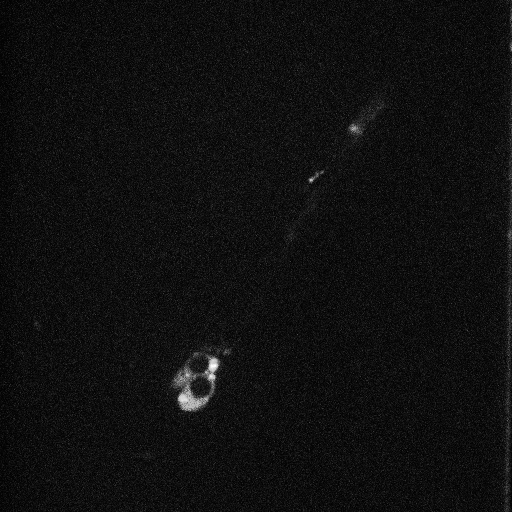

Supplement: Supplementary file 10 — Source data Fig. 1 [file 44318_2024_118_MOESM10_ESM.zip › Figure 1F Micr. image/20230224 Scarlet-che-3; osm-3-gfp_2 phasmid/img_000000000_L-561_007.tif]

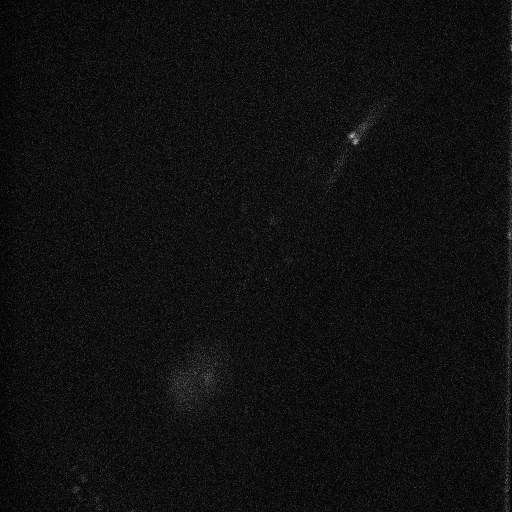

Supplement: Supplementary file 10 — Source data Fig. 1 [file 44318_2024_118_MOESM10_ESM.zip › Figure 1F Micr. image/20230224 Scarlet-che-3; osm-3-gfp_2 phasmid/img_000000000_L-561_013.tif]

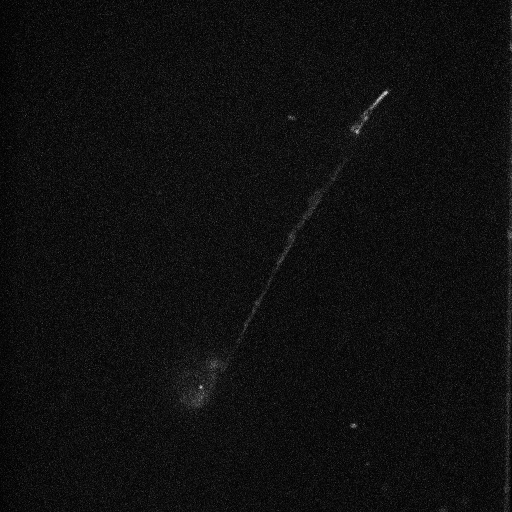

Supplement: Supplementary file 10 — Source data Fig. 1 [file 44318_2024_118_MOESM10_ESM.zip › Figure 1F Micr. image/20230224 Scarlet-che-3; osm-3-gfp_2 phasmid/img_000000000_L-561_003.tif]

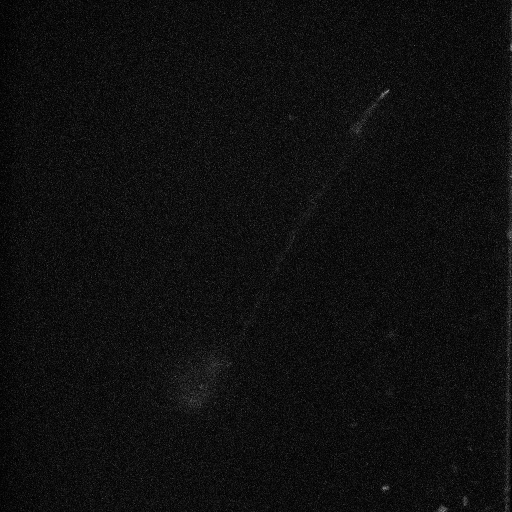

Supplement: Supplementary file 10 — Source data Fig. 1 [file 44318_2024_118_MOESM10_ESM.zip › Figure 1F Micr. image/20230224 Scarlet-che-3; osm-3-gfp_2 phasmid/img_000000000_L-561_002.tif]

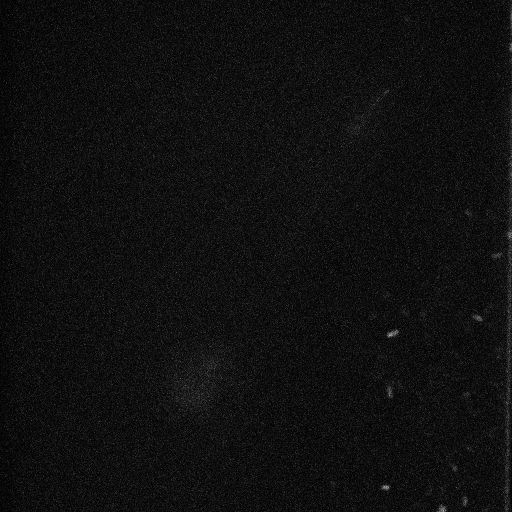

Supplement: Supplementary file 10 — Source data Fig. 1 [file 44318_2024_118_MOESM10_ESM.zip › Figure 1F Micr. image/20230224 Scarlet-che-3; osm-3-gfp_2 phasmid/img_000000000_L-561_000.tif]

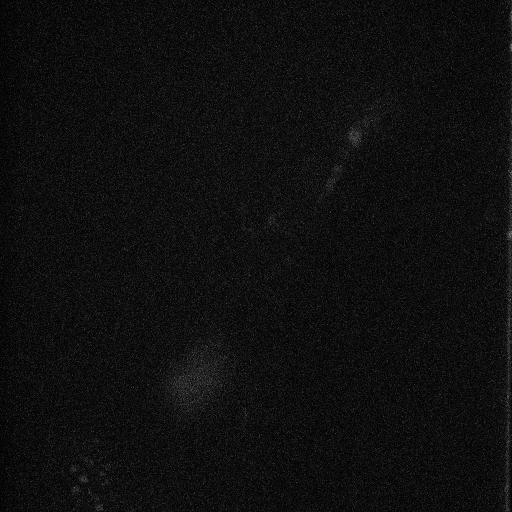

Supplement: Supplementary file 10 — Source data Fig. 1 [file 44318_2024_118_MOESM10_ESM.zip › Figure 1F Micr. image/20230224 Scarlet-che-3; osm-3-gfp_2 phasmid/img_000000000_L-561_014.tif]

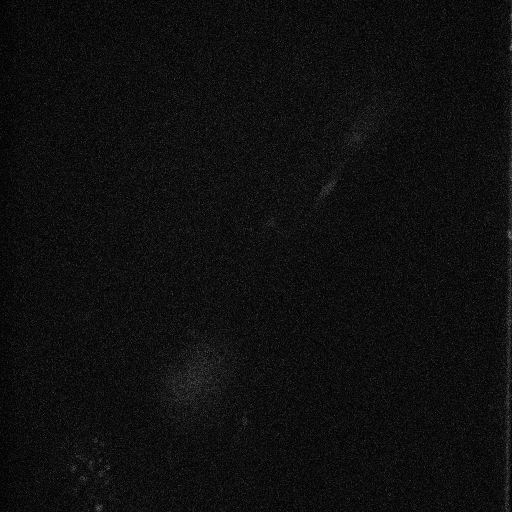

Supplement: Supplementary file 10 — Source data Fig. 1 [file 44318_2024_118_MOESM10_ESM.zip › Figure 1F Micr. image/20230224 Scarlet-che-3; osm-3-gfp_2 phasmid/img_000000000_L-561_015.tif]

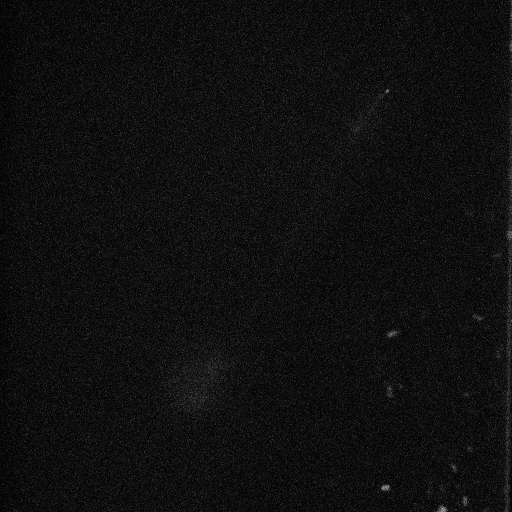

Supplement: Supplementary file 10 — Source data Fig. 1 [file 44318_2024_118_MOESM10_ESM.zip › Figure 1F Micr. image/20230224 Scarlet-che-3; osm-3-gfp_2 phasmid/img_000000000_L-561_001.tif]

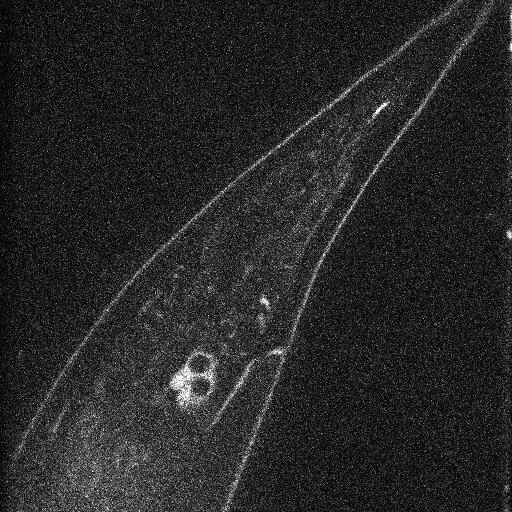

Supplement: Supplementary file 10 — Source data Fig. 1 [file 44318_2024_118_MOESM10_ESM.zip › Figure 1F Micr. image/20230224 Scarlet-che-3; osm-3-gfp_2 phasmid/img_000000000_L-488_009.tif]

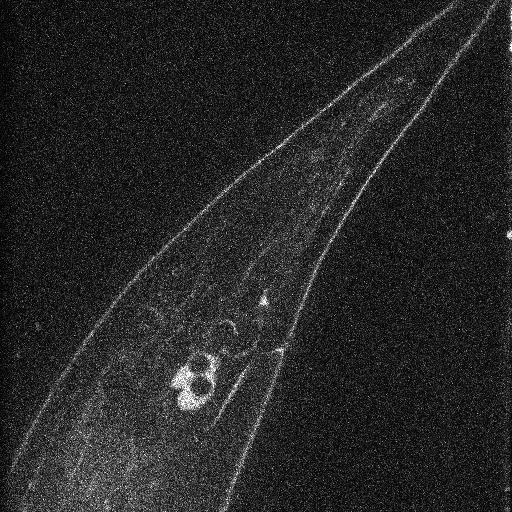

Supplement: Supplementary file 10 — Source data Fig. 1 [file 44318_2024_118_MOESM10_ESM.zip › Figure 1F Micr. image/20230224 Scarlet-che-3; osm-3-gfp_2 phasmid/img_000000000_L-488_008.tif]

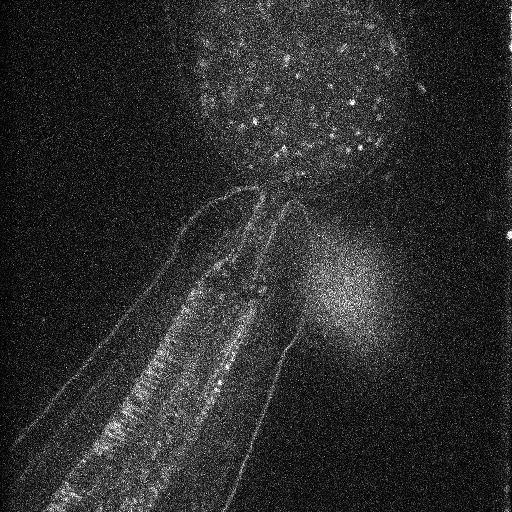

Supplement: Supplementary file 10 — Source data Fig. 1 [file 44318_2024_118_MOESM10_ESM.zip › Figure 1F Micr. image/20230306 OSM-3-G444E-GFP; Scarlet-CHE-3_13 amphid/img_000000000_L-488_006.tif]

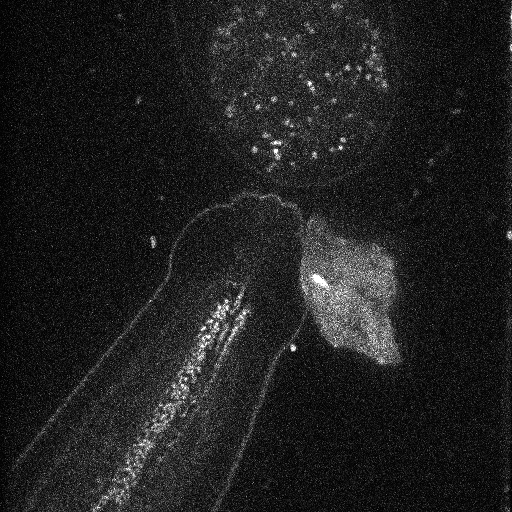

Supplement: Supplementary file 10 — Source data Fig. 1 [file 44318_2024_118_MOESM10_ESM.zip › Figure 1F Micr. image/20230306 OSM-3-G444E-GFP; Scarlet-CHE-3_13 amphid/img_000000000_L-488_012.tif]

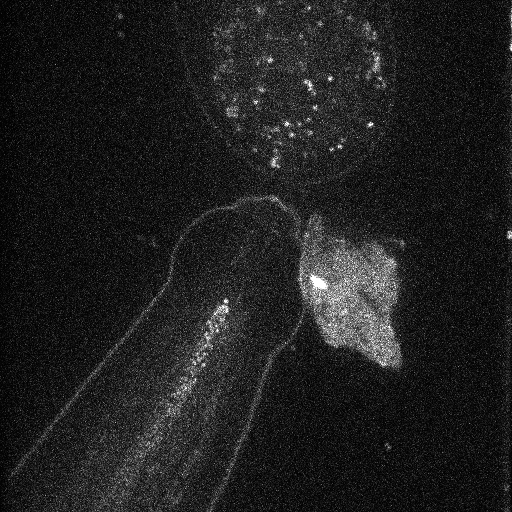

Supplement: Supplementary file 10 — Source data Fig. 1 [file 44318_2024_118_MOESM10_ESM.zip › Figure 1F Micr. image/20230306 OSM-3-G444E-GFP; Scarlet-CHE-3_13 amphid/img_000000000_L-488_013.tif]

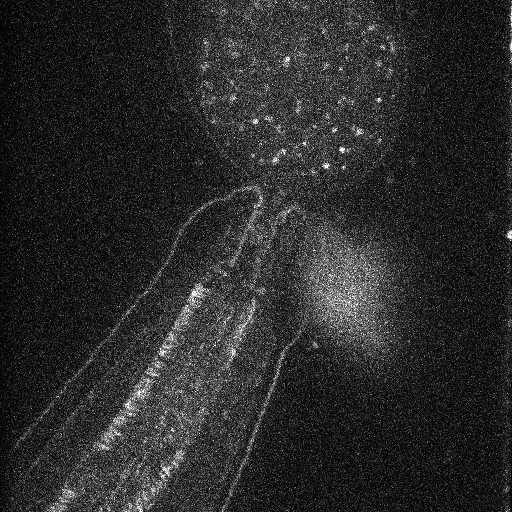

Supplement: Supplementary file 10 — Source data Fig. 1 [file 44318_2024_118_MOESM10_ESM.zip › Figure 1F Micr. image/20230306 OSM-3-G444E-GFP; Scarlet-CHE-3_13 amphid/img_000000000_L-488_007.tif]

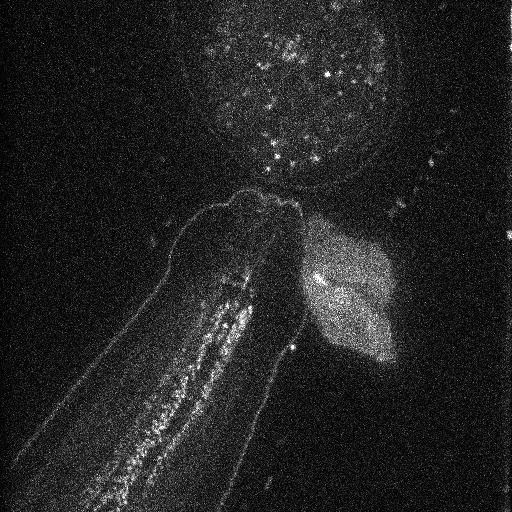

Supplement: Supplementary file 10 — Source data Fig. 1 [file 44318_2024_118_MOESM10_ESM.zip › Figure 1F Micr. image/20230306 OSM-3-G444E-GFP; Scarlet-CHE-3_13 amphid/img_000000000_L-488_011.tif]

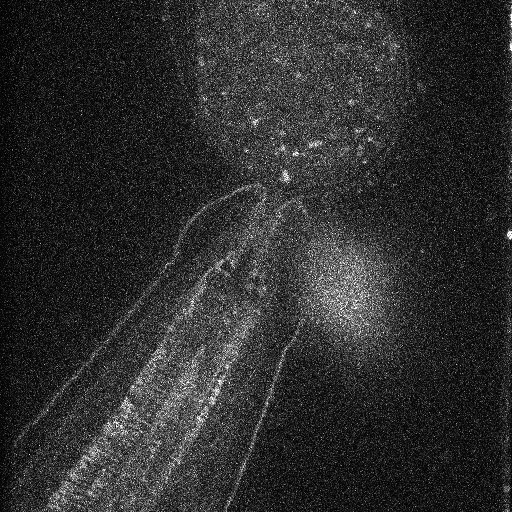

Supplement: Supplementary file 10 — Source data Fig. 1 [file 44318_2024_118_MOESM10_ESM.zip › Figure 1F Micr. image/20230306 OSM-3-G444E-GFP; Scarlet-CHE-3_13 amphid/img_000000000_L-488_005.tif]

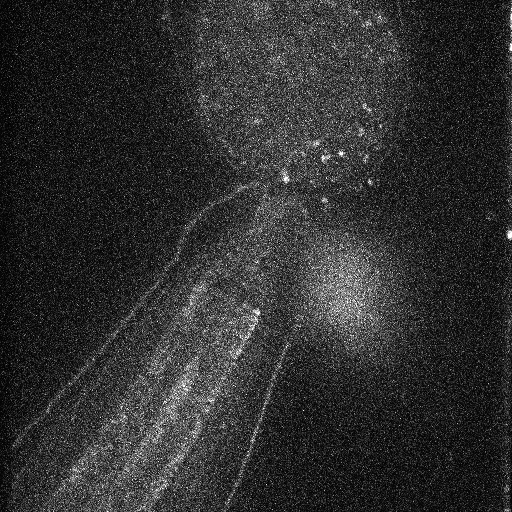

Supplement: Supplementary file 10 — Source data Fig. 1 [file 44318_2024_118_MOESM10_ESM.zip › Figure 1F Micr. image/20230306 OSM-3-G444E-GFP; Scarlet-CHE-3_13 amphid/img_000000000_L-488_004.tif]

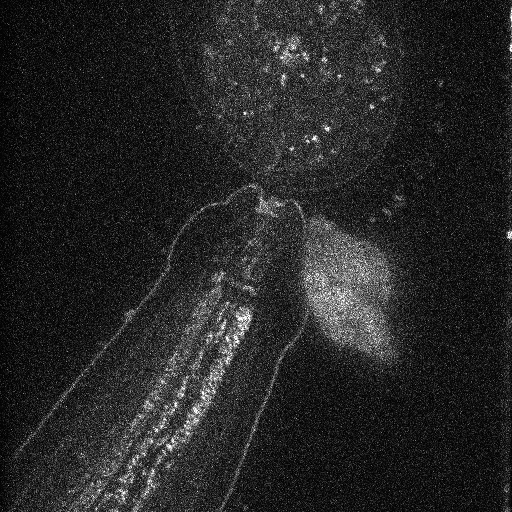

Supplement: Supplementary file 10 — Source data Fig. 1 [file 44318_2024_118_MOESM10_ESM.zip › Figure 1F Micr. image/20230306 OSM-3-G444E-GFP; Scarlet-CHE-3_13 amphid/img_000000000_L-488_010.tif]

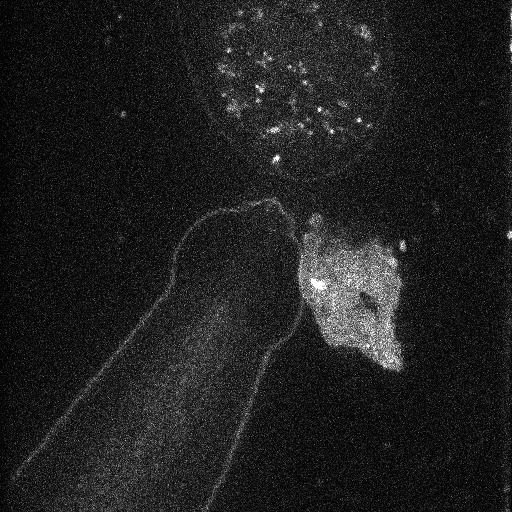

Supplement: Supplementary file 10 — Source data Fig. 1 [file 44318_2024_118_MOESM10_ESM.zip › Figure 1F Micr. image/20230306 OSM-3-G444E-GFP; Scarlet-CHE-3_13 amphid/img_000000000_L-488_014.tif]

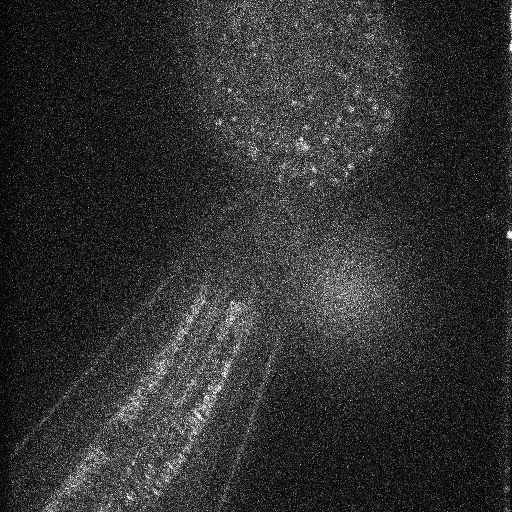

Supplement: Supplementary file 10 — Source data Fig. 1 [file 44318_2024_118_MOESM10_ESM.zip › Figure 1F Micr. image/20230306 OSM-3-G444E-GFP; Scarlet-CHE-3_13 amphid/img_000000000_L-488_000.tif]

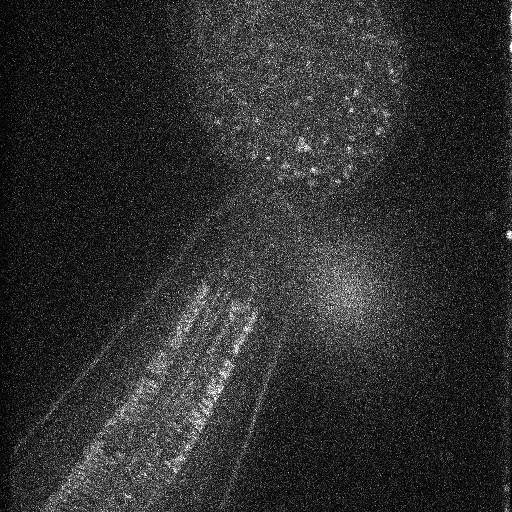

Supplement: Supplementary file 10 — Source data Fig. 1 [file 44318_2024_118_MOESM10_ESM.zip › Figure 1F Micr. image/20230306 OSM-3-G444E-GFP; Scarlet-CHE-3_13 amphid/img_000000000_L-488_001.tif]

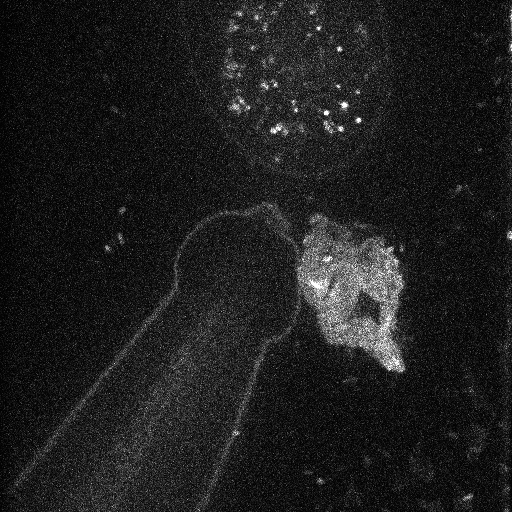

Supplement: Supplementary file 10 — Source data Fig. 1 [file 44318_2024_118_MOESM10_ESM.zip › Figure 1F Micr. image/20230306 OSM-3-G444E-GFP; Scarlet-CHE-3_13 amphid/img_000000000_L-488_015.tif]

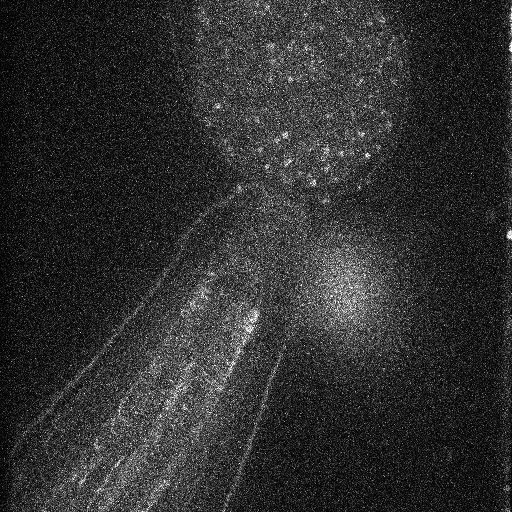

Supplement: Supplementary file 10 — Source data Fig. 1 [file 44318_2024_118_MOESM10_ESM.zip › Figure 1F Micr. image/20230306 OSM-3-G444E-GFP; Scarlet-CHE-3_13 amphid/img_000000000_L-488_003.tif]

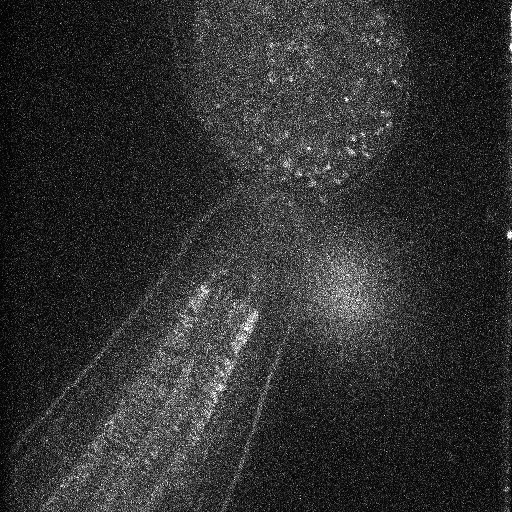

Supplement: Supplementary file 10 — Source data Fig. 1 [file 44318_2024_118_MOESM10_ESM.zip › Figure 1F Micr. image/20230306 OSM-3-G444E-GFP; Scarlet-CHE-3_13 amphid/img_000000000_L-488_002.tif]

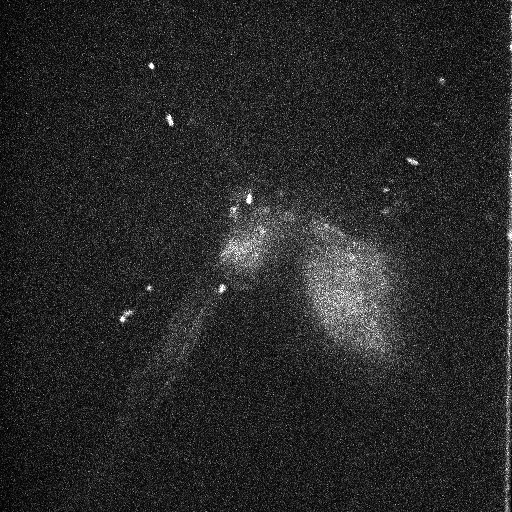

Supplement: Supplementary file 10 — Source data Fig. 1 [file 44318_2024_118_MOESM10_ESM.zip › Figure 1F Micr. image/20230306 OSM-3-G444E-GFP; Scarlet-CHE-3_13 amphid/img_000000000_L-561_009.tif]

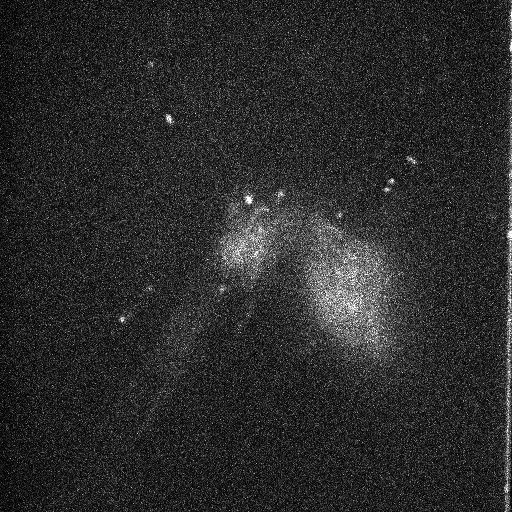

Supplement: Supplementary file 10 — Source data Fig. 1 [file 44318_2024_118_MOESM10_ESM.zip › Figure 1F Micr. image/20230306 OSM-3-G444E-GFP; Scarlet-CHE-3_13 amphid/img_000000000_L-561_008.tif]

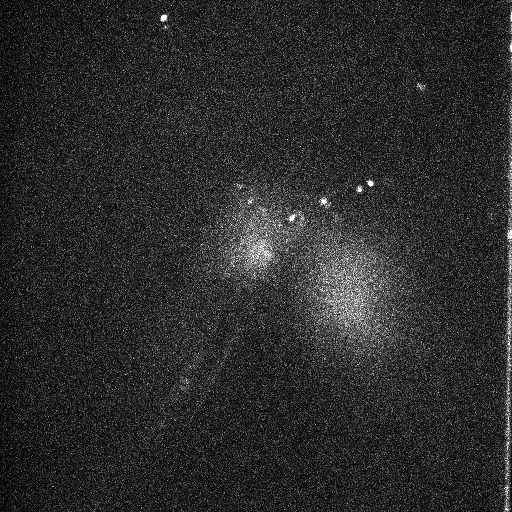

Supplement: Supplementary file 10 — Source data Fig. 1 [file 44318_2024_118_MOESM10_ESM.zip › Figure 1F Micr. image/20230306 OSM-3-G444E-GFP; Scarlet-CHE-3_13 amphid/img_000000000_L-561_005.tif]

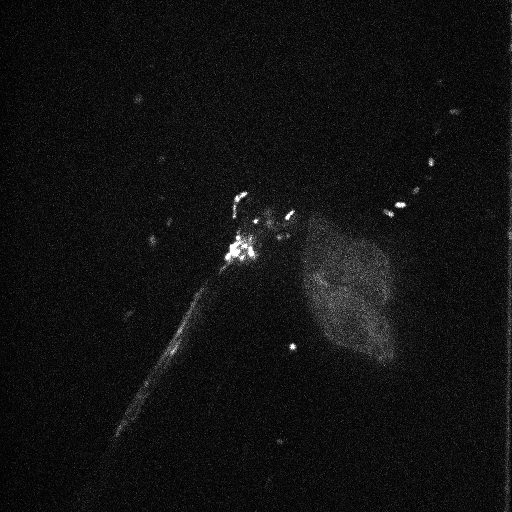

Supplement: Supplementary file 10 — Source data Fig. 1 [file 44318_2024_118_MOESM10_ESM.zip › Figure 1F Micr. image/20230306 OSM-3-G444E-GFP; Scarlet-CHE-3_13 amphid/img_000000000_L-561_011.tif]

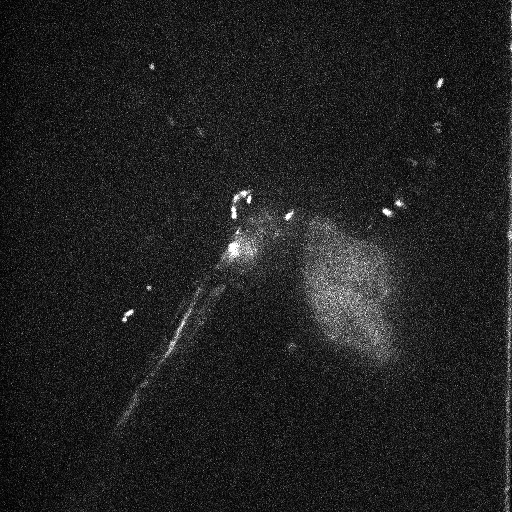

Supplement: Supplementary file 10 — Source data Fig. 1 [file 44318_2024_118_MOESM10_ESM.zip › Figure 1F Micr. image/20230306 OSM-3-G444E-GFP; Scarlet-CHE-3_13 amphid/img_000000000_L-561_010.tif]

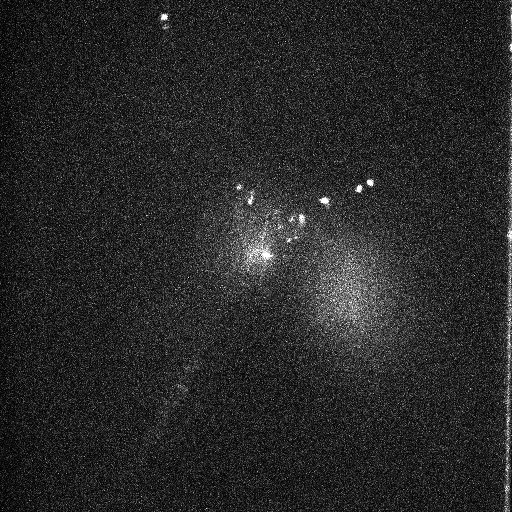

Supplement: Supplementary file 10 — Source data Fig. 1 [file 44318_2024_118_MOESM10_ESM.zip › Figure 1F Micr. image/20230306 OSM-3-G444E-GFP; Scarlet-CHE-3_13 amphid/img_000000000_L-561_004.tif]

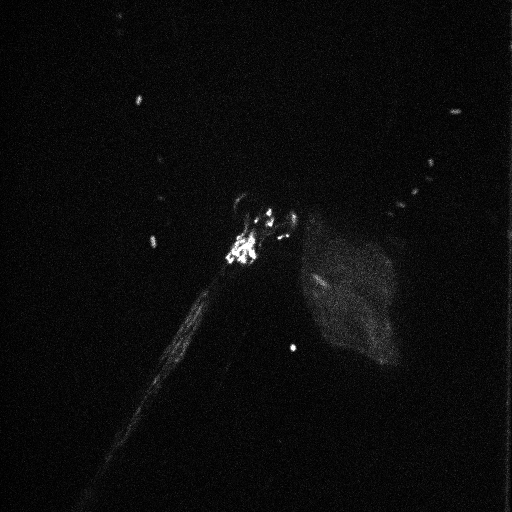

Supplement: Supplementary file 10 — Source data Fig. 1 [file 44318_2024_118_MOESM10_ESM.zip › Figure 1F Micr. image/20230306 OSM-3-G444E-GFP; Scarlet-CHE-3_13 amphid/img_000000000_L-561_012.tif]

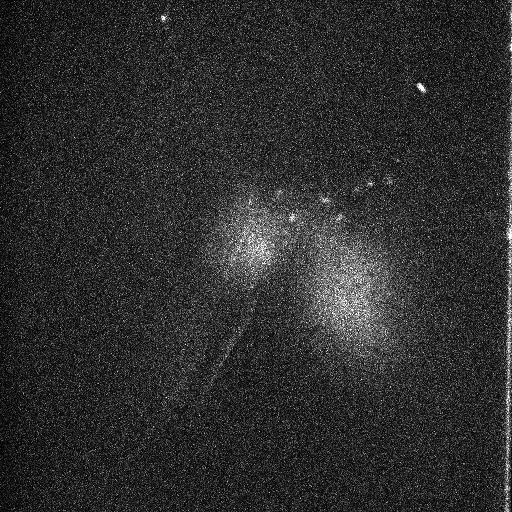

Supplement: Supplementary file 10 — Source data Fig. 1 [file 44318_2024_118_MOESM10_ESM.zip › Figure 1F Micr. image/20230306 OSM-3-G444E-GFP; Scarlet-CHE-3_13 amphid/img_000000000_L-561_006.tif]

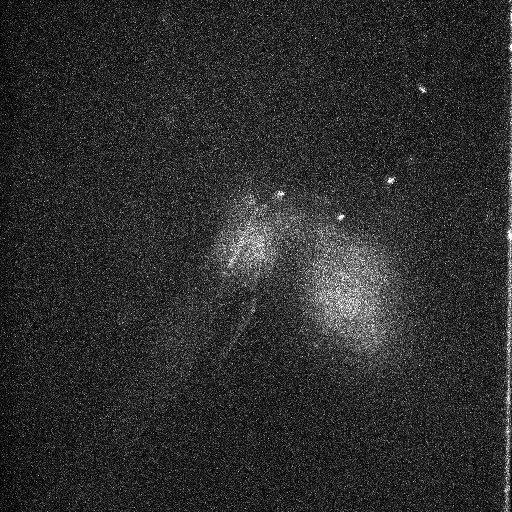

Supplement: Supplementary file 10 — Source data Fig. 1 [file 44318_2024_118_MOESM10_ESM.zip › Figure 1F Micr. image/20230306 OSM-3-G444E-GFP; Scarlet-CHE-3_13 amphid/img_000000000_L-561_007.tif]

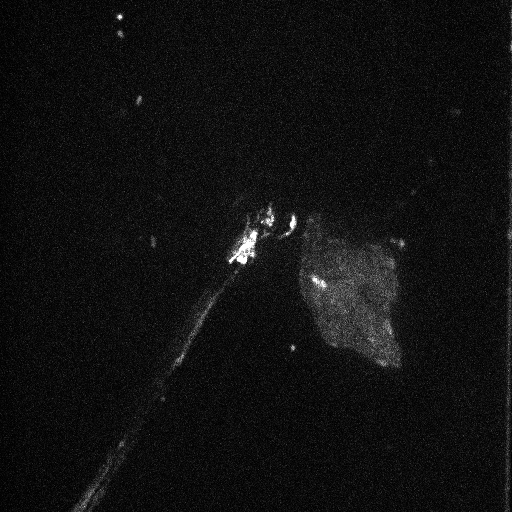

Supplement: Supplementary file 10 — Source data Fig. 1 [file 44318_2024_118_MOESM10_ESM.zip › Figure 1F Micr. image/20230306 OSM-3-G444E-GFP; Scarlet-CHE-3_13 amphid/img_000000000_L-561_013.tif]

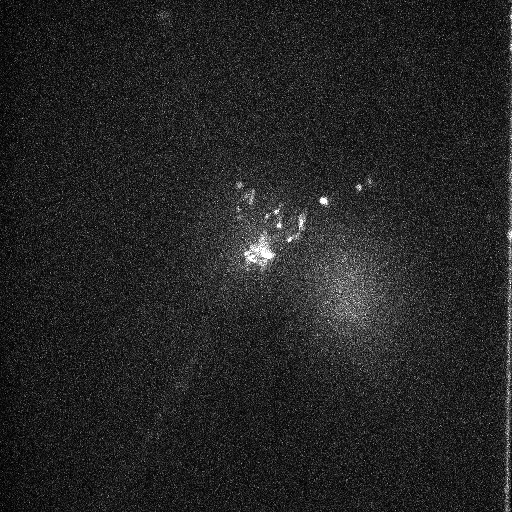

Supplement: Supplementary file 10 — Source data Fig. 1 [file 44318_2024_118_MOESM10_ESM.zip › Figure 1F Micr. image/20230306 OSM-3-G444E-GFP; Scarlet-CHE-3_13 amphid/img_000000000_L-561_003.tif]

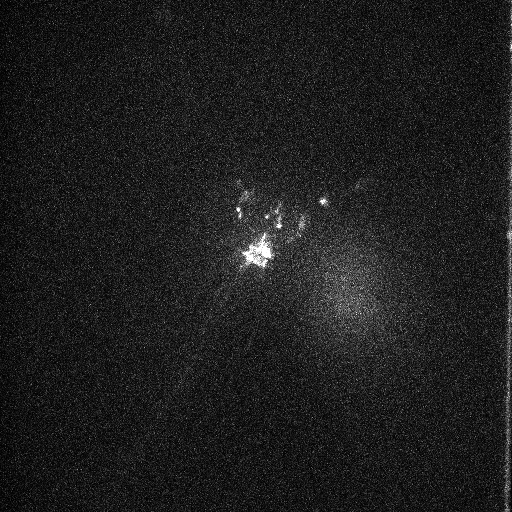

Supplement: Supplementary file 10 — Source data Fig. 1 [file 44318_2024_118_MOESM10_ESM.zip › Figure 1F Micr. image/20230306 OSM-3-G444E-GFP; Scarlet-CHE-3_13 amphid/img_000000000_L-561_002.tif]

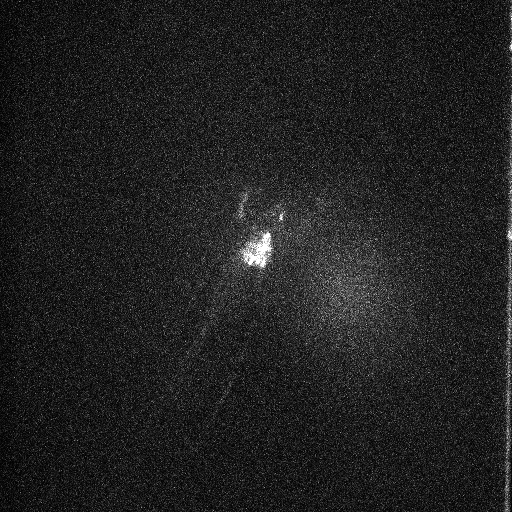

Supplement: Supplementary file 10 — Source data Fig. 1 [file 44318_2024_118_MOESM10_ESM.zip › Figure 1F Micr. image/20230306 OSM-3-G444E-GFP; Scarlet-CHE-3_13 amphid/img_000000000_L-561_000.tif]

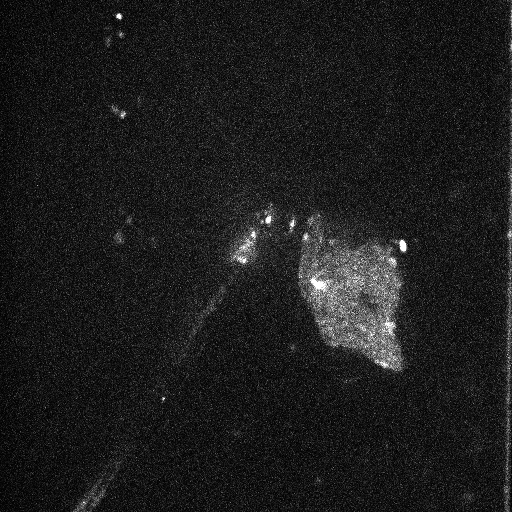

Supplement: Supplementary file 10 — Source data Fig. 1 [file 44318_2024_118_MOESM10_ESM.zip › Figure 1F Micr. image/20230306 OSM-3-G444E-GFP; Scarlet-CHE-3_13 amphid/img_000000000_L-561_014.tif]

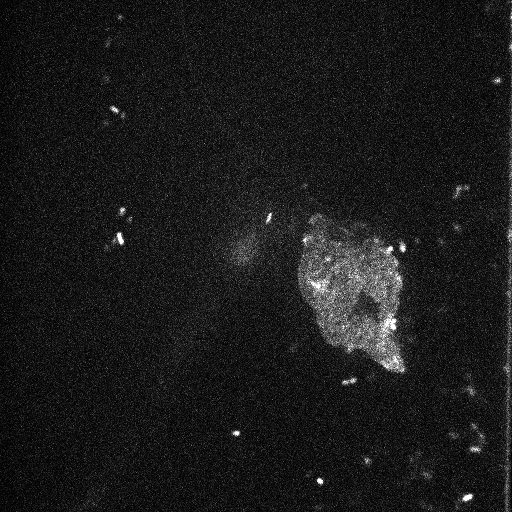

Supplement: Supplementary file 10 — Source data Fig. 1 [file 44318_2024_118_MOESM10_ESM.zip › Figure 1F Micr. image/20230306 OSM-3-G444E-GFP; Scarlet-CHE-3_13 amphid/img_000000000_L-561_015.tif]

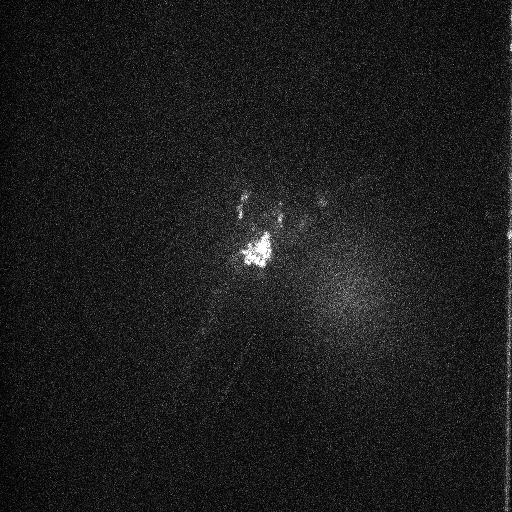

Supplement: Supplementary file 10 — Source data Fig. 1 [file 44318_2024_118_MOESM10_ESM.zip › Figure 1F Micr. image/20230306 OSM-3-G444E-GFP; Scarlet-CHE-3_13 amphid/img_000000000_L-561_001.tif]

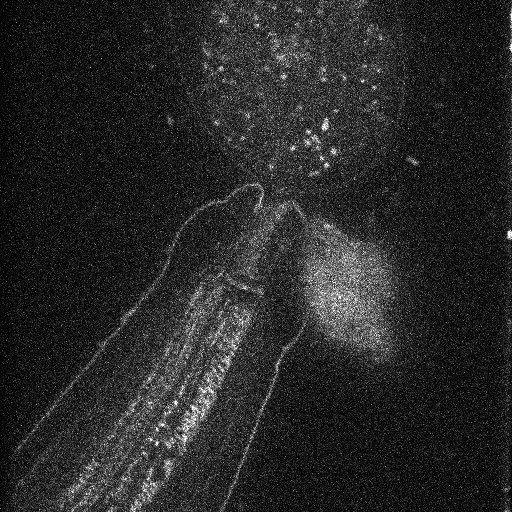

Supplement: Supplementary file 10 — Source data Fig. 1 [file 44318_2024_118_MOESM10_ESM.zip › Figure 1F Micr. image/20230306 OSM-3-G444E-GFP; Scarlet-CHE-3_13 amphid/img_000000000_L-488_009.tif]

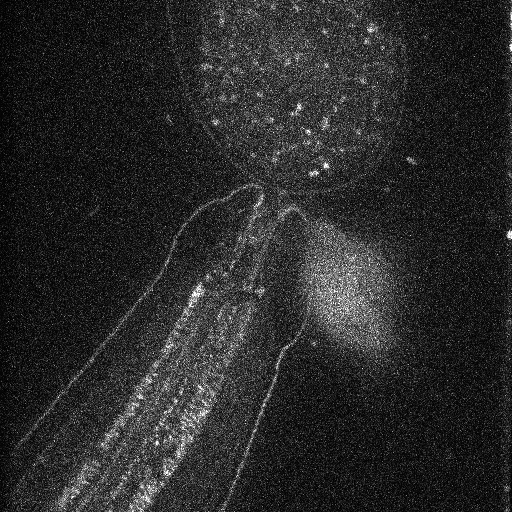

Supplement: Supplementary file 10 — Source data Fig. 1 [file 44318_2024_118_MOESM10_ESM.zip › Figure 1F Micr. image/20230306 OSM-3-G444E-GFP; Scarlet-CHE-3_13 amphid/img_000000000_L-488_008.tif]

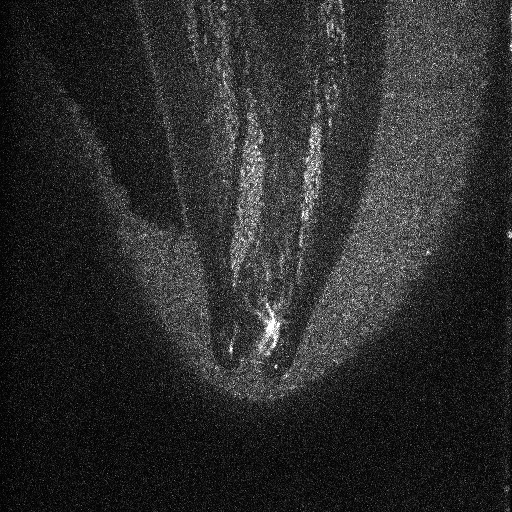

Supplement: Supplementary file 10 — Source data Fig. 1 [file 44318_2024_118_MOESM10_ESM.zip › Figure 1F Micr. image/20230224 Scarlet-che-3; osm-3-gfp_1 amphid/img_000000000_L-488_006.tif]

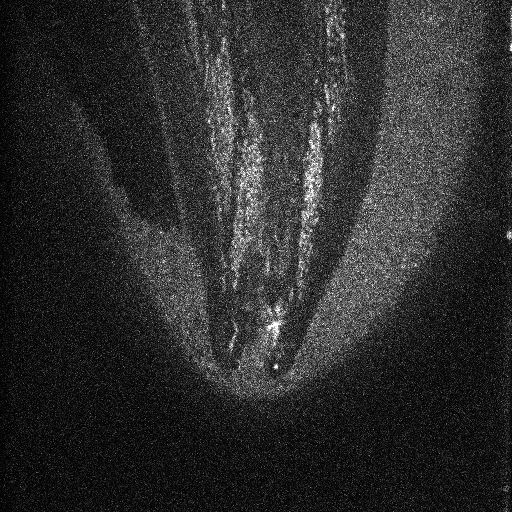

Supplement: Supplementary file 10 — Source data Fig. 1 [file 44318_2024_118_MOESM10_ESM.zip › Figure 1F Micr. image/20230224 Scarlet-che-3; osm-3-gfp_1 amphid/img_000000000_L-488_007.tif]

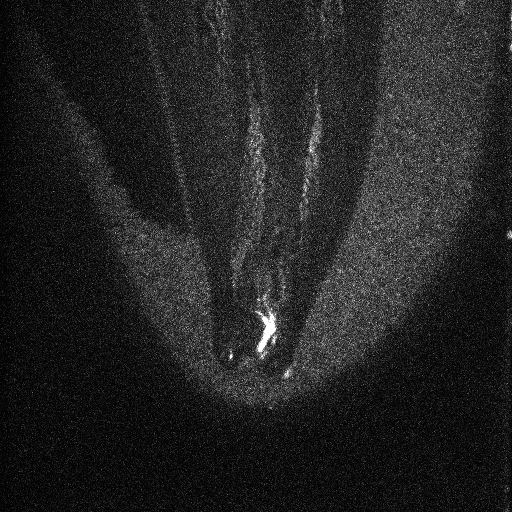

Supplement: Supplementary file 10 — Source data Fig. 1 [file 44318_2024_118_MOESM10_ESM.zip › Figure 1F Micr. image/20230224 Scarlet-che-3; osm-3-gfp_1 amphid/img_000000000_L-488_005.tif]

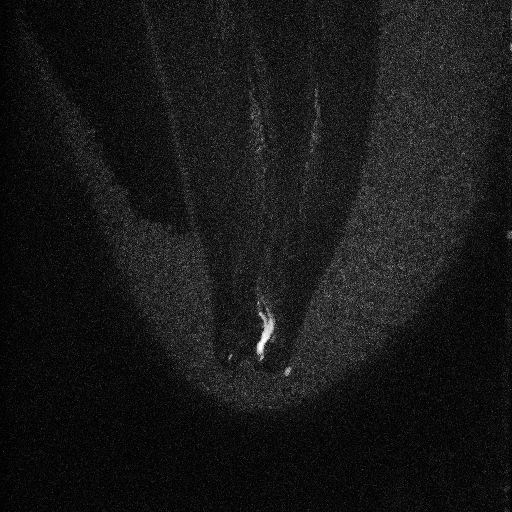

Supplement: Supplementary file 10 — Source data Fig. 1 [file 44318_2024_118_MOESM10_ESM.zip › Figure 1F Micr. image/20230224 Scarlet-che-3; osm-3-gfp_1 amphid/img_000000000_L-488_004.tif]

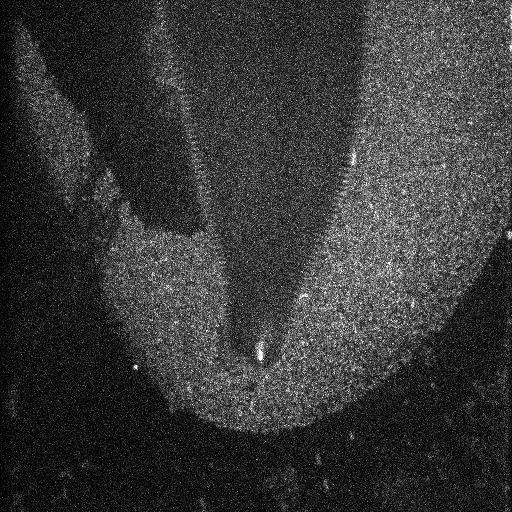

Supplement: Supplementary file 10 — Source data Fig. 1 [file 44318_2024_118_MOESM10_ESM.zip › Figure 1F Micr. image/20230224 Scarlet-che-3; osm-3-gfp_1 amphid/img_000000000_L-488_000.tif]

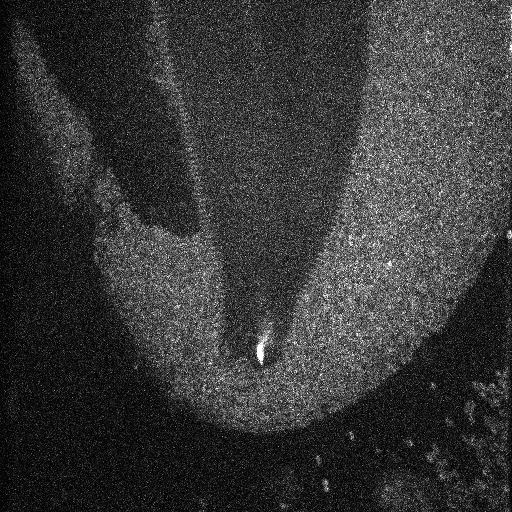

Supplement: Supplementary file 10 — Source data Fig. 1 [file 44318_2024_118_MOESM10_ESM.zip › Figure 1F Micr. image/20230224 Scarlet-che-3; osm-3-gfp_1 amphid/img_000000000_L-488_001.tif]

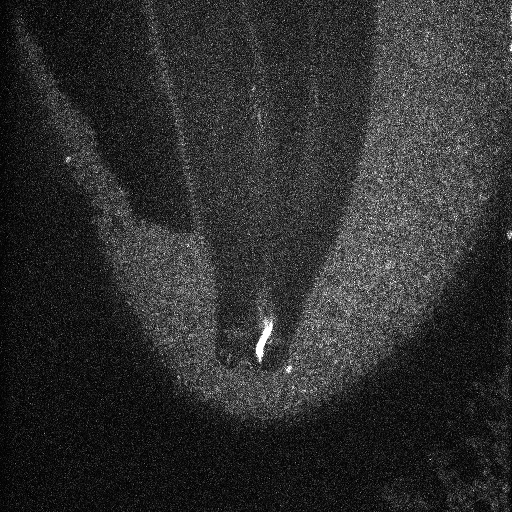

Supplement: Supplementary file 10 — Source data Fig. 1 [file 44318_2024_118_MOESM10_ESM.zip › Figure 1F Micr. image/20230224 Scarlet-che-3; osm-3-gfp_1 amphid/img_000000000_L-488_003.tif]

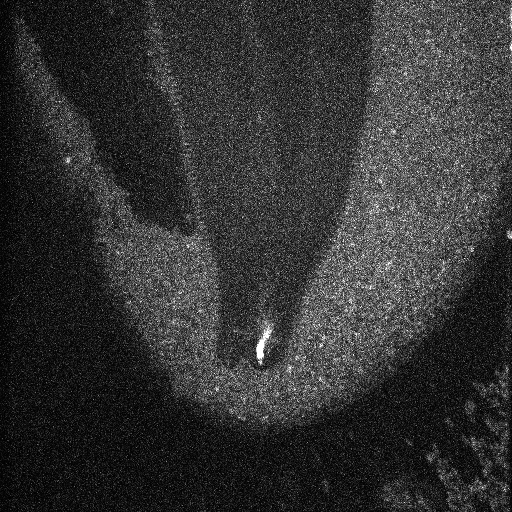

Supplement: Supplementary file 10 — Source data Fig. 1 [file 44318_2024_118_MOESM10_ESM.zip › Figure 1F Micr. image/20230224 Scarlet-che-3; osm-3-gfp_1 amphid/img_000000000_L-488_002.tif]

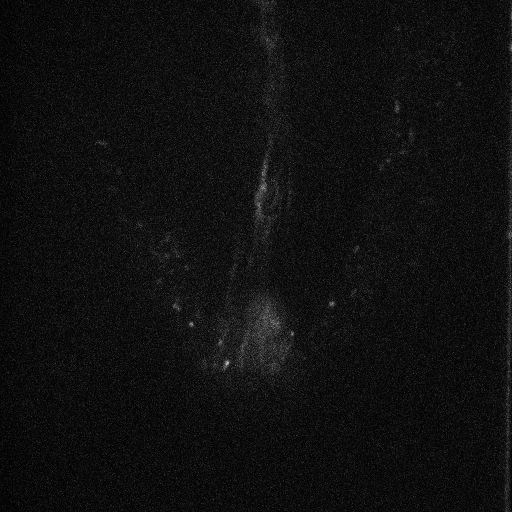

Supplement: Supplementary file 10 — Source data Fig. 1 [file 44318_2024_118_MOESM10_ESM.zip › Figure 1F Micr. image/20230224 Scarlet-che-3; osm-3-gfp_1 amphid/img_000000000_L-561_009.tif]

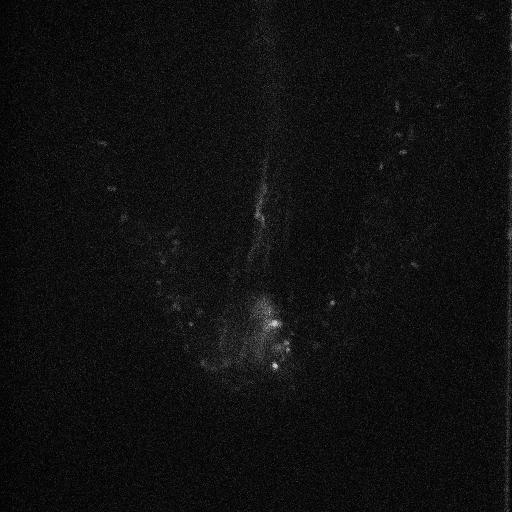

Supplement: Supplementary file 10 — Source data Fig. 1 [file 44318_2024_118_MOESM10_ESM.zip › Figure 1F Micr. image/20230224 Scarlet-che-3; osm-3-gfp_1 amphid/img_000000000_L-561_008.tif]

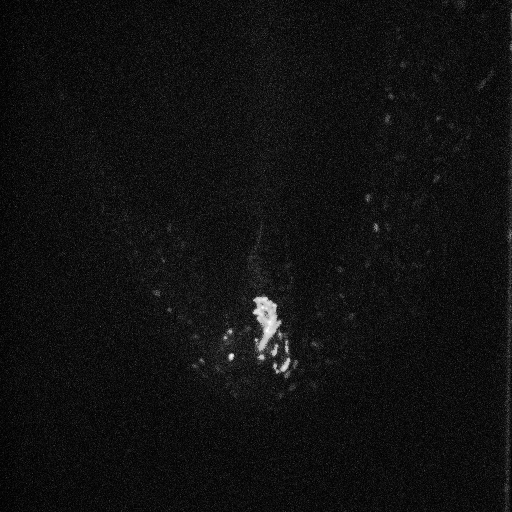

Supplement: Supplementary file 10 — Source data Fig. 1 [file 44318_2024_118_MOESM10_ESM.zip › Figure 1F Micr. image/20230224 Scarlet-che-3; osm-3-gfp_1 amphid/img_000000000_L-561_005.tif]

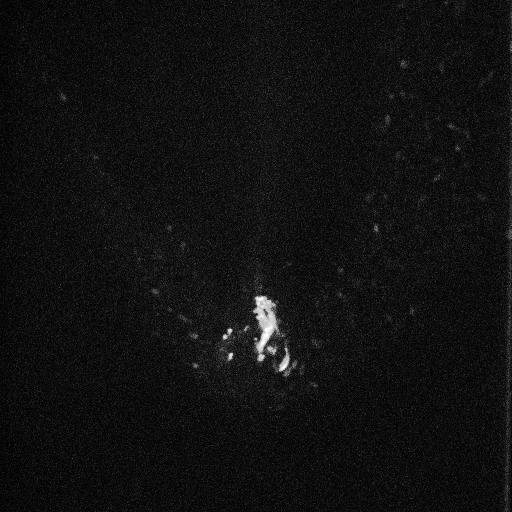

Supplement: Supplementary file 10 — Source data Fig. 1 [file 44318_2024_118_MOESM10_ESM.zip › Figure 1F Micr. image/20230224 Scarlet-che-3; osm-3-gfp_1 amphid/img_000000000_L-561_004.tif]

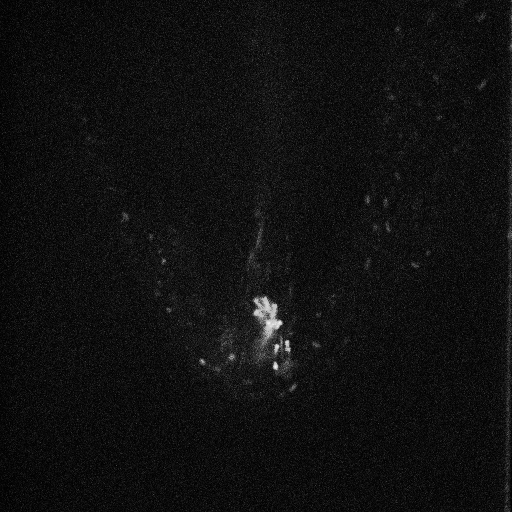

Supplement: Supplementary file 10 — Source data Fig. 1 [file 44318_2024_118_MOESM10_ESM.zip › Figure 1F Micr. image/20230224 Scarlet-che-3; osm-3-gfp_1 amphid/img_000000000_L-561_006.tif]

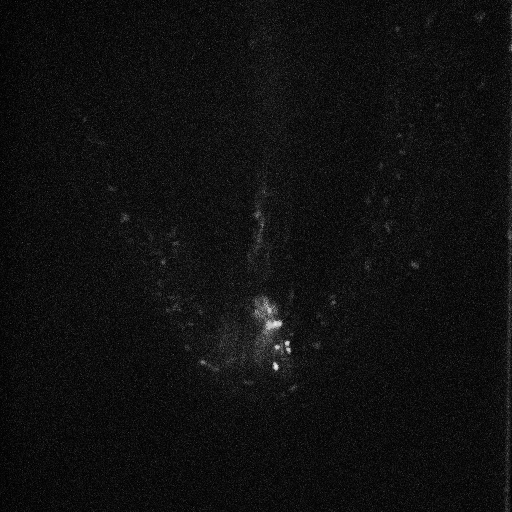

Supplement: Supplementary file 10 — Source data Fig. 1 [file 44318_2024_118_MOESM10_ESM.zip › Figure 1F Micr. image/20230224 Scarlet-che-3; osm-3-gfp_1 amphid/img_000000000_L-561_007.tif]

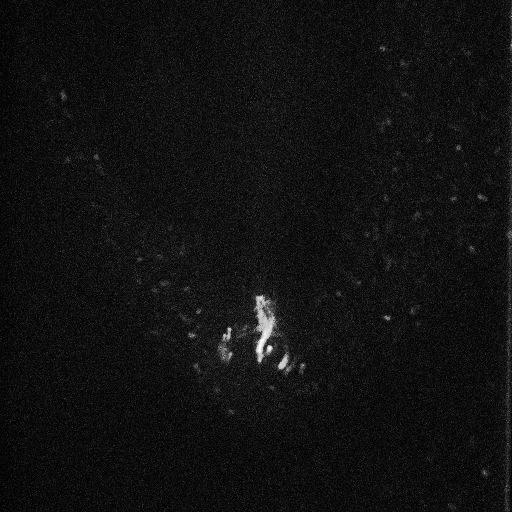

Supplement: Supplementary file 10 — Source data Fig. 1 [file 44318_2024_118_MOESM10_ESM.zip › Figure 1F Micr. image/20230224 Scarlet-che-3; osm-3-gfp_1 amphid/img_000000000_L-561_003.tif]

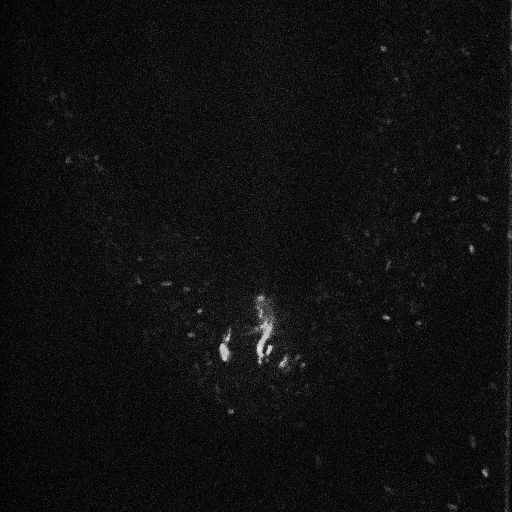

Supplement: Supplementary file 10 — Source data Fig. 1 [file 44318_2024_118_MOESM10_ESM.zip › Figure 1F Micr. image/20230224 Scarlet-che-3; osm-3-gfp_1 amphid/img_000000000_L-561_002.tif]

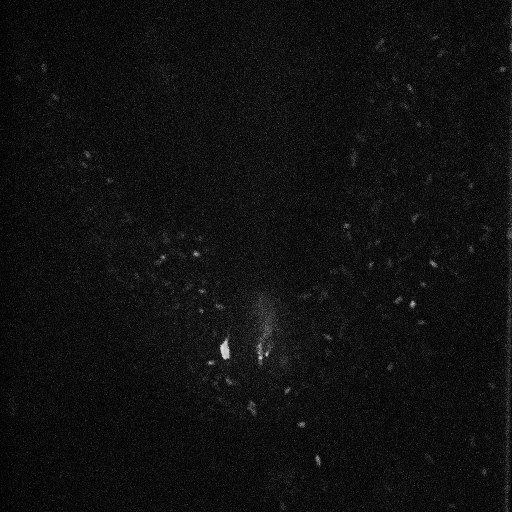

Supplement: Supplementary file 10 — Source data Fig. 1 [file 44318_2024_118_MOESM10_ESM.zip › Figure 1F Micr. image/20230224 Scarlet-che-3; osm-3-gfp_1 amphid/img_000000000_L-561_000.tif]

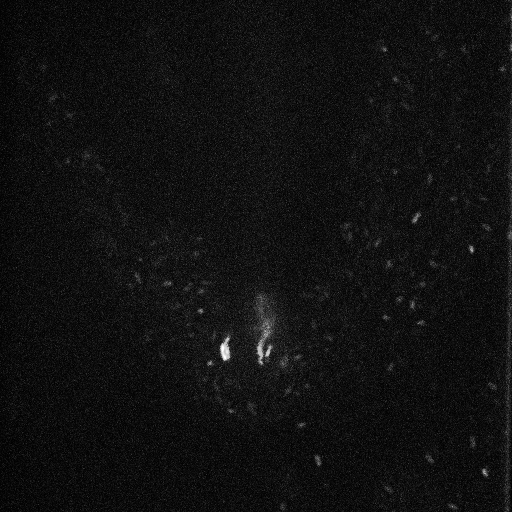

Supplement: Supplementary file 10 — Source data Fig. 1 [file 44318_2024_118_MOESM10_ESM.zip › Figure 1F Micr. image/20230224 Scarlet-che-3; osm-3-gfp_1 amphid/img_000000000_L-561_001.tif]

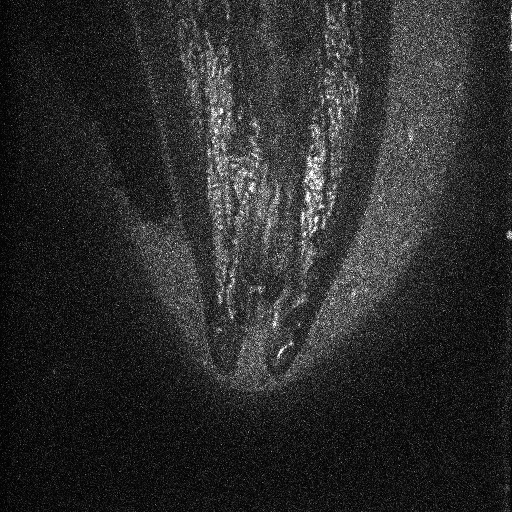

Supplement: Supplementary file 10 — Source data Fig. 1 [file 44318_2024_118_MOESM10_ESM.zip › Figure 1F Micr. image/20230224 Scarlet-che-3; osm-3-gfp_1 amphid/img_000000000_L-488_009.tif]

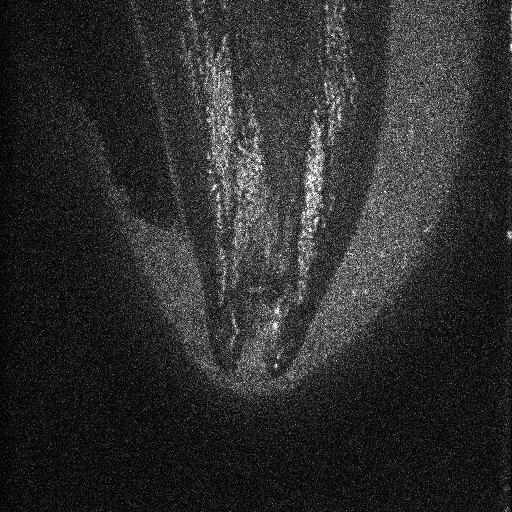

Supplement: Supplementary file 10 — Source data Fig. 1 [file 44318_2024_118_MOESM10_ESM.zip › Figure 1F Micr. image/20230224 Scarlet-che-3; osm-3-gfp_1 amphid/img_000000000_L-488_008.tif]

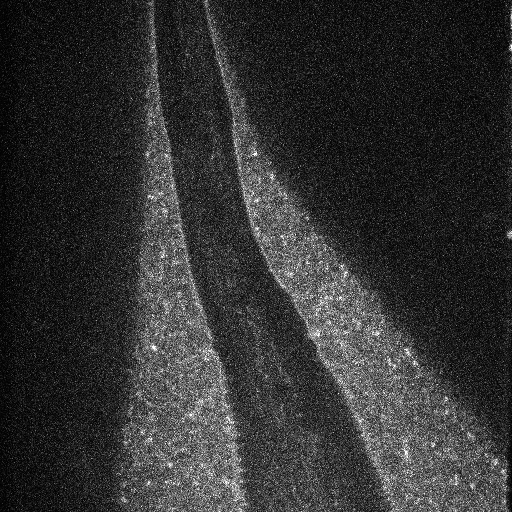

Supplement: Supplementary file 10 — Source data Fig. 1 [file 44318_2024_118_MOESM10_ESM.zip › Figure 1F Micr. image/20230324 Scarlet-che-3; osm-3-g444e-gfp_2 phasmid/img_000000000_L-488_006.tif]

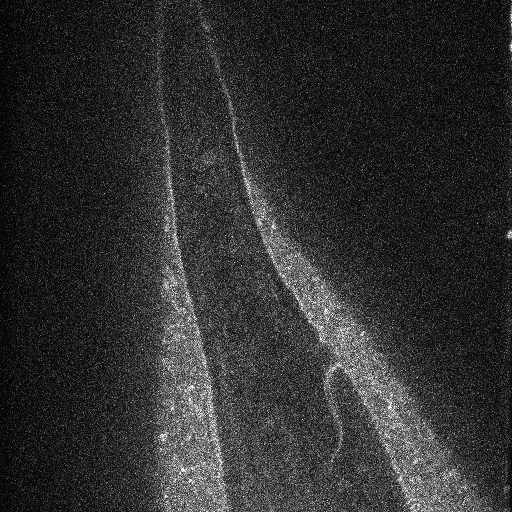

Supplement: Supplementary file 10 — Source data Fig. 1 [file 44318_2024_118_MOESM10_ESM.zip › Figure 1F Micr. image/20230324 Scarlet-che-3; osm-3-g444e-gfp_2 phasmid/img_000000000_L-488_012.tif]

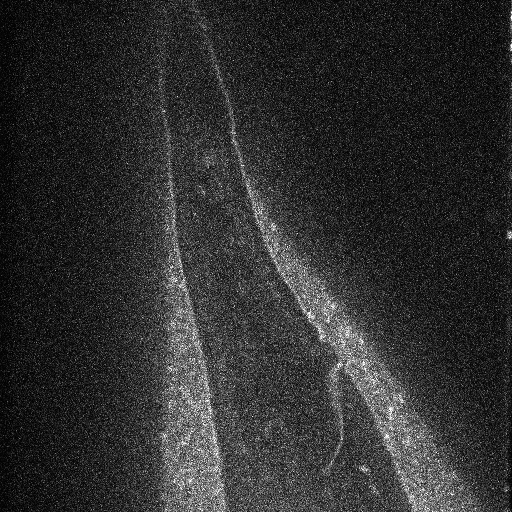

Supplement: Supplementary file 10 — Source data Fig. 1 [file 44318_2024_118_MOESM10_ESM.zip › Figure 1F Micr. image/20230324 Scarlet-che-3; osm-3-g444e-gfp_2 phasmid/img_000000000_L-488_013.tif]

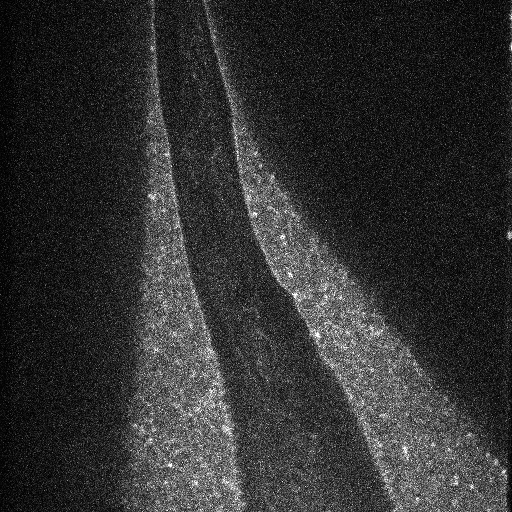

Supplement: Supplementary file 10 — Source data Fig. 1 [file 44318_2024_118_MOESM10_ESM.zip › Figure 1F Micr. image/20230324 Scarlet-che-3; osm-3-g444e-gfp_2 phasmid/img_000000000_L-488_007.tif]

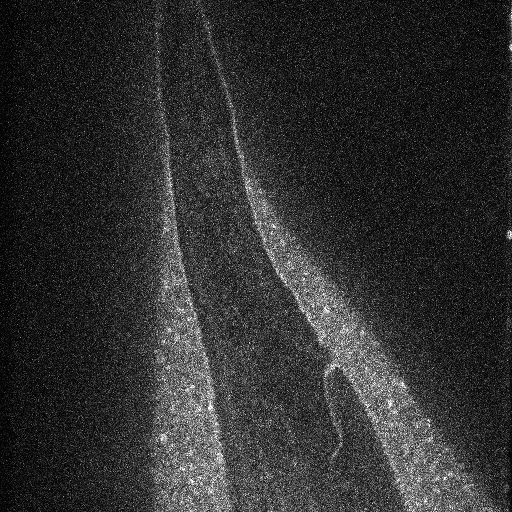

Supplement: Supplementary file 10 — Source data Fig. 1 [file 44318_2024_118_MOESM10_ESM.zip › Figure 1F Micr. image/20230324 Scarlet-che-3; osm-3-g444e-gfp_2 phasmid/img_000000000_L-488_011.tif]

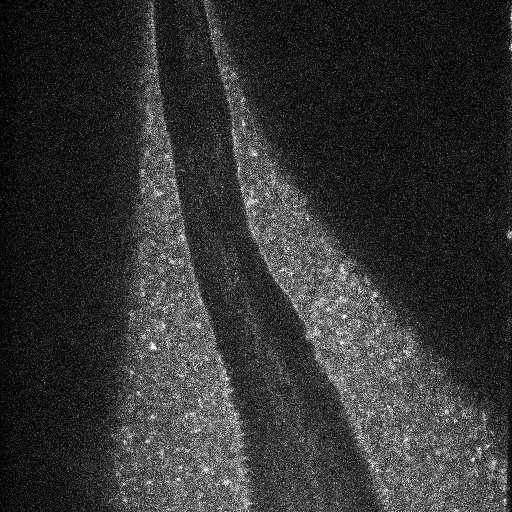

Supplement: Supplementary file 10 — Source data Fig. 1 [file 44318_2024_118_MOESM10_ESM.zip › Figure 1F Micr. image/20230324 Scarlet-che-3; osm-3-g444e-gfp_2 phasmid/img_000000000_L-488_005.tif]

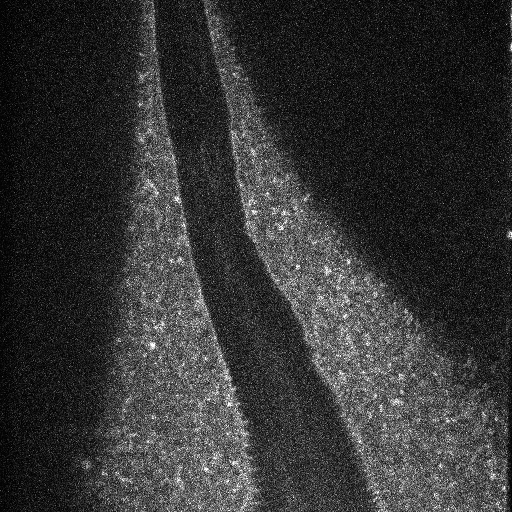

Supplement: Supplementary file 10 — Source data Fig. 1 [file 44318_2024_118_MOESM10_ESM.zip › Figure 1F Micr. image/20230324 Scarlet-che-3; osm-3-g444e-gfp_2 phasmid/img_000000000_L-488_004.tif]

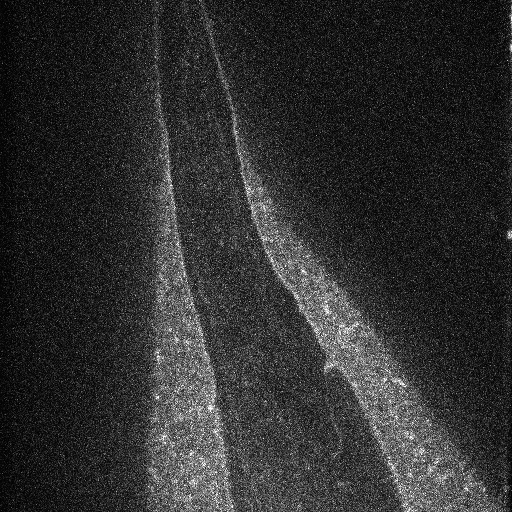

Supplement: Supplementary file 10 — Source data Fig. 1 [file 44318_2024_118_MOESM10_ESM.zip › Figure 1F Micr. image/20230324 Scarlet-che-3; osm-3-g444e-gfp_2 phasmid/img_000000000_L-488_010.tif]

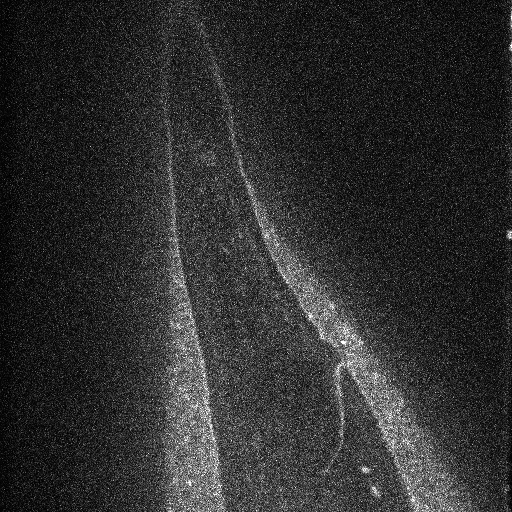

Supplement: Supplementary file 10 — Source data Fig. 1 [file 44318_2024_118_MOESM10_ESM.zip › Figure 1F Micr. image/20230324 Scarlet-che-3; osm-3-g444e-gfp_2 phasmid/img_000000000_L-488_014.tif]

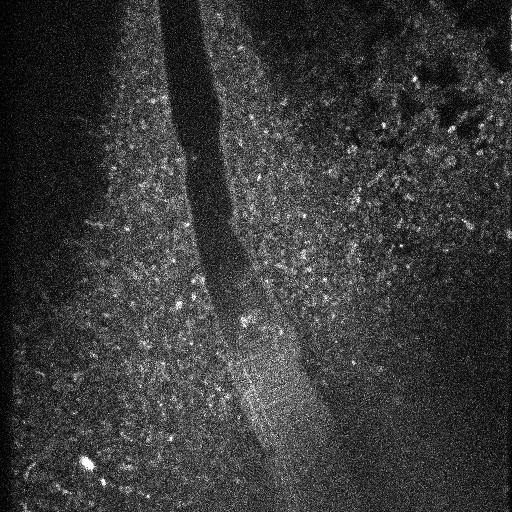

Supplement: Supplementary file 10 — Source data Fig. 1 [file 44318_2024_118_MOESM10_ESM.zip › Figure 1F Micr. image/20230324 Scarlet-che-3; osm-3-g444e-gfp_2 phasmid/img_000000000_L-488_000.tif]

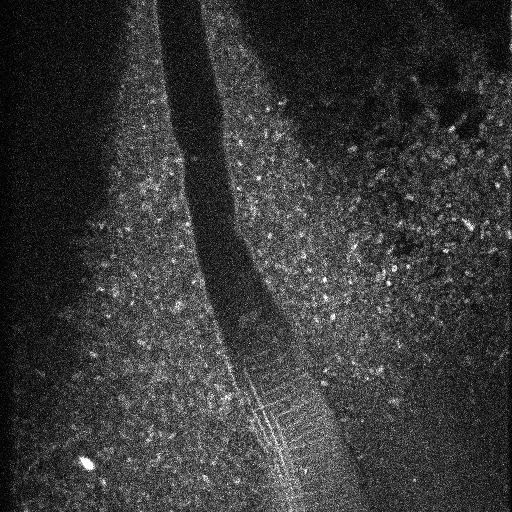

Supplement: Supplementary file 10 — Source data Fig. 1 [file 44318_2024_118_MOESM10_ESM.zip › Figure 1F Micr. image/20230324 Scarlet-che-3; osm-3-g444e-gfp_2 phasmid/img_000000000_L-488_001.tif]
