# Supplementary figures and images for: Neurons dispose of hyperactive kinesin into glial cells for clearance (part 4 of 9)
Source: EMBO J. 2024 May 28;43(13):5. doi: 10.1038/s44318-024-00118-0 (PMC11217292; doi:10.1038/s44318-024-00118-0)

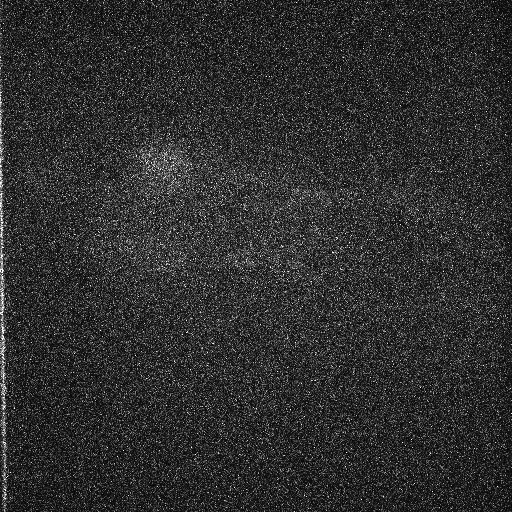

Supplement: Supplementary file 11 — Source data Fig. 2 [file 44318_2024_118_MOESM11_ESM.zip › Figure2/Figure 2A Micr. image/20201128 osm-3 G444E-gfp; HIS-54-BFP_3/Pos0/img_000000000_Confocal-561_018.tif]

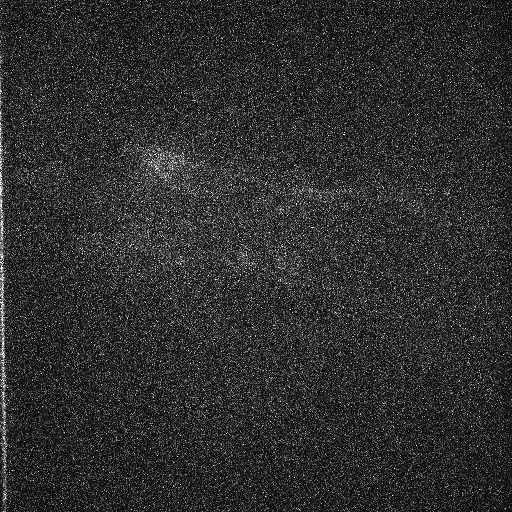

Supplement: Supplementary file 11 — Source data Fig. 2 [file 44318_2024_118_MOESM11_ESM.zip › Figure2/Figure 2A Micr. image/20201128 osm-3 G444E-gfp; HIS-54-BFP_3/Pos0/img_000000000_Confocal-561_019.tif]

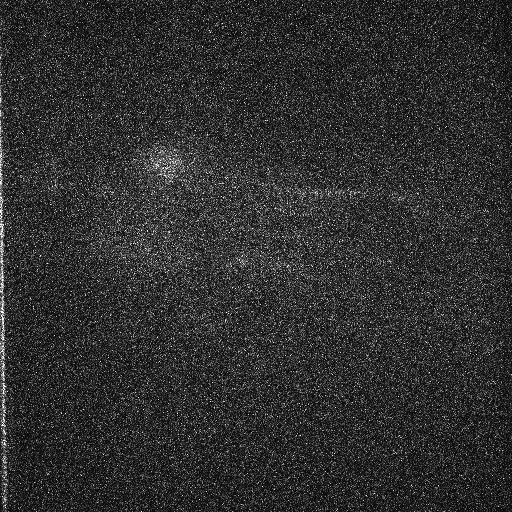

Supplement: Supplementary file 11 — Source data Fig. 2 [file 44318_2024_118_MOESM11_ESM.zip › Figure2/Figure 2A Micr. image/20201128 osm-3 G444E-gfp; HIS-54-BFP_3/Pos0/img_000000000_Confocal-561_020.tif]

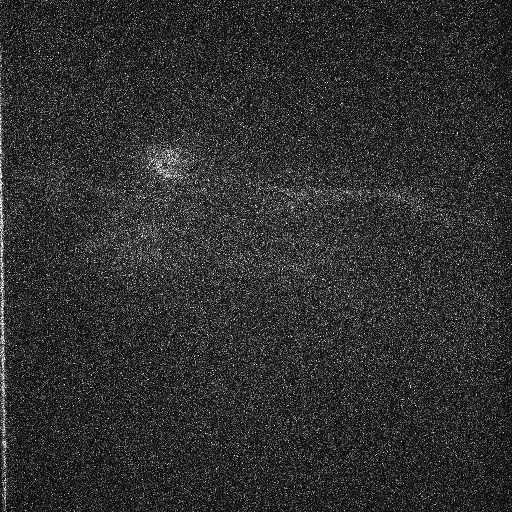

Supplement: Supplementary file 11 — Source data Fig. 2 [file 44318_2024_118_MOESM11_ESM.zip › Figure2/Figure 2A Micr. image/20201128 osm-3 G444E-gfp; HIS-54-BFP_3/Pos0/img_000000000_Confocal-561_021.tif]

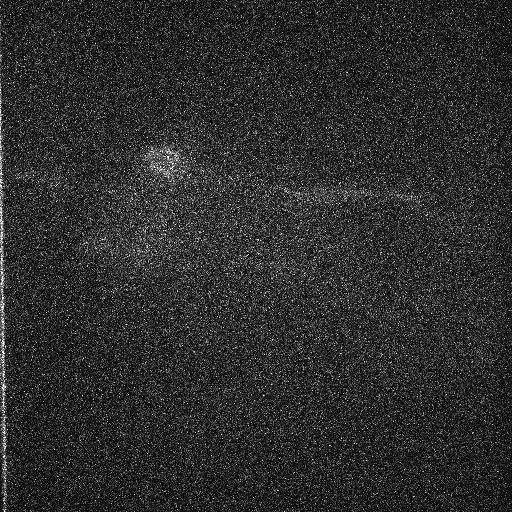

Supplement: Supplementary file 11 — Source data Fig. 2 [file 44318_2024_118_MOESM11_ESM.zip › Figure2/Figure 2A Micr. image/20201128 osm-3 G444E-gfp; HIS-54-BFP_3/Pos0/img_000000000_Confocal-561_022.tif]

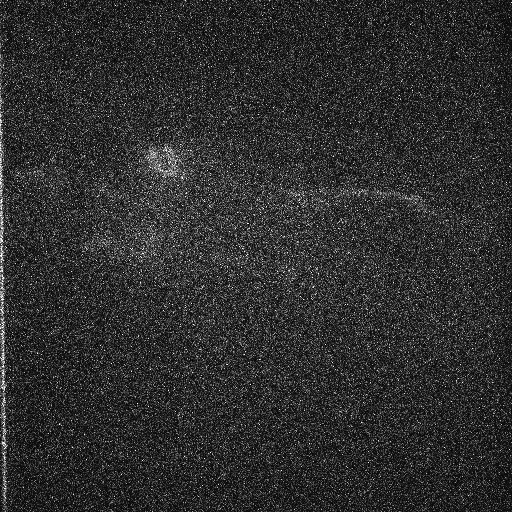

Supplement: Supplementary file 11 — Source data Fig. 2 [file 44318_2024_118_MOESM11_ESM.zip › Figure2/Figure 2A Micr. image/20201128 osm-3 G444E-gfp; HIS-54-BFP_3/Pos0/img_000000000_Confocal-561_023.tif]

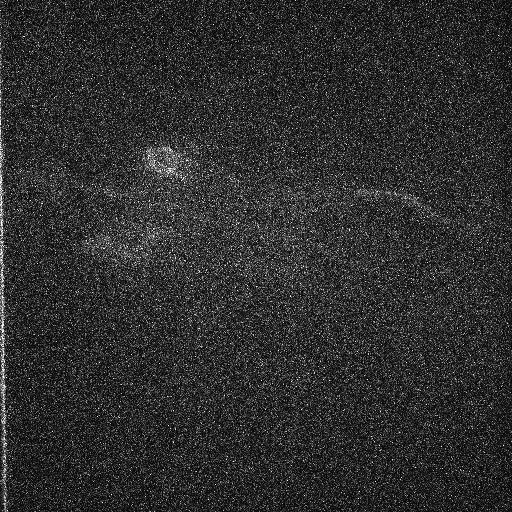

Supplement: Supplementary file 11 — Source data Fig. 2 [file 44318_2024_118_MOESM11_ESM.zip › Figure2/Figure 2A Micr. image/20201128 osm-3 G444E-gfp; HIS-54-BFP_3/Pos0/img_000000000_Confocal-561_024.tif]

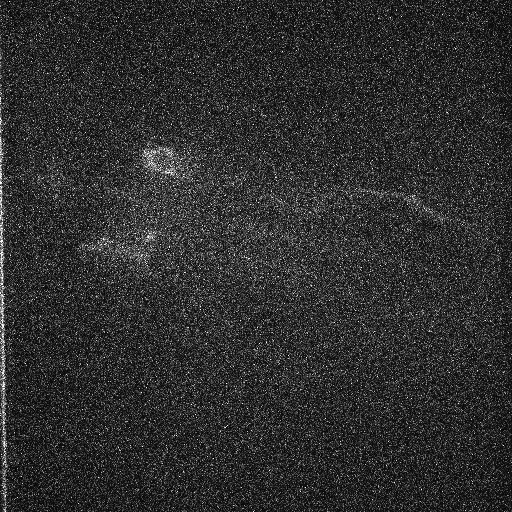

Supplement: Supplementary file 11 — Source data Fig. 2 [file 44318_2024_118_MOESM11_ESM.zip › Figure2/Figure 2A Micr. image/20201128 osm-3 G444E-gfp; HIS-54-BFP_3/Pos0/img_000000000_Confocal-561_025.tif]

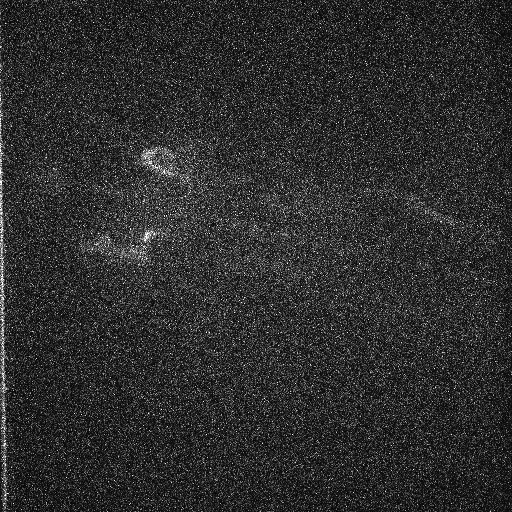

Supplement: Supplementary file 11 — Source data Fig. 2 [file 44318_2024_118_MOESM11_ESM.zip › Figure2/Figure 2A Micr. image/20201128 osm-3 G444E-gfp; HIS-54-BFP_3/Pos0/img_000000000_Confocal-561_026.tif]

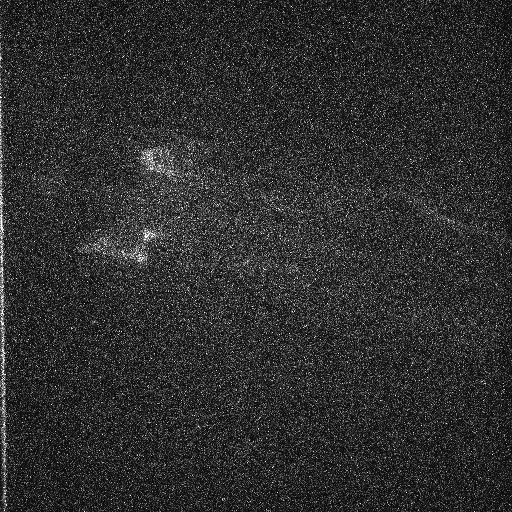

Supplement: Supplementary file 11 — Source data Fig. 2 [file 44318_2024_118_MOESM11_ESM.zip › Figure2/Figure 2A Micr. image/20201128 osm-3 G444E-gfp; HIS-54-BFP_3/Pos0/img_000000000_Confocal-561_027.tif]

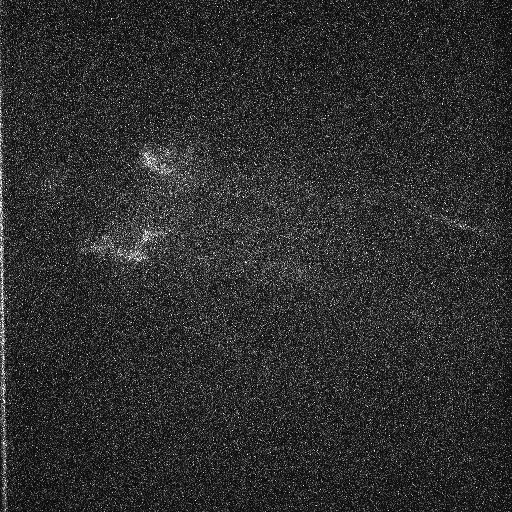

Supplement: Supplementary file 11 — Source data Fig. 2 [file 44318_2024_118_MOESM11_ESM.zip › Figure2/Figure 2A Micr. image/20201128 osm-3 G444E-gfp; HIS-54-BFP_3/Pos0/img_000000000_Confocal-561_028.tif]

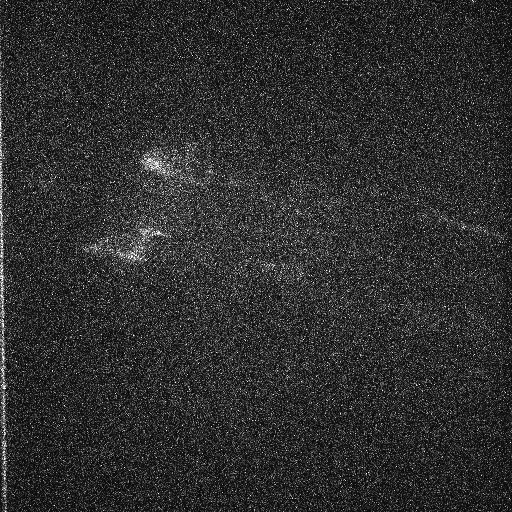

Supplement: Supplementary file 11 — Source data Fig. 2 [file 44318_2024_118_MOESM11_ESM.zip › Figure2/Figure 2A Micr. image/20201128 osm-3 G444E-gfp; HIS-54-BFP_3/Pos0/img_000000000_Confocal-561_029.tif]

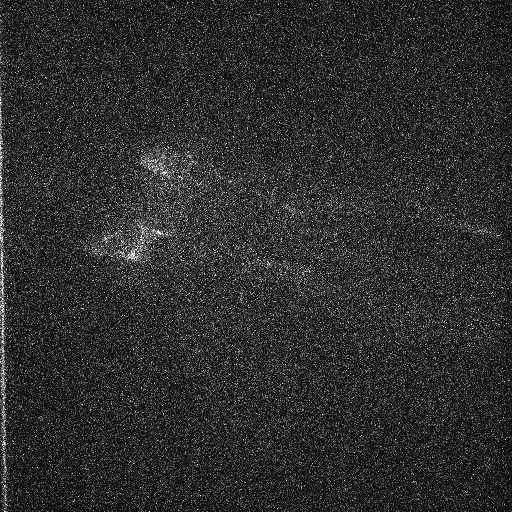

Supplement: Supplementary file 11 — Source data Fig. 2 [file 44318_2024_118_MOESM11_ESM.zip › Figure2/Figure 2A Micr. image/20201128 osm-3 G444E-gfp; HIS-54-BFP_3/Pos0/img_000000000_Confocal-561_030.tif]

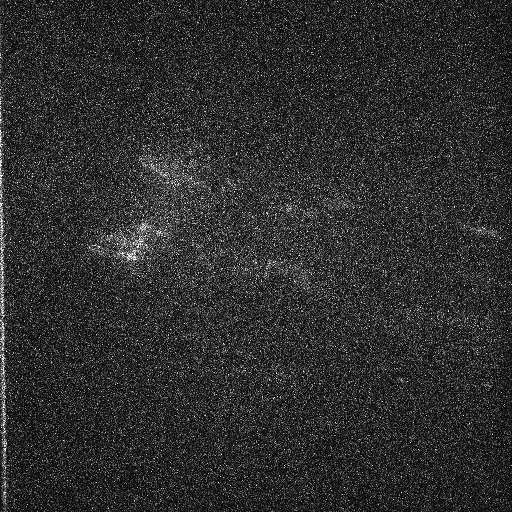

Supplement: Supplementary file 11 — Source data Fig. 2 [file 44318_2024_118_MOESM11_ESM.zip › Figure2/Figure 2A Micr. image/20201128 osm-3 G444E-gfp; HIS-54-BFP_3/Pos0/img_000000000_Confocal-561_031.tif]

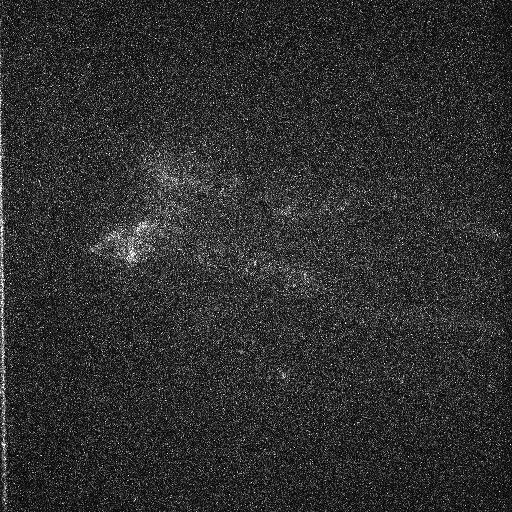

Supplement: Supplementary file 11 — Source data Fig. 2 [file 44318_2024_118_MOESM11_ESM.zip › Figure2/Figure 2A Micr. image/20201128 osm-3 G444E-gfp; HIS-54-BFP_3/Pos0/img_000000000_Confocal-561_032.tif]

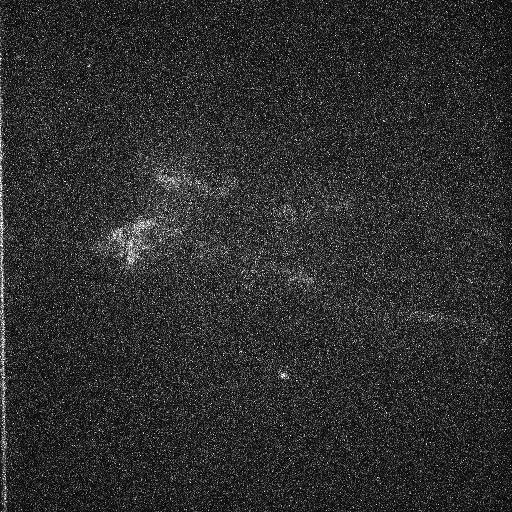

Supplement: Supplementary file 11 — Source data Fig. 2 [file 44318_2024_118_MOESM11_ESM.zip › Figure2/Figure 2A Micr. image/20201128 osm-3 G444E-gfp; HIS-54-BFP_3/Pos0/img_000000000_Confocal-561_033.tif]

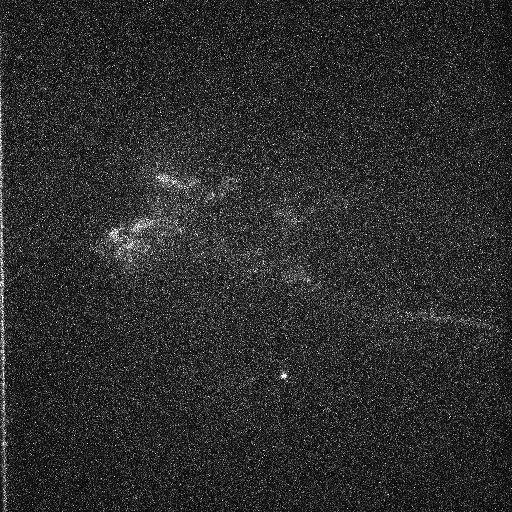

Supplement: Supplementary file 11 — Source data Fig. 2 [file 44318_2024_118_MOESM11_ESM.zip › Figure2/Figure 2A Micr. image/20201128 osm-3 G444E-gfp; HIS-54-BFP_3/Pos0/img_000000000_Confocal-561_034.tif]

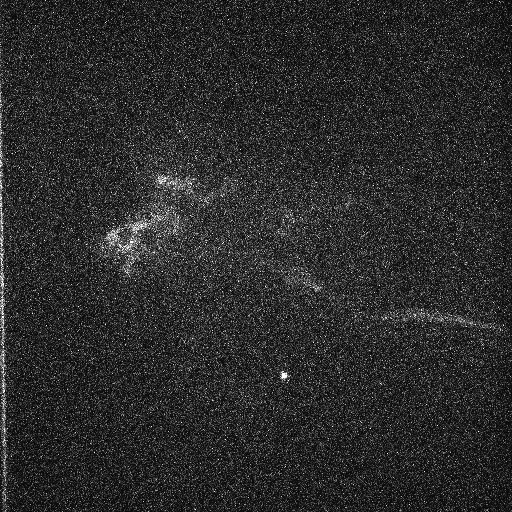

Supplement: Supplementary file 11 — Source data Fig. 2 [file 44318_2024_118_MOESM11_ESM.zip › Figure2/Figure 2A Micr. image/20201128 osm-3 G444E-gfp; HIS-54-BFP_3/Pos0/img_000000000_Confocal-561_035.tif]

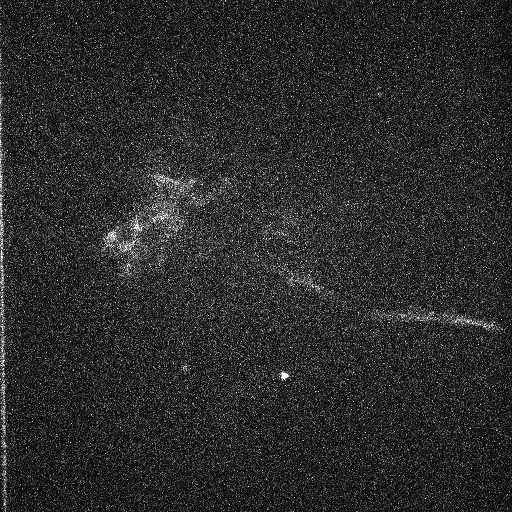

Supplement: Supplementary file 11 — Source data Fig. 2 [file 44318_2024_118_MOESM11_ESM.zip › Figure2/Figure 2A Micr. image/20201128 osm-3 G444E-gfp; HIS-54-BFP_3/Pos0/img_000000000_Confocal-561_036.tif]

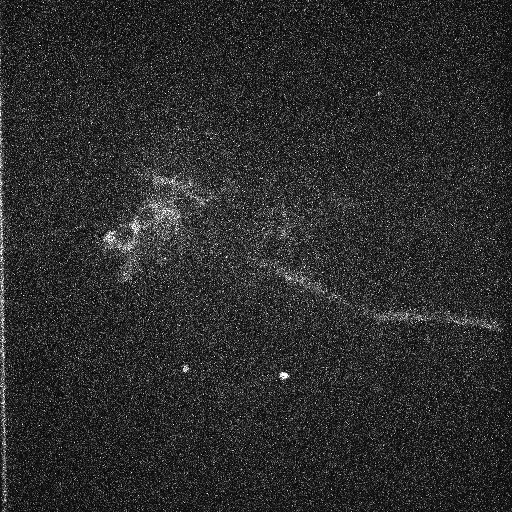

Supplement: Supplementary file 11 — Source data Fig. 2 [file 44318_2024_118_MOESM11_ESM.zip › Figure2/Figure 2A Micr. image/20201128 osm-3 G444E-gfp; HIS-54-BFP_3/Pos0/img_000000000_Confocal-561_037.tif]

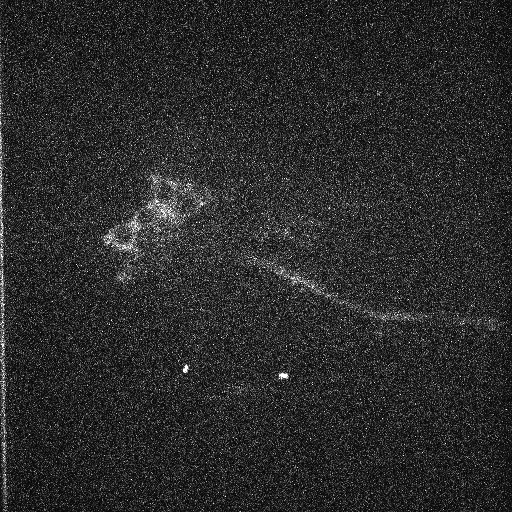

Supplement: Supplementary file 11 — Source data Fig. 2 [file 44318_2024_118_MOESM11_ESM.zip › Figure2/Figure 2A Micr. image/20201128 osm-3 G444E-gfp; HIS-54-BFP_3/Pos0/img_000000000_Confocal-561_038.tif]

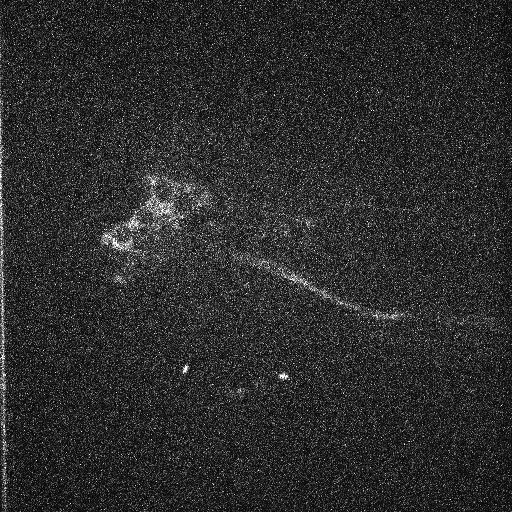

Supplement: Supplementary file 11 — Source data Fig. 2 [file 44318_2024_118_MOESM11_ESM.zip › Figure2/Figure 2A Micr. image/20201128 osm-3 G444E-gfp; HIS-54-BFP_3/Pos0/img_000000000_Confocal-561_039.tif]

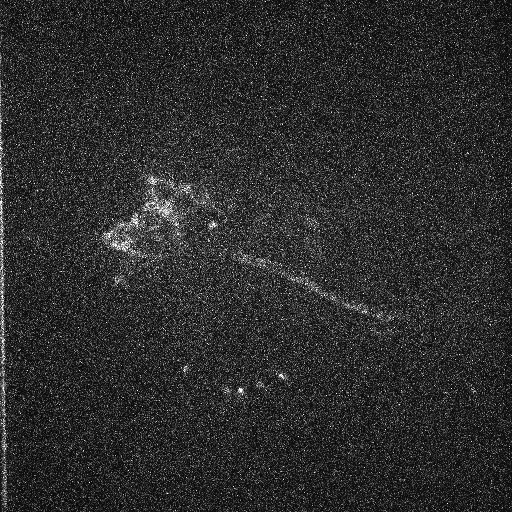

Supplement: Supplementary file 11 — Source data Fig. 2 [file 44318_2024_118_MOESM11_ESM.zip › Figure2/Figure 2A Micr. image/20201128 osm-3 G444E-gfp; HIS-54-BFP_3/Pos0/img_000000000_Confocal-561_040.tif]

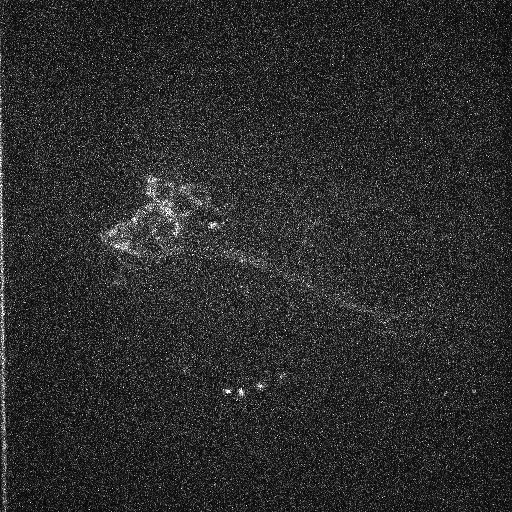

Supplement: Supplementary file 11 — Source data Fig. 2 [file 44318_2024_118_MOESM11_ESM.zip › Figure2/Figure 2A Micr. image/20201128 osm-3 G444E-gfp; HIS-54-BFP_3/Pos0/img_000000000_Confocal-561_041.tif]

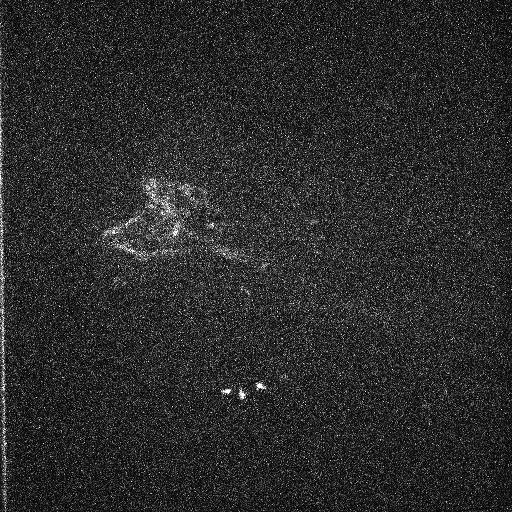

Supplement: Supplementary file 11 — Source data Fig. 2 [file 44318_2024_118_MOESM11_ESM.zip › Figure2/Figure 2A Micr. image/20201128 osm-3 G444E-gfp; HIS-54-BFP_3/Pos0/img_000000000_Confocal-561_042.tif]

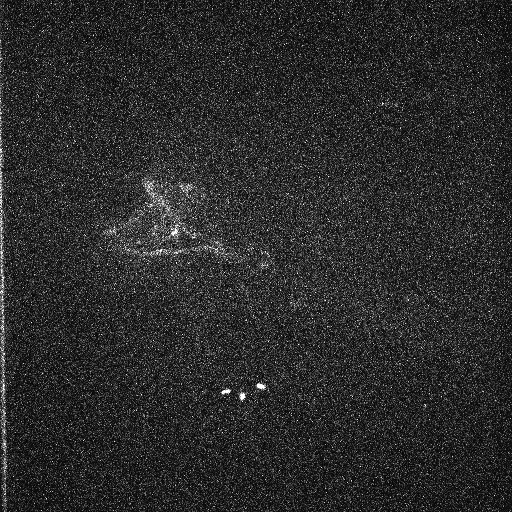

Supplement: Supplementary file 11 — Source data Fig. 2 [file 44318_2024_118_MOESM11_ESM.zip › Figure2/Figure 2A Micr. image/20201128 osm-3 G444E-gfp; HIS-54-BFP_3/Pos0/img_000000000_Confocal-561_043.tif]

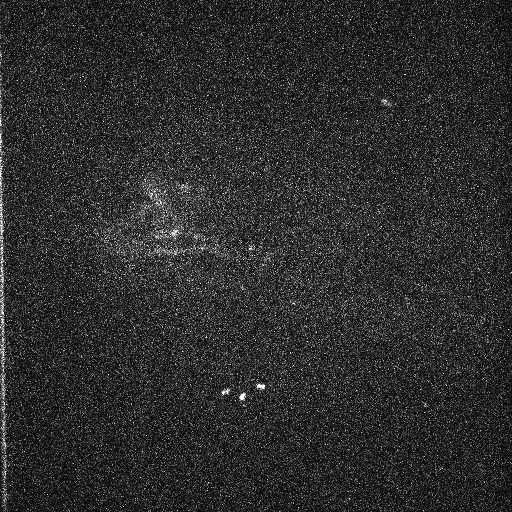

Supplement: Supplementary file 11 — Source data Fig. 2 [file 44318_2024_118_MOESM11_ESM.zip › Figure2/Figure 2A Micr. image/20201128 osm-3 G444E-gfp; HIS-54-BFP_3/Pos0/img_000000000_Confocal-561_044.tif]

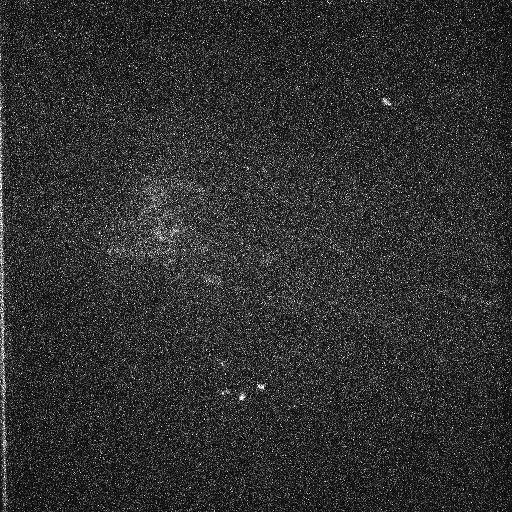

Supplement: Supplementary file 11 — Source data Fig. 2 [file 44318_2024_118_MOESM11_ESM.zip › Figure2/Figure 2A Micr. image/20201128 osm-3 G444E-gfp; HIS-54-BFP_3/Pos0/img_000000000_Confocal-561_045.tif]

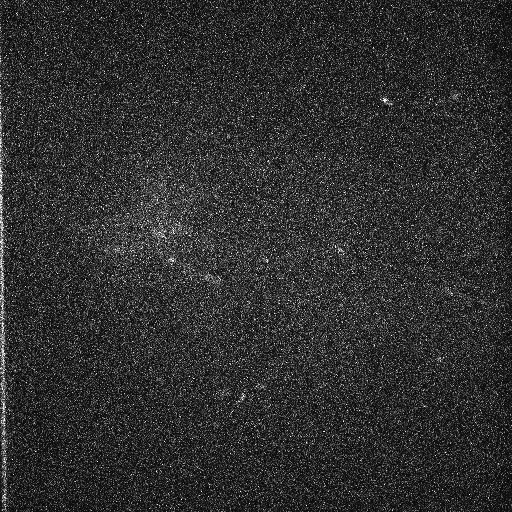

Supplement: Supplementary file 11 — Source data Fig. 2 [file 44318_2024_118_MOESM11_ESM.zip › Figure2/Figure 2A Micr. image/20201128 osm-3 G444E-gfp; HIS-54-BFP_3/Pos0/img_000000000_Confocal-561_046.tif]

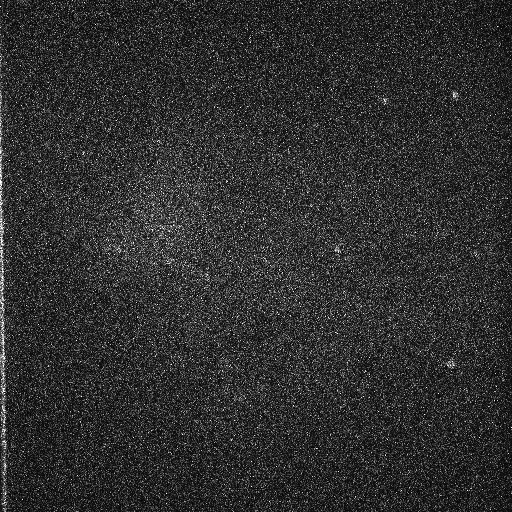

Supplement: Supplementary file 11 — Source data Fig. 2 [file 44318_2024_118_MOESM11_ESM.zip › Figure2/Figure 2A Micr. image/20201128 osm-3 G444E-gfp; HIS-54-BFP_3/Pos0/img_000000000_Confocal-561_047.tif]

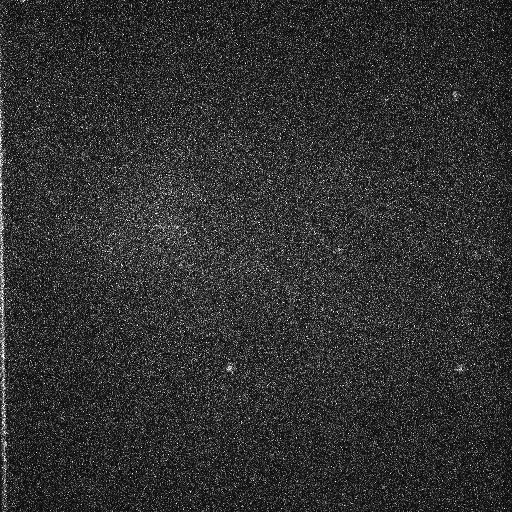

Supplement: Supplementary file 11 — Source data Fig. 2 [file 44318_2024_118_MOESM11_ESM.zip › Figure2/Figure 2A Micr. image/20201128 osm-3 G444E-gfp; HIS-54-BFP_3/Pos0/img_000000000_Confocal-561_048.tif]

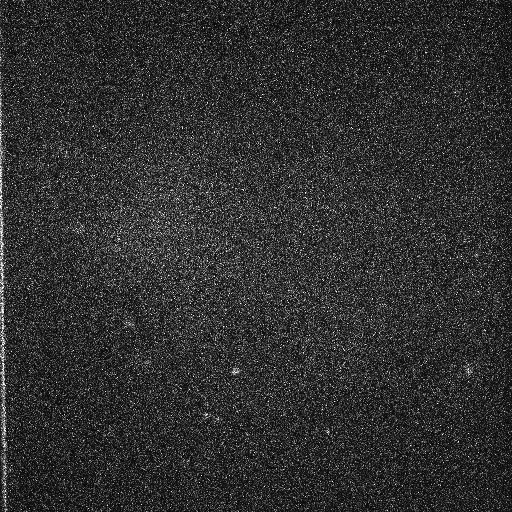

Supplement: Supplementary file 11 — Source data Fig. 2 [file 44318_2024_118_MOESM11_ESM.zip › Figure2/Figure 2A Micr. image/20201128 osm-3 G444E-gfp; HIS-54-BFP_3/Pos0/img_000000000_Confocal-561_049.tif]

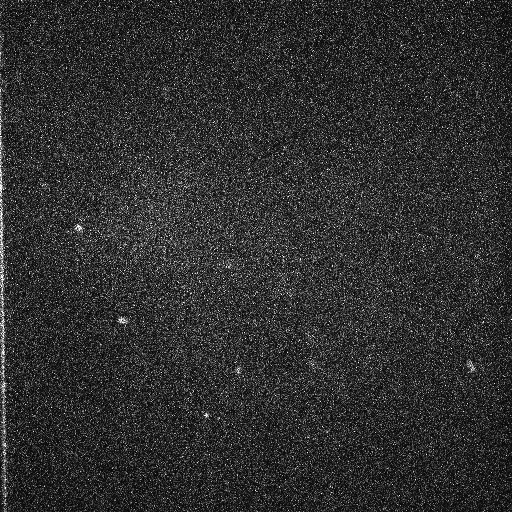

Supplement: Supplementary file 11 — Source data Fig. 2 [file 44318_2024_118_MOESM11_ESM.zip › Figure2/Figure 2A Micr. image/20201128 osm-3 G444E-gfp; HIS-54-BFP_3/Pos0/img_000000000_Confocal-561_050.tif]

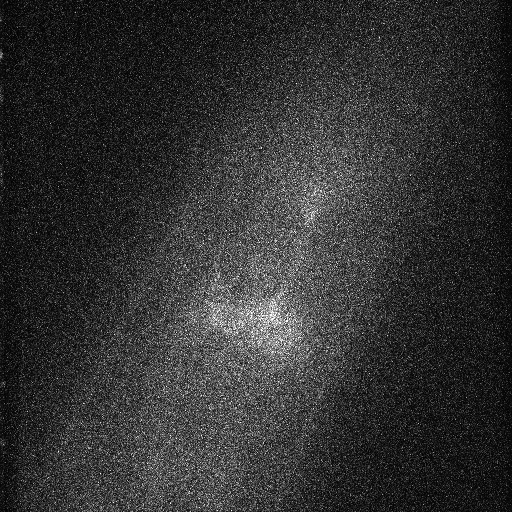

Supplement: Supplementary file 11 — Source data Fig. 2 [file 44318_2024_118_MOESM11_ESM.zip › Figure2/Figure 2A Micr. image/20211124 OSM-3-GFP KI_8/Pos0/img_000000000_Confocal-488-Acq_000.tif]

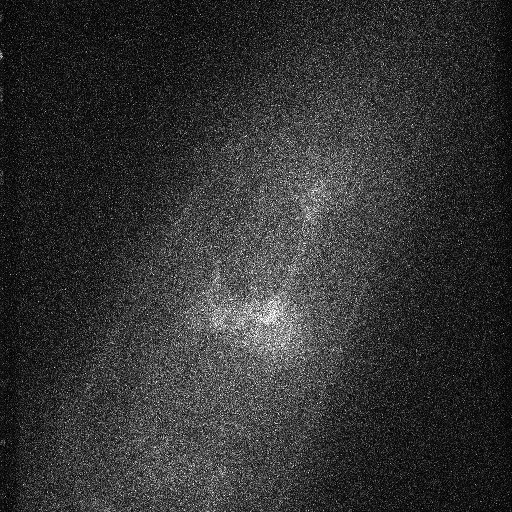

Supplement: Supplementary file 11 — Source data Fig. 2 [file 44318_2024_118_MOESM11_ESM.zip › Figure2/Figure 2A Micr. image/20211124 OSM-3-GFP KI_8/Pos0/img_000000000_Confocal-488-Acq_001.tif]

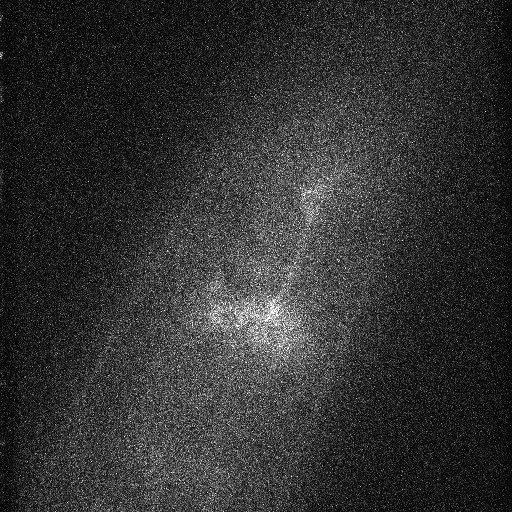

Supplement: Supplementary file 11 — Source data Fig. 2 [file 44318_2024_118_MOESM11_ESM.zip › Figure2/Figure 2A Micr. image/20211124 OSM-3-GFP KI_8/Pos0/img_000000000_Confocal-488-Acq_002.tif]

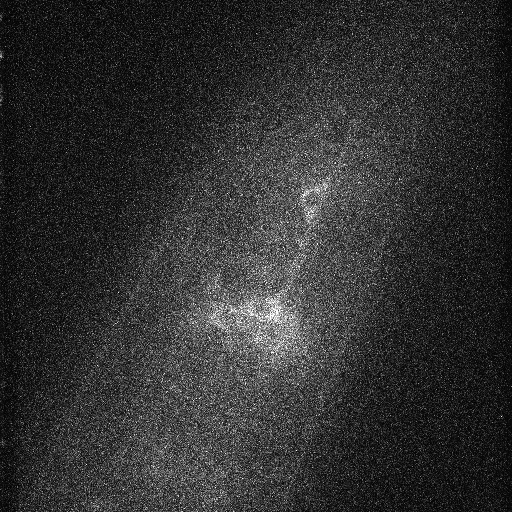

Supplement: Supplementary file 11 — Source data Fig. 2 [file 44318_2024_118_MOESM11_ESM.zip › Figure2/Figure 2A Micr. image/20211124 OSM-3-GFP KI_8/Pos0/img_000000000_Confocal-488-Acq_003.tif]

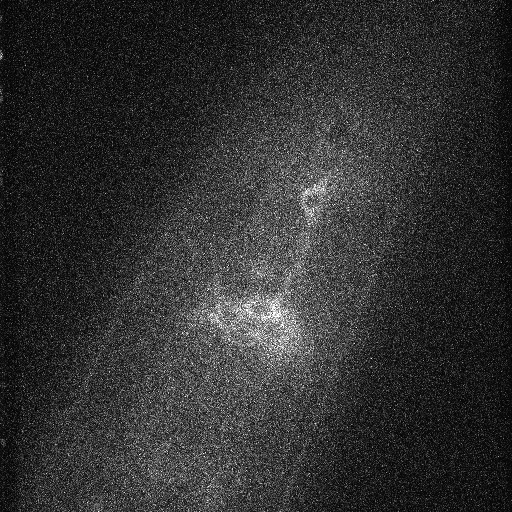

Supplement: Supplementary file 11 — Source data Fig. 2 [file 44318_2024_118_MOESM11_ESM.zip › Figure2/Figure 2A Micr. image/20211124 OSM-3-GFP KI_8/Pos0/img_000000000_Confocal-488-Acq_004.tif]

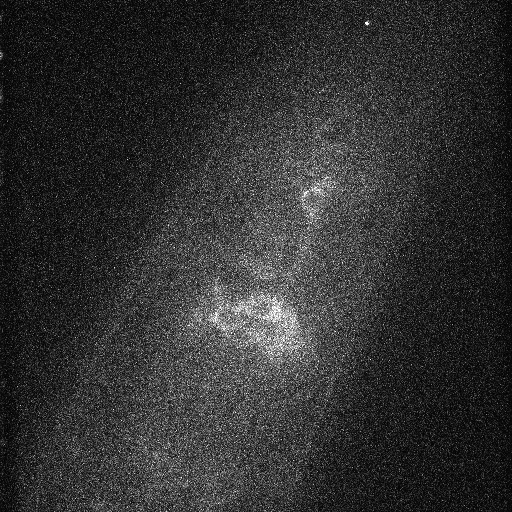

Supplement: Supplementary file 11 — Source data Fig. 2 [file 44318_2024_118_MOESM11_ESM.zip › Figure2/Figure 2A Micr. image/20211124 OSM-3-GFP KI_8/Pos0/img_000000000_Confocal-488-Acq_005.tif]

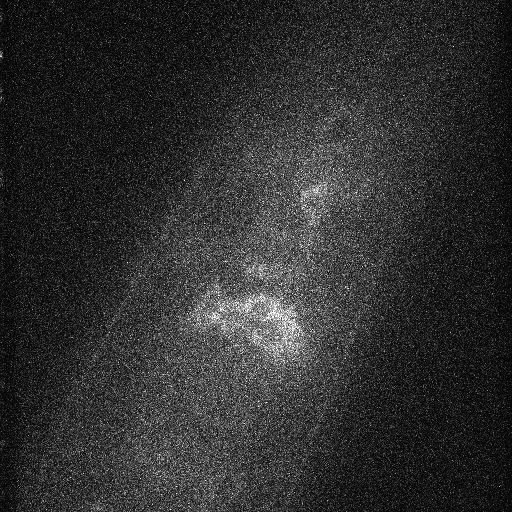

Supplement: Supplementary file 11 — Source data Fig. 2 [file 44318_2024_118_MOESM11_ESM.zip › Figure2/Figure 2A Micr. image/20211124 OSM-3-GFP KI_8/Pos0/img_000000000_Confocal-488-Acq_006.tif]

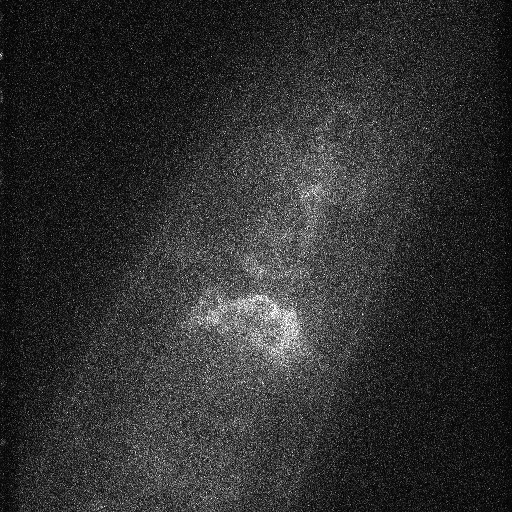

Supplement: Supplementary file 11 — Source data Fig. 2 [file 44318_2024_118_MOESM11_ESM.zip › Figure2/Figure 2A Micr. image/20211124 OSM-3-GFP KI_8/Pos0/img_000000000_Confocal-488-Acq_007.tif]

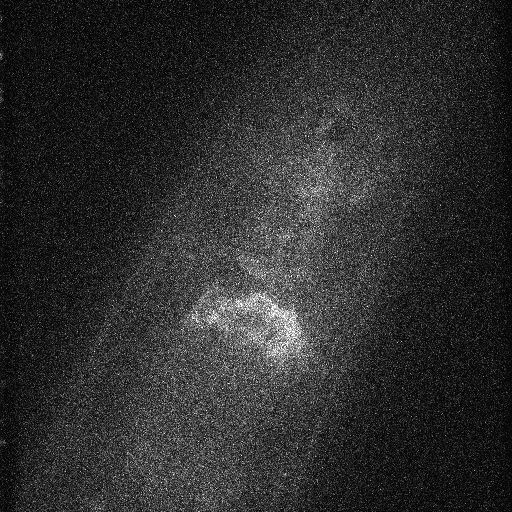

Supplement: Supplementary file 11 — Source data Fig. 2 [file 44318_2024_118_MOESM11_ESM.zip › Figure2/Figure 2A Micr. image/20211124 OSM-3-GFP KI_8/Pos0/img_000000000_Confocal-488-Acq_008.tif]

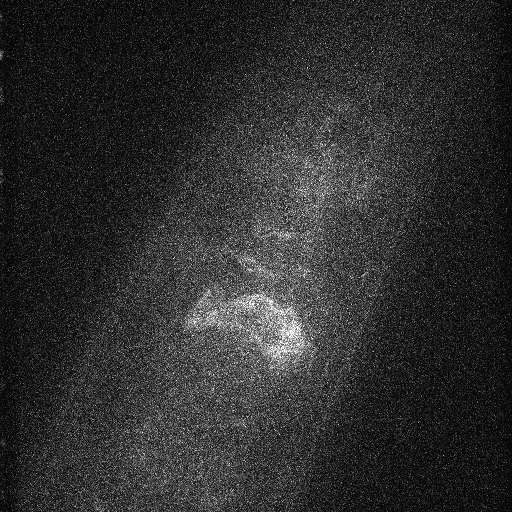

Supplement: Supplementary file 11 — Source data Fig. 2 [file 44318_2024_118_MOESM11_ESM.zip › Figure2/Figure 2A Micr. image/20211124 OSM-3-GFP KI_8/Pos0/img_000000000_Confocal-488-Acq_009.tif]

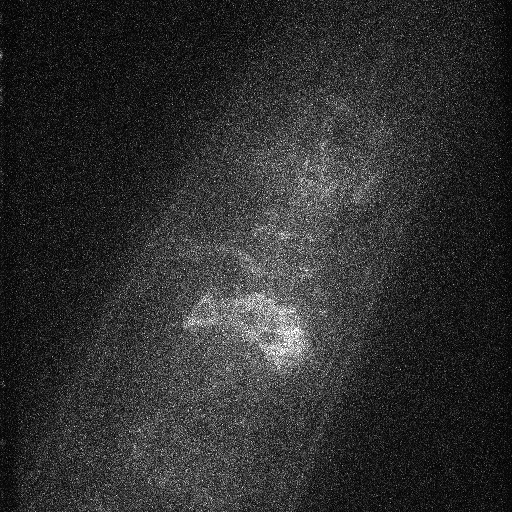

Supplement: Supplementary file 11 — Source data Fig. 2 [file 44318_2024_118_MOESM11_ESM.zip › Figure2/Figure 2A Micr. image/20211124 OSM-3-GFP KI_8/Pos0/img_000000000_Confocal-488-Acq_010.tif]

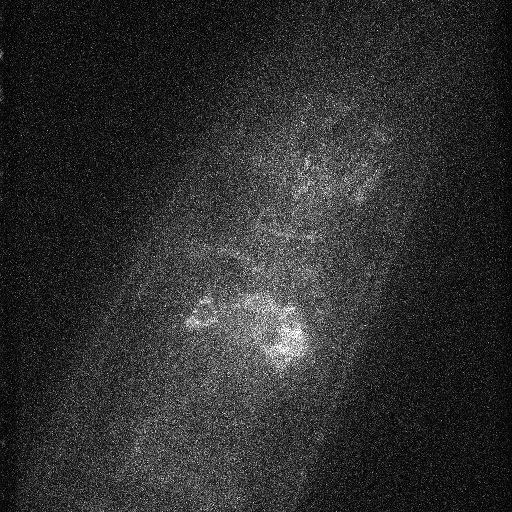

Supplement: Supplementary file 11 — Source data Fig. 2 [file 44318_2024_118_MOESM11_ESM.zip › Figure2/Figure 2A Micr. image/20211124 OSM-3-GFP KI_8/Pos0/img_000000000_Confocal-488-Acq_011.tif]

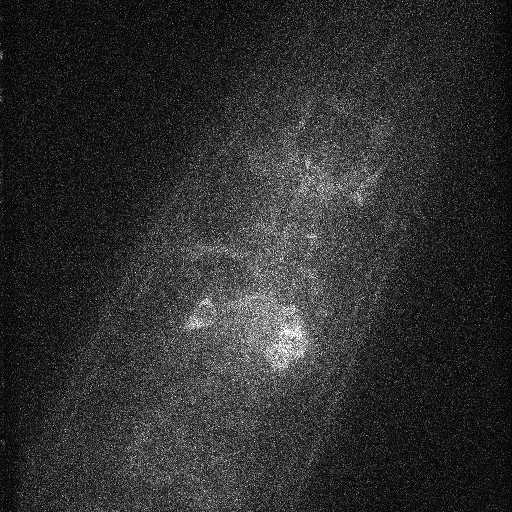

Supplement: Supplementary file 11 — Source data Fig. 2 [file 44318_2024_118_MOESM11_ESM.zip › Figure2/Figure 2A Micr. image/20211124 OSM-3-GFP KI_8/Pos0/img_000000000_Confocal-488-Acq_012.tif]

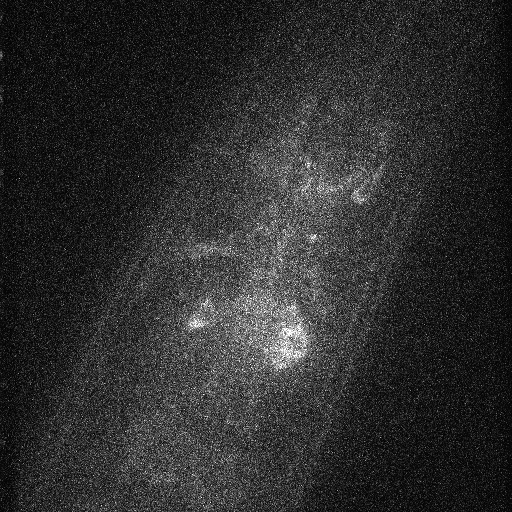

Supplement: Supplementary file 11 — Source data Fig. 2 [file 44318_2024_118_MOESM11_ESM.zip › Figure2/Figure 2A Micr. image/20211124 OSM-3-GFP KI_8/Pos0/img_000000000_Confocal-488-Acq_013.tif]

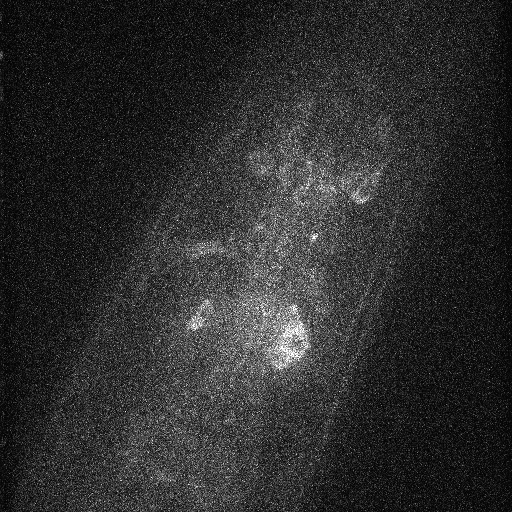

Supplement: Supplementary file 11 — Source data Fig. 2 [file 44318_2024_118_MOESM11_ESM.zip › Figure2/Figure 2A Micr. image/20211124 OSM-3-GFP KI_8/Pos0/img_000000000_Confocal-488-Acq_014.tif]

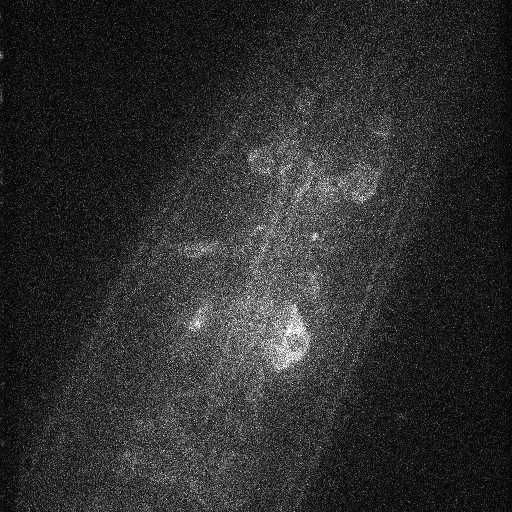

Supplement: Supplementary file 11 — Source data Fig. 2 [file 44318_2024_118_MOESM11_ESM.zip › Figure2/Figure 2A Micr. image/20211124 OSM-3-GFP KI_8/Pos0/img_000000000_Confocal-488-Acq_015.tif]

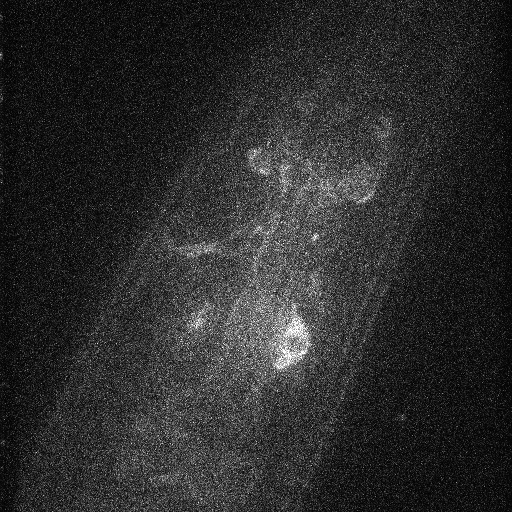

Supplement: Supplementary file 11 — Source data Fig. 2 [file 44318_2024_118_MOESM11_ESM.zip › Figure2/Figure 2A Micr. image/20211124 OSM-3-GFP KI_8/Pos0/img_000000000_Confocal-488-Acq_016.tif]

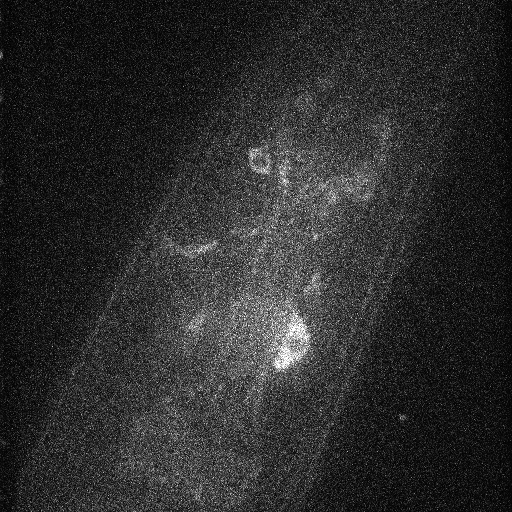

Supplement: Supplementary file 11 — Source data Fig. 2 [file 44318_2024_118_MOESM11_ESM.zip › Figure2/Figure 2A Micr. image/20211124 OSM-3-GFP KI_8/Pos0/img_000000000_Confocal-488-Acq_017.tif]

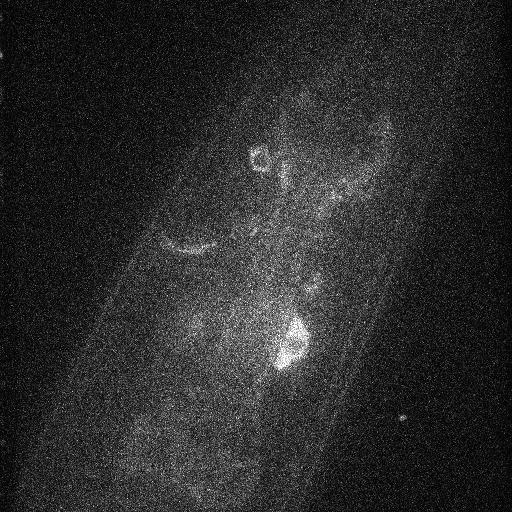

Supplement: Supplementary file 11 — Source data Fig. 2 [file 44318_2024_118_MOESM11_ESM.zip › Figure2/Figure 2A Micr. image/20211124 OSM-3-GFP KI_8/Pos0/img_000000000_Confocal-488-Acq_018.tif]

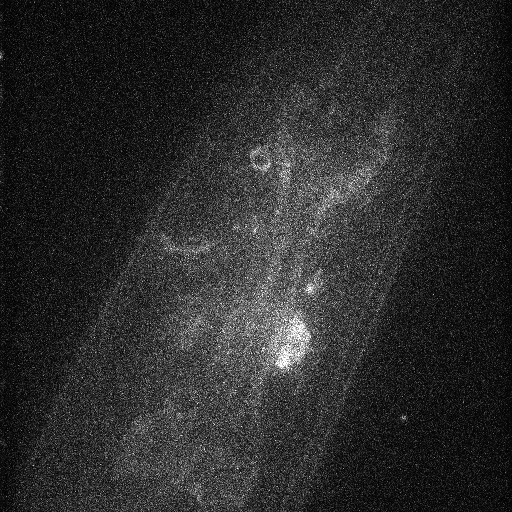

Supplement: Supplementary file 11 — Source data Fig. 2 [file 44318_2024_118_MOESM11_ESM.zip › Figure2/Figure 2A Micr. image/20211124 OSM-3-GFP KI_8/Pos0/img_000000000_Confocal-488-Acq_019.tif]

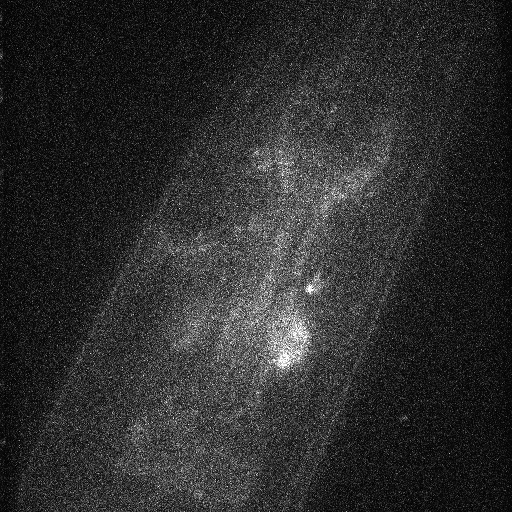

Supplement: Supplementary file 11 — Source data Fig. 2 [file 44318_2024_118_MOESM11_ESM.zip › Figure2/Figure 2A Micr. image/20211124 OSM-3-GFP KI_8/Pos0/img_000000000_Confocal-488-Acq_020.tif]

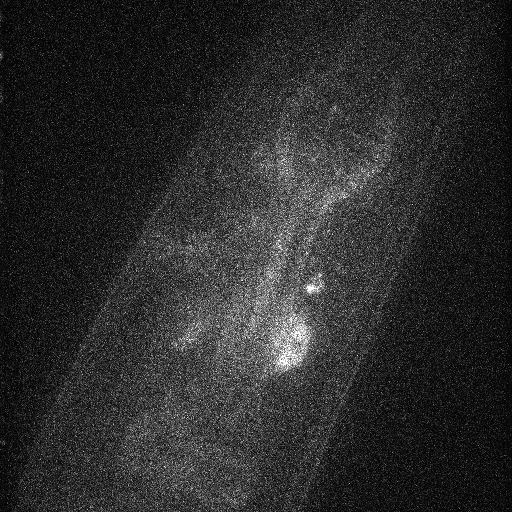

Supplement: Supplementary file 11 — Source data Fig. 2 [file 44318_2024_118_MOESM11_ESM.zip › Figure2/Figure 2A Micr. image/20211124 OSM-3-GFP KI_8/Pos0/img_000000000_Confocal-488-Acq_021.tif]

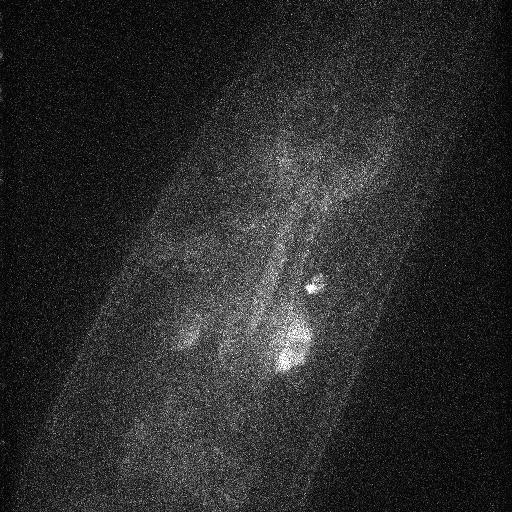

Supplement: Supplementary file 11 — Source data Fig. 2 [file 44318_2024_118_MOESM11_ESM.zip › Figure2/Figure 2A Micr. image/20211124 OSM-3-GFP KI_8/Pos0/img_000000000_Confocal-488-Acq_022.tif]

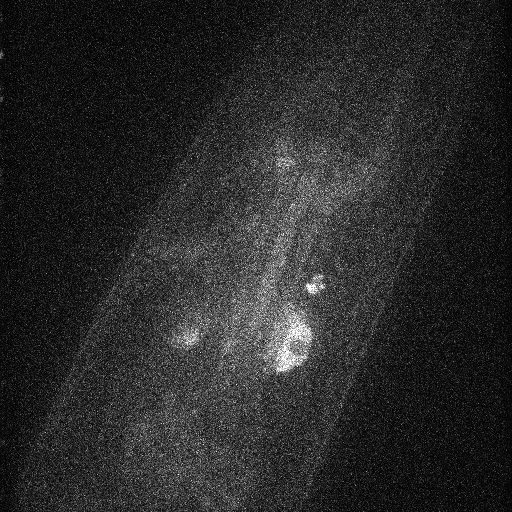

Supplement: Supplementary file 11 — Source data Fig. 2 [file 44318_2024_118_MOESM11_ESM.zip › Figure2/Figure 2A Micr. image/20211124 OSM-3-GFP KI_8/Pos0/img_000000000_Confocal-488-Acq_023.tif]

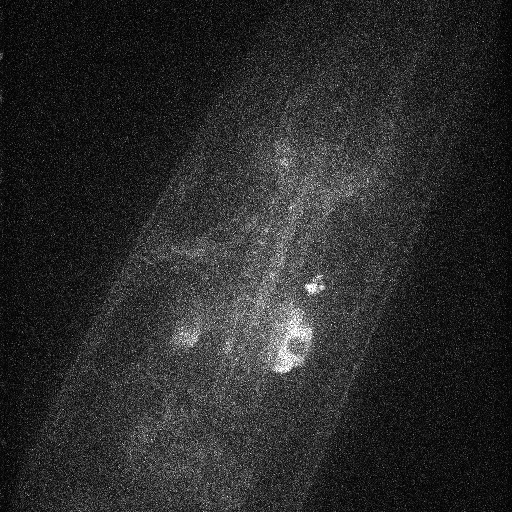

Supplement: Supplementary file 11 — Source data Fig. 2 [file 44318_2024_118_MOESM11_ESM.zip › Figure2/Figure 2A Micr. image/20211124 OSM-3-GFP KI_8/Pos0/img_000000000_Confocal-488-Acq_024.tif]

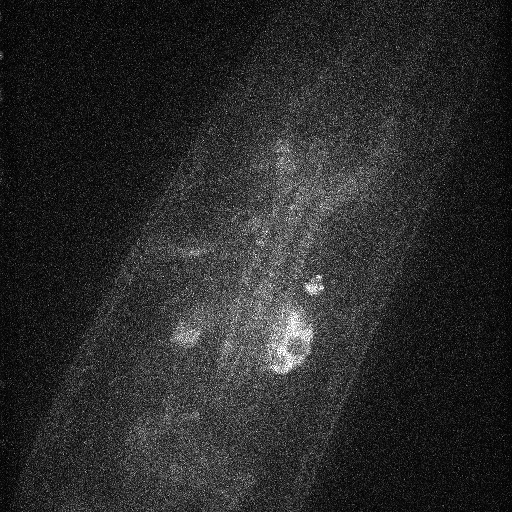

Supplement: Supplementary file 11 — Source data Fig. 2 [file 44318_2024_118_MOESM11_ESM.zip › Figure2/Figure 2A Micr. image/20211124 OSM-3-GFP KI_8/Pos0/img_000000000_Confocal-488-Acq_025.tif]

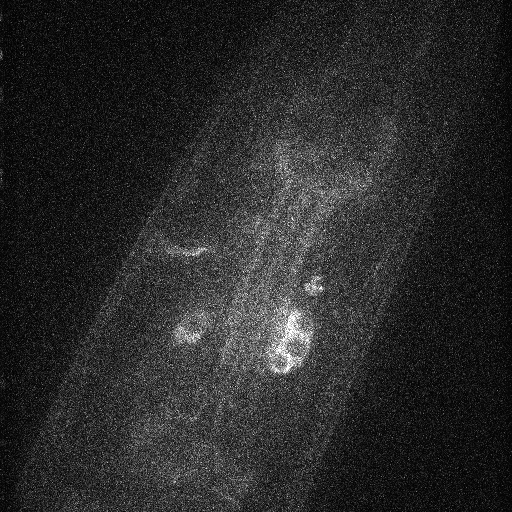

Supplement: Supplementary file 11 — Source data Fig. 2 [file 44318_2024_118_MOESM11_ESM.zip › Figure2/Figure 2A Micr. image/20211124 OSM-3-GFP KI_8/Pos0/img_000000000_Confocal-488-Acq_026.tif]

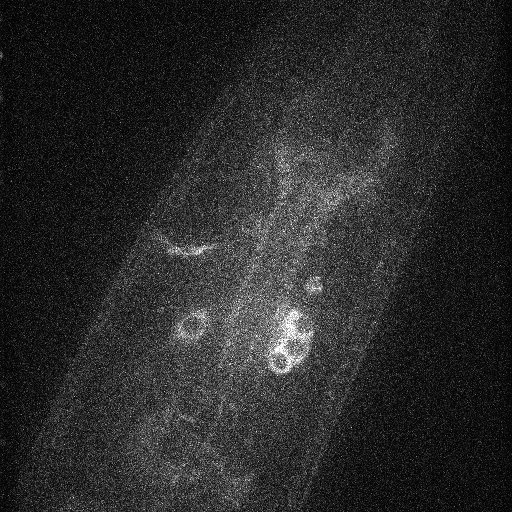

Supplement: Supplementary file 11 — Source data Fig. 2 [file 44318_2024_118_MOESM11_ESM.zip › Figure2/Figure 2A Micr. image/20211124 OSM-3-GFP KI_8/Pos0/img_000000000_Confocal-488-Acq_027.tif]

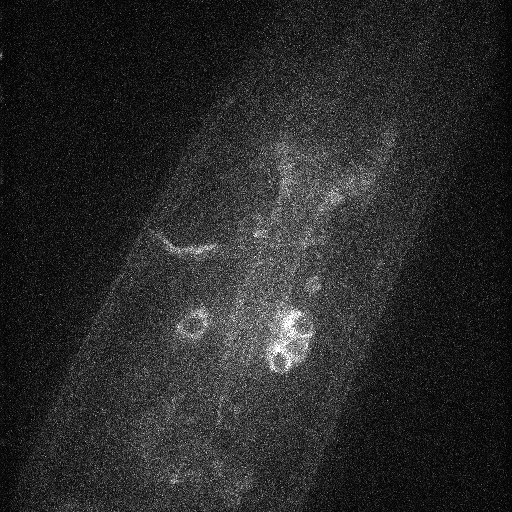

Supplement: Supplementary file 11 — Source data Fig. 2 [file 44318_2024_118_MOESM11_ESM.zip › Figure2/Figure 2A Micr. image/20211124 OSM-3-GFP KI_8/Pos0/img_000000000_Confocal-488-Acq_028.tif]

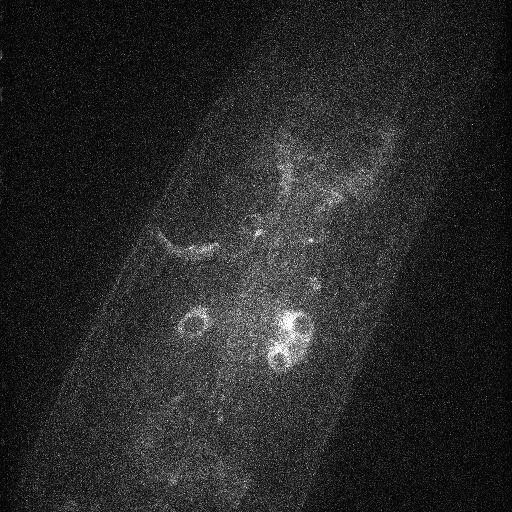

Supplement: Supplementary file 11 — Source data Fig. 2 [file 44318_2024_118_MOESM11_ESM.zip › Figure2/Figure 2A Micr. image/20211124 OSM-3-GFP KI_8/Pos0/img_000000000_Confocal-488-Acq_029.tif]

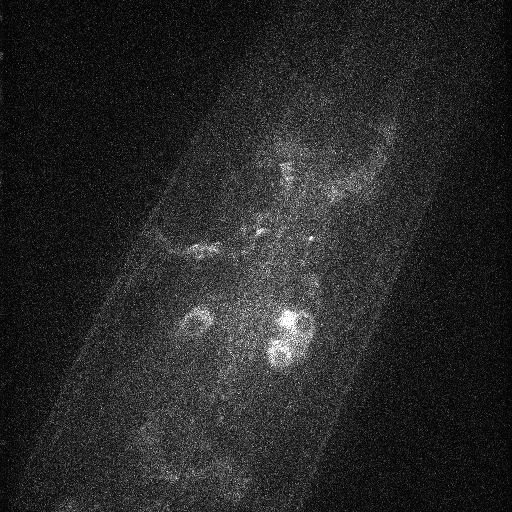

Supplement: Supplementary file 11 — Source data Fig. 2 [file 44318_2024_118_MOESM11_ESM.zip › Figure2/Figure 2A Micr. image/20211124 OSM-3-GFP KI_8/Pos0/img_000000000_Confocal-488-Acq_030.tif]

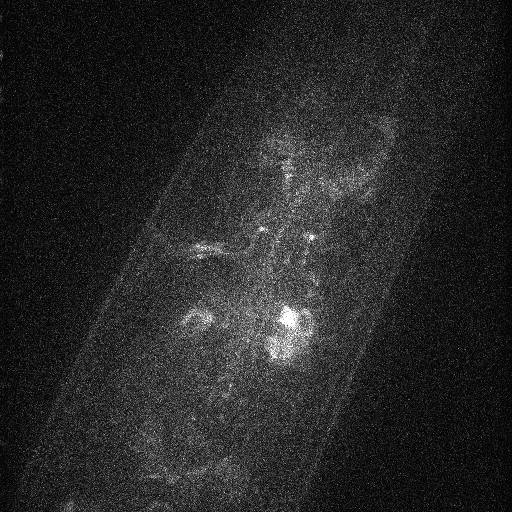

Supplement: Supplementary file 11 — Source data Fig. 2 [file 44318_2024_118_MOESM11_ESM.zip › Figure2/Figure 2A Micr. image/20211124 OSM-3-GFP KI_8/Pos0/img_000000000_Confocal-488-Acq_031.tif]

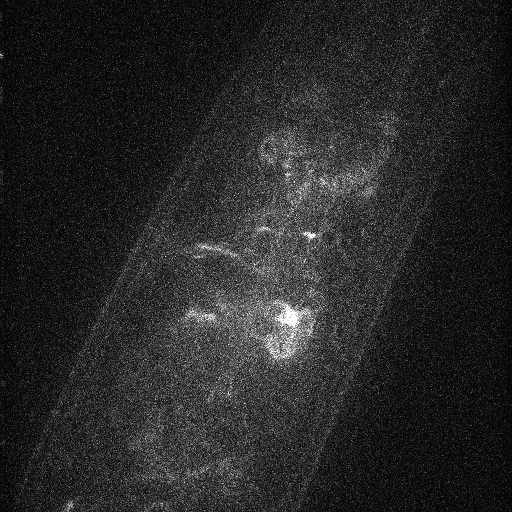

Supplement: Supplementary file 11 — Source data Fig. 2 [file 44318_2024_118_MOESM11_ESM.zip › Figure2/Figure 2A Micr. image/20211124 OSM-3-GFP KI_8/Pos0/img_000000000_Confocal-488-Acq_032.tif]

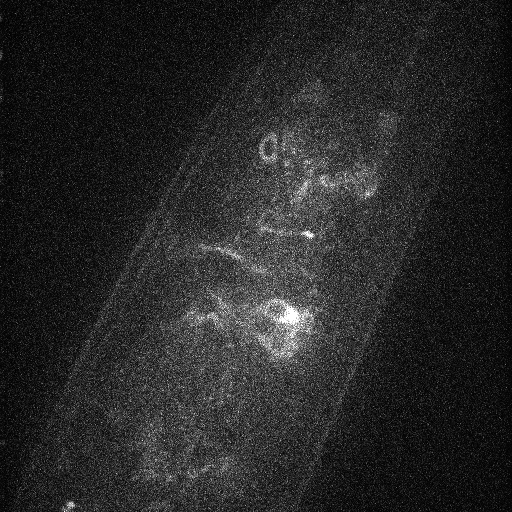

Supplement: Supplementary file 11 — Source data Fig. 2 [file 44318_2024_118_MOESM11_ESM.zip › Figure2/Figure 2A Micr. image/20211124 OSM-3-GFP KI_8/Pos0/img_000000000_Confocal-488-Acq_033.tif]

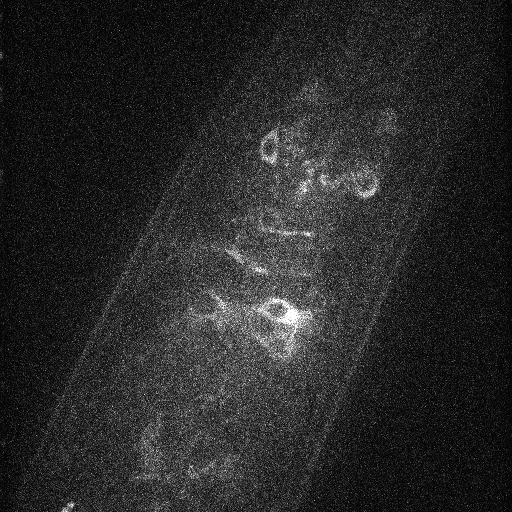

Supplement: Supplementary file 11 — Source data Fig. 2 [file 44318_2024_118_MOESM11_ESM.zip › Figure2/Figure 2A Micr. image/20211124 OSM-3-GFP KI_8/Pos0/img_000000000_Confocal-488-Acq_034.tif]

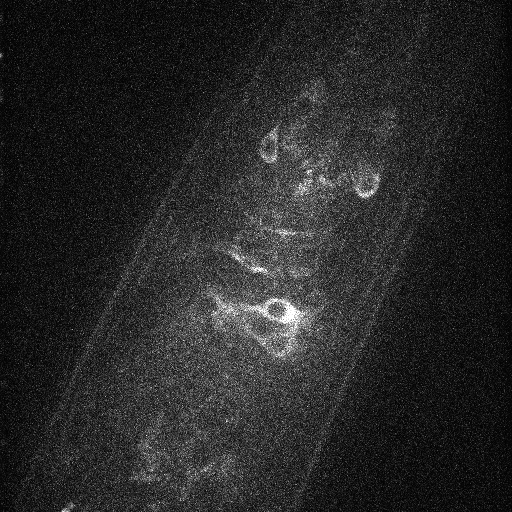

Supplement: Supplementary file 11 — Source data Fig. 2 [file 44318_2024_118_MOESM11_ESM.zip › Figure2/Figure 2A Micr. image/20211124 OSM-3-GFP KI_8/Pos0/img_000000000_Confocal-488-Acq_035.tif]

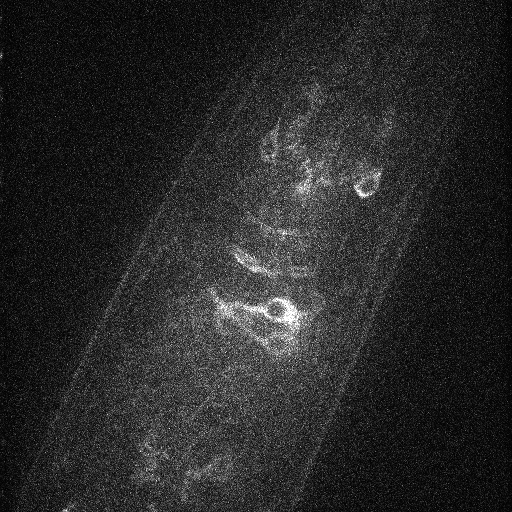

Supplement: Supplementary file 11 — Source data Fig. 2 [file 44318_2024_118_MOESM11_ESM.zip › Figure2/Figure 2A Micr. image/20211124 OSM-3-GFP KI_8/Pos0/img_000000000_Confocal-488-Acq_036.tif]

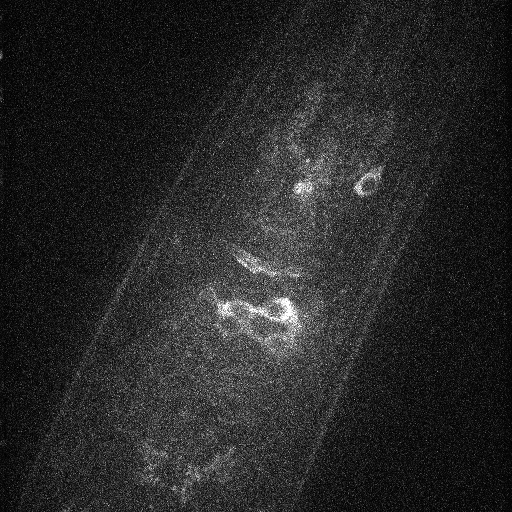

Supplement: Supplementary file 11 — Source data Fig. 2 [file 44318_2024_118_MOESM11_ESM.zip › Figure2/Figure 2A Micr. image/20211124 OSM-3-GFP KI_8/Pos0/img_000000000_Confocal-488-Acq_037.tif]

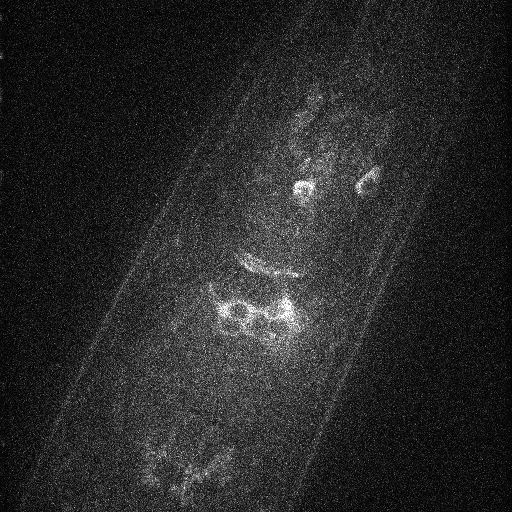

Supplement: Supplementary file 11 — Source data Fig. 2 [file 44318_2024_118_MOESM11_ESM.zip › Figure2/Figure 2A Micr. image/20211124 OSM-3-GFP KI_8/Pos0/img_000000000_Confocal-488-Acq_038.tif]

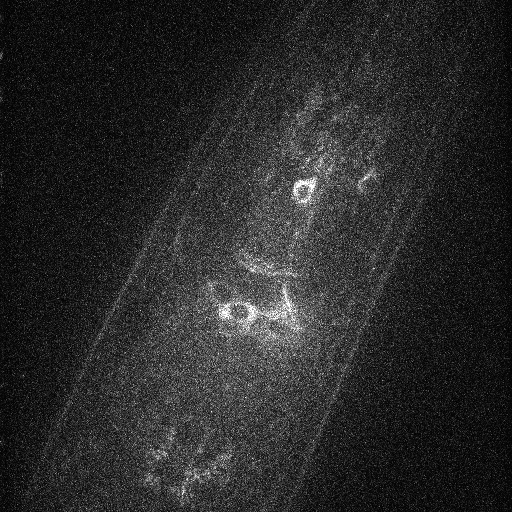

Supplement: Supplementary file 11 — Source data Fig. 2 [file 44318_2024_118_MOESM11_ESM.zip › Figure2/Figure 2A Micr. image/20211124 OSM-3-GFP KI_8/Pos0/img_000000000_Confocal-488-Acq_039.tif]

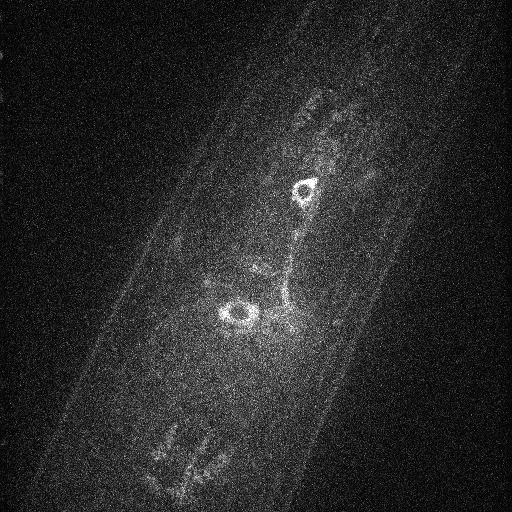

Supplement: Supplementary file 11 — Source data Fig. 2 [file 44318_2024_118_MOESM11_ESM.zip › Figure2/Figure 2A Micr. image/20211124 OSM-3-GFP KI_8/Pos0/img_000000000_Confocal-488-Acq_040.tif]

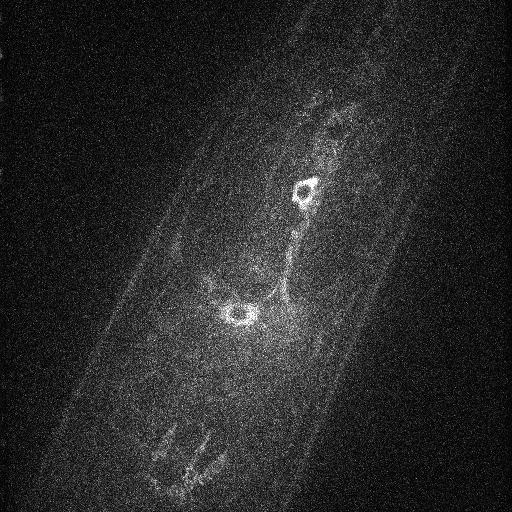

Supplement: Supplementary file 11 — Source data Fig. 2 [file 44318_2024_118_MOESM11_ESM.zip › Figure2/Figure 2A Micr. image/20211124 OSM-3-GFP KI_8/Pos0/img_000000000_Confocal-488-Acq_041.tif]

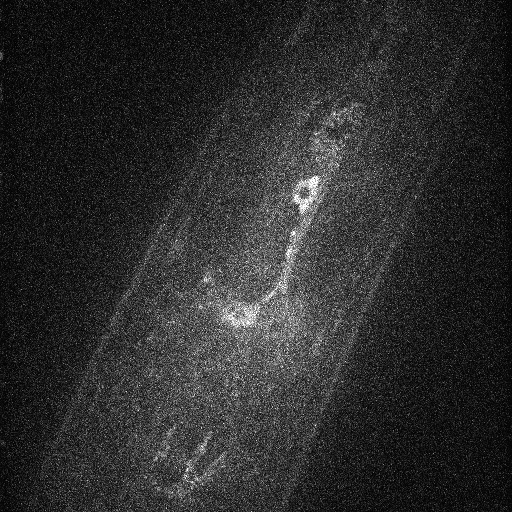

Supplement: Supplementary file 11 — Source data Fig. 2 [file 44318_2024_118_MOESM11_ESM.zip › Figure2/Figure 2A Micr. image/20211124 OSM-3-GFP KI_8/Pos0/img_000000000_Confocal-488-Acq_042.tif]

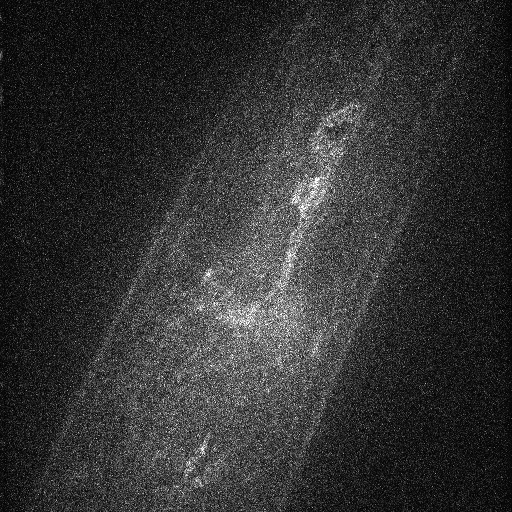

Supplement: Supplementary file 11 — Source data Fig. 2 [file 44318_2024_118_MOESM11_ESM.zip › Figure2/Figure 2A Micr. image/20211124 OSM-3-GFP KI_8/Pos0/img_000000000_Confocal-488-Acq_043.tif]

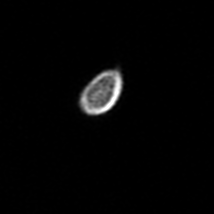

Supplement: Supplementary file 12 — Source data Fig. 3 [file 44318_2024_118_MOESM12_ESM.zip › Figure3/Figure 3A time series video/2.tif]

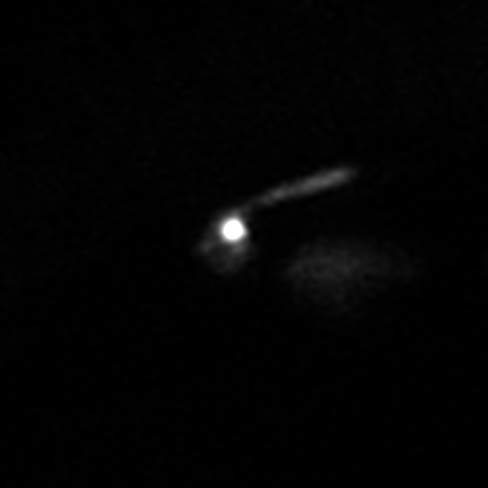

Supplement: Supplementary file 12 — Source data Fig. 3 [file 44318_2024_118_MOESM12_ESM.zip › Figure3/Figure 3C time series video/13.tif]

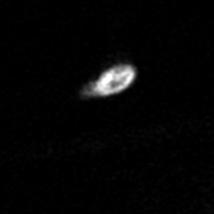

Supplement: Supplementary file 12 — Source data Fig. 3 [file 44318_2024_118_MOESM12_ESM.zip › Figure3/Figure 3F time series video/12.tif]

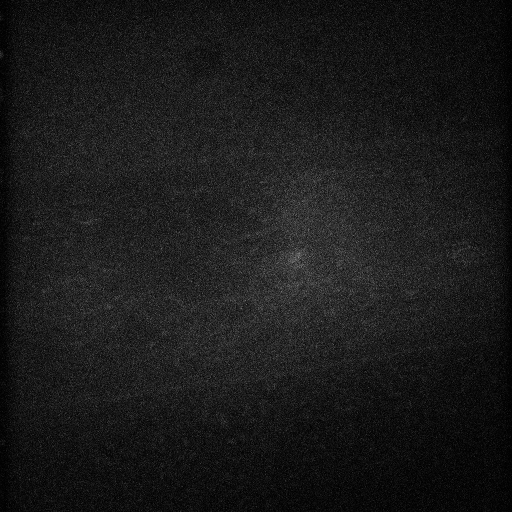

Supplement: Supplementary file 13 — Source data Fig. 4 [file 44318_2024_118_MOESM13_ESM.zip › Figure4/Figure 4B Micr. image/20210908 Phlh-17-mCherry; osm-3-g444e-gfp100x_5/Pos0/img_000000000_Confocal-488-Acq_000.tif]

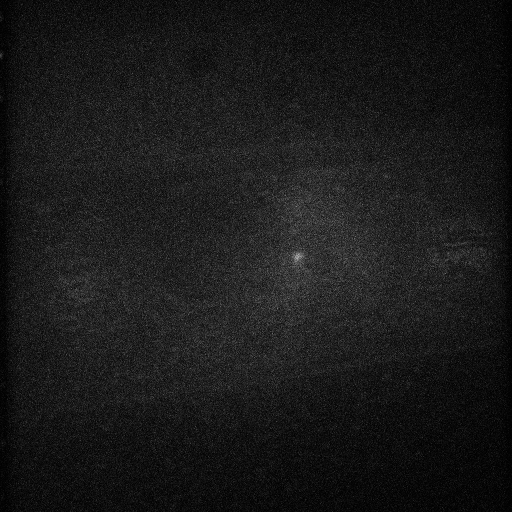

Supplement: Supplementary file 13 — Source data Fig. 4 [file 44318_2024_118_MOESM13_ESM.zip › Figure4/Figure 4B Micr. image/20210908 Phlh-17-mCherry; osm-3-g444e-gfp100x_5/Pos0/img_000000000_Confocal-488-Acq_001.tif]

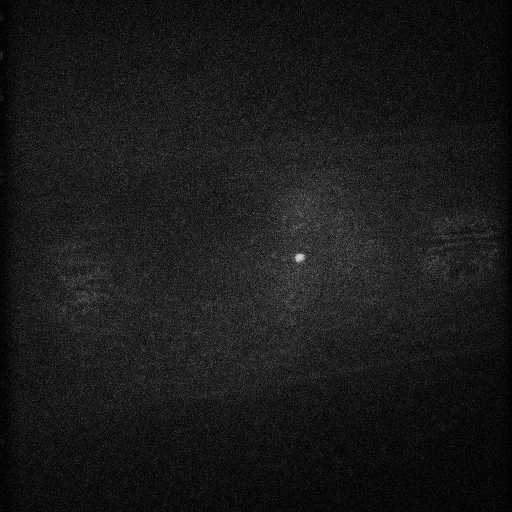

Supplement: Supplementary file 13 — Source data Fig. 4 [file 44318_2024_118_MOESM13_ESM.zip › Figure4/Figure 4B Micr. image/20210908 Phlh-17-mCherry; osm-3-g444e-gfp100x_5/Pos0/img_000000000_Confocal-488-Acq_002.tif]

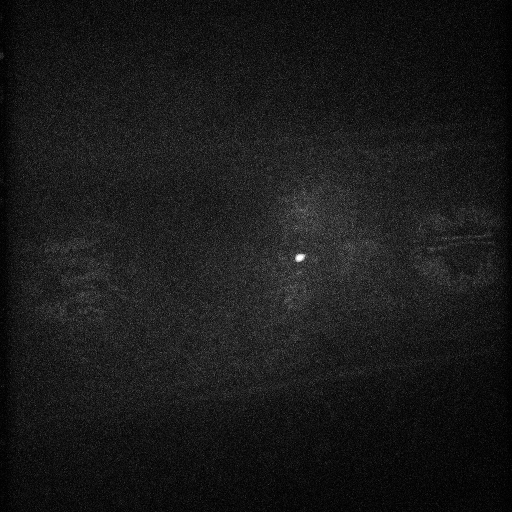

Supplement: Supplementary file 13 — Source data Fig. 4 [file 44318_2024_118_MOESM13_ESM.zip › Figure4/Figure 4B Micr. image/20210908 Phlh-17-mCherry; osm-3-g444e-gfp100x_5/Pos0/img_000000000_Confocal-488-Acq_003.tif]

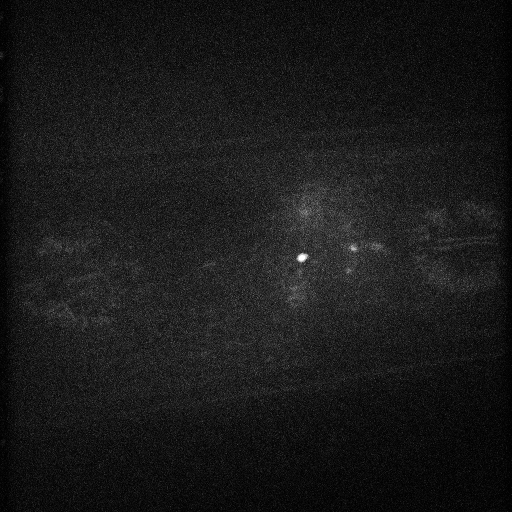

Supplement: Supplementary file 13 — Source data Fig. 4 [file 44318_2024_118_MOESM13_ESM.zip › Figure4/Figure 4B Micr. image/20210908 Phlh-17-mCherry; osm-3-g444e-gfp100x_5/Pos0/img_000000000_Confocal-488-Acq_004.tif]

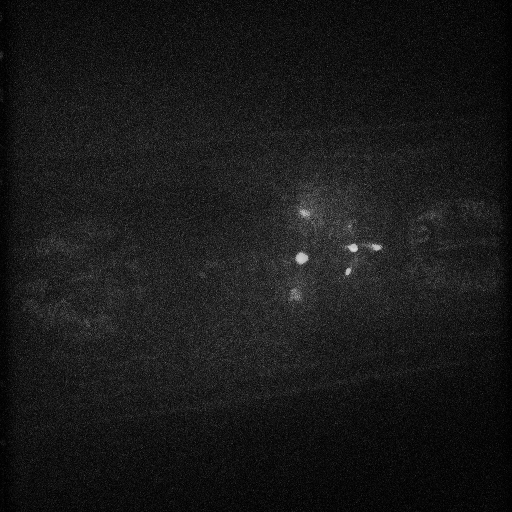

Supplement: Supplementary file 13 — Source data Fig. 4 [file 44318_2024_118_MOESM13_ESM.zip › Figure4/Figure 4B Micr. image/20210908 Phlh-17-mCherry; osm-3-g444e-gfp100x_5/Pos0/img_000000000_Confocal-488-Acq_005.tif]

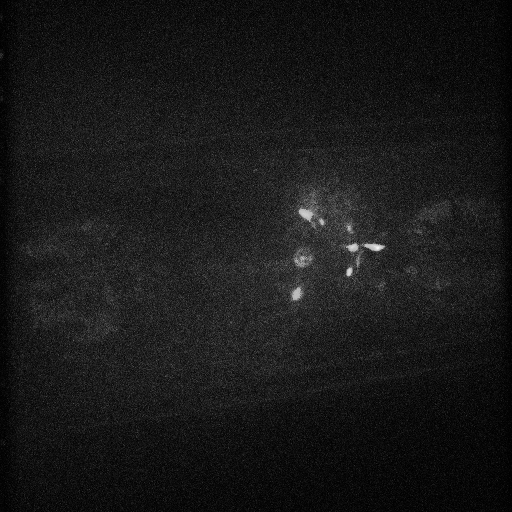

Supplement: Supplementary file 13 — Source data Fig. 4 [file 44318_2024_118_MOESM13_ESM.zip › Figure4/Figure 4B Micr. image/20210908 Phlh-17-mCherry; osm-3-g444e-gfp100x_5/Pos0/img_000000000_Confocal-488-Acq_006.tif]

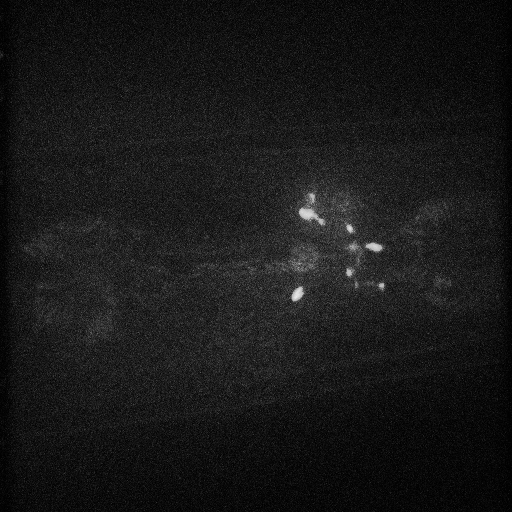

Supplement: Supplementary file 13 — Source data Fig. 4 [file 44318_2024_118_MOESM13_ESM.zip › Figure4/Figure 4B Micr. image/20210908 Phlh-17-mCherry; osm-3-g444e-gfp100x_5/Pos0/img_000000000_Confocal-488-Acq_007.tif]

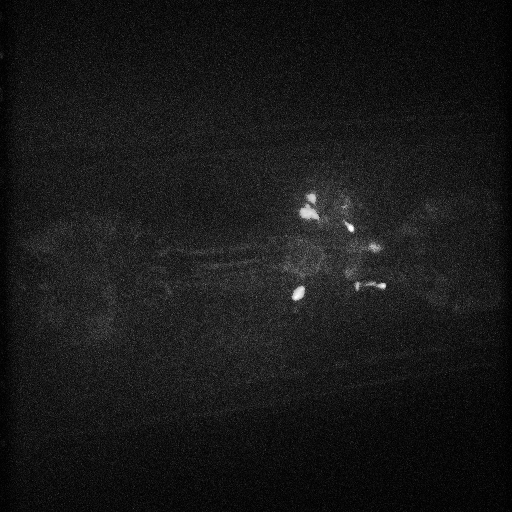

Supplement: Supplementary file 13 — Source data Fig. 4 [file 44318_2024_118_MOESM13_ESM.zip › Figure4/Figure 4B Micr. image/20210908 Phlh-17-mCherry; osm-3-g444e-gfp100x_5/Pos0/img_000000000_Confocal-488-Acq_008.tif]

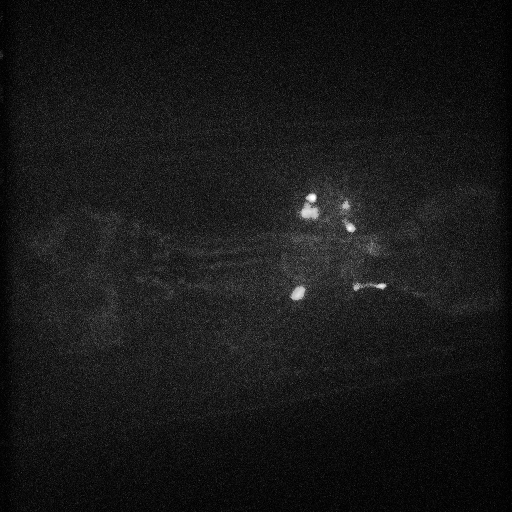

Supplement: Supplementary file 13 — Source data Fig. 4 [file 44318_2024_118_MOESM13_ESM.zip › Figure4/Figure 4B Micr. image/20210908 Phlh-17-mCherry; osm-3-g444e-gfp100x_5/Pos0/img_000000000_Confocal-488-Acq_009.tif]

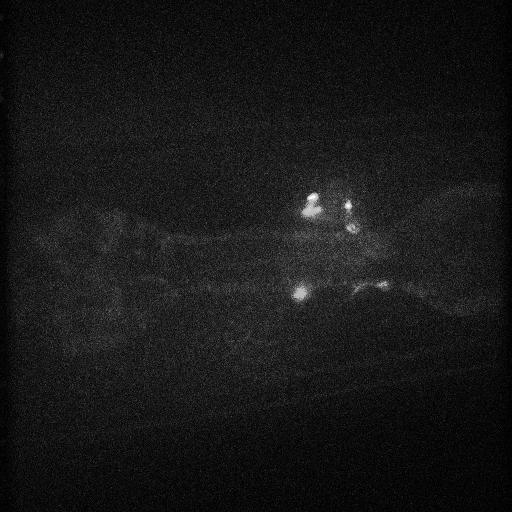

Supplement: Supplementary file 13 — Source data Fig. 4 [file 44318_2024_118_MOESM13_ESM.zip › Figure4/Figure 4B Micr. image/20210908 Phlh-17-mCherry; osm-3-g444e-gfp100x_5/Pos0/img_000000000_Confocal-488-Acq_010.tif]

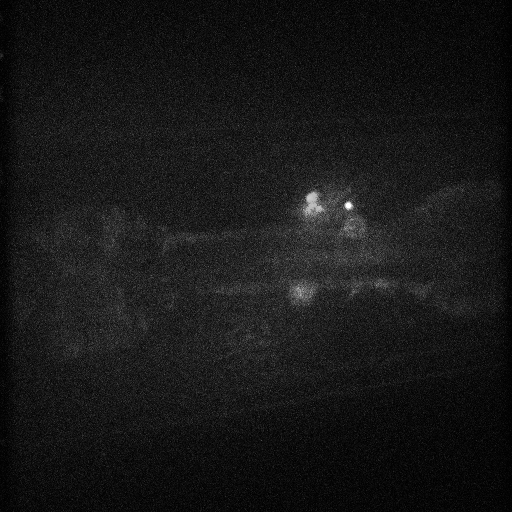

Supplement: Supplementary file 13 — Source data Fig. 4 [file 44318_2024_118_MOESM13_ESM.zip › Figure4/Figure 4B Micr. image/20210908 Phlh-17-mCherry; osm-3-g444e-gfp100x_5/Pos0/img_000000000_Confocal-488-Acq_011.tif]

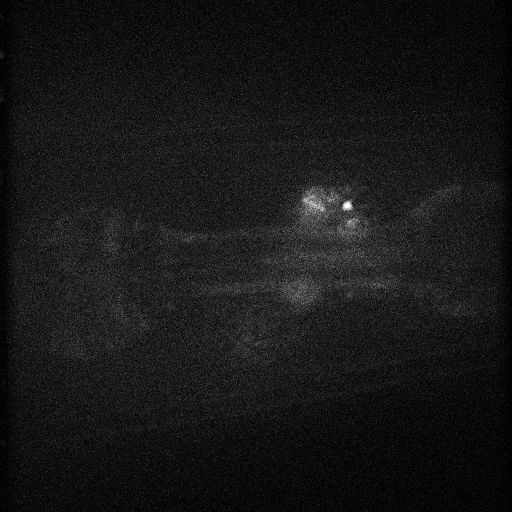

Supplement: Supplementary file 13 — Source data Fig. 4 [file 44318_2024_118_MOESM13_ESM.zip › Figure4/Figure 4B Micr. image/20210908 Phlh-17-mCherry; osm-3-g444e-gfp100x_5/Pos0/img_000000000_Confocal-488-Acq_012.tif]

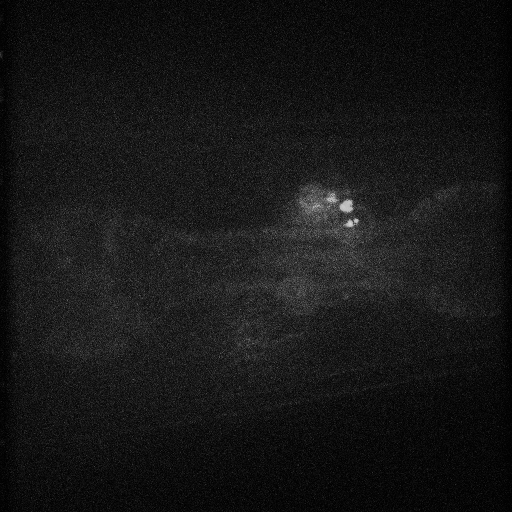

Supplement: Supplementary file 13 — Source data Fig. 4 [file 44318_2024_118_MOESM13_ESM.zip › Figure4/Figure 4B Micr. image/20210908 Phlh-17-mCherry; osm-3-g444e-gfp100x_5/Pos0/img_000000000_Confocal-488-Acq_013.tif]

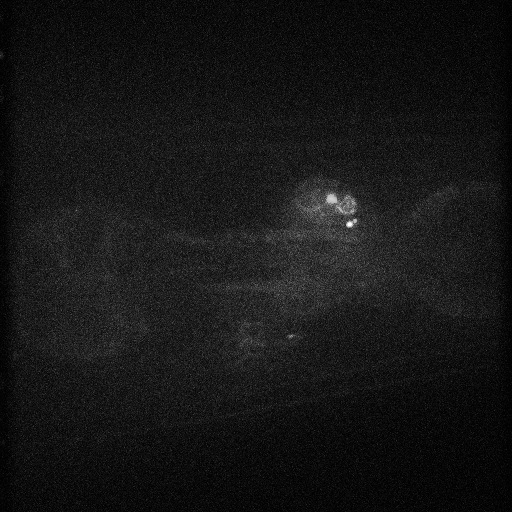

Supplement: Supplementary file 13 — Source data Fig. 4 [file 44318_2024_118_MOESM13_ESM.zip › Figure4/Figure 4B Micr. image/20210908 Phlh-17-mCherry; osm-3-g444e-gfp100x_5/Pos0/img_000000000_Confocal-488-Acq_014.tif]

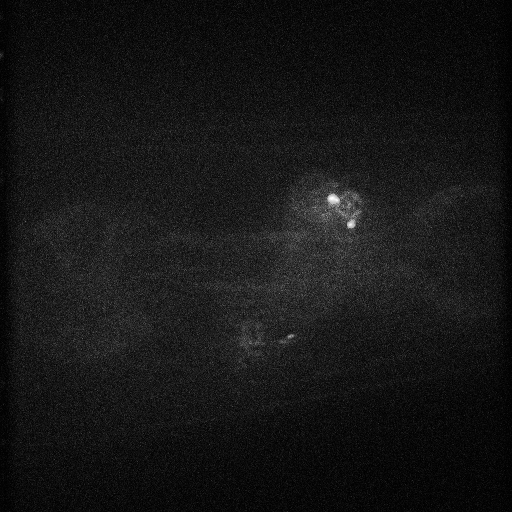

Supplement: Supplementary file 13 — Source data Fig. 4 [file 44318_2024_118_MOESM13_ESM.zip › Figure4/Figure 4B Micr. image/20210908 Phlh-17-mCherry; osm-3-g444e-gfp100x_5/Pos0/img_000000000_Confocal-488-Acq_015.tif]

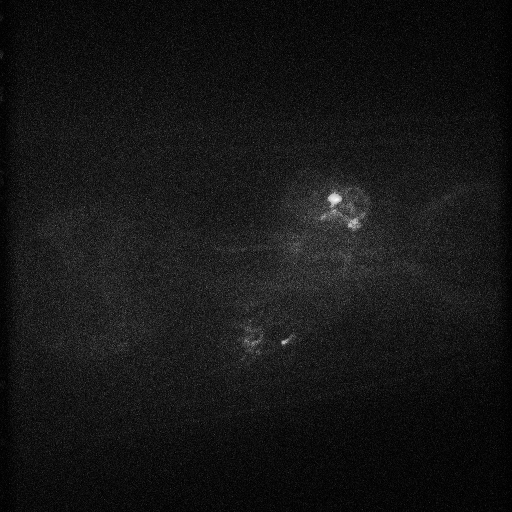

Supplement: Supplementary file 13 — Source data Fig. 4 [file 44318_2024_118_MOESM13_ESM.zip › Figure4/Figure 4B Micr. image/20210908 Phlh-17-mCherry; osm-3-g444e-gfp100x_5/Pos0/img_000000000_Confocal-488-Acq_016.tif]

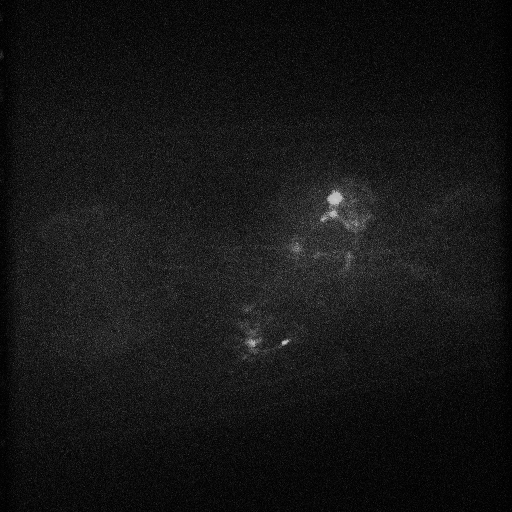

Supplement: Supplementary file 13 — Source data Fig. 4 [file 44318_2024_118_MOESM13_ESM.zip › Figure4/Figure 4B Micr. image/20210908 Phlh-17-mCherry; osm-3-g444e-gfp100x_5/Pos0/img_000000000_Confocal-488-Acq_017.tif]

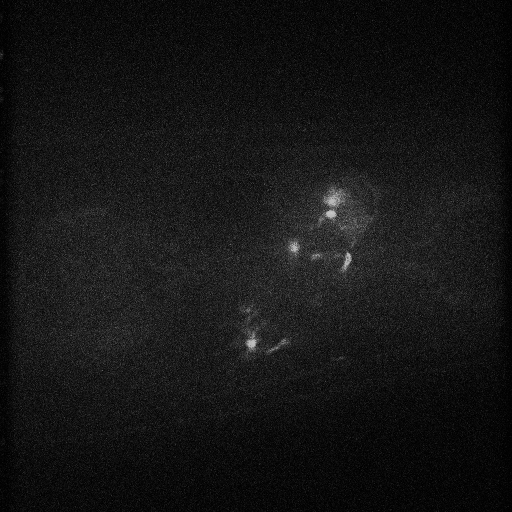

Supplement: Supplementary file 13 — Source data Fig. 4 [file 44318_2024_118_MOESM13_ESM.zip › Figure4/Figure 4B Micr. image/20210908 Phlh-17-mCherry; osm-3-g444e-gfp100x_5/Pos0/img_000000000_Confocal-488-Acq_018.tif]

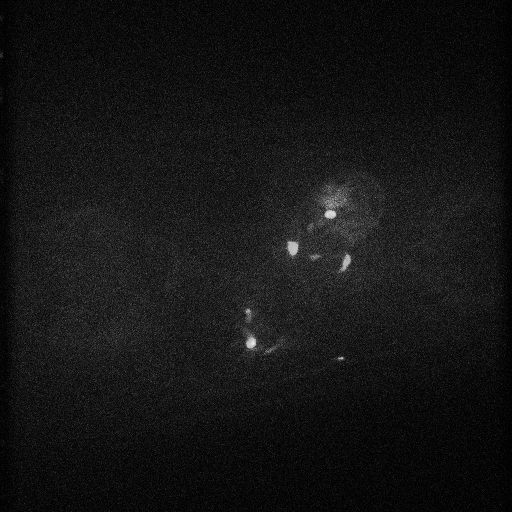

Supplement: Supplementary file 13 — Source data Fig. 4 [file 44318_2024_118_MOESM13_ESM.zip › Figure4/Figure 4B Micr. image/20210908 Phlh-17-mCherry; osm-3-g444e-gfp100x_5/Pos0/img_000000000_Confocal-488-Acq_019.tif]
