# Supplementary figures and images for: Neurons dispose of hyperactive kinesin into glial cells for clearance (part 3 of 9)
Source: EMBO J. 2024 May 28;43(13):5. doi: 10.1038/s44318-024-00118-0 (PMC11217292; doi:10.1038/s44318-024-00118-0)

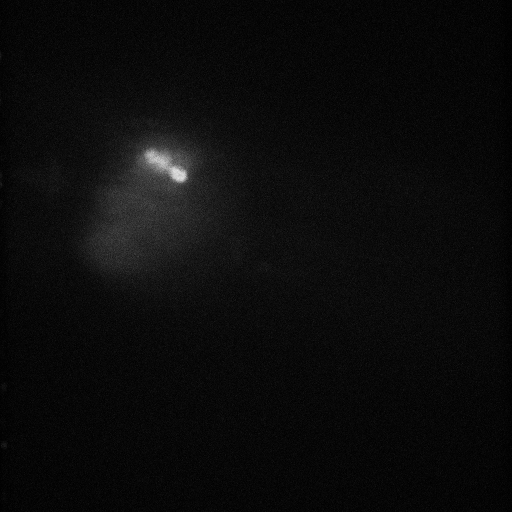

Supplement: Supplementary file 11 — Source data Fig. 2 [file 44318_2024_118_MOESM11_ESM.zip › Figure2/Figure 2A Micr. image/20201128 osm-3 G444E-gfp; HIS-54-BFP_3/Pos0/img_000000000_Confocal-405_020.tif]

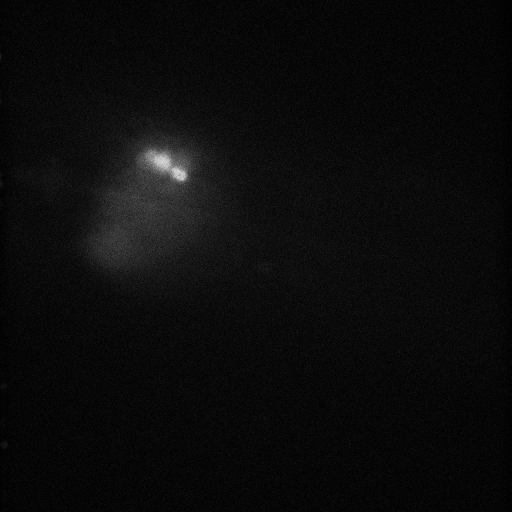

Supplement: Supplementary file 11 — Source data Fig. 2 [file 44318_2024_118_MOESM11_ESM.zip › Figure2/Figure 2A Micr. image/20201128 osm-3 G444E-gfp; HIS-54-BFP_3/Pos0/img_000000000_Confocal-405_021.tif]

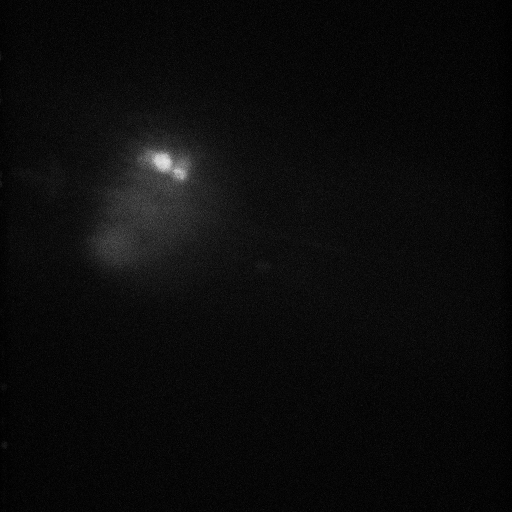

Supplement: Supplementary file 11 — Source data Fig. 2 [file 44318_2024_118_MOESM11_ESM.zip › Figure2/Figure 2A Micr. image/20201128 osm-3 G444E-gfp; HIS-54-BFP_3/Pos0/img_000000000_Confocal-405_022.tif]

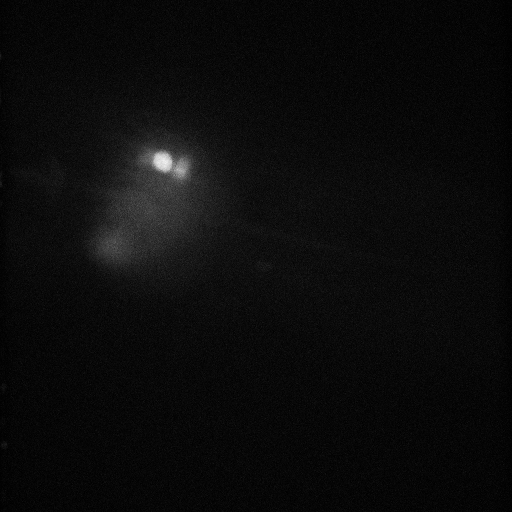

Supplement: Supplementary file 11 — Source data Fig. 2 [file 44318_2024_118_MOESM11_ESM.zip › Figure2/Figure 2A Micr. image/20201128 osm-3 G444E-gfp; HIS-54-BFP_3/Pos0/img_000000000_Confocal-405_023.tif]

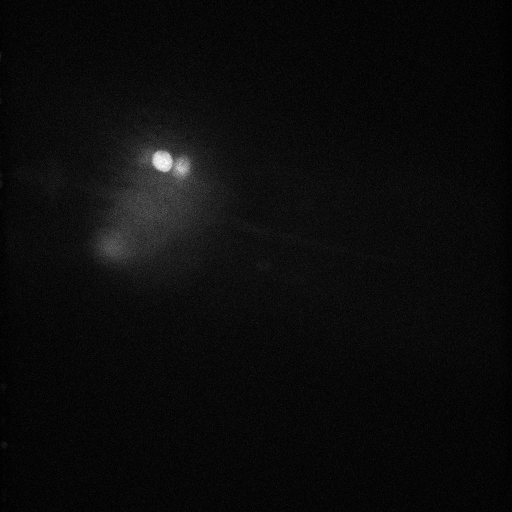

Supplement: Supplementary file 11 — Source data Fig. 2 [file 44318_2024_118_MOESM11_ESM.zip › Figure2/Figure 2A Micr. image/20201128 osm-3 G444E-gfp; HIS-54-BFP_3/Pos0/img_000000000_Confocal-405_024.tif]

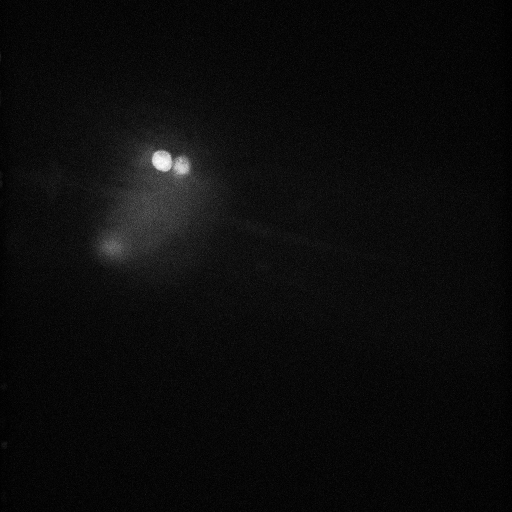

Supplement: Supplementary file 11 — Source data Fig. 2 [file 44318_2024_118_MOESM11_ESM.zip › Figure2/Figure 2A Micr. image/20201128 osm-3 G444E-gfp; HIS-54-BFP_3/Pos0/img_000000000_Confocal-405_025.tif]

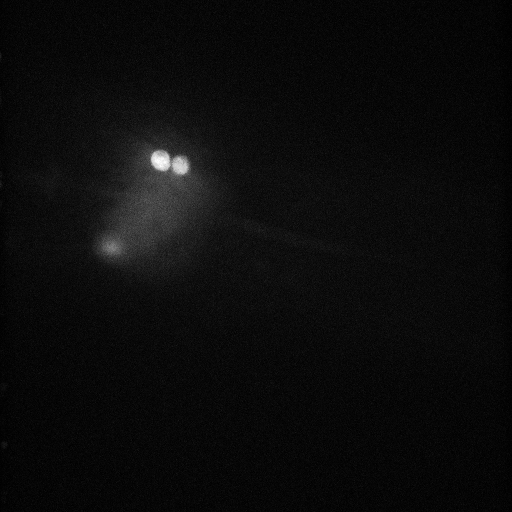

Supplement: Supplementary file 11 — Source data Fig. 2 [file 44318_2024_118_MOESM11_ESM.zip › Figure2/Figure 2A Micr. image/20201128 osm-3 G444E-gfp; HIS-54-BFP_3/Pos0/img_000000000_Confocal-405_026.tif]

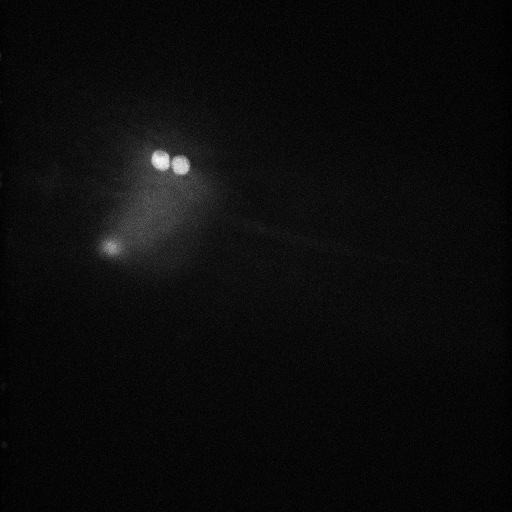

Supplement: Supplementary file 11 — Source data Fig. 2 [file 44318_2024_118_MOESM11_ESM.zip › Figure2/Figure 2A Micr. image/20201128 osm-3 G444E-gfp; HIS-54-BFP_3/Pos0/img_000000000_Confocal-405_027.tif]

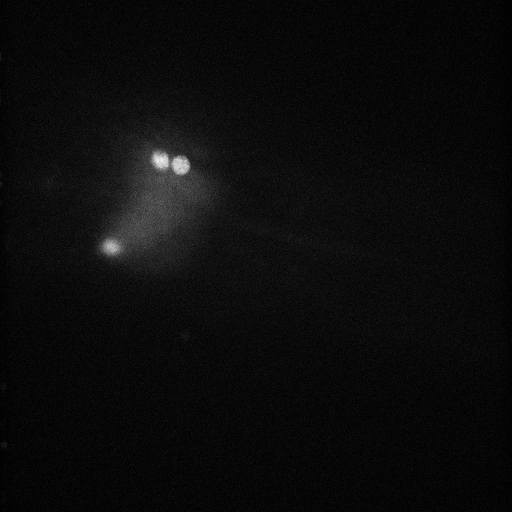

Supplement: Supplementary file 11 — Source data Fig. 2 [file 44318_2024_118_MOESM11_ESM.zip › Figure2/Figure 2A Micr. image/20201128 osm-3 G444E-gfp; HIS-54-BFP_3/Pos0/img_000000000_Confocal-405_028.tif]

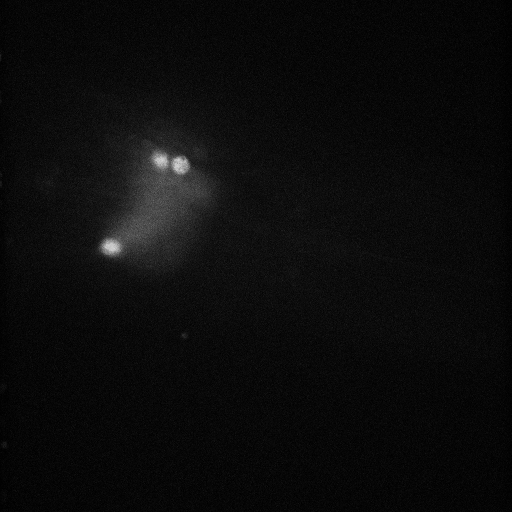

Supplement: Supplementary file 11 — Source data Fig. 2 [file 44318_2024_118_MOESM11_ESM.zip › Figure2/Figure 2A Micr. image/20201128 osm-3 G444E-gfp; HIS-54-BFP_3/Pos0/img_000000000_Confocal-405_029.tif]

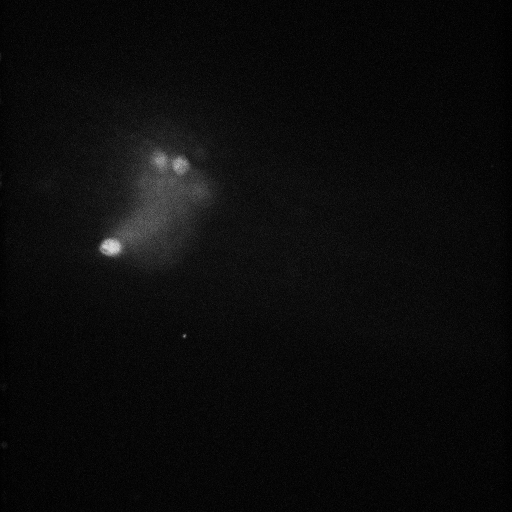

Supplement: Supplementary file 11 — Source data Fig. 2 [file 44318_2024_118_MOESM11_ESM.zip › Figure2/Figure 2A Micr. image/20201128 osm-3 G444E-gfp; HIS-54-BFP_3/Pos0/img_000000000_Confocal-405_030.tif]

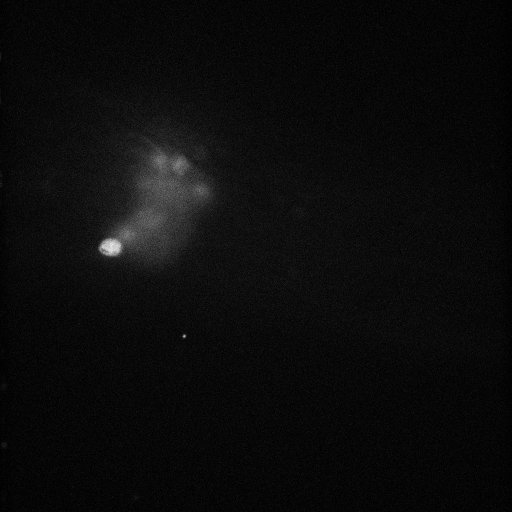

Supplement: Supplementary file 11 — Source data Fig. 2 [file 44318_2024_118_MOESM11_ESM.zip › Figure2/Figure 2A Micr. image/20201128 osm-3 G444E-gfp; HIS-54-BFP_3/Pos0/img_000000000_Confocal-405_031.tif]

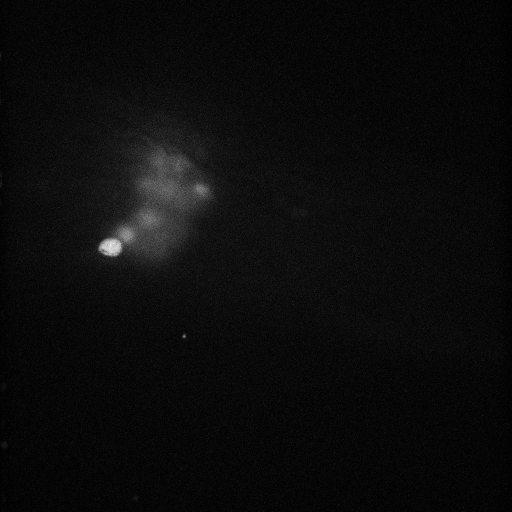

Supplement: Supplementary file 11 — Source data Fig. 2 [file 44318_2024_118_MOESM11_ESM.zip › Figure2/Figure 2A Micr. image/20201128 osm-3 G444E-gfp; HIS-54-BFP_3/Pos0/img_000000000_Confocal-405_032.tif]

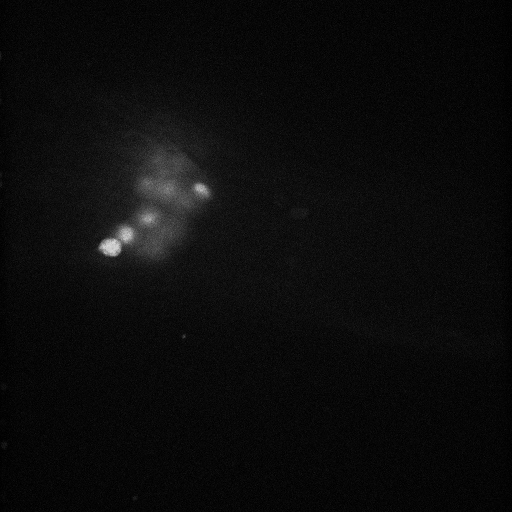

Supplement: Supplementary file 11 — Source data Fig. 2 [file 44318_2024_118_MOESM11_ESM.zip › Figure2/Figure 2A Micr. image/20201128 osm-3 G444E-gfp; HIS-54-BFP_3/Pos0/img_000000000_Confocal-405_033.tif]

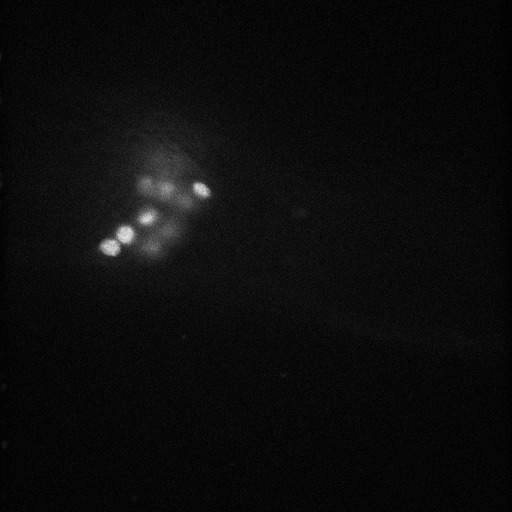

Supplement: Supplementary file 11 — Source data Fig. 2 [file 44318_2024_118_MOESM11_ESM.zip › Figure2/Figure 2A Micr. image/20201128 osm-3 G444E-gfp; HIS-54-BFP_3/Pos0/img_000000000_Confocal-405_034.tif]

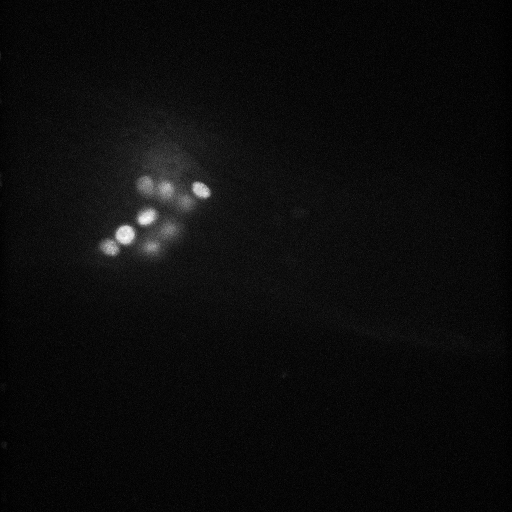

Supplement: Supplementary file 11 — Source data Fig. 2 [file 44318_2024_118_MOESM11_ESM.zip › Figure2/Figure 2A Micr. image/20201128 osm-3 G444E-gfp; HIS-54-BFP_3/Pos0/img_000000000_Confocal-405_035.tif]

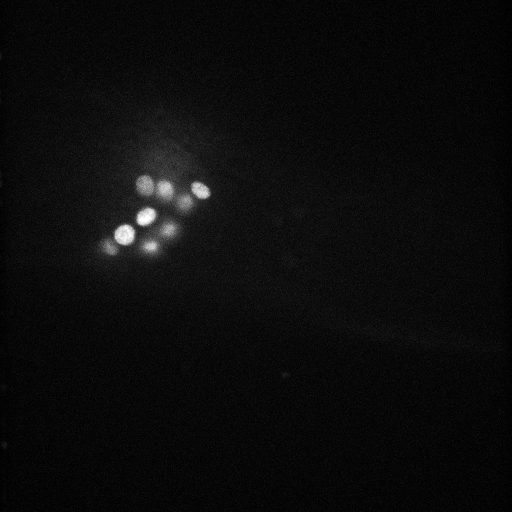

Supplement: Supplementary file 11 — Source data Fig. 2 [file 44318_2024_118_MOESM11_ESM.zip › Figure2/Figure 2A Micr. image/20201128 osm-3 G444E-gfp; HIS-54-BFP_3/Pos0/img_000000000_Confocal-405_036.tif]

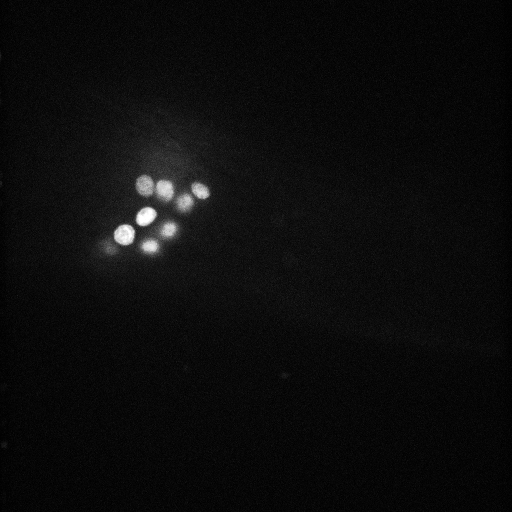

Supplement: Supplementary file 11 — Source data Fig. 2 [file 44318_2024_118_MOESM11_ESM.zip › Figure2/Figure 2A Micr. image/20201128 osm-3 G444E-gfp; HIS-54-BFP_3/Pos0/img_000000000_Confocal-405_037.tif]

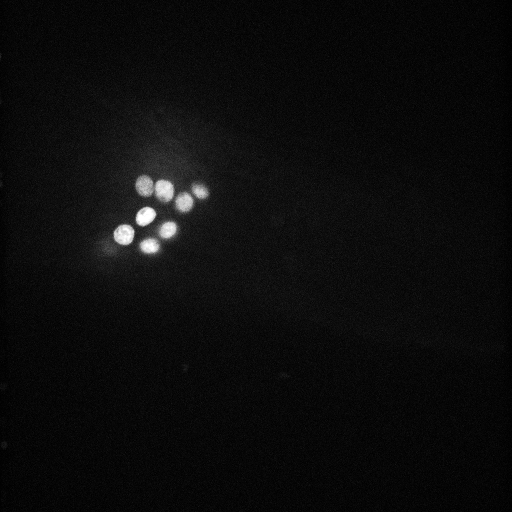

Supplement: Supplementary file 11 — Source data Fig. 2 [file 44318_2024_118_MOESM11_ESM.zip › Figure2/Figure 2A Micr. image/20201128 osm-3 G444E-gfp; HIS-54-BFP_3/Pos0/img_000000000_Confocal-405_038.tif]

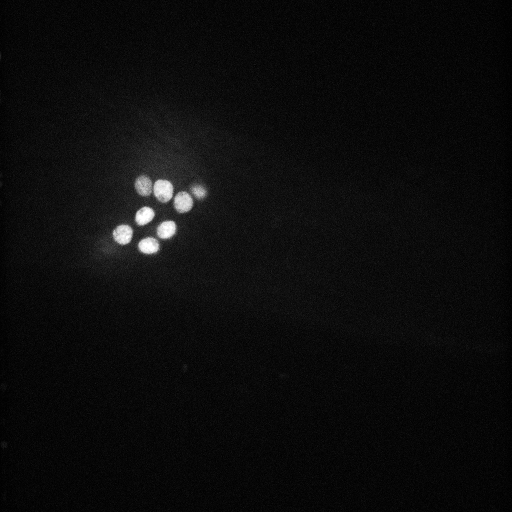

Supplement: Supplementary file 11 — Source data Fig. 2 [file 44318_2024_118_MOESM11_ESM.zip › Figure2/Figure 2A Micr. image/20201128 osm-3 G444E-gfp; HIS-54-BFP_3/Pos0/img_000000000_Confocal-405_039.tif]

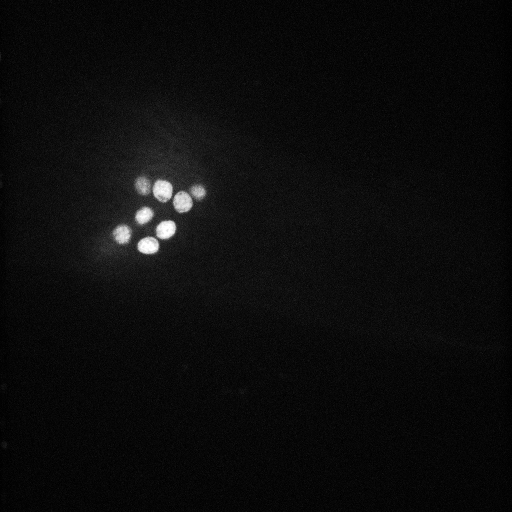

Supplement: Supplementary file 11 — Source data Fig. 2 [file 44318_2024_118_MOESM11_ESM.zip › Figure2/Figure 2A Micr. image/20201128 osm-3 G444E-gfp; HIS-54-BFP_3/Pos0/img_000000000_Confocal-405_040.tif]

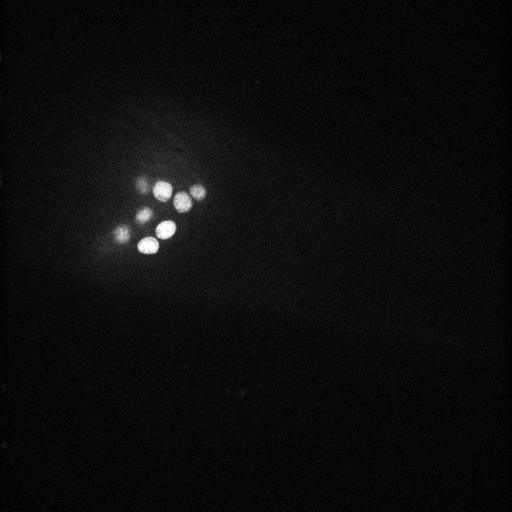

Supplement: Supplementary file 11 — Source data Fig. 2 [file 44318_2024_118_MOESM11_ESM.zip › Figure2/Figure 2A Micr. image/20201128 osm-3 G444E-gfp; HIS-54-BFP_3/Pos0/img_000000000_Confocal-405_041.tif]

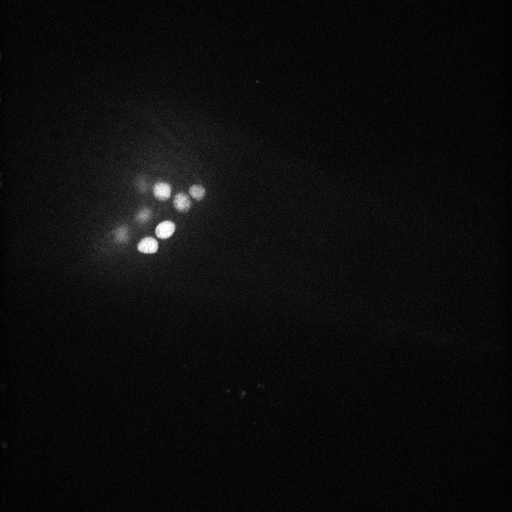

Supplement: Supplementary file 11 — Source data Fig. 2 [file 44318_2024_118_MOESM11_ESM.zip › Figure2/Figure 2A Micr. image/20201128 osm-3 G444E-gfp; HIS-54-BFP_3/Pos0/img_000000000_Confocal-405_042.tif]

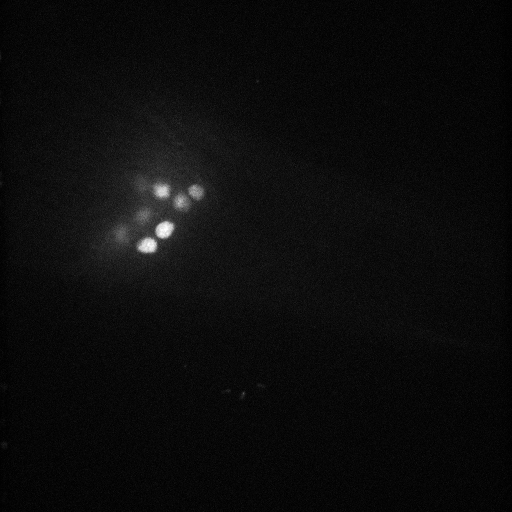

Supplement: Supplementary file 11 — Source data Fig. 2 [file 44318_2024_118_MOESM11_ESM.zip › Figure2/Figure 2A Micr. image/20201128 osm-3 G444E-gfp; HIS-54-BFP_3/Pos0/img_000000000_Confocal-405_043.tif]

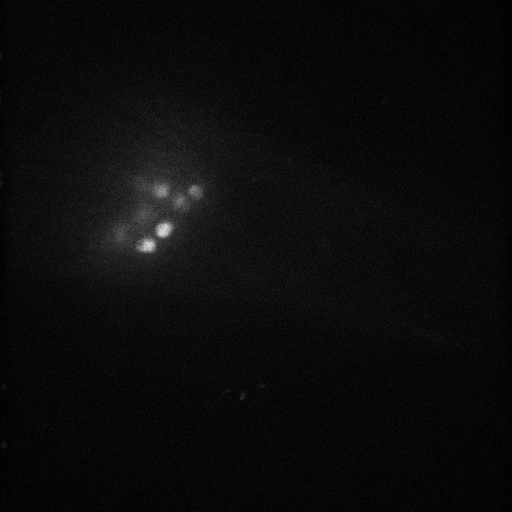

Supplement: Supplementary file 11 — Source data Fig. 2 [file 44318_2024_118_MOESM11_ESM.zip › Figure2/Figure 2A Micr. image/20201128 osm-3 G444E-gfp; HIS-54-BFP_3/Pos0/img_000000000_Confocal-405_044.tif]

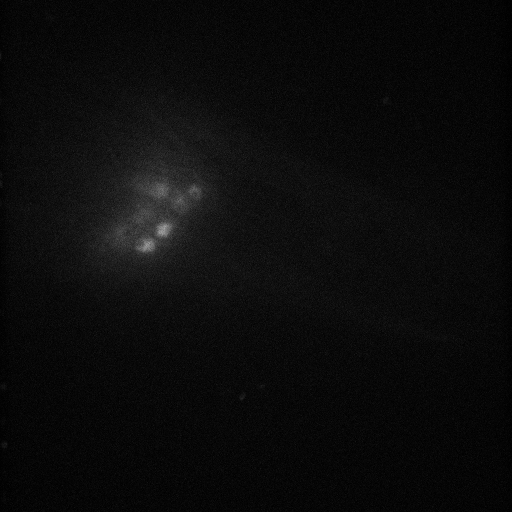

Supplement: Supplementary file 11 — Source data Fig. 2 [file 44318_2024_118_MOESM11_ESM.zip › Figure2/Figure 2A Micr. image/20201128 osm-3 G444E-gfp; HIS-54-BFP_3/Pos0/img_000000000_Confocal-405_045.tif]

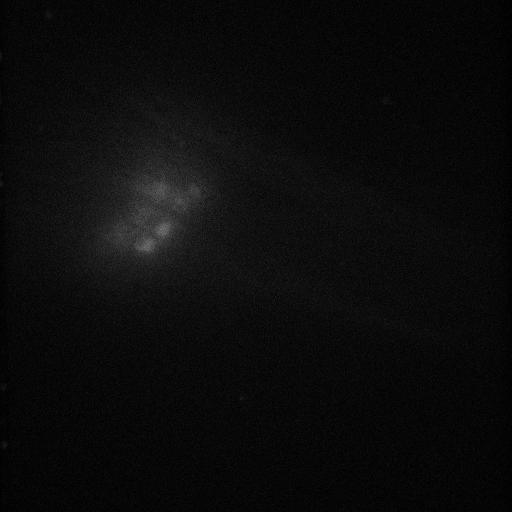

Supplement: Supplementary file 11 — Source data Fig. 2 [file 44318_2024_118_MOESM11_ESM.zip › Figure2/Figure 2A Micr. image/20201128 osm-3 G444E-gfp; HIS-54-BFP_3/Pos0/img_000000000_Confocal-405_046.tif]

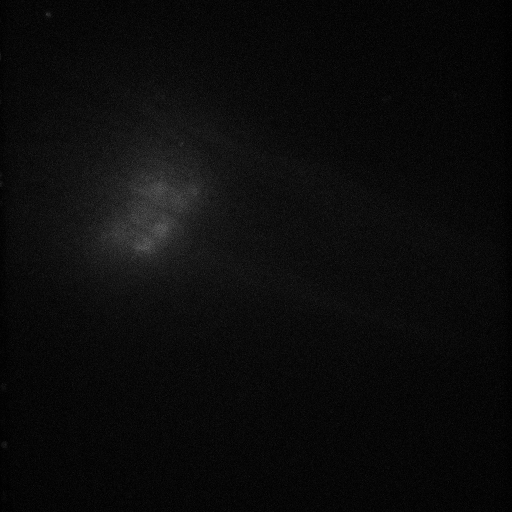

Supplement: Supplementary file 11 — Source data Fig. 2 [file 44318_2024_118_MOESM11_ESM.zip › Figure2/Figure 2A Micr. image/20201128 osm-3 G444E-gfp; HIS-54-BFP_3/Pos0/img_000000000_Confocal-405_047.tif]

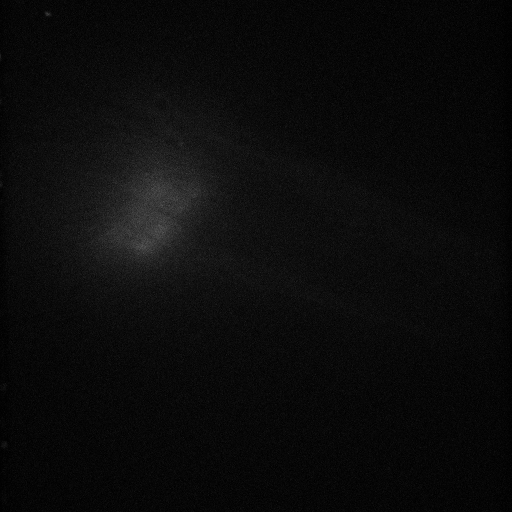

Supplement: Supplementary file 11 — Source data Fig. 2 [file 44318_2024_118_MOESM11_ESM.zip › Figure2/Figure 2A Micr. image/20201128 osm-3 G444E-gfp; HIS-54-BFP_3/Pos0/img_000000000_Confocal-405_048.tif]

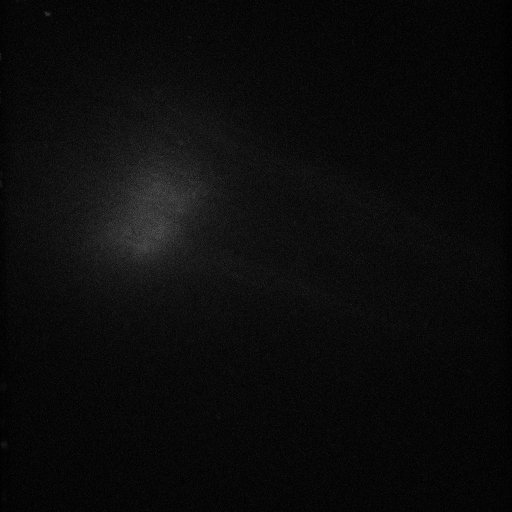

Supplement: Supplementary file 11 — Source data Fig. 2 [file 44318_2024_118_MOESM11_ESM.zip › Figure2/Figure 2A Micr. image/20201128 osm-3 G444E-gfp; HIS-54-BFP_3/Pos0/img_000000000_Confocal-405_049.tif]

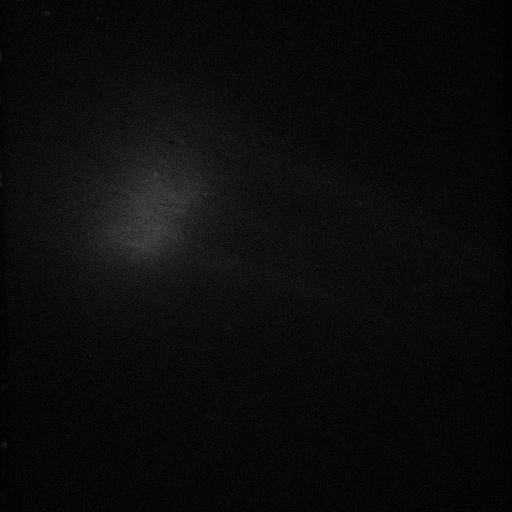

Supplement: Supplementary file 11 — Source data Fig. 2 [file 44318_2024_118_MOESM11_ESM.zip › Figure2/Figure 2A Micr. image/20201128 osm-3 G444E-gfp; HIS-54-BFP_3/Pos0/img_000000000_Confocal-405_050.tif]

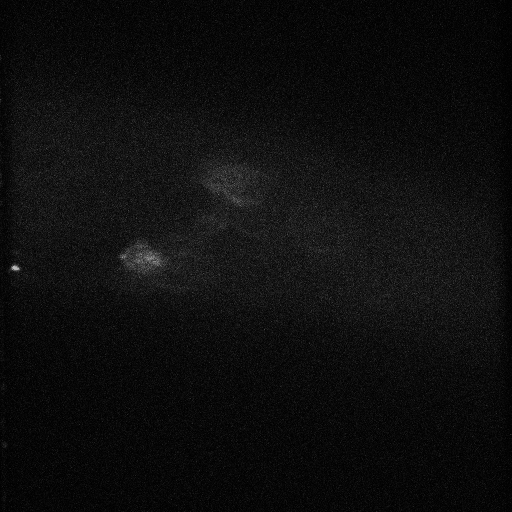

Supplement: Supplementary file 11 — Source data Fig. 2 [file 44318_2024_118_MOESM11_ESM.zip › Figure2/Figure 2A Micr. image/20201128 osm-3 G444E-gfp; HIS-54-BFP_3/Pos0/img_000000000_Confocal-488-Acq_000.tif]

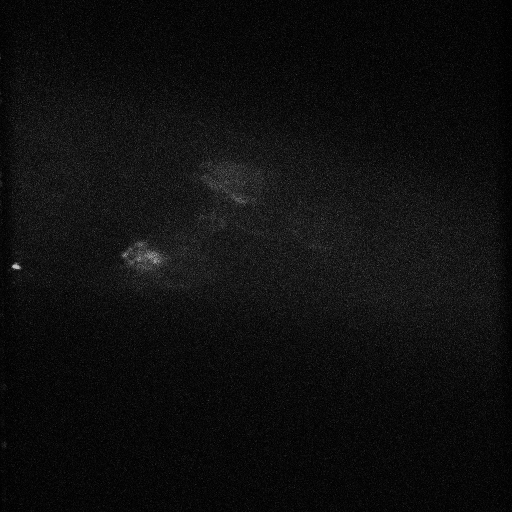

Supplement: Supplementary file 11 — Source data Fig. 2 [file 44318_2024_118_MOESM11_ESM.zip › Figure2/Figure 2A Micr. image/20201128 osm-3 G444E-gfp; HIS-54-BFP_3/Pos0/img_000000000_Confocal-488-Acq_001.tif]

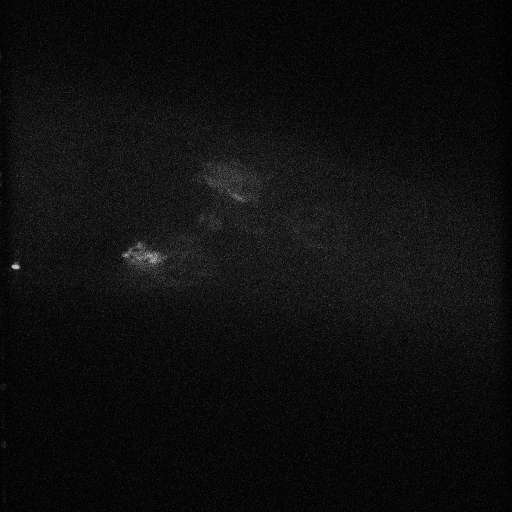

Supplement: Supplementary file 11 — Source data Fig. 2 [file 44318_2024_118_MOESM11_ESM.zip › Figure2/Figure 2A Micr. image/20201128 osm-3 G444E-gfp; HIS-54-BFP_3/Pos0/img_000000000_Confocal-488-Acq_002.tif]

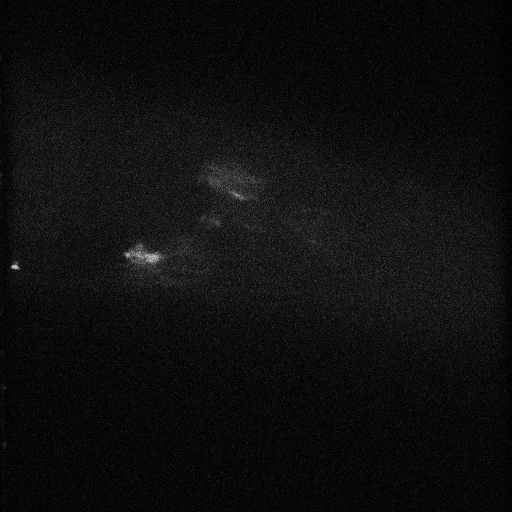

Supplement: Supplementary file 11 — Source data Fig. 2 [file 44318_2024_118_MOESM11_ESM.zip › Figure2/Figure 2A Micr. image/20201128 osm-3 G444E-gfp; HIS-54-BFP_3/Pos0/img_000000000_Confocal-488-Acq_003.tif]

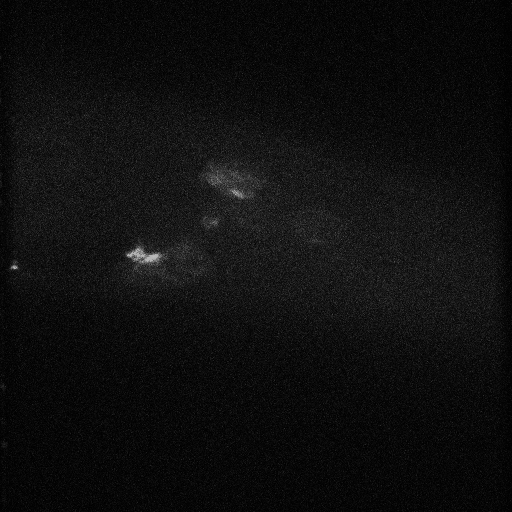

Supplement: Supplementary file 11 — Source data Fig. 2 [file 44318_2024_118_MOESM11_ESM.zip › Figure2/Figure 2A Micr. image/20201128 osm-3 G444E-gfp; HIS-54-BFP_3/Pos0/img_000000000_Confocal-488-Acq_004.tif]

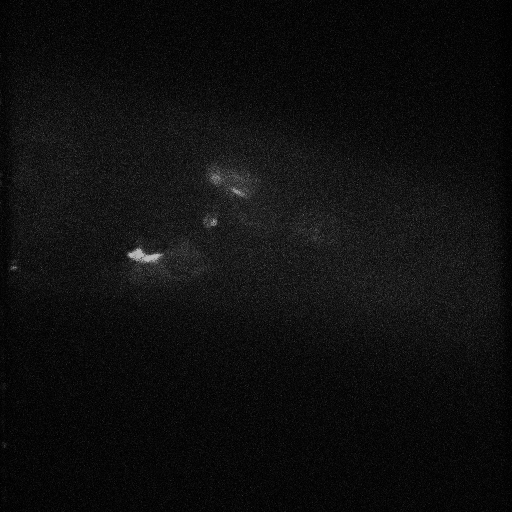

Supplement: Supplementary file 11 — Source data Fig. 2 [file 44318_2024_118_MOESM11_ESM.zip › Figure2/Figure 2A Micr. image/20201128 osm-3 G444E-gfp; HIS-54-BFP_3/Pos0/img_000000000_Confocal-488-Acq_005.tif]

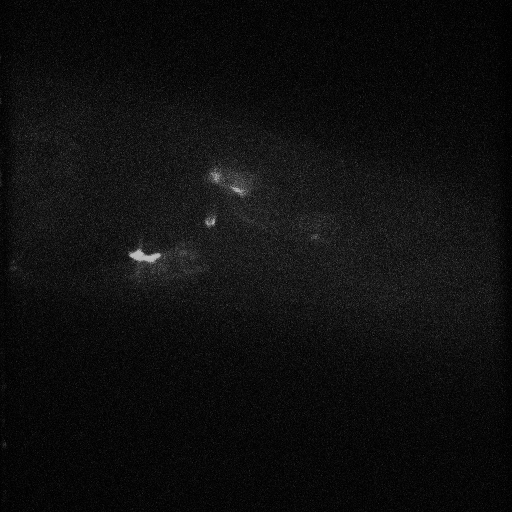

Supplement: Supplementary file 11 — Source data Fig. 2 [file 44318_2024_118_MOESM11_ESM.zip › Figure2/Figure 2A Micr. image/20201128 osm-3 G444E-gfp; HIS-54-BFP_3/Pos0/img_000000000_Confocal-488-Acq_006.tif]

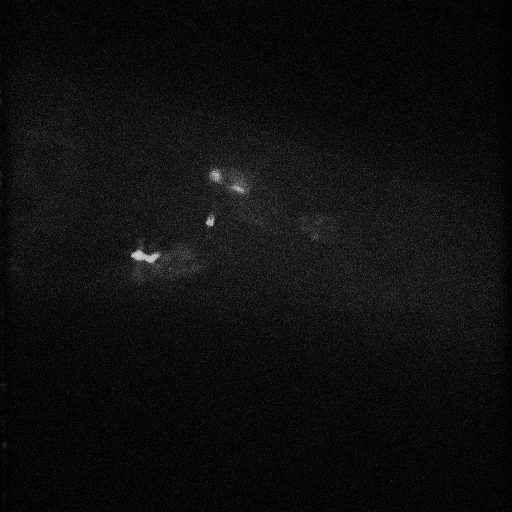

Supplement: Supplementary file 11 — Source data Fig. 2 [file 44318_2024_118_MOESM11_ESM.zip › Figure2/Figure 2A Micr. image/20201128 osm-3 G444E-gfp; HIS-54-BFP_3/Pos0/img_000000000_Confocal-488-Acq_007.tif]

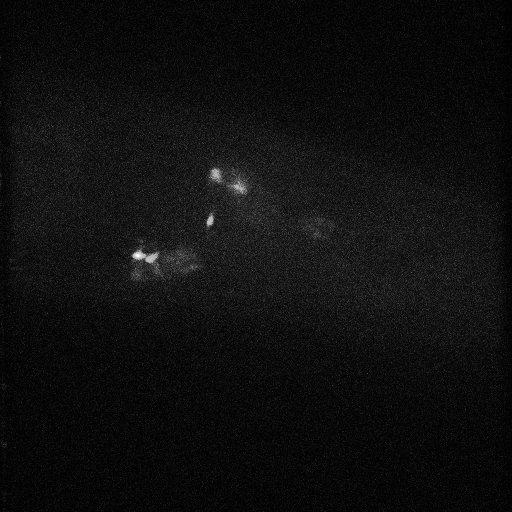

Supplement: Supplementary file 11 — Source data Fig. 2 [file 44318_2024_118_MOESM11_ESM.zip › Figure2/Figure 2A Micr. image/20201128 osm-3 G444E-gfp; HIS-54-BFP_3/Pos0/img_000000000_Confocal-488-Acq_008.tif]

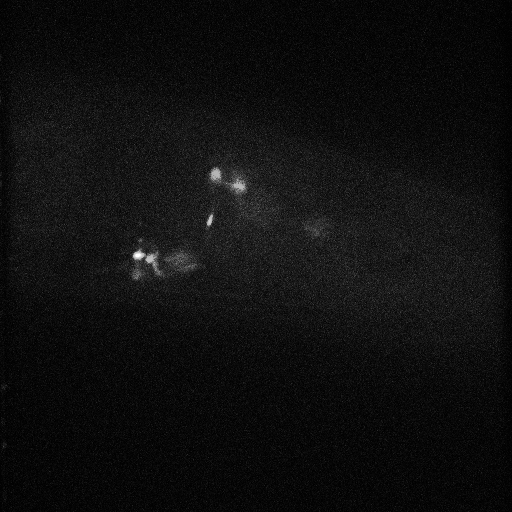

Supplement: Supplementary file 11 — Source data Fig. 2 [file 44318_2024_118_MOESM11_ESM.zip › Figure2/Figure 2A Micr. image/20201128 osm-3 G444E-gfp; HIS-54-BFP_3/Pos0/img_000000000_Confocal-488-Acq_009.tif]

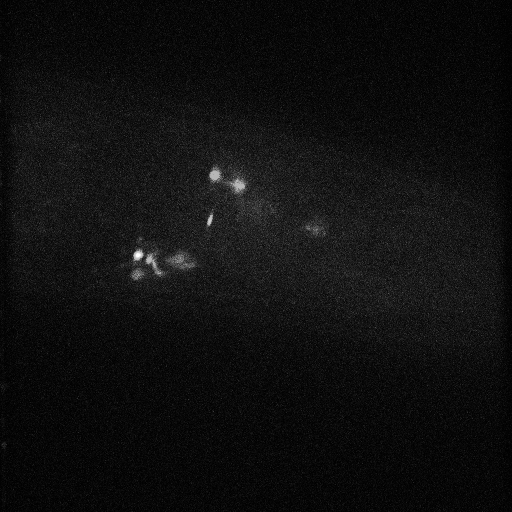

Supplement: Supplementary file 11 — Source data Fig. 2 [file 44318_2024_118_MOESM11_ESM.zip › Figure2/Figure 2A Micr. image/20201128 osm-3 G444E-gfp; HIS-54-BFP_3/Pos0/img_000000000_Confocal-488-Acq_010.tif]

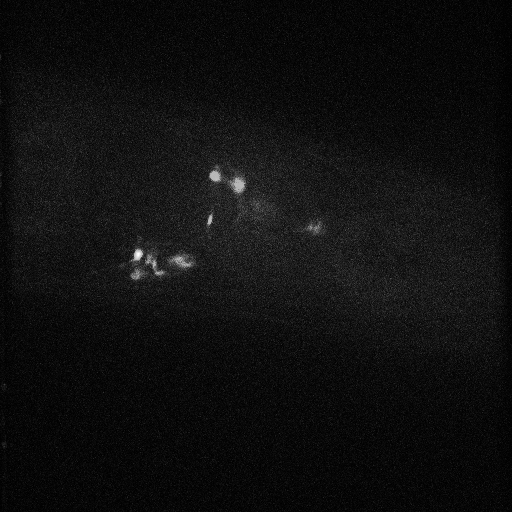

Supplement: Supplementary file 11 — Source data Fig. 2 [file 44318_2024_118_MOESM11_ESM.zip › Figure2/Figure 2A Micr. image/20201128 osm-3 G444E-gfp; HIS-54-BFP_3/Pos0/img_000000000_Confocal-488-Acq_011.tif]

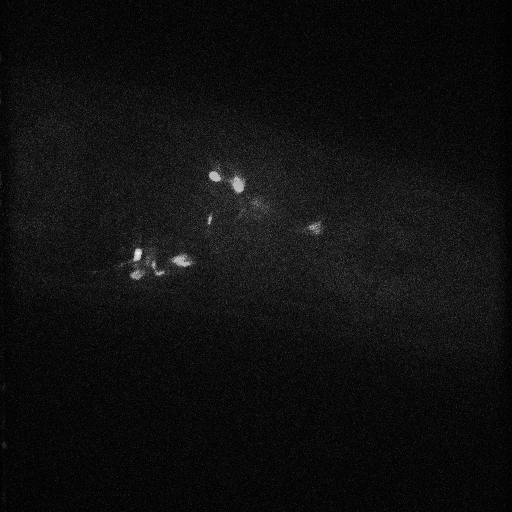

Supplement: Supplementary file 11 — Source data Fig. 2 [file 44318_2024_118_MOESM11_ESM.zip › Figure2/Figure 2A Micr. image/20201128 osm-3 G444E-gfp; HIS-54-BFP_3/Pos0/img_000000000_Confocal-488-Acq_012.tif]

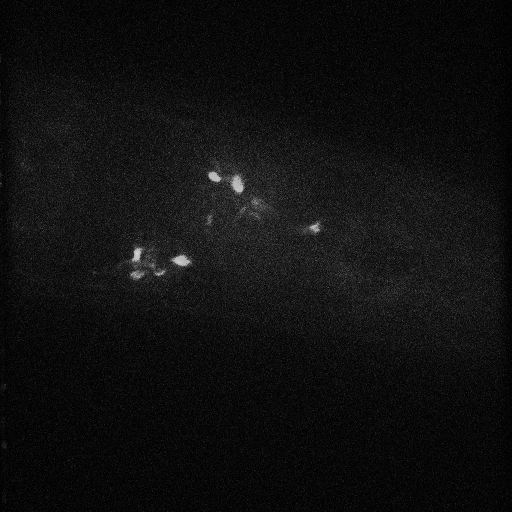

Supplement: Supplementary file 11 — Source data Fig. 2 [file 44318_2024_118_MOESM11_ESM.zip › Figure2/Figure 2A Micr. image/20201128 osm-3 G444E-gfp; HIS-54-BFP_3/Pos0/img_000000000_Confocal-488-Acq_013.tif]

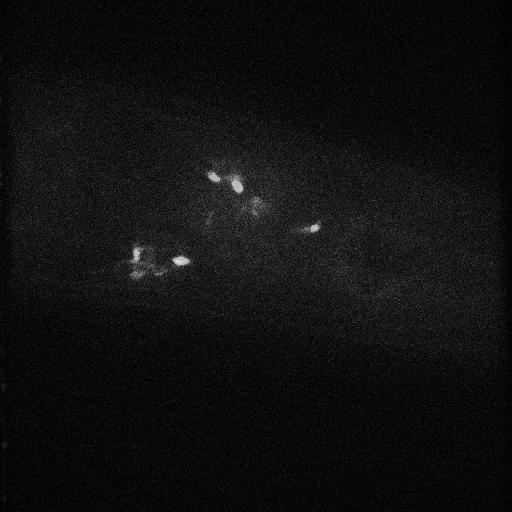

Supplement: Supplementary file 11 — Source data Fig. 2 [file 44318_2024_118_MOESM11_ESM.zip › Figure2/Figure 2A Micr. image/20201128 osm-3 G444E-gfp; HIS-54-BFP_3/Pos0/img_000000000_Confocal-488-Acq_014.tif]

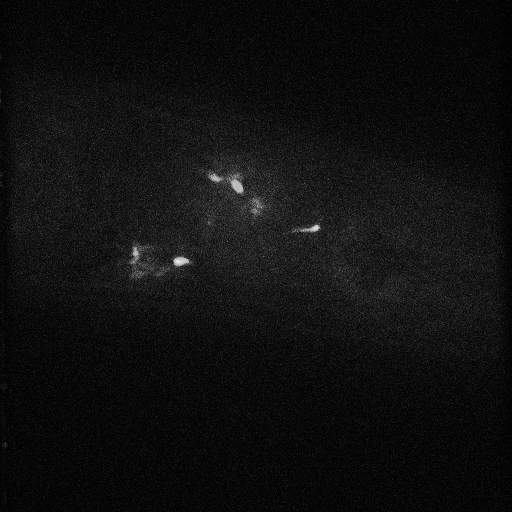

Supplement: Supplementary file 11 — Source data Fig. 2 [file 44318_2024_118_MOESM11_ESM.zip › Figure2/Figure 2A Micr. image/20201128 osm-3 G444E-gfp; HIS-54-BFP_3/Pos0/img_000000000_Confocal-488-Acq_015.tif]

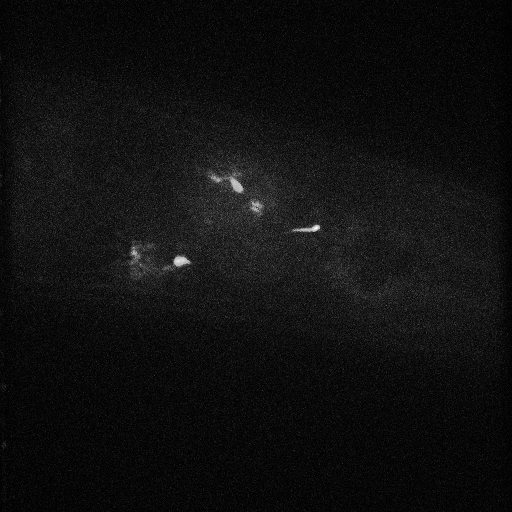

Supplement: Supplementary file 11 — Source data Fig. 2 [file 44318_2024_118_MOESM11_ESM.zip › Figure2/Figure 2A Micr. image/20201128 osm-3 G444E-gfp; HIS-54-BFP_3/Pos0/img_000000000_Confocal-488-Acq_016.tif]

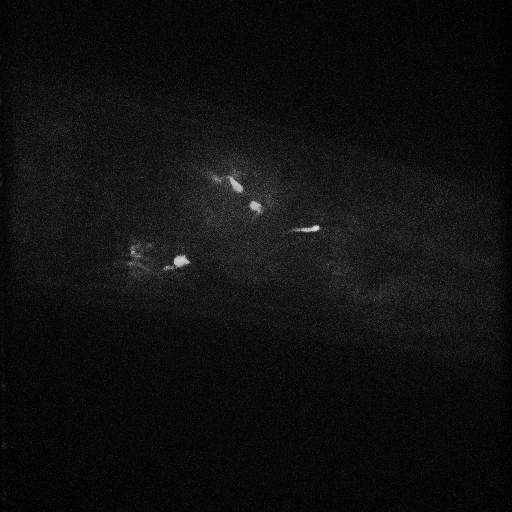

Supplement: Supplementary file 11 — Source data Fig. 2 [file 44318_2024_118_MOESM11_ESM.zip › Figure2/Figure 2A Micr. image/20201128 osm-3 G444E-gfp; HIS-54-BFP_3/Pos0/img_000000000_Confocal-488-Acq_017.tif]

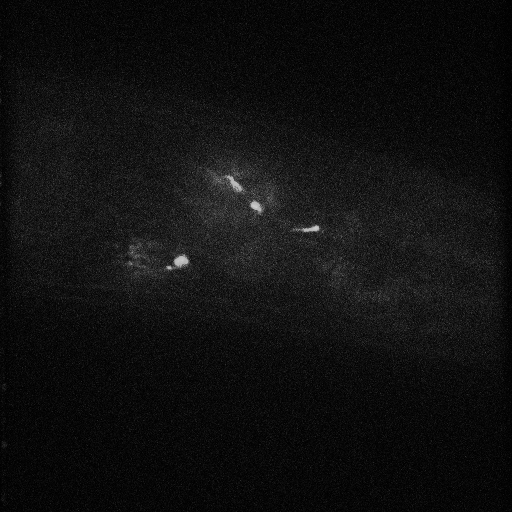

Supplement: Supplementary file 11 — Source data Fig. 2 [file 44318_2024_118_MOESM11_ESM.zip › Figure2/Figure 2A Micr. image/20201128 osm-3 G444E-gfp; HIS-54-BFP_3/Pos0/img_000000000_Confocal-488-Acq_018.tif]

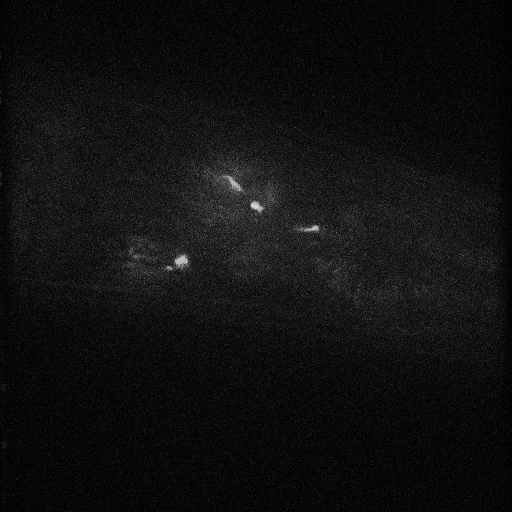

Supplement: Supplementary file 11 — Source data Fig. 2 [file 44318_2024_118_MOESM11_ESM.zip › Figure2/Figure 2A Micr. image/20201128 osm-3 G444E-gfp; HIS-54-BFP_3/Pos0/img_000000000_Confocal-488-Acq_019.tif]

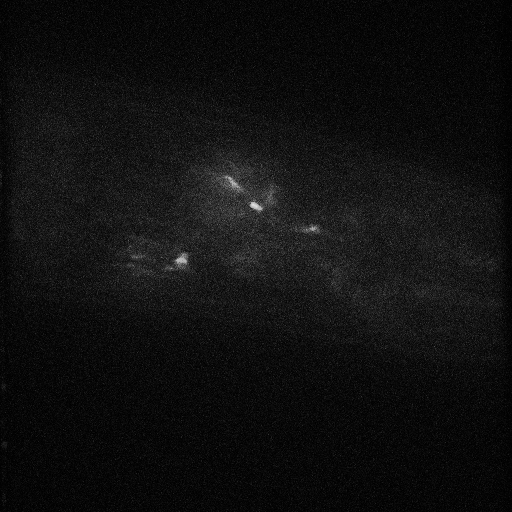

Supplement: Supplementary file 11 — Source data Fig. 2 [file 44318_2024_118_MOESM11_ESM.zip › Figure2/Figure 2A Micr. image/20201128 osm-3 G444E-gfp; HIS-54-BFP_3/Pos0/img_000000000_Confocal-488-Acq_020.tif]

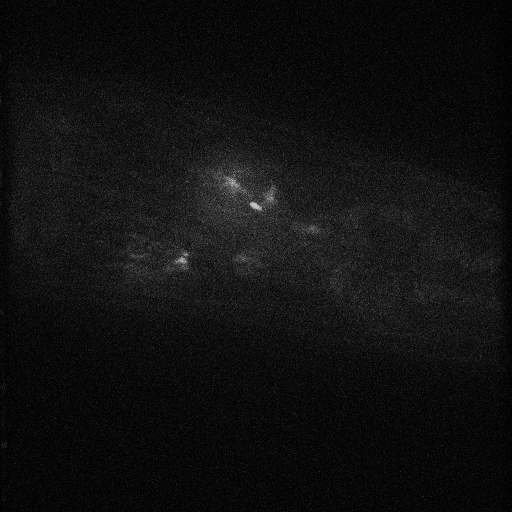

Supplement: Supplementary file 11 — Source data Fig. 2 [file 44318_2024_118_MOESM11_ESM.zip › Figure2/Figure 2A Micr. image/20201128 osm-3 G444E-gfp; HIS-54-BFP_3/Pos0/img_000000000_Confocal-488-Acq_021.tif]

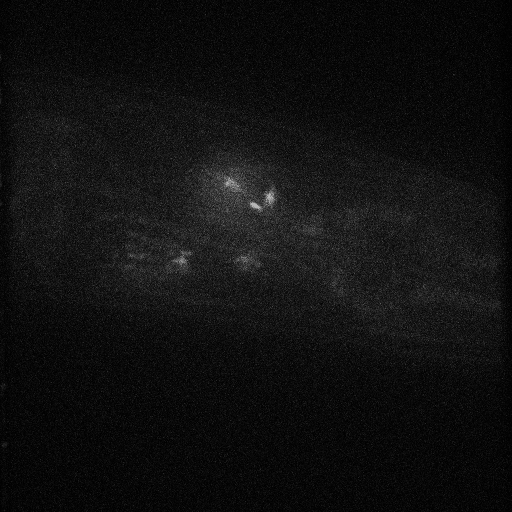

Supplement: Supplementary file 11 — Source data Fig. 2 [file 44318_2024_118_MOESM11_ESM.zip › Figure2/Figure 2A Micr. image/20201128 osm-3 G444E-gfp; HIS-54-BFP_3/Pos0/img_000000000_Confocal-488-Acq_022.tif]

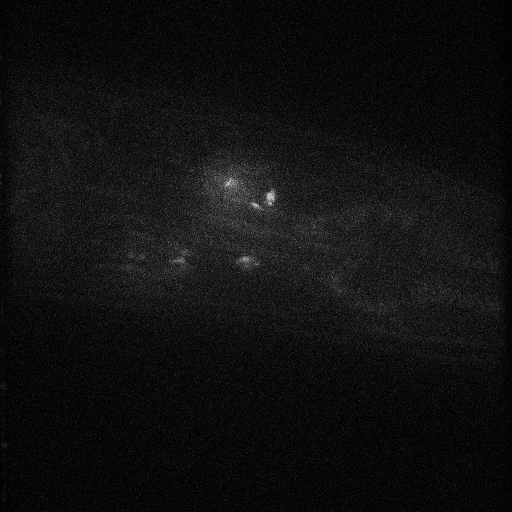

Supplement: Supplementary file 11 — Source data Fig. 2 [file 44318_2024_118_MOESM11_ESM.zip › Figure2/Figure 2A Micr. image/20201128 osm-3 G444E-gfp; HIS-54-BFP_3/Pos0/img_000000000_Confocal-488-Acq_023.tif]

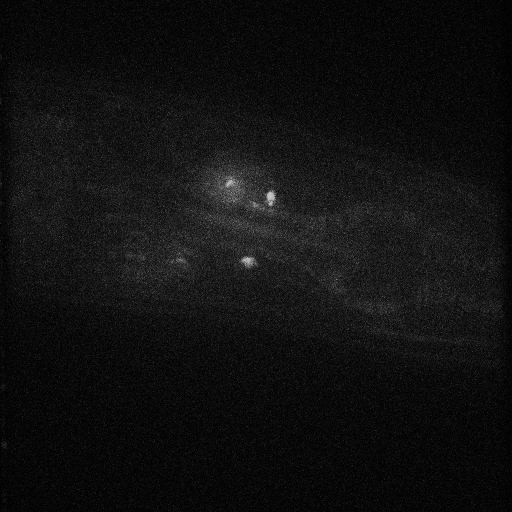

Supplement: Supplementary file 11 — Source data Fig. 2 [file 44318_2024_118_MOESM11_ESM.zip › Figure2/Figure 2A Micr. image/20201128 osm-3 G444E-gfp; HIS-54-BFP_3/Pos0/img_000000000_Confocal-488-Acq_024.tif]

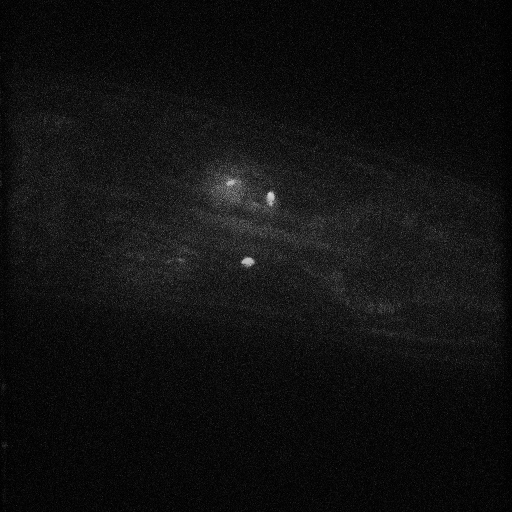

Supplement: Supplementary file 11 — Source data Fig. 2 [file 44318_2024_118_MOESM11_ESM.zip › Figure2/Figure 2A Micr. image/20201128 osm-3 G444E-gfp; HIS-54-BFP_3/Pos0/img_000000000_Confocal-488-Acq_025.tif]

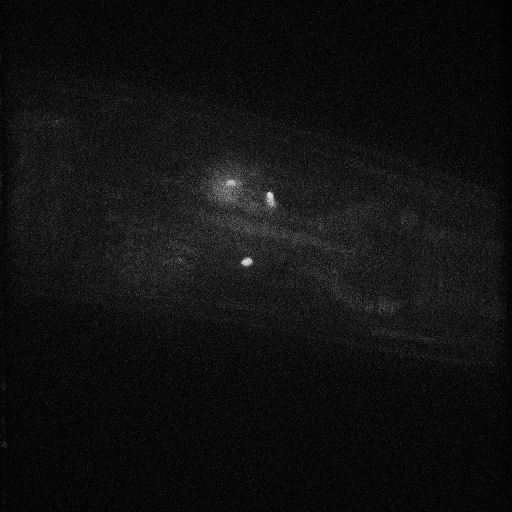

Supplement: Supplementary file 11 — Source data Fig. 2 [file 44318_2024_118_MOESM11_ESM.zip › Figure2/Figure 2A Micr. image/20201128 osm-3 G444E-gfp; HIS-54-BFP_3/Pos0/img_000000000_Confocal-488-Acq_026.tif]

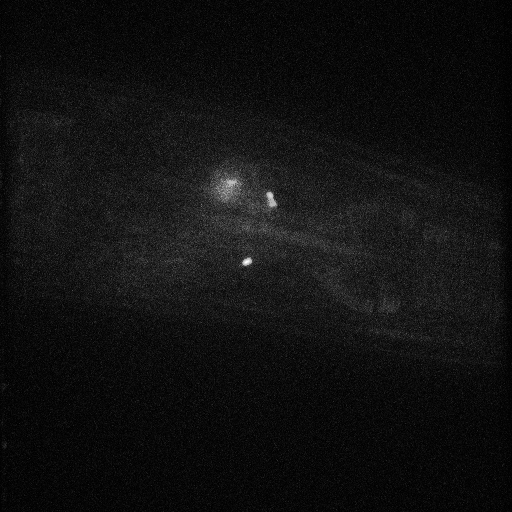

Supplement: Supplementary file 11 — Source data Fig. 2 [file 44318_2024_118_MOESM11_ESM.zip › Figure2/Figure 2A Micr. image/20201128 osm-3 G444E-gfp; HIS-54-BFP_3/Pos0/img_000000000_Confocal-488-Acq_027.tif]

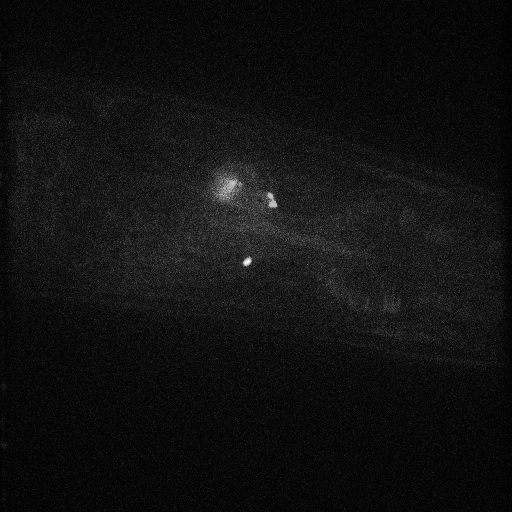

Supplement: Supplementary file 11 — Source data Fig. 2 [file 44318_2024_118_MOESM11_ESM.zip › Figure2/Figure 2A Micr. image/20201128 osm-3 G444E-gfp; HIS-54-BFP_3/Pos0/img_000000000_Confocal-488-Acq_028.tif]

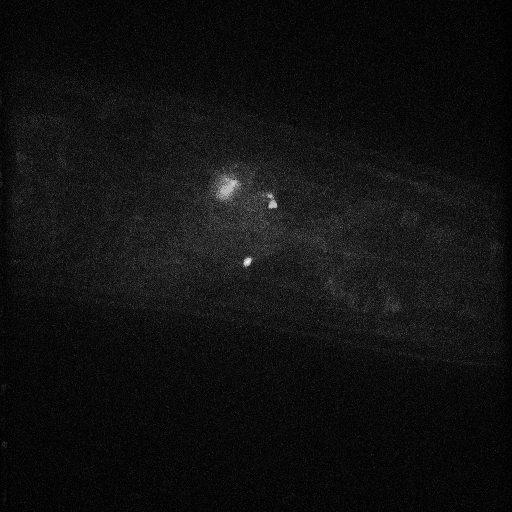

Supplement: Supplementary file 11 — Source data Fig. 2 [file 44318_2024_118_MOESM11_ESM.zip › Figure2/Figure 2A Micr. image/20201128 osm-3 G444E-gfp; HIS-54-BFP_3/Pos0/img_000000000_Confocal-488-Acq_029.tif]

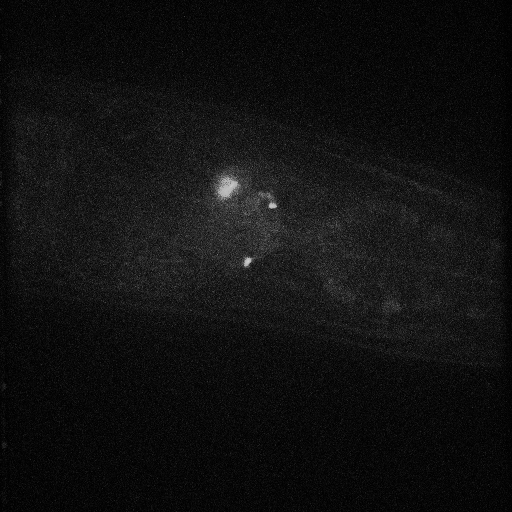

Supplement: Supplementary file 11 — Source data Fig. 2 [file 44318_2024_118_MOESM11_ESM.zip › Figure2/Figure 2A Micr. image/20201128 osm-3 G444E-gfp; HIS-54-BFP_3/Pos0/img_000000000_Confocal-488-Acq_030.tif]

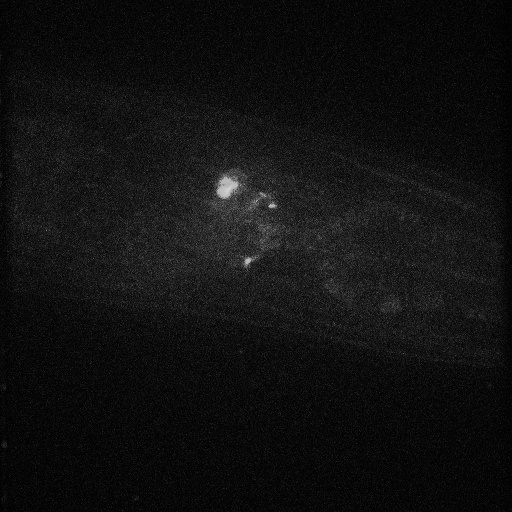

Supplement: Supplementary file 11 — Source data Fig. 2 [file 44318_2024_118_MOESM11_ESM.zip › Figure2/Figure 2A Micr. image/20201128 osm-3 G444E-gfp; HIS-54-BFP_3/Pos0/img_000000000_Confocal-488-Acq_031.tif]

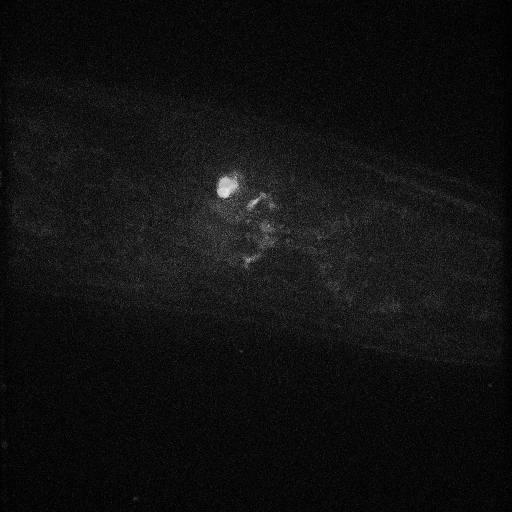

Supplement: Supplementary file 11 — Source data Fig. 2 [file 44318_2024_118_MOESM11_ESM.zip › Figure2/Figure 2A Micr. image/20201128 osm-3 G444E-gfp; HIS-54-BFP_3/Pos0/img_000000000_Confocal-488-Acq_032.tif]

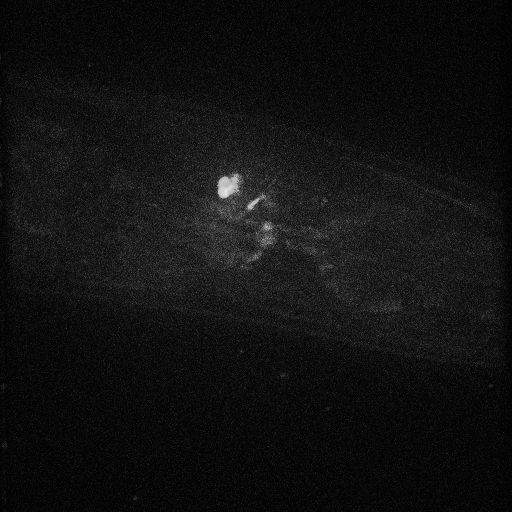

Supplement: Supplementary file 11 — Source data Fig. 2 [file 44318_2024_118_MOESM11_ESM.zip › Figure2/Figure 2A Micr. image/20201128 osm-3 G444E-gfp; HIS-54-BFP_3/Pos0/img_000000000_Confocal-488-Acq_033.tif]

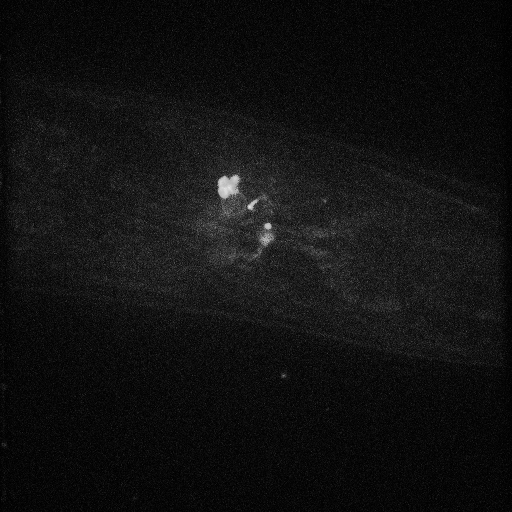

Supplement: Supplementary file 11 — Source data Fig. 2 [file 44318_2024_118_MOESM11_ESM.zip › Figure2/Figure 2A Micr. image/20201128 osm-3 G444E-gfp; HIS-54-BFP_3/Pos0/img_000000000_Confocal-488-Acq_034.tif]

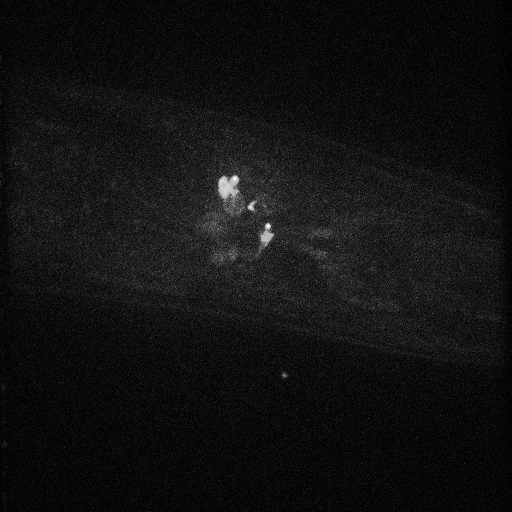

Supplement: Supplementary file 11 — Source data Fig. 2 [file 44318_2024_118_MOESM11_ESM.zip › Figure2/Figure 2A Micr. image/20201128 osm-3 G444E-gfp; HIS-54-BFP_3/Pos0/img_000000000_Confocal-488-Acq_035.tif]

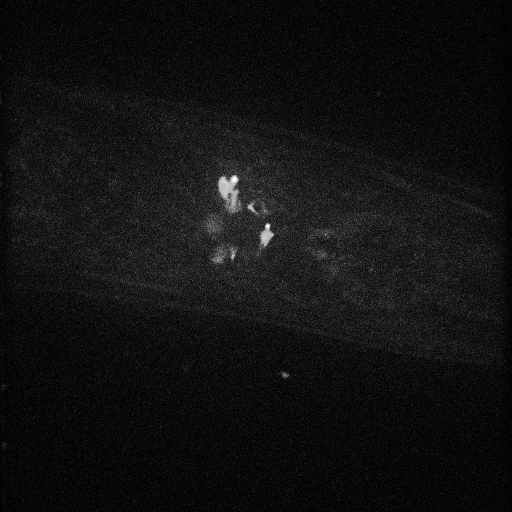

Supplement: Supplementary file 11 — Source data Fig. 2 [file 44318_2024_118_MOESM11_ESM.zip › Figure2/Figure 2A Micr. image/20201128 osm-3 G444E-gfp; HIS-54-BFP_3/Pos0/img_000000000_Confocal-488-Acq_036.tif]

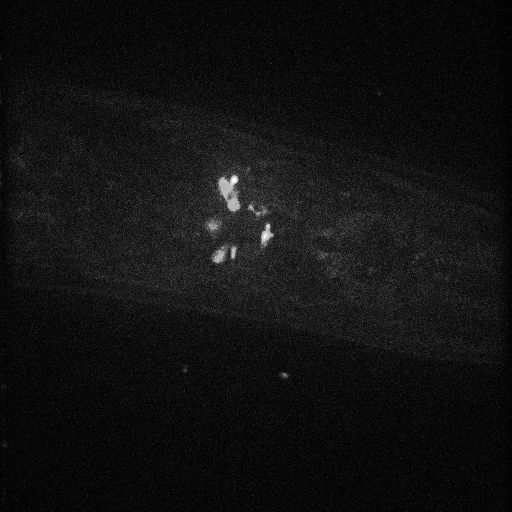

Supplement: Supplementary file 11 — Source data Fig. 2 [file 44318_2024_118_MOESM11_ESM.zip › Figure2/Figure 2A Micr. image/20201128 osm-3 G444E-gfp; HIS-54-BFP_3/Pos0/img_000000000_Confocal-488-Acq_037.tif]

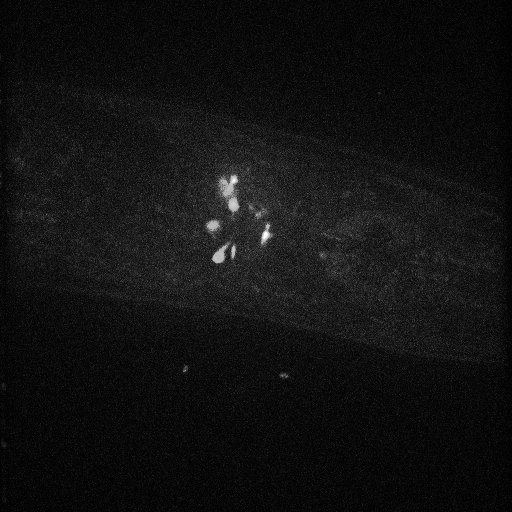

Supplement: Supplementary file 11 — Source data Fig. 2 [file 44318_2024_118_MOESM11_ESM.zip › Figure2/Figure 2A Micr. image/20201128 osm-3 G444E-gfp; HIS-54-BFP_3/Pos0/img_000000000_Confocal-488-Acq_038.tif]

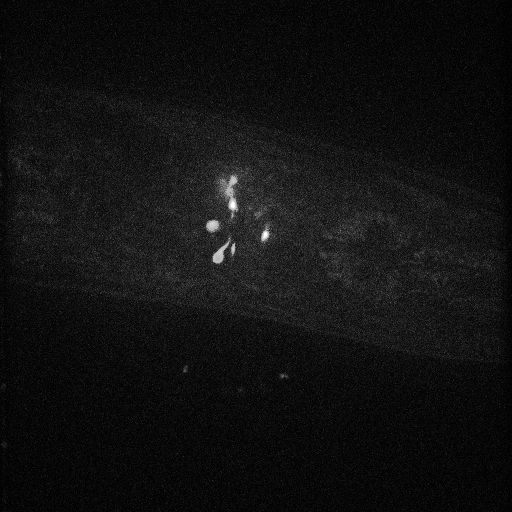

Supplement: Supplementary file 11 — Source data Fig. 2 [file 44318_2024_118_MOESM11_ESM.zip › Figure2/Figure 2A Micr. image/20201128 osm-3 G444E-gfp; HIS-54-BFP_3/Pos0/img_000000000_Confocal-488-Acq_039.tif]

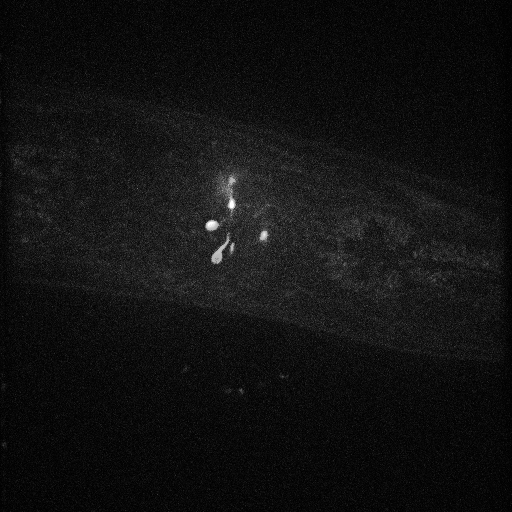

Supplement: Supplementary file 11 — Source data Fig. 2 [file 44318_2024_118_MOESM11_ESM.zip › Figure2/Figure 2A Micr. image/20201128 osm-3 G444E-gfp; HIS-54-BFP_3/Pos0/img_000000000_Confocal-488-Acq_040.tif]

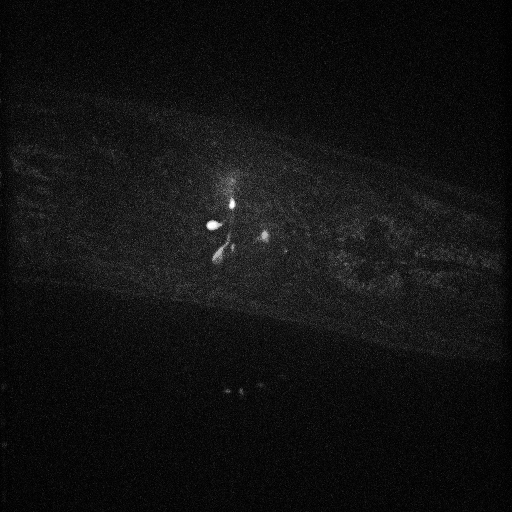

Supplement: Supplementary file 11 — Source data Fig. 2 [file 44318_2024_118_MOESM11_ESM.zip › Figure2/Figure 2A Micr. image/20201128 osm-3 G444E-gfp; HIS-54-BFP_3/Pos0/img_000000000_Confocal-488-Acq_041.tif]

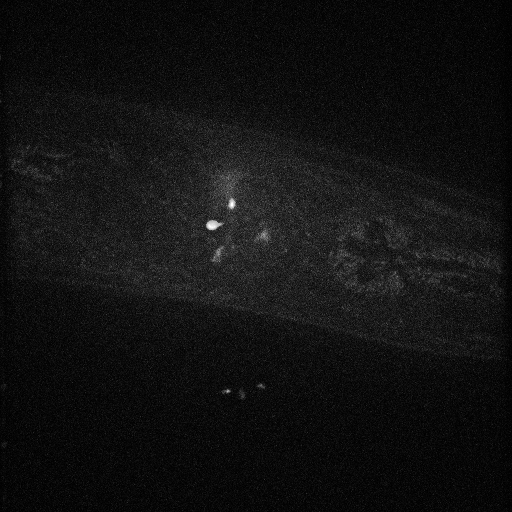

Supplement: Supplementary file 11 — Source data Fig. 2 [file 44318_2024_118_MOESM11_ESM.zip › Figure2/Figure 2A Micr. image/20201128 osm-3 G444E-gfp; HIS-54-BFP_3/Pos0/img_000000000_Confocal-488-Acq_042.tif]

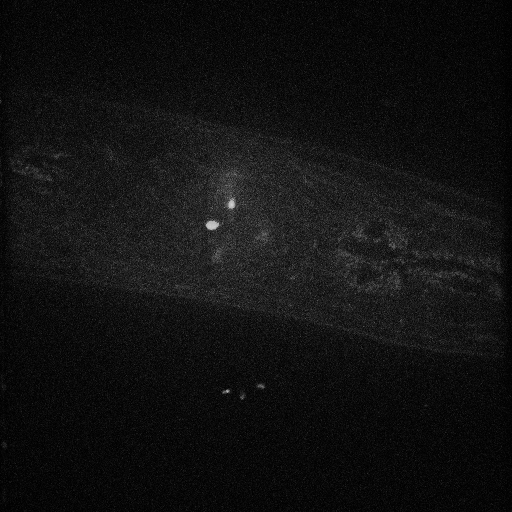

Supplement: Supplementary file 11 — Source data Fig. 2 [file 44318_2024_118_MOESM11_ESM.zip › Figure2/Figure 2A Micr. image/20201128 osm-3 G444E-gfp; HIS-54-BFP_3/Pos0/img_000000000_Confocal-488-Acq_043.tif]

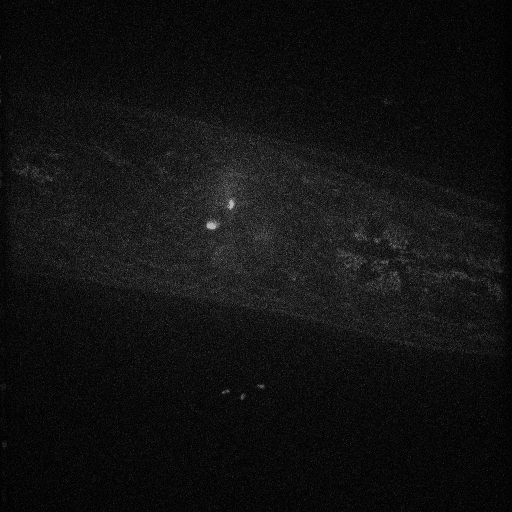

Supplement: Supplementary file 11 — Source data Fig. 2 [file 44318_2024_118_MOESM11_ESM.zip › Figure2/Figure 2A Micr. image/20201128 osm-3 G444E-gfp; HIS-54-BFP_3/Pos0/img_000000000_Confocal-488-Acq_044.tif]

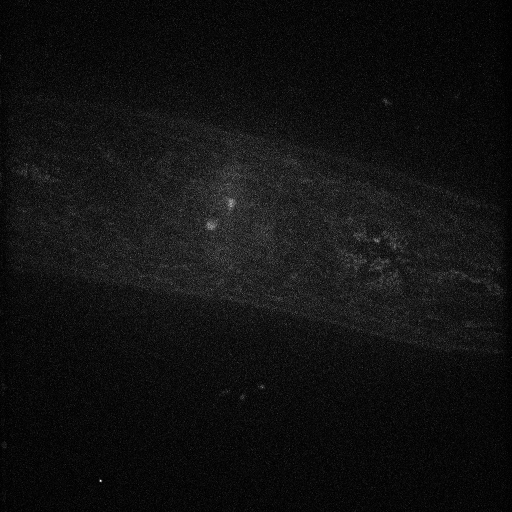

Supplement: Supplementary file 11 — Source data Fig. 2 [file 44318_2024_118_MOESM11_ESM.zip › Figure2/Figure 2A Micr. image/20201128 osm-3 G444E-gfp; HIS-54-BFP_3/Pos0/img_000000000_Confocal-488-Acq_045.tif]

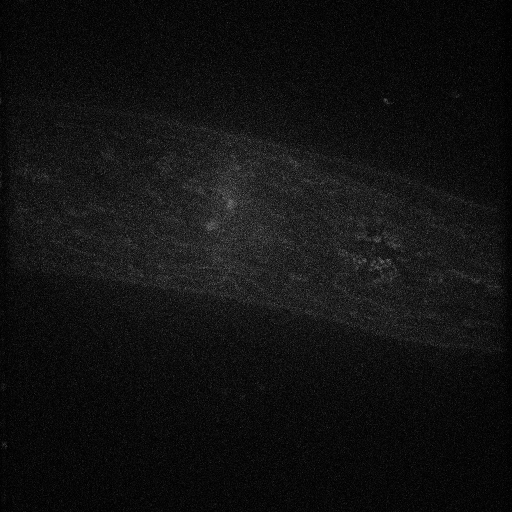

Supplement: Supplementary file 11 — Source data Fig. 2 [file 44318_2024_118_MOESM11_ESM.zip › Figure2/Figure 2A Micr. image/20201128 osm-3 G444E-gfp; HIS-54-BFP_3/Pos0/img_000000000_Confocal-488-Acq_046.tif]

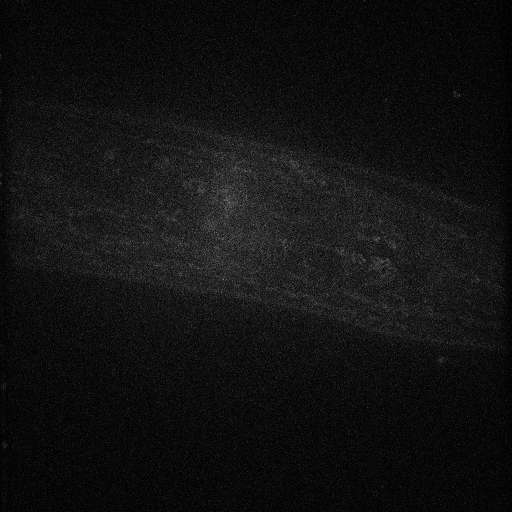

Supplement: Supplementary file 11 — Source data Fig. 2 [file 44318_2024_118_MOESM11_ESM.zip › Figure2/Figure 2A Micr. image/20201128 osm-3 G444E-gfp; HIS-54-BFP_3/Pos0/img_000000000_Confocal-488-Acq_047.tif]

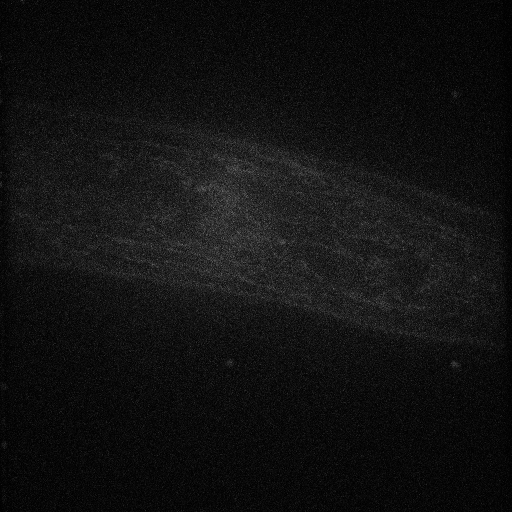

Supplement: Supplementary file 11 — Source data Fig. 2 [file 44318_2024_118_MOESM11_ESM.zip › Figure2/Figure 2A Micr. image/20201128 osm-3 G444E-gfp; HIS-54-BFP_3/Pos0/img_000000000_Confocal-488-Acq_048.tif]

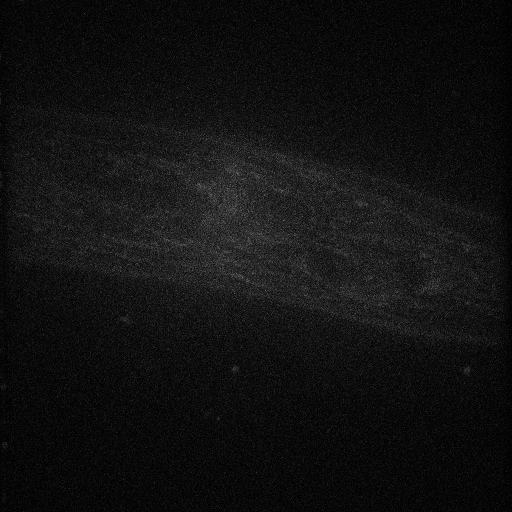

Supplement: Supplementary file 11 — Source data Fig. 2 [file 44318_2024_118_MOESM11_ESM.zip › Figure2/Figure 2A Micr. image/20201128 osm-3 G444E-gfp; HIS-54-BFP_3/Pos0/img_000000000_Confocal-488-Acq_049.tif]

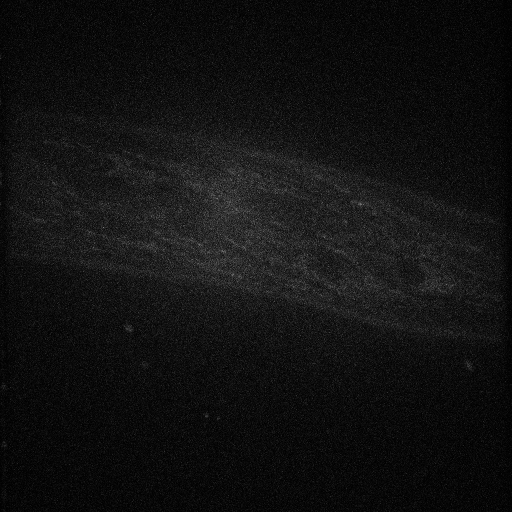

Supplement: Supplementary file 11 — Source data Fig. 2 [file 44318_2024_118_MOESM11_ESM.zip › Figure2/Figure 2A Micr. image/20201128 osm-3 G444E-gfp; HIS-54-BFP_3/Pos0/img_000000000_Confocal-488-Acq_050.tif]

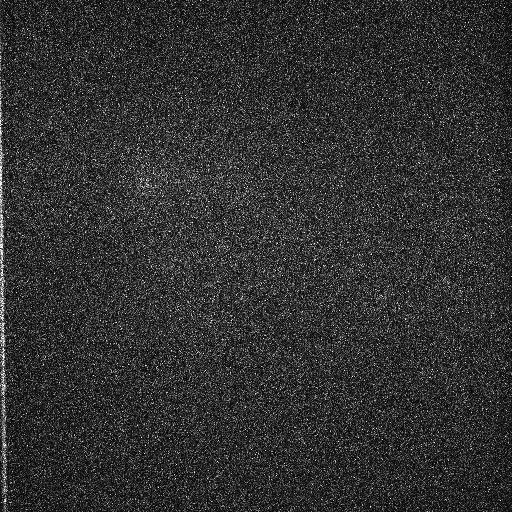

Supplement: Supplementary file 11 — Source data Fig. 2 [file 44318_2024_118_MOESM11_ESM.zip › Figure2/Figure 2A Micr. image/20201128 osm-3 G444E-gfp; HIS-54-BFP_3/Pos0/img_000000000_Confocal-561_000.tif]

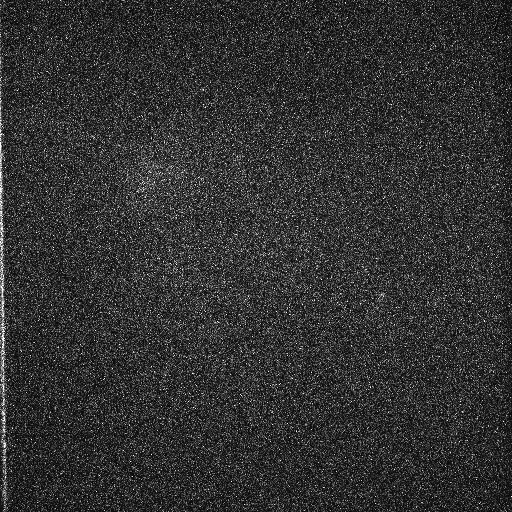

Supplement: Supplementary file 11 — Source data Fig. 2 [file 44318_2024_118_MOESM11_ESM.zip › Figure2/Figure 2A Micr. image/20201128 osm-3 G444E-gfp; HIS-54-BFP_3/Pos0/img_000000000_Confocal-561_001.tif]

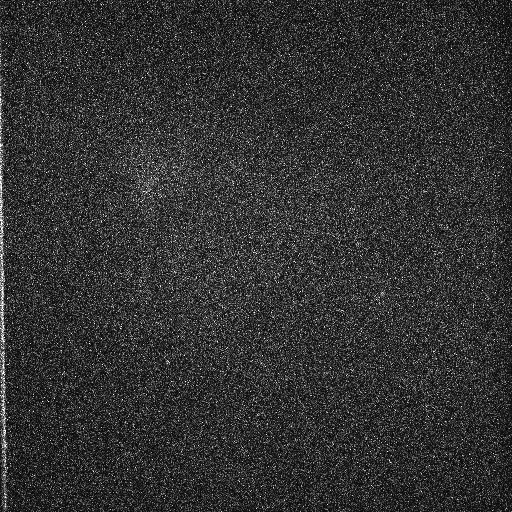

Supplement: Supplementary file 11 — Source data Fig. 2 [file 44318_2024_118_MOESM11_ESM.zip › Figure2/Figure 2A Micr. image/20201128 osm-3 G444E-gfp; HIS-54-BFP_3/Pos0/img_000000000_Confocal-561_002.tif]

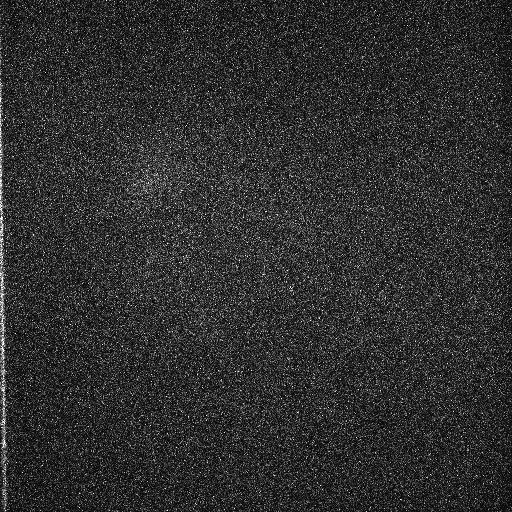

Supplement: Supplementary file 11 — Source data Fig. 2 [file 44318_2024_118_MOESM11_ESM.zip › Figure2/Figure 2A Micr. image/20201128 osm-3 G444E-gfp; HIS-54-BFP_3/Pos0/img_000000000_Confocal-561_003.tif]

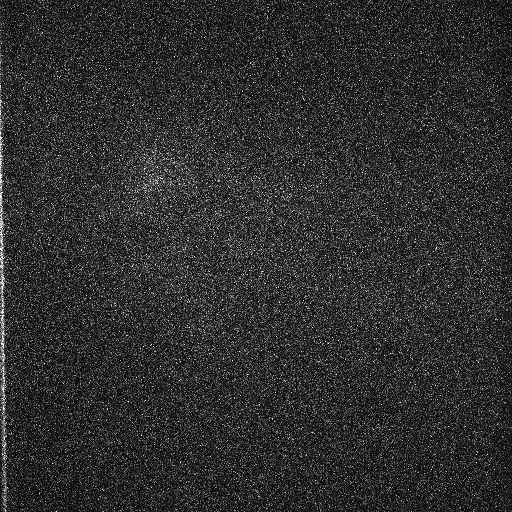

Supplement: Supplementary file 11 — Source data Fig. 2 [file 44318_2024_118_MOESM11_ESM.zip › Figure2/Figure 2A Micr. image/20201128 osm-3 G444E-gfp; HIS-54-BFP_3/Pos0/img_000000000_Confocal-561_004.tif]

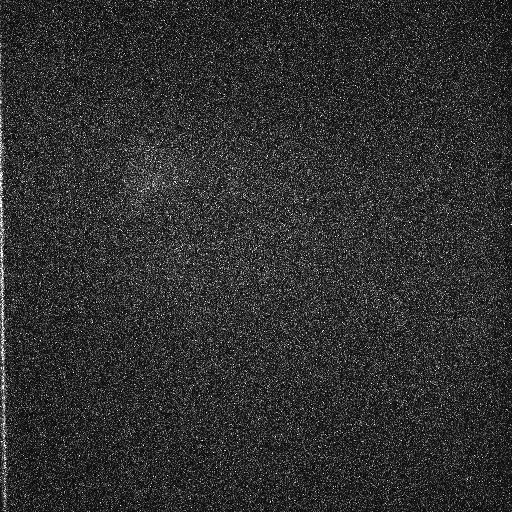

Supplement: Supplementary file 11 — Source data Fig. 2 [file 44318_2024_118_MOESM11_ESM.zip › Figure2/Figure 2A Micr. image/20201128 osm-3 G444E-gfp; HIS-54-BFP_3/Pos0/img_000000000_Confocal-561_005.tif]

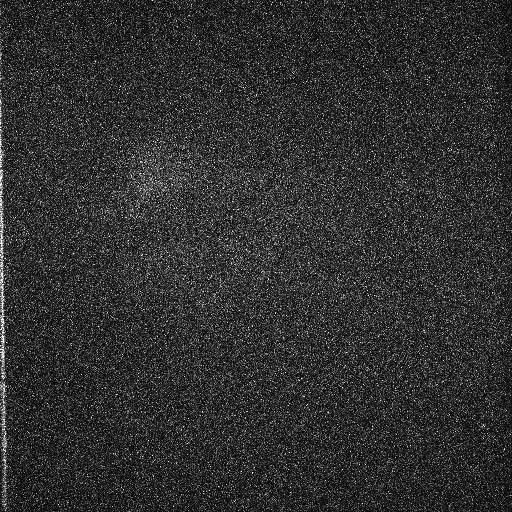

Supplement: Supplementary file 11 — Source data Fig. 2 [file 44318_2024_118_MOESM11_ESM.zip › Figure2/Figure 2A Micr. image/20201128 osm-3 G444E-gfp; HIS-54-BFP_3/Pos0/img_000000000_Confocal-561_006.tif]

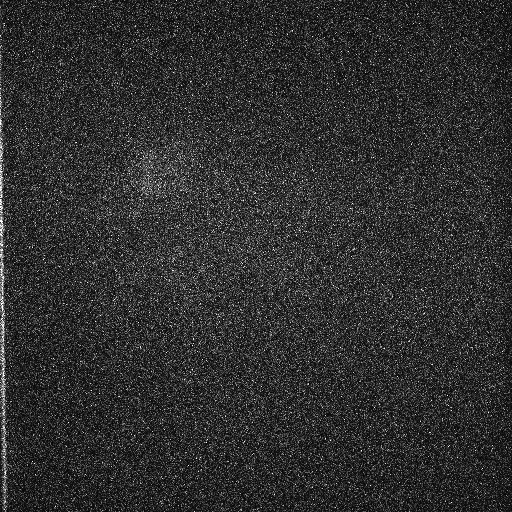

Supplement: Supplementary file 11 — Source data Fig. 2 [file 44318_2024_118_MOESM11_ESM.zip › Figure2/Figure 2A Micr. image/20201128 osm-3 G444E-gfp; HIS-54-BFP_3/Pos0/img_000000000_Confocal-561_007.tif]

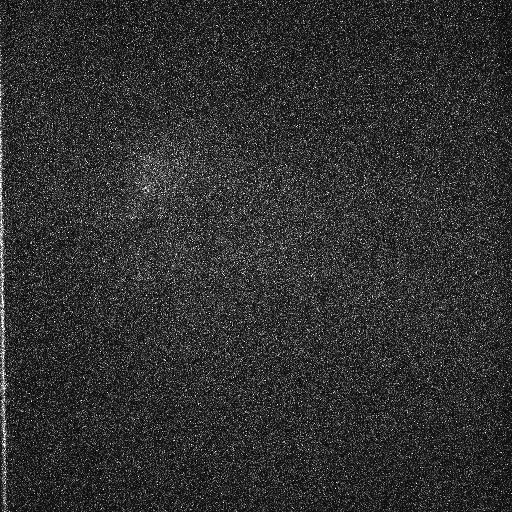

Supplement: Supplementary file 11 — Source data Fig. 2 [file 44318_2024_118_MOESM11_ESM.zip › Figure2/Figure 2A Micr. image/20201128 osm-3 G444E-gfp; HIS-54-BFP_3/Pos0/img_000000000_Confocal-561_008.tif]

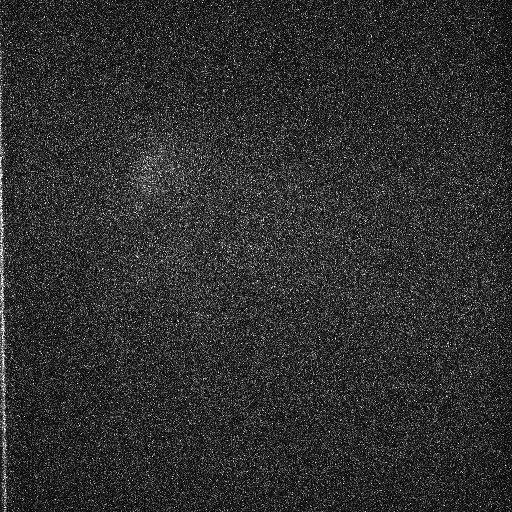

Supplement: Supplementary file 11 — Source data Fig. 2 [file 44318_2024_118_MOESM11_ESM.zip › Figure2/Figure 2A Micr. image/20201128 osm-3 G444E-gfp; HIS-54-BFP_3/Pos0/img_000000000_Confocal-561_009.tif]

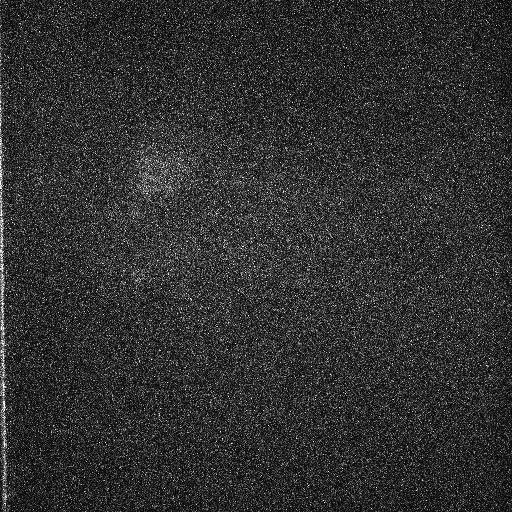

Supplement: Supplementary file 11 — Source data Fig. 2 [file 44318_2024_118_MOESM11_ESM.zip › Figure2/Figure 2A Micr. image/20201128 osm-3 G444E-gfp; HIS-54-BFP_3/Pos0/img_000000000_Confocal-561_010.tif]

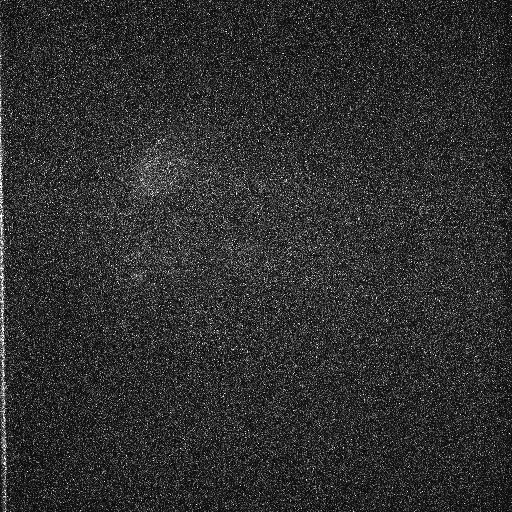

Supplement: Supplementary file 11 — Source data Fig. 2 [file 44318_2024_118_MOESM11_ESM.zip › Figure2/Figure 2A Micr. image/20201128 osm-3 G444E-gfp; HIS-54-BFP_3/Pos0/img_000000000_Confocal-561_011.tif]

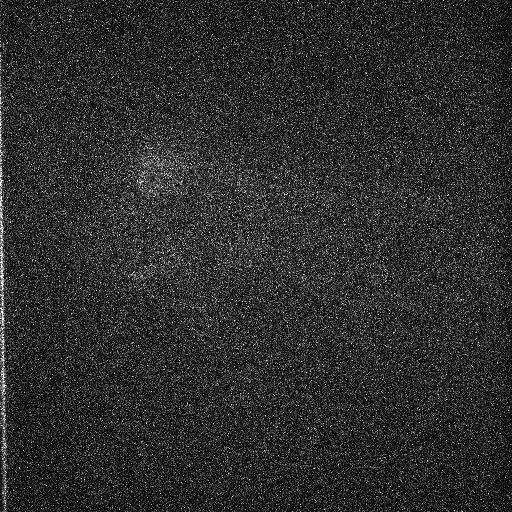

Supplement: Supplementary file 11 — Source data Fig. 2 [file 44318_2024_118_MOESM11_ESM.zip › Figure2/Figure 2A Micr. image/20201128 osm-3 G444E-gfp; HIS-54-BFP_3/Pos0/img_000000000_Confocal-561_012.tif]

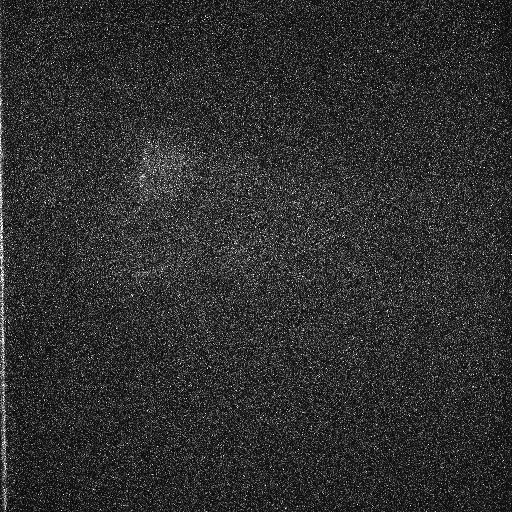

Supplement: Supplementary file 11 — Source data Fig. 2 [file 44318_2024_118_MOESM11_ESM.zip › Figure2/Figure 2A Micr. image/20201128 osm-3 G444E-gfp; HIS-54-BFP_3/Pos0/img_000000000_Confocal-561_013.tif]

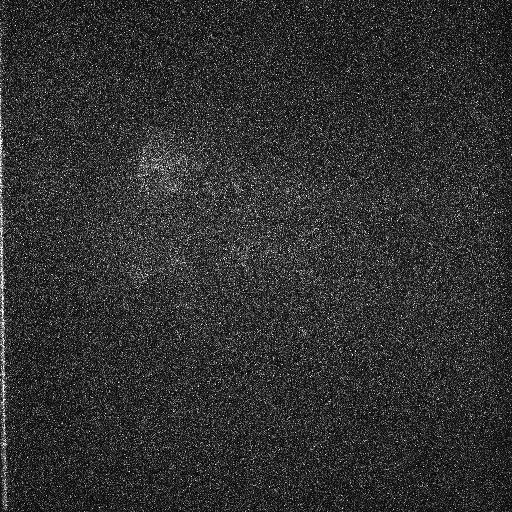

Supplement: Supplementary file 11 — Source data Fig. 2 [file 44318_2024_118_MOESM11_ESM.zip › Figure2/Figure 2A Micr. image/20201128 osm-3 G444E-gfp; HIS-54-BFP_3/Pos0/img_000000000_Confocal-561_014.tif]

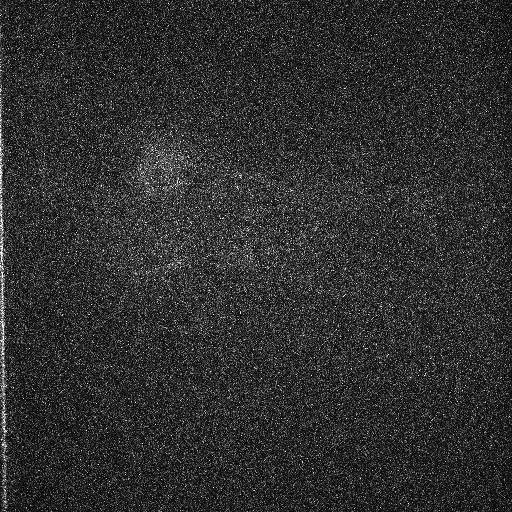

Supplement: Supplementary file 11 — Source data Fig. 2 [file 44318_2024_118_MOESM11_ESM.zip › Figure2/Figure 2A Micr. image/20201128 osm-3 G444E-gfp; HIS-54-BFP_3/Pos0/img_000000000_Confocal-561_015.tif]

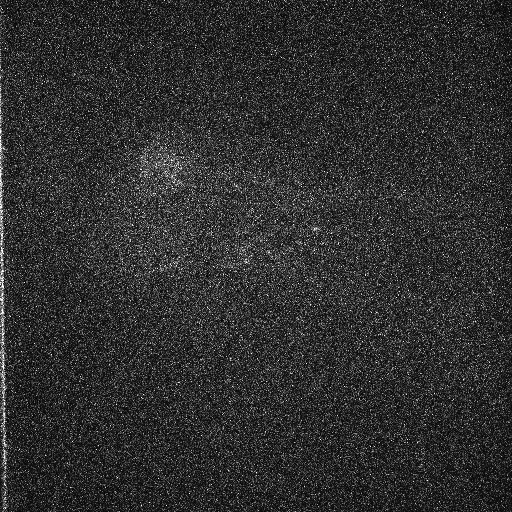

Supplement: Supplementary file 11 — Source data Fig. 2 [file 44318_2024_118_MOESM11_ESM.zip › Figure2/Figure 2A Micr. image/20201128 osm-3 G444E-gfp; HIS-54-BFP_3/Pos0/img_000000000_Confocal-561_016.tif]

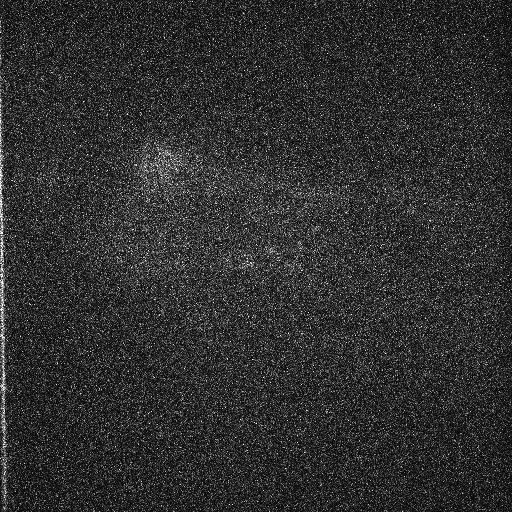

Supplement: Supplementary file 11 — Source data Fig. 2 [file 44318_2024_118_MOESM11_ESM.zip › Figure2/Figure 2A Micr. image/20201128 osm-3 G444E-gfp; HIS-54-BFP_3/Pos0/img_000000000_Confocal-561_017.tif]
