# Supplementary figures and images for: Neurons dispose of hyperactive kinesin into glial cells for clearance (part 6 of 9)
Source: EMBO J. 2024 May 28;43(13):5. doi: 10.1038/s44318-024-00118-0 (PMC11217292; doi:10.1038/s44318-024-00118-0)

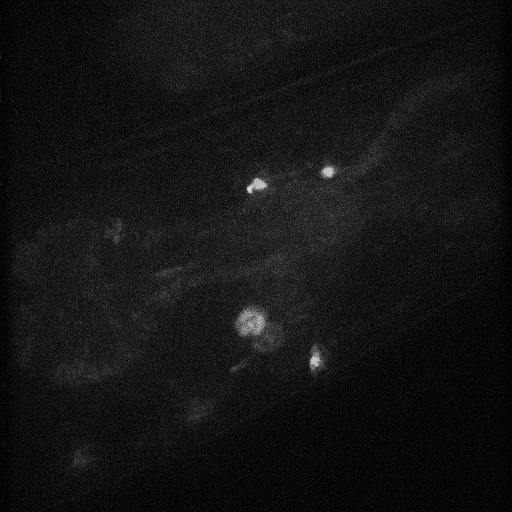

Supplement: Supplementary file 13 — Source data Fig. 4 [file 44318_2024_118_MOESM13_ESM.zip › Figure4/Figure 4C Micr. image/20220412 osm-3-G444E-gfp; ced-1_12/Pos0/img_000000000_Confocal-488-Acq_011.tif]

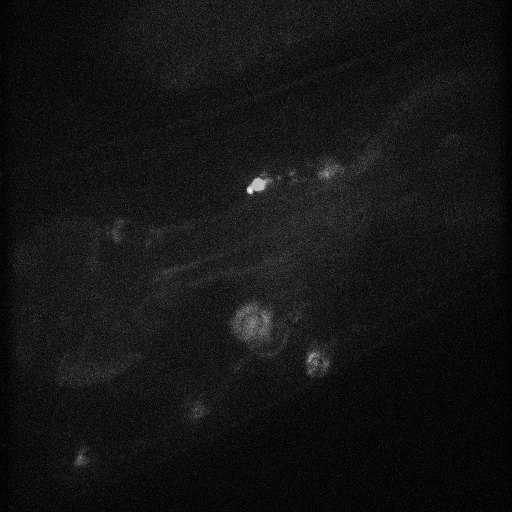

Supplement: Supplementary file 13 — Source data Fig. 4 [file 44318_2024_118_MOESM13_ESM.zip › Figure4/Figure 4C Micr. image/20220412 osm-3-G444E-gfp; ced-1_12/Pos0/img_000000000_Confocal-488-Acq_012.tif]

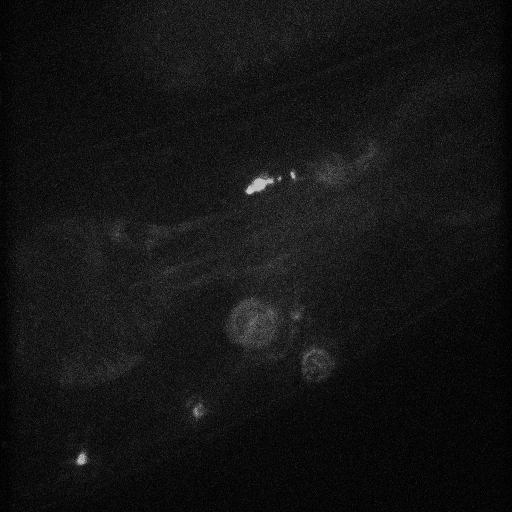

Supplement: Supplementary file 13 — Source data Fig. 4 [file 44318_2024_118_MOESM13_ESM.zip › Figure4/Figure 4C Micr. image/20220412 osm-3-G444E-gfp; ced-1_12/Pos0/img_000000000_Confocal-488-Acq_013.tif]

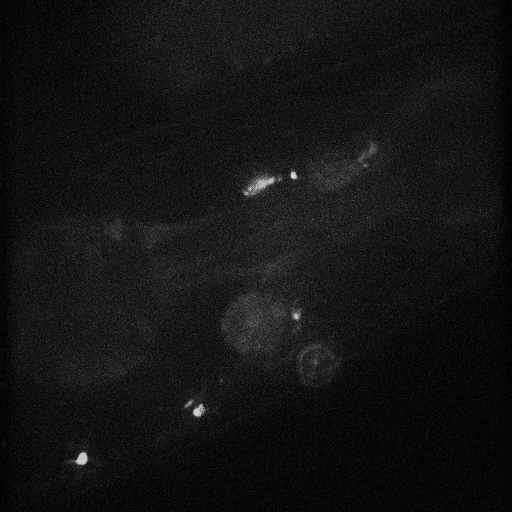

Supplement: Supplementary file 13 — Source data Fig. 4 [file 44318_2024_118_MOESM13_ESM.zip › Figure4/Figure 4C Micr. image/20220412 osm-3-G444E-gfp; ced-1_12/Pos0/img_000000000_Confocal-488-Acq_014.tif]

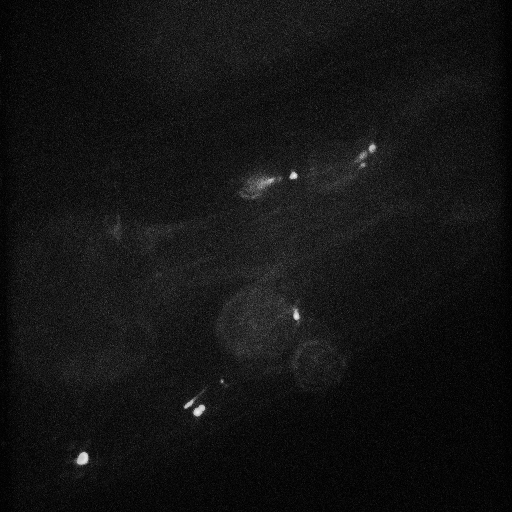

Supplement: Supplementary file 13 — Source data Fig. 4 [file 44318_2024_118_MOESM13_ESM.zip › Figure4/Figure 4C Micr. image/20220412 osm-3-G444E-gfp; ced-1_12/Pos0/img_000000000_Confocal-488-Acq_015.tif]

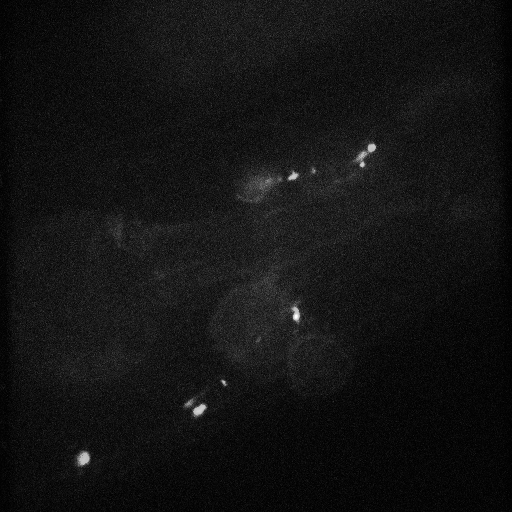

Supplement: Supplementary file 13 — Source data Fig. 4 [file 44318_2024_118_MOESM13_ESM.zip › Figure4/Figure 4C Micr. image/20220412 osm-3-G444E-gfp; ced-1_12/Pos0/img_000000000_Confocal-488-Acq_016.tif]

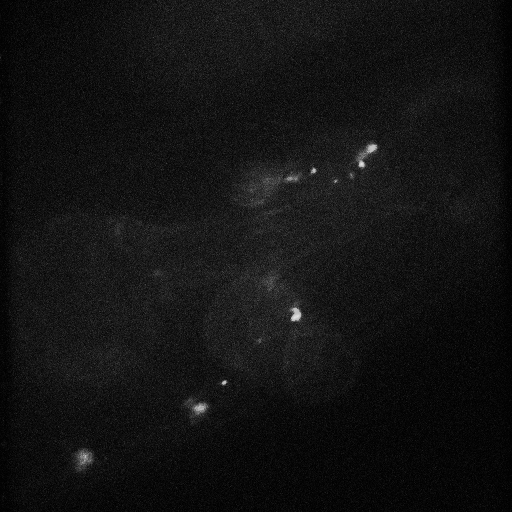

Supplement: Supplementary file 13 — Source data Fig. 4 [file 44318_2024_118_MOESM13_ESM.zip › Figure4/Figure 4C Micr. image/20220412 osm-3-G444E-gfp; ced-1_12/Pos0/img_000000000_Confocal-488-Acq_017.tif]

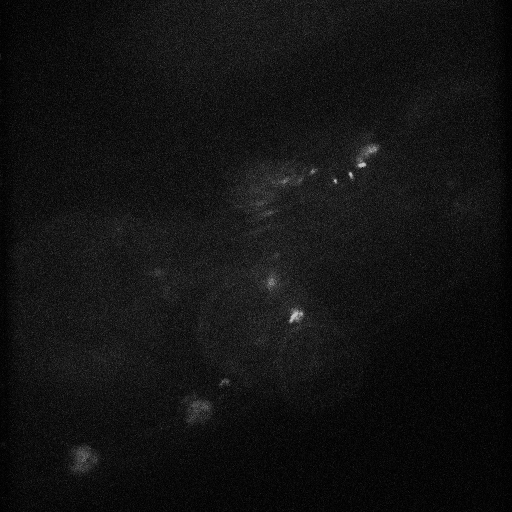

Supplement: Supplementary file 13 — Source data Fig. 4 [file 44318_2024_118_MOESM13_ESM.zip › Figure4/Figure 4C Micr. image/20220412 osm-3-G444E-gfp; ced-1_12/Pos0/img_000000000_Confocal-488-Acq_018.tif]

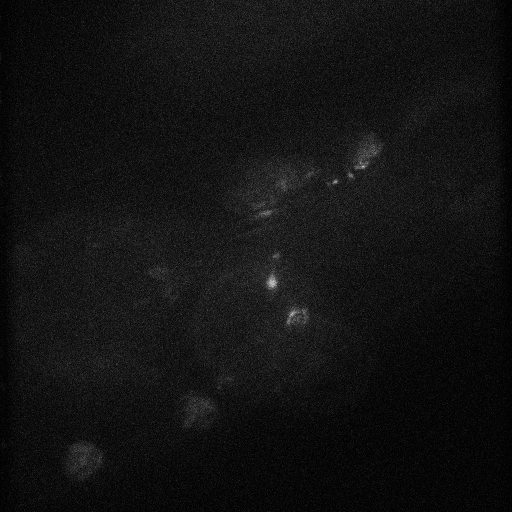

Supplement: Supplementary file 13 — Source data Fig. 4 [file 44318_2024_118_MOESM13_ESM.zip › Figure4/Figure 4C Micr. image/20220412 osm-3-G444E-gfp; ced-1_12/Pos0/img_000000000_Confocal-488-Acq_019.tif]

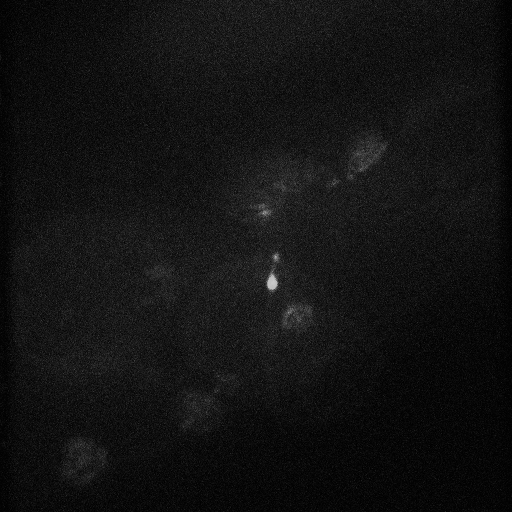

Supplement: Supplementary file 13 — Source data Fig. 4 [file 44318_2024_118_MOESM13_ESM.zip › Figure4/Figure 4C Micr. image/20220412 osm-3-G444E-gfp; ced-1_12/Pos0/img_000000000_Confocal-488-Acq_020.tif]

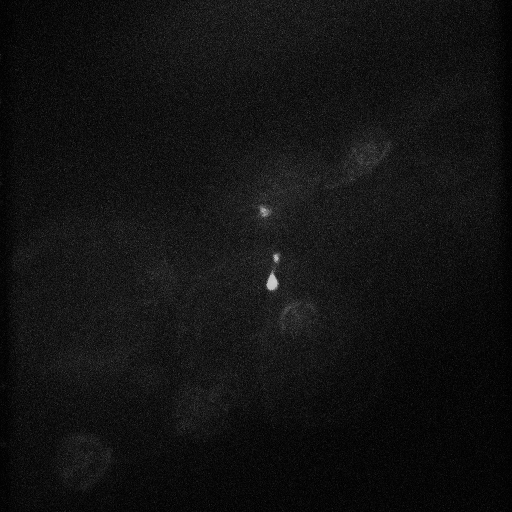

Supplement: Supplementary file 13 — Source data Fig. 4 [file 44318_2024_118_MOESM13_ESM.zip › Figure4/Figure 4C Micr. image/20220412 osm-3-G444E-gfp; ced-1_12/Pos0/img_000000000_Confocal-488-Acq_021.tif]

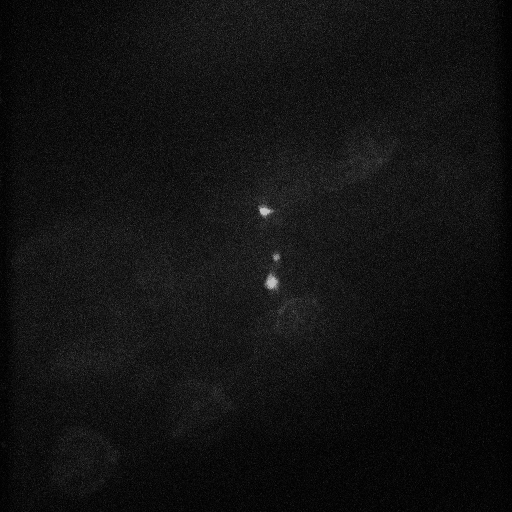

Supplement: Supplementary file 13 — Source data Fig. 4 [file 44318_2024_118_MOESM13_ESM.zip › Figure4/Figure 4C Micr. image/20220412 osm-3-G444E-gfp; ced-1_12/Pos0/img_000000000_Confocal-488-Acq_022.tif]

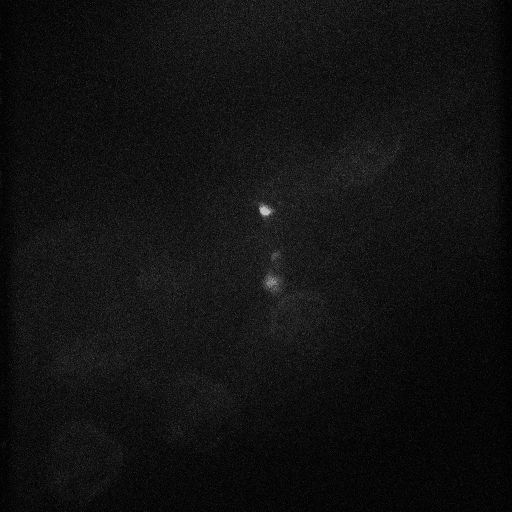

Supplement: Supplementary file 13 — Source data Fig. 4 [file 44318_2024_118_MOESM13_ESM.zip › Figure4/Figure 4C Micr. image/20220412 osm-3-G444E-gfp; ced-1_12/Pos0/img_000000000_Confocal-488-Acq_023.tif]

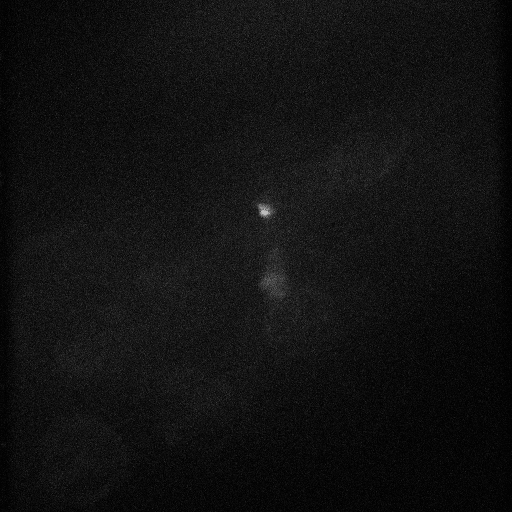

Supplement: Supplementary file 13 — Source data Fig. 4 [file 44318_2024_118_MOESM13_ESM.zip › Figure4/Figure 4C Micr. image/20220412 osm-3-G444E-gfp; ced-1_12/Pos0/img_000000000_Confocal-488-Acq_024.tif]

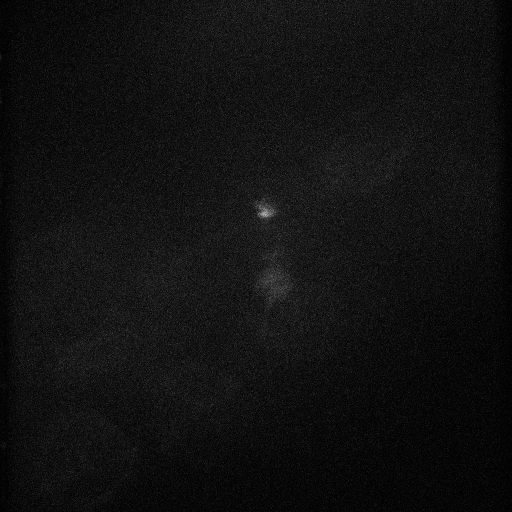

Supplement: Supplementary file 13 — Source data Fig. 4 [file 44318_2024_118_MOESM13_ESM.zip › Figure4/Figure 4C Micr. image/20220412 osm-3-G444E-gfp; ced-1_12/Pos0/img_000000000_Confocal-488-Acq_025.tif]

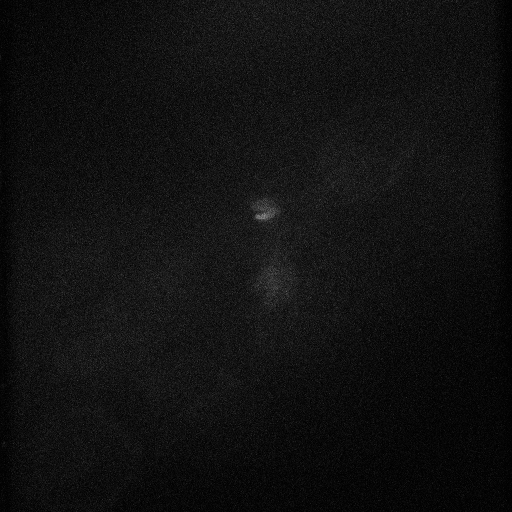

Supplement: Supplementary file 13 — Source data Fig. 4 [file 44318_2024_118_MOESM13_ESM.zip › Figure4/Figure 4C Micr. image/20220412 osm-3-G444E-gfp; ced-1_12/Pos0/img_000000000_Confocal-488-Acq_026.tif]

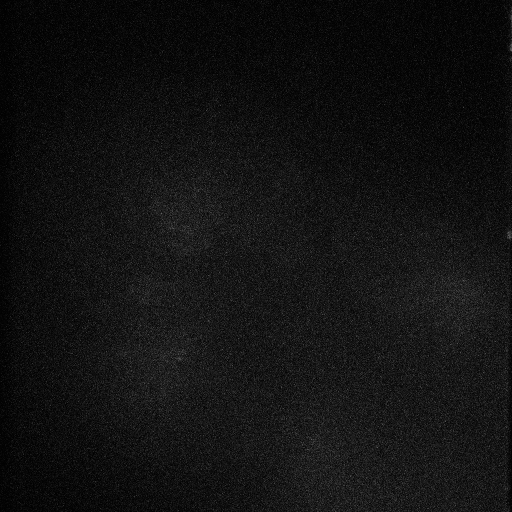

Supplement: Supplementary file 13 — Source data Fig. 4 [file 44318_2024_118_MOESM13_ESM.zip › Figure4/Figure 4C Micr. image/20221202 Pced-1-ced-1-mCherry; osm-3-G444E-gfp; ced-1(e1735)_6/img_000000000_L-488_000.tif]

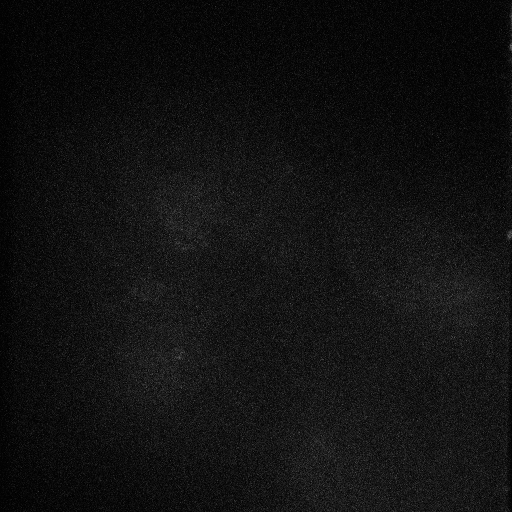

Supplement: Supplementary file 13 — Source data Fig. 4 [file 44318_2024_118_MOESM13_ESM.zip › Figure4/Figure 4C Micr. image/20221202 Pced-1-ced-1-mCherry; osm-3-G444E-gfp; ced-1(e1735)_6/img_000000000_L-488_001.tif]

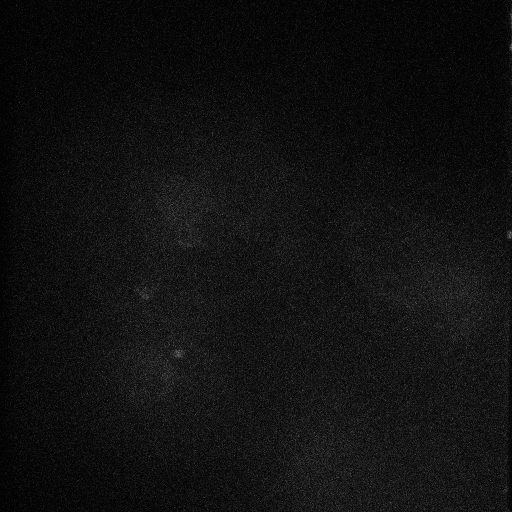

Supplement: Supplementary file 13 — Source data Fig. 4 [file 44318_2024_118_MOESM13_ESM.zip › Figure4/Figure 4C Micr. image/20221202 Pced-1-ced-1-mCherry; osm-3-G444E-gfp; ced-1(e1735)_6/img_000000000_L-488_002.tif]

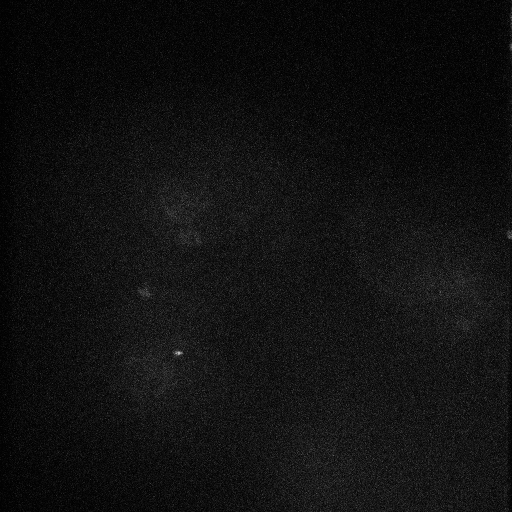

Supplement: Supplementary file 13 — Source data Fig. 4 [file 44318_2024_118_MOESM13_ESM.zip › Figure4/Figure 4C Micr. image/20221202 Pced-1-ced-1-mCherry; osm-3-G444E-gfp; ced-1(e1735)_6/img_000000000_L-488_003.tif]

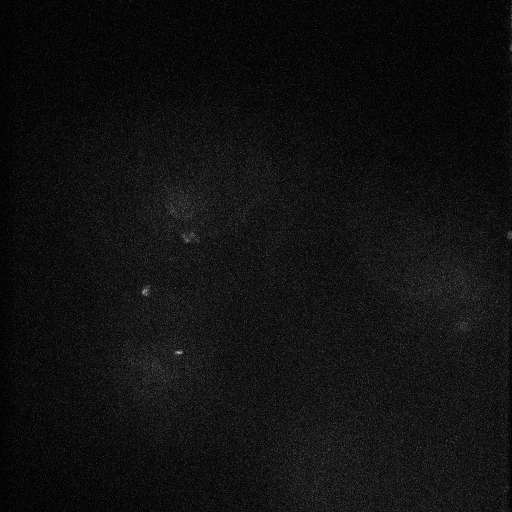

Supplement: Supplementary file 13 — Source data Fig. 4 [file 44318_2024_118_MOESM13_ESM.zip › Figure4/Figure 4C Micr. image/20221202 Pced-1-ced-1-mCherry; osm-3-G444E-gfp; ced-1(e1735)_6/img_000000000_L-488_004.tif]

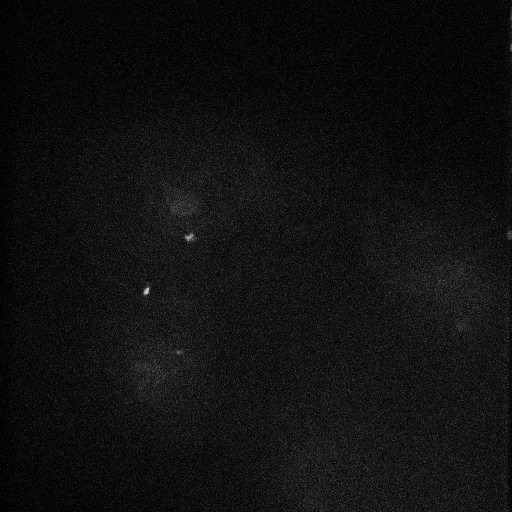

Supplement: Supplementary file 13 — Source data Fig. 4 [file 44318_2024_118_MOESM13_ESM.zip › Figure4/Figure 4C Micr. image/20221202 Pced-1-ced-1-mCherry; osm-3-G444E-gfp; ced-1(e1735)_6/img_000000000_L-488_005.tif]

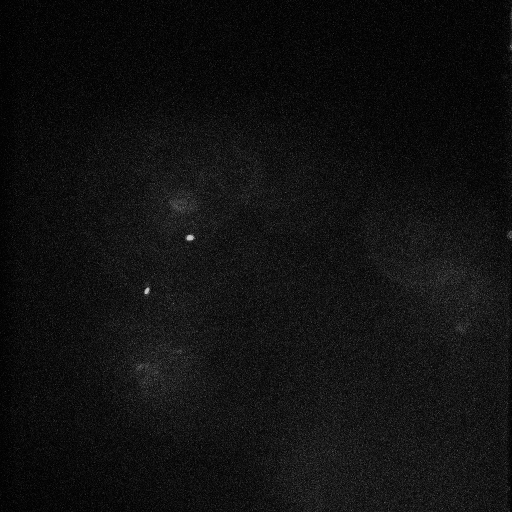

Supplement: Supplementary file 13 — Source data Fig. 4 [file 44318_2024_118_MOESM13_ESM.zip › Figure4/Figure 4C Micr. image/20221202 Pced-1-ced-1-mCherry; osm-3-G444E-gfp; ced-1(e1735)_6/img_000000000_L-488_006.tif]

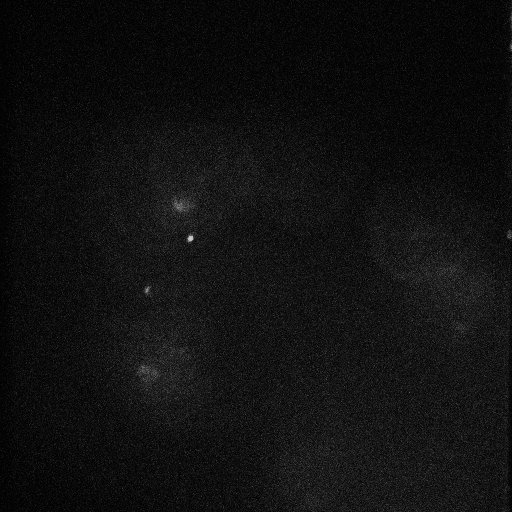

Supplement: Supplementary file 13 — Source data Fig. 4 [file 44318_2024_118_MOESM13_ESM.zip › Figure4/Figure 4C Micr. image/20221202 Pced-1-ced-1-mCherry; osm-3-G444E-gfp; ced-1(e1735)_6/img_000000000_L-488_007.tif]

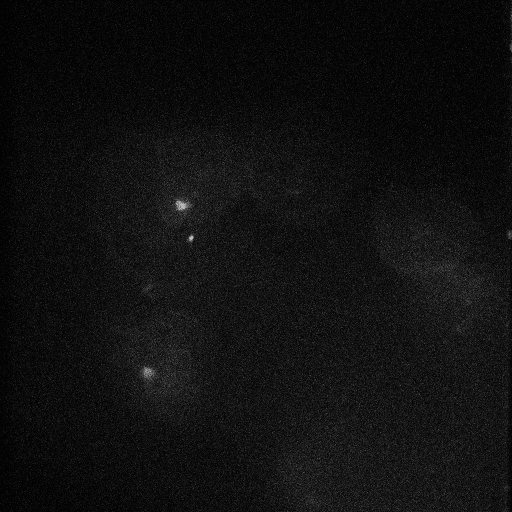

Supplement: Supplementary file 13 — Source data Fig. 4 [file 44318_2024_118_MOESM13_ESM.zip › Figure4/Figure 4C Micr. image/20221202 Pced-1-ced-1-mCherry; osm-3-G444E-gfp; ced-1(e1735)_6/img_000000000_L-488_008.tif]

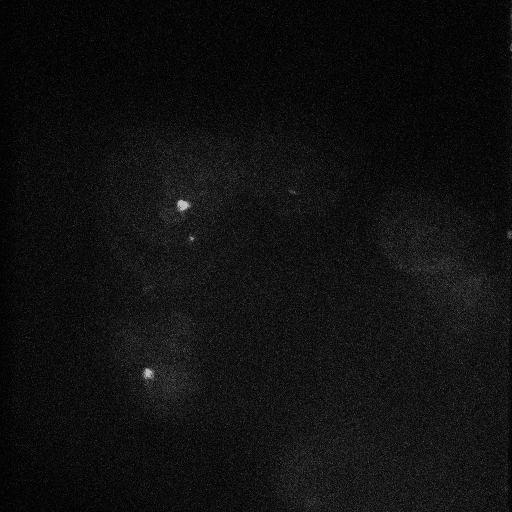

Supplement: Supplementary file 13 — Source data Fig. 4 [file 44318_2024_118_MOESM13_ESM.zip › Figure4/Figure 4C Micr. image/20221202 Pced-1-ced-1-mCherry; osm-3-G444E-gfp; ced-1(e1735)_6/img_000000000_L-488_009.tif]

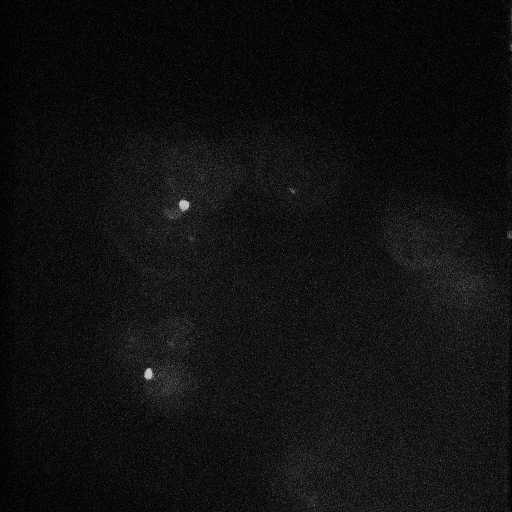

Supplement: Supplementary file 13 — Source data Fig. 4 [file 44318_2024_118_MOESM13_ESM.zip › Figure4/Figure 4C Micr. image/20221202 Pced-1-ced-1-mCherry; osm-3-G444E-gfp; ced-1(e1735)_6/img_000000000_L-488_010.tif]

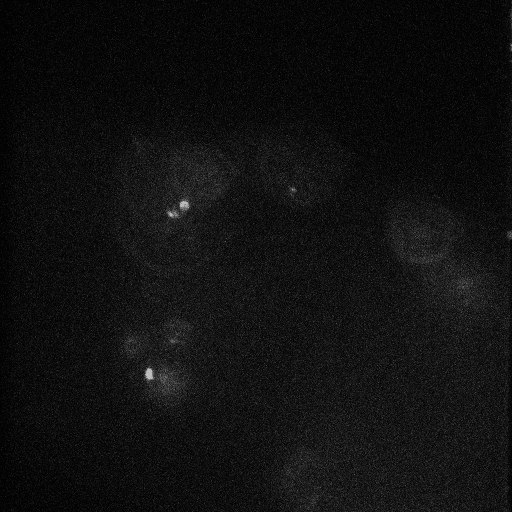

Supplement: Supplementary file 13 — Source data Fig. 4 [file 44318_2024_118_MOESM13_ESM.zip › Figure4/Figure 4C Micr. image/20221202 Pced-1-ced-1-mCherry; osm-3-G444E-gfp; ced-1(e1735)_6/img_000000000_L-488_011.tif]

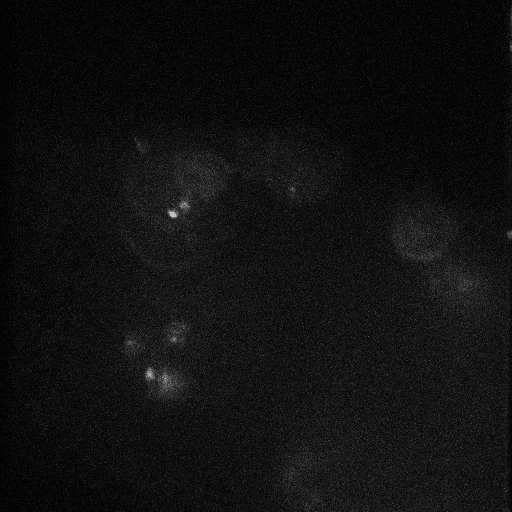

Supplement: Supplementary file 13 — Source data Fig. 4 [file 44318_2024_118_MOESM13_ESM.zip › Figure4/Figure 4C Micr. image/20221202 Pced-1-ced-1-mCherry; osm-3-G444E-gfp; ced-1(e1735)_6/img_000000000_L-488_012.tif]

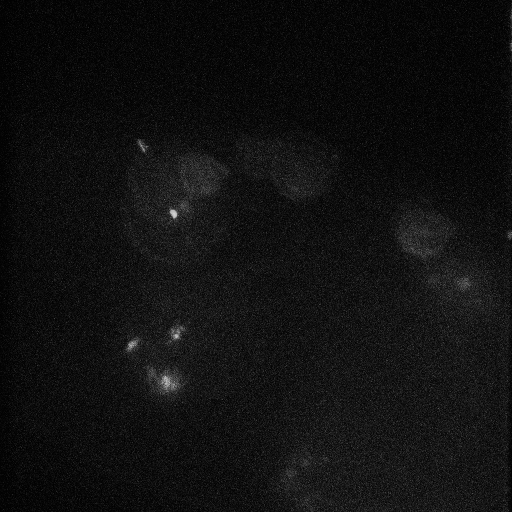

Supplement: Supplementary file 13 — Source data Fig. 4 [file 44318_2024_118_MOESM13_ESM.zip › Figure4/Figure 4C Micr. image/20221202 Pced-1-ced-1-mCherry; osm-3-G444E-gfp; ced-1(e1735)_6/img_000000000_L-488_013.tif]

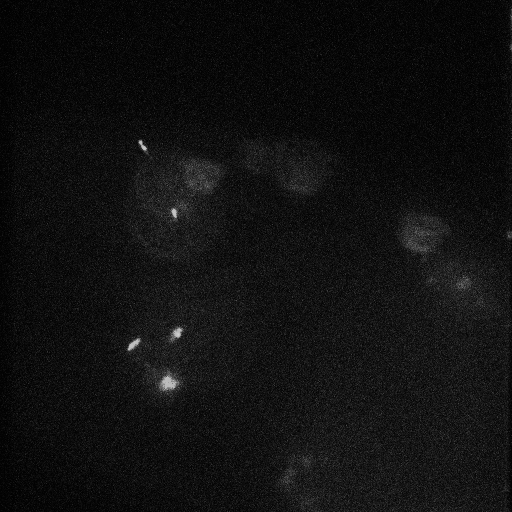

Supplement: Supplementary file 13 — Source data Fig. 4 [file 44318_2024_118_MOESM13_ESM.zip › Figure4/Figure 4C Micr. image/20221202 Pced-1-ced-1-mCherry; osm-3-G444E-gfp; ced-1(e1735)_6/img_000000000_L-488_014.tif]

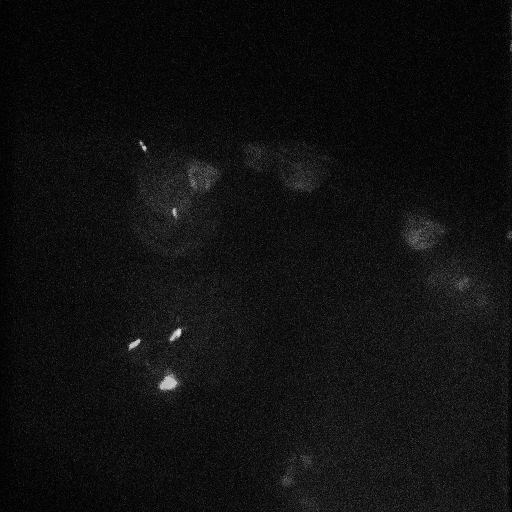

Supplement: Supplementary file 13 — Source data Fig. 4 [file 44318_2024_118_MOESM13_ESM.zip › Figure4/Figure 4C Micr. image/20221202 Pced-1-ced-1-mCherry; osm-3-G444E-gfp; ced-1(e1735)_6/img_000000000_L-488_015.tif]

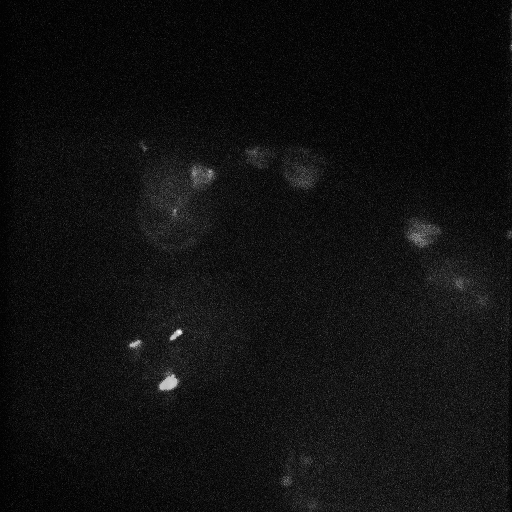

Supplement: Supplementary file 13 — Source data Fig. 4 [file 44318_2024_118_MOESM13_ESM.zip › Figure4/Figure 4C Micr. image/20221202 Pced-1-ced-1-mCherry; osm-3-G444E-gfp; ced-1(e1735)_6/img_000000000_L-488_016.tif]

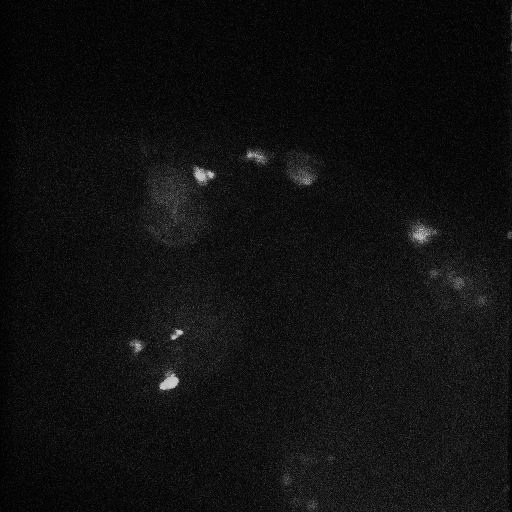

Supplement: Supplementary file 13 — Source data Fig. 4 [file 44318_2024_118_MOESM13_ESM.zip › Figure4/Figure 4C Micr. image/20221202 Pced-1-ced-1-mCherry; osm-3-G444E-gfp; ced-1(e1735)_6/img_000000000_L-488_017.tif]

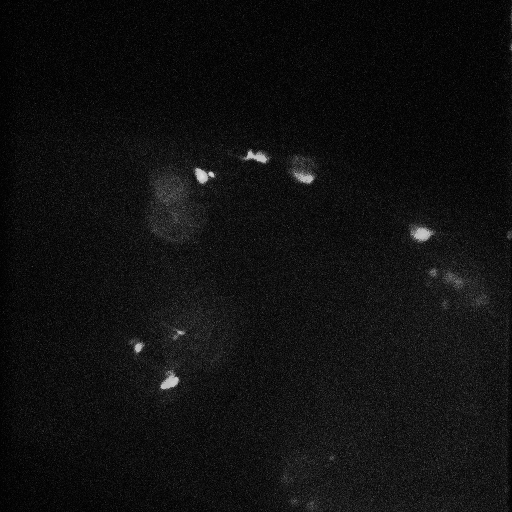

Supplement: Supplementary file 13 — Source data Fig. 4 [file 44318_2024_118_MOESM13_ESM.zip › Figure4/Figure 4C Micr. image/20221202 Pced-1-ced-1-mCherry; osm-3-G444E-gfp; ced-1(e1735)_6/img_000000000_L-488_018.tif]

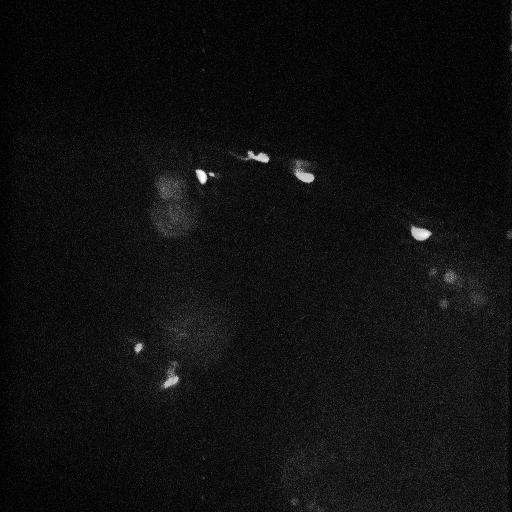

Supplement: Supplementary file 13 — Source data Fig. 4 [file 44318_2024_118_MOESM13_ESM.zip › Figure4/Figure 4C Micr. image/20221202 Pced-1-ced-1-mCherry; osm-3-G444E-gfp; ced-1(e1735)_6/img_000000000_L-488_019.tif]

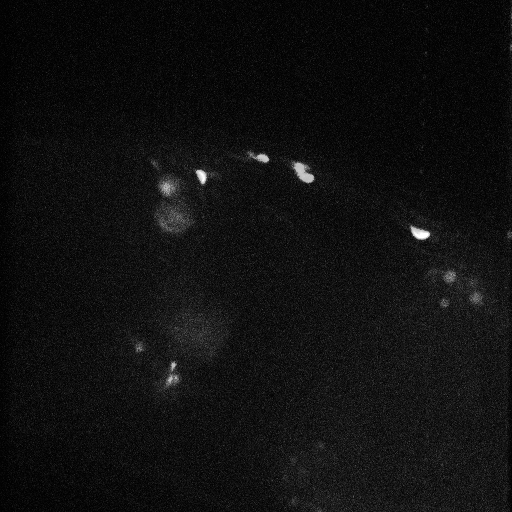

Supplement: Supplementary file 13 — Source data Fig. 4 [file 44318_2024_118_MOESM13_ESM.zip › Figure4/Figure 4C Micr. image/20221202 Pced-1-ced-1-mCherry; osm-3-G444E-gfp; ced-1(e1735)_6/img_000000000_L-488_020.tif]

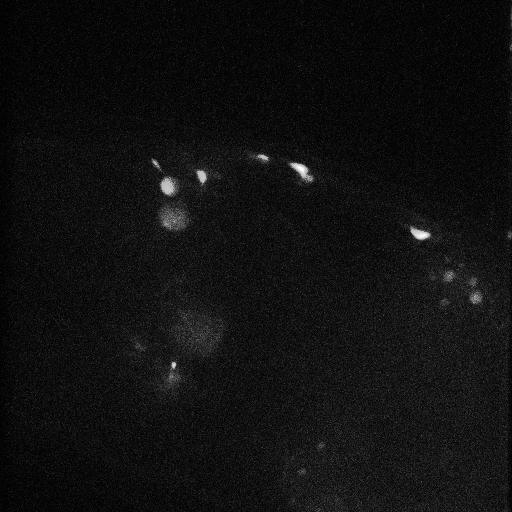

Supplement: Supplementary file 13 — Source data Fig. 4 [file 44318_2024_118_MOESM13_ESM.zip › Figure4/Figure 4C Micr. image/20221202 Pced-1-ced-1-mCherry; osm-3-G444E-gfp; ced-1(e1735)_6/img_000000000_L-488_021.tif]

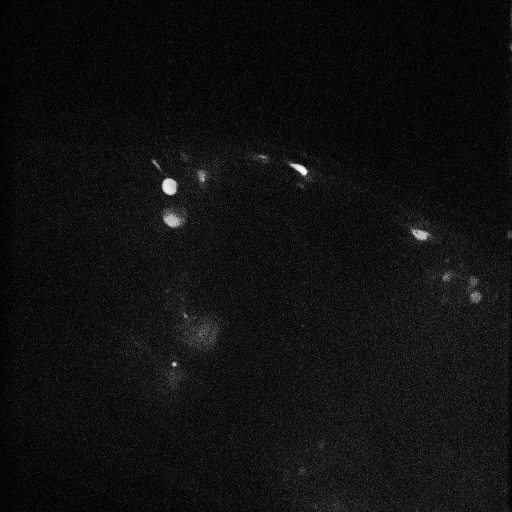

Supplement: Supplementary file 13 — Source data Fig. 4 [file 44318_2024_118_MOESM13_ESM.zip › Figure4/Figure 4C Micr. image/20221202 Pced-1-ced-1-mCherry; osm-3-G444E-gfp; ced-1(e1735)_6/img_000000000_L-488_022.tif]

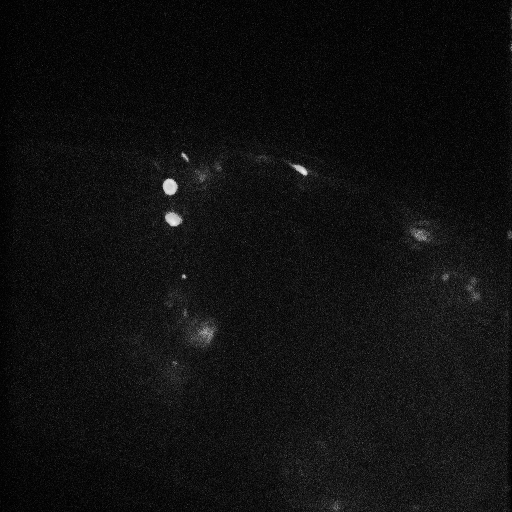

Supplement: Supplementary file 13 — Source data Fig. 4 [file 44318_2024_118_MOESM13_ESM.zip › Figure4/Figure 4C Micr. image/20221202 Pced-1-ced-1-mCherry; osm-3-G444E-gfp; ced-1(e1735)_6/img_000000000_L-488_023.tif]

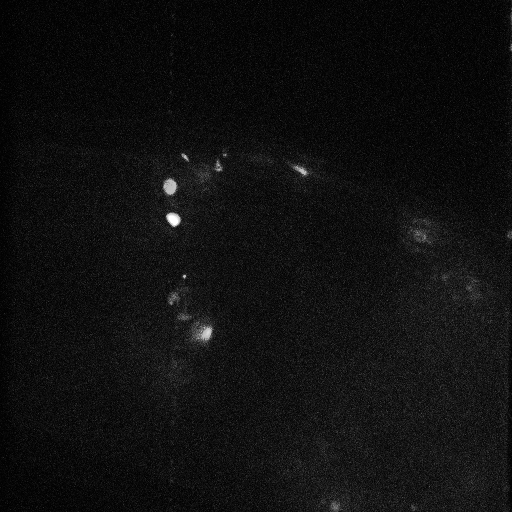

Supplement: Supplementary file 13 — Source data Fig. 4 [file 44318_2024_118_MOESM13_ESM.zip › Figure4/Figure 4C Micr. image/20221202 Pced-1-ced-1-mCherry; osm-3-G444E-gfp; ced-1(e1735)_6/img_000000000_L-488_024.tif]

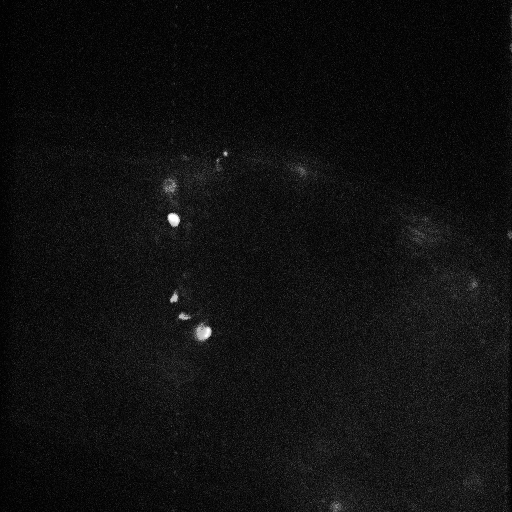

Supplement: Supplementary file 13 — Source data Fig. 4 [file 44318_2024_118_MOESM13_ESM.zip › Figure4/Figure 4C Micr. image/20221202 Pced-1-ced-1-mCherry; osm-3-G444E-gfp; ced-1(e1735)_6/img_000000000_L-488_025.tif]

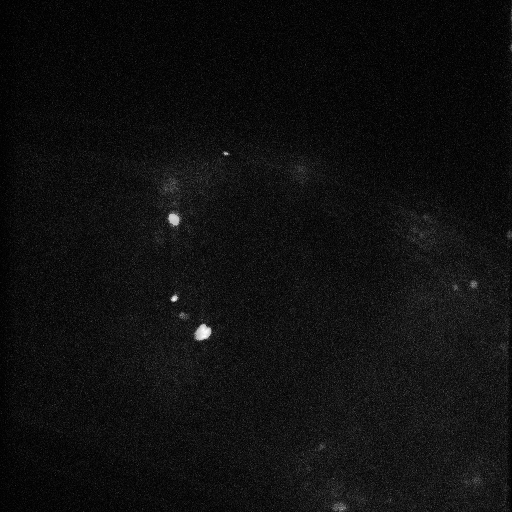

Supplement: Supplementary file 13 — Source data Fig. 4 [file 44318_2024_118_MOESM13_ESM.zip › Figure4/Figure 4C Micr. image/20221202 Pced-1-ced-1-mCherry; osm-3-G444E-gfp; ced-1(e1735)_6/img_000000000_L-488_026.tif]

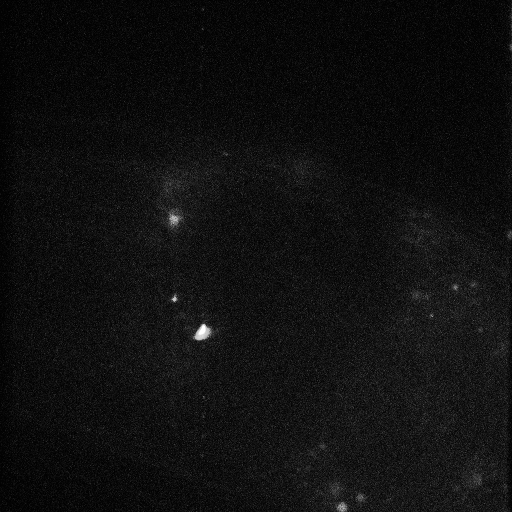

Supplement: Supplementary file 13 — Source data Fig. 4 [file 44318_2024_118_MOESM13_ESM.zip › Figure4/Figure 4C Micr. image/20221202 Pced-1-ced-1-mCherry; osm-3-G444E-gfp; ced-1(e1735)_6/img_000000000_L-488_027.tif]

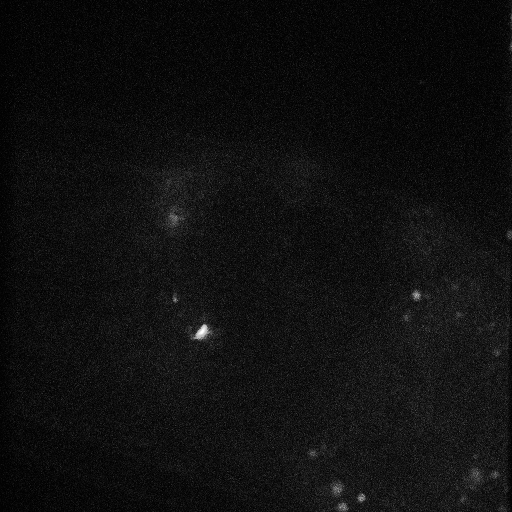

Supplement: Supplementary file 13 — Source data Fig. 4 [file 44318_2024_118_MOESM13_ESM.zip › Figure4/Figure 4C Micr. image/20221202 Pced-1-ced-1-mCherry; osm-3-G444E-gfp; ced-1(e1735)_6/img_000000000_L-488_028.tif]

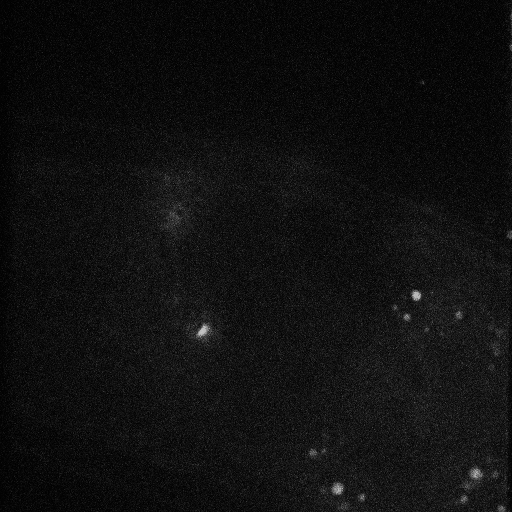

Supplement: Supplementary file 13 — Source data Fig. 4 [file 44318_2024_118_MOESM13_ESM.zip › Figure4/Figure 4C Micr. image/20221202 Pced-1-ced-1-mCherry; osm-3-G444E-gfp; ced-1(e1735)_6/img_000000000_L-488_029.tif]

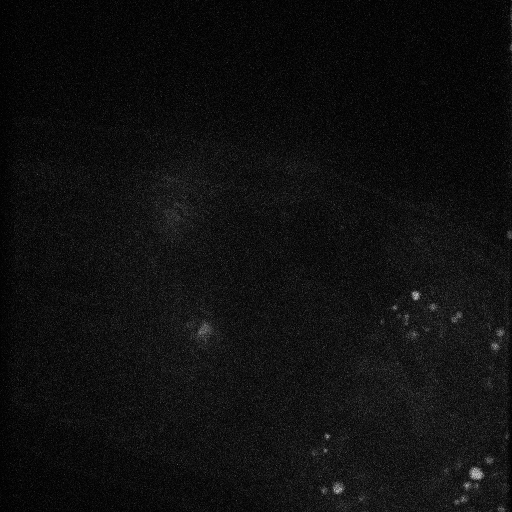

Supplement: Supplementary file 13 — Source data Fig. 4 [file 44318_2024_118_MOESM13_ESM.zip › Figure4/Figure 4C Micr. image/20221202 Pced-1-ced-1-mCherry; osm-3-G444E-gfp; ced-1(e1735)_6/img_000000000_L-488_030.tif]

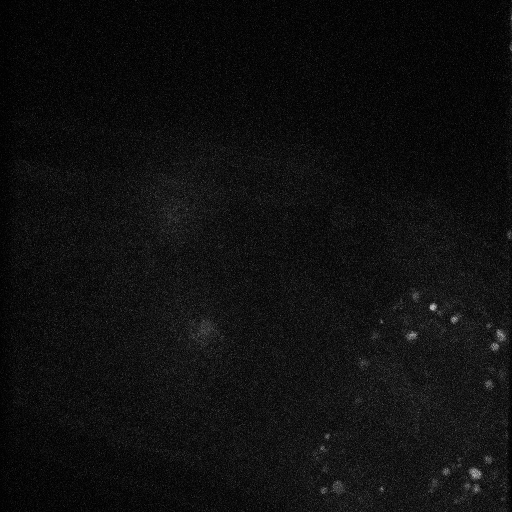

Supplement: Supplementary file 13 — Source data Fig. 4 [file 44318_2024_118_MOESM13_ESM.zip › Figure4/Figure 4C Micr. image/20221202 Pced-1-ced-1-mCherry; osm-3-G444E-gfp; ced-1(e1735)_6/img_000000000_L-488_031.tif]

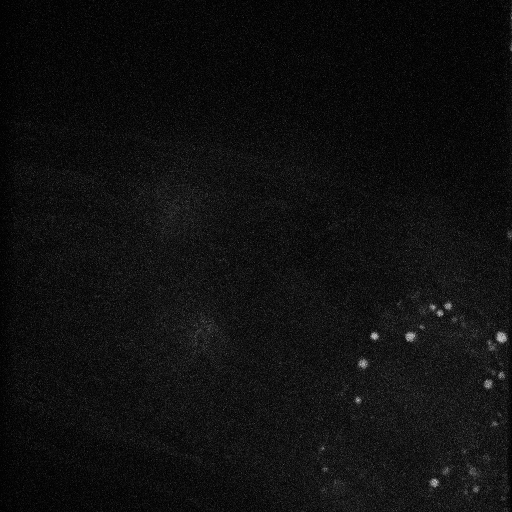

Supplement: Supplementary file 13 — Source data Fig. 4 [file 44318_2024_118_MOESM13_ESM.zip › Figure4/Figure 4C Micr. image/20221202 Pced-1-ced-1-mCherry; osm-3-G444E-gfp; ced-1(e1735)_6/img_000000000_L-488_032.tif]

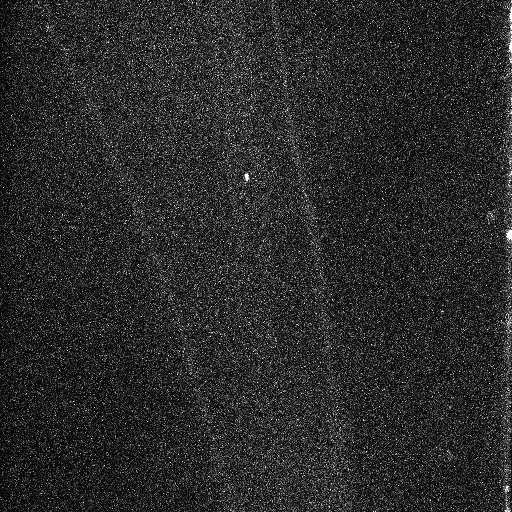

Supplement: Supplementary file 13 — Source data Fig. 4 [file 44318_2024_118_MOESM13_ESM.zip › Figure4/Figure 4C Micr. image/20230216 Phlh-17-ced-1; ced-1(e1735); G444E-gfp young adult_3/img_000000000_L-488_000.tif]

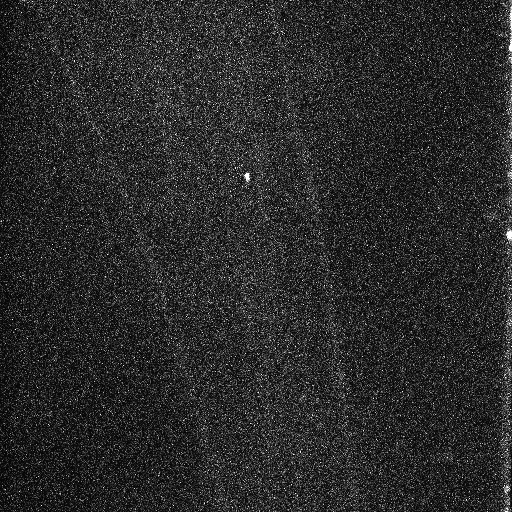

Supplement: Supplementary file 13 — Source data Fig. 4 [file 44318_2024_118_MOESM13_ESM.zip › Figure4/Figure 4C Micr. image/20230216 Phlh-17-ced-1; ced-1(e1735); G444E-gfp young adult_3/img_000000000_L-488_001.tif]

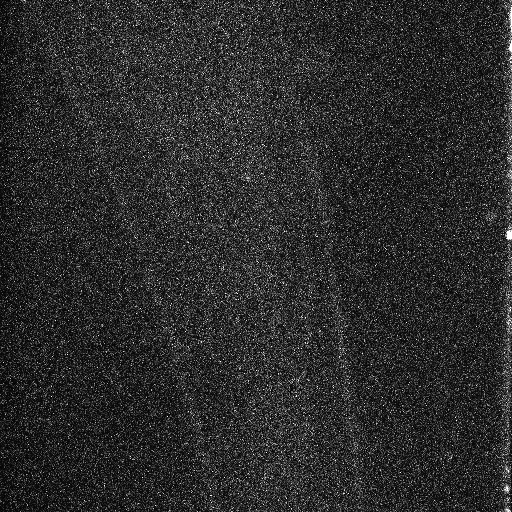

Supplement: Supplementary file 13 — Source data Fig. 4 [file 44318_2024_118_MOESM13_ESM.zip › Figure4/Figure 4C Micr. image/20230216 Phlh-17-ced-1; ced-1(e1735); G444E-gfp young adult_3/img_000000000_L-488_002.tif]

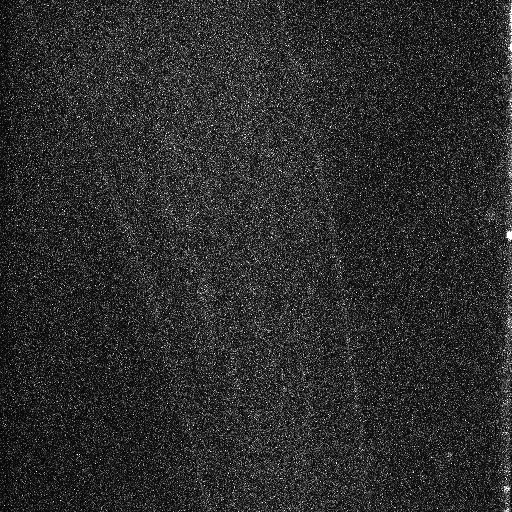

Supplement: Supplementary file 13 — Source data Fig. 4 [file 44318_2024_118_MOESM13_ESM.zip › Figure4/Figure 4C Micr. image/20230216 Phlh-17-ced-1; ced-1(e1735); G444E-gfp young adult_3/img_000000000_L-488_003.tif]

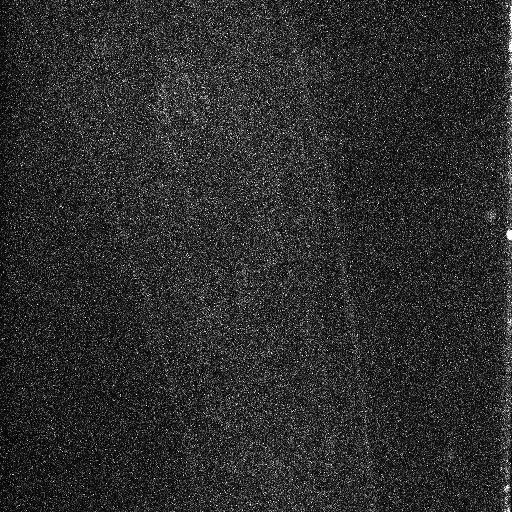

Supplement: Supplementary file 13 — Source data Fig. 4 [file 44318_2024_118_MOESM13_ESM.zip › Figure4/Figure 4C Micr. image/20230216 Phlh-17-ced-1; ced-1(e1735); G444E-gfp young adult_3/img_000000000_L-488_004.tif]

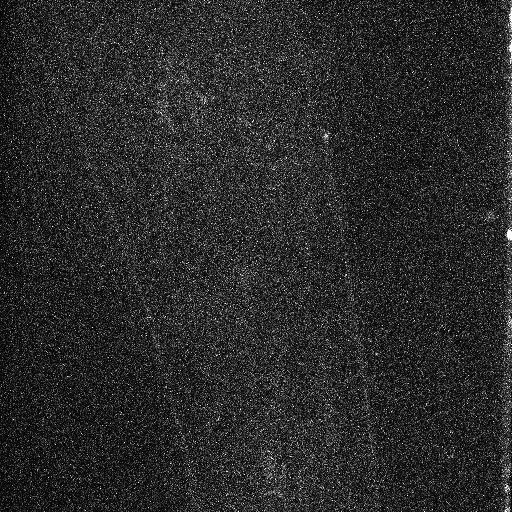

Supplement: Supplementary file 13 — Source data Fig. 4 [file 44318_2024_118_MOESM13_ESM.zip › Figure4/Figure 4C Micr. image/20230216 Phlh-17-ced-1; ced-1(e1735); G444E-gfp young adult_3/img_000000000_L-488_005.tif]

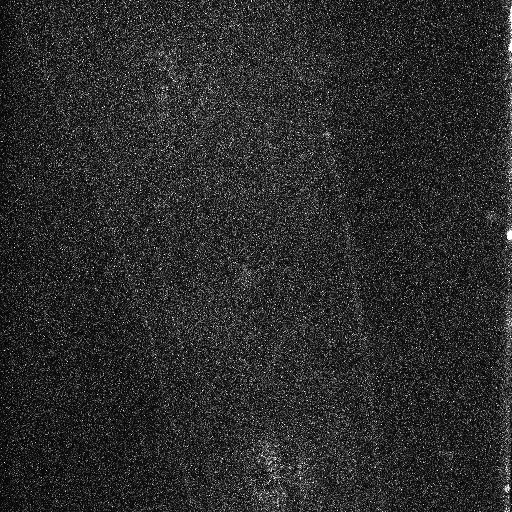

Supplement: Supplementary file 13 — Source data Fig. 4 [file 44318_2024_118_MOESM13_ESM.zip › Figure4/Figure 4C Micr. image/20230216 Phlh-17-ced-1; ced-1(e1735); G444E-gfp young adult_3/img_000000000_L-488_006.tif]

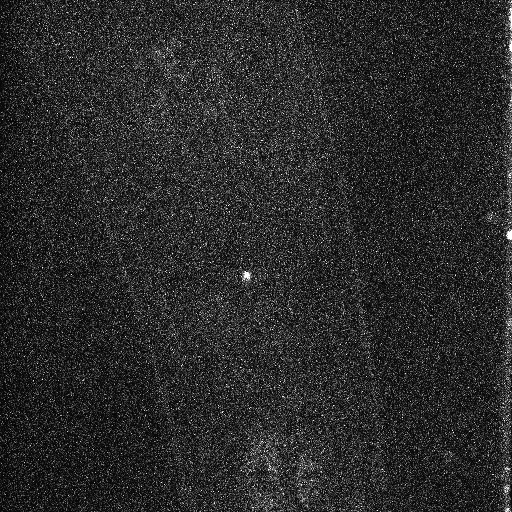

Supplement: Supplementary file 13 — Source data Fig. 4 [file 44318_2024_118_MOESM13_ESM.zip › Figure4/Figure 4C Micr. image/20230216 Phlh-17-ced-1; ced-1(e1735); G444E-gfp young adult_3/img_000000000_L-488_007.tif]

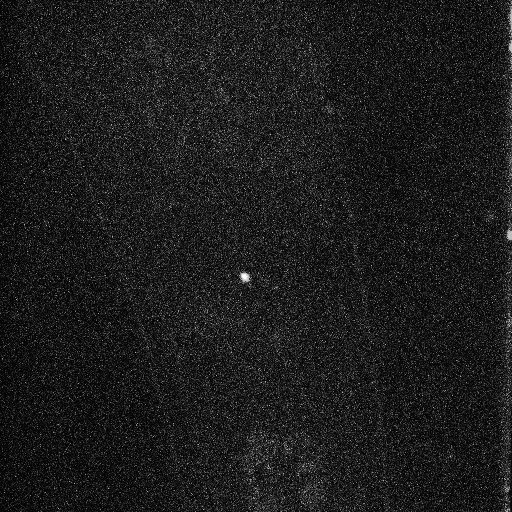

Supplement: Supplementary file 13 — Source data Fig. 4 [file 44318_2024_118_MOESM13_ESM.zip › Figure4/Figure 4C Micr. image/20230216 Phlh-17-ced-1; ced-1(e1735); G444E-gfp young adult_3/img_000000000_L-488_008.tif]

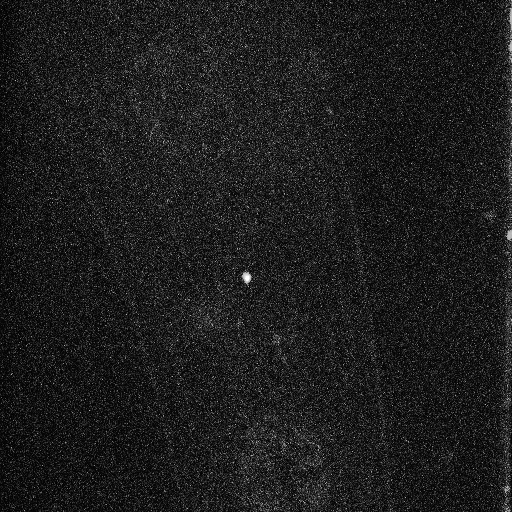

Supplement: Supplementary file 13 — Source data Fig. 4 [file 44318_2024_118_MOESM13_ESM.zip › Figure4/Figure 4C Micr. image/20230216 Phlh-17-ced-1; ced-1(e1735); G444E-gfp young adult_3/img_000000000_L-488_009.tif]

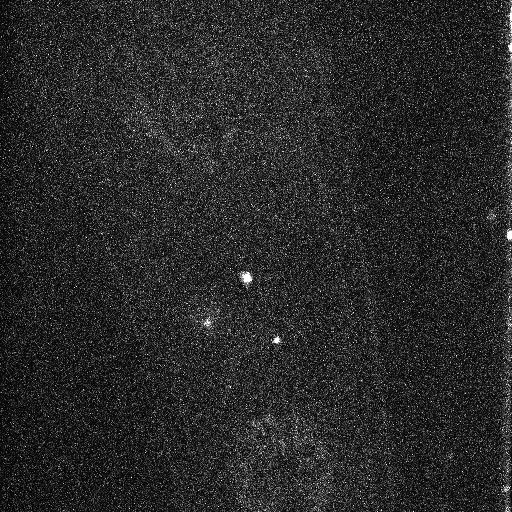

Supplement: Supplementary file 13 — Source data Fig. 4 [file 44318_2024_118_MOESM13_ESM.zip › Figure4/Figure 4C Micr. image/20230216 Phlh-17-ced-1; ced-1(e1735); G444E-gfp young adult_3/img_000000000_L-488_010.tif]

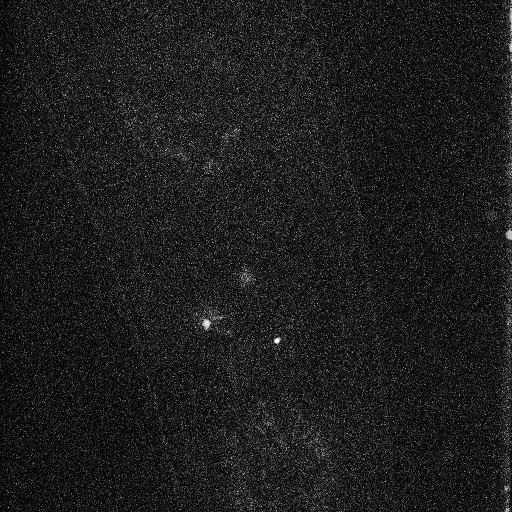

Supplement: Supplementary file 13 — Source data Fig. 4 [file 44318_2024_118_MOESM13_ESM.zip › Figure4/Figure 4C Micr. image/20230216 Phlh-17-ced-1; ced-1(e1735); G444E-gfp young adult_3/img_000000000_L-488_011.tif]

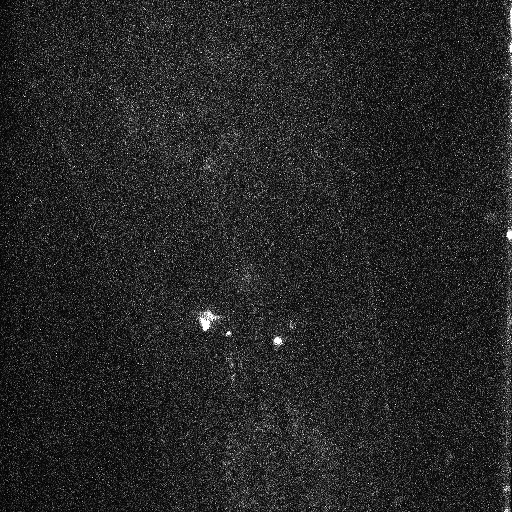

Supplement: Supplementary file 13 — Source data Fig. 4 [file 44318_2024_118_MOESM13_ESM.zip › Figure4/Figure 4C Micr. image/20230216 Phlh-17-ced-1; ced-1(e1735); G444E-gfp young adult_3/img_000000000_L-488_012.tif]

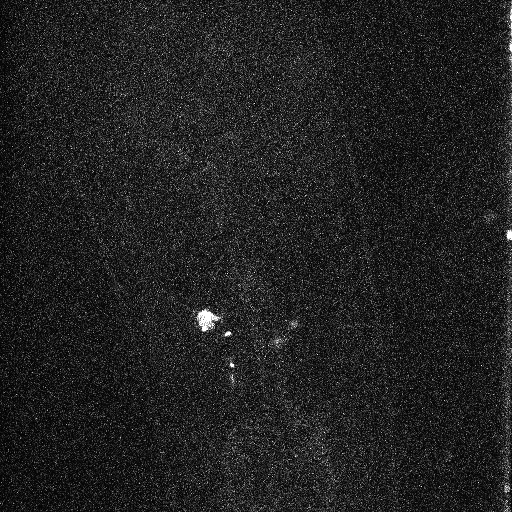

Supplement: Supplementary file 13 — Source data Fig. 4 [file 44318_2024_118_MOESM13_ESM.zip › Figure4/Figure 4C Micr. image/20230216 Phlh-17-ced-1; ced-1(e1735); G444E-gfp young adult_3/img_000000000_L-488_013.tif]

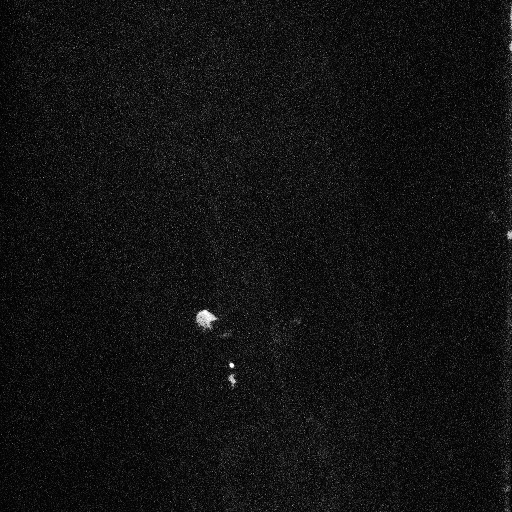

Supplement: Supplementary file 13 — Source data Fig. 4 [file 44318_2024_118_MOESM13_ESM.zip › Figure4/Figure 4C Micr. image/20230216 Phlh-17-ced-1; ced-1(e1735); G444E-gfp young adult_3/img_000000000_L-488_014.tif]

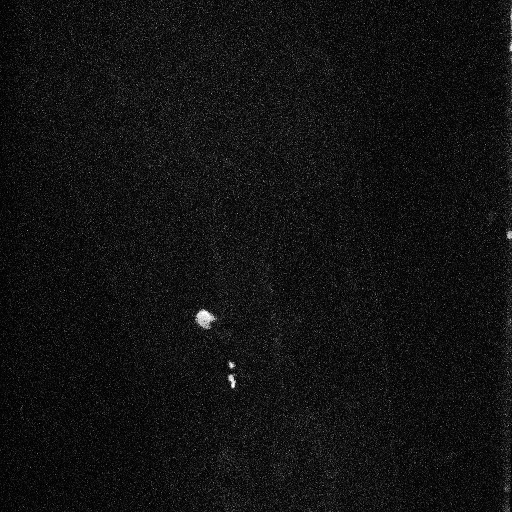

Supplement: Supplementary file 13 — Source data Fig. 4 [file 44318_2024_118_MOESM13_ESM.zip › Figure4/Figure 4C Micr. image/20230216 Phlh-17-ced-1; ced-1(e1735); G444E-gfp young adult_3/img_000000000_L-488_015.tif]

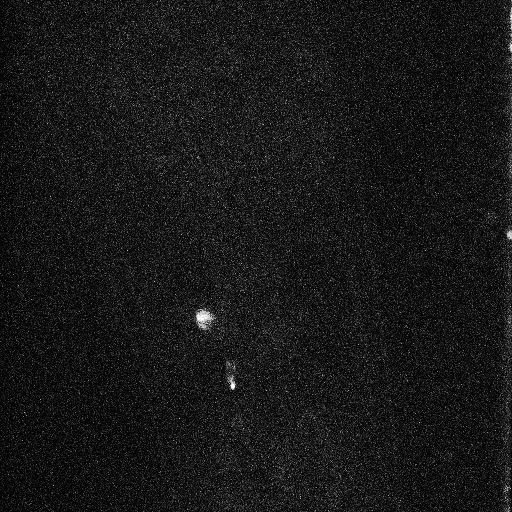

Supplement: Supplementary file 13 — Source data Fig. 4 [file 44318_2024_118_MOESM13_ESM.zip › Figure4/Figure 4C Micr. image/20230216 Phlh-17-ced-1; ced-1(e1735); G444E-gfp young adult_3/img_000000000_L-488_016.tif]

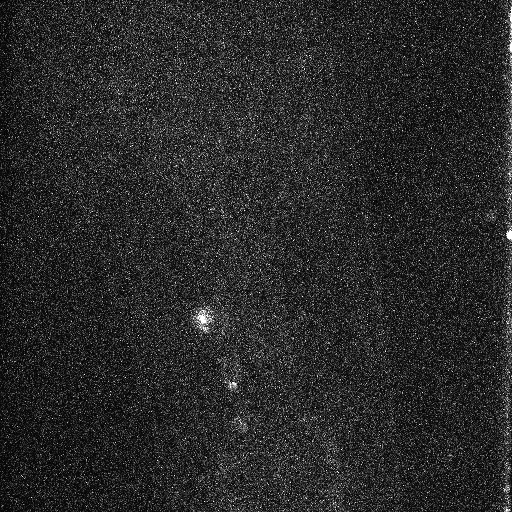

Supplement: Supplementary file 13 — Source data Fig. 4 [file 44318_2024_118_MOESM13_ESM.zip › Figure4/Figure 4C Micr. image/20230216 Phlh-17-ced-1; ced-1(e1735); G444E-gfp young adult_3/img_000000000_L-488_017.tif]

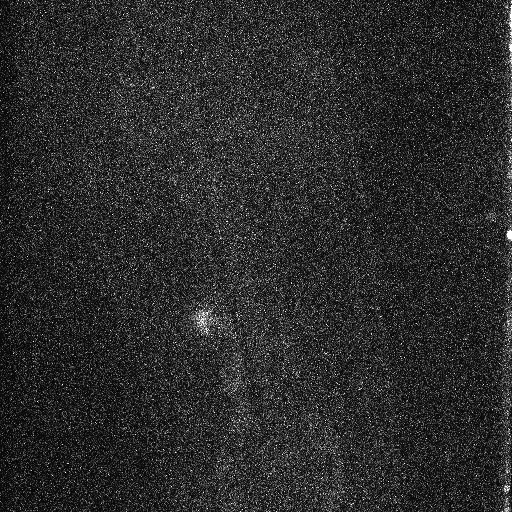

Supplement: Supplementary file 13 — Source data Fig. 4 [file 44318_2024_118_MOESM13_ESM.zip › Figure4/Figure 4C Micr. image/20230216 Phlh-17-ced-1; ced-1(e1735); G444E-gfp young adult_3/img_000000000_L-488_018.tif]

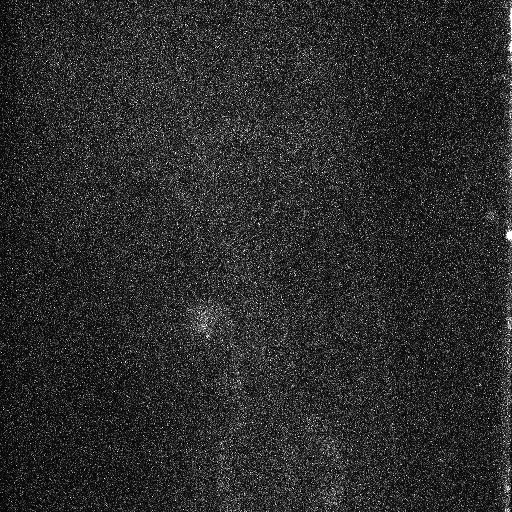

Supplement: Supplementary file 13 — Source data Fig. 4 [file 44318_2024_118_MOESM13_ESM.zip › Figure4/Figure 4C Micr. image/20230216 Phlh-17-ced-1; ced-1(e1735); G444E-gfp young adult_3/img_000000000_L-488_019.tif]

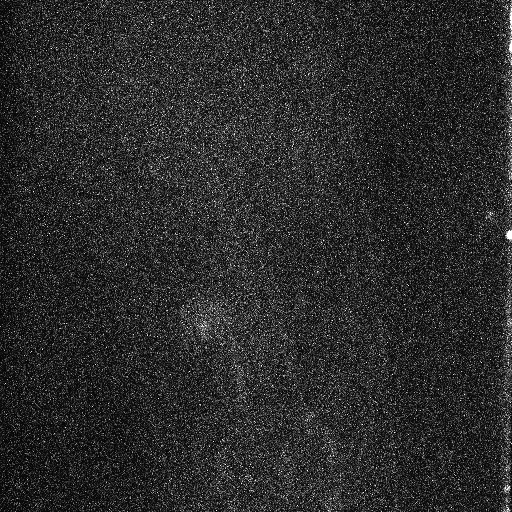

Supplement: Supplementary file 13 — Source data Fig. 4 [file 44318_2024_118_MOESM13_ESM.zip › Figure4/Figure 4C Micr. image/20230216 Phlh-17-ced-1; ced-1(e1735); G444E-gfp young adult_3/img_000000000_L-488_020.tif]

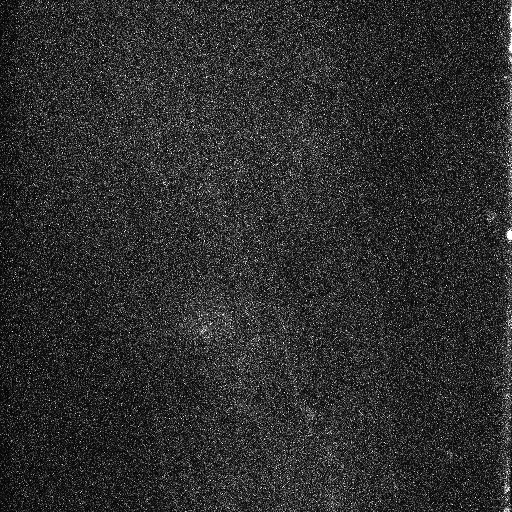

Supplement: Supplementary file 13 — Source data Fig. 4 [file 44318_2024_118_MOESM13_ESM.zip › Figure4/Figure 4C Micr. image/20230216 Phlh-17-ced-1; ced-1(e1735); G444E-gfp young adult_3/img_000000000_L-488_021.tif]

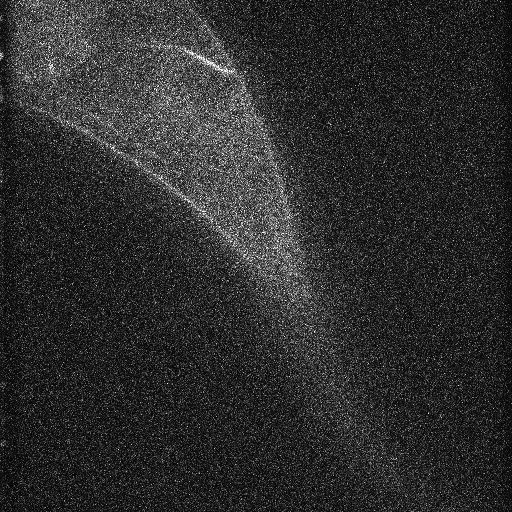

Supplement: Supplementary file 14 — Source data Fig. 5 [file 44318_2024_118_MOESM14_ESM.zip › Figure5/Figure 5C Micr. image/20211124 OSM-3-GFP KI_17/Pos0/img_000000000_Confocal-488-Acq_000.tif]

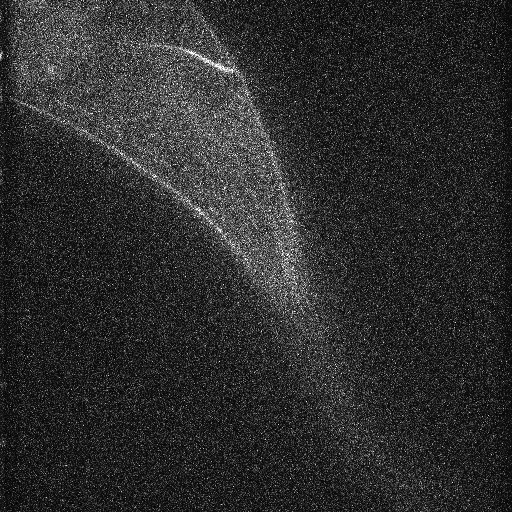

Supplement: Supplementary file 14 — Source data Fig. 5 [file 44318_2024_118_MOESM14_ESM.zip › Figure5/Figure 5C Micr. image/20211124 OSM-3-GFP KI_17/Pos0/img_000000000_Confocal-488-Acq_001.tif]

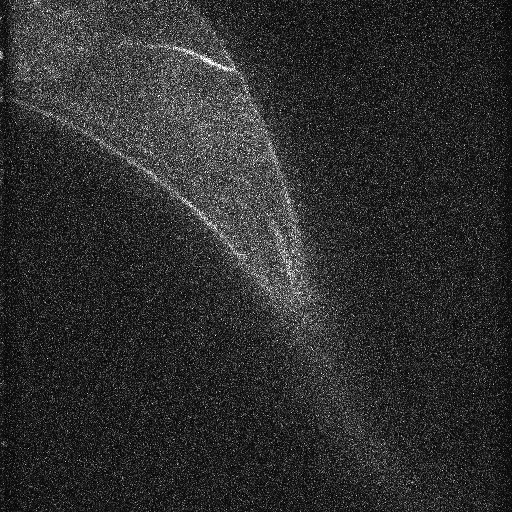

Supplement: Supplementary file 14 — Source data Fig. 5 [file 44318_2024_118_MOESM14_ESM.zip › Figure5/Figure 5C Micr. image/20211124 OSM-3-GFP KI_17/Pos0/img_000000000_Confocal-488-Acq_002.tif]

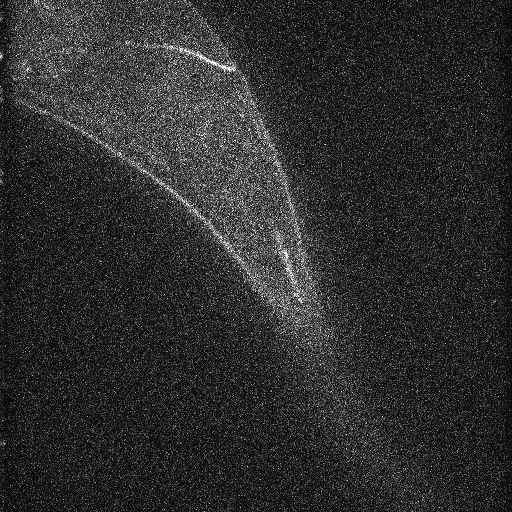

Supplement: Supplementary file 14 — Source data Fig. 5 [file 44318_2024_118_MOESM14_ESM.zip › Figure5/Figure 5C Micr. image/20211124 OSM-3-GFP KI_17/Pos0/img_000000000_Confocal-488-Acq_003.tif]

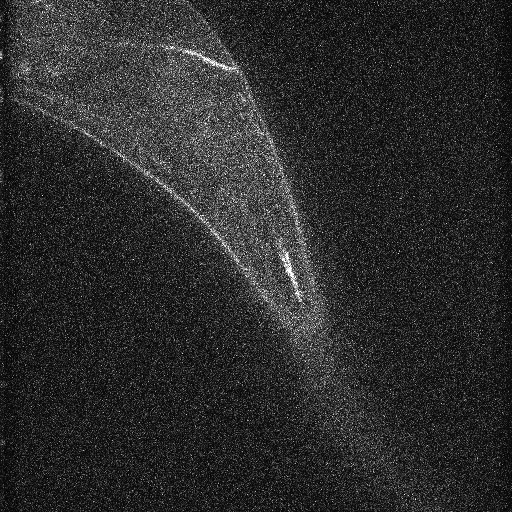

Supplement: Supplementary file 14 — Source data Fig. 5 [file 44318_2024_118_MOESM14_ESM.zip › Figure5/Figure 5C Micr. image/20211124 OSM-3-GFP KI_17/Pos0/img_000000000_Confocal-488-Acq_004.tif]

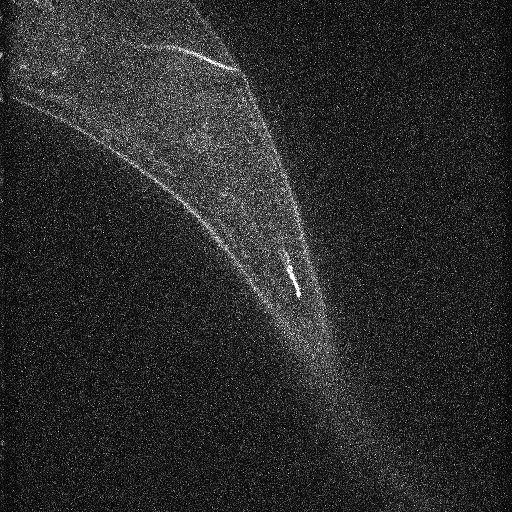

Supplement: Supplementary file 14 — Source data Fig. 5 [file 44318_2024_118_MOESM14_ESM.zip › Figure5/Figure 5C Micr. image/20211124 OSM-3-GFP KI_17/Pos0/img_000000000_Confocal-488-Acq_005.tif]

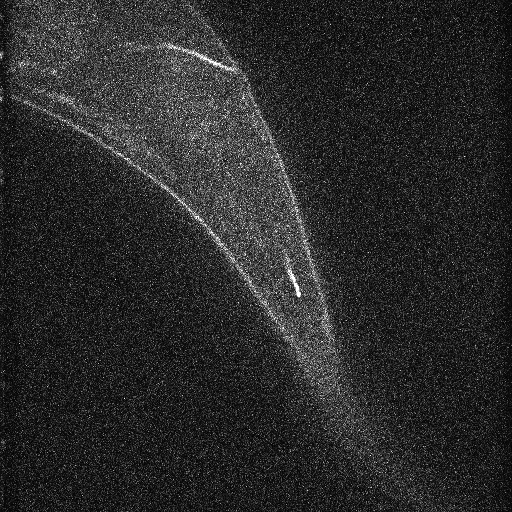

Supplement: Supplementary file 14 — Source data Fig. 5 [file 44318_2024_118_MOESM14_ESM.zip › Figure5/Figure 5C Micr. image/20211124 OSM-3-GFP KI_17/Pos0/img_000000000_Confocal-488-Acq_006.tif]

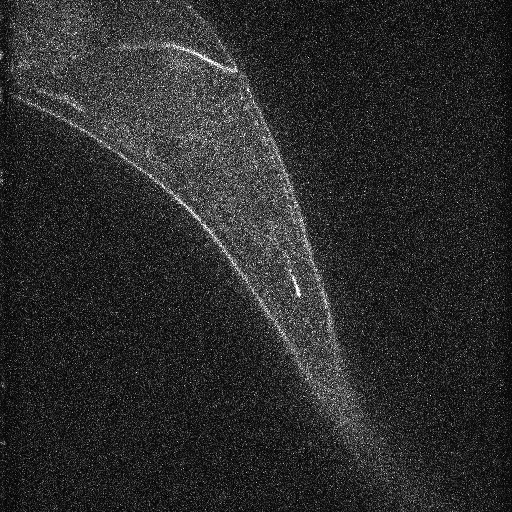

Supplement: Supplementary file 14 — Source data Fig. 5 [file 44318_2024_118_MOESM14_ESM.zip › Figure5/Figure 5C Micr. image/20211124 OSM-3-GFP KI_17/Pos0/img_000000000_Confocal-488-Acq_007.tif]

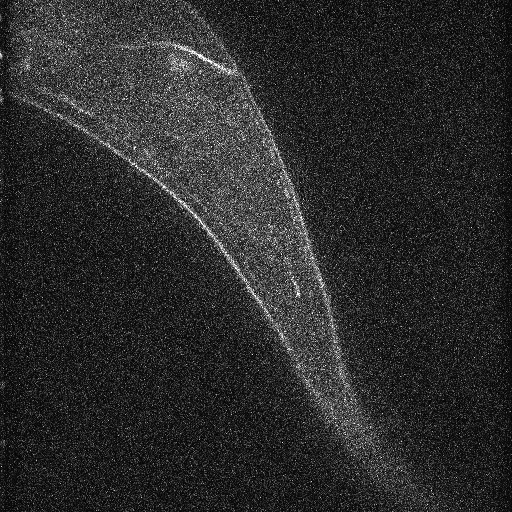

Supplement: Supplementary file 14 — Source data Fig. 5 [file 44318_2024_118_MOESM14_ESM.zip › Figure5/Figure 5C Micr. image/20211124 OSM-3-GFP KI_17/Pos0/img_000000000_Confocal-488-Acq_008.tif]

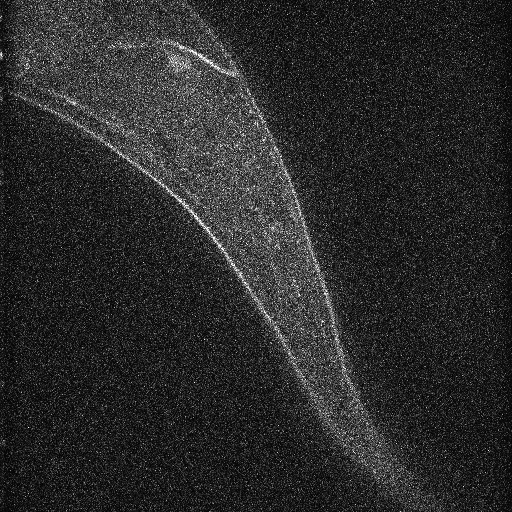

Supplement: Supplementary file 14 — Source data Fig. 5 [file 44318_2024_118_MOESM14_ESM.zip › Figure5/Figure 5C Micr. image/20211124 OSM-3-GFP KI_17/Pos0/img_000000000_Confocal-488-Acq_009.tif]

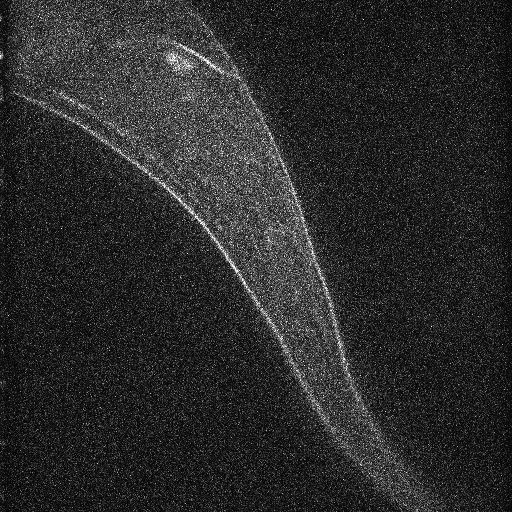

Supplement: Supplementary file 14 — Source data Fig. 5 [file 44318_2024_118_MOESM14_ESM.zip › Figure5/Figure 5C Micr. image/20211124 OSM-3-GFP KI_17/Pos0/img_000000000_Confocal-488-Acq_010.tif]

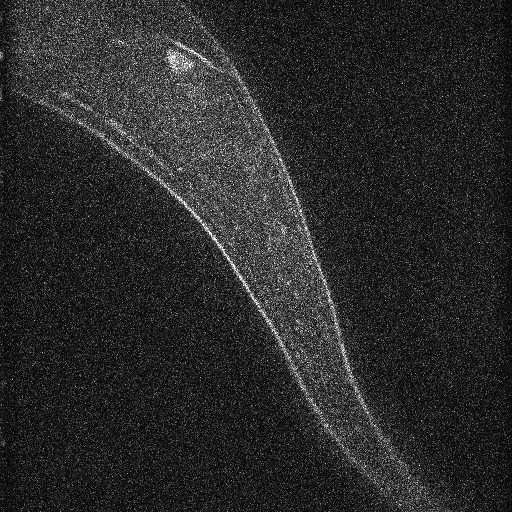

Supplement: Supplementary file 14 — Source data Fig. 5 [file 44318_2024_118_MOESM14_ESM.zip › Figure5/Figure 5C Micr. image/20211124 OSM-3-GFP KI_17/Pos0/img_000000000_Confocal-488-Acq_011.tif]

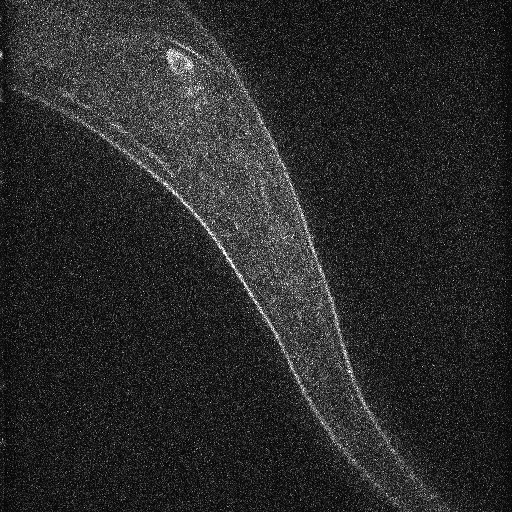

Supplement: Supplementary file 14 — Source data Fig. 5 [file 44318_2024_118_MOESM14_ESM.zip › Figure5/Figure 5C Micr. image/20211124 OSM-3-GFP KI_17/Pos0/img_000000000_Confocal-488-Acq_012.tif]

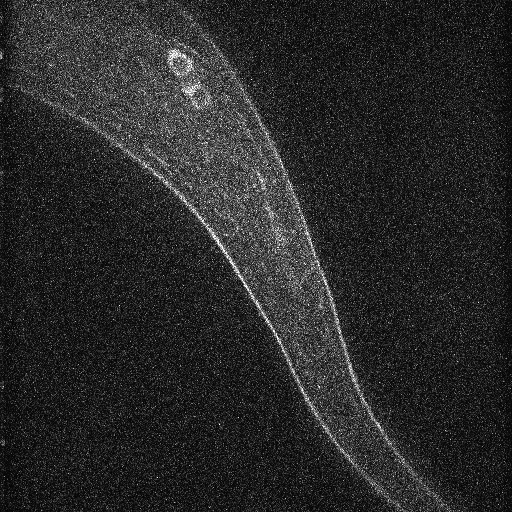

Supplement: Supplementary file 14 — Source data Fig. 5 [file 44318_2024_118_MOESM14_ESM.zip › Figure5/Figure 5C Micr. image/20211124 OSM-3-GFP KI_17/Pos0/img_000000000_Confocal-488-Acq_013.tif]

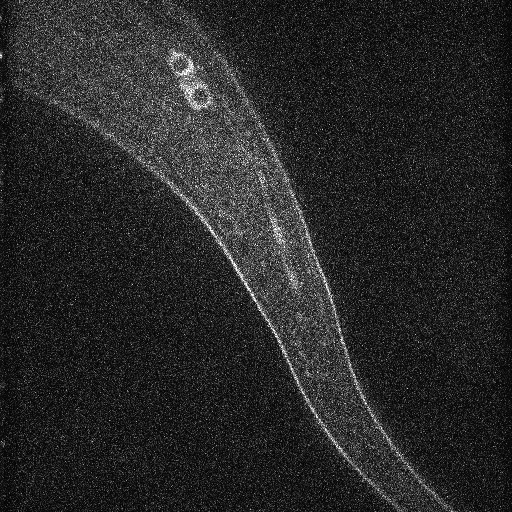

Supplement: Supplementary file 14 — Source data Fig. 5 [file 44318_2024_118_MOESM14_ESM.zip › Figure5/Figure 5C Micr. image/20211124 OSM-3-GFP KI_17/Pos0/img_000000000_Confocal-488-Acq_014.tif]

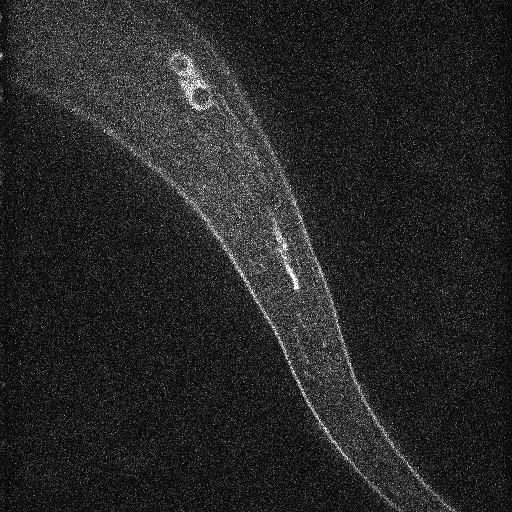

Supplement: Supplementary file 14 — Source data Fig. 5 [file 44318_2024_118_MOESM14_ESM.zip › Figure5/Figure 5C Micr. image/20211124 OSM-3-GFP KI_17/Pos0/img_000000000_Confocal-488-Acq_015.tif]

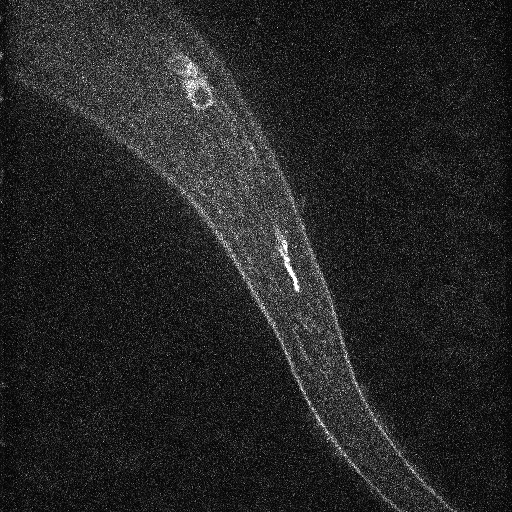

Supplement: Supplementary file 14 — Source data Fig. 5 [file 44318_2024_118_MOESM14_ESM.zip › Figure5/Figure 5C Micr. image/20211124 OSM-3-GFP KI_17/Pos0/img_000000000_Confocal-488-Acq_016.tif]

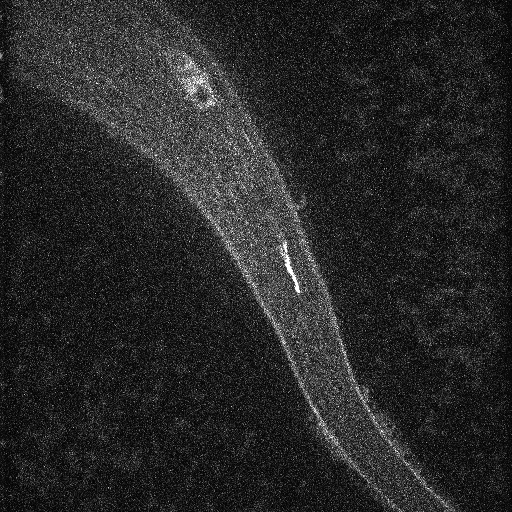

Supplement: Supplementary file 14 — Source data Fig. 5 [file 44318_2024_118_MOESM14_ESM.zip › Figure5/Figure 5C Micr. image/20211124 OSM-3-GFP KI_17/Pos0/img_000000000_Confocal-488-Acq_017.tif]

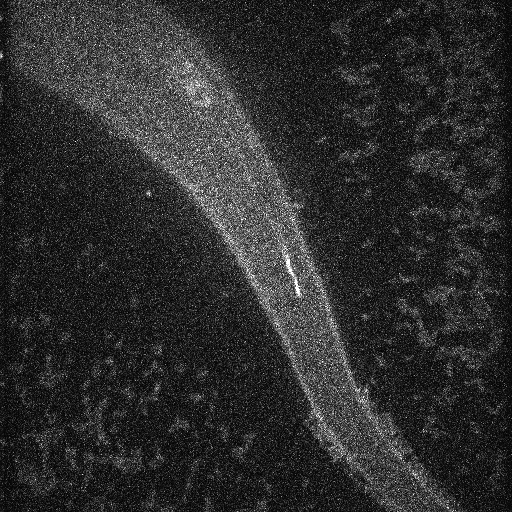

Supplement: Supplementary file 14 — Source data Fig. 5 [file 44318_2024_118_MOESM14_ESM.zip › Figure5/Figure 5C Micr. image/20211124 OSM-3-GFP KI_17/Pos0/img_000000000_Confocal-488-Acq_018.tif]

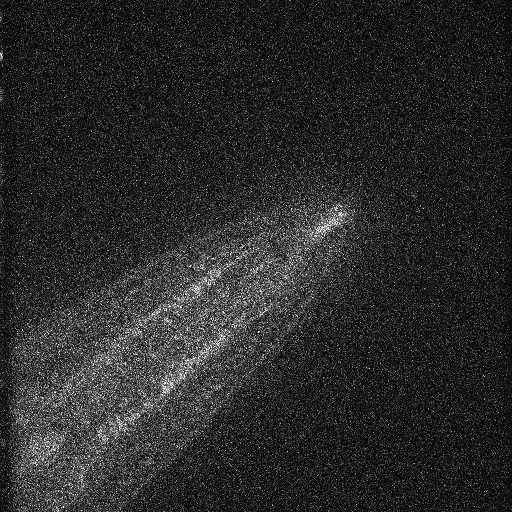

Supplement: Supplementary file 14 — Source data Fig. 5 [file 44318_2024_118_MOESM14_ESM.zip › Figure5/Figure 5C Micr. image/20211124 OSM-3-GFP KI_25/Pos0/img_000000000_Confocal-488-Acq_000.tif]

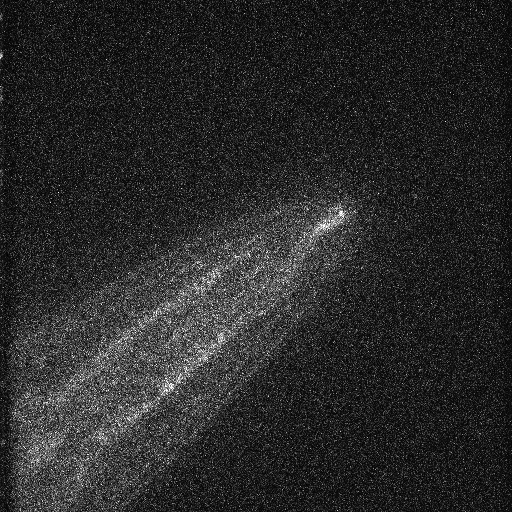

Supplement: Supplementary file 14 — Source data Fig. 5 [file 44318_2024_118_MOESM14_ESM.zip › Figure5/Figure 5C Micr. image/20211124 OSM-3-GFP KI_25/Pos0/img_000000000_Confocal-488-Acq_001.tif]

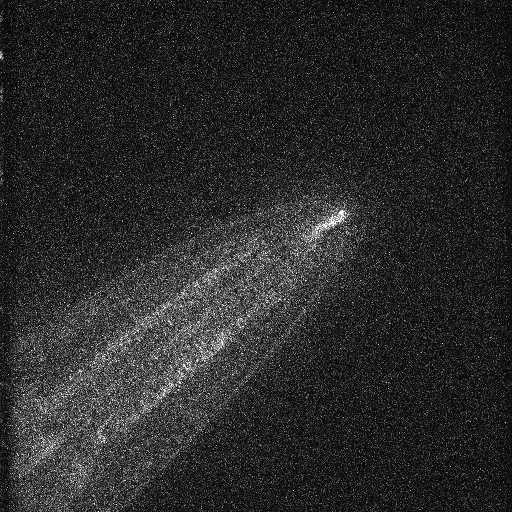

Supplement: Supplementary file 14 — Source data Fig. 5 [file 44318_2024_118_MOESM14_ESM.zip › Figure5/Figure 5C Micr. image/20211124 OSM-3-GFP KI_25/Pos0/img_000000000_Confocal-488-Acq_002.tif]

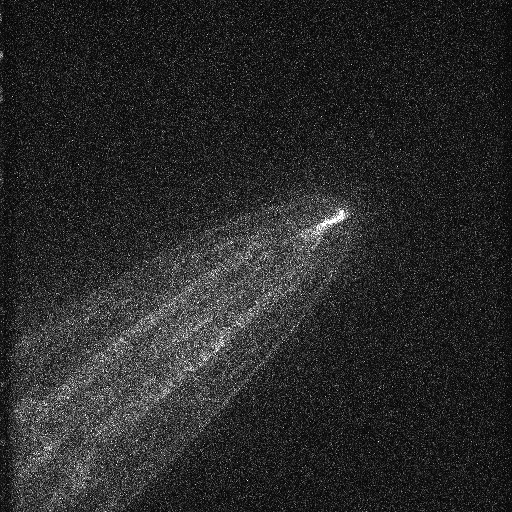

Supplement: Supplementary file 14 — Source data Fig. 5 [file 44318_2024_118_MOESM14_ESM.zip › Figure5/Figure 5C Micr. image/20211124 OSM-3-GFP KI_25/Pos0/img_000000000_Confocal-488-Acq_003.tif]

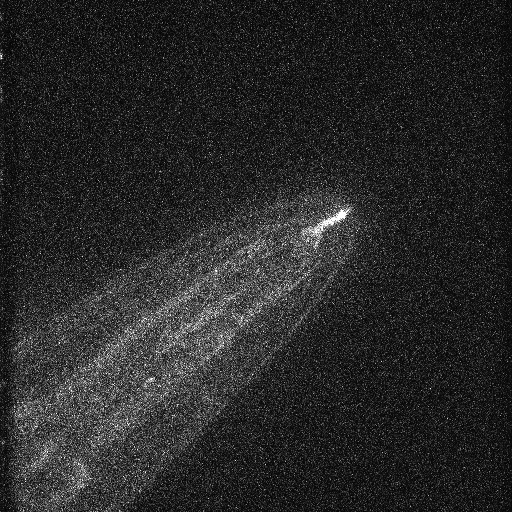

Supplement: Supplementary file 14 — Source data Fig. 5 [file 44318_2024_118_MOESM14_ESM.zip › Figure5/Figure 5C Micr. image/20211124 OSM-3-GFP KI_25/Pos0/img_000000000_Confocal-488-Acq_004.tif]

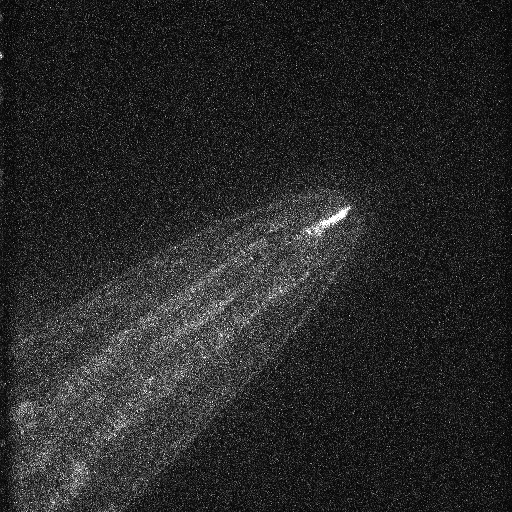

Supplement: Supplementary file 14 — Source data Fig. 5 [file 44318_2024_118_MOESM14_ESM.zip › Figure5/Figure 5C Micr. image/20211124 OSM-3-GFP KI_25/Pos0/img_000000000_Confocal-488-Acq_005.tif]

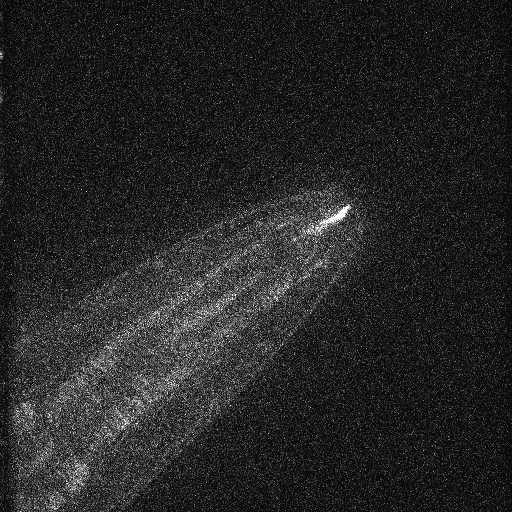

Supplement: Supplementary file 14 — Source data Fig. 5 [file 44318_2024_118_MOESM14_ESM.zip › Figure5/Figure 5C Micr. image/20211124 OSM-3-GFP KI_25/Pos0/img_000000000_Confocal-488-Acq_006.tif]

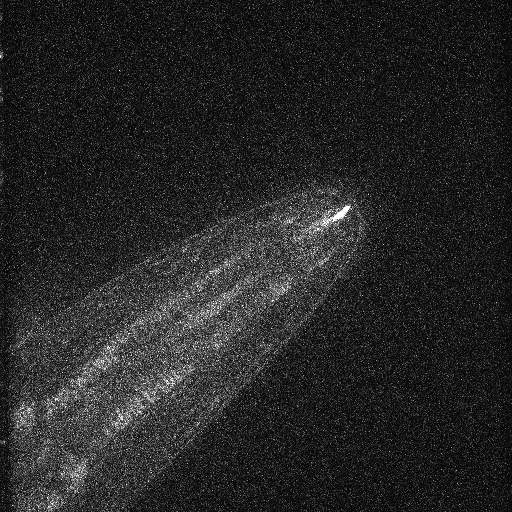

Supplement: Supplementary file 14 — Source data Fig. 5 [file 44318_2024_118_MOESM14_ESM.zip › Figure5/Figure 5C Micr. image/20211124 OSM-3-GFP KI_25/Pos0/img_000000000_Confocal-488-Acq_007.tif]

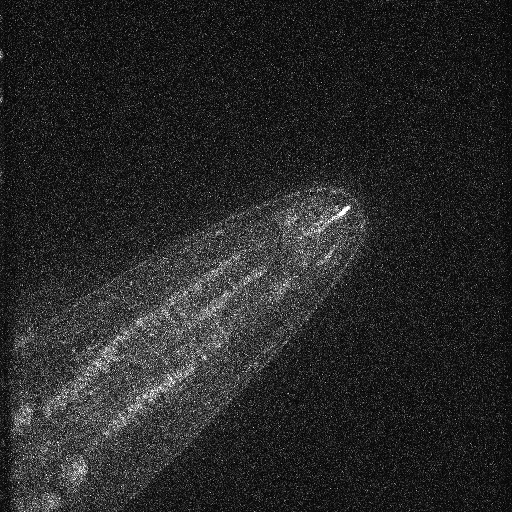

Supplement: Supplementary file 14 — Source data Fig. 5 [file 44318_2024_118_MOESM14_ESM.zip › Figure5/Figure 5C Micr. image/20211124 OSM-3-GFP KI_25/Pos0/img_000000000_Confocal-488-Acq_008.tif]

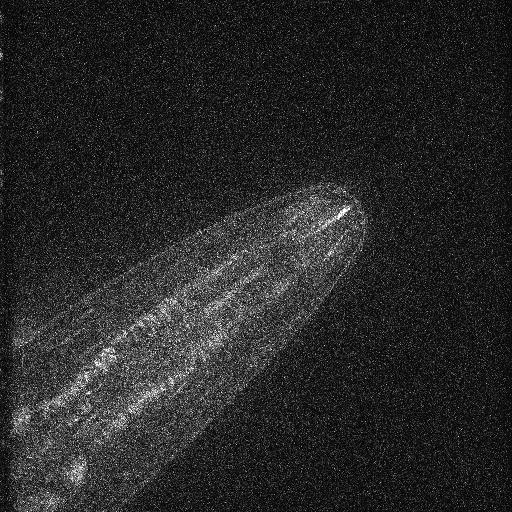

Supplement: Supplementary file 14 — Source data Fig. 5 [file 44318_2024_118_MOESM14_ESM.zip › Figure5/Figure 5C Micr. image/20211124 OSM-3-GFP KI_25/Pos0/img_000000000_Confocal-488-Acq_009.tif]
