# Supplementary figures and images for: Neurons dispose of hyperactive kinesin into glial cells for clearance (part 5 of 9)
Source: EMBO J. 2024 May 28;43(13):5. doi: 10.1038/s44318-024-00118-0 (PMC11217292; doi:10.1038/s44318-024-00118-0)

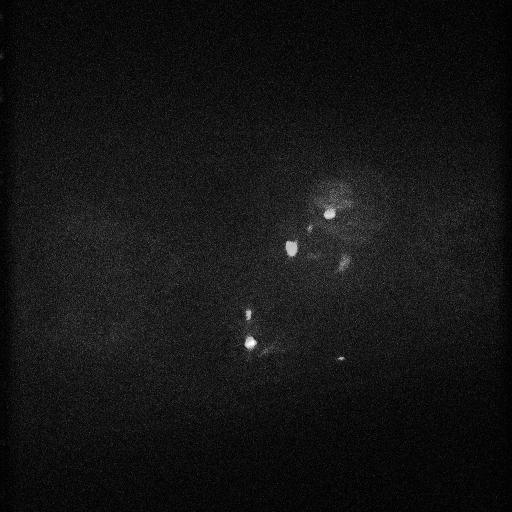

Supplement: Supplementary file 13 — Source data Fig. 4 [file 44318_2024_118_MOESM13_ESM.zip › Figure4/Figure 4B Micr. image/20210908 Phlh-17-mCherry; osm-3-g444e-gfp100x_5/Pos0/img_000000000_Confocal-488-Acq_020.tif]

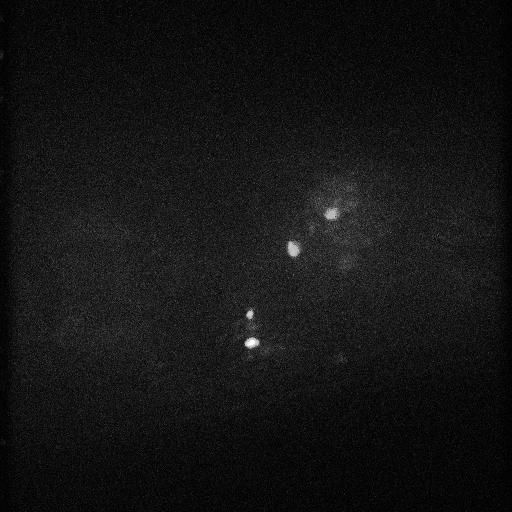

Supplement: Supplementary file 13 — Source data Fig. 4 [file 44318_2024_118_MOESM13_ESM.zip › Figure4/Figure 4B Micr. image/20210908 Phlh-17-mCherry; osm-3-g444e-gfp100x_5/Pos0/img_000000000_Confocal-488-Acq_021.tif]

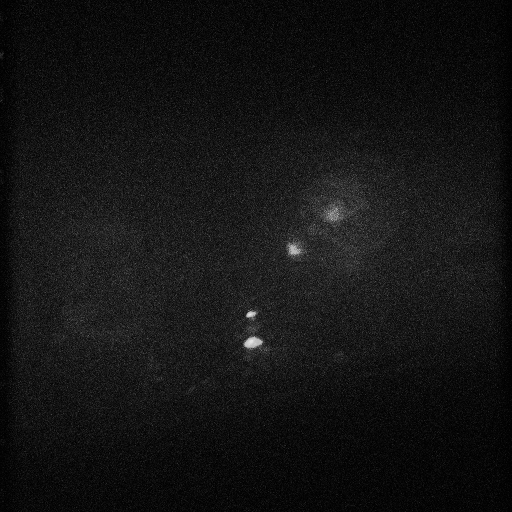

Supplement: Supplementary file 13 — Source data Fig. 4 [file 44318_2024_118_MOESM13_ESM.zip › Figure4/Figure 4B Micr. image/20210908 Phlh-17-mCherry; osm-3-g444e-gfp100x_5/Pos0/img_000000000_Confocal-488-Acq_022.tif]

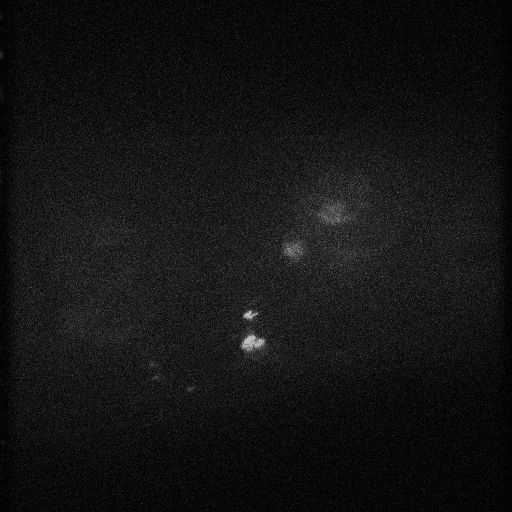

Supplement: Supplementary file 13 — Source data Fig. 4 [file 44318_2024_118_MOESM13_ESM.zip › Figure4/Figure 4B Micr. image/20210908 Phlh-17-mCherry; osm-3-g444e-gfp100x_5/Pos0/img_000000000_Confocal-488-Acq_023.tif]

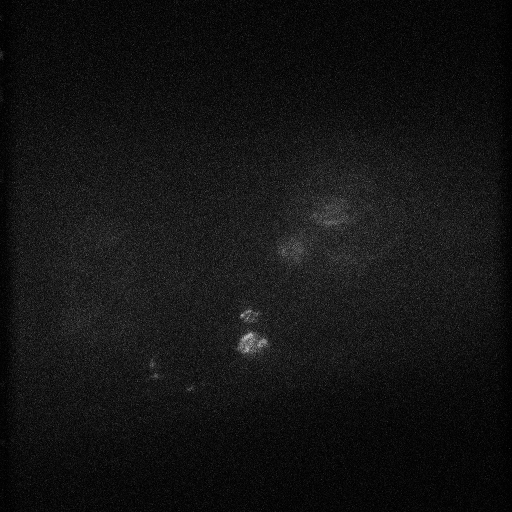

Supplement: Supplementary file 13 — Source data Fig. 4 [file 44318_2024_118_MOESM13_ESM.zip › Figure4/Figure 4B Micr. image/20210908 Phlh-17-mCherry; osm-3-g444e-gfp100x_5/Pos0/img_000000000_Confocal-488-Acq_024.tif]

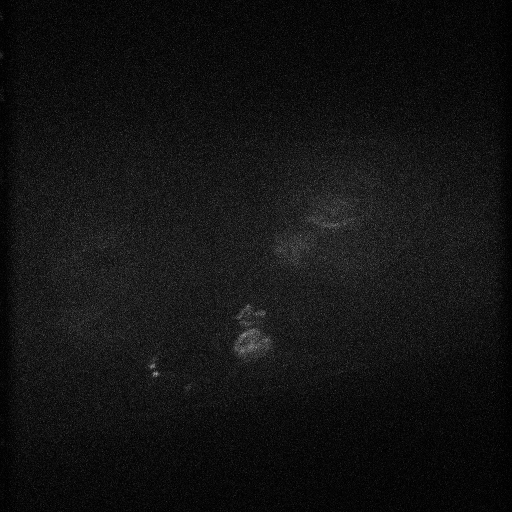

Supplement: Supplementary file 13 — Source data Fig. 4 [file 44318_2024_118_MOESM13_ESM.zip › Figure4/Figure 4B Micr. image/20210908 Phlh-17-mCherry; osm-3-g444e-gfp100x_5/Pos0/img_000000000_Confocal-488-Acq_025.tif]

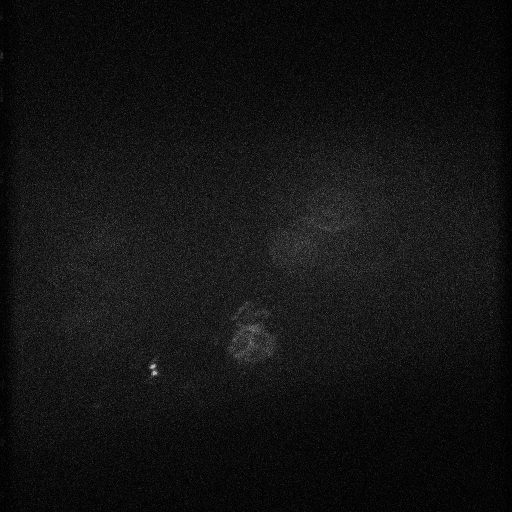

Supplement: Supplementary file 13 — Source data Fig. 4 [file 44318_2024_118_MOESM13_ESM.zip › Figure4/Figure 4B Micr. image/20210908 Phlh-17-mCherry; osm-3-g444e-gfp100x_5/Pos0/img_000000000_Confocal-488-Acq_026.tif]

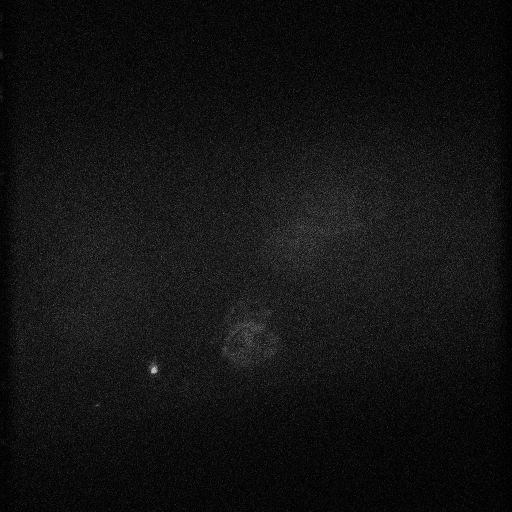

Supplement: Supplementary file 13 — Source data Fig. 4 [file 44318_2024_118_MOESM13_ESM.zip › Figure4/Figure 4B Micr. image/20210908 Phlh-17-mCherry; osm-3-g444e-gfp100x_5/Pos0/img_000000000_Confocal-488-Acq_027.tif]

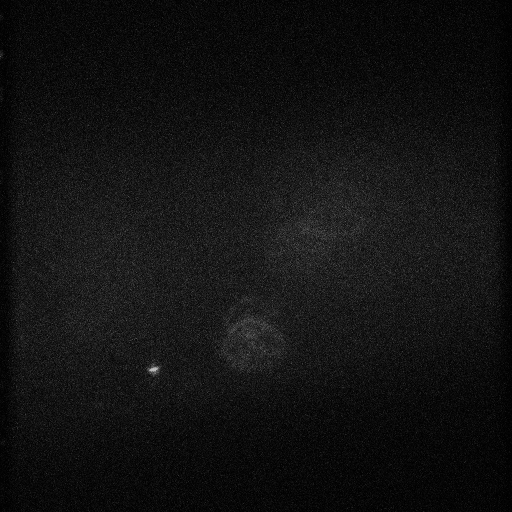

Supplement: Supplementary file 13 — Source data Fig. 4 [file 44318_2024_118_MOESM13_ESM.zip › Figure4/Figure 4B Micr. image/20210908 Phlh-17-mCherry; osm-3-g444e-gfp100x_5/Pos0/img_000000000_Confocal-488-Acq_028.tif]

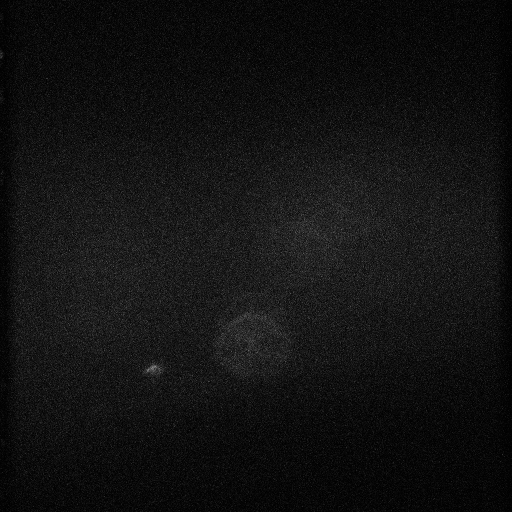

Supplement: Supplementary file 13 — Source data Fig. 4 [file 44318_2024_118_MOESM13_ESM.zip › Figure4/Figure 4B Micr. image/20210908 Phlh-17-mCherry; osm-3-g444e-gfp100x_5/Pos0/img_000000000_Confocal-488-Acq_029.tif]

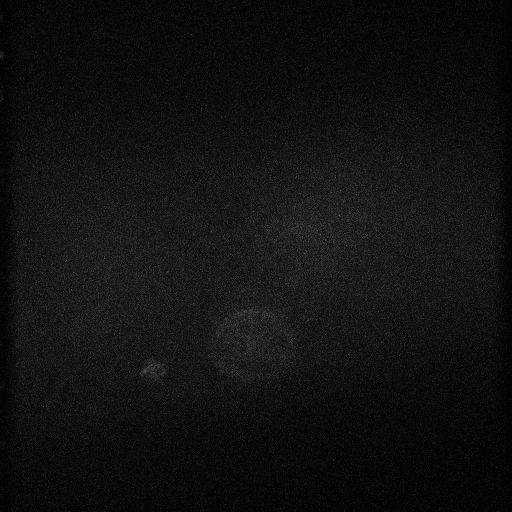

Supplement: Supplementary file 13 — Source data Fig. 4 [file 44318_2024_118_MOESM13_ESM.zip › Figure4/Figure 4B Micr. image/20210908 Phlh-17-mCherry; osm-3-g444e-gfp100x_5/Pos0/img_000000000_Confocal-488-Acq_030.tif]

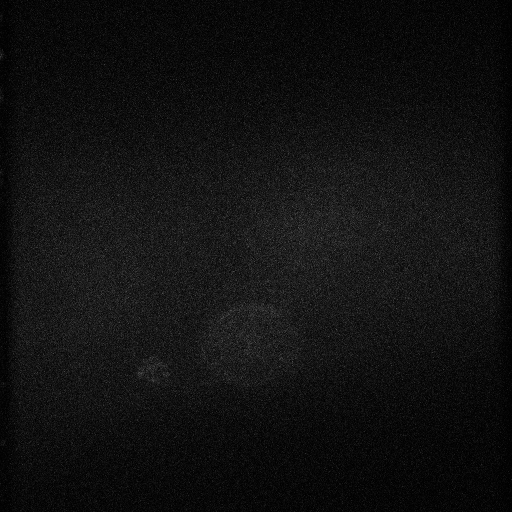

Supplement: Supplementary file 13 — Source data Fig. 4 [file 44318_2024_118_MOESM13_ESM.zip › Figure4/Figure 4B Micr. image/20210908 Phlh-17-mCherry; osm-3-g444e-gfp100x_5/Pos0/img_000000000_Confocal-488-Acq_031.tif]

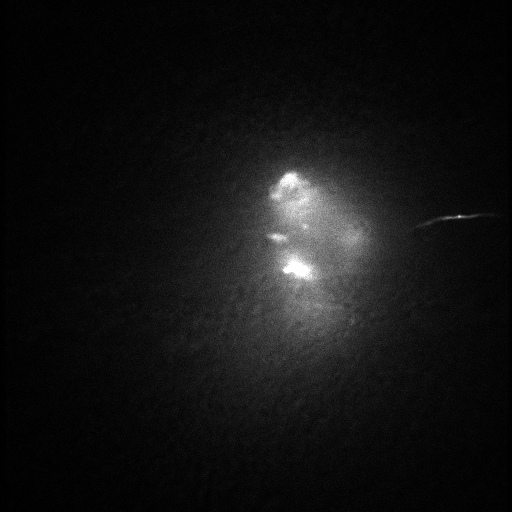

Supplement: Supplementary file 13 — Source data Fig. 4 [file 44318_2024_118_MOESM13_ESM.zip › Figure4/Figure 4B Micr. image/20210908 Phlh-17-mCherry; osm-3-g444e-gfp100x_5/Pos0/img_000000000_Confocal-561_000.tif]

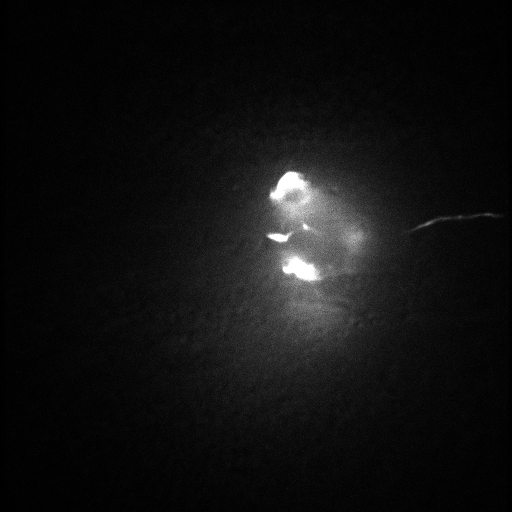

Supplement: Supplementary file 13 — Source data Fig. 4 [file 44318_2024_118_MOESM13_ESM.zip › Figure4/Figure 4B Micr. image/20210908 Phlh-17-mCherry; osm-3-g444e-gfp100x_5/Pos0/img_000000000_Confocal-561_001.tif]

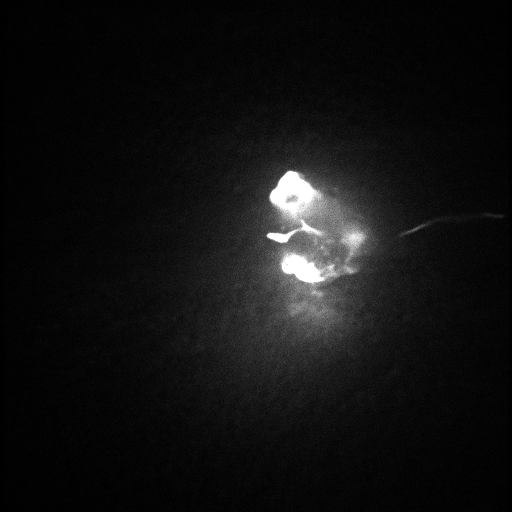

Supplement: Supplementary file 13 — Source data Fig. 4 [file 44318_2024_118_MOESM13_ESM.zip › Figure4/Figure 4B Micr. image/20210908 Phlh-17-mCherry; osm-3-g444e-gfp100x_5/Pos0/img_000000000_Confocal-561_002.tif]

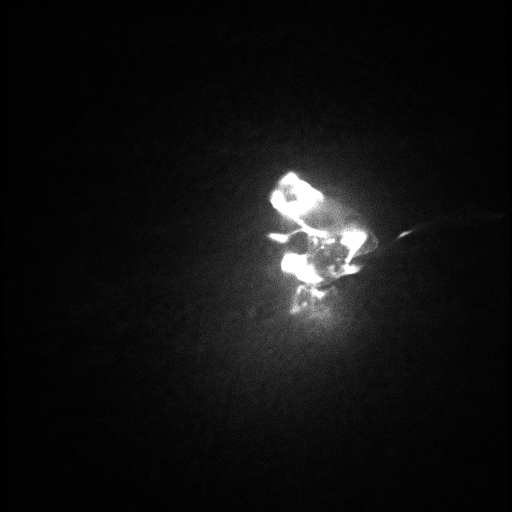

Supplement: Supplementary file 13 — Source data Fig. 4 [file 44318_2024_118_MOESM13_ESM.zip › Figure4/Figure 4B Micr. image/20210908 Phlh-17-mCherry; osm-3-g444e-gfp100x_5/Pos0/img_000000000_Confocal-561_003.tif]

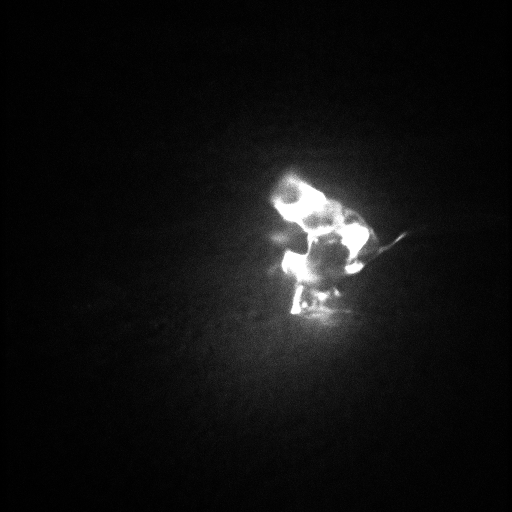

Supplement: Supplementary file 13 — Source data Fig. 4 [file 44318_2024_118_MOESM13_ESM.zip › Figure4/Figure 4B Micr. image/20210908 Phlh-17-mCherry; osm-3-g444e-gfp100x_5/Pos0/img_000000000_Confocal-561_004.tif]

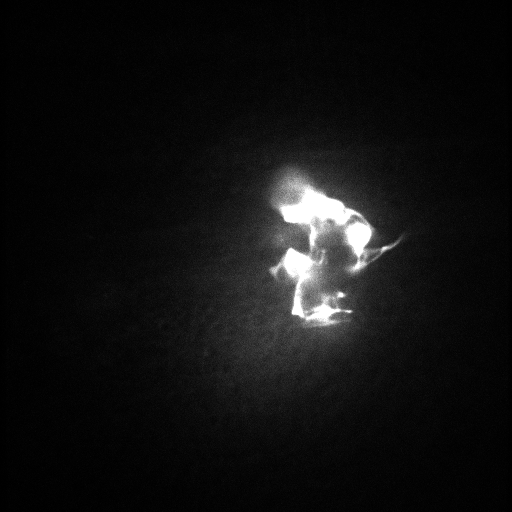

Supplement: Supplementary file 13 — Source data Fig. 4 [file 44318_2024_118_MOESM13_ESM.zip › Figure4/Figure 4B Micr. image/20210908 Phlh-17-mCherry; osm-3-g444e-gfp100x_5/Pos0/img_000000000_Confocal-561_005.tif]

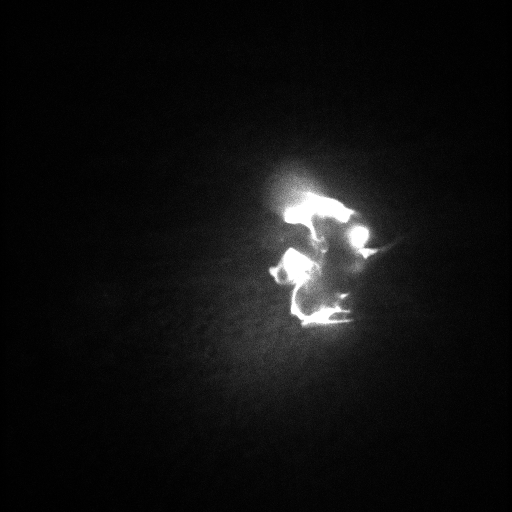

Supplement: Supplementary file 13 — Source data Fig. 4 [file 44318_2024_118_MOESM13_ESM.zip › Figure4/Figure 4B Micr. image/20210908 Phlh-17-mCherry; osm-3-g444e-gfp100x_5/Pos0/img_000000000_Confocal-561_006.tif]

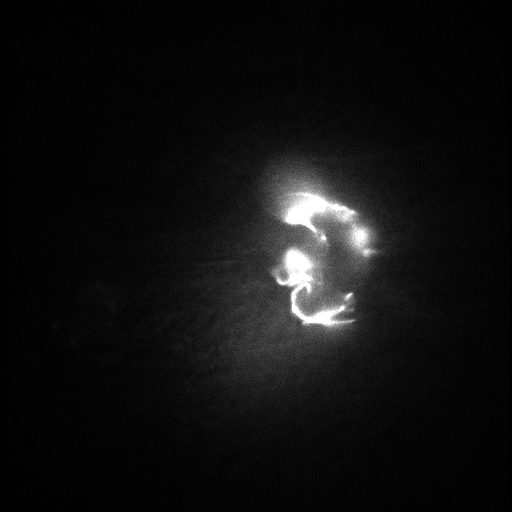

Supplement: Supplementary file 13 — Source data Fig. 4 [file 44318_2024_118_MOESM13_ESM.zip › Figure4/Figure 4B Micr. image/20210908 Phlh-17-mCherry; osm-3-g444e-gfp100x_5/Pos0/img_000000000_Confocal-561_007.tif]

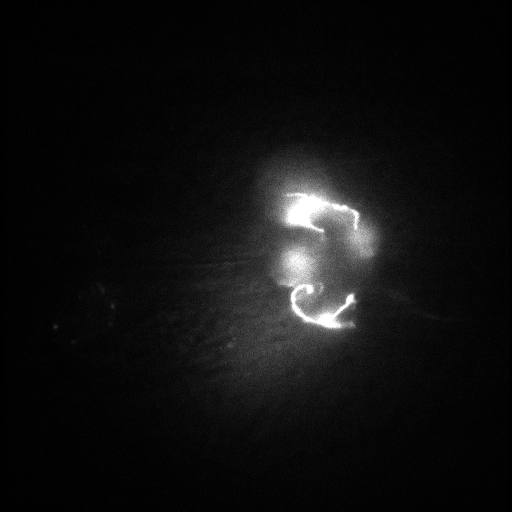

Supplement: Supplementary file 13 — Source data Fig. 4 [file 44318_2024_118_MOESM13_ESM.zip › Figure4/Figure 4B Micr. image/20210908 Phlh-17-mCherry; osm-3-g444e-gfp100x_5/Pos0/img_000000000_Confocal-561_008.tif]

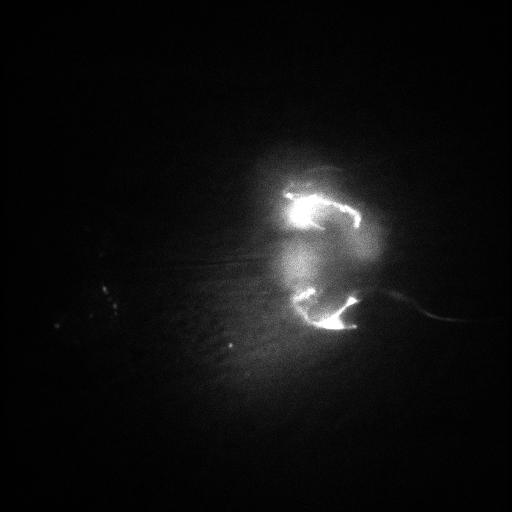

Supplement: Supplementary file 13 — Source data Fig. 4 [file 44318_2024_118_MOESM13_ESM.zip › Figure4/Figure 4B Micr. image/20210908 Phlh-17-mCherry; osm-3-g444e-gfp100x_5/Pos0/img_000000000_Confocal-561_009.tif]

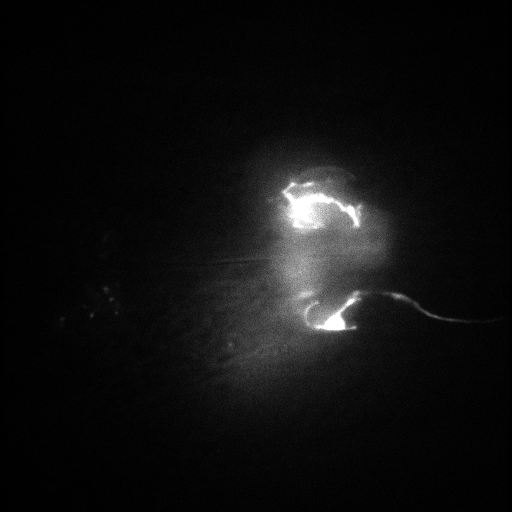

Supplement: Supplementary file 13 — Source data Fig. 4 [file 44318_2024_118_MOESM13_ESM.zip › Figure4/Figure 4B Micr. image/20210908 Phlh-17-mCherry; osm-3-g444e-gfp100x_5/Pos0/img_000000000_Confocal-561_010.tif]

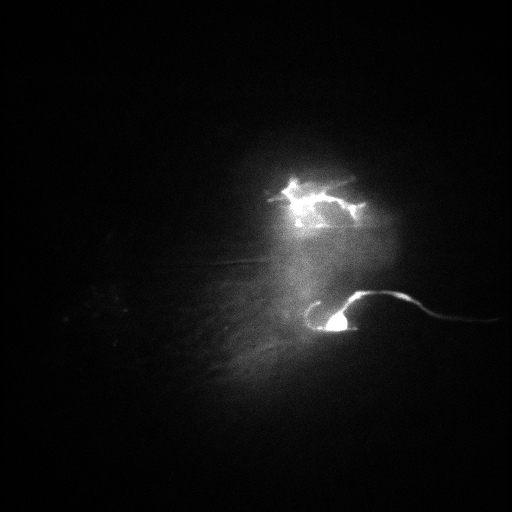

Supplement: Supplementary file 13 — Source data Fig. 4 [file 44318_2024_118_MOESM13_ESM.zip › Figure4/Figure 4B Micr. image/20210908 Phlh-17-mCherry; osm-3-g444e-gfp100x_5/Pos0/img_000000000_Confocal-561_011.tif]

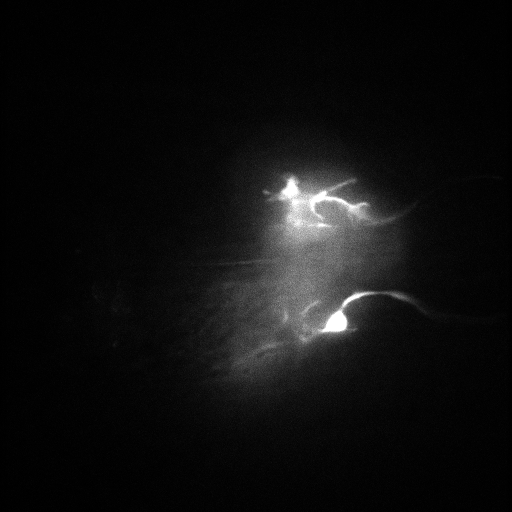

Supplement: Supplementary file 13 — Source data Fig. 4 [file 44318_2024_118_MOESM13_ESM.zip › Figure4/Figure 4B Micr. image/20210908 Phlh-17-mCherry; osm-3-g444e-gfp100x_5/Pos0/img_000000000_Confocal-561_012.tif]

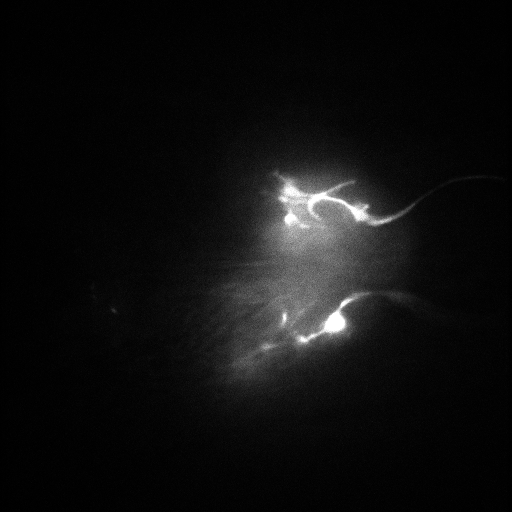

Supplement: Supplementary file 13 — Source data Fig. 4 [file 44318_2024_118_MOESM13_ESM.zip › Figure4/Figure 4B Micr. image/20210908 Phlh-17-mCherry; osm-3-g444e-gfp100x_5/Pos0/img_000000000_Confocal-561_013.tif]

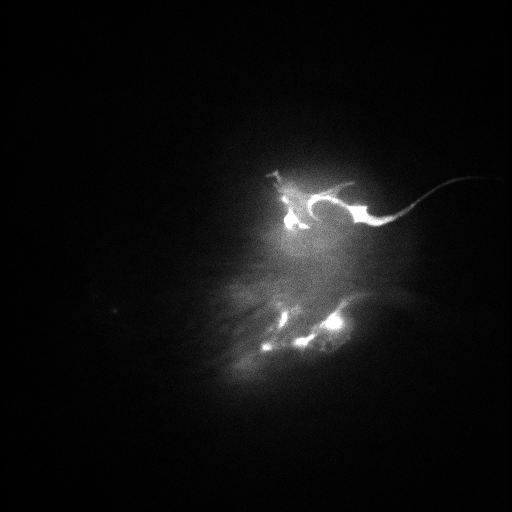

Supplement: Supplementary file 13 — Source data Fig. 4 [file 44318_2024_118_MOESM13_ESM.zip › Figure4/Figure 4B Micr. image/20210908 Phlh-17-mCherry; osm-3-g444e-gfp100x_5/Pos0/img_000000000_Confocal-561_014.tif]

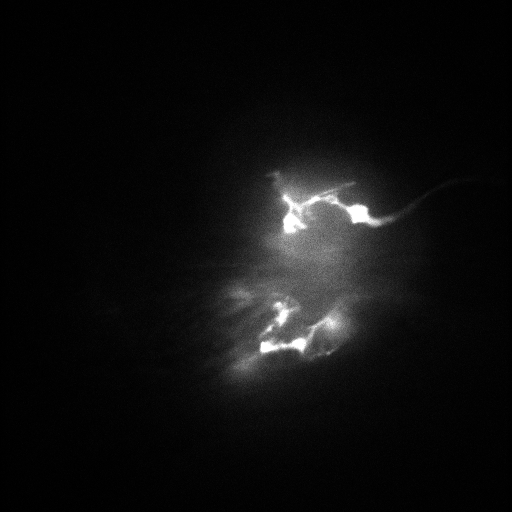

Supplement: Supplementary file 13 — Source data Fig. 4 [file 44318_2024_118_MOESM13_ESM.zip › Figure4/Figure 4B Micr. image/20210908 Phlh-17-mCherry; osm-3-g444e-gfp100x_5/Pos0/img_000000000_Confocal-561_015.tif]

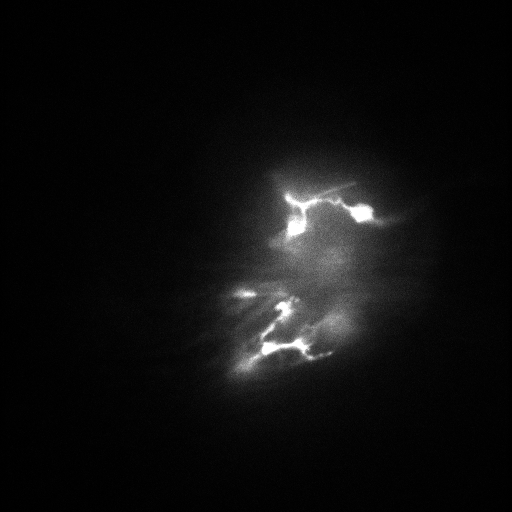

Supplement: Supplementary file 13 — Source data Fig. 4 [file 44318_2024_118_MOESM13_ESM.zip › Figure4/Figure 4B Micr. image/20210908 Phlh-17-mCherry; osm-3-g444e-gfp100x_5/Pos0/img_000000000_Confocal-561_016.tif]

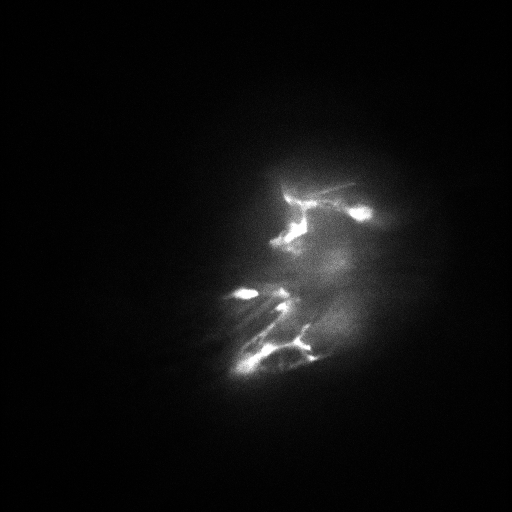

Supplement: Supplementary file 13 — Source data Fig. 4 [file 44318_2024_118_MOESM13_ESM.zip › Figure4/Figure 4B Micr. image/20210908 Phlh-17-mCherry; osm-3-g444e-gfp100x_5/Pos0/img_000000000_Confocal-561_017.tif]

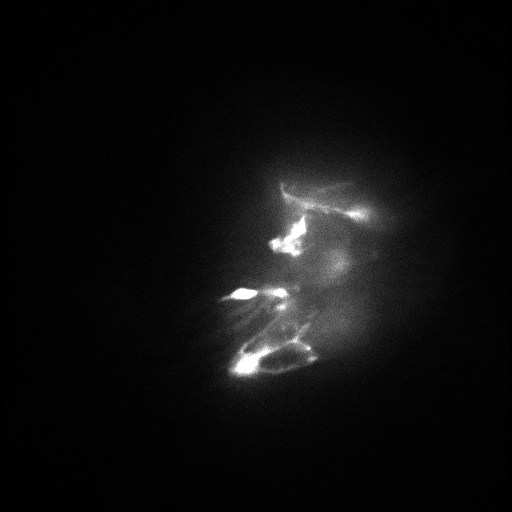

Supplement: Supplementary file 13 — Source data Fig. 4 [file 44318_2024_118_MOESM13_ESM.zip › Figure4/Figure 4B Micr. image/20210908 Phlh-17-mCherry; osm-3-g444e-gfp100x_5/Pos0/img_000000000_Confocal-561_018.tif]

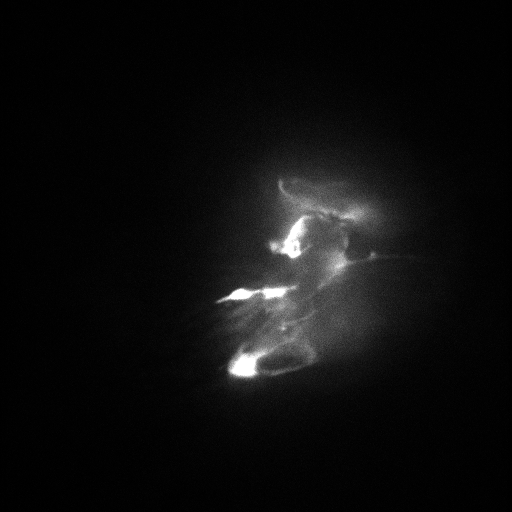

Supplement: Supplementary file 13 — Source data Fig. 4 [file 44318_2024_118_MOESM13_ESM.zip › Figure4/Figure 4B Micr. image/20210908 Phlh-17-mCherry; osm-3-g444e-gfp100x_5/Pos0/img_000000000_Confocal-561_019.tif]

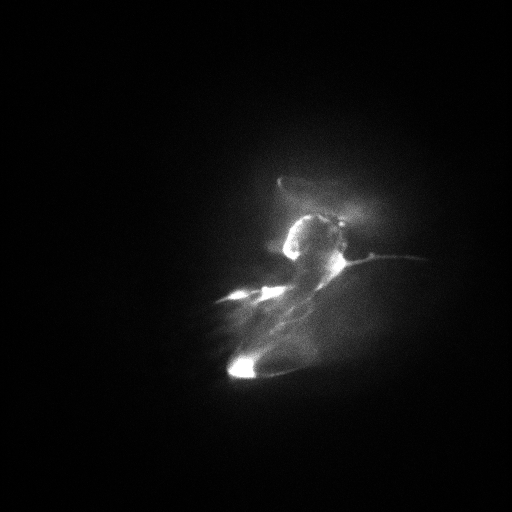

Supplement: Supplementary file 13 — Source data Fig. 4 [file 44318_2024_118_MOESM13_ESM.zip › Figure4/Figure 4B Micr. image/20210908 Phlh-17-mCherry; osm-3-g444e-gfp100x_5/Pos0/img_000000000_Confocal-561_020.tif]

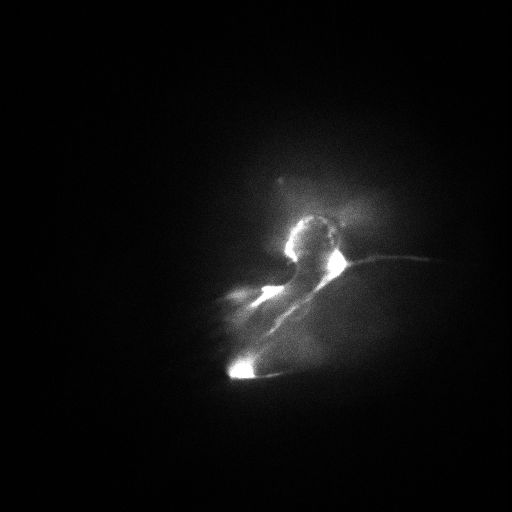

Supplement: Supplementary file 13 — Source data Fig. 4 [file 44318_2024_118_MOESM13_ESM.zip › Figure4/Figure 4B Micr. image/20210908 Phlh-17-mCherry; osm-3-g444e-gfp100x_5/Pos0/img_000000000_Confocal-561_021.tif]

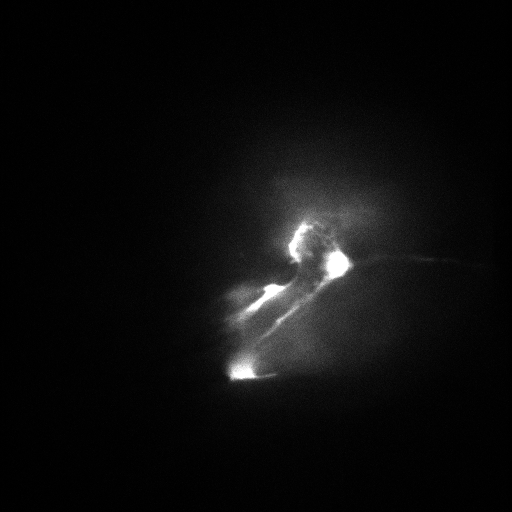

Supplement: Supplementary file 13 — Source data Fig. 4 [file 44318_2024_118_MOESM13_ESM.zip › Figure4/Figure 4B Micr. image/20210908 Phlh-17-mCherry; osm-3-g444e-gfp100x_5/Pos0/img_000000000_Confocal-561_022.tif]

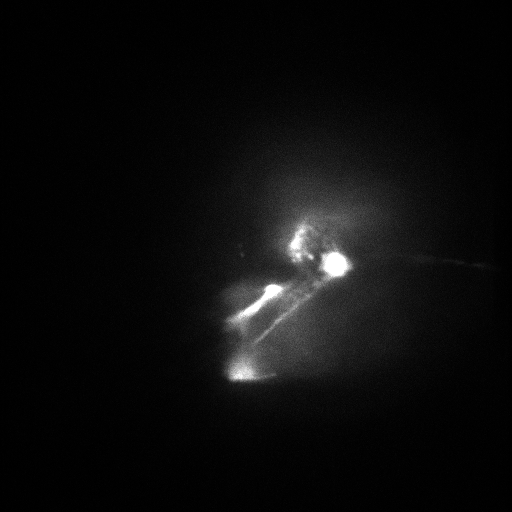

Supplement: Supplementary file 13 — Source data Fig. 4 [file 44318_2024_118_MOESM13_ESM.zip › Figure4/Figure 4B Micr. image/20210908 Phlh-17-mCherry; osm-3-g444e-gfp100x_5/Pos0/img_000000000_Confocal-561_023.tif]

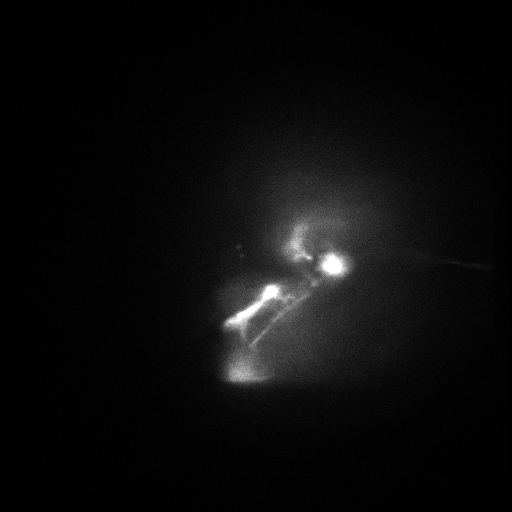

Supplement: Supplementary file 13 — Source data Fig. 4 [file 44318_2024_118_MOESM13_ESM.zip › Figure4/Figure 4B Micr. image/20210908 Phlh-17-mCherry; osm-3-g444e-gfp100x_5/Pos0/img_000000000_Confocal-561_024.tif]

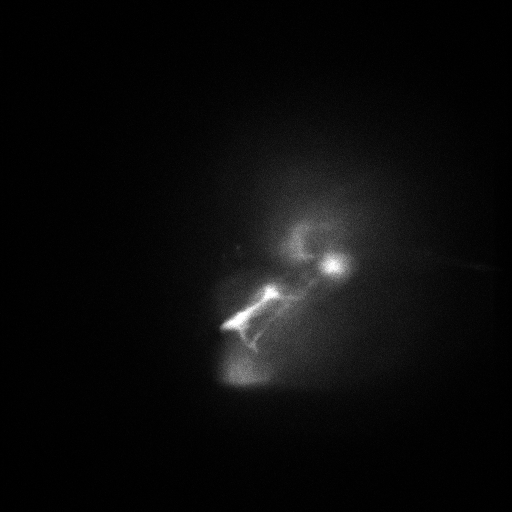

Supplement: Supplementary file 13 — Source data Fig. 4 [file 44318_2024_118_MOESM13_ESM.zip › Figure4/Figure 4B Micr. image/20210908 Phlh-17-mCherry; osm-3-g444e-gfp100x_5/Pos0/img_000000000_Confocal-561_025.tif]

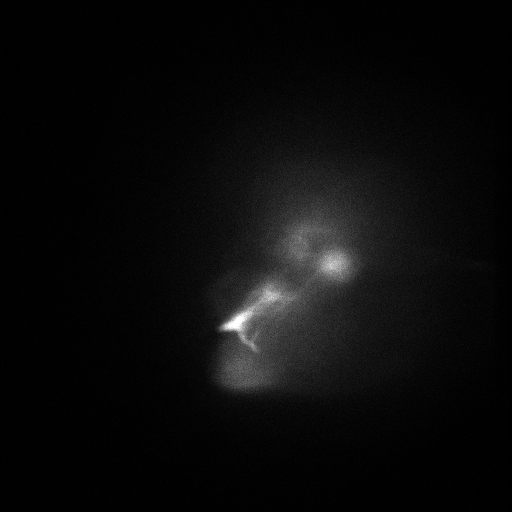

Supplement: Supplementary file 13 — Source data Fig. 4 [file 44318_2024_118_MOESM13_ESM.zip › Figure4/Figure 4B Micr. image/20210908 Phlh-17-mCherry; osm-3-g444e-gfp100x_5/Pos0/img_000000000_Confocal-561_026.tif]

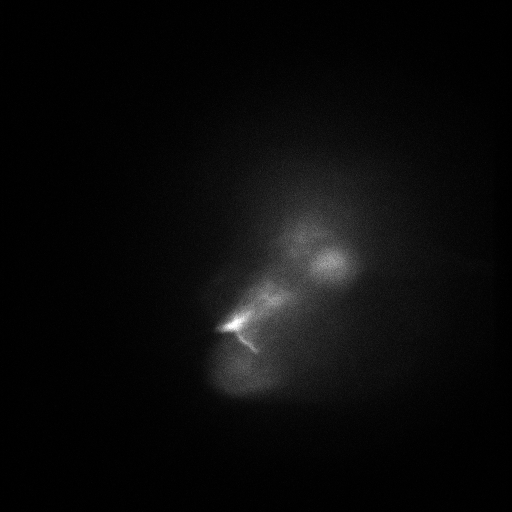

Supplement: Supplementary file 13 — Source data Fig. 4 [file 44318_2024_118_MOESM13_ESM.zip › Figure4/Figure 4B Micr. image/20210908 Phlh-17-mCherry; osm-3-g444e-gfp100x_5/Pos0/img_000000000_Confocal-561_027.tif]

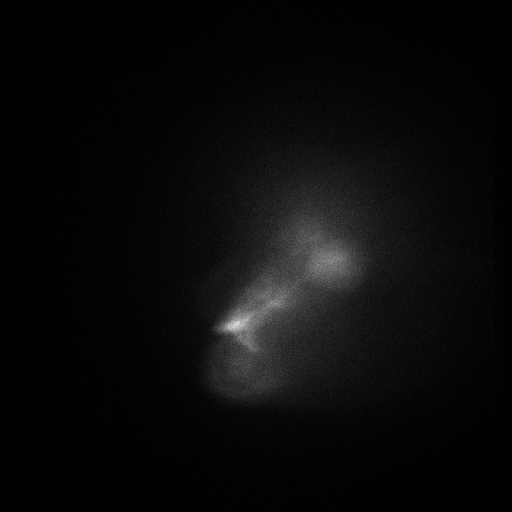

Supplement: Supplementary file 13 — Source data Fig. 4 [file 44318_2024_118_MOESM13_ESM.zip › Figure4/Figure 4B Micr. image/20210908 Phlh-17-mCherry; osm-3-g444e-gfp100x_5/Pos0/img_000000000_Confocal-561_028.tif]

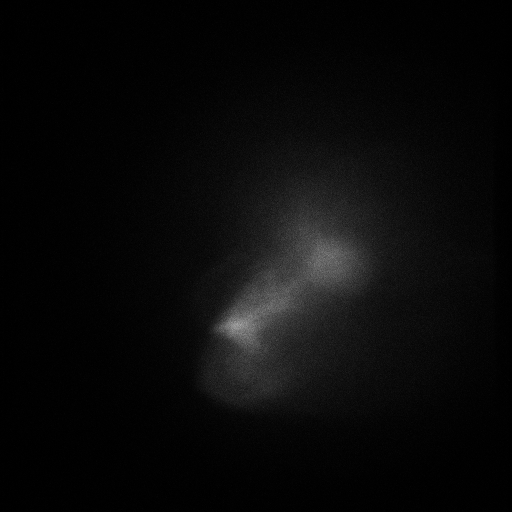

Supplement: Supplementary file 13 — Source data Fig. 4 [file 44318_2024_118_MOESM13_ESM.zip › Figure4/Figure 4B Micr. image/20210908 Phlh-17-mCherry; osm-3-g444e-gfp100x_5/Pos0/img_000000000_Confocal-561_029.tif]

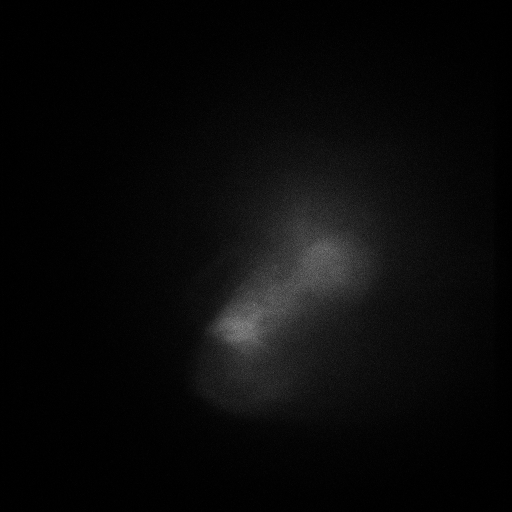

Supplement: Supplementary file 13 — Source data Fig. 4 [file 44318_2024_118_MOESM13_ESM.zip › Figure4/Figure 4B Micr. image/20210908 Phlh-17-mCherry; osm-3-g444e-gfp100x_5/Pos0/img_000000000_Confocal-561_030.tif]

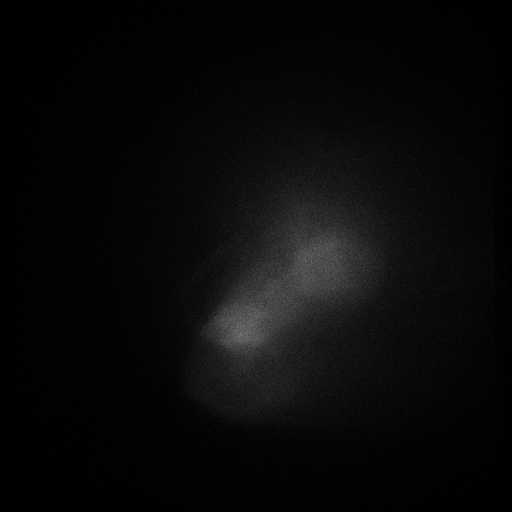

Supplement: Supplementary file 13 — Source data Fig. 4 [file 44318_2024_118_MOESM13_ESM.zip › Figure4/Figure 4B Micr. image/20210908 Phlh-17-mCherry; osm-3-g444e-gfp100x_5/Pos0/img_000000000_Confocal-561_031.tif]

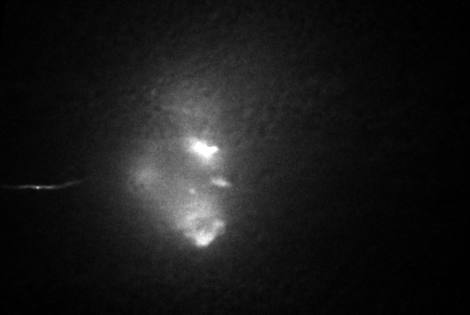

Supplement: Supplementary file 13 — Source data Fig. 4 [file 44318_2024_118_MOESM13_ESM.zip › Figure4/Figure 4B Micr. image/20210908 Phlh-17-mCherry; osm-3-g444e-gfp100x_5-2.tif]

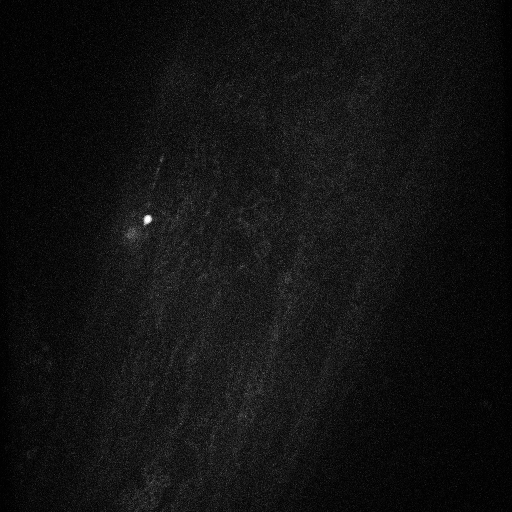

Supplement: Supplementary file 13 — Source data Fig. 4 [file 44318_2024_118_MOESM13_ESM.zip › Figure4/Figure 4C Micr. image/20210510 osm-3 G444E-gfp/Pos0/img_000000000_Confocal-488-Acq_000.tif]

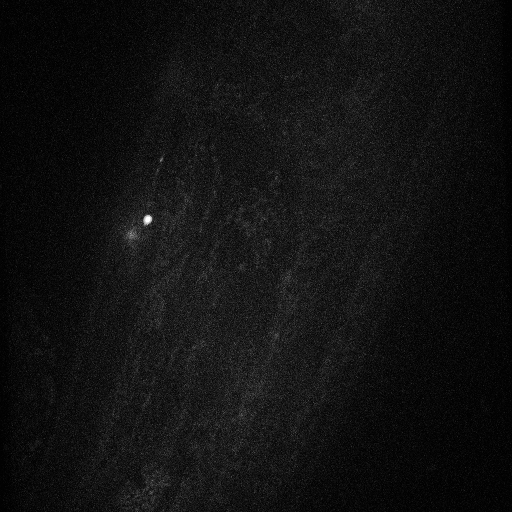

Supplement: Supplementary file 13 — Source data Fig. 4 [file 44318_2024_118_MOESM13_ESM.zip › Figure4/Figure 4C Micr. image/20210510 osm-3 G444E-gfp/Pos0/img_000000000_Confocal-488-Acq_001.tif]

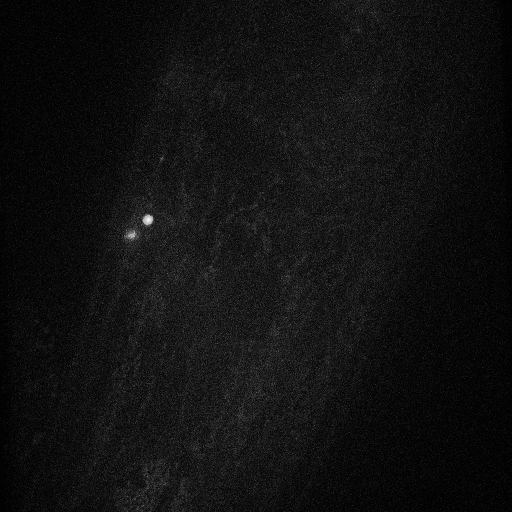

Supplement: Supplementary file 13 — Source data Fig. 4 [file 44318_2024_118_MOESM13_ESM.zip › Figure4/Figure 4C Micr. image/20210510 osm-3 G444E-gfp/Pos0/img_000000000_Confocal-488-Acq_002.tif]

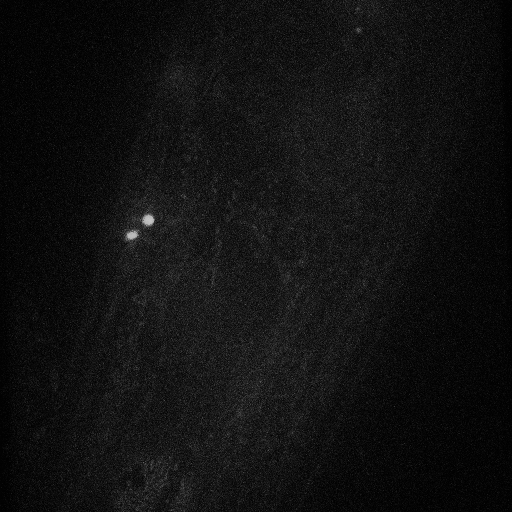

Supplement: Supplementary file 13 — Source data Fig. 4 [file 44318_2024_118_MOESM13_ESM.zip › Figure4/Figure 4C Micr. image/20210510 osm-3 G444E-gfp/Pos0/img_000000000_Confocal-488-Acq_003.tif]

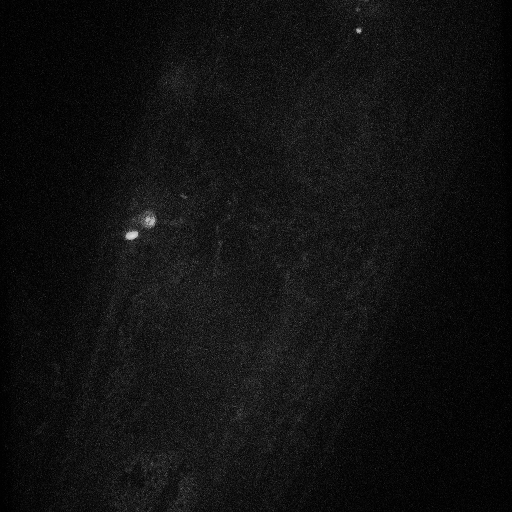

Supplement: Supplementary file 13 — Source data Fig. 4 [file 44318_2024_118_MOESM13_ESM.zip › Figure4/Figure 4C Micr. image/20210510 osm-3 G444E-gfp/Pos0/img_000000000_Confocal-488-Acq_004.tif]

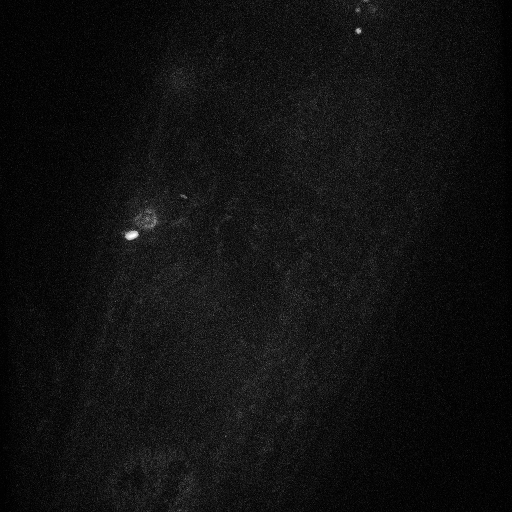

Supplement: Supplementary file 13 — Source data Fig. 4 [file 44318_2024_118_MOESM13_ESM.zip › Figure4/Figure 4C Micr. image/20210510 osm-3 G444E-gfp/Pos0/img_000000000_Confocal-488-Acq_005.tif]

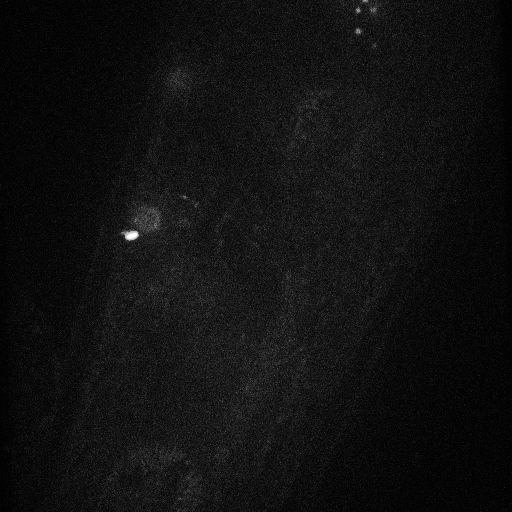

Supplement: Supplementary file 13 — Source data Fig. 4 [file 44318_2024_118_MOESM13_ESM.zip › Figure4/Figure 4C Micr. image/20210510 osm-3 G444E-gfp/Pos0/img_000000000_Confocal-488-Acq_006.tif]

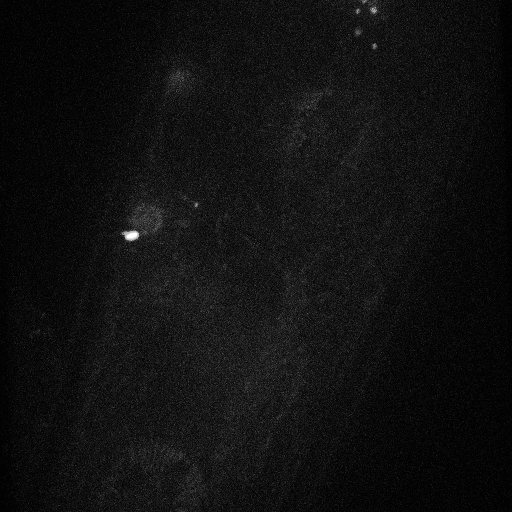

Supplement: Supplementary file 13 — Source data Fig. 4 [file 44318_2024_118_MOESM13_ESM.zip › Figure4/Figure 4C Micr. image/20210510 osm-3 G444E-gfp/Pos0/img_000000000_Confocal-488-Acq_007.tif]

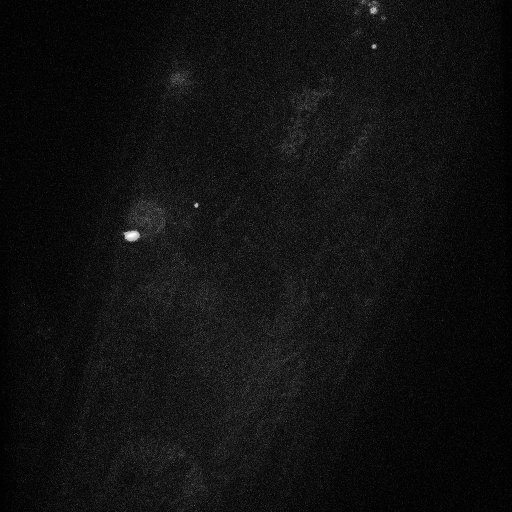

Supplement: Supplementary file 13 — Source data Fig. 4 [file 44318_2024_118_MOESM13_ESM.zip › Figure4/Figure 4C Micr. image/20210510 osm-3 G444E-gfp/Pos0/img_000000000_Confocal-488-Acq_008.tif]

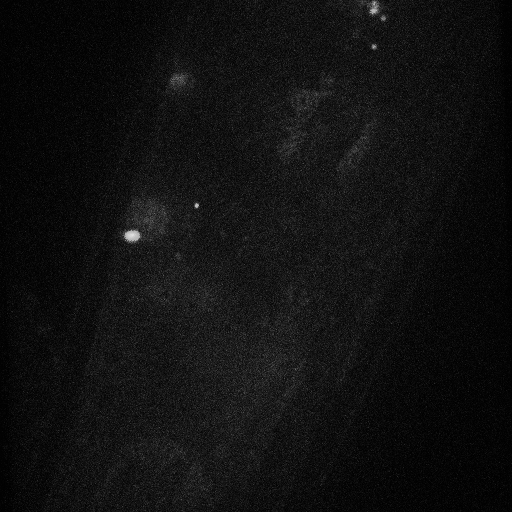

Supplement: Supplementary file 13 — Source data Fig. 4 [file 44318_2024_118_MOESM13_ESM.zip › Figure4/Figure 4C Micr. image/20210510 osm-3 G444E-gfp/Pos0/img_000000000_Confocal-488-Acq_009.tif]

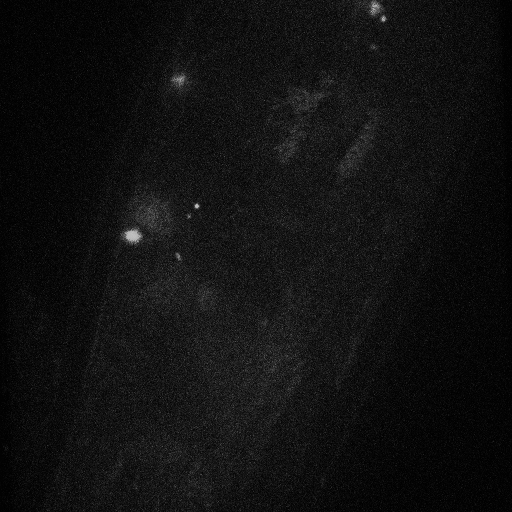

Supplement: Supplementary file 13 — Source data Fig. 4 [file 44318_2024_118_MOESM13_ESM.zip › Figure4/Figure 4C Micr. image/20210510 osm-3 G444E-gfp/Pos0/img_000000000_Confocal-488-Acq_010.tif]

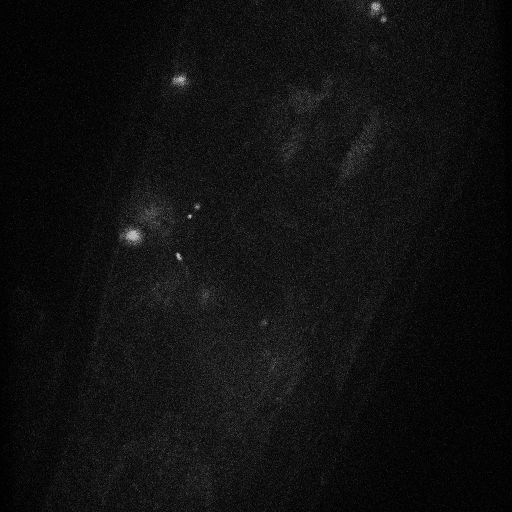

Supplement: Supplementary file 13 — Source data Fig. 4 [file 44318_2024_118_MOESM13_ESM.zip › Figure4/Figure 4C Micr. image/20210510 osm-3 G444E-gfp/Pos0/img_000000000_Confocal-488-Acq_011.tif]

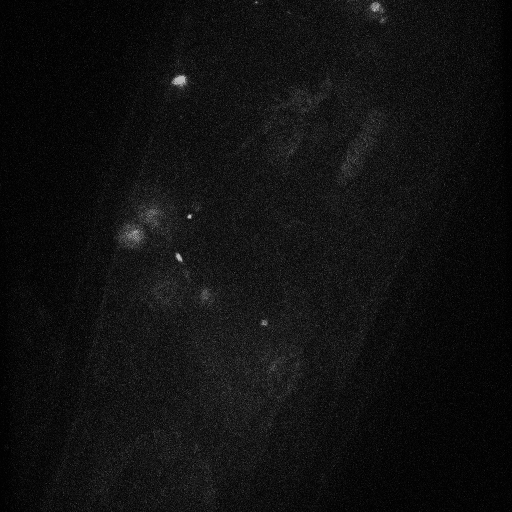

Supplement: Supplementary file 13 — Source data Fig. 4 [file 44318_2024_118_MOESM13_ESM.zip › Figure4/Figure 4C Micr. image/20210510 osm-3 G444E-gfp/Pos0/img_000000000_Confocal-488-Acq_012.tif]

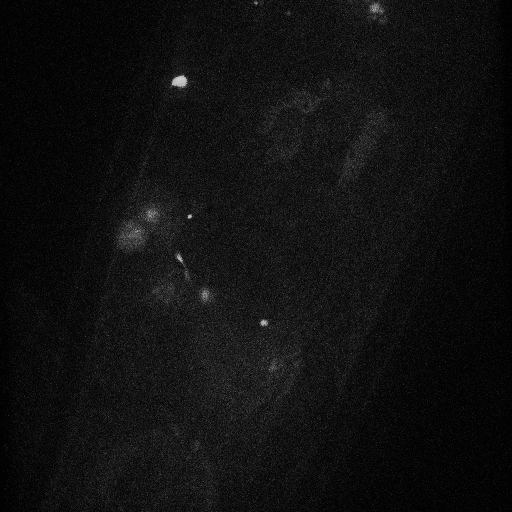

Supplement: Supplementary file 13 — Source data Fig. 4 [file 44318_2024_118_MOESM13_ESM.zip › Figure4/Figure 4C Micr. image/20210510 osm-3 G444E-gfp/Pos0/img_000000000_Confocal-488-Acq_013.tif]

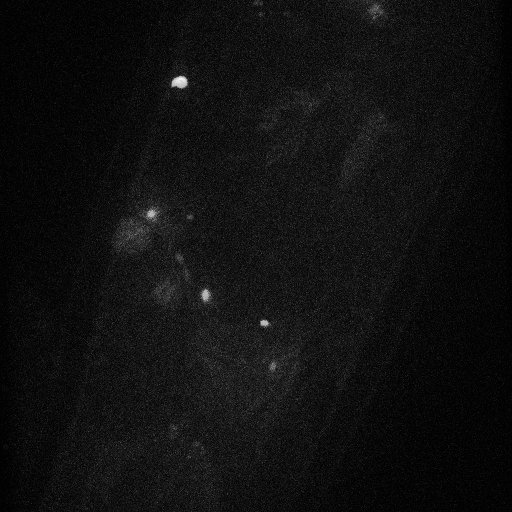

Supplement: Supplementary file 13 — Source data Fig. 4 [file 44318_2024_118_MOESM13_ESM.zip › Figure4/Figure 4C Micr. image/20210510 osm-3 G444E-gfp/Pos0/img_000000000_Confocal-488-Acq_014.tif]

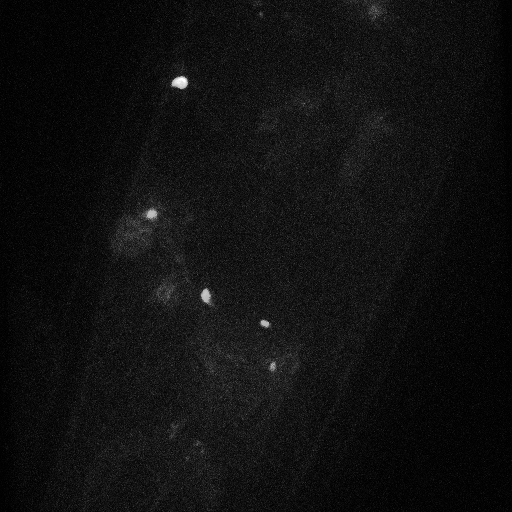

Supplement: Supplementary file 13 — Source data Fig. 4 [file 44318_2024_118_MOESM13_ESM.zip › Figure4/Figure 4C Micr. image/20210510 osm-3 G444E-gfp/Pos0/img_000000000_Confocal-488-Acq_015.tif]

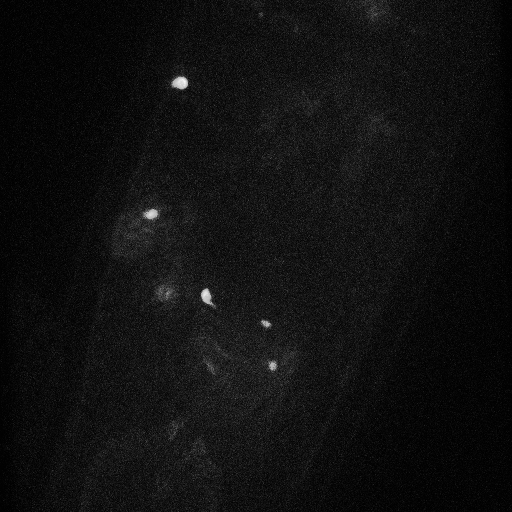

Supplement: Supplementary file 13 — Source data Fig. 4 [file 44318_2024_118_MOESM13_ESM.zip › Figure4/Figure 4C Micr. image/20210510 osm-3 G444E-gfp/Pos0/img_000000000_Confocal-488-Acq_016.tif]

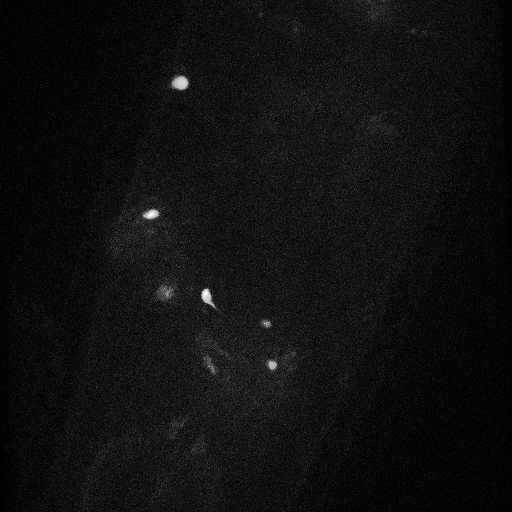

Supplement: Supplementary file 13 — Source data Fig. 4 [file 44318_2024_118_MOESM13_ESM.zip › Figure4/Figure 4C Micr. image/20210510 osm-3 G444E-gfp/Pos0/img_000000000_Confocal-488-Acq_017.tif]

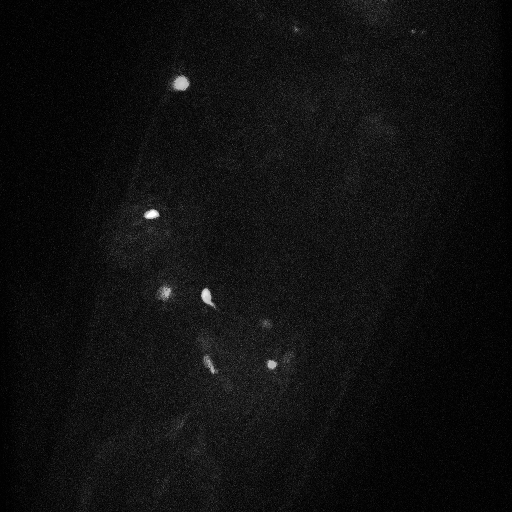

Supplement: Supplementary file 13 — Source data Fig. 4 [file 44318_2024_118_MOESM13_ESM.zip › Figure4/Figure 4C Micr. image/20210510 osm-3 G444E-gfp/Pos0/img_000000000_Confocal-488-Acq_018.tif]

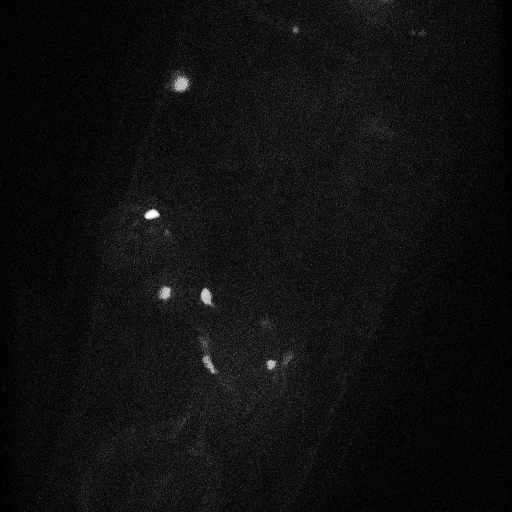

Supplement: Supplementary file 13 — Source data Fig. 4 [file 44318_2024_118_MOESM13_ESM.zip › Figure4/Figure 4C Micr. image/20210510 osm-3 G444E-gfp/Pos0/img_000000000_Confocal-488-Acq_019.tif]

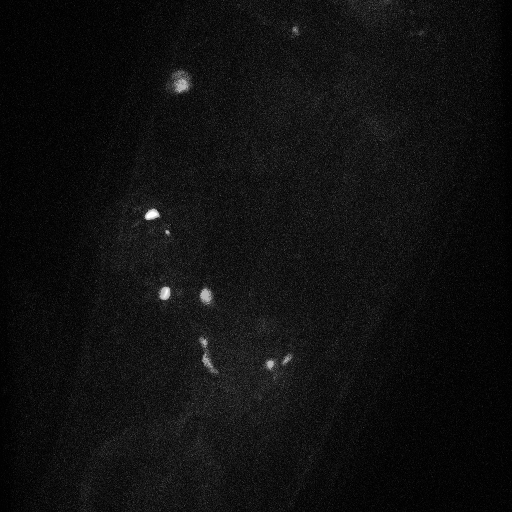

Supplement: Supplementary file 13 — Source data Fig. 4 [file 44318_2024_118_MOESM13_ESM.zip › Figure4/Figure 4C Micr. image/20210510 osm-3 G444E-gfp/Pos0/img_000000000_Confocal-488-Acq_020.tif]

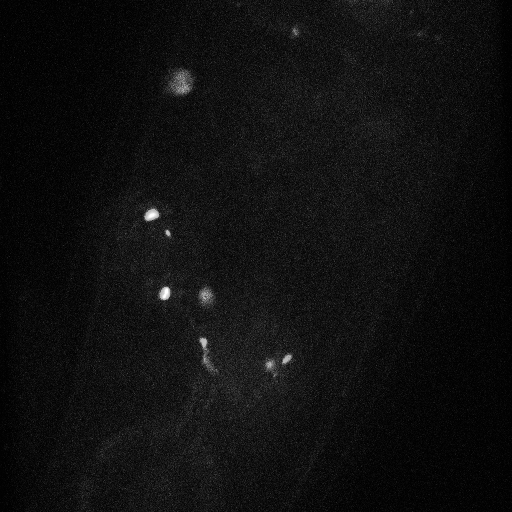

Supplement: Supplementary file 13 — Source data Fig. 4 [file 44318_2024_118_MOESM13_ESM.zip › Figure4/Figure 4C Micr. image/20210510 osm-3 G444E-gfp/Pos0/img_000000000_Confocal-488-Acq_021.tif]

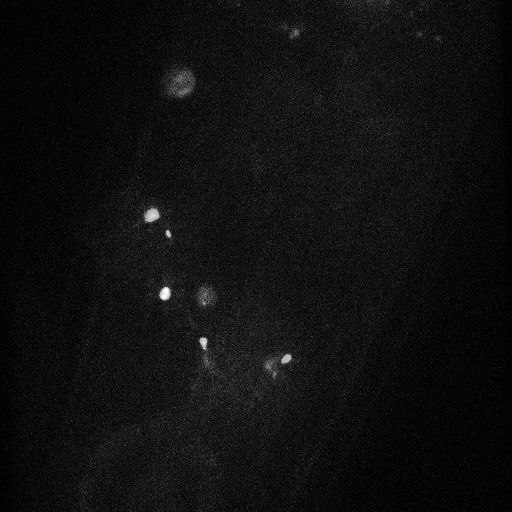

Supplement: Supplementary file 13 — Source data Fig. 4 [file 44318_2024_118_MOESM13_ESM.zip › Figure4/Figure 4C Micr. image/20210510 osm-3 G444E-gfp/Pos0/img_000000000_Confocal-488-Acq_022.tif]

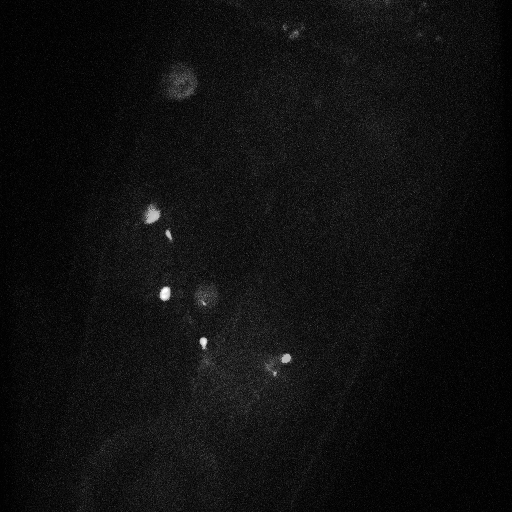

Supplement: Supplementary file 13 — Source data Fig. 4 [file 44318_2024_118_MOESM13_ESM.zip › Figure4/Figure 4C Micr. image/20210510 osm-3 G444E-gfp/Pos0/img_000000000_Confocal-488-Acq_023.tif]

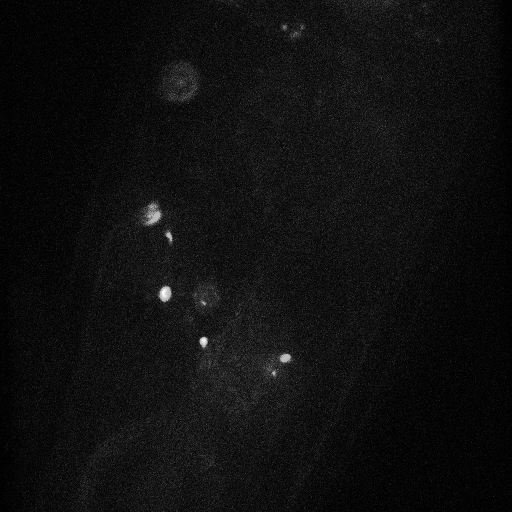

Supplement: Supplementary file 13 — Source data Fig. 4 [file 44318_2024_118_MOESM13_ESM.zip › Figure4/Figure 4C Micr. image/20210510 osm-3 G444E-gfp/Pos0/img_000000000_Confocal-488-Acq_024.tif]

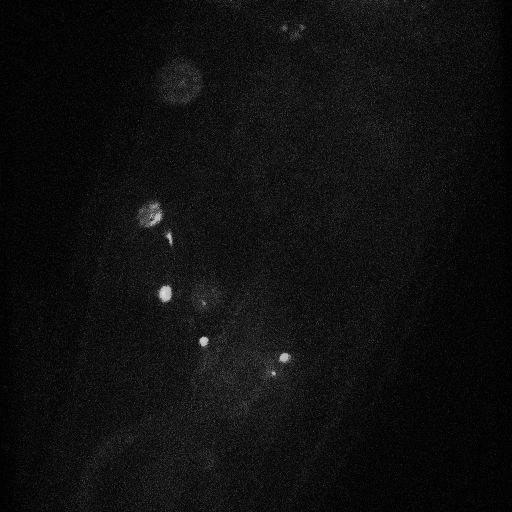

Supplement: Supplementary file 13 — Source data Fig. 4 [file 44318_2024_118_MOESM13_ESM.zip › Figure4/Figure 4C Micr. image/20210510 osm-3 G444E-gfp/Pos0/img_000000000_Confocal-488-Acq_025.tif]

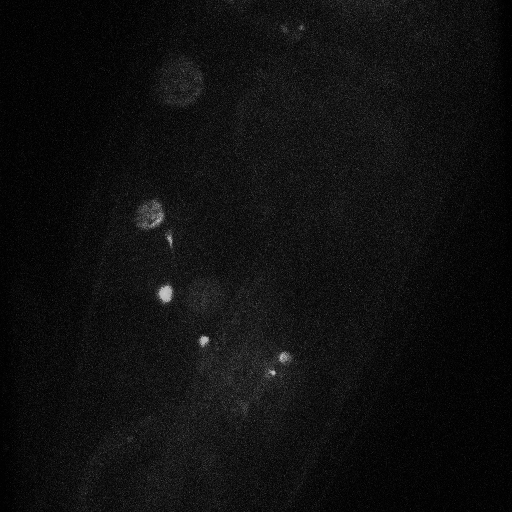

Supplement: Supplementary file 13 — Source data Fig. 4 [file 44318_2024_118_MOESM13_ESM.zip › Figure4/Figure 4C Micr. image/20210510 osm-3 G444E-gfp/Pos0/img_000000000_Confocal-488-Acq_026.tif]

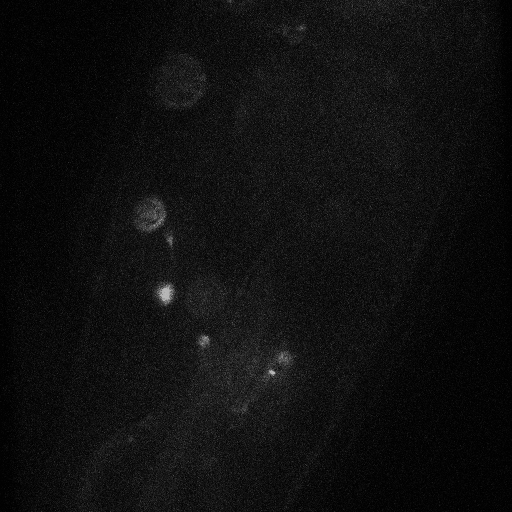

Supplement: Supplementary file 13 — Source data Fig. 4 [file 44318_2024_118_MOESM13_ESM.zip › Figure4/Figure 4C Micr. image/20210510 osm-3 G444E-gfp/Pos0/img_000000000_Confocal-488-Acq_027.tif]

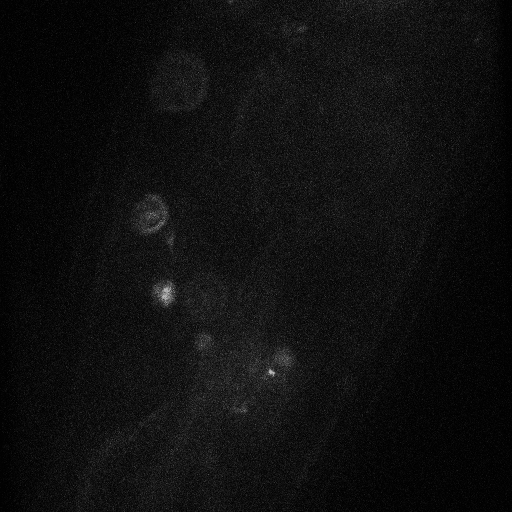

Supplement: Supplementary file 13 — Source data Fig. 4 [file 44318_2024_118_MOESM13_ESM.zip › Figure4/Figure 4C Micr. image/20210510 osm-3 G444E-gfp/Pos0/img_000000000_Confocal-488-Acq_028.tif]

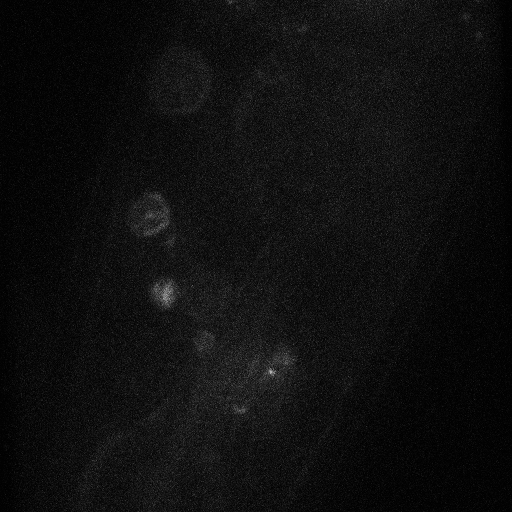

Supplement: Supplementary file 13 — Source data Fig. 4 [file 44318_2024_118_MOESM13_ESM.zip › Figure4/Figure 4C Micr. image/20210510 osm-3 G444E-gfp/Pos0/img_000000000_Confocal-488-Acq_029.tif]

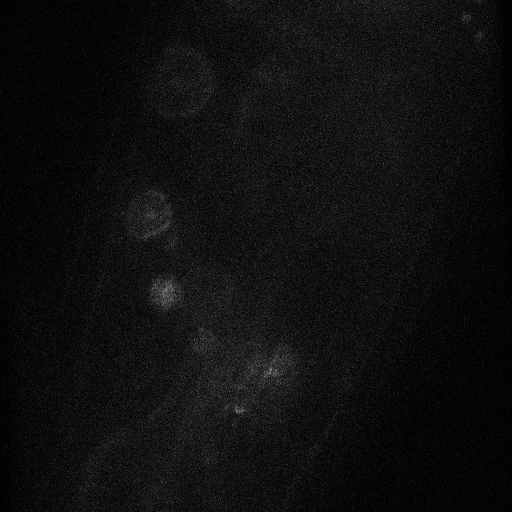

Supplement: Supplementary file 13 — Source data Fig. 4 [file 44318_2024_118_MOESM13_ESM.zip › Figure4/Figure 4C Micr. image/20210510 osm-3 G444E-gfp/Pos0/img_000000000_Confocal-488-Acq_030.tif]

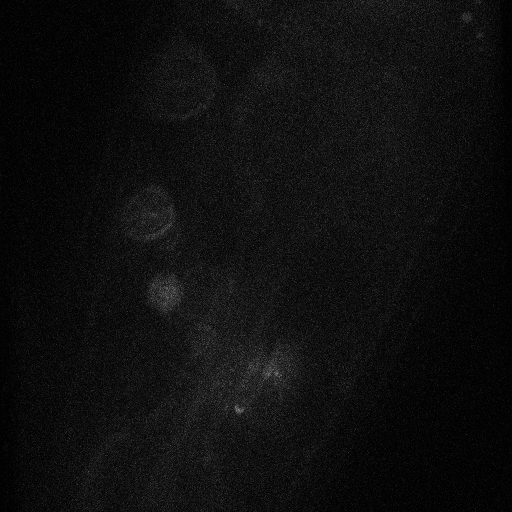

Supplement: Supplementary file 13 — Source data Fig. 4 [file 44318_2024_118_MOESM13_ESM.zip › Figure4/Figure 4C Micr. image/20210510 osm-3 G444E-gfp/Pos0/img_000000000_Confocal-488-Acq_031.tif]

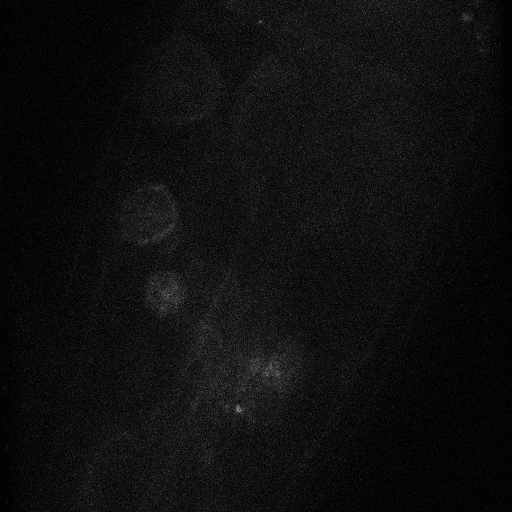

Supplement: Supplementary file 13 — Source data Fig. 4 [file 44318_2024_118_MOESM13_ESM.zip › Figure4/Figure 4C Micr. image/20210510 osm-3 G444E-gfp/Pos0/img_000000000_Confocal-488-Acq_032.tif]

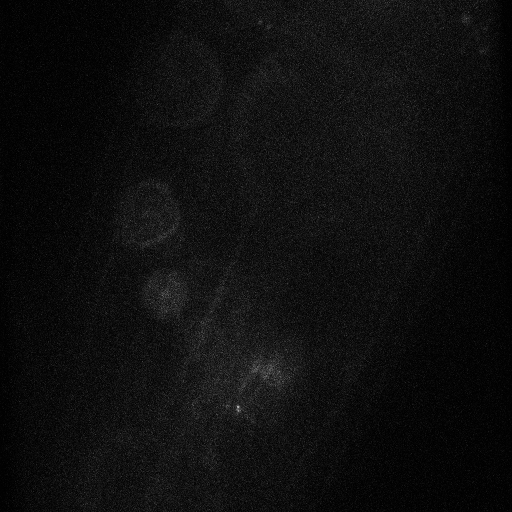

Supplement: Supplementary file 13 — Source data Fig. 4 [file 44318_2024_118_MOESM13_ESM.zip › Figure4/Figure 4C Micr. image/20210510 osm-3 G444E-gfp/Pos0/img_000000000_Confocal-488-Acq_033.tif]

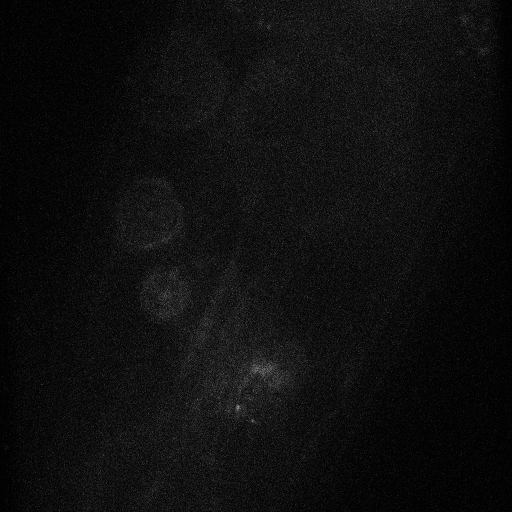

Supplement: Supplementary file 13 — Source data Fig. 4 [file 44318_2024_118_MOESM13_ESM.zip › Figure4/Figure 4C Micr. image/20210510 osm-3 G444E-gfp/Pos0/img_000000000_Confocal-488-Acq_034.tif]

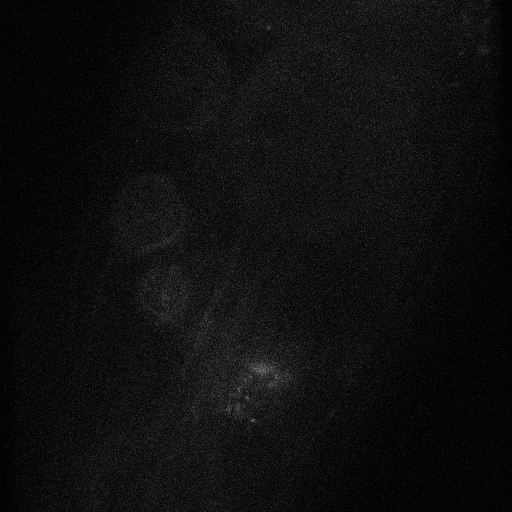

Supplement: Supplementary file 13 — Source data Fig. 4 [file 44318_2024_118_MOESM13_ESM.zip › Figure4/Figure 4C Micr. image/20210510 osm-3 G444E-gfp/Pos0/img_000000000_Confocal-488-Acq_035.tif]

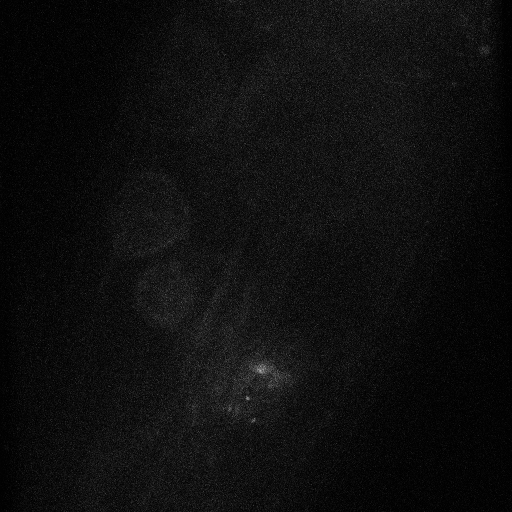

Supplement: Supplementary file 13 — Source data Fig. 4 [file 44318_2024_118_MOESM13_ESM.zip › Figure4/Figure 4C Micr. image/20210510 osm-3 G444E-gfp/Pos0/img_000000000_Confocal-488-Acq_036.tif]

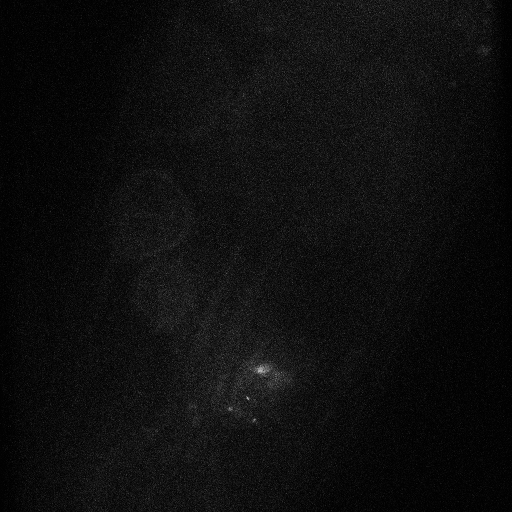

Supplement: Supplementary file 13 — Source data Fig. 4 [file 44318_2024_118_MOESM13_ESM.zip › Figure4/Figure 4C Micr. image/20210510 osm-3 G444E-gfp/Pos0/img_000000000_Confocal-488-Acq_037.tif]

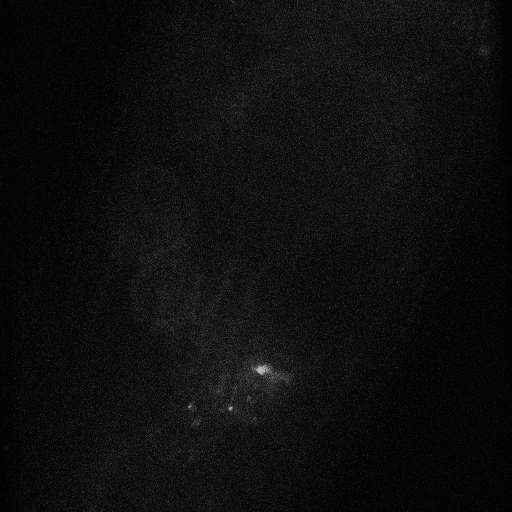

Supplement: Supplementary file 13 — Source data Fig. 4 [file 44318_2024_118_MOESM13_ESM.zip › Figure4/Figure 4C Micr. image/20210510 osm-3 G444E-gfp/Pos0/img_000000000_Confocal-488-Acq_038.tif]

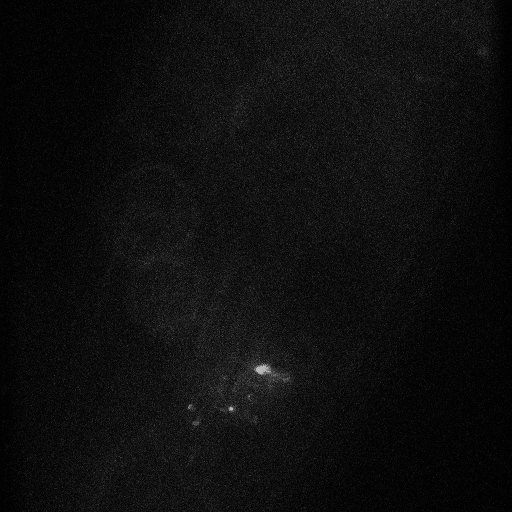

Supplement: Supplementary file 13 — Source data Fig. 4 [file 44318_2024_118_MOESM13_ESM.zip › Figure4/Figure 4C Micr. image/20210510 osm-3 G444E-gfp/Pos0/img_000000000_Confocal-488-Acq_039.tif]

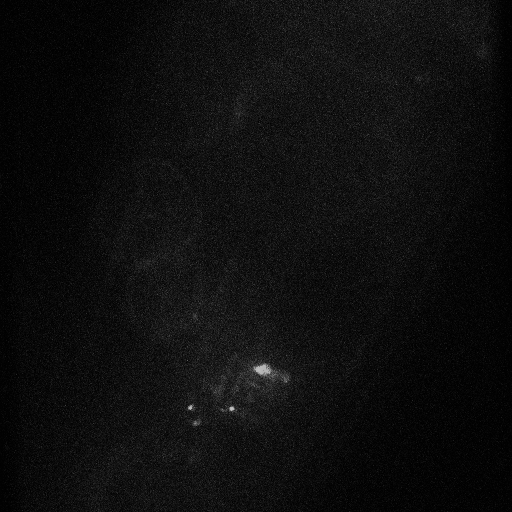

Supplement: Supplementary file 13 — Source data Fig. 4 [file 44318_2024_118_MOESM13_ESM.zip › Figure4/Figure 4C Micr. image/20210510 osm-3 G444E-gfp/Pos0/img_000000000_Confocal-488-Acq_040.tif]

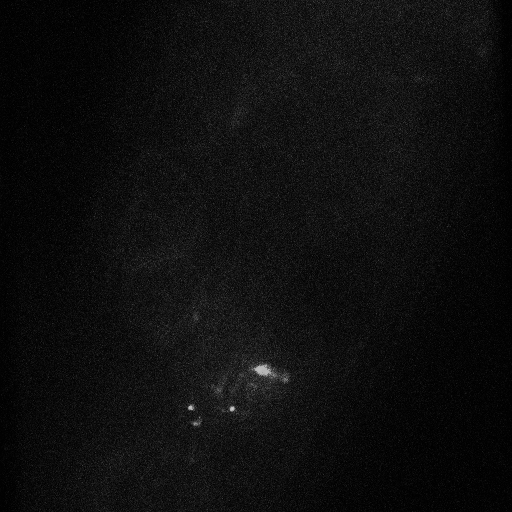

Supplement: Supplementary file 13 — Source data Fig. 4 [file 44318_2024_118_MOESM13_ESM.zip › Figure4/Figure 4C Micr. image/20210510 osm-3 G444E-gfp/Pos0/img_000000000_Confocal-488-Acq_041.tif]

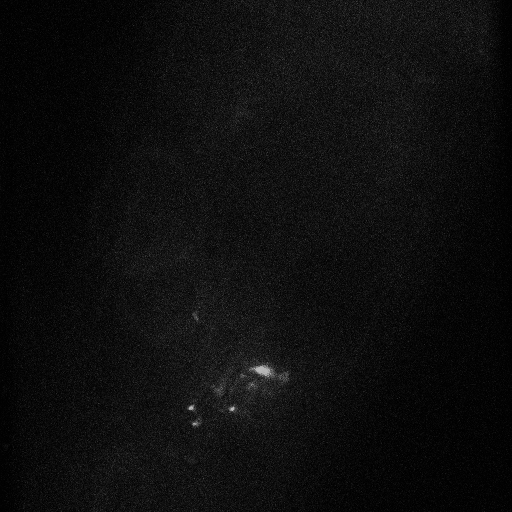

Supplement: Supplementary file 13 — Source data Fig. 4 [file 44318_2024_118_MOESM13_ESM.zip › Figure4/Figure 4C Micr. image/20210510 osm-3 G444E-gfp/Pos0/img_000000000_Confocal-488-Acq_042.tif]

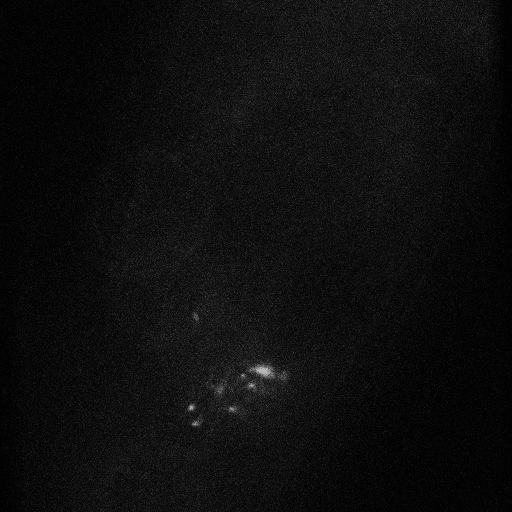

Supplement: Supplementary file 13 — Source data Fig. 4 [file 44318_2024_118_MOESM13_ESM.zip › Figure4/Figure 4C Micr. image/20210510 osm-3 G444E-gfp/Pos0/img_000000000_Confocal-488-Acq_043.tif]

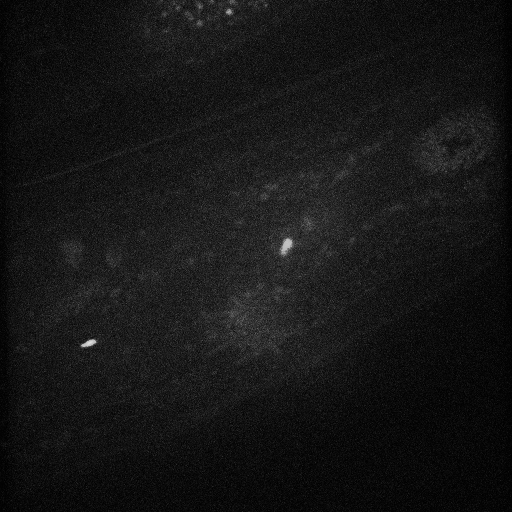

Supplement: Supplementary file 13 — Source data Fig. 4 [file 44318_2024_118_MOESM13_ESM.zip › Figure4/Figure 4C Micr. image/20220412 osm-3-G444E-gfp; ced-1_12/Pos0/img_000000000_Confocal-488-Acq_000.tif]

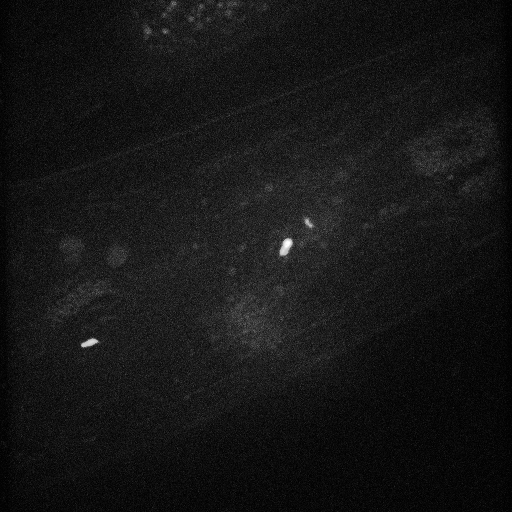

Supplement: Supplementary file 13 — Source data Fig. 4 [file 44318_2024_118_MOESM13_ESM.zip › Figure4/Figure 4C Micr. image/20220412 osm-3-G444E-gfp; ced-1_12/Pos0/img_000000000_Confocal-488-Acq_001.tif]

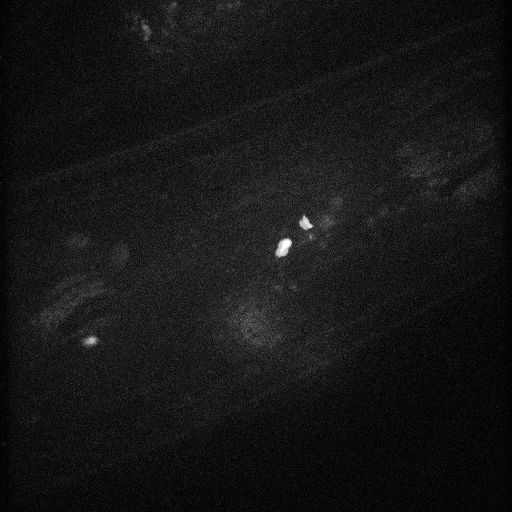

Supplement: Supplementary file 13 — Source data Fig. 4 [file 44318_2024_118_MOESM13_ESM.zip › Figure4/Figure 4C Micr. image/20220412 osm-3-G444E-gfp; ced-1_12/Pos0/img_000000000_Confocal-488-Acq_002.tif]

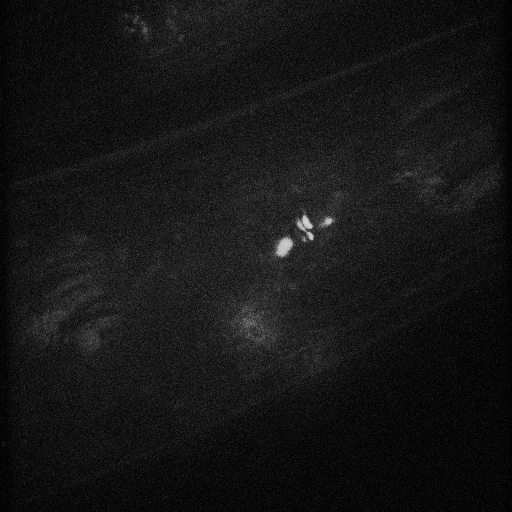

Supplement: Supplementary file 13 — Source data Fig. 4 [file 44318_2024_118_MOESM13_ESM.zip › Figure4/Figure 4C Micr. image/20220412 osm-3-G444E-gfp; ced-1_12/Pos0/img_000000000_Confocal-488-Acq_003.tif]

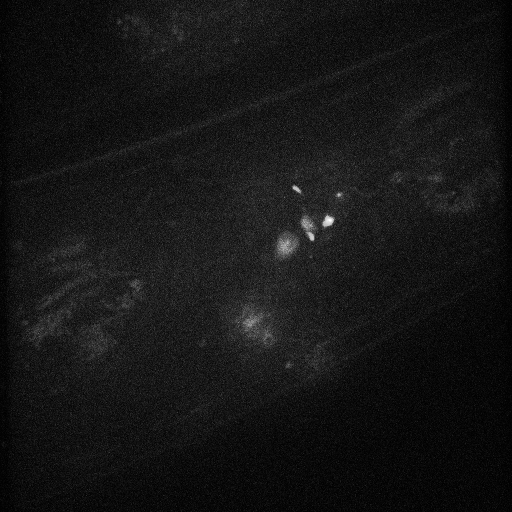

Supplement: Supplementary file 13 — Source data Fig. 4 [file 44318_2024_118_MOESM13_ESM.zip › Figure4/Figure 4C Micr. image/20220412 osm-3-G444E-gfp; ced-1_12/Pos0/img_000000000_Confocal-488-Acq_004.tif]

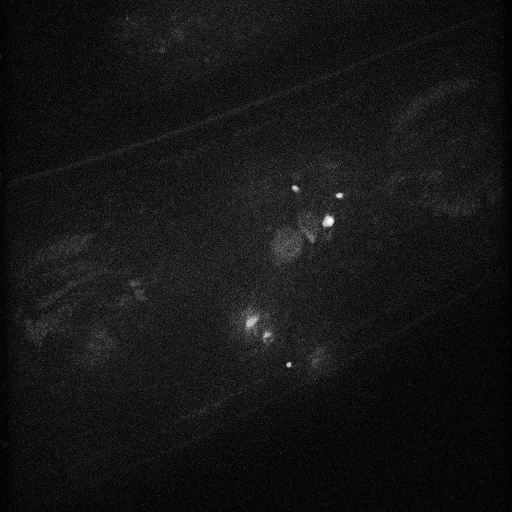

Supplement: Supplementary file 13 — Source data Fig. 4 [file 44318_2024_118_MOESM13_ESM.zip › Figure4/Figure 4C Micr. image/20220412 osm-3-G444E-gfp; ced-1_12/Pos0/img_000000000_Confocal-488-Acq_005.tif]

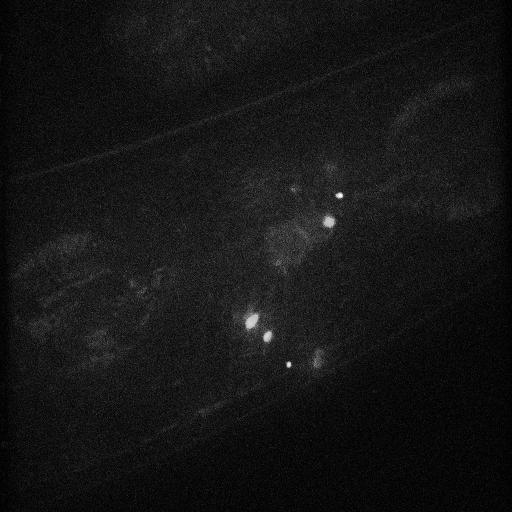

Supplement: Supplementary file 13 — Source data Fig. 4 [file 44318_2024_118_MOESM13_ESM.zip › Figure4/Figure 4C Micr. image/20220412 osm-3-G444E-gfp; ced-1_12/Pos0/img_000000000_Confocal-488-Acq_006.tif]

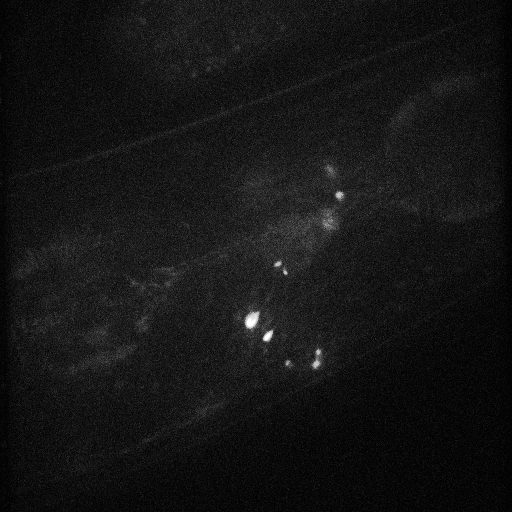

Supplement: Supplementary file 13 — Source data Fig. 4 [file 44318_2024_118_MOESM13_ESM.zip › Figure4/Figure 4C Micr. image/20220412 osm-3-G444E-gfp; ced-1_12/Pos0/img_000000000_Confocal-488-Acq_007.tif]

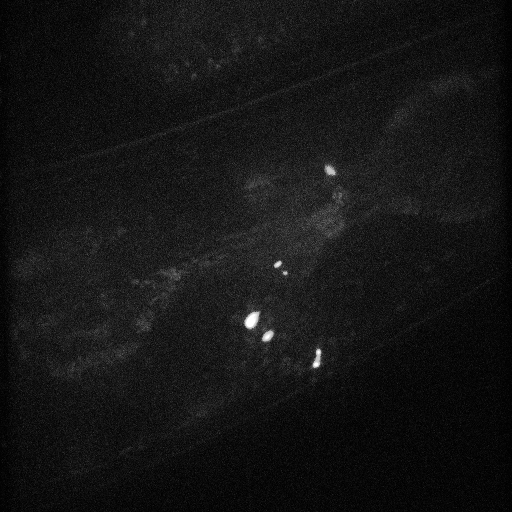

Supplement: Supplementary file 13 — Source data Fig. 4 [file 44318_2024_118_MOESM13_ESM.zip › Figure4/Figure 4C Micr. image/20220412 osm-3-G444E-gfp; ced-1_12/Pos0/img_000000000_Confocal-488-Acq_008.tif]

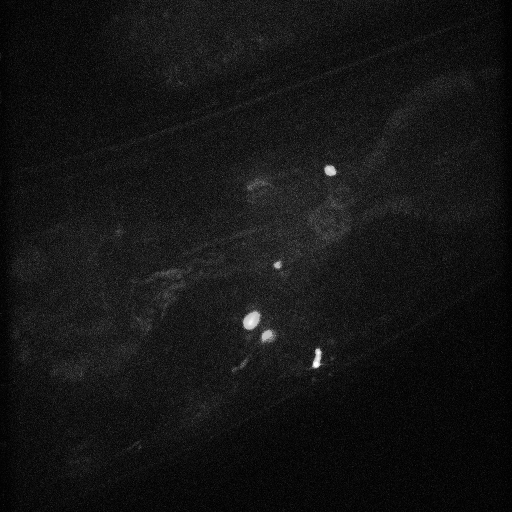

Supplement: Supplementary file 13 — Source data Fig. 4 [file 44318_2024_118_MOESM13_ESM.zip › Figure4/Figure 4C Micr. image/20220412 osm-3-G444E-gfp; ced-1_12/Pos0/img_000000000_Confocal-488-Acq_009.tif]

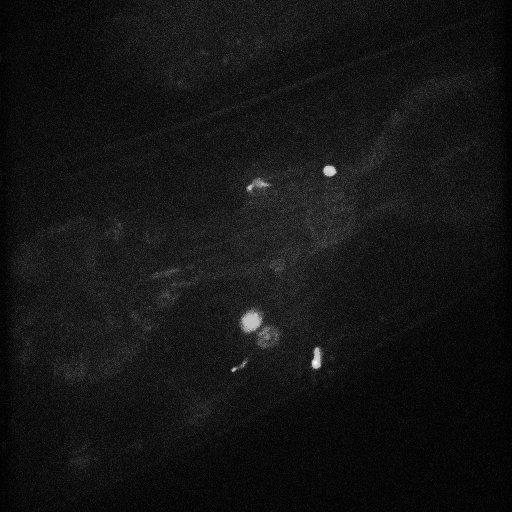

Supplement: Supplementary file 13 — Source data Fig. 4 [file 44318_2024_118_MOESM13_ESM.zip › Figure4/Figure 4C Micr. image/20220412 osm-3-G444E-gfp; ced-1_12/Pos0/img_000000000_Confocal-488-Acq_010.tif]
