# Supplementary figures and images for: Neurons dispose of hyperactive kinesin into glial cells for clearance (part 7 of 9)
Source: EMBO J. 2024 May 28;43(13):5. doi: 10.1038/s44318-024-00118-0 (PMC11217292; doi:10.1038/s44318-024-00118-0)

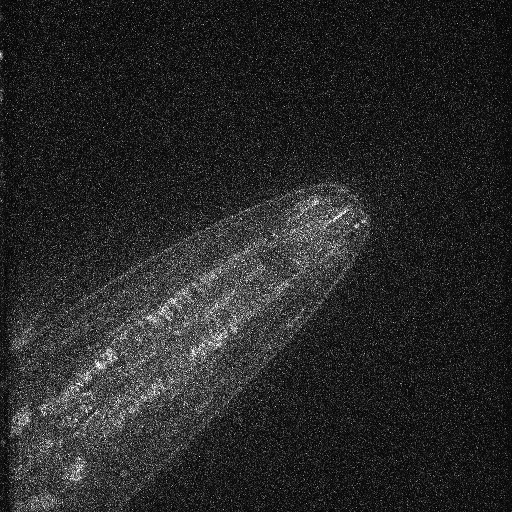

Supplement: Supplementary file 14 — Source data Fig. 5 [file 44318_2024_118_MOESM14_ESM.zip › Figure5/Figure 5C Micr. image/20211124 OSM-3-GFP KI_25/Pos0/img_000000000_Confocal-488-Acq_010.tif]

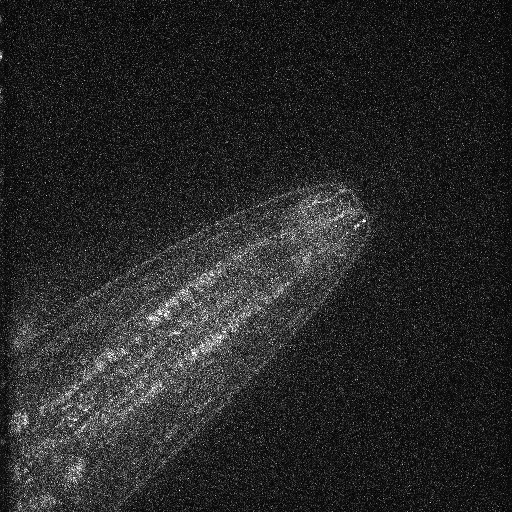

Supplement: Supplementary file 14 — Source data Fig. 5 [file 44318_2024_118_MOESM14_ESM.zip › Figure5/Figure 5C Micr. image/20211124 OSM-3-GFP KI_25/Pos0/img_000000000_Confocal-488-Acq_011.tif]

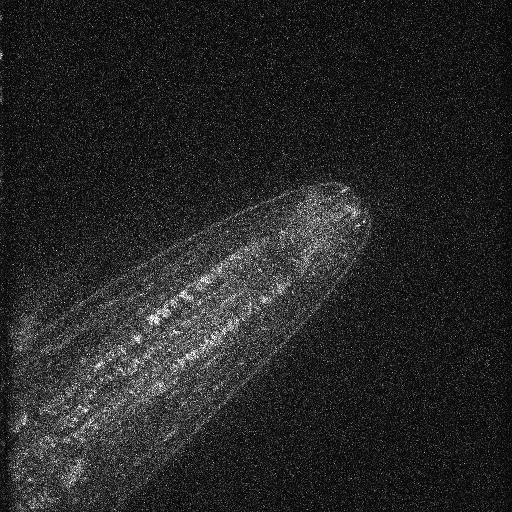

Supplement: Supplementary file 14 — Source data Fig. 5 [file 44318_2024_118_MOESM14_ESM.zip › Figure5/Figure 5C Micr. image/20211124 OSM-3-GFP KI_25/Pos0/img_000000000_Confocal-488-Acq_012.tif]

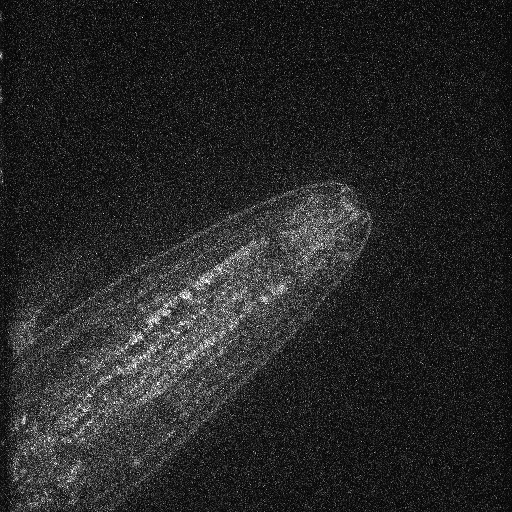

Supplement: Supplementary file 14 — Source data Fig. 5 [file 44318_2024_118_MOESM14_ESM.zip › Figure5/Figure 5C Micr. image/20211124 OSM-3-GFP KI_25/Pos0/img_000000000_Confocal-488-Acq_013.tif]

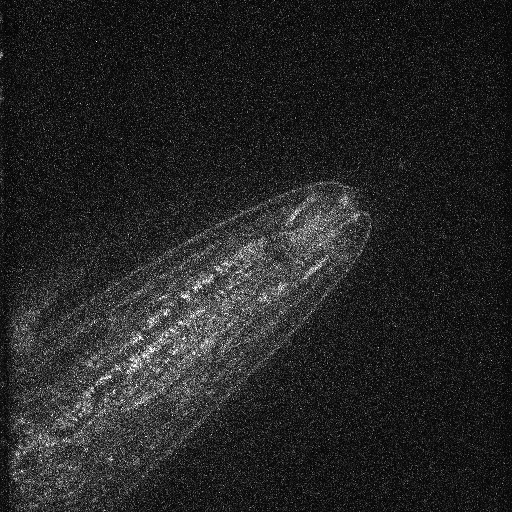

Supplement: Supplementary file 14 — Source data Fig. 5 [file 44318_2024_118_MOESM14_ESM.zip › Figure5/Figure 5C Micr. image/20211124 OSM-3-GFP KI_25/Pos0/img_000000000_Confocal-488-Acq_014.tif]

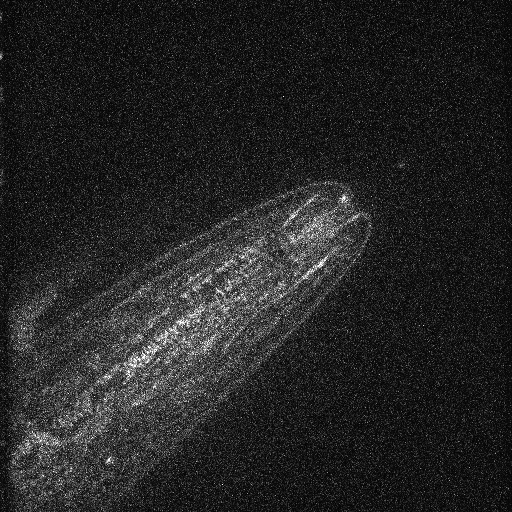

Supplement: Supplementary file 14 — Source data Fig. 5 [file 44318_2024_118_MOESM14_ESM.zip › Figure5/Figure 5C Micr. image/20211124 OSM-3-GFP KI_25/Pos0/img_000000000_Confocal-488-Acq_015.tif]

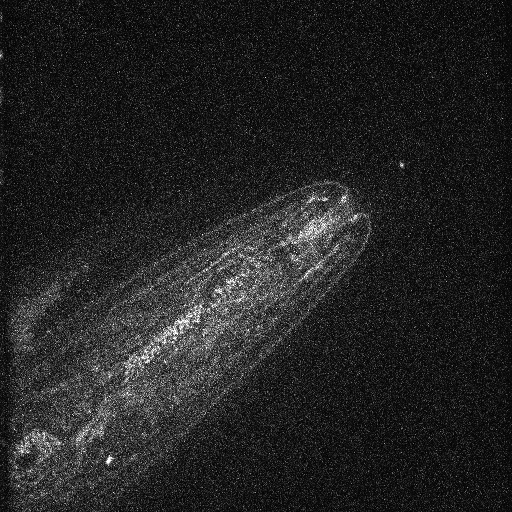

Supplement: Supplementary file 14 — Source data Fig. 5 [file 44318_2024_118_MOESM14_ESM.zip › Figure5/Figure 5C Micr. image/20211124 OSM-3-GFP KI_25/Pos0/img_000000000_Confocal-488-Acq_016.tif]

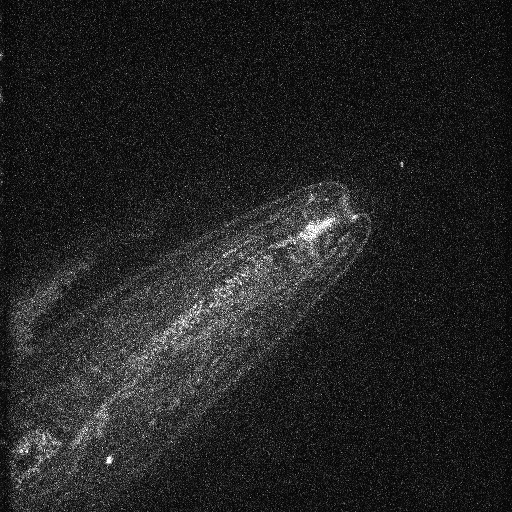

Supplement: Supplementary file 14 — Source data Fig. 5 [file 44318_2024_118_MOESM14_ESM.zip › Figure5/Figure 5C Micr. image/20211124 OSM-3-GFP KI_25/Pos0/img_000000000_Confocal-488-Acq_017.tif]

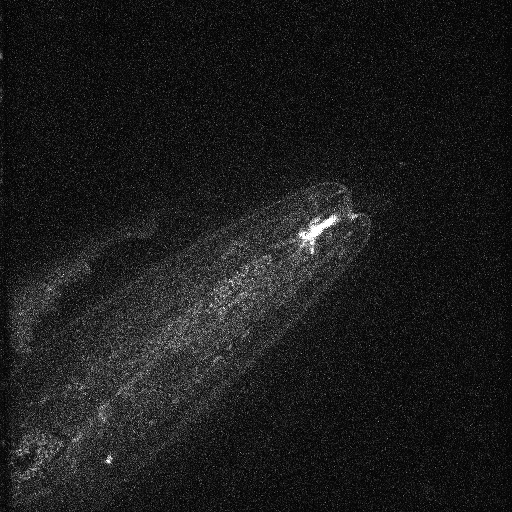

Supplement: Supplementary file 14 — Source data Fig. 5 [file 44318_2024_118_MOESM14_ESM.zip › Figure5/Figure 5C Micr. image/20211124 OSM-3-GFP KI_25/Pos0/img_000000000_Confocal-488-Acq_018.tif]

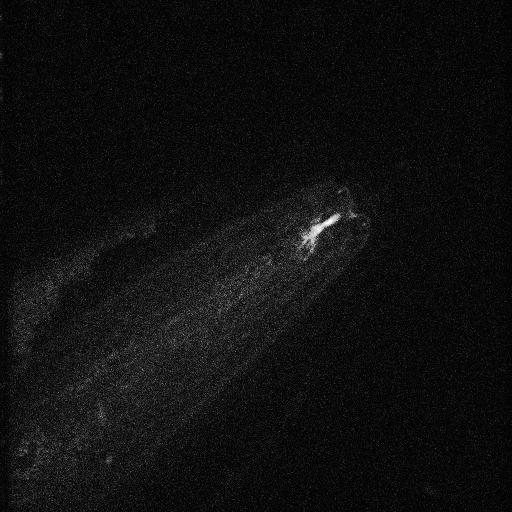

Supplement: Supplementary file 14 — Source data Fig. 5 [file 44318_2024_118_MOESM14_ESM.zip › Figure5/Figure 5C Micr. image/20211124 OSM-3-GFP KI_25/Pos0/img_000000000_Confocal-488-Acq_019.tif]

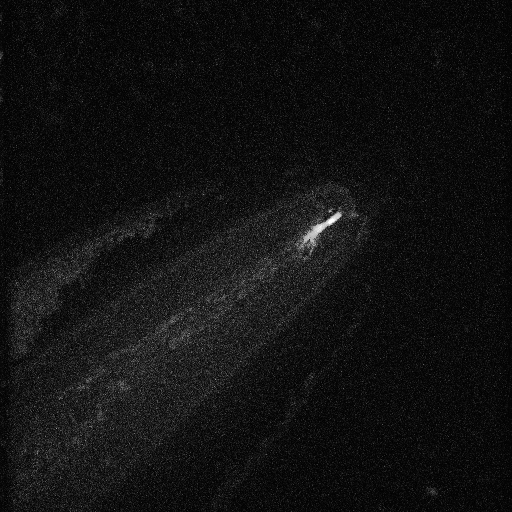

Supplement: Supplementary file 14 — Source data Fig. 5 [file 44318_2024_118_MOESM14_ESM.zip › Figure5/Figure 5C Micr. image/20211124 OSM-3-GFP KI_25/Pos0/img_000000000_Confocal-488-Acq_020.tif]

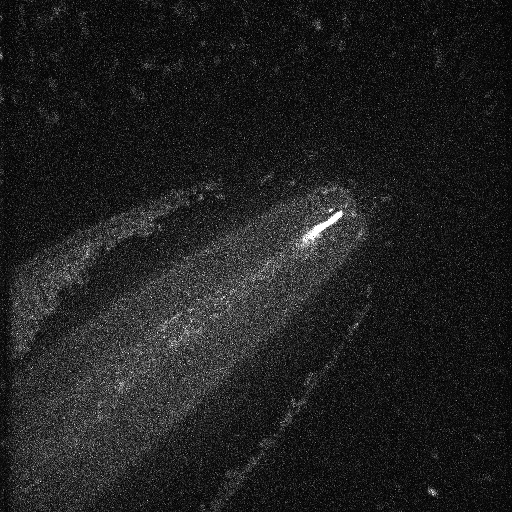

Supplement: Supplementary file 14 — Source data Fig. 5 [file 44318_2024_118_MOESM14_ESM.zip › Figure5/Figure 5C Micr. image/20211124 OSM-3-GFP KI_25/Pos0/img_000000000_Confocal-488-Acq_021.tif]

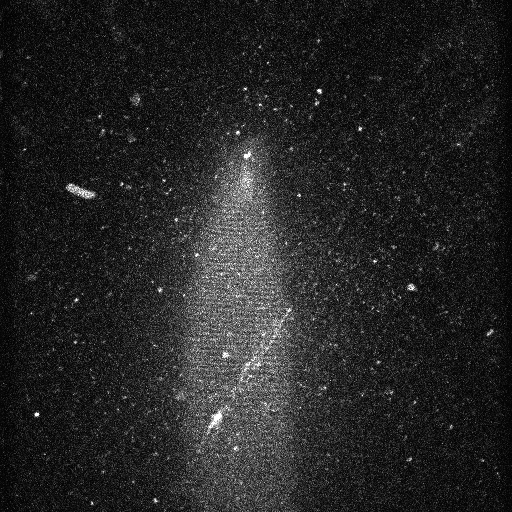

Supplement: Supplementary file 14 — Source data Fig. 5 [file 44318_2024_118_MOESM14_ESM.zip › Figure5/Figure 5C Micr. image/20211124 osm-3-R238W-gfp ki amphid/Pos0/img_000000000_Confocal-488-Acq_000.tif]

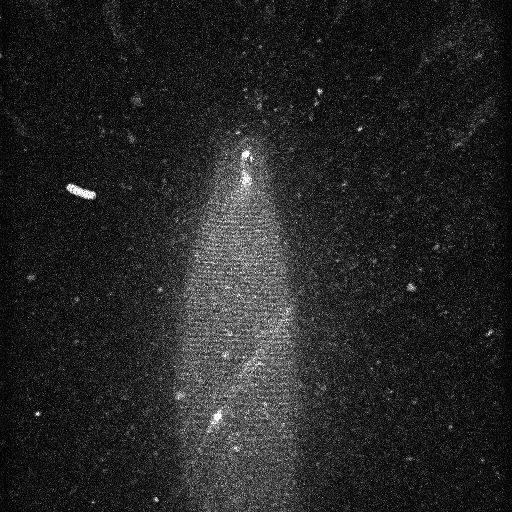

Supplement: Supplementary file 14 — Source data Fig. 5 [file 44318_2024_118_MOESM14_ESM.zip › Figure5/Figure 5C Micr. image/20211124 osm-3-R238W-gfp ki amphid/Pos0/img_000000000_Confocal-488-Acq_001.tif]

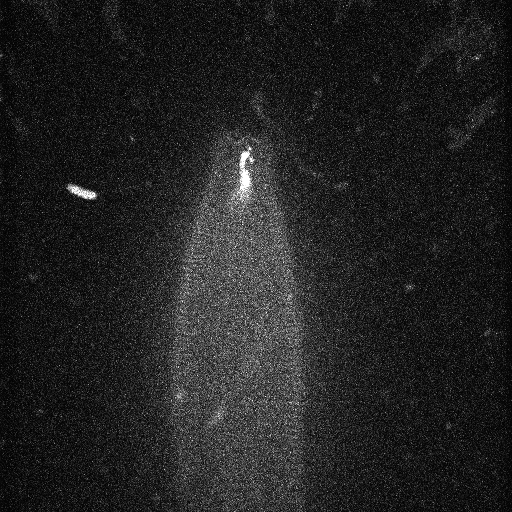

Supplement: Supplementary file 14 — Source data Fig. 5 [file 44318_2024_118_MOESM14_ESM.zip › Figure5/Figure 5C Micr. image/20211124 osm-3-R238W-gfp ki amphid/Pos0/img_000000000_Confocal-488-Acq_002.tif]

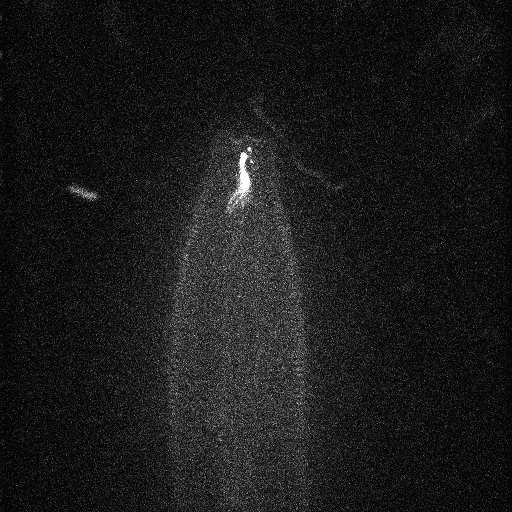

Supplement: Supplementary file 14 — Source data Fig. 5 [file 44318_2024_118_MOESM14_ESM.zip › Figure5/Figure 5C Micr. image/20211124 osm-3-R238W-gfp ki amphid/Pos0/img_000000000_Confocal-488-Acq_003.tif]

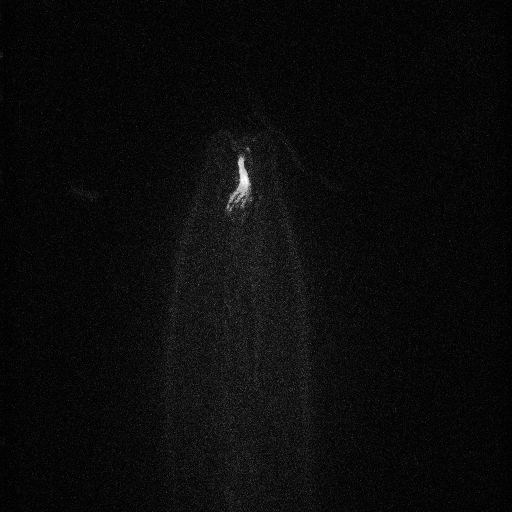

Supplement: Supplementary file 14 — Source data Fig. 5 [file 44318_2024_118_MOESM14_ESM.zip › Figure5/Figure 5C Micr. image/20211124 osm-3-R238W-gfp ki amphid/Pos0/img_000000000_Confocal-488-Acq_004.tif]

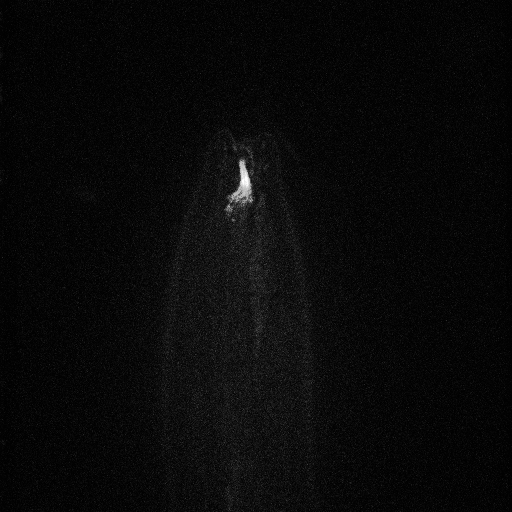

Supplement: Supplementary file 14 — Source data Fig. 5 [file 44318_2024_118_MOESM14_ESM.zip › Figure5/Figure 5C Micr. image/20211124 osm-3-R238W-gfp ki amphid/Pos0/img_000000000_Confocal-488-Acq_005.tif]

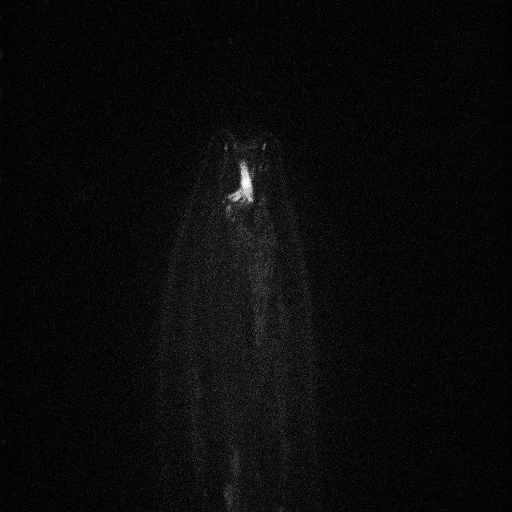

Supplement: Supplementary file 14 — Source data Fig. 5 [file 44318_2024_118_MOESM14_ESM.zip › Figure5/Figure 5C Micr. image/20211124 osm-3-R238W-gfp ki amphid/Pos0/img_000000000_Confocal-488-Acq_006.tif]

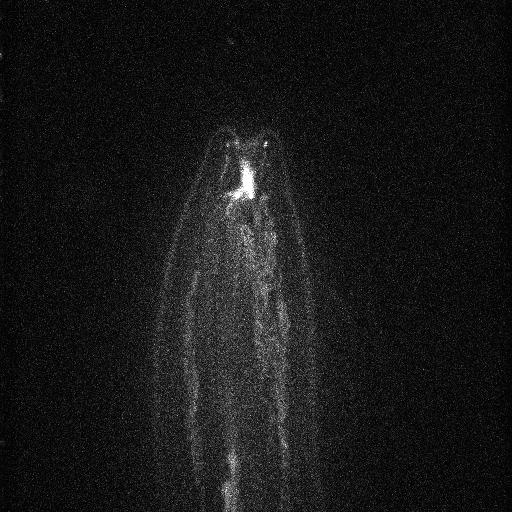

Supplement: Supplementary file 14 — Source data Fig. 5 [file 44318_2024_118_MOESM14_ESM.zip › Figure5/Figure 5C Micr. image/20211124 osm-3-R238W-gfp ki amphid/Pos0/img_000000000_Confocal-488-Acq_007.tif]

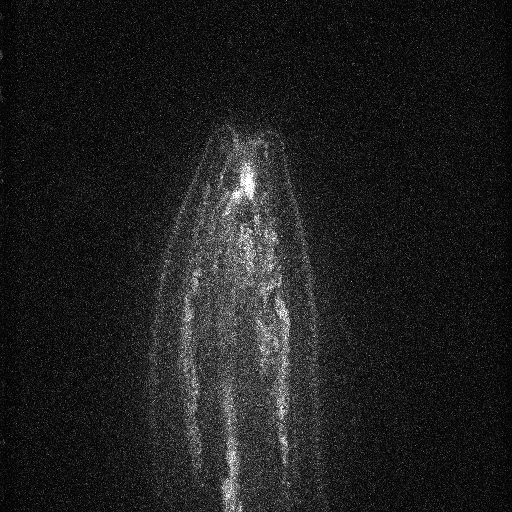

Supplement: Supplementary file 14 — Source data Fig. 5 [file 44318_2024_118_MOESM14_ESM.zip › Figure5/Figure 5C Micr. image/20211124 osm-3-R238W-gfp ki amphid/Pos0/img_000000000_Confocal-488-Acq_008.tif]

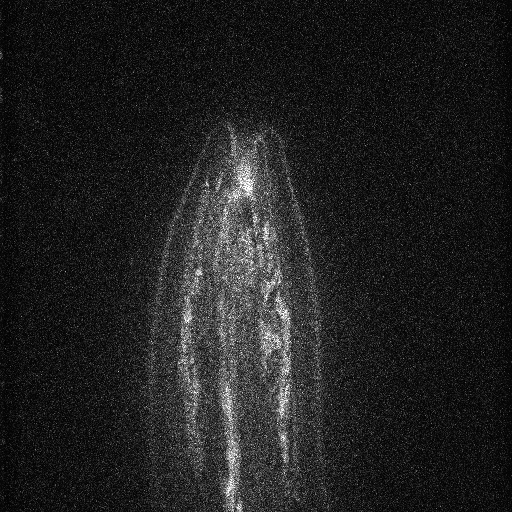

Supplement: Supplementary file 14 — Source data Fig. 5 [file 44318_2024_118_MOESM14_ESM.zip › Figure5/Figure 5C Micr. image/20211124 osm-3-R238W-gfp ki amphid/Pos0/img_000000000_Confocal-488-Acq_009.tif]

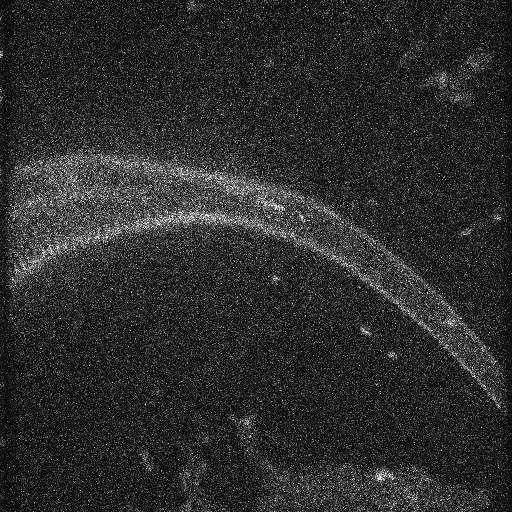

Supplement: Supplementary file 14 — Source data Fig. 5 [file 44318_2024_118_MOESM14_ESM.zip › Figure5/Figure 5C Micr. image/20211124 osm-3-R238W-gfp ki phasmid/Pos0/img_000000000_Confocal-488-Acq_000.tif]

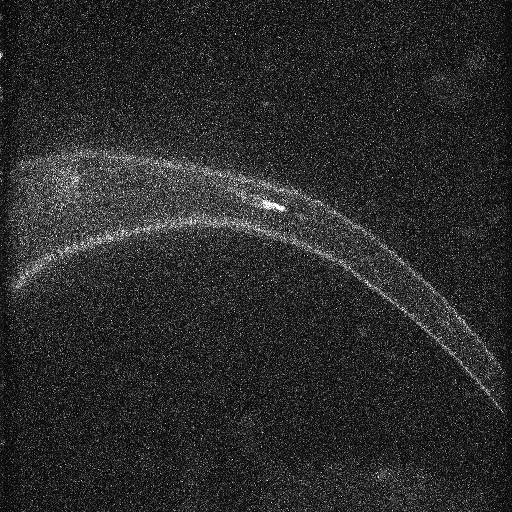

Supplement: Supplementary file 14 — Source data Fig. 5 [file 44318_2024_118_MOESM14_ESM.zip › Figure5/Figure 5C Micr. image/20211124 osm-3-R238W-gfp ki phasmid/Pos0/img_000000000_Confocal-488-Acq_001.tif]

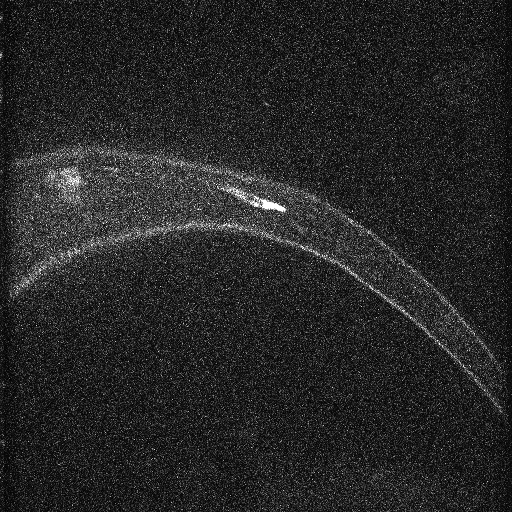

Supplement: Supplementary file 14 — Source data Fig. 5 [file 44318_2024_118_MOESM14_ESM.zip › Figure5/Figure 5C Micr. image/20211124 osm-3-R238W-gfp ki phasmid/Pos0/img_000000000_Confocal-488-Acq_002.tif]

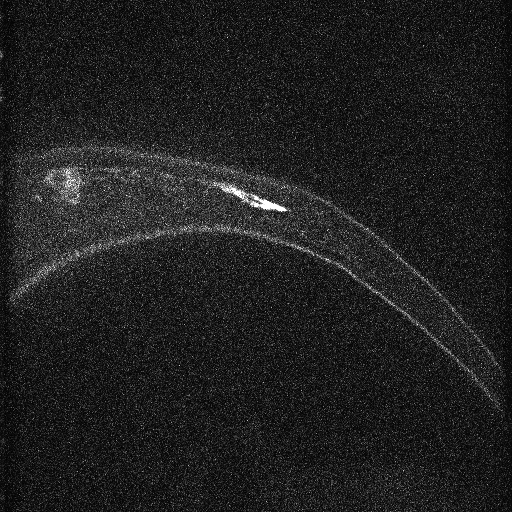

Supplement: Supplementary file 14 — Source data Fig. 5 [file 44318_2024_118_MOESM14_ESM.zip › Figure5/Figure 5C Micr. image/20211124 osm-3-R238W-gfp ki phasmid/Pos0/img_000000000_Confocal-488-Acq_003.tif]

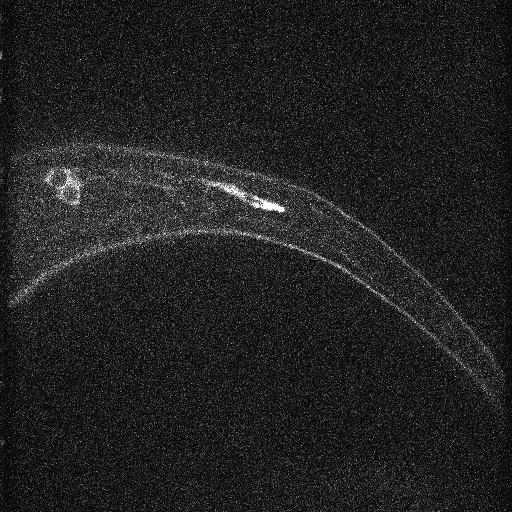

Supplement: Supplementary file 14 — Source data Fig. 5 [file 44318_2024_118_MOESM14_ESM.zip › Figure5/Figure 5C Micr. image/20211124 osm-3-R238W-gfp ki phasmid/Pos0/img_000000000_Confocal-488-Acq_004.tif]

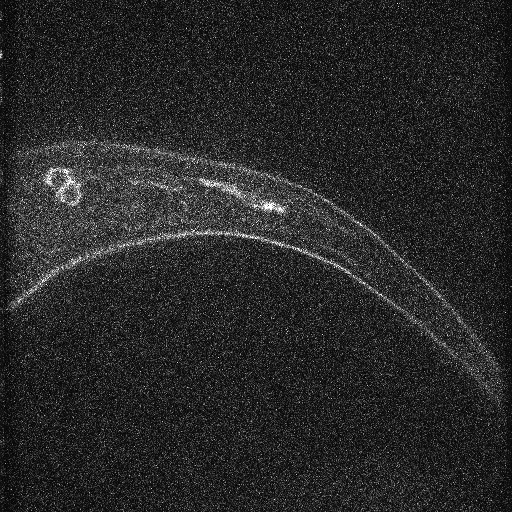

Supplement: Supplementary file 14 — Source data Fig. 5 [file 44318_2024_118_MOESM14_ESM.zip › Figure5/Figure 5C Micr. image/20211124 osm-3-R238W-gfp ki phasmid/Pos0/img_000000000_Confocal-488-Acq_005.tif]

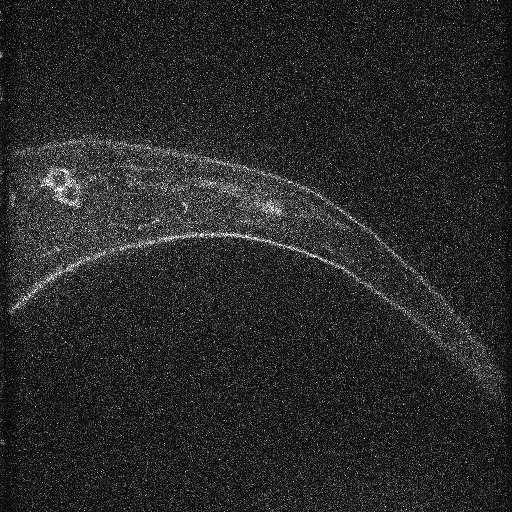

Supplement: Supplementary file 14 — Source data Fig. 5 [file 44318_2024_118_MOESM14_ESM.zip › Figure5/Figure 5C Micr. image/20211124 osm-3-R238W-gfp ki phasmid/Pos0/img_000000000_Confocal-488-Acq_006.tif]

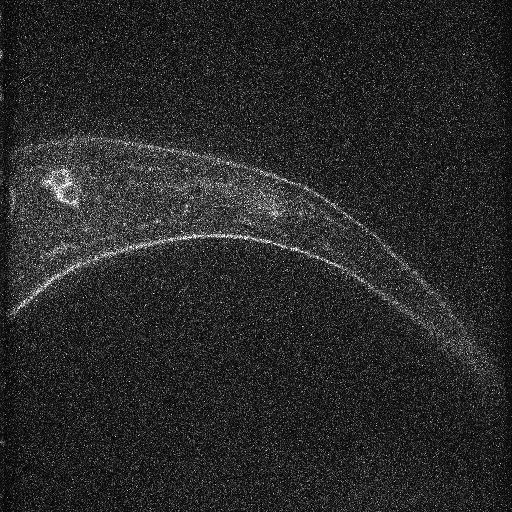

Supplement: Supplementary file 14 — Source data Fig. 5 [file 44318_2024_118_MOESM14_ESM.zip › Figure5/Figure 5C Micr. image/20211124 osm-3-R238W-gfp ki phasmid/Pos0/img_000000000_Confocal-488-Acq_007.tif]

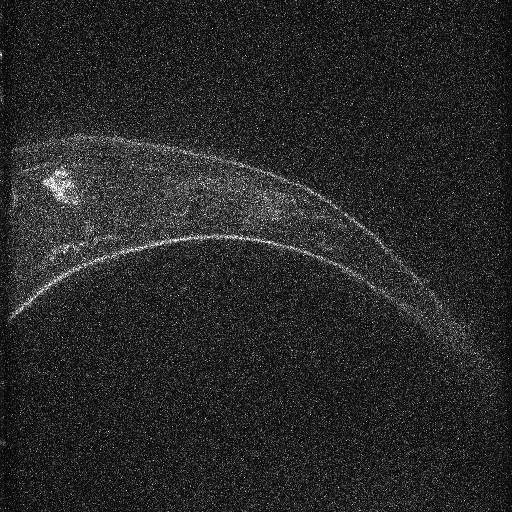

Supplement: Supplementary file 14 — Source data Fig. 5 [file 44318_2024_118_MOESM14_ESM.zip › Figure5/Figure 5C Micr. image/20211124 osm-3-R238W-gfp ki phasmid/Pos0/img_000000000_Confocal-488-Acq_008.tif]

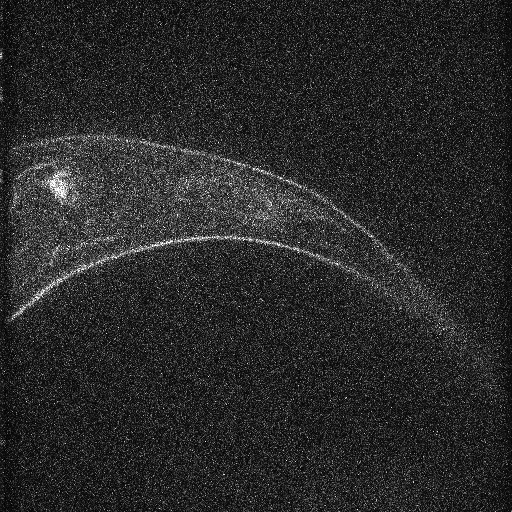

Supplement: Supplementary file 14 — Source data Fig. 5 [file 44318_2024_118_MOESM14_ESM.zip › Figure5/Figure 5C Micr. image/20211124 osm-3-R238W-gfp ki phasmid/Pos0/img_000000000_Confocal-488-Acq_009.tif]

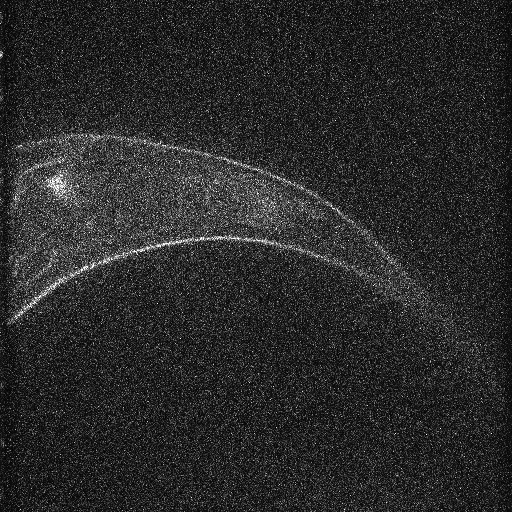

Supplement: Supplementary file 14 — Source data Fig. 5 [file 44318_2024_118_MOESM14_ESM.zip › Figure5/Figure 5C Micr. image/20211124 osm-3-R238W-gfp ki phasmid/Pos0/img_000000000_Confocal-488-Acq_010.tif]

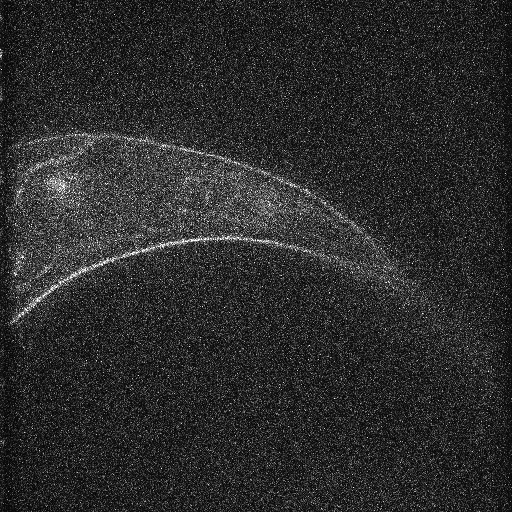

Supplement: Supplementary file 14 — Source data Fig. 5 [file 44318_2024_118_MOESM14_ESM.zip › Figure5/Figure 5C Micr. image/20211124 osm-3-R238W-gfp ki phasmid/Pos0/img_000000000_Confocal-488-Acq_011.tif]

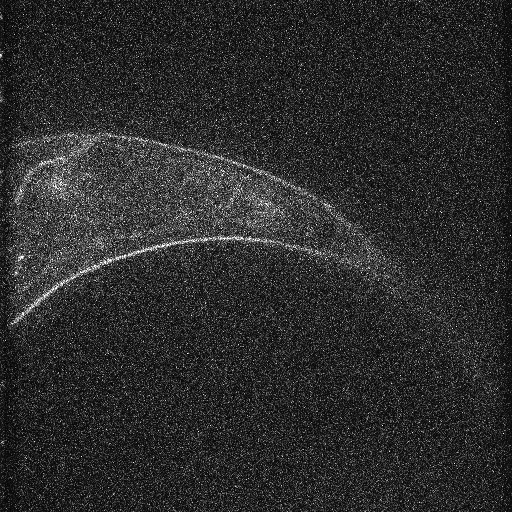

Supplement: Supplementary file 14 — Source data Fig. 5 [file 44318_2024_118_MOESM14_ESM.zip › Figure5/Figure 5C Micr. image/20211124 osm-3-R238W-gfp ki phasmid/Pos0/img_000000000_Confocal-488-Acq_012.tif]

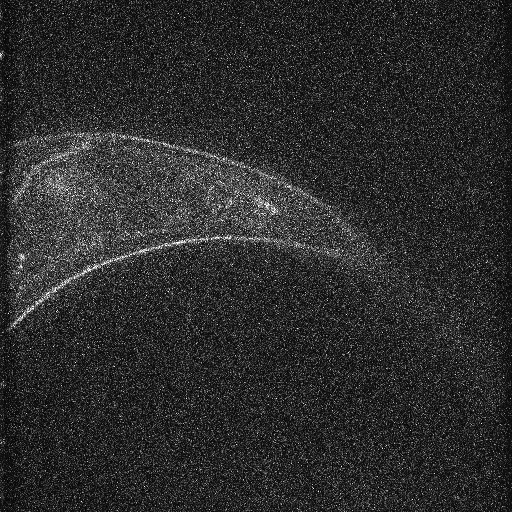

Supplement: Supplementary file 14 — Source data Fig. 5 [file 44318_2024_118_MOESM14_ESM.zip › Figure5/Figure 5C Micr. image/20211124 osm-3-R238W-gfp ki phasmid/Pos0/img_000000000_Confocal-488-Acq_013.tif]

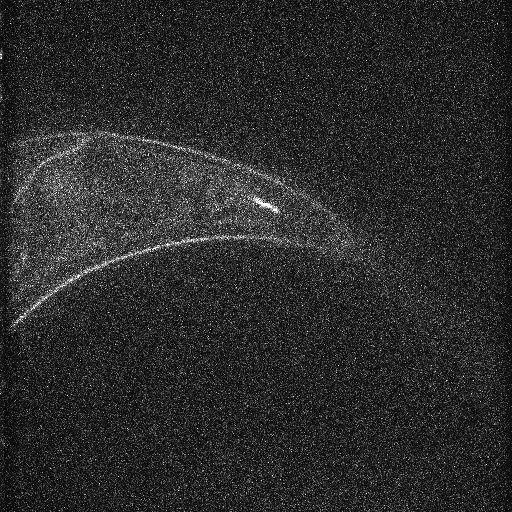

Supplement: Supplementary file 14 — Source data Fig. 5 [file 44318_2024_118_MOESM14_ESM.zip › Figure5/Figure 5C Micr. image/20211124 osm-3-R238W-gfp ki phasmid/Pos0/img_000000000_Confocal-488-Acq_014.tif]

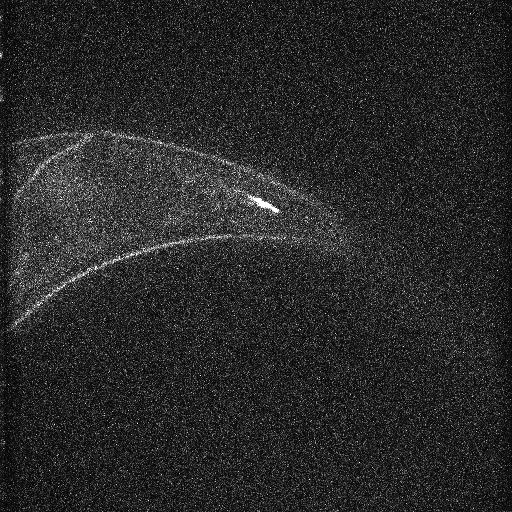

Supplement: Supplementary file 14 — Source data Fig. 5 [file 44318_2024_118_MOESM14_ESM.zip › Figure5/Figure 5C Micr. image/20211124 osm-3-R238W-gfp ki phasmid/Pos0/img_000000000_Confocal-488-Acq_015.tif]

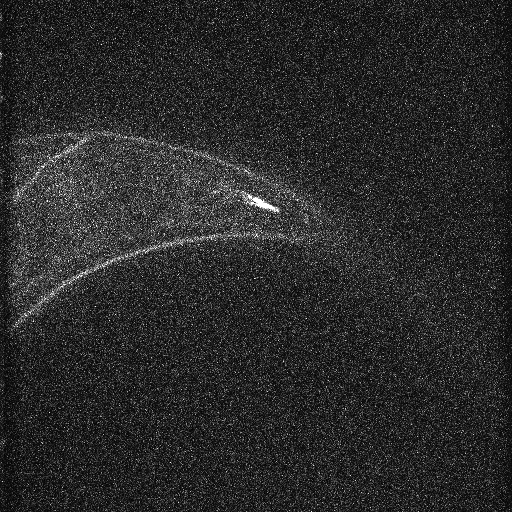

Supplement: Supplementary file 14 — Source data Fig. 5 [file 44318_2024_118_MOESM14_ESM.zip › Figure5/Figure 5C Micr. image/20211124 osm-3-R238W-gfp ki phasmid/Pos0/img_000000000_Confocal-488-Acq_016.tif]

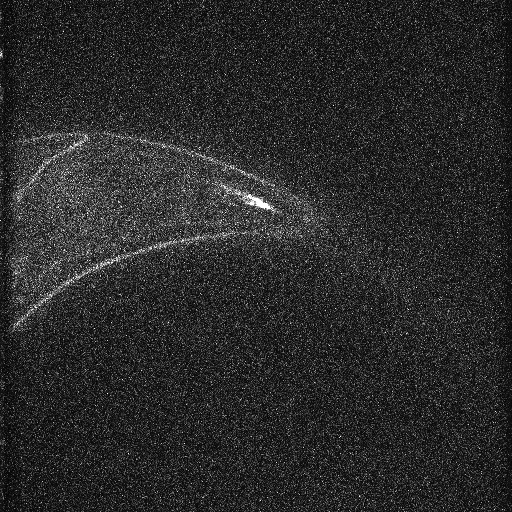

Supplement: Supplementary file 14 — Source data Fig. 5 [file 44318_2024_118_MOESM14_ESM.zip › Figure5/Figure 5C Micr. image/20211124 osm-3-R238W-gfp ki phasmid/Pos0/img_000000000_Confocal-488-Acq_017.tif]

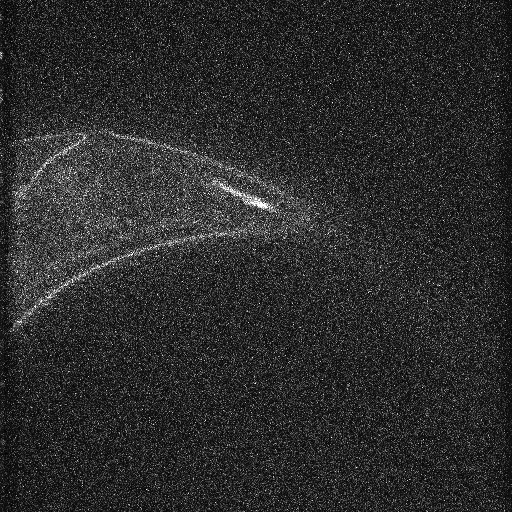

Supplement: Supplementary file 14 — Source data Fig. 5 [file 44318_2024_118_MOESM14_ESM.zip › Figure5/Figure 5C Micr. image/20211124 osm-3-R238W-gfp ki phasmid/Pos0/img_000000000_Confocal-488-Acq_018.tif]

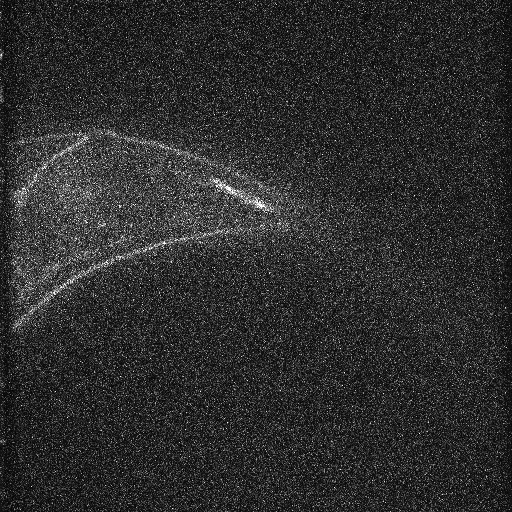

Supplement: Supplementary file 14 — Source data Fig. 5 [file 44318_2024_118_MOESM14_ESM.zip › Figure5/Figure 5C Micr. image/20211124 osm-3-R238W-gfp ki phasmid/Pos0/img_000000000_Confocal-488-Acq_019.tif]

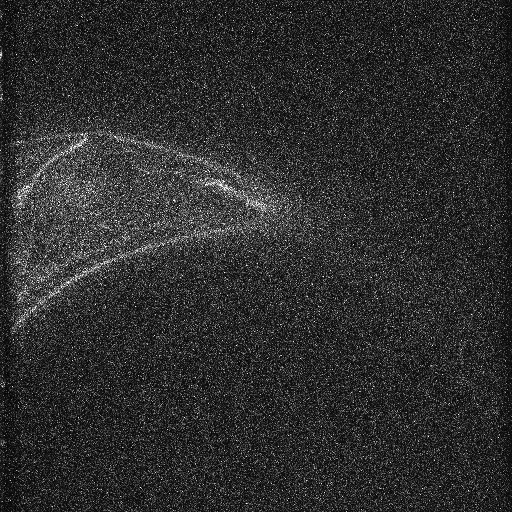

Supplement: Supplementary file 14 — Source data Fig. 5 [file 44318_2024_118_MOESM14_ESM.zip › Figure5/Figure 5C Micr. image/20211124 osm-3-R238W-gfp ki phasmid/Pos0/img_000000000_Confocal-488-Acq_020.tif]

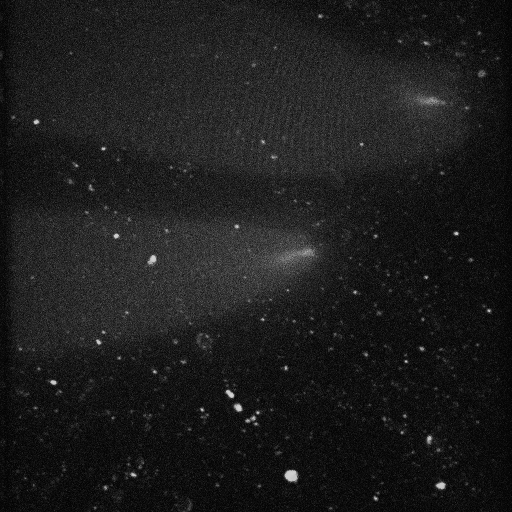

Supplement: Supplementary file 14 — Source data Fig. 5 [file 44318_2024_118_MOESM14_ESM.zip › Figure5/Figure 5C Micr. image/20211213 osm-3-H207Q-G444E-gfp sup_37 amphid/Pos0/img_000000000_Confocal-488-Acq_000.tif]

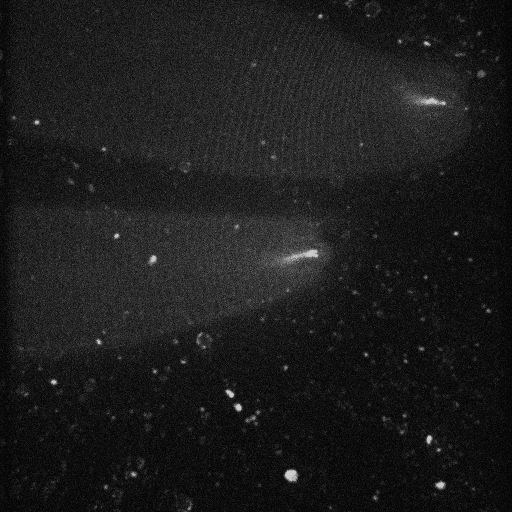

Supplement: Supplementary file 14 — Source data Fig. 5 [file 44318_2024_118_MOESM14_ESM.zip › Figure5/Figure 5C Micr. image/20211213 osm-3-H207Q-G444E-gfp sup_37 amphid/Pos0/img_000000000_Confocal-488-Acq_001.tif]

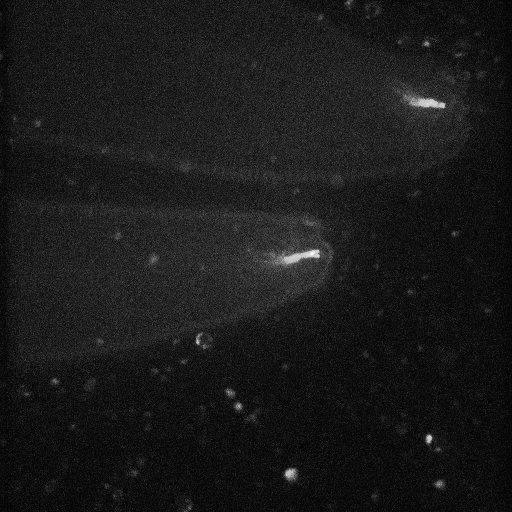

Supplement: Supplementary file 14 — Source data Fig. 5 [file 44318_2024_118_MOESM14_ESM.zip › Figure5/Figure 5C Micr. image/20211213 osm-3-H207Q-G444E-gfp sup_37 amphid/Pos0/img_000000000_Confocal-488-Acq_002.tif]

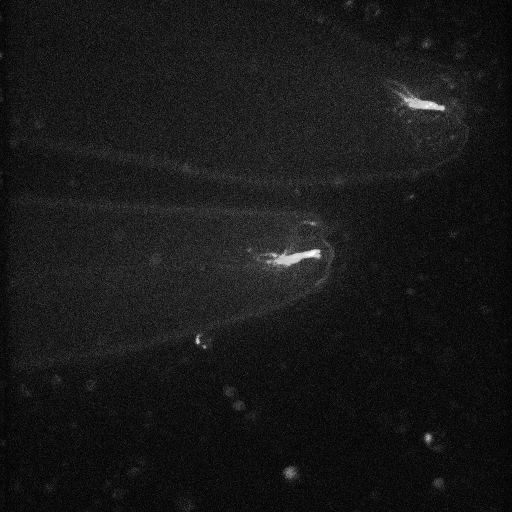

Supplement: Supplementary file 14 — Source data Fig. 5 [file 44318_2024_118_MOESM14_ESM.zip › Figure5/Figure 5C Micr. image/20211213 osm-3-H207Q-G444E-gfp sup_37 amphid/Pos0/img_000000000_Confocal-488-Acq_003.tif]

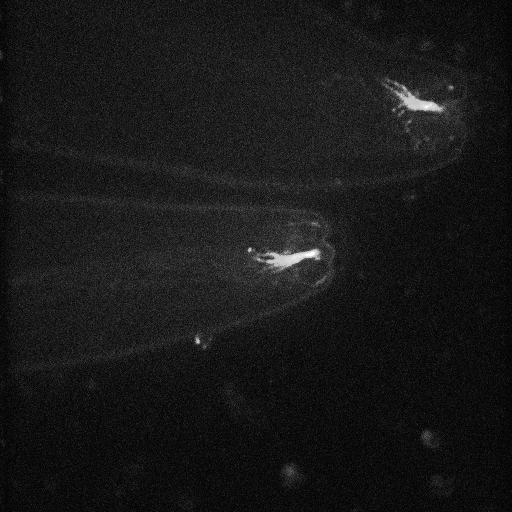

Supplement: Supplementary file 14 — Source data Fig. 5 [file 44318_2024_118_MOESM14_ESM.zip › Figure5/Figure 5C Micr. image/20211213 osm-3-H207Q-G444E-gfp sup_37 amphid/Pos0/img_000000000_Confocal-488-Acq_004.tif]

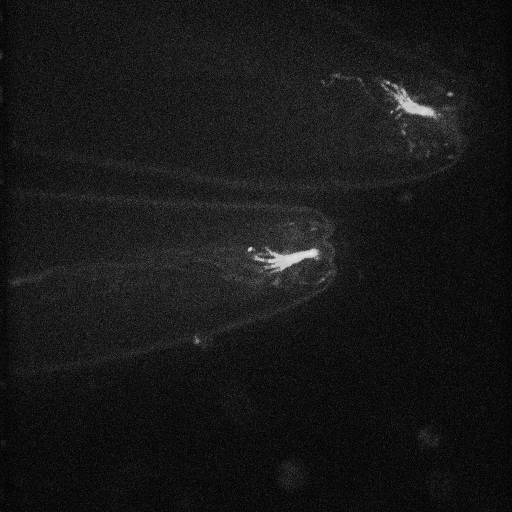

Supplement: Supplementary file 14 — Source data Fig. 5 [file 44318_2024_118_MOESM14_ESM.zip › Figure5/Figure 5C Micr. image/20211213 osm-3-H207Q-G444E-gfp sup_37 amphid/Pos0/img_000000000_Confocal-488-Acq_005.tif]

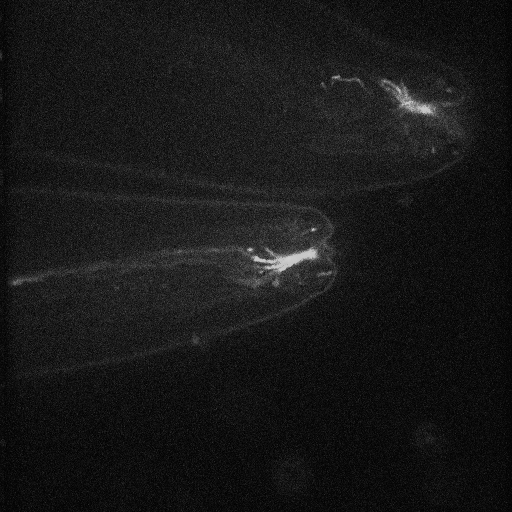

Supplement: Supplementary file 14 — Source data Fig. 5 [file 44318_2024_118_MOESM14_ESM.zip › Figure5/Figure 5C Micr. image/20211213 osm-3-H207Q-G444E-gfp sup_37 amphid/Pos0/img_000000000_Confocal-488-Acq_006.tif]

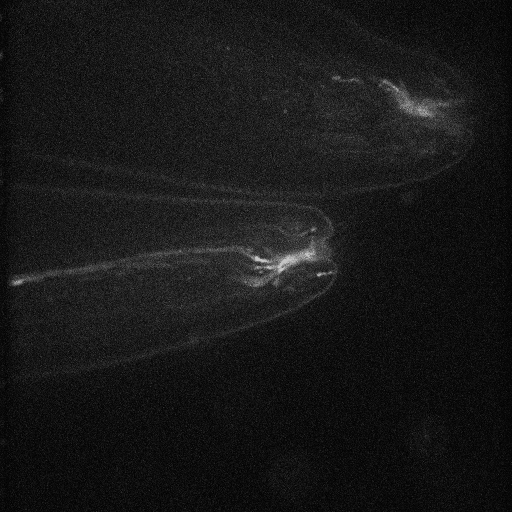

Supplement: Supplementary file 14 — Source data Fig. 5 [file 44318_2024_118_MOESM14_ESM.zip › Figure5/Figure 5C Micr. image/20211213 osm-3-H207Q-G444E-gfp sup_37 amphid/Pos0/img_000000000_Confocal-488-Acq_007.tif]

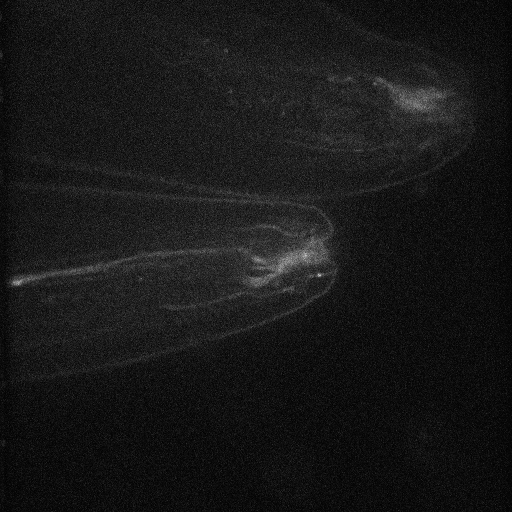

Supplement: Supplementary file 14 — Source data Fig. 5 [file 44318_2024_118_MOESM14_ESM.zip › Figure5/Figure 5C Micr. image/20211213 osm-3-H207Q-G444E-gfp sup_37 amphid/Pos0/img_000000000_Confocal-488-Acq_008.tif]

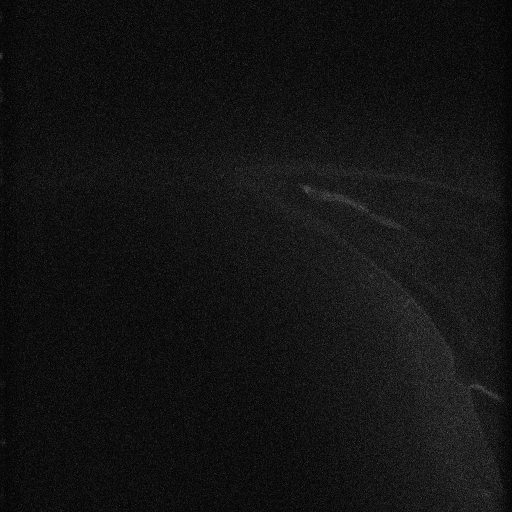

Supplement: Supplementary file 14 — Source data Fig. 5 [file 44318_2024_118_MOESM14_ESM.zip › Figure5/Figure 5C Micr. image/20211213 osm-3-H207Q-G444E-gfp sup_39 phasmid/Pos0/img_000000000_Confocal-488-Acq_000.tif]

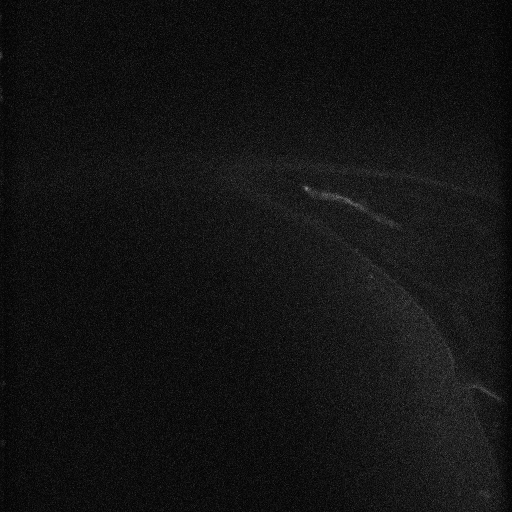

Supplement: Supplementary file 14 — Source data Fig. 5 [file 44318_2024_118_MOESM14_ESM.zip › Figure5/Figure 5C Micr. image/20211213 osm-3-H207Q-G444E-gfp sup_39 phasmid/Pos0/img_000000000_Confocal-488-Acq_001.tif]

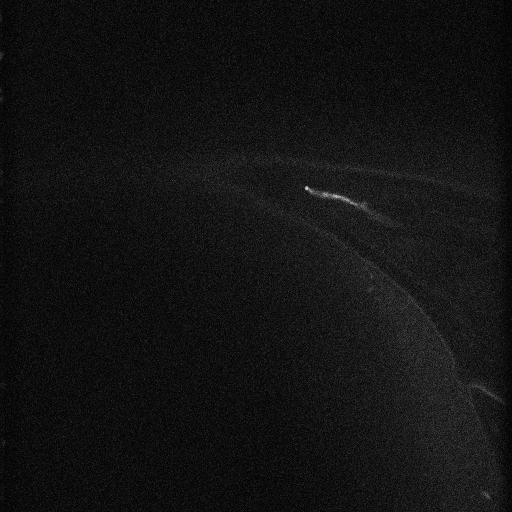

Supplement: Supplementary file 14 — Source data Fig. 5 [file 44318_2024_118_MOESM14_ESM.zip › Figure5/Figure 5C Micr. image/20211213 osm-3-H207Q-G444E-gfp sup_39 phasmid/Pos0/img_000000000_Confocal-488-Acq_002.tif]

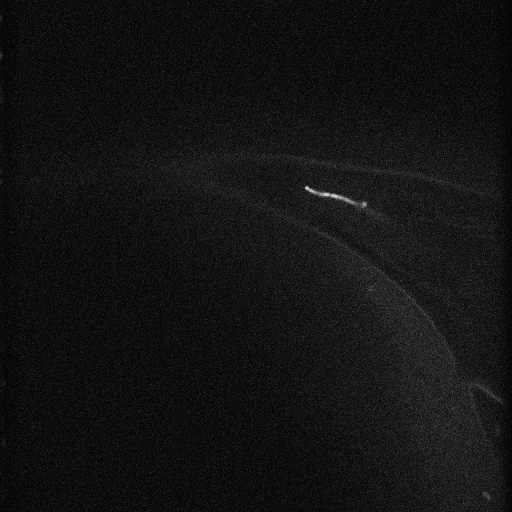

Supplement: Supplementary file 14 — Source data Fig. 5 [file 44318_2024_118_MOESM14_ESM.zip › Figure5/Figure 5C Micr. image/20211213 osm-3-H207Q-G444E-gfp sup_39 phasmid/Pos0/img_000000000_Confocal-488-Acq_003.tif]

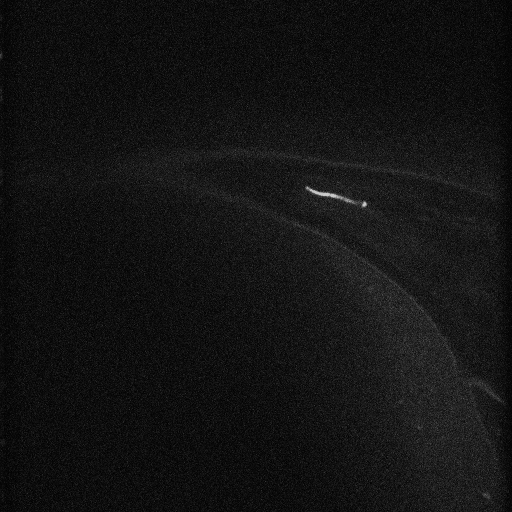

Supplement: Supplementary file 14 — Source data Fig. 5 [file 44318_2024_118_MOESM14_ESM.zip › Figure5/Figure 5C Micr. image/20211213 osm-3-H207Q-G444E-gfp sup_39 phasmid/Pos0/img_000000000_Confocal-488-Acq_004.tif]

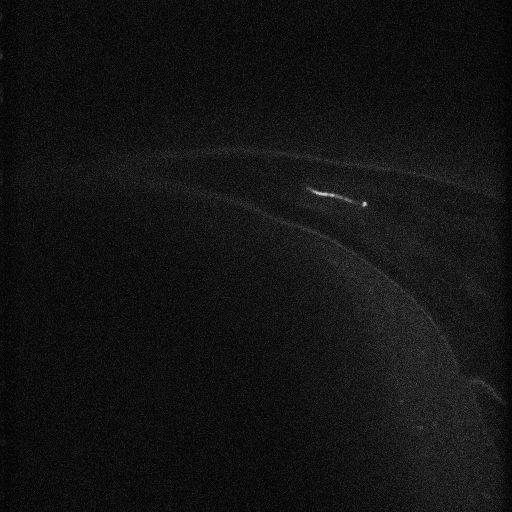

Supplement: Supplementary file 14 — Source data Fig. 5 [file 44318_2024_118_MOESM14_ESM.zip › Figure5/Figure 5C Micr. image/20211213 osm-3-H207Q-G444E-gfp sup_39 phasmid/Pos0/img_000000000_Confocal-488-Acq_005.tif]

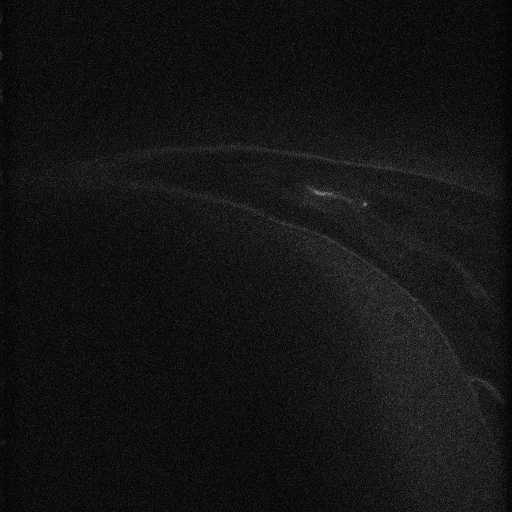

Supplement: Supplementary file 14 — Source data Fig. 5 [file 44318_2024_118_MOESM14_ESM.zip › Figure5/Figure 5C Micr. image/20211213 osm-3-H207Q-G444E-gfp sup_39 phasmid/Pos0/img_000000000_Confocal-488-Acq_006.tif]

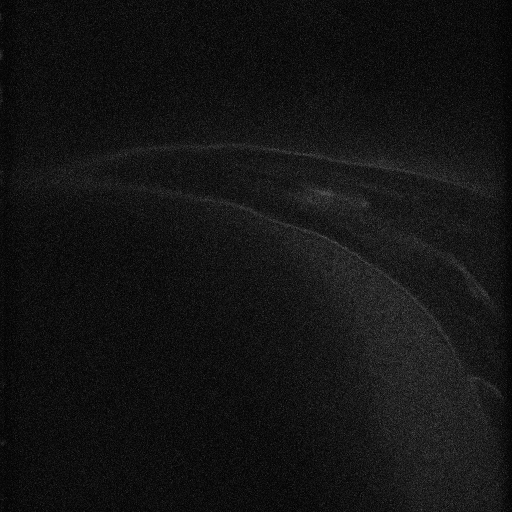

Supplement: Supplementary file 14 — Source data Fig. 5 [file 44318_2024_118_MOESM14_ESM.zip › Figure5/Figure 5C Micr. image/20211213 osm-3-H207Q-G444E-gfp sup_39 phasmid/Pos0/img_000000000_Confocal-488-Acq_007.tif]

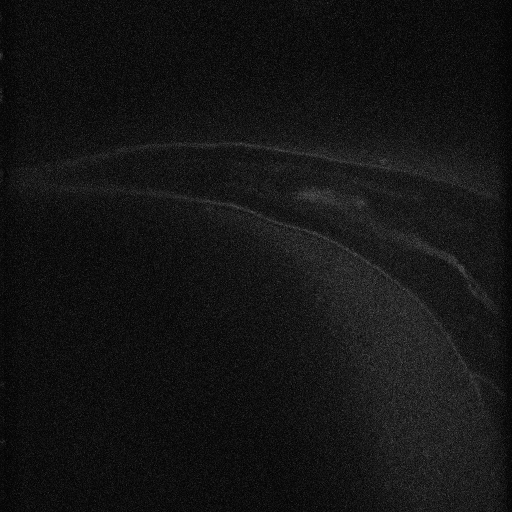

Supplement: Supplementary file 14 — Source data Fig. 5 [file 44318_2024_118_MOESM14_ESM.zip › Figure5/Figure 5C Micr. image/20211213 osm-3-H207Q-G444E-gfp sup_39 phasmid/Pos0/img_000000000_Confocal-488-Acq_008.tif]

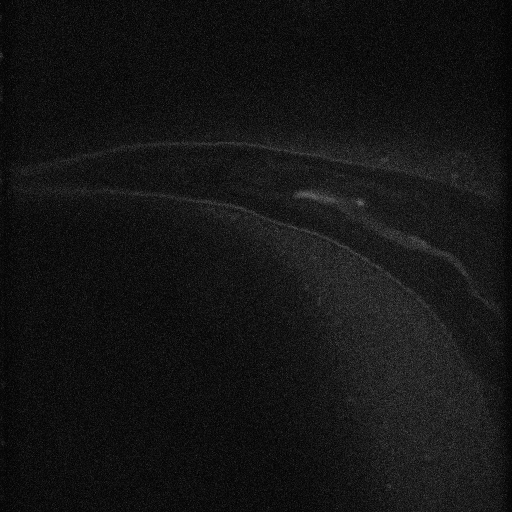

Supplement: Supplementary file 14 — Source data Fig. 5 [file 44318_2024_118_MOESM14_ESM.zip › Figure5/Figure 5C Micr. image/20211213 osm-3-H207Q-G444E-gfp sup_39 phasmid/Pos0/img_000000000_Confocal-488-Acq_009.tif]

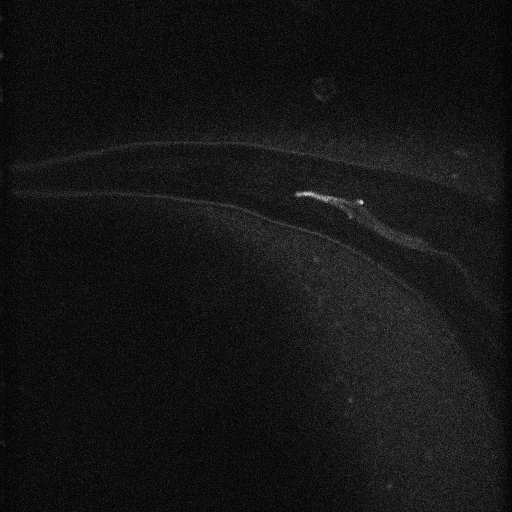

Supplement: Supplementary file 14 — Source data Fig. 5 [file 44318_2024_118_MOESM14_ESM.zip › Figure5/Figure 5C Micr. image/20211213 osm-3-H207Q-G444E-gfp sup_39 phasmid/Pos0/img_000000000_Confocal-488-Acq_010.tif]

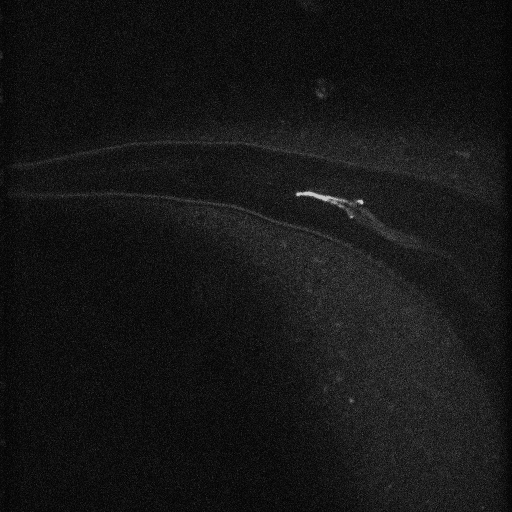

Supplement: Supplementary file 14 — Source data Fig. 5 [file 44318_2024_118_MOESM14_ESM.zip › Figure5/Figure 5C Micr. image/20211213 osm-3-H207Q-G444E-gfp sup_39 phasmid/Pos0/img_000000000_Confocal-488-Acq_011.tif]

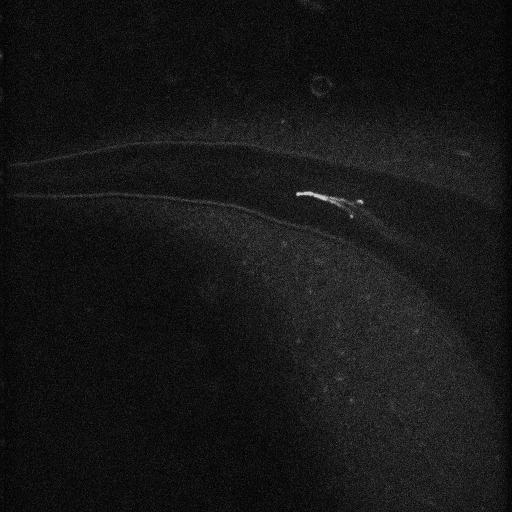

Supplement: Supplementary file 14 — Source data Fig. 5 [file 44318_2024_118_MOESM14_ESM.zip › Figure5/Figure 5C Micr. image/20211213 osm-3-H207Q-G444E-gfp sup_39 phasmid/Pos0/img_000000000_Confocal-488-Acq_012.tif]

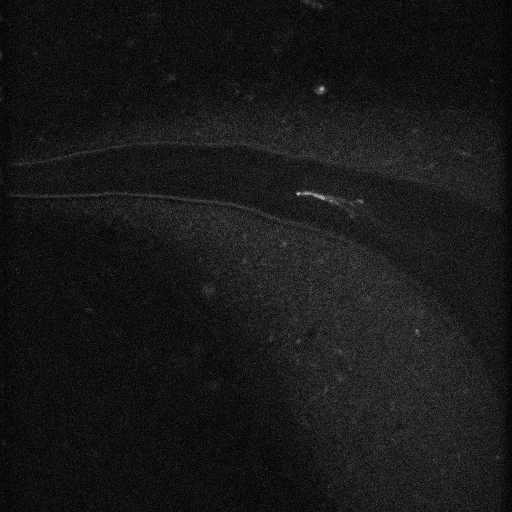

Supplement: Supplementary file 14 — Source data Fig. 5 [file 44318_2024_118_MOESM14_ESM.zip › Figure5/Figure 5C Micr. image/20211213 osm-3-H207Q-G444E-gfp sup_39 phasmid/Pos0/img_000000000_Confocal-488-Acq_013.tif]

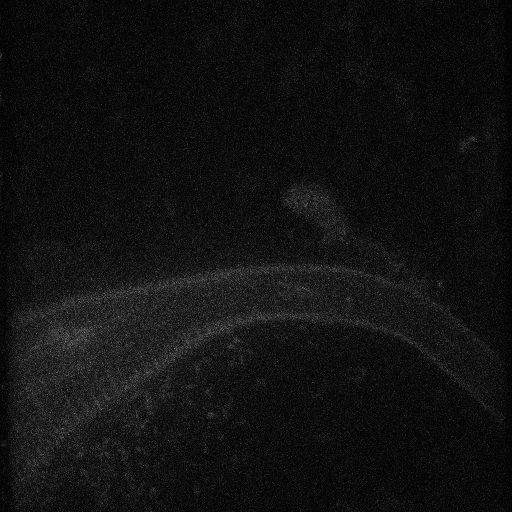

Supplement: Supplementary file 14 — Source data Fig. 5 [file 44318_2024_118_MOESM14_ESM.zip › Figure5/Figure 5C Micr. image/20220302 osm-3-H207Q-gfp ki_2 phasmid/Pos0/img_000000000_Confocal-488-Acq_000.tif]

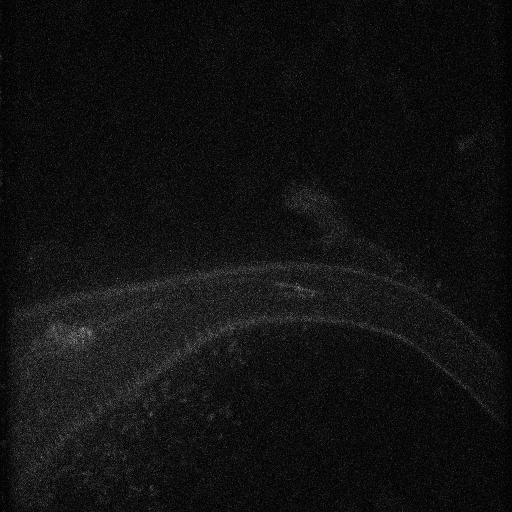

Supplement: Supplementary file 14 — Source data Fig. 5 [file 44318_2024_118_MOESM14_ESM.zip › Figure5/Figure 5C Micr. image/20220302 osm-3-H207Q-gfp ki_2 phasmid/Pos0/img_000000000_Confocal-488-Acq_001.tif]

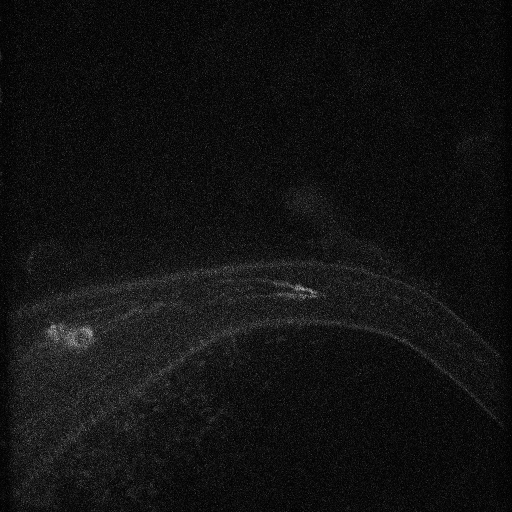

Supplement: Supplementary file 14 — Source data Fig. 5 [file 44318_2024_118_MOESM14_ESM.zip › Figure5/Figure 5C Micr. image/20220302 osm-3-H207Q-gfp ki_2 phasmid/Pos0/img_000000000_Confocal-488-Acq_002.tif]

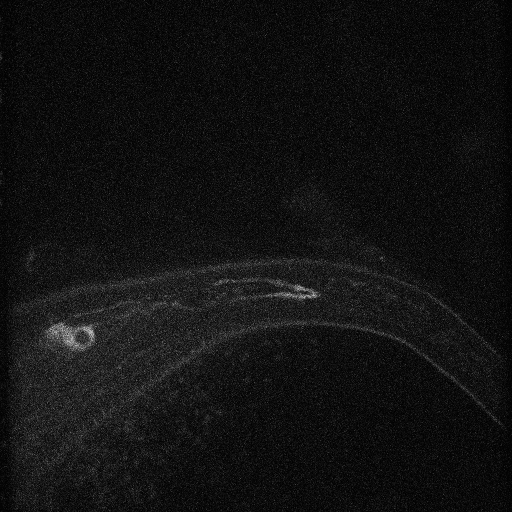

Supplement: Supplementary file 14 — Source data Fig. 5 [file 44318_2024_118_MOESM14_ESM.zip › Figure5/Figure 5C Micr. image/20220302 osm-3-H207Q-gfp ki_2 phasmid/Pos0/img_000000000_Confocal-488-Acq_003.tif]

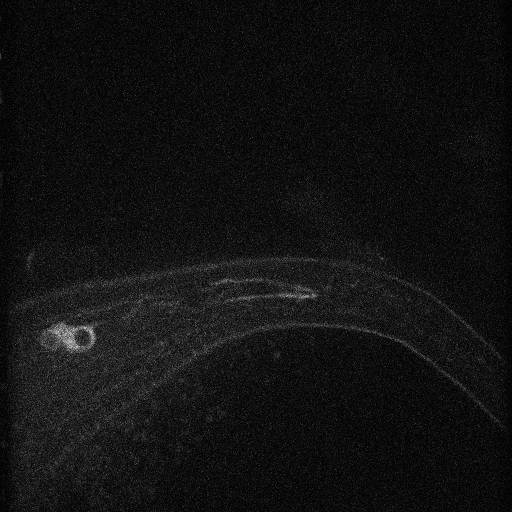

Supplement: Supplementary file 14 — Source data Fig. 5 [file 44318_2024_118_MOESM14_ESM.zip › Figure5/Figure 5C Micr. image/20220302 osm-3-H207Q-gfp ki_2 phasmid/Pos0/img_000000000_Confocal-488-Acq_004.tif]

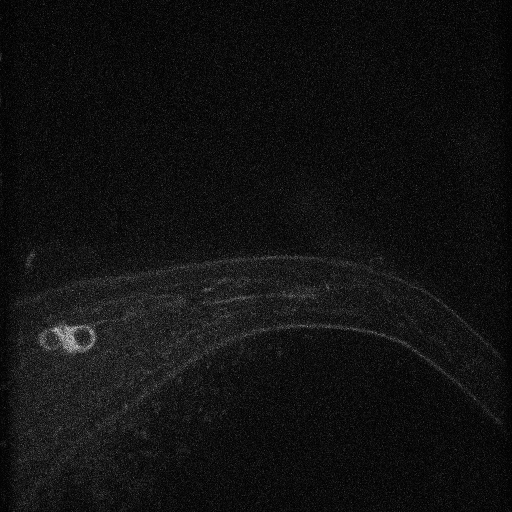

Supplement: Supplementary file 14 — Source data Fig. 5 [file 44318_2024_118_MOESM14_ESM.zip › Figure5/Figure 5C Micr. image/20220302 osm-3-H207Q-gfp ki_2 phasmid/Pos0/img_000000000_Confocal-488-Acq_005.tif]

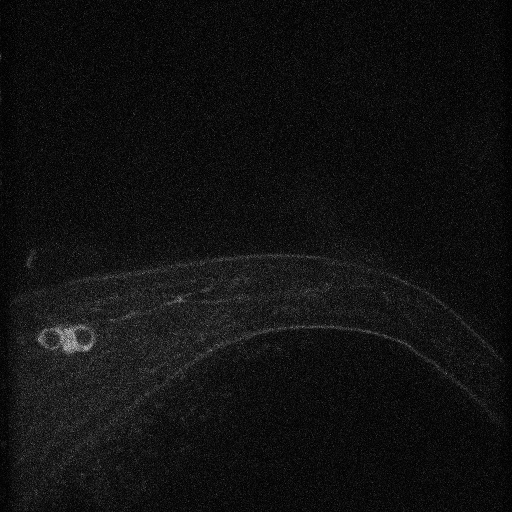

Supplement: Supplementary file 14 — Source data Fig. 5 [file 44318_2024_118_MOESM14_ESM.zip › Figure5/Figure 5C Micr. image/20220302 osm-3-H207Q-gfp ki_2 phasmid/Pos0/img_000000000_Confocal-488-Acq_006.tif]

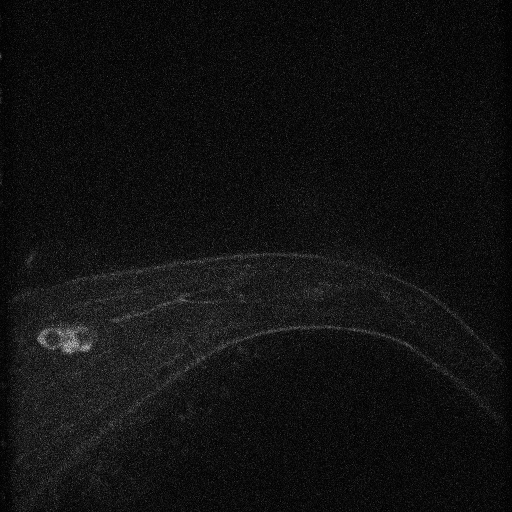

Supplement: Supplementary file 14 — Source data Fig. 5 [file 44318_2024_118_MOESM14_ESM.zip › Figure5/Figure 5C Micr. image/20220302 osm-3-H207Q-gfp ki_2 phasmid/Pos0/img_000000000_Confocal-488-Acq_007.tif]

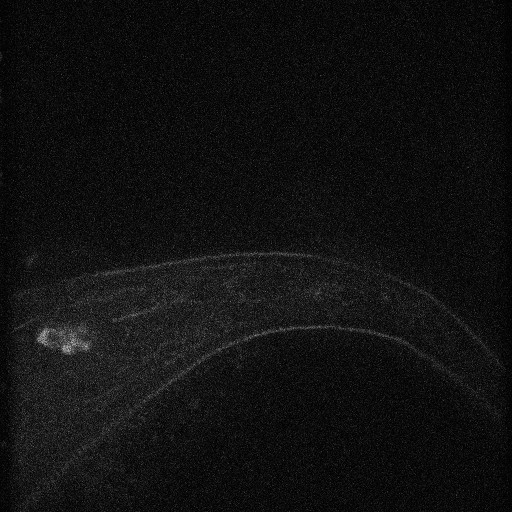

Supplement: Supplementary file 14 — Source data Fig. 5 [file 44318_2024_118_MOESM14_ESM.zip › Figure5/Figure 5C Micr. image/20220302 osm-3-H207Q-gfp ki_2 phasmid/Pos0/img_000000000_Confocal-488-Acq_008.tif]

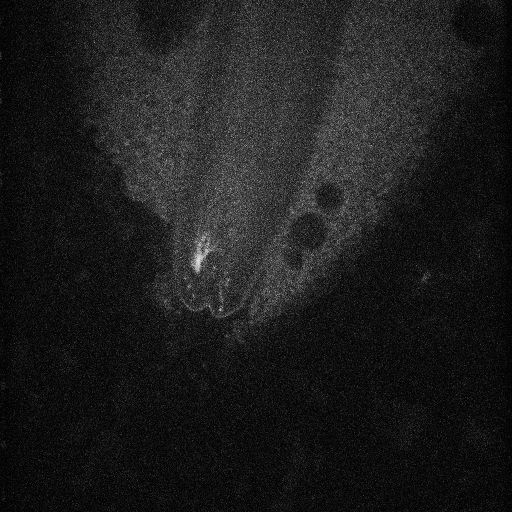

Supplement: Supplementary file 14 — Source data Fig. 5 [file 44318_2024_118_MOESM14_ESM.zip › Figure5/Figure 5C Micr. image/20220302 osm-3-H207Q-gfp ki_3 amphid/Pos0/img_000000000_Confocal-488-Acq_000.tif]

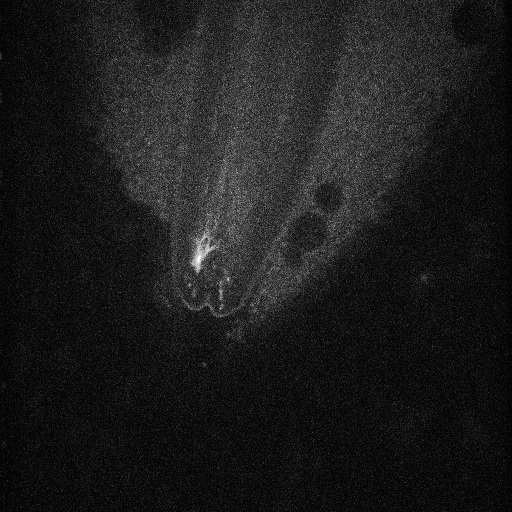

Supplement: Supplementary file 14 — Source data Fig. 5 [file 44318_2024_118_MOESM14_ESM.zip › Figure5/Figure 5C Micr. image/20220302 osm-3-H207Q-gfp ki_3 amphid/Pos0/img_000000000_Confocal-488-Acq_001.tif]

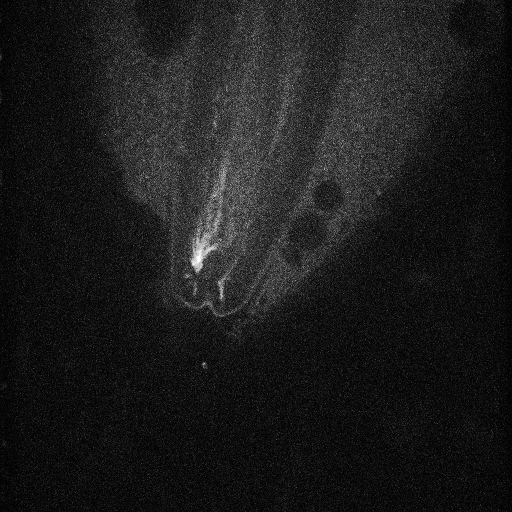

Supplement: Supplementary file 14 — Source data Fig. 5 [file 44318_2024_118_MOESM14_ESM.zip › Figure5/Figure 5C Micr. image/20220302 osm-3-H207Q-gfp ki_3 amphid/Pos0/img_000000000_Confocal-488-Acq_002.tif]

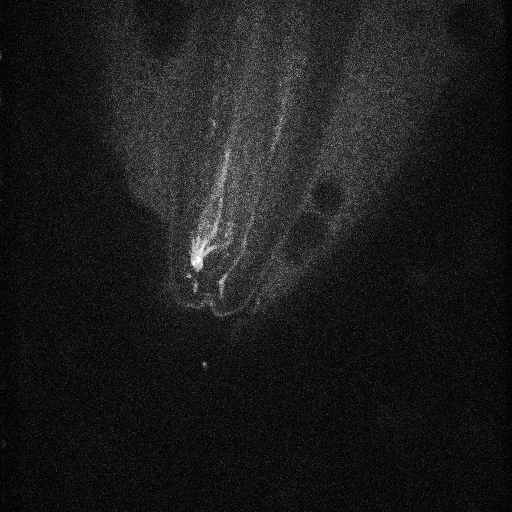

Supplement: Supplementary file 14 — Source data Fig. 5 [file 44318_2024_118_MOESM14_ESM.zip › Figure5/Figure 5C Micr. image/20220302 osm-3-H207Q-gfp ki_3 amphid/Pos0/img_000000000_Confocal-488-Acq_003.tif]

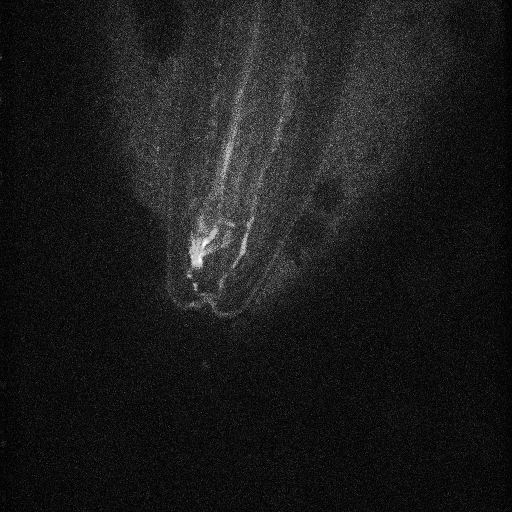

Supplement: Supplementary file 14 — Source data Fig. 5 [file 44318_2024_118_MOESM14_ESM.zip › Figure5/Figure 5C Micr. image/20220302 osm-3-H207Q-gfp ki_3 amphid/Pos0/img_000000000_Confocal-488-Acq_004.tif]

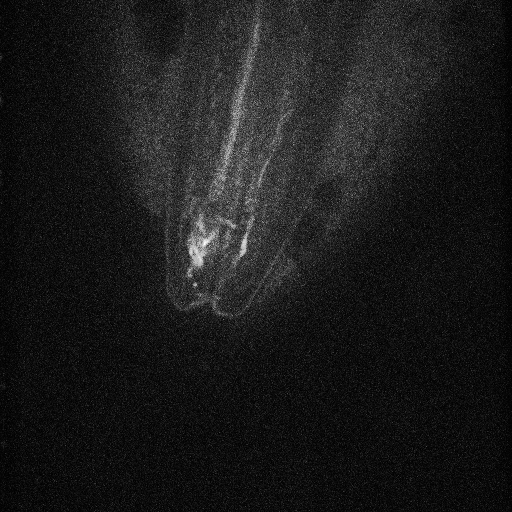

Supplement: Supplementary file 14 — Source data Fig. 5 [file 44318_2024_118_MOESM14_ESM.zip › Figure5/Figure 5C Micr. image/20220302 osm-3-H207Q-gfp ki_3 amphid/Pos0/img_000000000_Confocal-488-Acq_005.tif]

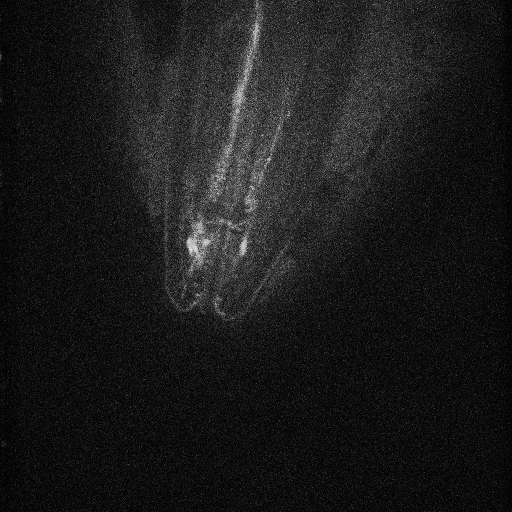

Supplement: Supplementary file 14 — Source data Fig. 5 [file 44318_2024_118_MOESM14_ESM.zip › Figure5/Figure 5C Micr. image/20220302 osm-3-H207Q-gfp ki_3 amphid/Pos0/img_000000000_Confocal-488-Acq_006.tif]

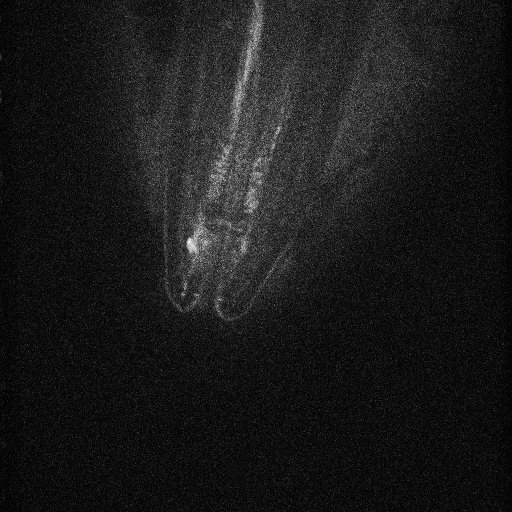

Supplement: Supplementary file 14 — Source data Fig. 5 [file 44318_2024_118_MOESM14_ESM.zip › Figure5/Figure 5C Micr. image/20220302 osm-3-H207Q-gfp ki_3 amphid/Pos0/img_000000000_Confocal-488-Acq_007.tif]

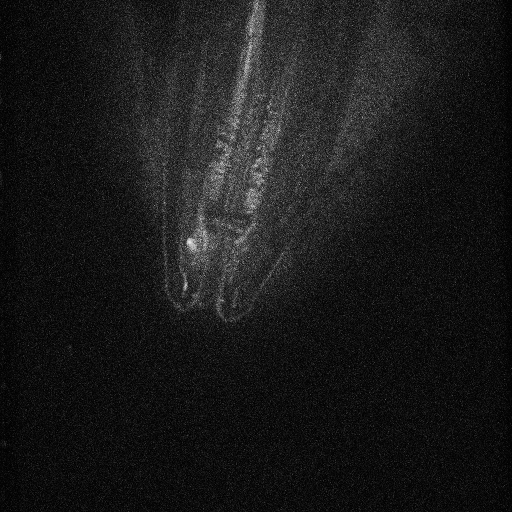

Supplement: Supplementary file 14 — Source data Fig. 5 [file 44318_2024_118_MOESM14_ESM.zip › Figure5/Figure 5C Micr. image/20220302 osm-3-H207Q-gfp ki_3 amphid/Pos0/img_000000000_Confocal-488-Acq_008.tif]

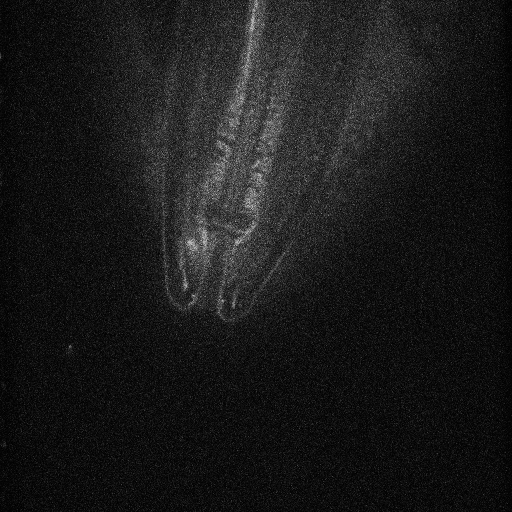

Supplement: Supplementary file 14 — Source data Fig. 5 [file 44318_2024_118_MOESM14_ESM.zip › Figure5/Figure 5C Micr. image/20220302 osm-3-H207Q-gfp ki_3 amphid/Pos0/img_000000000_Confocal-488-Acq_009.tif]

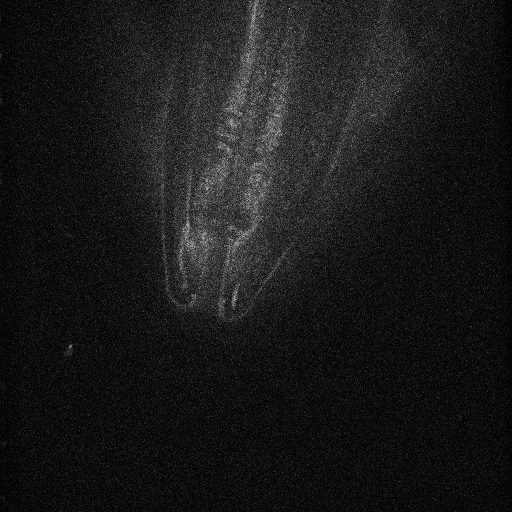

Supplement: Supplementary file 14 — Source data Fig. 5 [file 44318_2024_118_MOESM14_ESM.zip › Figure5/Figure 5C Micr. image/20220302 osm-3-H207Q-gfp ki_3 amphid/Pos0/img_000000000_Confocal-488-Acq_010.tif]

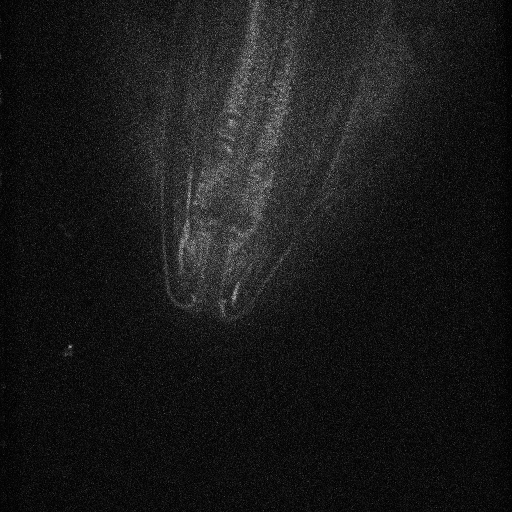

Supplement: Supplementary file 14 — Source data Fig. 5 [file 44318_2024_118_MOESM14_ESM.zip › Figure5/Figure 5C Micr. image/20220302 osm-3-H207Q-gfp ki_3 amphid/Pos0/img_000000000_Confocal-488-Acq_011.tif]

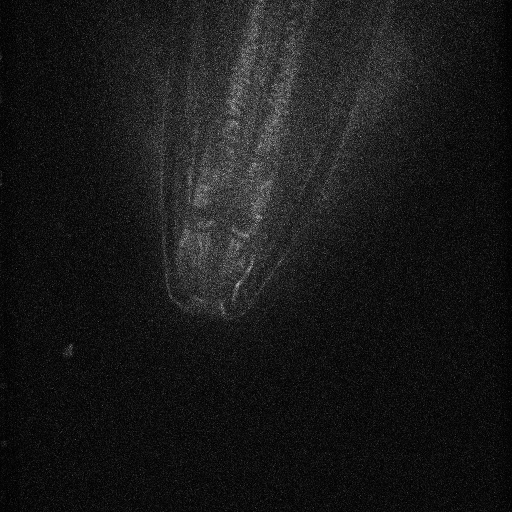

Supplement: Supplementary file 14 — Source data Fig. 5 [file 44318_2024_118_MOESM14_ESM.zip › Figure5/Figure 5C Micr. image/20220302 osm-3-H207Q-gfp ki_3 amphid/Pos0/img_000000000_Confocal-488-Acq_012.tif]

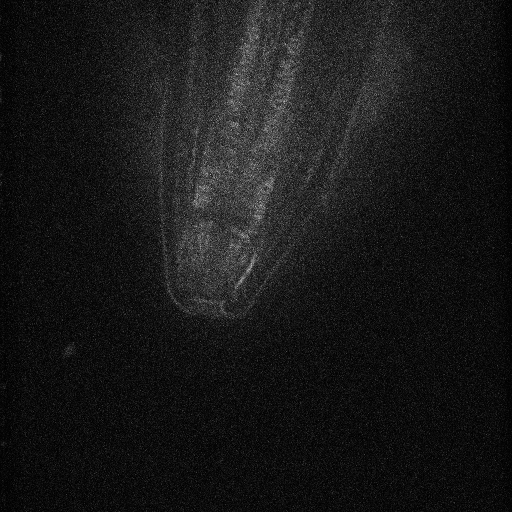

Supplement: Supplementary file 14 — Source data Fig. 5 [file 44318_2024_118_MOESM14_ESM.zip › Figure5/Figure 5C Micr. image/20220302 osm-3-H207Q-gfp ki_3 amphid/Pos0/img_000000000_Confocal-488-Acq_013.tif]

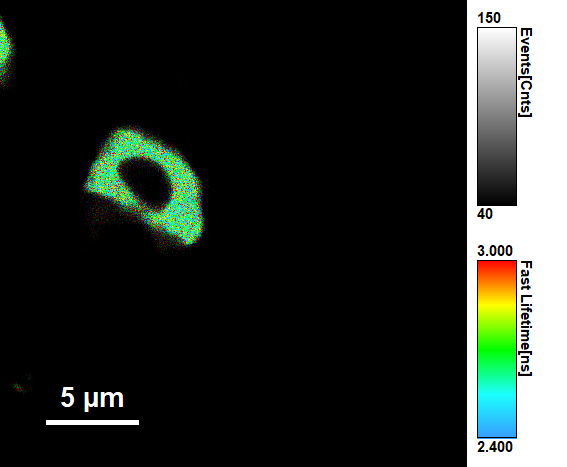

Supplement: Supplementary file 15 — Source data Fig. 6 [file 44318_2024_118_MOESM15_ESM.zip › Figure6/Figure 6B Micr. image/mScarlet-osm-3-gfp.bmp]

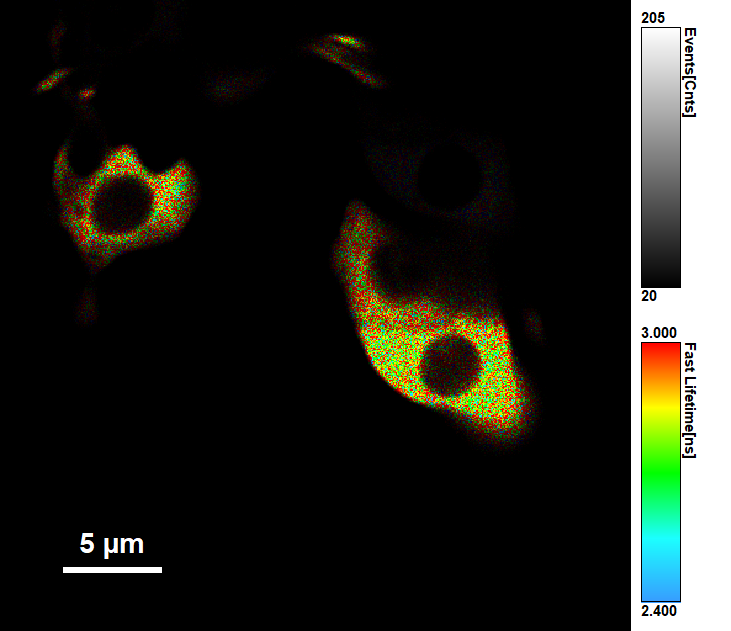

Supplement: Supplementary file 15 — Source data Fig. 6 [file 44318_2024_118_MOESM15_ESM.zip › Figure6/Figure 6B Micr. image/osm-3-gfp.bmp]

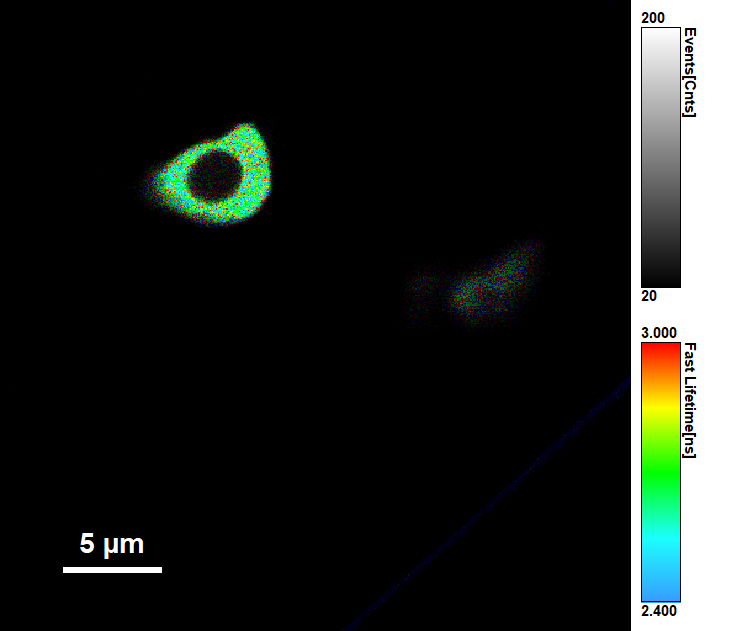

Supplement: Supplementary file 15 — Source data Fig. 6 [file 44318_2024_118_MOESM15_ESM.zip › Figure6/Figure 6B Micr. image/osm-3-gfp-mScarlet.bmp]

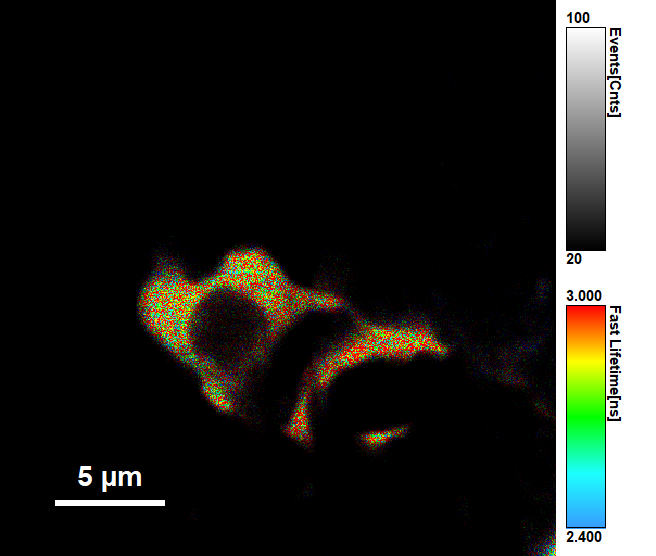

Supplement: Supplementary file 15 — Source data Fig. 6 [file 44318_2024_118_MOESM15_ESM.zip › Figure6/Figure 6D Micr. image/G444E.bmp]

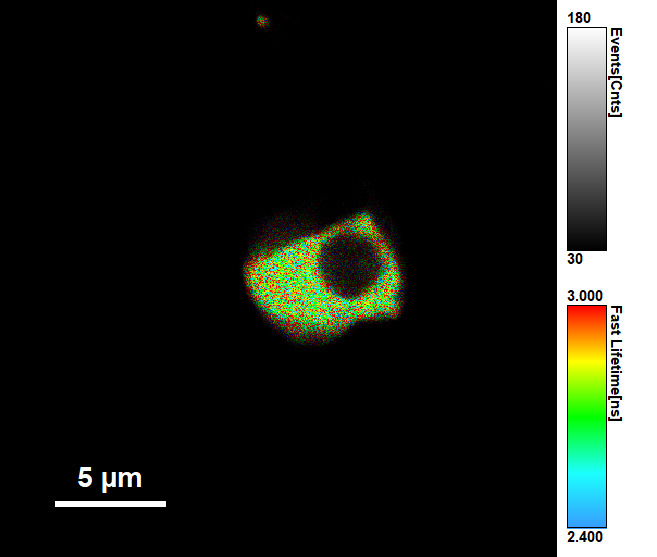

Supplement: Supplementary file 15 — Source data Fig. 6 [file 44318_2024_118_MOESM15_ESM.zip › Figure6/Figure 6D Micr. image/h207q-g444e.bmp]

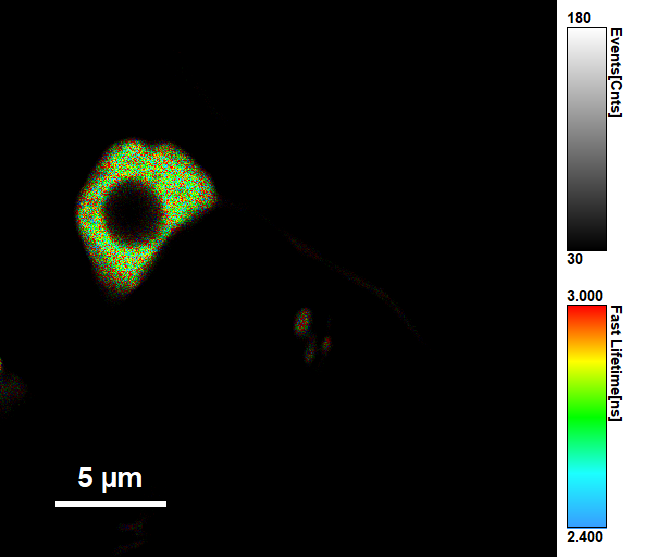

Supplement: Supplementary file 15 — Source data Fig. 6 [file 44318_2024_118_MOESM15_ESM.zip › Figure6/Figure 6D Micr. image/r238w-g444e.bmp]

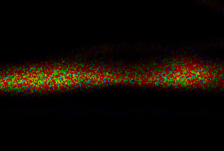

Supplement: Supplementary file 15 — Source data Fig. 6 [file 44318_2024_118_MOESM15_ESM.zip › Figure6/Figure 6F Micr. image/gfp.tif]

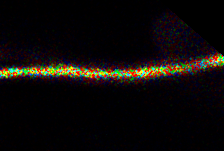

Supplement: Supplementary file 15 — Source data Fig. 6 [file 44318_2024_118_MOESM15_ESM.zip › Figure6/Figure 6F Micr. image/mScarlet-osm-3-gfp.tif]

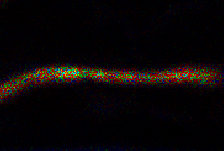

Supplement: Supplementary file 15 — Source data Fig. 6 [file 44318_2024_118_MOESM15_ESM.zip › Figure6/Figure 6F Micr. image/osm-3-gfp.tif]

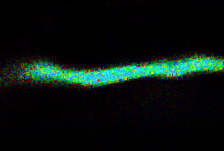

Supplement: Supplementary file 15 — Source data Fig. 6 [file 44318_2024_118_MOESM15_ESM.zip › Figure6/Figure 6F Micr. image/scarlet-gfp.tif]

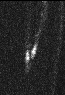

Supplement: Supplementary file 16 — Source data Fig. 7 [file 44318_2024_118_MOESM16_ESM.zip › Figure7/Figure 7A Micr. image/20210831 osm-3-g235a-gfp ki_8-1 movie for kymograph.tif]
