# Supplementary figures and images for: Neurons dispose of hyperactive kinesin into glial cells for clearance (part 9 of 9)
Source: EMBO J. 2024 May 28;43(13):5. doi: 10.1038/s44318-024-00118-0 (PMC11217292; doi:10.1038/s44318-024-00118-0)

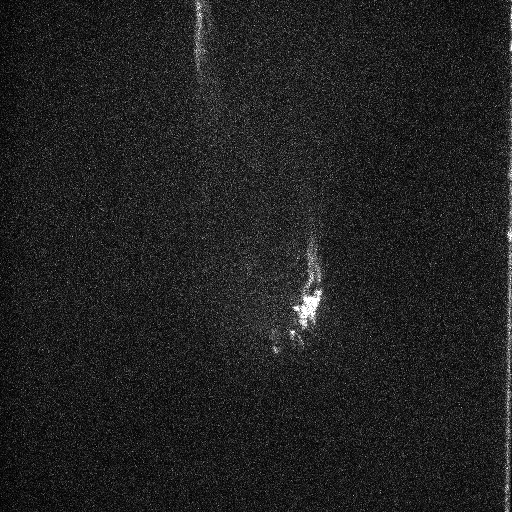

Supplement: Supplementary file 16 — Source data Fig. 7 [file 44318_2024_118_MOESM16_ESM.zip › Figure7/Figure 7B Micr. image/20230424 osm-3-G235A-G444E-GFP; Scarlet-che-3_2 amphid/img_000000000_L-561_015.tif]

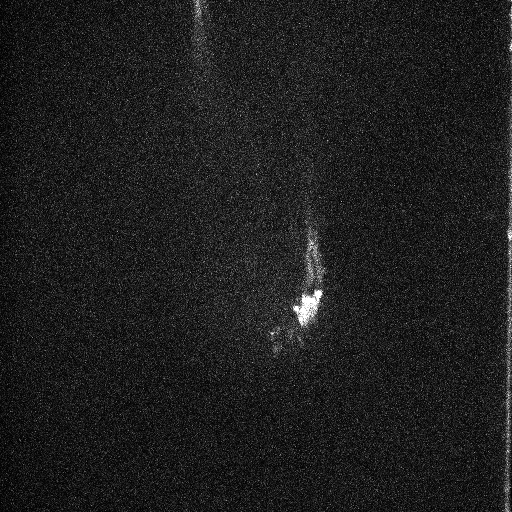

Supplement: Supplementary file 16 — Source data Fig. 7 [file 44318_2024_118_MOESM16_ESM.zip › Figure7/Figure 7B Micr. image/20230424 osm-3-G235A-G444E-GFP; Scarlet-che-3_2 amphid/img_000000000_L-561_016.tif]

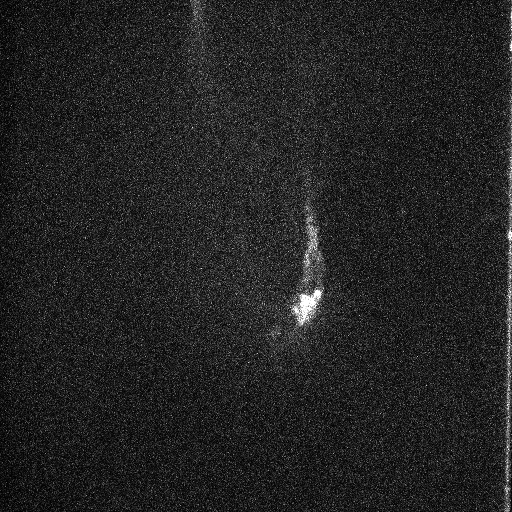

Supplement: Supplementary file 16 — Source data Fig. 7 [file 44318_2024_118_MOESM16_ESM.zip › Figure7/Figure 7B Micr. image/20230424 osm-3-G235A-G444E-GFP; Scarlet-che-3_2 amphid/img_000000000_L-561_017.tif]

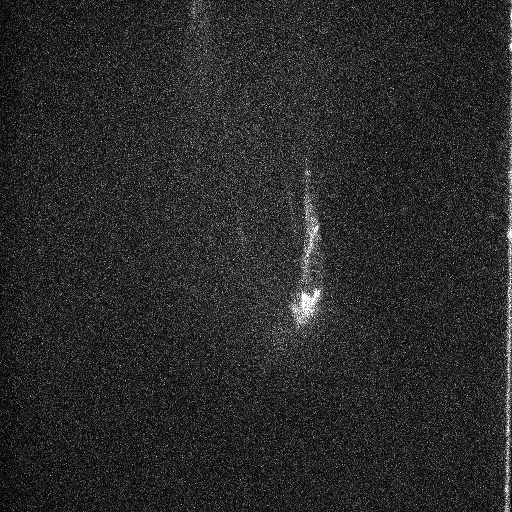

Supplement: Supplementary file 16 — Source data Fig. 7 [file 44318_2024_118_MOESM16_ESM.zip › Figure7/Figure 7B Micr. image/20230424 osm-3-G235A-G444E-GFP; Scarlet-che-3_2 amphid/img_000000000_L-561_018.tif]

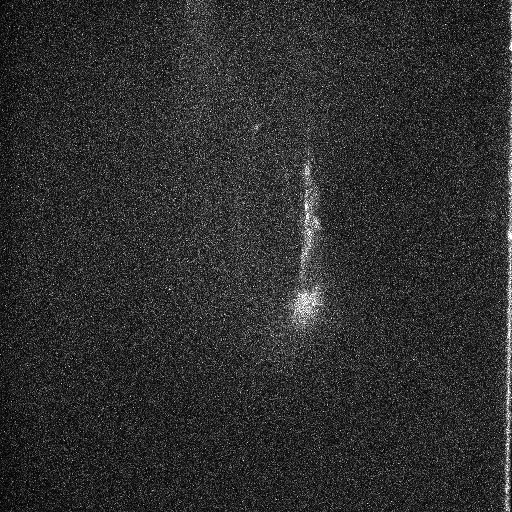

Supplement: Supplementary file 16 — Source data Fig. 7 [file 44318_2024_118_MOESM16_ESM.zip › Figure7/Figure 7B Micr. image/20230424 osm-3-G235A-G444E-GFP; Scarlet-che-3_2 amphid/img_000000000_L-561_019.tif]

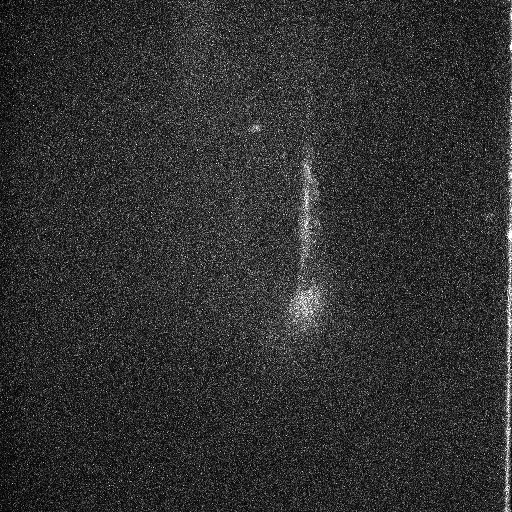

Supplement: Supplementary file 16 — Source data Fig. 7 [file 44318_2024_118_MOESM16_ESM.zip › Figure7/Figure 7B Micr. image/20230424 osm-3-G235A-G444E-GFP; Scarlet-che-3_2 amphid/img_000000000_L-561_020.tif]

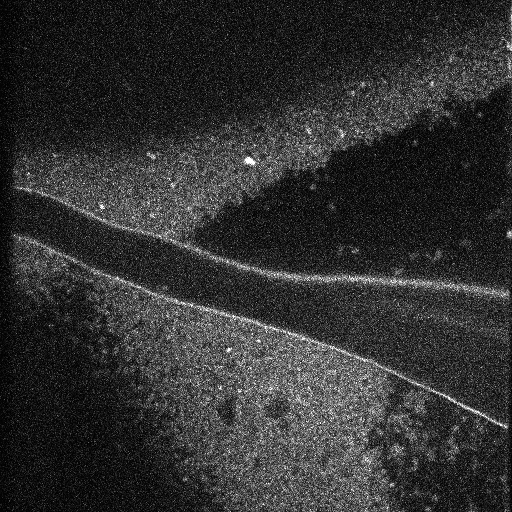

Supplement: Supplementary file 16 — Source data Fig. 7 [file 44318_2024_118_MOESM16_ESM.zip › Figure7/Figure 7B Micr. image/20230424 osm-3-G235A-G444E-GFP; Scarlet-che-3_4 phasmid/img_000000000_L-488_000.tif]

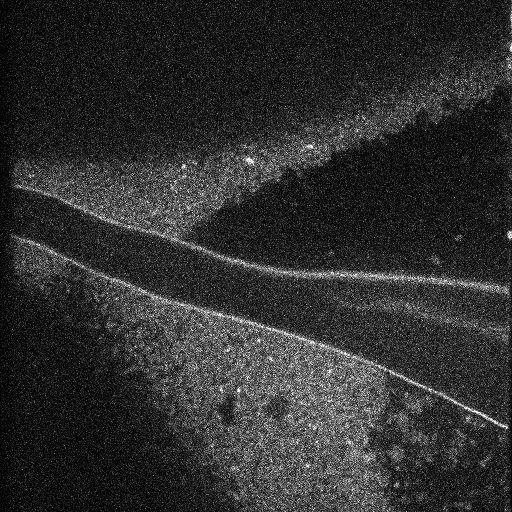

Supplement: Supplementary file 16 — Source data Fig. 7 [file 44318_2024_118_MOESM16_ESM.zip › Figure7/Figure 7B Micr. image/20230424 osm-3-G235A-G444E-GFP; Scarlet-che-3_4 phasmid/img_000000000_L-488_001.tif]

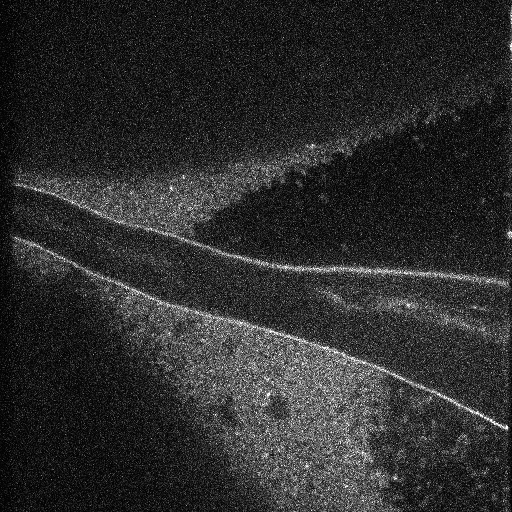

Supplement: Supplementary file 16 — Source data Fig. 7 [file 44318_2024_118_MOESM16_ESM.zip › Figure7/Figure 7B Micr. image/20230424 osm-3-G235A-G444E-GFP; Scarlet-che-3_4 phasmid/img_000000000_L-488_002.tif]

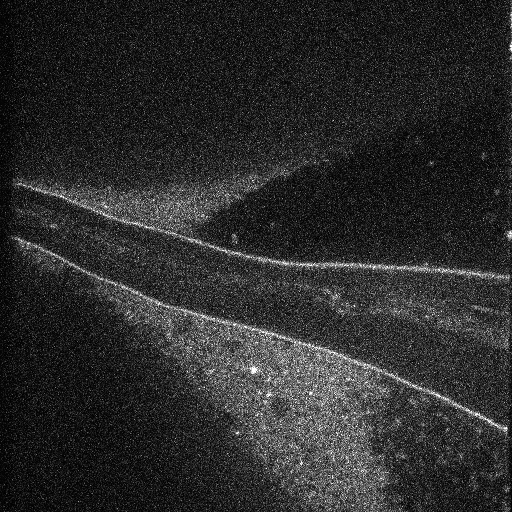

Supplement: Supplementary file 16 — Source data Fig. 7 [file 44318_2024_118_MOESM16_ESM.zip › Figure7/Figure 7B Micr. image/20230424 osm-3-G235A-G444E-GFP; Scarlet-che-3_4 phasmid/img_000000000_L-488_003.tif]

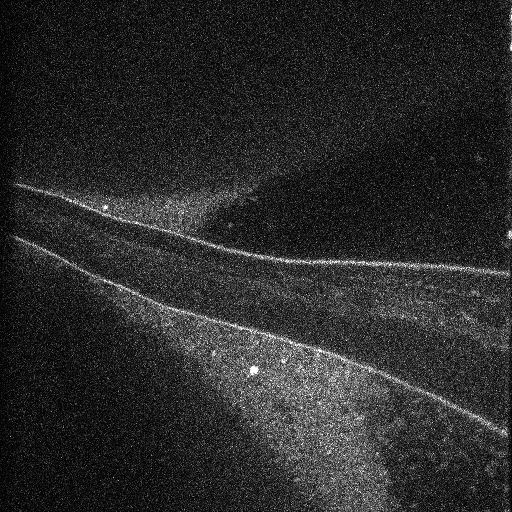

Supplement: Supplementary file 16 — Source data Fig. 7 [file 44318_2024_118_MOESM16_ESM.zip › Figure7/Figure 7B Micr. image/20230424 osm-3-G235A-G444E-GFP; Scarlet-che-3_4 phasmid/img_000000000_L-488_004.tif]

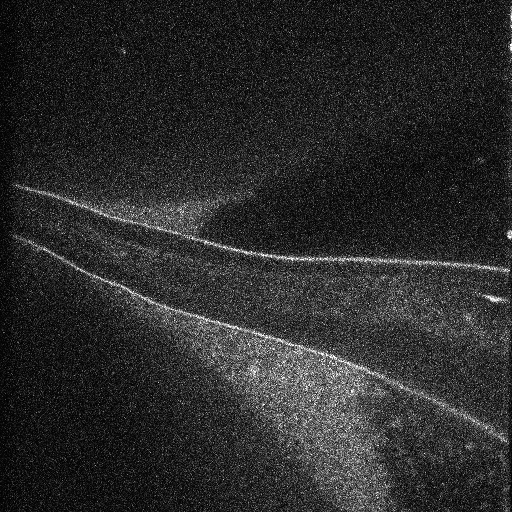

Supplement: Supplementary file 16 — Source data Fig. 7 [file 44318_2024_118_MOESM16_ESM.zip › Figure7/Figure 7B Micr. image/20230424 osm-3-G235A-G444E-GFP; Scarlet-che-3_4 phasmid/img_000000000_L-488_005.tif]

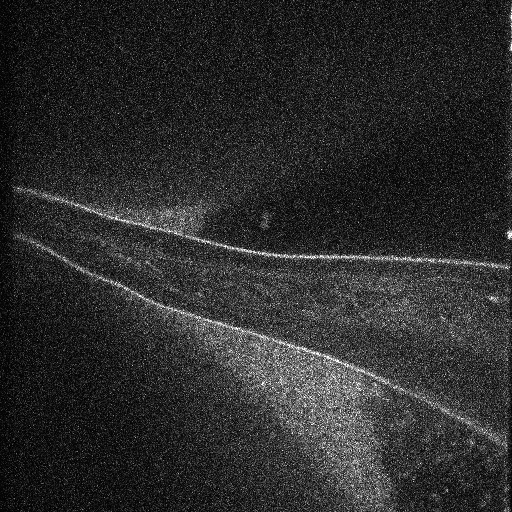

Supplement: Supplementary file 16 — Source data Fig. 7 [file 44318_2024_118_MOESM16_ESM.zip › Figure7/Figure 7B Micr. image/20230424 osm-3-G235A-G444E-GFP; Scarlet-che-3_4 phasmid/img_000000000_L-488_006.tif]

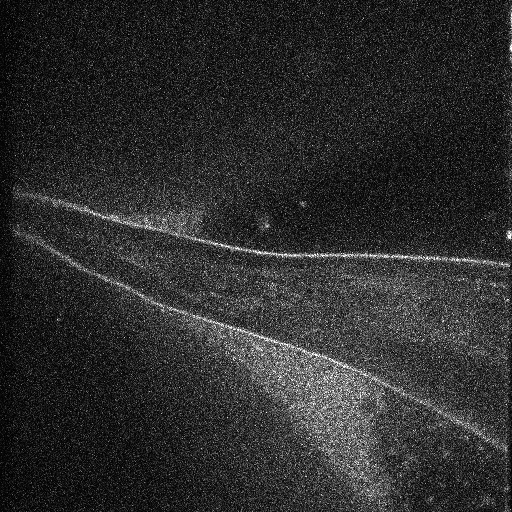

Supplement: Supplementary file 16 — Source data Fig. 7 [file 44318_2024_118_MOESM16_ESM.zip › Figure7/Figure 7B Micr. image/20230424 osm-3-G235A-G444E-GFP; Scarlet-che-3_4 phasmid/img_000000000_L-488_007.tif]

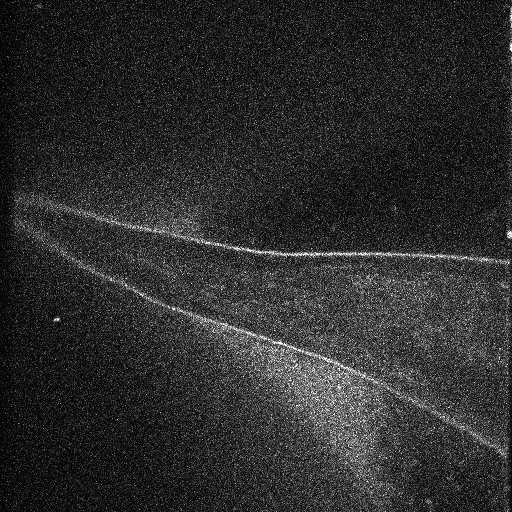

Supplement: Supplementary file 16 — Source data Fig. 7 [file 44318_2024_118_MOESM16_ESM.zip › Figure7/Figure 7B Micr. image/20230424 osm-3-G235A-G444E-GFP; Scarlet-che-3_4 phasmid/img_000000000_L-488_008.tif]

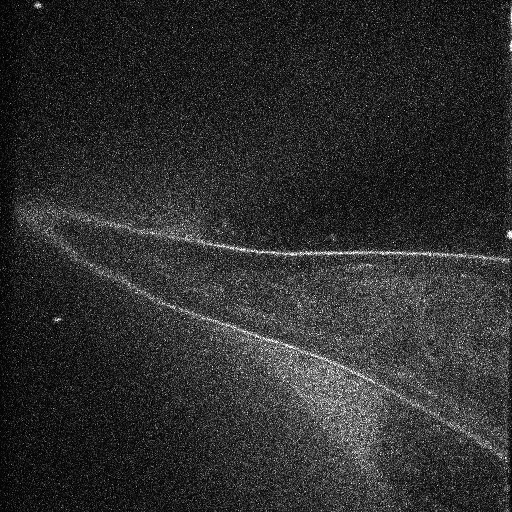

Supplement: Supplementary file 16 — Source data Fig. 7 [file 44318_2024_118_MOESM16_ESM.zip › Figure7/Figure 7B Micr. image/20230424 osm-3-G235A-G444E-GFP; Scarlet-che-3_4 phasmid/img_000000000_L-488_009.tif]

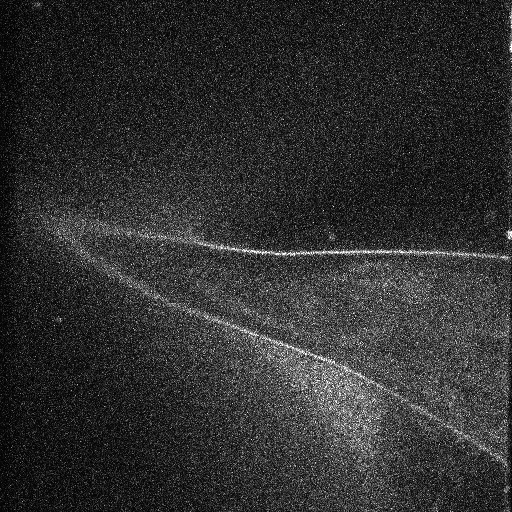

Supplement: Supplementary file 16 — Source data Fig. 7 [file 44318_2024_118_MOESM16_ESM.zip › Figure7/Figure 7B Micr. image/20230424 osm-3-G235A-G444E-GFP; Scarlet-che-3_4 phasmid/img_000000000_L-488_010.tif]

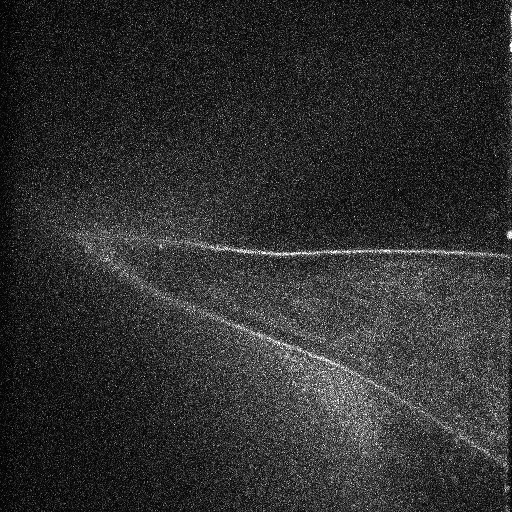

Supplement: Supplementary file 16 — Source data Fig. 7 [file 44318_2024_118_MOESM16_ESM.zip › Figure7/Figure 7B Micr. image/20230424 osm-3-G235A-G444E-GFP; Scarlet-che-3_4 phasmid/img_000000000_L-488_011.tif]

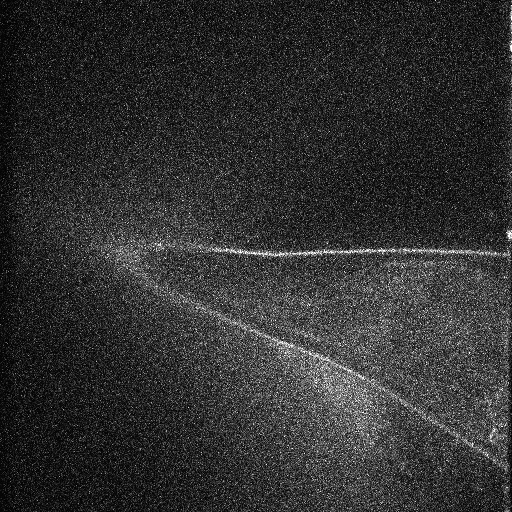

Supplement: Supplementary file 16 — Source data Fig. 7 [file 44318_2024_118_MOESM16_ESM.zip › Figure7/Figure 7B Micr. image/20230424 osm-3-G235A-G444E-GFP; Scarlet-che-3_4 phasmid/img_000000000_L-488_012.tif]

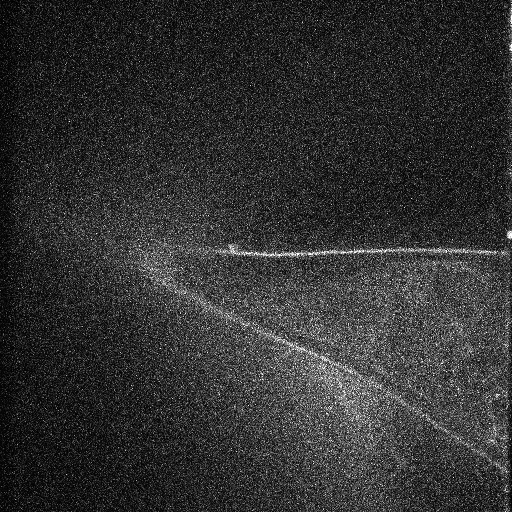

Supplement: Supplementary file 16 — Source data Fig. 7 [file 44318_2024_118_MOESM16_ESM.zip › Figure7/Figure 7B Micr. image/20230424 osm-3-G235A-G444E-GFP; Scarlet-che-3_4 phasmid/img_000000000_L-488_013.tif]

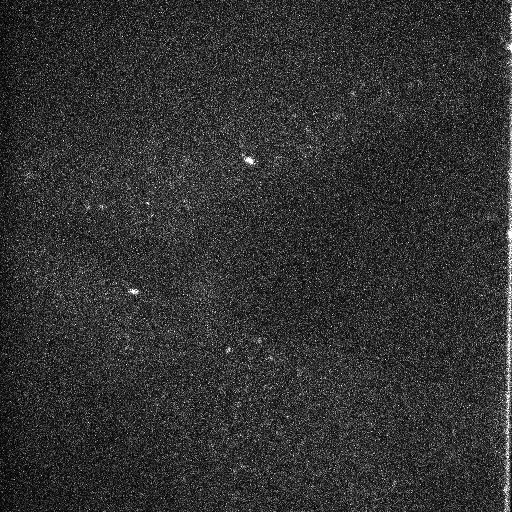

Supplement: Supplementary file 16 — Source data Fig. 7 [file 44318_2024_118_MOESM16_ESM.zip › Figure7/Figure 7B Micr. image/20230424 osm-3-G235A-G444E-GFP; Scarlet-che-3_4 phasmid/img_000000000_L-561_000.tif]

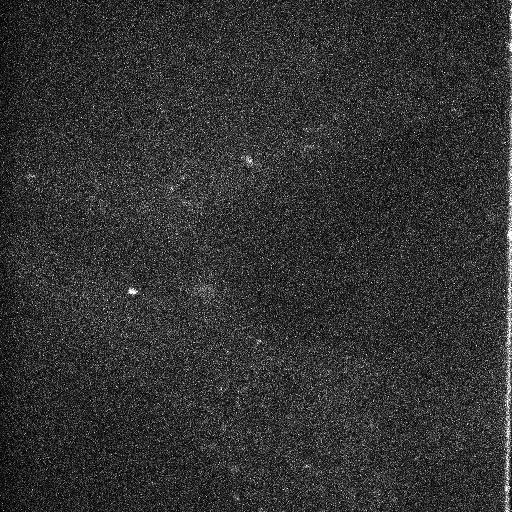

Supplement: Supplementary file 16 — Source data Fig. 7 [file 44318_2024_118_MOESM16_ESM.zip › Figure7/Figure 7B Micr. image/20230424 osm-3-G235A-G444E-GFP; Scarlet-che-3_4 phasmid/img_000000000_L-561_001.tif]

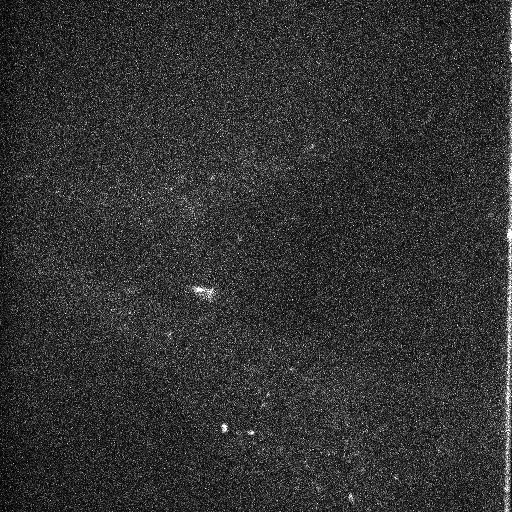

Supplement: Supplementary file 16 — Source data Fig. 7 [file 44318_2024_118_MOESM16_ESM.zip › Figure7/Figure 7B Micr. image/20230424 osm-3-G235A-G444E-GFP; Scarlet-che-3_4 phasmid/img_000000000_L-561_002.tif]

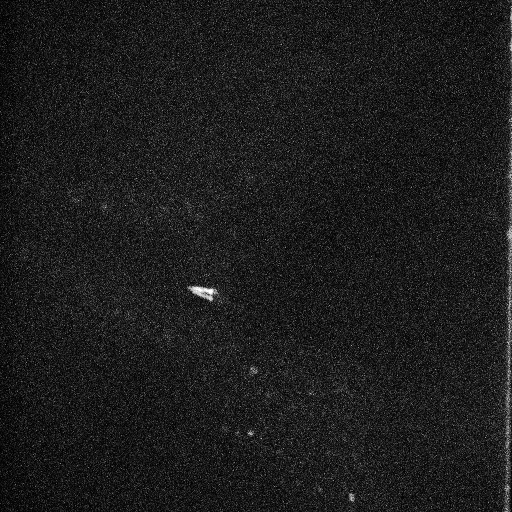

Supplement: Supplementary file 16 — Source data Fig. 7 [file 44318_2024_118_MOESM16_ESM.zip › Figure7/Figure 7B Micr. image/20230424 osm-3-G235A-G444E-GFP; Scarlet-che-3_4 phasmid/img_000000000_L-561_003.tif]

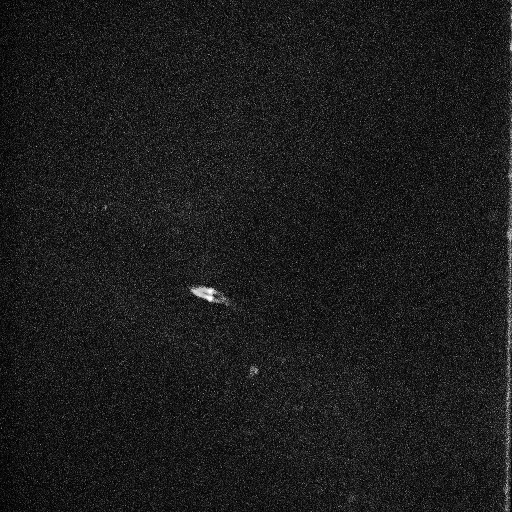

Supplement: Supplementary file 16 — Source data Fig. 7 [file 44318_2024_118_MOESM16_ESM.zip › Figure7/Figure 7B Micr. image/20230424 osm-3-G235A-G444E-GFP; Scarlet-che-3_4 phasmid/img_000000000_L-561_004.tif]

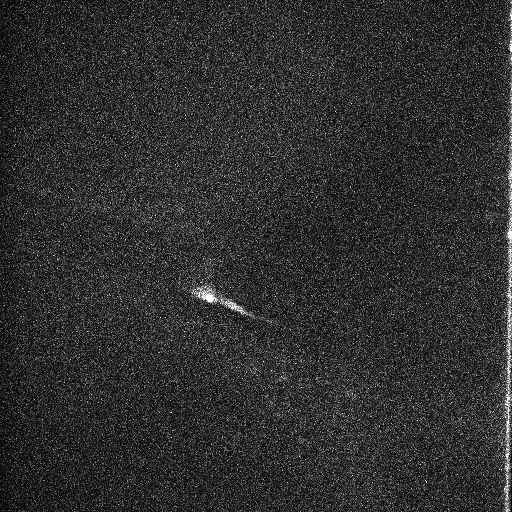

Supplement: Supplementary file 16 — Source data Fig. 7 [file 44318_2024_118_MOESM16_ESM.zip › Figure7/Figure 7B Micr. image/20230424 osm-3-G235A-G444E-GFP; Scarlet-che-3_4 phasmid/img_000000000_L-561_005.tif]

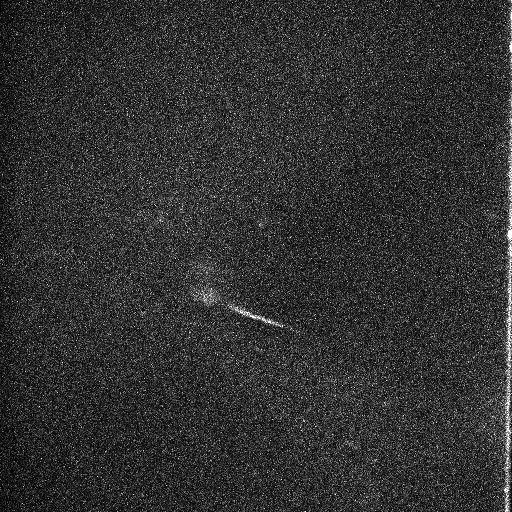

Supplement: Supplementary file 16 — Source data Fig. 7 [file 44318_2024_118_MOESM16_ESM.zip › Figure7/Figure 7B Micr. image/20230424 osm-3-G235A-G444E-GFP; Scarlet-che-3_4 phasmid/img_000000000_L-561_006.tif]

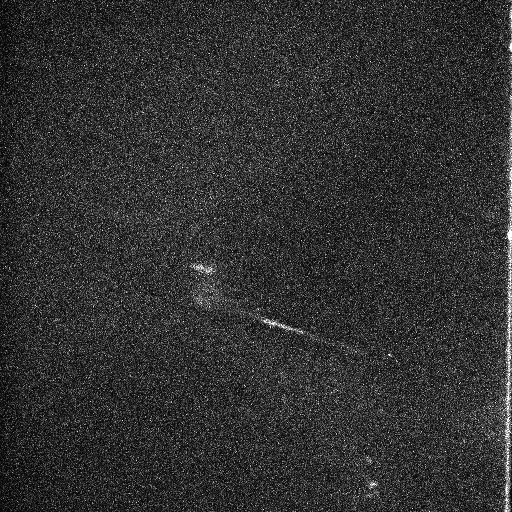

Supplement: Supplementary file 16 — Source data Fig. 7 [file 44318_2024_118_MOESM16_ESM.zip › Figure7/Figure 7B Micr. image/20230424 osm-3-G235A-G444E-GFP; Scarlet-che-3_4 phasmid/img_000000000_L-561_007.tif]

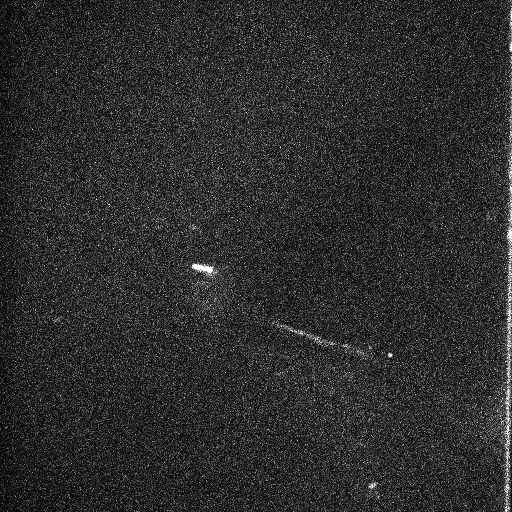

Supplement: Supplementary file 16 — Source data Fig. 7 [file 44318_2024_118_MOESM16_ESM.zip › Figure7/Figure 7B Micr. image/20230424 osm-3-G235A-G444E-GFP; Scarlet-che-3_4 phasmid/img_000000000_L-561_008.tif]

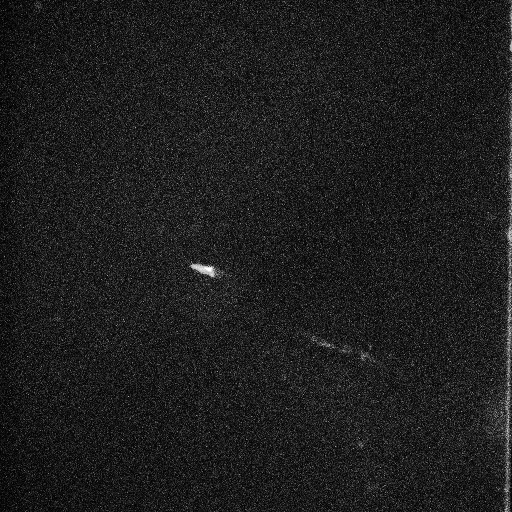

Supplement: Supplementary file 16 — Source data Fig. 7 [file 44318_2024_118_MOESM16_ESM.zip › Figure7/Figure 7B Micr. image/20230424 osm-3-G235A-G444E-GFP; Scarlet-che-3_4 phasmid/img_000000000_L-561_009.tif]

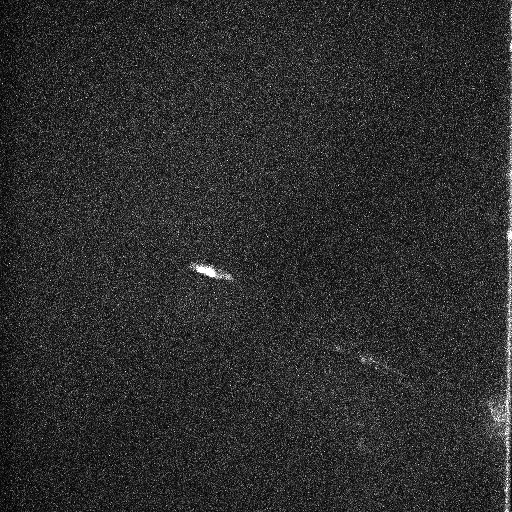

Supplement: Supplementary file 16 — Source data Fig. 7 [file 44318_2024_118_MOESM16_ESM.zip › Figure7/Figure 7B Micr. image/20230424 osm-3-G235A-G444E-GFP; Scarlet-che-3_4 phasmid/img_000000000_L-561_010.tif]

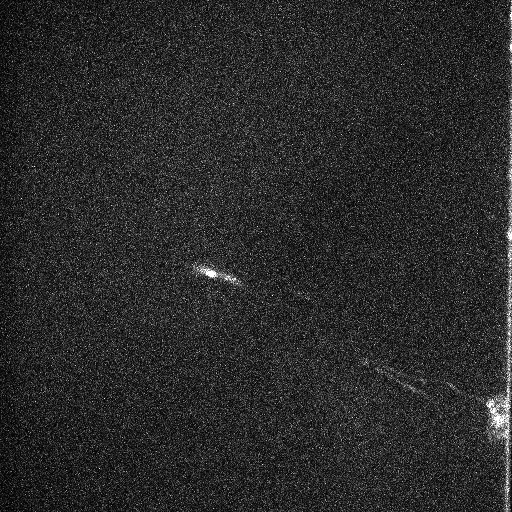

Supplement: Supplementary file 16 — Source data Fig. 7 [file 44318_2024_118_MOESM16_ESM.zip › Figure7/Figure 7B Micr. image/20230424 osm-3-G235A-G444E-GFP; Scarlet-che-3_4 phasmid/img_000000000_L-561_011.tif]

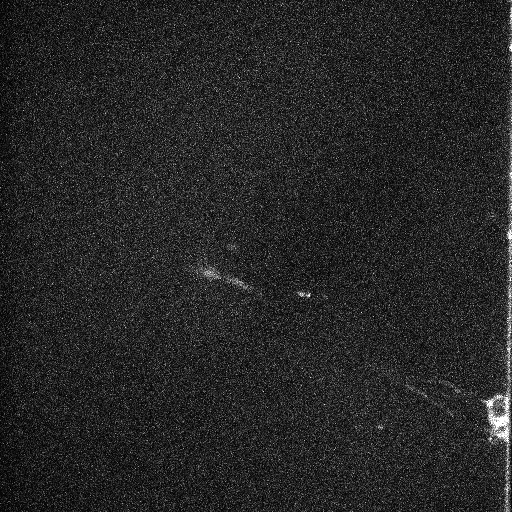

Supplement: Supplementary file 16 — Source data Fig. 7 [file 44318_2024_118_MOESM16_ESM.zip › Figure7/Figure 7B Micr. image/20230424 osm-3-G235A-G444E-GFP; Scarlet-che-3_4 phasmid/img_000000000_L-561_012.tif]

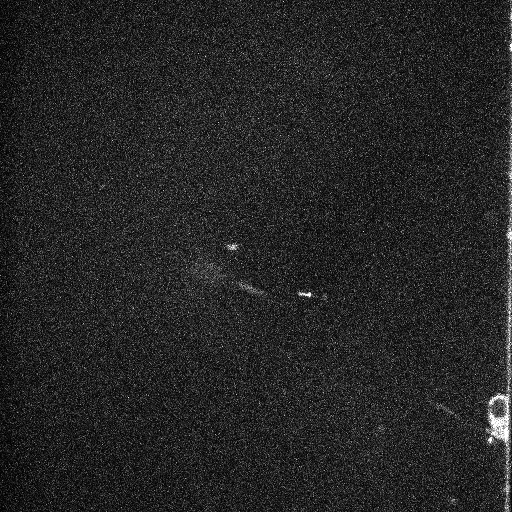

Supplement: Supplementary file 16 — Source data Fig. 7 [file 44318_2024_118_MOESM16_ESM.zip › Figure7/Figure 7B Micr. image/20230424 osm-3-G235A-G444E-GFP; Scarlet-che-3_4 phasmid/img_000000000_L-561_013.tif]

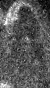

Supplement: Supplementary file 16 — Source data Fig. 7 [file 44318_2024_118_MOESM16_ESM.zip › Figure7/Figure 7B Micr. image/AVG_20230424 osm-3-G235A-G444E-GFP; Scarlet-che-3_2-1 amphid 50x88.tif]

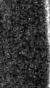

Supplement: Supplementary file 16 — Source data Fig. 7 [file 44318_2024_118_MOESM16_ESM.zip › Figure7/Figure 7B Micr. image/AVG_20230424 osm-3-G235A-G444E-GFP; Scarlet-che-3_4-3 phasmid 50x88.tif]

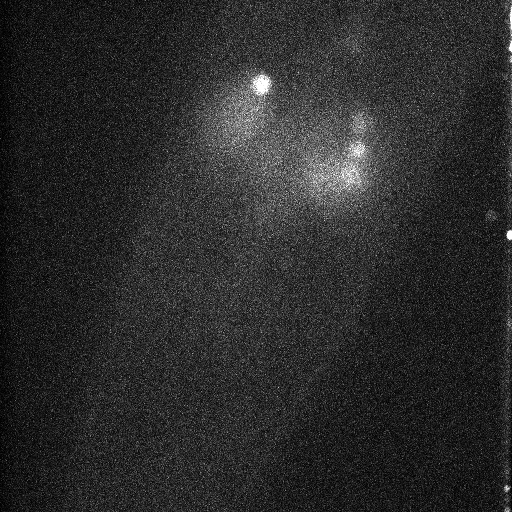

Supplement: Supplementary file 16 — Source data Fig. 7 [file 44318_2024_118_MOESM16_ESM.zip › Figure7/Figure 7C Micr. image/20221227 osm-3-G444E-gfp; Pdyf-1-myri-Scarlet; HIS-54-bfp_3/img_000000000_EX405-EM-GFP_000.tif]

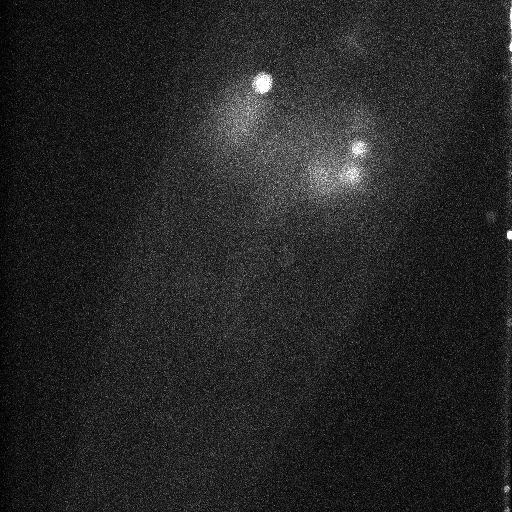

Supplement: Supplementary file 16 — Source data Fig. 7 [file 44318_2024_118_MOESM16_ESM.zip › Figure7/Figure 7C Micr. image/20221227 osm-3-G444E-gfp; Pdyf-1-myri-Scarlet; HIS-54-bfp_3/img_000000000_EX405-EM-GFP_001.tif]

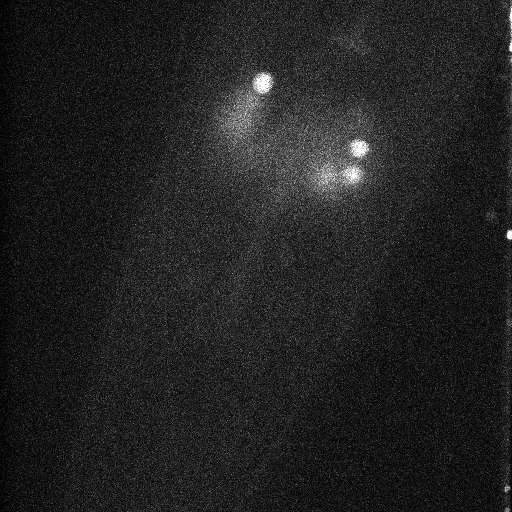

Supplement: Supplementary file 16 — Source data Fig. 7 [file 44318_2024_118_MOESM16_ESM.zip › Figure7/Figure 7C Micr. image/20221227 osm-3-G444E-gfp; Pdyf-1-myri-Scarlet; HIS-54-bfp_3/img_000000000_EX405-EM-GFP_002.tif]

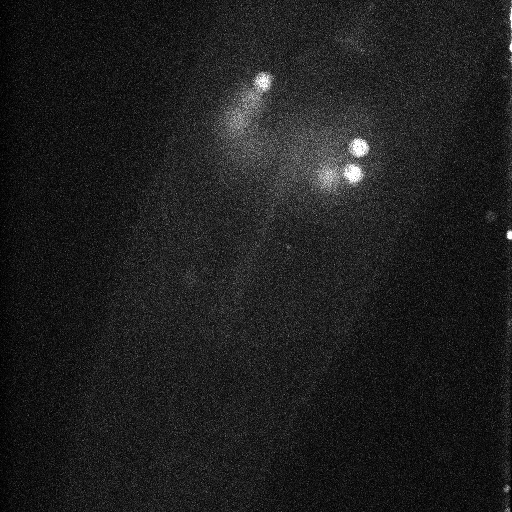

Supplement: Supplementary file 16 — Source data Fig. 7 [file 44318_2024_118_MOESM16_ESM.zip › Figure7/Figure 7C Micr. image/20221227 osm-3-G444E-gfp; Pdyf-1-myri-Scarlet; HIS-54-bfp_3/img_000000000_EX405-EM-GFP_003.tif]
