# Supplementary material for: Neurons dispose of hyperactive kinesin into glial cells for clearance
Source: EMBO J. 2024 May 28;43(13):5. doi: 10.1038/s44318-024-00118-0 (PMC11217292; doi:10.1038/s44318-024-00118-0)
Supplement: Supplementary file 18 — Expanded View Figures [file 44318_2024_118_MOESM18_ESM.pdf]

Expanded View Figures

Figure EV1. The OSM-3-G444E mutation is recessive and loss-of-function, related to Fig. 1.

(**A**, **B**) Frequency distribution of single-molecule velocity of WT and G444E mutant OSM-3. The velocity curve was fitted with a Gaussian distribution. *n* number of single-molecule events measured, v velocity. Data are mean ± SD. (**C**) Representative image of non-fluorescently-tagged OSM-3-G444E in *osm-3(sa125)*, yellow dashed lines showed the pharynx, A, anterior; P, posterior. Scale bar, 10 μm. (**D**) Heterozygotic GFP-KI animals expressing one copy of WT *osm-3* and the other copy of *osm-3(G444E)*. Cilia that are visualized with mScarlet-tagged endogenous DYF-11 are indistinguishable from WT animals. Arrowheads indicate the ciliary base. Arrows indicate the junctions between the middle and distal segments. Scale bar, 5 μm. (**D**) shows GFP fluorescence in cilia whereas (**E**) shows GFP puncta around axons or ejecta outside of sensory neurons. Scale bar, 10 μm. (**F**) Expression of the WT *osm-3* gene under the control of a ciliated neuron-specific promoter P*dyf-1* did not rescue the ejecta formation in *osm-3 (G444E)* mutants. Scale bar, 10 μm. (**G**) Expression profiles at *osm-3* locus in WT and *osm-3(G444E)* strains. Gene body schematic is shown at the bottom. (**H**) Representative RNA editing analysis at *osm-3* locus in WT and *osm-3CA* strains. No editing events were found. The bar showing editing level and gene body schematic are shown above.

 © The Author(s)

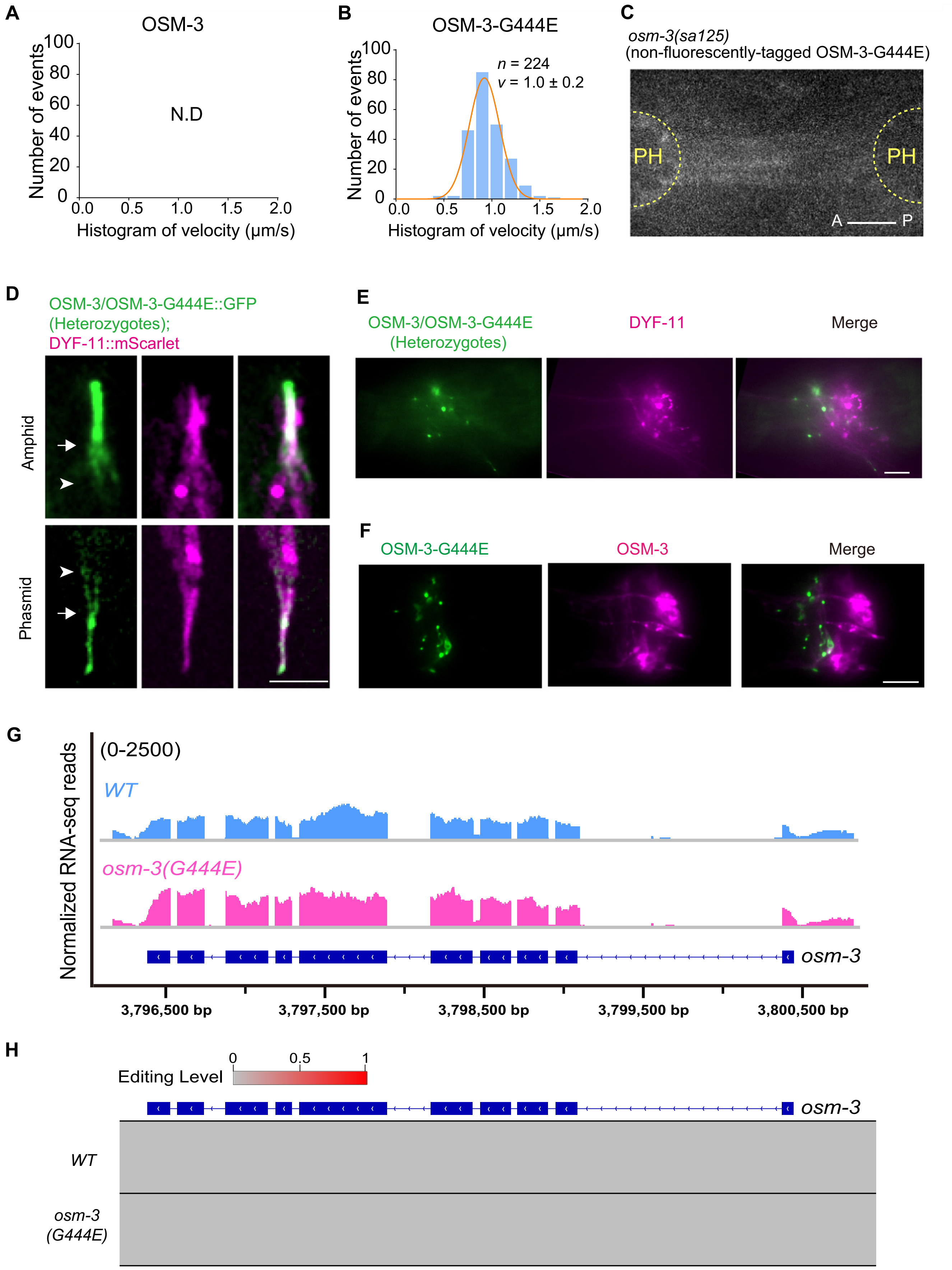
A
OSM-3
Number of events
N.D
Histogram of velocity (μm/s)
B
OSM-3-G444E
n = 224
v = 1.0 ± 0.2
Number of events
Histogram of velocity (μm/s)
C
osm-3(sa125)
(non-fluorescently-tagged OSM-3-G444E)
PH
PH
A P
D
OSM-3/OSM-3-G444E::GFP
(Heterozygotes);
DYF-11::mScarlet
Amphid
Phasmid
E
OSM-3/OSM-3-G444E
(Heterozygotes)
DYF-11
Merge
F
OSM-3-G444E
OSM-3
Merge
G
(0-2500)
WT
osm-3(G444E)
Normalized RNA-seq reads
osm-3
3,796,500 bp
3,797,500 bp
3,798,500 bp
3,799,500 bp
3,800,500 bp
H
Editing Level
0
0.5
1
osm-3
WT
osm-3
(G444E)

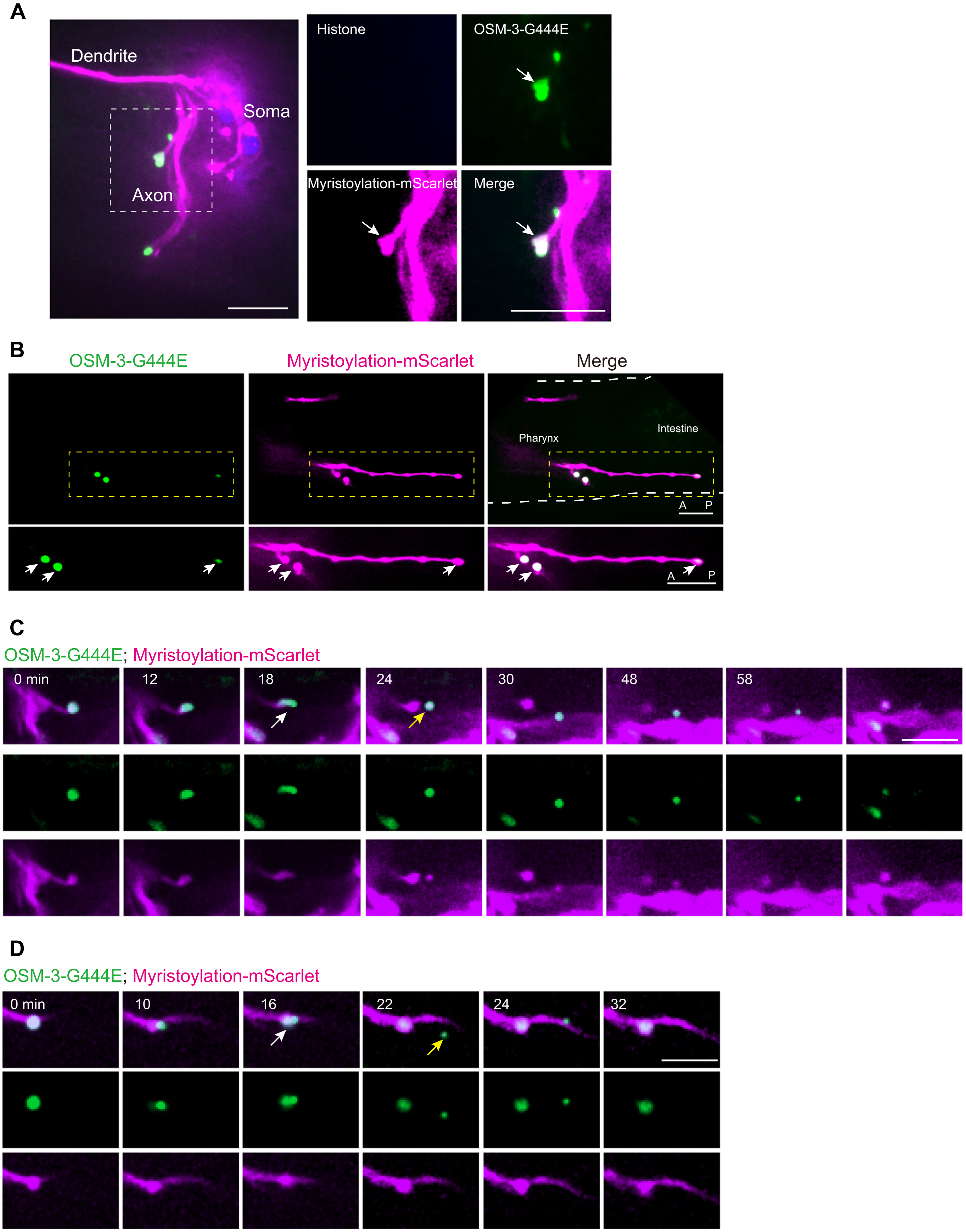
A
Dendrite
Soma
Axon
Histone
OSM-3-G444E
Myristoylation-mScarlet
Merge
B
OSM-3-G444E
Myristoylation-mScarlet
Merge
Pharynx
Intestine
A
P
C
OSM-3-G444E; Myristoylation-mScarlet
0 min
12
18
24
30
48
58
D
OSM-3-G444E; Myristoylation-mScarlet
0 min
10
16
22
24
32

 © The Author(s)

Figure EV2. Localization and disposal of hyperactive OSM-3 at the tips of ectopic neurites, related to Figs. 2 and 3.

(**A**, **B**) Representative fluorescence images of OSM-3-G444E granules at the tips of axons or ectopic neurites. HIS-54::BFP and Myristoylation::mScarlet are expressed under the control of P*dyf-1* in OSM-3-G444E::GFP KI animals. Dashed box is enlarged on the right (**A**) or the bottom (**B**). Arrows, the tip of the neurites where the hyperactive OSM-3 localized. Scale bars, 10 µm. A anterior, P posterior. (**C**, **D**) Fluorescence time-lapse images of endogenous OSM-3-G444E in the sensory neurons. White arrows, the punctum that is about to be released; yellow arrows, the released punctum. Scale bars, 5 µm.

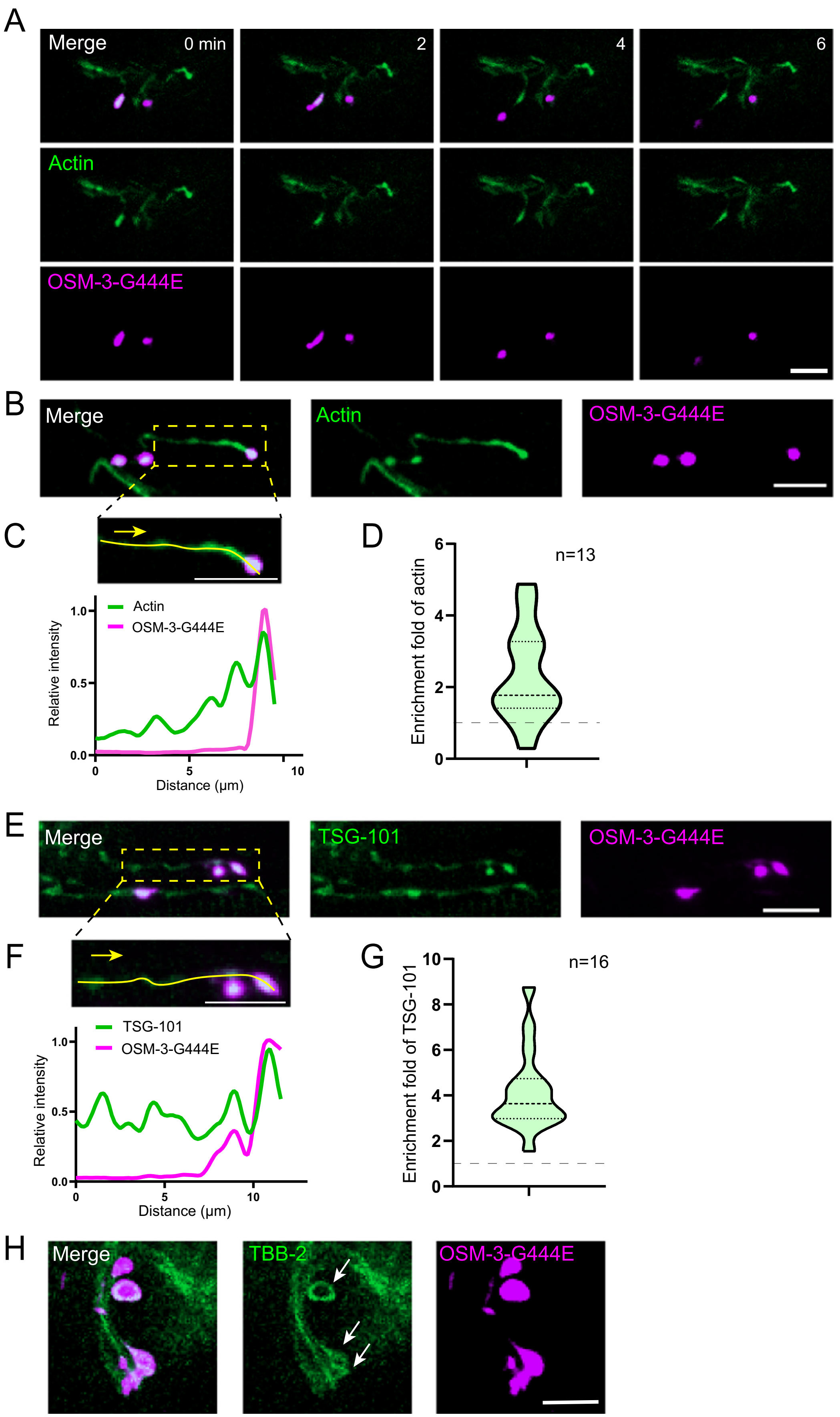
A
Merge
0 min
2
4
6
Actin
OSM-3-G444E
B
Merge
Actin
OSM-3-G444E
C
Actin
OSM-3-G444E
Relative intensity
1.0
0.5
0.0
0
5
10
Distance (μm)
D
n=13
Enrichment fold of actin
6
4
2
0
E
Merge
TSG-101
OSM-3-G444E
F
TSG-101
OSM-3-G444E
Relative intensity
1.0
0.5
0.0
0
5
10
Distance (μm)
G
n=16
Enrichment fold of TSG-101
10
8
6
4
2
0
H
Merge
TBB-2
OSM-3-G444E

 © The Author(s)

Figure EV3. Colocalization of OSM-3-G444E ejecta with actin, ESCRT I component TSG-101 and microtubules.

(**A**) Fluorescence time-lapse images of OSM-3-G444E and its colocalization with actin in the sensory neurons. GFP-tagged MoesinABD is expressed under the control of Pdyf-1 in OSM-3-G444E-mScarlet KI worms. Scale bar, 5 μm. (**B**) Representative fluorescence images of OSM-3-G444E puncta that colocalize with actin. Dashed box is enlarged in (**C**). Scale bar, 5 μm. (**C**) Line scan shows colocalization of OSM-3-G444E-mScarlet and MoesinABD labeled actin. Relative intensities are plotted using normalized gray values along the scanning line. Yellow arrow shows the scanning direction on the scanning line. Scale bar, 5 μm. (**D**) Enrichment fold of MoesinABD labeled actin. Enrichment fold = [(Mean Gray Value of Colocalized Area)-(Background Gray Value)]/ [(Mean Gray Value of Non-Colocalized Neurites)-(Background Gray Value)]. *n* number of events analyzed. (**E**) Representative fluorescence images of OSM-3-G444E puncta that colocalize with GFP-tagged TSG-101. Dashed box is enlarged in (**F**). Scale bar, 5 μm. (**F**) Line scan shows colocalization of OSM-3-G444E-mScarlet and GFP::TSG-101. Relative intensities are plotted using normalized gray values along the scanning line. Yellow arrow shows the scanning direction on the scanning line. Scale bar, 5 μm. (**G**) Enrichment fold of GFP::TSG-101. *n* number of events analyzed. (**H**) Representative fluorescence images of OSM-3-G444E puncta that colocalize with TBB-2, TBB-2 forms ring-like structures at the edges of the puncta. Scale bar, 5 μm.

© The Author(s)

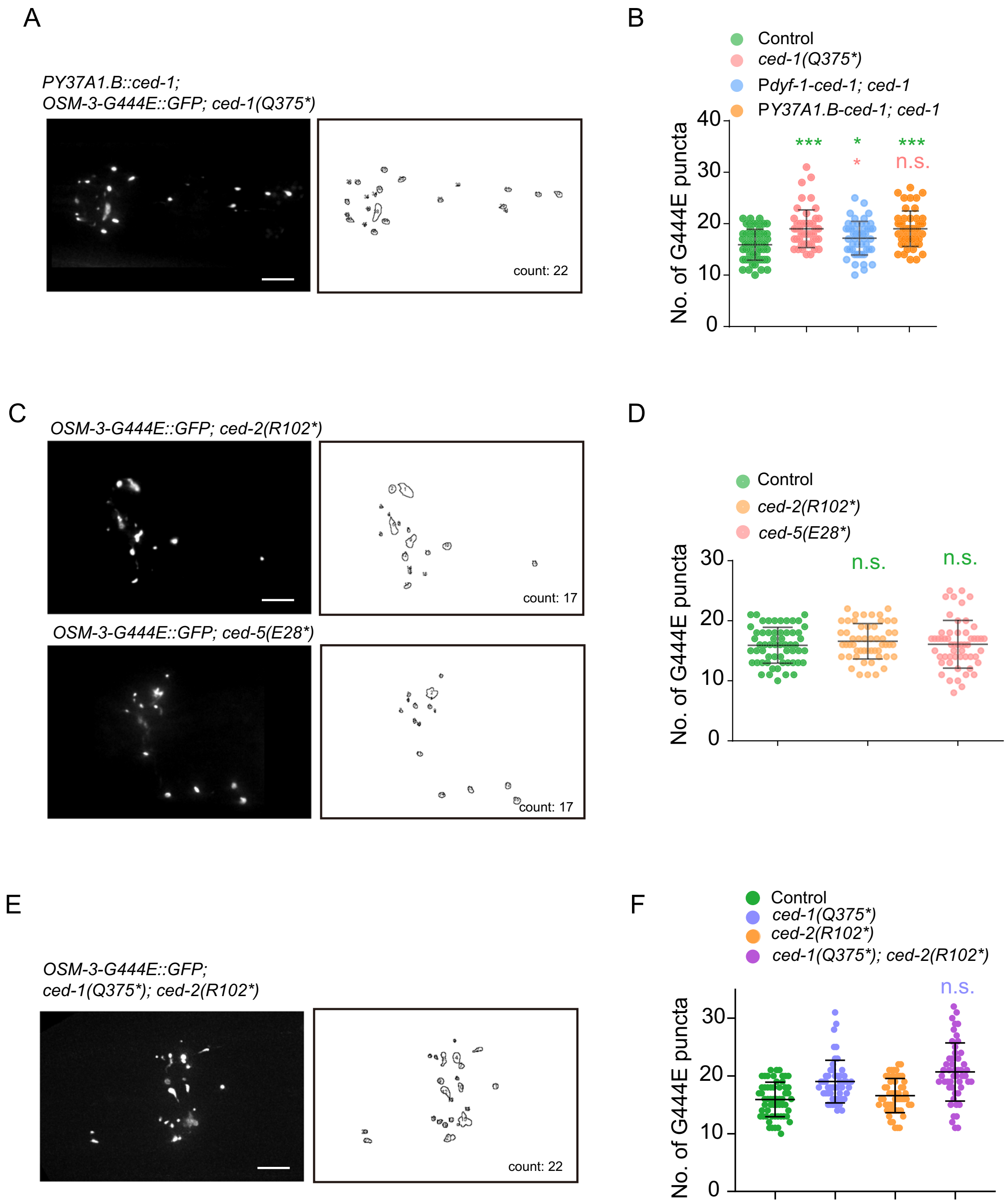

Figure EV4. ***ced-2* and *ced-5* are not involved in the clearance of OSM-3-G444E ejecta.**

(**A**) Representative fluorescence image of OSM-3-G444E ejecta in *ced-1(Q375Stop)* mutants with the overexpression of wild-type *ced-1* under the control of PY37A1.B. Count, the number of ejecta. Scale bar, 10 μm. (**B**) Quantification of the number of OSM-3-G444E ejecta in the strains shown in (**A**), $n > 50$ worms were analyzed for each strain. Data are mean ± SD. (**C**) Representative fluorescence image of OSM-3-G444E ejecta in *ced-2(R102Stop)* or *ced-5(E28Stop)* strains. (**D**) Quantification of the number of OSM-3-G444E ejecta in the strains shown in (**C**), $n > 50$ worms were analyzed for each strain. Data are mean ± SD. (**E**) Representative fluorescence image of OSM-3-G444E ejecta in *ced-1(Q375Stop); ced-2(R102Stop)* double mutant. (**F**) Quantification of the number of OSM-3-G444E ejecta in the strains shown in (**E**), $n > 50$ worms were analyzed for each strain. Data are mean ± SD. ***$P < 0.001$, *$P < 0.05$, n.s. not significant, by one-way ANOVA using BH method to adjust P values.

 © The Author(s)

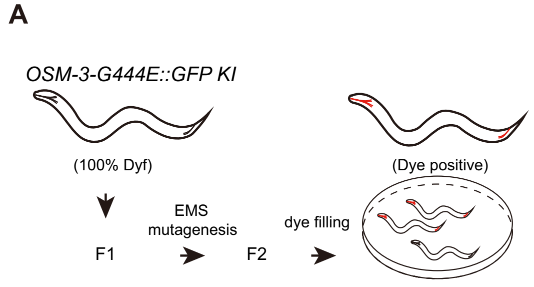

B

Gene	Allele	Mutation	Codon Change
**osm-3**	*cas11134*	H132Y	C/TAC
	cas1611	H207Q	CAC/A
	cas1696	E215K	G/AAA
	cas1612	R238W	C/TGG
	cas1614	R238W	C/TGG
	cas1621	R238W	C/TGG
	cas1629	R238W	C/TGG
	cas1634	R238W	C/TGG
	cas1646	R238W	C/TGG
	cas1653	R238W	C/TGG
	cas11108	R238W	C/TGG
	cas11157	K250E	A/GAG
	cas1650	T285I	AC/TT
**dyf-5**	*cas1622*	V85G	GT/GG
	cas1610	P135S	C/TCC
	cas1651	T164P	A/CCA
	cas1632	A174T	G/ACA
	cas1628	P175S	C/TCT
	cas1639	A192T	G/ACA
	cas1644	L200H	CT/AT
	cas1658	L200H	CT/AT
	cas11134	H132Y	C/TAC

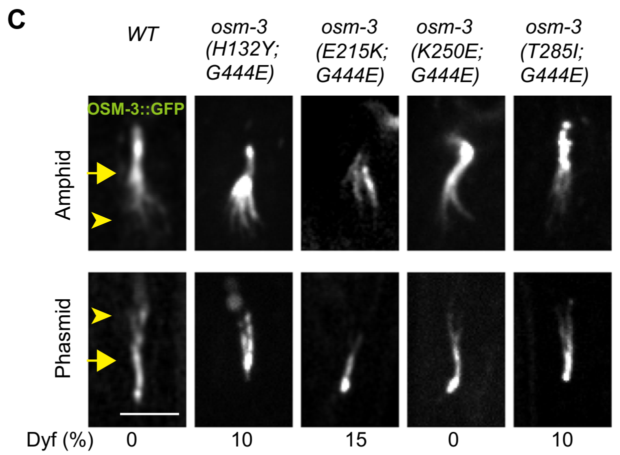

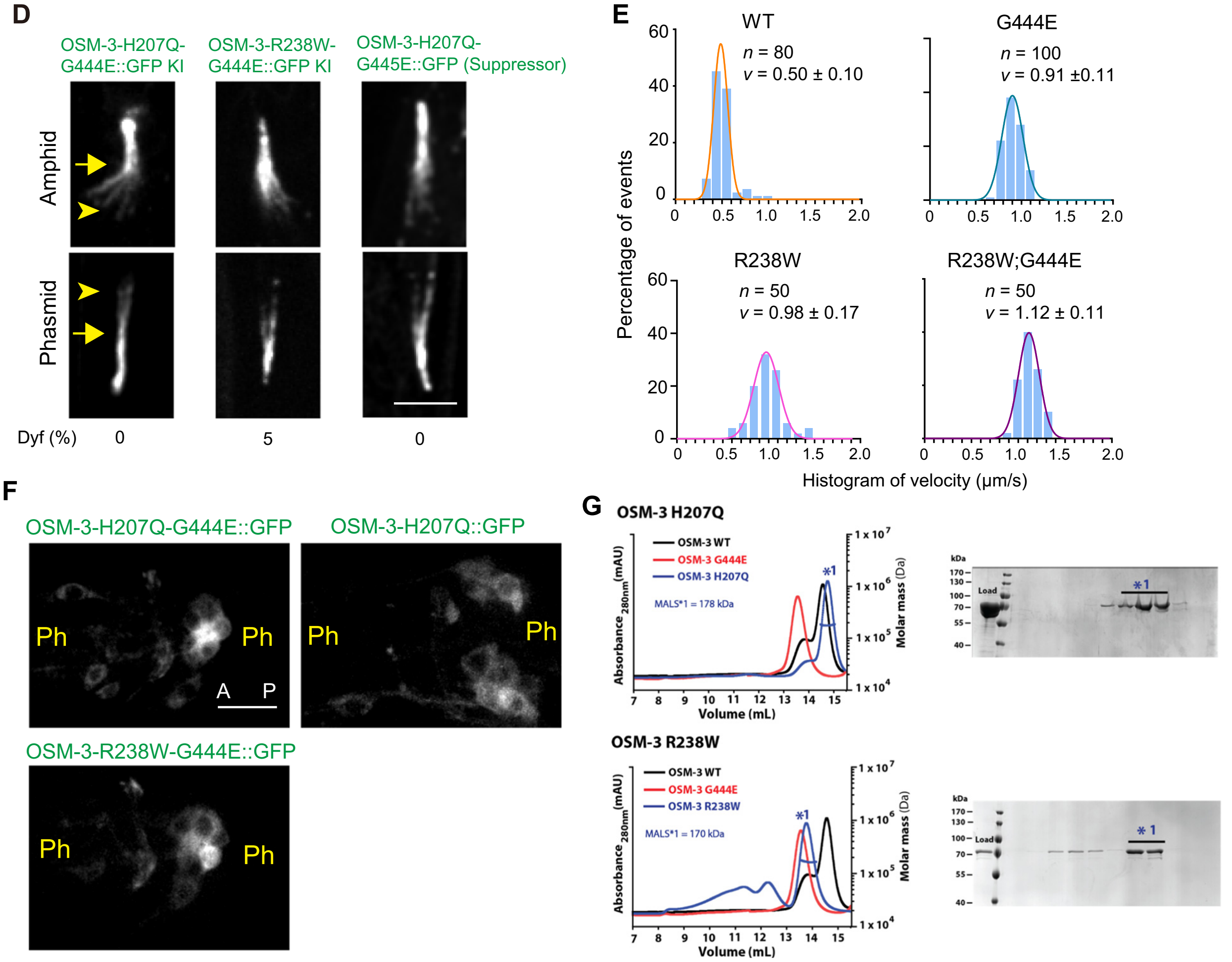

Figure EV5. Intragenic mutations that rescue the ciliary phenotypes in *osm-3(G444E)* mutant and restore the localization of endogenous OSM-3-G444E.

(**A**) Flowchart of the suppressor screening. OSM-3-G444E::GFP KI animals at the late L4 stage were treated with ethyl methanesulfonate (EMS). By filling with the fluorescent dye DiI, dye-positive F2 progenies were considered putative suppressors. About 10^5 haploid genomes were screened in 5 rounds of screenings. The localization of OSM-3-G444E were examined via confocal microscopy. Mutant genes were cloned by the whole-genome sequencing. (**B**) Molecular lesions of the *osm-3* and *dyf-5* suppressor alleles. (**C**) Amphid and phasmid cilia in WT and the suppressors harbor the mutations shown in Fig. 5A, B. GFP-tagged endogenous WT OSM-3 or the corresponding mutant OSM-3 are shown. Dyf, dye-filling defective; N ≥ 100. Arrowheads indicate the ciliary base. Arrows indicate the junctions between the middle and distal segments. The WT image is from Fig. 5C. Scale bar, 5 μm. (**D**) Distribution of endogenous OSM-3-H207Q-G444E (left), OSM-3-R238W-G444E (middle) and OSM-3-H207Q-G445E (right) in sensory cilia. For the strain shown on the left and in the middle, H207Q or R238W mutation was generated in OSM-3-G444E::GFP KI animals by CRISPR/Cas9-triggered genome editing. The OSM-3-H207Q-G445E strain shown on the right was obtained from a suppressor screen restoring ciliary defects caused by H207Q mutation in OSM-3-H207Q::GFP KI worms. Dyf, dye-filling defective; *N* ≥ 100. Arrowheads indicate the ciliary base. Arrows indicate the junctions between the middle and distal segments. Scale bar, 5 μm. (**E**) Frequency distribution of MT gliding velocities showed in Fig. 5G. The velocity distribution curves were fitted with a Gaussian distribution. *n* number of MTs measured, v velocity. Data are mean ± SD. (**F**) Distribution of the endogenous OSM-3-H207Q-G444E (top left), OSM-3-H207Q (top right) and OSM-3-R238W-G444E (bottom left) in the soma of sensory neurons. Ph, pharynx. Scale bar, 10 μm. A anterior, P posterior. (**G**) Left, overlays of the elution profiles of WT (line in black), G444E (red) and H207Q or R238W mutant (blue) OSM-3. The molar mass of OSM-3-H207Q or R238W determined from the MALS fit is shown on the left; right, SDS–PAGE analyses to identify the elution peak *1 shown on the left. Protein constituents were determined by subsequent liquid chromatography-tandem mass spectrometry (LC-MS/MS) analysis.

 © The Author(s)
